# Supplementary material for: SPROUTS_DB: An Implemented Database of Contaminants for Extracellular Vesicle Proteomics Studies
Source: Proteomics. 2026 Apr 1;26(7):69–83. doi: 10.1002/pmic.70128 (PMC13327711; doi:10.1002/pmic.70128)
Supplement: Supplementary file 2 — Supporting File 2: pmic70128‐sup‐0002‐SuppMat2.docx. [file PMIC-26--s001.docx]

>sp|Q58DD0|ACE2_BOVIN Angiotensin-converting enzyme 2 OS=Bos taurus OX=9913 GN=ACE2 PE=1 SV=1

MTGSFWLLLSLVAVTAAQSTTEEQAKTFLEKFNHEAEDLSYQSSLASWNYNTNITDENVQ

KMNEARAKWSAFYEEQSRMAKTYSLEEIQNLTLKRQLKALQHSGTSALSAEKSKRLNTIL

NKMSTIYSTGKVLDPNTQECLALEPGLDDIMENSRDYNRRLWAWEGWRAEVGKQLRPLYE

EYVVLENEMARANNYEDYGDYWRGDYEVTGAGDYDYSRDQLMKDVERTFAEIKPLYEQLH

AYVRAKLMHTYPSYISPTGCLPAHLLGDMWGRFWTNLYSLTVPFEHKPSIDVTEKMENQS

WDAERIFKEAEKFFVSISLPYMTQGFWDNSMLTEPGDGRKVVCHPTAWDLGKGDFRIKMC

TKVTMDDFLTAHHEMGHIQYDMAYAAQPYLLRNGANEGFHEAVGEIMSLSAATPHYLKAL

GLLAPDFHEDNETEINFLLKQALTIVGTLPFTYMLEKWRWMVFKGEIPKQQWMEKWWEMK

REIVGVVEPLPHDETYCDPACLFHVAEDYSFIRYYTRTIYQFQFHEALCKTAKHEGALFK

CDISNSTEAGQRLLQMLRLGKSEPWTLALENIVGIKTMDVKPLLNYFEPLFTWLKEQNRN

SFVGWSTEWTPYSDQSIKVRISLKSALGENAYEWNDNEMYLFQSSVAYAMRKYFSEARNE

TVLFGEDNVWVSDKKPRISFKFFVTSPNNVSDIIPRTEVENAIRLSRDRINDVFQLDDNS

LEFLGIQPTLGPPYEPPVTIWLIIFGVVMGVVVIGIVVLIFTGIRNRRKKNQASSEENPY

GSVDLNKGENNSGFQNIDDVQTSL

>sp|P11064|PPAC_BOVIN Low molecular weight phosphotyrosine protein phosphatase OS=Bos taurus OX=9913 GN=ACP1 PE=1 SV=3

MAEQVTKSVLFVCLGNICRSPIAEAVFRKLVTDQNISDNWVIDSGAVSDWNVGRSPDPRA

VSCLRNHGINTAHKARQVTKEDFVTFDYILCMDESNLRDLNRKSNQVKNCRAKIELLGSY

DPQKQLIIEDPYYGNDADFETVYQQCVRCCRAFLEKVR

>sp|A7MB62|ARP2_BOVIN Actin-related protein 2 OS=Bos taurus OX=9913 GN=ACTR2 PE=1 SV=1

MDSQGRKVVVCDNGTGFVKCGYAGSNFPEHIFPALVGRPIIRSTTKVGNIEIKDLMVGDE

ASELRSMLEVNYPMENGIVRNWDDMKHLWDYTFGPEKLNIDTRNCKILLTEPPMNPTKNR

EKIVEVMFETYQFSGVYVAIQAVLTLYAQGLLTGVVVDSGDGVTHICPVYEGFSLPHLTR

RLDIAGRDITRYLIKLLLLRGYAFNHSADFETVRMIKEKLCYVGYNIEQEQKLALETTVL

VESYTLPDGRIIKVGGERFEAPEALFQPHLINVEGVGVAELLFNTIQAADIDTRSEFYKH

IVLSGGSTMYPGLPSRLERELKQLYLERVLKGDVEKLSKFKIRIEDPPRRKHMVFLGGAV

LADIMKDKDNFWMTRQEYQEKGVRVLEKLGVTVR

>sp|P20000|ALDH2_BOVIN Aldehyde dehydrogenase, mitochondrial OS=Bos taurus OX=9913 GN=ALDH2 PE=1 SV=2

MLRAVALAAARLGPRQGRRLLSAATQAVPTPNQQPEVLYNQIFINNEWHDAVSKKTFPTV

NPSTGDVICHVAEGDKADVDRAVKAARAAFQLGSPWRRMDASERGRLLNRLADLIERDRT

YLAALETLDNGKPYIISYLVDLDMVLKCLRYYAGWADKYHGKTIPIDGDYFSYTRHEPVG

VCGQIIPWNFPLLMQAWKLGPALATGNVVVMKVAEQTPLTALYVANLIKEAGFPPGVVNV

IPGFGPTAGAAIASHEDVDKVAFTGSTEVGHLIQVAAGKSNLKRVTLELGGKSPNIIMSD

ADMDWAVEQAHFALFFNQGQCCCAGSRTFVQEDIYAEFVERSVARAKSRVVGNPFDSRTE

QGPQVDETQFKKVLGYIKSGKEEGAKLLCGGGAAADRGYFIQPTVFGDVQDGMTIAKEEI

FGPVMQILKFKSMEEVVGRANNSKYGLAAAVFTKDLDKANYLSQALQAGTVWVNCYDVFG

AQSPFGGYKLSGSGRELGEYGLQAYTEVKTVTVRVPQKNS

>sp|Q3T0S5|ALDOB_BOVIN Fructose-bisphosphate aldolase B OS=Bos taurus OX=9913 GN=ALDOB PE=2 SV=1

MAHQFPALTSEQKKALSETARRIVANGKGILAADESVGTMGNRLQRIKVENTEENRRQFR

ELLFTVDSSVSQSIGGVILFHETLYQKDGQGKLFRDILKEKGIVVGIKLDQGVAPLAGTN

KETTVQGLDGLSERCAQYKKDGADFGKWRAVLKIDNQCPSHLAIQENANTLARYASICQQ

NGLVPIVEPEVIPDGSHDMEHCQYVTEKVLAAVYKALNDHHVYLEGTLLKPNMVTAGHAC

TKKYTPEQVAMATVTALHRTVPAAVPGICFLSGGMSEEDATLNLNAINLCPLPKPWKLSF

SYGRALQASALAAWGGKAENKKTTQEAFMKRALANSQAAKGQYVHMGSSGSASTQSLFTA

SYTY

>sp|P27214|ANX11_BOVIN Annexin A11 OS=Bos taurus OX=9913 GN=ANXA11 PE=1 SV=1

MSYPGYPPPAGGYPPGAPGGGAWGGAGYPPPTMPPIGLDNVANYAGQFNQDYLSGVAANM

SGTFGGANVPNLYPGAPGGGYPPVPPGGFGQPPPAQQPVPSYGMYPPPGGNPTSGMPSYP

PYPGAPVPGQPMLPPGQQPPGVYPGQPPMTYPGQSPVPPPGQQPVPSYPGYSGSGTVTPA

VSPAQFGNRGTITDASGFDPLRDAEVLRKAMKGFGTDEQAIIDCLGSRSNKQRQQILLSF

KTAYGKDLIKDLKSELSGNFEKTILALMKTPVLFDAYEIKEAIKGAGTDEACLIEILASR

SNEHIRELNRVYKTEFKKTLEEAIRSDTSGHFQRLLISLSQGNRDESTNVDMTLVQRDVQ

ELYAAGENRLGTDESKFNAILCSRSRAHLVAVFNEYQRMTGRDIEKSICREMSGDLEQGM

LAVVKCLKNTPAFFAERLNKAMRGAGTKDRTLIRIMVSRSEIDLLDIRAEYKRLYGKSLY

HDITGDTSGDYRKILLKICGGND

>sp|P04272|ANXA2_BOVIN Annexin A2 OS=Bos taurus OX=9913 GN=ANXA2 PE=1 SV=2

MSTVHEILCKLSLEGDHSTPPSAYGSVKAYTNFDAERDALNIETAIKTKGVDEVTIVNIL

TNRSNEQRQDIAFAYQRRTKKELASALKSALSGHLETVILGLLKTPAQYDASELKASMKG

LGTDEDSLIEIICSRTNQELQEINRVYKEMYKTDLEKDIVSDTSGDFRKLMVALAKGRRA

EDGSVIDYELIDQDARDLYDAGVKRKGTDVPKWISIMTERSVCHLQKVFERYKSYSPYDM

LESIKKEVKGDLENAFLNLVQCIQNKPLYFADRLYDSMKGKGTRDKVLIRIMVSRSEVDM

LKIRSEFKKKYGKSLYYYIQQDTKGDYQKALLYLCGGDD

>sp|Q3SWX7|ANXA3_BOVIN Annexin A3 OS=Bos taurus OX=9913 GN=ANXA3 PE=2 SV=3

MASIWVGKRGTIRDYAGFNPSVDAEAIRKAIRGIGTDEKTLISILTERTNAQRLLIAKEY

QALCGKELKDDLKGDLSGHFKHLMVALVTPPAVFDAKQLKKSMKGMGTNEDALIEILTTR

TSKQMQEIGHAYYTAYKKSLGDEISSETSGDFRKALLILANGRRDESLKVDEQLARKDAQ

ILYNAGEKRWGTDEDAFTNILCLRSFPQLKLTFDEYRNISQKDIEDSIKGELSGHFEDLL

LAIVRCARNTPAFLAERLYRALKGAGTDEFTLNRIMVSRSEIDLLDIRAEFKKLSGYSLY

SAIKSDTSGDYEITLLKICGGDD

>sp|P81287|ANXA5_BOVIN Annexin A5 OS=Bos taurus OX=9913 GN=ANXA5 PE=1 SV=3

MAQVLRGTVADFPGFDERADAETLRKAMKGLGTDEETILTLLTSRSNAQRQEIAVAFKTL

FGRDLLDDLKSELTGKFEKLIVALMKPSRLYDAYELKHALKGAGTDEKVLTEIIASRTPE

ELRAIEQVYEEEYGSSLEDDVVGDTSGYYQRMLVVLLQANRDPDARIDEAQVEQDAQALF

QAGELKWGTDEEKFITIFGTRSVSHLRRVFDKYMTISGFQIEETIDRETSGNLEQLLLAV

VKSIRSIPAYLAETLYYAMKGAGTDDHTLIRVVVSRSEIDLYNIRKEFRKNFGTSLYSMI

KGDTSGDYKKALLLLCGGEDD

>sp|P79134|ANXA6_BOVIN Annexin A6 OS=Bos taurus OX=9913 GN=ANXA6 PE=1 SV=2

MAKPAQGAKYRGSIRDFPDFNPSQDAETLYNAMKGFGSDKEAILELITSRSNRQRQEICQ

NYKSLYGKDLIADLKYELTGKFERLIVGLMRPPAYADAKEIKDAISGIGTDEKCLIEILA

SRTNEQIHQLVAAYKDAYERDLEADITGDTSGHFRKMLVVLLQGTREEDDVVSEDLVQQD

VQDLYEAGELKWGTDEAQFIYILGNRSKQHLRLVFDEYLKTTGKPIEASIRGELSGDFEK

LMLAVVKCIRSTAEYFAERLFKAMKGLGTRDNTLIRIMVSRSELDMLDIREIFRTKYEKS

LYSMIKNDTSGEYKKTLLKLCGGDDDAAGQFFPEAAQVAYQMWELSAVARVELKGTVRPA

GDFNPDADAKALRKAMKGLGTDEDTIIDIIAHRSNAQRQQIRQTFKSHFGRDLMADLKSE

LSGDLARLILGLMMPPAHYDAKQLKKAMEGAGTDEKALIEILATRTNAEIQAINKAYKED

YHKSLEDALSSDTSGHFKRILISLATGNREEGGEDRERAREDAQVAAEILEIADTTSGDK

SSLETRFMMILCTRSYPHLRRVFQEFIKMTNYDVEHTIKKEMSGDVRDVFVAIVQSVKNK

PLFFADKLYKSMKGAGTDEKTLTRIMVSRSEIDLLNIRREFIEKYDKSLHQAIEGDTSGH

FLKALLAICGGED

>sp|P23196|APEX1_BOVIN DNA-(apurinic or apyrimidinic site) endonuclease OS=Bos taurus OX=9913 GN=APEX1 PE=1 SV=2

MPKRGKKGAVVEDAEEPKTEPEAKKSKAGAKKNEKEAVGEGAVLYEDPPDQKTSPSGKSA

TLKICSWNVDGLRAWIKKKGLDWVKEEAPDILCLQETKCSENKLPVELQELSGLSHQYWS

APSDKEGYSGVGLLSRQCPLKVSYGIGEEEHDQEGRVIVAEYDAFVLVTAYVPNAGRGLV

RLEYRQRWDEAFRKFLKGLASRKPLVLCGDLNVAHEEIDLRNPKGNKKNAGFTPQERQGF

GELLQAVPLTDSFRHLYPNTAYAYTFWTYMMNARSKNVGWRLDYFLLSQSVLPALCDSKI

RSKALGSDHCPITLYLAL

>sp|P19803|GDIR1_BOVIN Rho GDP-dissociation inhibitor 1 OS=Bos taurus OX=9913 GN=ARHGDIA PE=1 SV=3

MAEQEPTAEQLAQIAAENEEDEHSVNYKPPAQKSIQEIQELDKDDESLRKYKEALLGRVA

VSADPNVPNVVVTRLTLVCSTAPGPLELDLTGDLESFKKQSFVLKEGVEYRIKISFRVNR

EIVSGMKYIQHTYRKGVKIDKTDYMVGSYGPRAEEYEFLTPMEEAPKGMLARGSYNIKSR

FTDDDRTDHLSWEWNLTIKKEWKD

>sp|Q3SZJ0|ARLY_BOVIN Argininosuccinate lyase OS=Bos taurus OX=9913 GN=ASL PE=2 SV=1

MASESGKLWGGRFVGTVDPIMEKFNSSITYDRHLWEADVQGSKAYSRGLEKAGLLTKAEM

DQILHGLDKVAEEWAQGTFKLNPNDEDIHTANERRLKELIGETAGKLHTGRSRNDQVVTD

LRLWMRQNCSMLSALLCELIRTMVDRAEAERDVLFPGYTHLQRAQPIRWSHWILSHAVAL

TRDSERLLEVRKRINVLPLGSGAIAGNPLGVDRELLRAELDFGAITLNSMDATSERDFVA

EFLFWASLCMTHLSRMAEDLILYGTKEFSFVQLSDAYSTGSSLMPQKKNPDSLELIRSKA

GRVFGRCAGLLMTLKGLPSTYNKDLQEDKEAVFEVSDTMSAVLQVATGVISTLQIHRENM

GRALSPDMLATDLAYYLVRKGMPFRQAHEASGKAVFMAETKGVALNQLSLQELQTISPLF

SGDVSHVWDYGHSVEQYEALGGTARSSVDWQIGQLRALLRAQQTESPPHASPK

>sp|Q1LZA3|ASNS_BOVIN Asparagine synthetase [glutamine-hydrolyzing] OS=Bos taurus OX=9913 GN=ASNS PE=2 SV=3

MCGIWALFGSDDCLSVQCLSAMKIAHRGPDAFRFENVNGYTNCCFGFHRLAVVDQLFGMQ

PIRVKKYPYLWLCYNGEIYNHKKLQHHFEFEYQTKVDGEIILHLYDKGGIEQTVCMLDGV

FAFILLDTANKKVFLGRDTYGVRPLFKAMTEDGFLAVCSEAKGLVNLKHSMTPFLKVEPF

LPGHYEVLDLKPNGKVASVEMVKHHHCRDEPLHALYDGVEKLFPGFEIETVKSNLRILFD

NAVKKRLMTDRRIGCLLSGGLDSSLVAATLLKQLKEAQVQYPLQTFAIGMEDSPDLLAAR

KVANHIGSEHHEVLFNSEEGIQVLDEVIFSLETYDITTVRASVGMYLISKYIRKNTDSVV

IFSGEGSDELTQGYIYFHKAPSPEKAEEESERLLRELYLFDVLRADRTTAAHGLELRVPF

LDHRFSSYYLSLPPDMRVPKNGIEKHLLRETFEDSNLIPKEILWRPKEAFSDGITSVKNS

WFRILQDYIEHQVDDAAMASAAQKFPINTPKTKEGYYYRQIFENHYPGRADWLPHYWMPR

WTNATDPSARTLTHYKAAAKA

>sp|Q0VCL3|ATG3_BOVIN Ubiquitin-like-conjugating enzyme ATG3 OS=Bos taurus OX=9913 GN=ATG3 PE=2 SV=1

MQNVINTVKGKALEVAEYLTPVLKESKFKETGVITPEEFVAAGDHLVHHCPTWQWATGEE

LKVKAYLPSGKQFLVTKNVPCYKRCKQMEYSDELEAIIEEDDGDGGWVDTYHNTGIAGIT

EAVKEITLESKDSIKLQDCSALCEEEEEEDEGEAADMEEYEESGLLETDEATLDTRKIVE

ACKAKTDAGGEDAILQTRTYDLYITYDKYYQTPRLWLFGYDEQRQPLTVEHMYEDISQDH

VKKTVTIENHPHLPPPPMCSVHPCRHAEVMKKIIETVAEGGGELGVHMYLLIFLKFVQAV

IPTIEYDYTRHFTM

>sp|Q3T0C6|AT1B3_BOVIN Sodium/potassium-transporting ATPase subunit beta-3 OS=Bos taurus OX=9913 GN=ATP1B3 PE=2 SV=1

MTKTDKKSFNQSLAEWKLFIYNRTTGEFLGRTAKSWGLILLFYLVFYGFLAALFSFTMWA

MLQTLNDEVPKYRDQIPSPGLMVFPKPVSALDFSFSLSDAESYQGYIDDLKKFLKPYGLE

EQKNLTDCTNGTFFEQKGPEYTACQFPLALLEACSGVDDPTFGYQEGKPCILVKMNRIIG

LKPQGEPRIECTGKSESTAFLSTYPSNGTIDLKYFPYYGKKLHGNYLQPLVAVQVGFGSD

ADVREVMVECKIDGSPNLKTQNDRDKFLGRVAFKITMHA

>sp|P19483|ATPA_BOVIN ATP synthase subunit alpha, mitochondrial OS=Bos taurus OX=9913 GN=ATP5F1A PE=1 SV=1

MLSVRVAAAVARALPRRAGLVSKNALGSSFIAARNLHASNSRLQKTGTAEVSSILEERIL

GADTSVDLEETGRVLSIGDGIARVHGLRNVQAEEMVEFSSGLKGMSLNLEPDNVGVVVFG

NDKLIKEGDIVKRTGAIVDVPVGEELLGRVVDALGNAIDGKGPIGSKARRRVGLKAPGII

PRISVREPMQTGIKAVDSLVPIGRGQRELIIGDRQTGKTSIAIDTIINQKRFNDGTDEKK

KLYCIYVAIGQKRSTVAQLVKRLTDADAMKYTIVVSATASDAAPLQYLAPYSGCSMGEYF

RDNGKHALIIYDDLSKQAVAYRQMSLLLRRPPGREAYPGDVFYLHSRLLERAAKMNDAFG

GGSLTALPVIETQAGDVSAYIPTNVISITDGQIFLETELFYKGIRPAINVGLSVSRVGSA

AQTRAMKQVAGTMKLELAQYREVAAFAQFGSDLDAATQQLLSRGVRLTELLKQGQYSPMA

IEEQVAVIYAGVRGYLDKLEPSKITKFENAFLSHVISQHQALLSKIRTDGKISEESDAKL

KEIVTNFLAGFEA

>sp|P00829|ATPB_BOVIN ATP synthase subunit beta, mitochondrial OS=Bos taurus OX=9913 GN=ATP5F1B PE=1 SV=2

MLGLVGRVVAASASGALRGLSPSAPLPQAQLLLRAAPAALQPARDYAAQASPSPKAGATT

GRIVAVIGAVVDVQFDEGLPPILNALEVQGRETRLVLEVAQHLGESTVRTIAMDGTEGLV

RGQKVLDSGAPIRIPVGPETLGRIMNVIGEPIDERGPIKTKQFAAIHAEAPEFVEMSVEQ

EILVTGIKVVDLLAPYAKGGKIGLFGGAGVGKTVLIMELINNVAKAHGGYSVFAGVGERT

REGNDLYHEMIESGVINLKDATSKVALVYGQMNEPPGARARVALTGLTVAEYFRDQEGQD

VLLFIDNIFRFTQAGSEVSALLGRIPSAVGYQPTLATDMGTMQERITTTKKGSITSVQAI

YVPADDLTDPAPATTFAHLDATTVLSRAIAELGIYPAVDPLDSTSRIMDPNIVGSEHYDV

ARGVQKILQDYKSLQDIIAILGMDELSEEDKLTVSRARKIQRFLSQPFQVAEVFTGHLGK

LVPLKETIKGFQQILAGEYDHLPEQAFYMVGPIEEAVAKADKLAEEHS

>sp|P23956|VATL_BOVIN V-type proton ATPase 16 kDa proteolipid subunit c OS=Bos taurus OX=9913 GN=ATP6V0C PE=1 SV=1

MSEAKNGPEYASFFAVMGASAAMVFSALGAAYGTAKSGTGIAAMSVMRPEMIMKSIIPVV

MAGIIAIYGLVVAVLIANSLNDGISLYRSFLQLGAGLSVGLSGLAAGFAIGIVGDAGVRG

TAQQPRLFVGMILILIFAEVLGLYGLIVALILSTK

>sp|P31404|VATA_BOVIN V-type proton ATPase catalytic subunit A OS=Bos taurus OX=9913 GN=ATP6V1A PE=1 SV=2

MDFSKLPKIRDEDKESTFGYVHGVSGPVVTACDMAGAAMYELVRVGHSELVGEIIRLEGD

MATIQVYEETSGVSVGDPVLRTGKPLSVELGPGIMGAIFDGIQRPLSDISSQTQSIYIPR

GVNVSALSRDVKWDFTPCKNLRVGSHITGGDIYGIVNENSLIKHKIMLPPRNRGTVTYIA

PPGNYDTSDVVLELEFEGIKEKFSMVQVWPVRQVRPVTEKLPANHPLLTGQRVLDALFPC

VQGGTTAIPGAFGCGKTVISQSLSKYSNSDVIIYVGCGERGNEMSEVLRDFPELTMEVDG

KVESIMKRTALVANTSNMPVAAREASIYTGITLSEYFRDMGYHVSMMADSTSRWAEALRE

ISGRLAEMPADSGYPAYLGARLASFYERAGRVKCLGNPEREGSVSIVGAVSPPGGDFSDP

VTSATLGIVQVFWGLDKKLAQRKHFPSVNWLISYSKYMRALDEYYDKHFTEFVPLRTKAK

EILQEEEDLAEIVQLVGKASLAETDKITLEVAKLIKDDFLQQNGYTPYDRFCPFYKTVGM

LSNMIAFYDMARRAVETTAQSDNKITWSIIREHMGEILYKLSSMKFKDPVKDGEAKIKAD

YAQLLEDMQNAFRSLED

>sp|Q5I597|BHMT1_BOVIN Betaine--homocysteine S-methyltransferase 1 OS=Bos taurus OX=9913 GN=BHMT PE=2 SV=1

MAPAGGKNVKKGILERLNSGEVIIGDGGFVFALEKRGYVKAGPWTPEAAVEHPEAVRQLH

REFLRAGSNVMQTFTFYASEDKLENRGNYVAEKISGQKVNEAACDIARQVADEGDALVAG

GVSQTPSYLSCKSETEVKKVFQQQLEVFVKKNVDFLIAEYFEHVEEAVWAVEALKASGKP

VAATMCIGPEGDLHSVTPGECAVRLVKAGASIVGVNCHFDPTISLQTVKLMKEGLEAAGL

KAHLMSQPLAYHTPDCGKQGFIDLPEFPFGLEPRVATRWDIQKYAREAYNLGVRYIGGCC

GFEPYHIRAIAEELAPERGFLPLASEKHGSWGSGLDMHTKPWIRARARKEYWENLQIASG

RPYNPSMSKPDAWGVTKGTAELMQQKEATTEQQLRELFEKQKFKSAQ

>sp|P62157|CALM_BOVIN Calmodulin OS=Bos taurus OX=9913 GN=CALM PE=1 SV=2

MADQLTEEQIAEFKEAFSLFDKDGDGTITTKELGTVMRSLGQNPTEAELQDMINEVDADG

NGTIDFPEFLTMMARKMKDTDSEEEIREAFRVFDKDGNGYISAAELRHVMTNLGEKLTDE

EVDEMIREADIDGDGQVNYEEFVQMMTAK

>sp|Q5E997|CAZA2_BOVIN F-actin-capping protein subunit alpha-2 OS=Bos taurus OX=9913 GN=CAPZA2 PE=1 SV=3

MADLEEQLSDEEKVRIAAKFIIHAPPGEFNEVFNDVRLLLNNDNLLREGAAHAFAQYNLD

QFTPVKIEGYEDQVLITEHGDLGNGKFLDPKNRISFKFDHLRKEATDPRPYEAENAIESW

RTSVETALRAYVKEHYPNGVCTVYGKKIDGQQTIIACIESHQFQAKNFWNGRWRSEWKFT

ITPSTTQVVGILKIQVHYYEDGNVQLVSHKDIQDSLTVSNEVQTAKEFIKIVEAAENEYQ

TAISENYQTMSDTTFKALRRQLPVTRTKIDWNKILSYKIGKEMQNA

>sp|Q2T9X2|TCPD_BOVIN T-complex protein 1 subunit delta OS=Bos taurus OX=9913 GN=CCT4 PE=1 SV=3

MPENVAPRTGPPAGAAGAAGGRGKSAYQDRDKPAQIRFSNISAAKAVADAIRTSLGPKGM

DKMIQDGKGDVTITNDGATILKQMQVLHPAARMLVELSKAQDIEAGDGTTSVVIIAGSLL

DSCTKLLQKGIHPTIISESFQKALEKGIEILTDMSRPEELSDRETLLNSAATSLNSKVVS

QYSSLLSPMSVDAVMKVIDPATATSVDLRDIKIVKKLGGTIDDCELVEGLVLTQKVANSG

ITRVEKAKIGLIQFCLSAPKTDMDNQIVVSDYVQMDRVLREERAYILNLVKQIKKTGCNV

LLIQKSILRDALSDLALHFLNKMKIMVVKDIEREDIEFICKTIGTKPVAHVDQFTADMLG

SAELAEEVSLNGSGKLIKITGCASPGKTVTIVVRGSNKLVIEEAERSIHDALCVIRCLVK

KRALIAGGGAPEIELALRLTEYSRTLSGMESYCIRAFADAMEVIPSTLAENAGLNPISTV

TELRNRHAQGEKTTGINVRKGGISNILEEQVVQPLLVSVSALTLATETVRSILKIDDVVN

TR

>sp|Q3MHL7|TCPZ_BOVIN T-complex protein 1 subunit zeta OS=Bos taurus OX=9913 GN=CCT6A PE=1 SV=3

MAAVKTLNPKAEVARAQAALAVNISAARGLQDVLRTNLGPKGTMKMLVSGAGDIKLTKDG

NVLLHEMQIQHPTASLIAKVATAQDDITGDGTTSNVLIIGELLKQADLYISEGLHPRIIT

EGFEAAKEKALQFLEQVKVSKEMDRETLIDVARTSLRTKVHAELADVLTEAVVDSILAIK

KQDEPIDLFMVEIMEMKHKSETDTSLIRGLVLDHGARHPDMKKRVEDAYILTCNVSLEYE

KTEVNSGFFYKSAEEREKLVKAERKFIEDRVKKIIELKKKVCGDSDKGFVVINQKGIDPF

SLDALAKEGIIALRRAKRRNMERLTLACGGIALNSLDDLNPDCLGHAGLVYEYTLGEEKF

TFIEKCNNPRSVTLLIKGPNKHTLTQIKDAIRDGLRAVKNAIDDGCVVPGAGAVEVAMAE

ALVKYKPSVKGRAQLGVQAFADALLIIPKVLAQNSGFDLQETLVKVQAEHSESGQLVGVD

LNTGEPMVAAEAGIWDNYCVKKQLLHSCTVIATNILLVDEIMRAGMSSLKG

>sp|Q2NKZ1|TCPH_BOVIN T-complex protein 1 subunit eta OS=Bos taurus OX=9913 GN=CCT7 PE=1 SV=1

MMPTPVILLKEGTDSSQGIPQLVSNISACQVIAEAVRTTLGPRGMDKLIVDGRGKATISN

DGATILKLLDVVHPAAKTLVDIAKSQDAEVGDGTTSVTLLAAEFLKQVKPYVEEGLHPQI

IIRAFRTATQLAVNKIKEIAVTVKKEDKVEQRKLLEKCAMTALSSKLISQQKAFFAKMVV

DAVMMLDDLLQLKMIGIKKVQGGALEESQLVAGVAFKKTFSYAGFEMQPKKYHNPMIALL

NVELELKAEKDNAEIRVHTVEDYQAIVDAEWNILYDKLEKIHHSGAKVVLSKLPIGDVAT

QYFADRDMFCAGRVPEEDLKRTMMACGGSIQTSVNALSSDVLGRCQVFEETQIGGERYNF

FTGCPKAKTCTIILRGGAEQFMEETERSLHDAIMIVRRAIKNDSVVAGGGAIEMELSKYL

RDYSRTIPGKQQLLIGAYAKALEIIPRQLCDNAGFDATNILNKLRARHAQGGMWYGVDIN

TEDIADNFEAFVWEPAMVRINALTAASEAACLIVSVDETIKNPRSTVDASPAAGRGRGRG

RLH

>sp|Q3ZCI9|TCPQ_BOVIN T-complex protein 1 subunit theta OS=Bos taurus OX=9913 GN=CCT8 PE=1 SV=3

MALHVPKAPGFAQMLKEGAKHFSGLEEAVYRNIQACKELAQTTRTAYGPNGMNKMVINHL

EKLFVTNDAATILRELEVQHPAAKMIVMASHMQEQEVGDGTNFVLVFAGALLELAEELLR

LGLSVSEVIEGYEIACKKAHEILPDLVCCSAKNLRDVDEVSSLLHTSVMSKQYGNEVFLA

KLIAQACVSIFPDSGHFNVDNIRVCKILGSGVHSSSVLHGMVFKKETEGDVTSVKDAKIA

VYSCPFDGMITETKGTVLIKSAEELMNFSKGEENLMDAQVKAIADTGANVVVTGGRVADM

ALHYANKYNIMLVRLNSKWDLRRLCKTVGATALPRLNPPVLEEMGHCDSVYLSEVGDTQV

VVFKHEKEDGAISTIVLRGSTDNLMDDIERAVDDGVNTFKVLTRDKRLVPGGGATEIELA

KQITSYGETCPGLEQYAIKKFAEAFEAIPRALAENSGVKANEVISKLYAVHQEGNKNVGL

DIEAEVPAVKDMLEAGVLDTYLGKYWAIKLATNAAVTVLRVDQIIMAKPAGGPKPPSGKK

DWDEDQND

>sp|Q6URK6|CADH5_BOVIN Cadherin-5 OS=Bos taurus OX=9913 GN=CDH5 PE=2 SV=1

MQALVMLLATGATYYLGLLAAAAAAVNPGRPNTPGSLPAHRRQKRDWIWNQMHIDEERND

SLPHYVGKIKSSVDPKKTEYQLRGESAGKVFRVDKNTGDVYALERLDREKISEYHLTALV

VDKDSKKNLESPSSFTIKVHDVNDNWPVFTHRVFNASVPEMSGIGTSVIQVTAMDADDPT

VADHASVVYQLTKGKENFDIRGSGLIVTMNKHLDRETQDKYEVVVKAEDAQGRRGESGTA

TVFITLQDVNDNFPIFTQNRYTFSVPEDIRVGSPLGSLFVEDPDEPQNRKTKYSFVQGEY

RDTFTIDTDPSHNEGIIKPIKPLDYERIRQYSFTIEATDPTIDLRYLGSTSPKNIARVII

NVTDVDEPPIFQQPFYHFQLQENQKKPLIGSVLAKDPDAAQRDIRYSIRRTSDKGQFFGI

TKKGGIYNDKELDREVYPWYNLTVEAKEVDLSGTPTGKESIVQVHIEVMDENDNAPEFAK

PYEPKVCENAPQGKLVVQISATDKDITPRDVKFKFSLSTEDSNFTLIDNHDNTANIIVKY

GYFDRERAKVHHLPVLISDNGRPSLTGTSTLHVTVCKCNEHGEFTLCEEAAAQVGISIQA

LVAIFLCILTFTVITLLIILRRRLRKQARAHGKSVPEIHEQLVTYDEEGGGEMDTTSYDV

SVLNSARHGGAKPPRPALDARPSLYAQVQKPPRQAPGAHAPGEMAAMIGVKKDEADHDGG

GPPYDTLHIYGYEGAESIAESLSSLGTDSSDSDIDYDFLNDWGTRFKMLAELYGSDPQEE

LVY

>sp|E1BB52|CDK13_BOVIN Cyclin-dependent kinase 13 OS=Bos taurus OX=9913 GN=CDK13 PE=3 SV=1

MPSSSDTALGGGGGLSWAEKKLEERRKRRRFLSPQQPPLLLPLLQPQLLQPPPPPPPLLF

LAAPGTAAAAAAAAAASSSCFSPGPPLEVKRLARGKRRAGGRQKRRRGPRAGQEAEKRRV

FSLPQPQQDGGGGASSGGGVTPLVEYEDVSSQSEQGLLLGGASAATAATAAGGTGGSGGS

PASSSGTQRRGEGSERRPRRDRRSSSGRSKDRHREHRRRDGQRGGGEASKSRSRHGHGGE

ERAEAGKSGSSSSSGGRRKSASATSSSSSSRKDRDPKAHRSRTKSSKEPPSAYKEPPKAY

REDKTEPKAYRRRQRSLSPLGGRDDSPVSHRASQSLRNRKSPSPAGGGSSPYSRRLARSP

SPYSRRRSPSYSRHSSYERGGDVSPSPYSSSSWRRSRSPYSPVIRRSAKSRSRSPYSSRH

SRSRSRHRLSRSRSRHSSISPSTLTLKSSLAAELNKNKKARAAEAARAAEAAKAAEAAKA

AEAAAKAAKAASTSTPTKGNTETGASASQTNHVKDVKKLKTEHAPSPSSGGTLKNDKAKT

KPPLQVTKVDNNLIVDKATKKAVVVGKESKSAATKEEPVSLKEKTKPLTPSIGAKEKEQH

VALVTSTLPPLPLPPMLPEDKDADSLRGNISVKAVKKEVEKKLRCLLADLPLPPELPGGD

DLSKSPEEKKTATQLHNKRRPKICGPRFGEIKEKDIDWGKRCVDKFDIIGIIGEGTYGQV

YKARDKDTGEMVALKKVRLDNEKEGFPITAIREIKILRQLTHQSIINMKEIVTDKEDALD

FKKDKGAFYLVFEYMDHDLMGLLESGLVHFNENHIKSFMRQLMEGLDYCHKKNFLHRDIK

CSNILLNNRGQIKLADFGLARLYSSEESRPYTNKVITLWYRPPELLLGEERYTPAIDVWS

CGCILGELFTKKPIFQANQELAQLELISRICGSPCPAVWPDVIKLPYFNTMKPKKQYRRK

LREEFVFIPAAALDLFDYMLALDPSKRCTAEQALQCEFLRDVEPSKMPPPDLPLWQDCHE

LWSKKRRRQKQMGMTDDVSTVKAPRKDLSLGMDDSRTSTPQSVLPSSQLKPQGNSNAAPV

KTGPGQQLNHSELAILLNLLQSKTSVNMADFVQVLNIKVNSETQQQLNKINLPAGILATG

EKQTDPSTPQQESSKPLGGIQPSQNMQPKVEPDAAQAAVQSAFAVLLTQLIKAQQSKQKD

VLLEERENGSGHEAPLQLRPPPEPATPASGQDDLIQHQDMRLLELTPEPDRPRILPPDQR

PPEPPEPPPVTEEDLDYRTENQHVPTTSSSLTDPHAGVKAALLQLLAQHQPQDDPKRESG

IDYQAGDTYVPSSDYKDNFGSSSFSSAPYVSNDGLGSASAPPLERRSFMGNSDIQSLDNY

STTSSHSGGPPQPSAFSESFPSSVAGYGDIYLNTGPMLFSGDKDHRFEYSHGPIAVLANS

SDPSTGPESTHPLPAKMHNYNYGGSLQETPGGHGLMHGQTWTSPAQGPGYSQGYRGHIST

SAGRGRGRGLPY

>sp|Q3T0A3|CFAD_BOVIN Complement factor D OS=Bos taurus OX=9913 GN=CFD PE=2 SV=1

MADRSLHLVVLILLGTALCAAQPRGRILRGQEAPSHSRPYMASVQVNGKHVCGGFLIAEQ

WVMSAAHCLEDVADGKVQVLLGAHSLSQPEPSKRLYDVLRVVPHPGSRTETIDHDLLLLQ

LSEKAVLGPAVQLLPWQREDRDVAAGTLCDVAGWGVVSHTGRKPDRLQHLLLPVLDRATC

NLRTYHDGTITERMMCAESNRRDTCKGDSGGPLVCGSVAEGVVTSGSRICGNHKKPGIYT

RLASYVAWIDGVMAEGAAA

>sp|Q148F1|COF2_BOVIN Cofilin-2 OS=Bos taurus OX=9913 GN=CFL2 PE=2 SV=1

MASGVTVNDEVIKVFNDMKVRKSSTQEEIKKRKKAVLFCLSDDKRQIIVEEAKQILVGDI

GDTVEDPYTSFVKLLPLNDCRYALYDATYETKESKKEDLVFIFWAPESAPLKSKMIYASS

KDAIKKKFTGIKHEWQVNGLDDIKDRSTLGEKLGGNVVVSLEGKPL

>sp|P23206|COAA1_BOVIN Collagen alpha-1(X) chain OS=Bos taurus OX=9913 GN=COL10A1 PE=2 SV=1

MLPQTALLLLMSLNLVHGVFYTERYQTPTGIKGPPSNTKTQFFIPYAIKGKGVSLRGEQG

IPGPPGPAGPRGHPGPSGPPGKPGTGSPGPQGQPGLPGPPGPSATGKPGLPGLPGKQGER

GLNGPKGDIGPAGLPGPRGPPGPPGIPGPAGISVPGKPGPQGPTGEPGPRGFPGEKGTSG

VPGLNGQKGEMGHCTPCRPGERGLPGPQGPTGPPGPPGVGKRGENGLPGQPGLKGDQGVP

GERGAAGPSGPQGPPGEQGPEGIGKPGAPGIPGQPGIPGMKGQPGAPGTAGLPGAPGFGK

PGLPGLKGQRGPVGLPGSPGAKGEQGPAGHPGEAGLPGPSGNMGPQGPKGIPGNPGLPGP

KGEMGPVGPAGNPGAKGERGSSGLDGKPGYPGEPGLNGPKGNPGLPGPKGDPGIAGSPGL

PGPVGPAGAKGVPGHNGEAGPRGVPGIPGTRGPIGPPGIPGFPGSKGDVGTPGPPGPAGI

AVKGLNGPTGPPGPPGPRGNAGEPGLPGPPGPPGPPGQVALPEDFVKAGQRPFVSANQGV

TGMPVSAFTVILSKAYPAIGTPIPFDKILYNKQQHYDPRTGIFTCKIPGIYYFSYHIHVK

GTHAWVGLYKNGTPVMYTYDEYIKGYLDQASGSAVIDLTENDQVWLQLPNAGSNGLYSPE

YVHSSFSGFLVAPM

>sp|P02459|CO2A1_BOVIN Collagen alpha-1(II) chain OS=Bos taurus OX=9913 GN=COL2A1 PE=1 SV=4

MIRLGAPQTLVLLTLLVAAVLRCHGQDVQKAGSCVQDGQRYNDKDVWKPEPCRICVCDTG

TVLCDDIICEDMKDCLSPETPFGECCPICSADLPTASGQPGPKGQKGEPGDIKDIVGPKG

PPGPQGPAGEQGPRGDRGDKGEKGAPGPRGRDGEPGTPGNPGPPGPPGPPGPPGLGGNFA

AQMAGGFDEKAGGAQMGVMQGPMGPMGPRGPPGPAGAPGPQGFQGNPGEPGEPGVSGPMG

PRGPPGPPGKPGDDGEAGKPGKSGERGPPGPQGARGFPGTPGLPGVKGHRGYPGLDGAKG

EAGAPGVKGESGSPGENGSPGPMGPRGLPGERGRTGPAGAAGARGNDGQPGPAGPPGPVG

PAGGPGFPGAPGAKGEAGPTGARGPEGAQGPRGEPGTPGSPGPAGAAGNPGTDGIPGAKG

SAGAPGIAGAPGFPGPRGPPGPQGATGPLGPKGQTGEPGIAGFKGEQGPKGEPGPAGPQG

APGPAGEEGKRGARGEPGGAGPAGPPGERGAPGNRGFPGQDGLAGPKGAPGERGPSGLAG

PKGANGDPGRPGEPGLPGARGLTGRPGDAGPQGKVGPSGAPGEDGRPGPPGPQGARGQPG

VMGFPGPKGANGEPGKAGEKGLPGAPGLRGLPGKDGETGAAGPPGPAGPAGERGEQGAPG

PSGFQGLPGPPGPPGEGGKPGDQGVPGEAGAPGLVGPRGERGFPGERGSPGSQGLQGARG

LPGTPGTDGPKGAAGPAGPPGAQGPPGLQGMPGERGAAGIAGPKGDRGDVGEKGPEGAPG

KDGGRGLTGPIGPPGPAGANGEKGEVGPPGPAGTAGARGAPGERGETGPPGPAGFAGPPG

ADGQPGAKGEQGEAGQKGDAGAPGPQGPSGAPGPQGPTGVTGPKGARGAQGPPGATGFPG

AAGRVGPPGSNGNPGPPGPPGPSGKDGPKGARGDSGPPGRAGDPGLQGPAGPPGEKGEPG

DDGPSGPDGPPGPQGLAGQRGIVGLPGQRGERGFPGLPGPSGEPGKQGAPGASGDRGPPG

PVGPPGLTGPAGEPGREGSPGADGPPGRDGAAGVKGDRGETGAVGAPGAPGPPGSPGPAG

PIGKQGDRGEAGAQGPMGPAGPAGARGMPGPQGPRGDKGETGEAGERGLKGHRGFTGLQG

LPGPPGPSGDQGASGPAGPSGPRGPPGPVGPSGKDGANGIPGPIGPPGPRGRSGETGPAG

PPGNPGPPGPPGPPGPGIDMSAFAGLGQREKGPDPLQYMRADEAAGNLRQHDAEVDATLK

SLNNQIESLRSPEGSRKNPARTCRDLKLCHPEWKSGDYWIDPNQGCTLDAMKVFCNMETG

ETCVYPNPASVPKKNWWSSKSKDKKHIWFGETINGGFHFSYGDDNLAPNTANVQMTFLRL

LSTEGSQNITYHCKNSIAYLDEAAGNLKKALLIQGSNDVEIRAEGNSRFTYTVLKDGCTK

HTGKWGKTMIEYRSQKTSRLPIIDIAPMDIGGPEQEFGVDIGPVCFL

>sp|Q7SIB2|CO4A1_BOVIN Collagen alpha-1(IV) chain OS=Bos taurus OX=9913 GN=COL4A1 PE=1 SV=2

MGPRLGVWLLLLLAALLLHEESSRAAAKGGCAGSGCGKCDCHGVKGQKGERGLPGLQGVI

GFPGMQGPEGPQGPPGQKGDTGEPGLPGTKGTRGPSGVPGYPGNPGLPGIPGQDGPPGPP

GIPGCNGTKGERGPVGPPGLPGFAGNPGPPGLPGMKGDPGEILGHIPGTLLKGERGYPGQ

PGAPGSPGLPGLQGPVGPPGFTGPPGPPGPPGPPGEKGQMGLSFQGPKGEKGDQGVSGPP

GLPGQAQVITKGDTAMRGEKGQKGEPGFPGLPGFGEKGEPGKPGPRGKPGKDGEKGEKGS

PGFPGDSGYPGQPGQDGLKGEKGEAGPPGLPGTVIGTGPLGEKGEPGYPGGPGAKGETGP

KGFPGIPGQPGPPGFPTPGLIGAPGFPGDRGEKGEPGLPGVSLPGPSGRDGLPGPPGPPG

PPGQPGHTNGIVECQPGPPGDQGPPGIPGQPGLTGEVGEKGQKGDSCLVCDTAELRGPPG

PQGPPGEIGFPGQPGAKGDRGLPGRDGLEGLPGPQGAPGLMGQPGAKGEPGEIYFDIRLK

GDKGDPGFPGQPGMPGRAGSPGRDGQPGLPGPRGSPGSVGLKGERGPPGGVGFPGSRGDI

GPPGPPGFGPIGPIGDKGQIGFPGTPGAPGQPGPKGEAGKVVPLPGPPGAEGLPGSPGFQ

GPQGDRGFPGSPGRPGLPGEKGAIGQPGIGFPGPPGPKGVDGLPGDAGPPGNPGRQGFNG

LPGNPGPPGQKGEPGVGLPGLKGLPGIPGIPGTPGEKGNVGGPGIPGEHGAIGPPGLQGL

RGDPGPPGFQGPKGAPGVPGIGPPGAMGPPGGQGPPGSSGPPGVKGEKGFPGFPGLDMPG

PKGDKGSQGLPGLTGQSGLPGLPGQQGTPGQPGIPGPKGEMGVMGTPGQPGSPGPAGVPG

LPGAKGDHGFPGSSGPRGDPGFKGDKGDVGLPGKPGSMDKVDMGSMKGEKGDQGEKGQTG

PTGDKGSRGDPGTPGVPGKDGQAGHPGQPGPKGDPGVSGIPGAPGLPGPKGSAGGMGLPG

MPGPKGVAGIPGPQGIPGLPGDKGAKGEKGQAGLPGIGIPGRPGDKGDQGLAGFPGSPGE

KGEKGSTGIPGMPGSPGPKGSPGSVGYPGSPGLPGEKGDKGLPGLDGIPGIKGEAGLPGK

PGPTGPAGQKGEPGSDGIPGSVGEKGESGLPGRGFPGFPGSKGDKGSKGDVGFPGLSGSP

GIPGSKGEQGFMGPPGPQGQPGLPGTPGHAVEGPKGDRGPQGQPGLPGRPGPMGPPGLPG

LEGLKGERGNPGWPGTPGAPGPKGDPGFQGMPGIGGSPGITGAKGDVGPPGVPGFHGQKG

APGLQGVKGDQGDQGFPGTKGLPGPPGPPGPFSIIKGEPGLPGPEGPAGLKGLQGPPGPK

GQQGVTGSVGLPGPPGEPGFDGAPGQKGETGPFGPPGPRGFPGPPGPDGLPGSMGPPGTP

SVDHGFLVTRHSQTTDDPQCPPGTKILYHGYSLLYVQGNERAHGQDLGTAGSCLRKFSTM

PFLFCNINNVCNFASRNDYSYWLSTPEPMPMSMAPITGENIRPFISRCAVCEAPAMVMAV

HSQTIQIPQCPTGWSSLWIGYSFVMHTSAGAEGSGQALASPGSCLEEFRSAPFIECHGRG

TCNYYANAYSFWLATIERSEMFKKPTPSTLKAGELRTHVSRCQVCMRRT

>sp|Q7SIB3|CO4A2_BOVIN Collagen alpha-2(IV) chain (Fragment) OS=Bos taurus OX=9913 GN=COL4A2 PE=1 SV=1

ISIGYLLVKHSQTDQEPMCPVGMNKLWSGYSLLYFEGQEKAHNQDLGLAGSCLARFSTMP

FLYCNPGDVCYYASRNDKSYWLSTTAPLPMMPVAEEDIRPYISRCSVCEAPAVAIAVHSQ

DVSIPHCPAGWRSLWIGYSFLMHTAAGDEGGGQSLVSPGSCLEDFRATPFIECNGARGTC

HYYANKYSFWLTTIPEQSFQGTPSADTLKAGLIRTHISRCQVCMKNL

>sp|O97764|QOR_BOVIN Zeta-crystallin OS=Bos taurus OX=9913 GN=CRYZ PE=2 SV=2

MATGQKLMRAIRVFEFGGPEVLKLQSDVAVPIPKDHQVLIKVQACGVNPVDTYIRSGTHN

IKPLLPYTPGFDVAGIIEAVGESVSAFKKGDRVFTTRTISGGYAEYALAADHTVYTLPEK

LDFKQGAAIGIPYFTAYRALLHSACVKPGESVLVHGASGGVGIAACQIARAYGLKVLGTA

STEEGQKIVLENGAHKVFNHKEADYIDKIKKSVGEKGVDVIIEMLANVNLSNDLNLLSHG

GRVIVVGSRGTIEINPRDTMTKESSIKGVTLFSSTKEEFQQFAAALQAGMEIGWLRPVIG

PQYLLEKATQAHENIIHSSGATGKMILLLN

>sp|Q0VCX4|CTNB1_BOVIN Catenin beta-1 OS=Bos taurus OX=9913 GN=CTNNB1 PE=1 SV=1

MATQADLMELDMAMEPDRKAAVSHWQQQSYLDSGIHSGATTTAPSLSGKGNPEEEDVDTT

QVLYEWEQGFSQSFTQEQVADIDGQYAMTRAQRVRAAMFPETLDEGMQIPSTQFDAAHPT

NVQRLAEPSQMLKHAVVNLINYQDDAELATRAIPELTKLLNDEDQVVVNKAAVMVHQLSK

KEASRHAIMRSPQMVSAIVRTMQNTNDVETARCTAGTLHNLSHHREGLLAIFKSGGIPAL

VKMLGSPVDSVLFYAITTLHNLLLHQEGAKMAVRLAGGLQKMVALLNKTNVKFLAITTDC

LQILAYGNQESKLIILASGGPQALVNIMRTYTYEKLLWTTSRVLKVLSVCSSNKPAIVEA

GGMQALGLHLTDPSQRLVQNCLWTLRNLSDAATKQEGMEGLLGTLVQLLGSDDINVVTCA

AGILSNLTCNNYKNKMMVCQVGGIEALVRTVLRAGDREDITEPAICALRHLTSRHQEAEM

AQNAVRLHYGLPVVVKLLHPPSHWPLIKATVGLIRNLALCPANHAPLREQGAIPRLVQLL

VRAHQDTQRRTSMGGTQQQFVEGVRMEEIVEGCTGALHILARDVHNRIVIRGLNTIPLFV

QLLYSPIENIQRVAAGVLCELAQDKEAAEAIEAEGATAPLTELLHSRNEGVATYAAAVLF

RMSEDKPQDYKKRLSVELTSSLFRTEPMAWNETADLGLDIGAQGEPLGYRQDDPSYRSFH

SGGYGQDALGMDPMMEHEMGGHHPGADYPVDGLPDLGHAQDLMDGLPPGDSNQLAWFDTD

L

>sp|P07688|CATB_BOVIN Cathepsin B OS=Bos taurus OX=9913 GN=CTSB PE=1 SV=5

MWRLLATLSCLLVLTSARSSLYFPPLSDELVNFVNKQNTTWKAGHNFYNVDLSYVKKLCG

AILGGPKLPQRDAFAADVVLPESFDAREQWPNCPTIKEIRDQGSCGSCWAFGAVEAISDR

ICIHSNGRVNVEVSAEDMLTCCGGECGDGCNGGFPSGAWNFWTKKGLVSGGLYNSHVGCR

PYSIPPCEHHVNGSRPPCTGEGDTPKCSKTCEPGYSPSYKEDKHFGCSSYSVANNEKEIM

AEIYKNGPVEGAFSVYSDFLLYKSGVYQHVSGEIMGGHAIRILGWGVENGTPYWLVGNSW

NTDWGDNGFFKILRGQDHCGIESEIVAGMPCTHQY

>sp|Q3ZCJ8|CATC_BOVIN Dipeptidyl peptidase 1 OS=Bos taurus OX=9913 GN=CTSC PE=2 SV=1

MGPWSGSRLVALLLLVYGAGSVRGDTPANCTYPDLLGTWVFQVGSSGSQRDVNCSVMGPP

EKKVVVHLKKLDTAYDDFGNSGHFTIIYNQGFEIVLNDYKWFAFFKYKEEGGKVTSYCHE

TMTGWVHDVLGRNRACFTGRKTGNTSENVNVNTARLAGLEETYSNRLYRYNHDFVKAINA

IQKSWTAAPYMEYETLTLKEMIRRGGGHSRRIPRPKPAPITAEIQKKILHLPTSWDWRNV

HGINFVTPVRNQGSCGSCYSFASMGMMEARIRILTNNTQTPILSPQEVVSCSQYAQGCEG

GFPYLIAGKYAQDFGLVEEDCFPYTGTDSPCRLKEGCFRYYSSEYHYVGGFYGGCNEALM

KLELVHQGPMAVAFEVYDDFLHYRKGVYHHTGLRDPFNPFELTNHAVLLVGYGTDAASGL

DYWIVKNSWGTSWGENGYFRIRRGTDECAIESIALAATPIPKL

>sp|P07514|NB5R3_BOVIN NADH-cytochrome b5 reductase 3 OS=Bos taurus OX=9913 GN=CYB5R3 PE=1 SV=3

MGAQLSTLGHVVLSPVWFLYSLIMKLFQRSTPAITLENPDIKYPLRLIDKEVISHDTRRF

RFALPSPEHILGLPVGQHIYLSARIDGNLVIRPYTPVSSDDDKGFVDLVIKVYFKDTHPK

FPAGGKMSQYLESMKIGDTIEFRGPNGLLVYQGKGKFAIRPDKKSDPVIKTVKSVGMIAG

GTGITPMLQVIRAIMKDPDDHTVCHLLFANQTEKDILLRPELEELRNEHSARFKLWYTVD

KAPEAWDYSQGFVNEEMIRDHLPPPEEEPLVLMCGPPPMIQYACLPNLDRVGHPKERCFA

F

>sp|Q3SYZ4|SYDC_BOVIN Aspartate--tRNA ligase, cytoplasmic OS=Bos taurus OX=9913 GN=DARS1 PE=2 SV=1

MPSANASRRSQEKPREIMDAAEDYAKERYGVSSMIQSQEKPDRVLVRISDLTVQKAGEVV

WVRARVHTSRAKGKQCFLVLRQQQFNVQALVAVGDHASKQMVKFAANINKESIVDVEGVV

RKVNQKIGSCTQQDVELHVQKIYVISSAEPRLPLQLDDAVRPEVEGEEEGRATVNQDTRL

DNRVIDLRTSTSQAIFRLQSGICHPFRETLTNKGFVEIQTPKIISAASEGGANVFTVSYF

KNNAYLAQSPQLYKQMCICADFEKVFCIGPVFRAEDSNTHRHLTEFVGLDIEMAFNYHYH

EVVEEIADTLVQIFKGLQKRFQTEIQTVNKQFPCEPFKFLEPTLRLEYCEALAMLREAGI

EMGDEEDLSTPNEKLLGRLVKEKYDTDFYILDKYPLAVRPFYTMPDPRNPKQSNSYDMFM

RGEEILSGAQRIHDPQLVTERALHHGIDLEKIKAYIDSFRFGAPPHAGGGIGLERVTMLF

LGLHNVRQTSMFPRDPKRLTP

>sp|Q3T0V9|DEOC_BOVIN Deoxyribose-phosphate aldolase OS=Bos taurus OX=9913 GN=DERA PE=2 SV=1

MSAHQQGTELDLSWISKIQVNKPAVLRRAEQIQARRPVKKEWQAAWLLKAVTCIDLTTLS

GDDTASNIQRLCYKAKYPIREDLLKALNMHDKGITTAAVCVYPARVCDAVRALKAAGCDI

PVASVATGFPAAQTHLKTRLEEIRLAVEDGATEIDVVINRTLVLTGQWKALYDEIRQFRK

ACGEAHLKTILATGELGSLTNVYKASMIAMMAGSDFIKTSTGKETVNATFPVAIVMLRAI

RDFFWKTGNKVGFKPAGGIRSAKDSLVWLSLIKEELGDEWMKPELFRIGASTLLADIERQ

IYHHVTGRYAAYHDLPMS

>sp|Q03763|DSG1_BOVIN Desmoglein-1 OS=Bos taurus OX=9913 GN=DSG1 PE=1 SV=1

MNWPFFRAAVVLFIFLVVLEVNSDFRIQVRDYNTKNGTIKWHSLRRQKREWIKFAAACRE

GEDNSKRNPIAKIHSDCAANQQVTYRISGVGIDQPPYGIFVINQKTGEINITSIVDREVT

PFFIIYCRALNSLGQDLEKPLELRVRVLDINDNPPVFSMSTFVGEIEENSNANTLVMVLN

ATDADEPNNLNSKIAFKIIRQEPSDSPMFIINRYTGEIRTMNNFLDREQYGQYSLAVRGS

DRDGGADGMSAECECNIKILDVNDNIPYMELPTQSISIEENSLNSNLLQIRVIDLDEEFS

ANWMAVIFFISGNEGNWFEIEMNERTNVGTLKVVKPLDFEAMNNLQLSLGVRNKAEFHQS

IMSQYKLTATAISVTVLNVVEGSVFRPGSKTFVVNSNMGQNYKIGEYVAWDLDANRPSTT

VRYVMGRNPTDLLAIDSKTAIITLRNKVTMEQYKILGGKYQGTILSIDDALQRTCTGTIV

INLENGGWKTERPNVNGSTTSAYGLTSGGVTTNGYTTGGGVGTVTFAVGTNGYGVGTGVY

QPLRDNVHFGPAGIGLLIMGFLVLGLVPFLLMCCDCGGAPGGGAAFEPVPECSDGAIHSW

AVEGAQADPGVLANSAVPCIPVTNANVIEYVDNSGVYTNEYGAREMQDLGGGERTTGFEL

TDGVKMSGGPEICQEYPGTLRRNSMRECREGGLNMNFMESYFCQKAYAYADEDEGRPSND

CLLIYDIEGAGSPAGSVGCCSFIGEDLDDSFLDTLGPKFKKLADISLGKDVEPFPDSDPS

WPPKSTEPVCPPQGTEPTGGGHPPISPRFGTTTVISENTYPSGPGVQHPTPIPDPLGYGN

VTVTESYTSSGTLKPSVHIHDNRHASNVVVTERVVGPISGADLQGMLEMPDLRDGSNVIV

TERVIAPSSSLPTTLTIPDPRQSSNVVVTERVIQPTSGIVGNLSMHPELSNTHNVIVTER

VVSGSGITGSSSLLGSAGGGSGGGIGLGSLGGGGGLSSSLGGAATIGHLRGSAEHHFSNT

LGSASPTTTRSRITKYSTVQYTK

>sp|Q5E9D5|DEST_BOVIN Destrin OS=Bos taurus OX=9913 GN=DSTN PE=2 SV=3

MASGVQVADEVCRIFYDMKVRKCSTPEEIKKRKKAVIFCLSADKKCIIVEEGKEILVGDV

GVTITDPFKHFVGMLPEKDCRYALYDASFETKESRKEELMFFLWAPELAPLKSKMIYASS

KDAIKKKFQGIKHECQANGPEDLNRACIAEKLGGSLIVAFEGCPV

>sp|Q5E983|EF1B_BOVIN Elongation factor 1-beta OS=Bos taurus OX=9913 GN=EEF1B PE=2 SV=3

MGFGDLKSPAGLQVLNDYLADKSYIEGYVPSQADVAVFEAVSGPPPADLCHALRWYNHIK

SYEKEKASLPGVKKALGKYGPANVEDTTESGATDSKDDDDIDLFGSDDEEESEEAKRLRE

ERLAQYESKKAKKPALVAKSSILLDVKPWDDETDMAKLEECVRSIQADGLVWGSSKLVPV

GYGIKKLQIQCVVEDDKVGTDMLEEQITAFDEYVQSMDVAAFNKI

>sp|Q5E9R3|EHD1_BOVIN EH domain-containing protein 1 OS=Bos taurus OX=9913 GN=EHD1 PE=1 SV=1

MFSWVSKDARRKKEPELFQTVSEGLRQLYAQKLLPLEEHYRFHEFHSPALEDADFDNKPM

VLLVGQYSTGKTTFIRHLIEQDFPGMRIGPEPTTDSFIAVMHGPTEGVVPGNALVVDPRR

PFRKLNAFGNAFLNRFMCAQLPNPVLDSISIIDTPGILSGEKQRISRGYDFAAVLEWFAE

RVDRIILLFDAHKLDISDEFSEVIKALKNHEDKIRVVLNKADQIETQQLMRVYGALMWSL

GKIINTPEVVRVYIGSFWSHPLLIPDNRKLFEAEEQDLFKDIQSLPRNAALRKLNDLIKR

ARLAKVHAYIISSLKKEMPNVFGKESKKKELVNNLGEIYQKIEREHQISPGDFPNLRKMQ

ELLQTQDFSKFQALKPKLLDTVDDMLANDIARLMVMVRQEESLMPAQVVKGGAFDGTMNG

PFGHGYGEGAGEGIDDVEWVVGKDKPTYDEIFYTLSPVNGKITGANAKKEMVKSKLPNTV

LGKIWKLADVDRDGLLDDEEFALANHLIKVKLEGHELPADLPPHLVPPSKRRHE

>sp|Q9N179|EPB41_BOVIN Protein 4.1 OS=Bos taurus OX=9913 GN=EPB41 PE=2 SV=1

MHCKVSLLDDTVYECVVEKHAKGQDLLKRVCEHLNLLEEDYFGLAIWDNATSKTWLDSAK

EIKKQVRGVPWNFTFNVKFYPPDPAQLTEDITRYYLCLQLRQDIVSGRLPCSFATLALLG

SYTIQSELGDYDPELHGADYVSDFKLAPNQTKELEEKVMELHKSYRSMTPAQADLEFLEN

AKKLSMYGVDLHKAKDLEGVDIILGVCSSGLLVYKEKLRINRFPWPKVLKISYKRSSFFI

KIRPGEQEQYESTIGFKLPSYRAAKKLWKVCVEHHTFFRLTSTDTIPKSKFLALGSKFRY

SGRTQAQTRQASALIDRPAPHFERTASKRASRSLDGAAAVEPADRTPRPTSAPAIAPSPA

AEGGVPGAPVKKAQKETVQVEVKQEEAPPEDAEPEPSEAWKKKRERLDGENIYIRHSNLM

LEDLDKSQEEIKKHHASISELKKNFMESVPEPRPSEWDKRLSTHSPFRTLNINGQIPTGE

GPPLVKTQTVTISDTANAVKSEIPTKDVPIVHTETKTITYEAAQTDDSNGDLDPGVLLTA

QTITSETTSSTTTTQITKTVKGGISETRIEKRIVITGDADIDHDQVLVQAIKEAKEQHPD

MSVTKVVVHQETEISEE

>sp|Q3T0L2|ERP44_BOVIN Endoplasmic reticulum resident protein 44 OS=Bos taurus OX=9913 GN=ERP44 PE=2 SV=1

MIPGIFLSLPDLRCSLLLLVTWVFTPVTAEIISLDTENIDDILNNADVALVNFYADWCRF

SQMLHPIFEEASNVIKEEYPNANQVVFARVDCDQHSDIAQRYRISKYPTLKLFRNGMMMK

REYRGQRSVKALADYIRQQKSDPIQELHDLAEITTPDRSKRNIIGYFEQKDSENYRVFER

VANILHDDCAFLAAFGVVSKPERYSGDNIVYKPPGHSAPDMVYLGSMTNFDGTYNWIQDK

CVPLVREITFENGEELTEEGLPFLILFHMKEDTESLEIFQNEVARQLISEKGTINFLHAD

CDKFRHPLLHIQKTPADCPVIAIDSFRHMYVFGDFRDVLIPGKLKQFVFDLHSGKLHREF

HHGPDPTDTAPGEEVQDVASSPPESSFQKLAPSEYRYTLLRDRDEL

>sp|Q2HJH3|CK054_BOVIN Ester hydrolase C11orf54 homolog OS=Bos taurus OX=9913 PE=2 SV=1

MACAEYSFHVPSLEELVGVLQKGLTDNFAEVQVSVVDCPDLTKEPFTFPIKGICGKTRIA

EVGGVPYLLPLVNEKKVYDLNKIAKDIQLPGAFVLGAGAGPFQTLGFNSEFMLLVQTESE

HRPPVNGSYFARVNPADGGCLLEKYSEKYHDFGCALLANLFASEGQPGKVIEVKVKRRTG

KLNFVTCMRQTLEKHYGDKPVGMGGAFIIQKGKVKTHIMPAEFSSCPLNSDEDVNKWLHF

YEMKAPLVCLPVFVSRDPGFDLRLEHTHCFSHHGEGGHYHYDTTPDIVEYLGYFLPAEFL

YRIDQPKETHSFGRD

>sp|P98140|FA12_BOVIN Coagulation factor XII OS=Bos taurus OX=9913 GN=F12 PE=1 SV=2

MRALLLLGALLVSLESTVSTPPWKGPKKHKLTDSEHTVVLTVTGEPCHFPFQYHRQLHHK

CIHRGRPGPRPWCATTPNFEKDQRWAYCLEPKKVKDHCSKHNPCQKGGTCVNMPDGPRCI

CADHFTGKHCQKEKCFEPQFFRFFHENEIWHRLEPAGVVKCQCKGPNAQCKPLASQVCRT

NPCLNGGSCLQAEGHRLCRCAPSFAGRLCDVDLKASCYDDRDRGLSYRGMAGTTLSGAPC

QSWASEATYWNVTAEQVLNWGLGDHAFCRASTPPRGYRNPDNDTRPLCFIWKGDRLSWNY

CRLAPCQAAAGHEHFPLPSPSALQKPESTTQTPLPSLTSGWCSPTPLASGGPGGCGQRLR

KWLSSLNRVVGGLVALPGAHPYIAALYWDQHFCAGSLIAPCWVLTAAHCLQNRPAPKELT

VVLGQDRHNQSCEQCQTLAVRDYRLHEAFSPITYQHDLALVRLQESADGCCAHPSPFVQP

VCLPSTAARPAESEAAVCEVAGWGHQFEGGEYSSFLQEAQVPLIDPQRCSAPDVHGAAFT

QGMLCAGFLEGGTDACQGDSGGPLVCEDETPERQLILRGIVSWGSGCGNRLKPGVYTDVA

NYLAWIREHTAS

>sp|Q9TRY0|FKBP4_BOVIN Peptidyl-prolyl cis-trans isomerase FKBP4 OS=Bos taurus OX=9913 GN=FKBP4 PE=1 SV=4

MTAEETKAAESGAQSAPLRLEGVDISPKQDEGVLKVIKREGTGTETPMIGDRVFVHYTGW

LLDGTKFDSSLDRKDRFSFDLGKGEVIKAWDIAVATMKVGEVCHITCKPEYAYGLAGSPP

KIPPNATLVFEVELFEFKGEDLTEEEDGGIIRRIRTRGEGYAKPNEGALVEVALEGYFKD

QVFDRRELRFEVGEGESMDLPCGLEKAIQRMEKGEHSIVYLKPRYAFGSAGKEKFQIPPN

AELKYEIHLKSFEKAKESWEMSSEEKLEQSTIVKERGTVYFKEGKYKQAVLQYKKIVSWL

EYESSFSDEDAEKAQALRLASHLNLAMCHLKLQAFSAAIENCNKALELDSNNEKGLFRRG

EAHLAVNDFDLARADFQKVLQLYPSNKAAKAQLVVCQQRIRKQLEKEKKLYANMFERLAE

EETKAKATVAAGDQPADAEMRDEPKNDVAGGQPQVEAEA

>sp|Q08DN8|FLOT1_BOVIN Flotillin-1 OS=Bos taurus OX=9913 GN=FLOT1 PE=2 SV=1

MFFTCGPNEAMVVSGFCRSPPVMVAGGRVFVLPCIQQIQRISLNTLTLNVKSEKVYTRHG

VPISVTGIAQVKIQGQNKEMLAAACQMFLGKTEAEIAHIALETLEGHQRAIMAHMTVEEI

YKDRQKFSEQVFKVASSDLVNMGISVVSYTLKDIHDDQDYLHSLGKARTAQVQKDARIGE

AEAKRDAGIREAKAKQEKVSAQYLSEIEMAKAQRDYELKKAAYDIEVNTRRAQADLAYQL

QVAKTKQQIEEQRVQVQVVERAQQVAVQEQEIARREKELEARVRKPAEAERYKLERLAEA

EKSQLIMQAEAEAEAVRMRGEAEAFAIGARARAEAEQMAKKAEAFQLYQEAAQLDMLLEK

LPQVAEEISGPLTSANKITLVSSGSGAMGAAKVTGEVLDILSRLPESVERLTGVSISQVN

HKPLRTA

>sp|P29702|FNTA_BOVIN Protein farnesyltransferase/geranylgeranyltransferase type-1 subunit alpha OS=Bos taurus OX=9913 GN=FNTA PE=2 SV=2

MAAADGVGEAAQGGDPGQPEPPPPPQPHPPPPPPQPPQEEAAAASPIDDGFLSLDSPTYV

LYRDRPEWADIDPVPQNDGPNPVVQIIYSEKFQDVYDYFRAVLQRDERSERAFKLTRDAI

ELNAANYTVWHFRRVLLKSLQKDLHEEMNYISAIIEEQPKNYQVWHHRRVLVEWLRDPSQ

ELEFIADILTQDAKNYHAWQHRQWVIQEFKLWDNELQYVDQLLKEDVRNNSVWNQRYFVI

SNTTGYNDRAILEREVQYTLEMIKLVPHNESAWNYLKGILQDRGLSKYPNLLNQLLDLQP

SHSSPYLIAFLVDIYEDMLENQCDNKEDILNKALELCEILAKEKDTIRKEYWRYIGRSLQ

SKHSTESDPPTNVQQ

>sp|Q58D84|FSTL1_BOVIN Follistatin-related protein 1 OS=Bos taurus OX=9913 GN=FSTL1 PE=2 SV=1

MMWRRWLALALVAVAWVHAEEQVRSKSKICANVFCGAGRECAVTEKGEPTCLCIEQCKPH

KRPVCGSNGKTYLNHCELHRDACLTGSKIQVDYDGHCKEKKSVSPSASPVVCYQSNRDEL

RRRIIQWLEAEIIPDGWFSKGSNYSEILDKYFKNFDNGDSRLDSSEFLKFVEQNETAINI

TTYADQENNKLLRGLCVDALIELSDENADWKLSFQEFLKCLNPSFNPPEKKCALEDETYA

DGAETEVDCNRCVCACGNWVCTAMTCDGKNQKGAQTQAEEEMTRYVQELQKHQETAEKSK

RVSTKEI

>sp|Q2KIM0|FUCO_BOVIN Tissue alpha-L-fucosidase OS=Bos taurus OX=9913 GN=FUCA1 PE=2 SV=1

MRSWVVGARLLLLLQLVLVLGAVRLPPCTDPRHCTDPPRYTPDWPSLDSRPLPAWFDEAK

FGVFVHWGVFSVPAWGSEWFWWHWQGEKLPQYESFMKENYPPDFSYADFGPRFTARFFNP

DSWADLFKAAGAKYVVLTTKHHEGYTNWPSPVSWNWNSKDVGPHRDLVGELGTAIRKRNI

RYGLYHSLLEWFHPLYLRDKKNGFKTQYFVNAKTMPELYDLVNRYKPDLIWSDGEWECPD

TYWNSTDFLAWLYNDSPVKDEVVVNDRWGQNCSCHHGGYYNCKDKFQPETLPDHKWEMCT

SIDQRSWGYRRDMEMADITNESTIISELVQTVSLGGNYLLNVGPTKDGLIVPIFQERLLA

VGKWLSINGEAIYASKPWRVQSEKNSVWYTSKGLAVYAILLHWPEYGILSLISPIATSTT

KVTMLGIQKDLKWSLNPSGKGLLVFLPQLPPAALPTEFAWTIKLTGVK

>sp|A5PJI5|GET3_BOVIN ATPase GET3 OS=Bos taurus OX=9913 GN=GET3 PE=2 SV=1

MAAGVAGWGVEAEEFEDAPDVEPLEPTLSNIIEQRSLKWIFVGGKGGVGKTTCSCSLAVQ

LSKGRESVLIISTDPAHNISDAFDQKFSKVPTKVKGYDNLFAMEIDPSLGVAELPDEFFE

EDNMLSMGKKMMQEAMSAFPGIDEAMSYAEVMRLVKGMNFSVVVFDTAPTGHTLRLLNFP

TIVERGLGRLMQIKNQISPFISQMCNMLGLGDMNADQLASKLEETLPVIRSVSEQFKDPE

QTTFICVCIAEFLSLYETERLIQELAKCKIDTHNIIVNQLVFPDPEKPCKMCEARHKIQA

KYLDQMEDLYEDFHIVKLPLLPHEVRGADKVNTFSALLLEPYKPPSAQ

>sp|Q28115|GFAP_BOVIN Glial fibrillary acidic protein OS=Bos taurus OX=9913 GN=GFAP PE=1 SV=2

MERRRVTSATRRSYVSSSEMVVGGRRLGPGTRLSLARMPPPLPARVDFSLAGALNSGFKE

TRASERAEMMELNDRFASYIEKVRFLEQQNKALAAELNQLRAKEPTKLADVYQAELRELR

LRLDQLTANSARLEVERDNLAQDLGTLRQKLQDETNQRLEAENNLAAYRQEADEATLARL

DLERKIESLEEEIRFLRKIHEEEVRELQEQLAQQQVHVEMDVAKPDLTAALREIRTQYEA

VASSNMHEAEEWYRSKFADLNDAAARNAELLRQAKHEANDYRRQLQALTCDLESLRGTNE

SLERQMREQEERHAREAASYQEALARLEEEGQSLKDEMARHLQEYQDLLNVKLALDIEIA

TYRKLLEGEENRITIPVQTFSNLQIRETSLDTKSVSEGHLKRNIVVKTVEMRDGEVIKES

KQEHKDVM

>sp|Q08DQ2|GFPT2_BOVIN Glutamine--fructose-6-phosphate aminotransferase [isomerizing] 2 OS=Bos taurus OX=9913 GN=GFPT2 PE=2 SV=1

MCGIFAYMNYRVPRTRKEIFETLIKGLQRLEYRGYDSAGVAIDGNNNEVKERHIQLVKKR

GNVKALDEELYKQDSMDLKVEFETHFGIAHTRWATHGVPSAVNSHPQRSDKGNEFVVIHN

GIITNYKDLRKFLESKGYEFESETDTETIAKLIKYVFDNRETEDITFSTLVERVIQQLEG

AFALVFKSIHYPGEAVATRRGSPLLIGVRSKYKLSTEQIPVLYRTRNIENVKNICKTRMK

RLDSSTCLHAVGNKAVEFFFASDASAIIEHTNRVIFLEDDDIAAVADGKLSIHRVKRLAS

DDPSRAIQTLQMELQQIMKGNFSAFMQKEIFEQPESVFNTMRGRVNFETNTVLLGGLKDH

LKEIRRCRRLIVIGCGTSYHAAVATRQVLEELTELPVMVELASDFLDRNTPVFRDDVCFF

ISQSGETADTLLALRYCKDRRALTVGVTNTVGSSISRETDCGVHINAGPEIGVASTKAYT

SQFISLVMFGLMMSEDRISLQNRRREIIHGLKSLPELIKEVLSLDEKIHDLALELYTQRS

LLVMGRGYNYATCLEGALKIKEITYMHSEGILAGELKHGPLALIDKQMPVIMVIMKDPCF

AKCQNALQQVTARQGRPIILCSKDDTESSKFAYKTIELPHTVDCLQGILSVIPLQLLSFH

LAVLRGYDVDFPRNLAKSVTVE

>sp|A7YWG4|GGH_BOVIN Gamma-glutamyl hydrolase OS=Bos taurus OX=9913 GN=GGH PE=2 SV=1

MARLGRLLSVLGLVLCGATGLGLSAPPAPTPKKPIIGILMQKCHNKNMRALGKYYIAASY

VKFLESAGARVVPVRLDLKNEEYEKLFKSINGVLFPGGSVNLMRSGYARVAKMFYNLSIK

SFGEGDYFPVWGTCLGFEELIYLVSGESLLTLTDTVGIKLPLNFSRGTLQSRMFQNFPAD

LLLSLAVEPLTAHFHKWSLSVMNFTKNEKLKAFFSILTTNTDGNIDFISTMEGYRYPIYG

VQWHPEKAPYEWGQLRGISHAPNAVKAAFYLAEFFVAEARKSNHHFESDVEETKALIYQY

RPTYTGNVSSFQQSYIFD

>sp|P00366|DHE3_BOVIN Glutamate dehydrogenase 1, mitochondrial OS=Bos taurus OX=9913 GN=GLUD1 PE=1 SV=2

MYRYLGEALLLSRAGPAALGSASADSAALLGWARGQPAAAPQPGLVPPARRHYSEAAADR

EDDPNFFKMVEGFFDRGASIVEDKLVEDLKTRETEEQKRNRVRSILRIIKPCNHVLSLSF

PIRRDDGSWEVIEGYRAQHSQHRTPCKGGIRYSTDVSVDEVKALASLMTYKCAVVDVPFG

GAKAGVKINPKNYTDNELEKITRRFTMELAKKGFIGPGVDVPAPDMSTGEREMSWIADTY

ASTIGHYDINAHACVTGKPISQGGIHGRISATGRGVFHGIENFINEASYMSILGMTPGFG

DKTFVVQGFGNVGLHSMRYLHRFGAKCITVGESDGSIWNPDGIDPKELEDFKLQHGTILG

FPKAKIYEGSILEVDCDILIPAASEKQLTKSNAPRVKAKIIAEGANGPTTPEADKIFLER

NIMVIPDLYLNAGGVTVSYFEWLNNLNHVSYGRLTFKYERDSNYHLLMSVQESLERKFGK

HGGTIPIVPTAEFQDRISGASEKDIVHSGLAYTMERSARQIMRTAMKYNLGLDLRTAAYV

NAIEKVFRVYNEAGVTFT

>sp|Q56JZ9|GMFG_BOVIN Glia maturation factor gamma OS=Bos taurus OX=9913 GN=GMFG PE=2 SV=1

MSDSLVVCEVDPELKEKLRKFRFRKETDNAAIVMKVDKDRQMVVLEEEFQNISPEELKME

LPERQPRFVVYSYKYVHADGRVSYPLCFIFSSPVGCKPEQQMMYAGSKNRLVQTAELTKV

FEIRTTDDLTEAWLKEKLSFFR

>sp|Q2YDJ9|GMPPB_BOVIN Mannose-1-phosphate guanyltransferase beta OS=Bos taurus OX=9913 GN=GMPPB PE=2 SV=1

MKALILVGGYGTRLRPLTLSIPKPLADFCNKPILLHQVEALAAAGVDHVILAVSYMSQVL

EKEMKAQEQKLGIRISMSHEEEPLGTAGPLALARDLLCETADPFFVLNSDVICDFPFEAM

VQFHRHHGQEGSILVTKVEEPSKYGVVVCEADTGRVHRFVEKPQVFVSNKINAGVYILSP

SVLRRIQLQPTSIEKEIFPVMAKEGQLYAMELQGFWMDIGQPKDFLTGMCLFLKSLRQKH

PEQLCSGPGIVGNVLVDPRARIGENCSIGPNVSLGPGVVVEDGVCIRRCTVLRDAHIRSH

SWLESCIVGWRCRVGQWVRMENVTVLGEDVIVNDELYLNGASVLPHKSIGESVPEPRIIM

>sp|P63097|GNAI1_BOVIN Guanine nucleotide-binding protein G(i) subunit alpha-1 OS=Bos taurus OX=9913 GN=GNAI1 PE=1 SV=2

MGCTLSAEDKAAVERSKMIDRNLREDGEKAAREVKLLLLGAGESGKSTIVKQMKIIHEAG

YSEEECKQYKAVVYSNTIQSIIAIIRAMGRLKIDFGDSARADDARQLFVLAGAAEEGFMT

AELAGVIKRLWKDSGVQACFNRSREYQLNDSAAYYLNDLDRIAQPNYIPTQQDVLRTRVK

TTGIVETHFTFKDLHFKMFDVGGQRSERKKWIHCFEGVTAIIFCVALSDYDLVLAEDEEM

NRMHESMKLFDSICNNKWFTDTSIILFLNKKDLFEEKIKKSPLTICYPEYAGSNTYEEAA

AYIQCQFEDLNKRKDTKEIYTHFTCATDTKNVQFVFDAVTDVIIKNNLKDCGLF

>sp|P08239|GNAO_BOVIN Guanine nucleotide-binding protein G(o) subunit alpha OS=Bos taurus OX=9913 GN=GNAO1 PE=1 SV=4

MGCTLSAEERAALERSKAIEKNLKEDGISAAKDVKLLLLGAGESGKSTIVKQMKIIHEDG

FSGEDVKQYKPVVYSNTIQSLAAIVRAMDTLGIEYGDKERKADAKMVCDVVSRMEDTEPF

SPELLSAMMRLWGDSGIQECFNRSREYQLNDSAKYYLDSLDRIGAADYQPTEQDILRTRV

KTTGIVETHFTFKNLHFRLFDVGGQRSERKKWIHCFEDVTAIIFCVALSGYDQVLHEDET

TNRMHESLMLFDSICNNKFFIDTSIILFLNKKDLFGEKIKKSPLTICFPEYTGSNTYEDA

AAYIQAQFESKNRSPNKEIYCHMTCATDTNNIQVVFDAVTDIIIANNLRGCGLY

>sp|P04896|GNAS2_BOVIN Guanine nucleotide-binding protein G(s) subunit alpha isoforms short OS=Bos taurus OX=9913 GN=GNAS PE=1 SV=1

MGCLGNSKTEDQRNEEKAQREANKKIEKQLQKDKQVYRATHRLLLLGAGESGKSTIVKQM

RILHVNGFNGEGGEEDPQAARSNSDGEKATKVQDIKNNLKEAIETIVAAMSNLVPPVELA

NPENQFRVDYILSVMNVPDFDFPPEFYEHAKALWEDEGVRACYERSNEYQLIDCAQYFLD

KIDVIKQDDYVPSDQDLLRCRVLTSGIFETKFQVDKVNFHMFDVGGQRDERRKWIQCFND

VTAIIFVVASSSYNMVIREDNQTNRLQEALNLFKSIWNNRWLRTISVILFLNKQDLLAEK

VLAGKSKIEDYFPEFARYTTPEDATPEPGEDPRVTRAKYFIRDEFLRISTASGDGRHYCY

PHFTCAVDTENIRRVFNDCRDIIQRMHLRQYELL

>sp|P04695|GNAT1_BOVIN Guanine nucleotide-binding protein G(t) subunit alpha-1 OS=Bos taurus OX=9913 GN=GNAT1 PE=1 SV=3

MGAGASAEEKHSRELEKKLKEDAEKDARTVKLLLLGAGESGKSTIVKQMKIIHQDGYSLE

ECLEFIAIIYGNTLQSILAIVRAMTTLNIQYGDSARQDDARKLMHMADTIEEGTMPKEMS

DIIQRLWKDSGIQACFDRASEYQLNDSAGYYLSDLERLVTPGYVPTEQDVLRSRVKTTGI

IETQFSFKDLNFRMFDVGGQRSERKKWIHCFEGVTCIIFIAALSAYDMVLVEDDEVNRMH

ESLHLFNSICNHRYFATTSIVLFLNKKDVFSEKIKKAHLSICFPDYNGPNTYEDAGNYIK

VQFLELNMRRDVKEIYSHMTCATDTQNVKFVFDAVTDIIIKENLKDCGLF

>sp|P04696|GNAT2_BOVIN Guanine nucleotide-binding protein G(t) subunit alpha-2 OS=Bos taurus OX=9913 GN=GNAT2 PE=1 SV=3

MGSGASAEDKELAKRSKELEKKLQEDADKEAKTVKLLLLGAGESGKSTIVKQMKIIHQDG

YSPEECLEYKAIIYGNVLQSILAIIRAMPTLGIDYAEVSCVDNGRQLNNLADSIEEGTMP

PELVEVIRKLWKDGGVQACFDRAAEYQLNDSASYYLNQLDRITAPDYLPNEQDVLRSRVK

TTGIIETKFSVKDLNFRMFDVGGQRSERKKWIHCFEGVTCIIFCAALSAYDMVLVEDDEV

NRMHESLHLFNSICNHKFFAATSIVLFLNKKDLFEEKIKKVHLSICFPEYDGNNSYEDAG

NYIKSQFLDLNMRKDVKEIYSHMTCATDTQNVKFVFDAVTDIIIKENLKDCGLF

>sp|P0C7Q4|GNAT3_BOVIN Guanine nucleotide-binding protein G(t) subunit alpha-3 OS=Bos taurus OX=9913 GN=GNAT3 PE=1 SV=1

MGIGISSESKESAKRSKELEKKLQEDAERDARTVKLLLLGAGESGKSTIVKQMKIIHKNG

YSEQECMEFKAVIYSNTLQSILAIVKAMATLEIDYVNPRSAEDQQQLCAMANTLEDGSMT

PELAEIIKRLWRDPGVQACFERASEYQLNDSAAYYLNDLDRIAAPGYVPNEQDVLHSRVK

TTGIIETQFSFKDLHFRMFDVGGQRSERKKWIHCFEGVTCIIFCAALSAYDMVLVEDEEV

NRMHESLHLFNSICNHKYFATTSIVLFLNKKDLFQEKVTKVHLSICFPEYTGPNTFEDAG

NYIKNQFLDLNLKKEDKEIYSHMTCATDTQNVKFVFDAVTDIIIKENLKDCGLF

>sp|P62871|GBB1_BOVIN Guanine nucleotide-binding protein G(I)/G(S)/G(T) subunit beta-1 OS=Bos taurus OX=9913 GN=GNB1 PE=1 SV=3

MSELDQLRQEAEQLKNQIRDARKACADATLSQITNNIDPVGRIQMRTRRTLRGHLAKIYA

MHWGTDSRLLVSASQDGKLIIWDSYTTNKVHAIPLRSSWVMTCAYAPSGNYVACGGLDNI

CSIYNLKTREGNVRVSRELAGHTGYLSCCRFLDDNQIVTSSGDTTCALWDIETGQQTTTF

TGHTGDVMSLSLAPDTRLFVSGACDASAKLWDVREGMCRQTFTGHESDINAICFFPNGNA

FATGSDDATCRLFDLRADQELMTYSHDNIICGITSVSFSKSGRLLLAGYDDFNCNVWDAL

KADRAGVLAGHDNRVSCLGVTDDGMAVATGSWDSFLKIWN

>sp|Q58CS8|GNPTG_BOVIN N-acetylglucosamine-1-phosphotransferase subunit gamma OS=Bos taurus OX=9913 GN=GNPTG PE=1 SV=4

MAARLAGLAVVLGFAARGPAPGGAAKMKVVEEPNTFGLNNPFLPQTSRLQPKRDPSPVSG

PAHLSRLSGKCFSLVESTYKYELCPFHNVTQHEQTFRWNAYSGILGIWHEWEITNNTFRG

MWMRDGDACQSRSRQSKVELTCGKSNRLAHVSEPSTCVYALTFETPLVCHPHSLLVYPTL

PAALQQRWDQLEQDLVDELITAQGYEKSLRAIFEDAGYLKTSEPNEAAQQEGGTKGLRFE

TLESCQEAHKALSQEIKRLQGVLTQHGVPYGKPTETPSSEHWGPQVPTAGMAEPLRGDPG

LRGDTL

>sp|Q5EA88|GPDA_BOVIN Glycerol-3-phosphate dehydrogenase [NAD(+)], cytoplasmic OS=Bos taurus OX=9913 GN=GPD1 PE=2 SV=3

MTGKKVCIVGSGNWGSAIAKIVGGNAAQLAHFDPRVTMWVFEEDIGGRKLTEIINTQHEN

VKYLPGHKLPPNVVAVPDVVQAAADADILIFVVPHQFIGKICDQLKGHLKADTIGVSLIK

GVDEGPKGLKLISEVIGERLGIPMSVLMGANIANEVADEKFCETTIGSKNQAHGQLLKEL

MQTPNFRITVVQEVDTVEICGALKNIVAVGAGFCDGLGFGDNTKAAVIRLGLMEMIAFAK

LFCSGSVSSATFLESCGVADLITTCYGGRNRKVAEAFARTGKSIEQLEKEMLNGQKLQGP

QTARELHSILQHKGMVDKFPLFTAVYKVCYENQPVGEFIHCLQNHPEHV

>sp|P19687|GCYA1_BOVIN Guanylate cyclase soluble subunit alpha-1 OS=Bos taurus OX=9913 GN=GUCY1A1 PE=1 SV=1

MFCAKLKDLQITGDCPFSLLAPGQVPREPLGEATGSGPASTPGQPGVCPGVPDKNPPGRL

PRRKTSRSRVYLHTLAESICKLIFPEFERLNLALQRTLAKHKIKENRKSLEREDFEKIVV

DQAIAAGVPVEIIKESLGEELFKICYEEDEYILGVVGGTLKDFLNSFSTLLKQSSHCQEA

EKKGRFEDASILCLDKDPDVLYVYYFFPKRITSLILPGIIKAAARILYETEVEVSSTPSR

FHQDCREFVDQPCELYSVHIRSARPHPPPGKPVSSLVIPASLFCKTFPFHFMLDRDMSIL

QLGHGIRRLMSRRDVQGKPHFDEYFEILTPKISQTFSGIMTMLNMQFLVRVRRWDNSMKK

SSRVMDLKGQMIYMVESSSILFLGSPCVDRLEDFTGRGLYLSDIPIHNALRDVVLIGEQA

RAQDGLKKRLGKLKATLEQAHQALEEEKRKTVDLLCSIFPSEVARQLWQGHAVQAKRFGN

VTMLFSDIVGFTAICSQCSPLQVITMLNALYTRFDRQCGELDVYKVETIGDAYCVAGGLH

KESDTHAVQIALMALKMMELSHEVVSPHGEPIKMRIGLHSGSVFAGVVGVKMPRYCLFGN

NVTLANKFESCSVPRKINVSPTTYRLLKDCPGFVFTPRSREELPPNFPSDIPGICHFLEA

YQQGTTSKPWFQKKDVEEANANFLGKASGID

>sp|P02253|H12_BOVIN Histone H1.2 OS=Bos taurus OX=9913 GN=H1-2 PE=1 SV=2

MSETAPAAPAAAPPAEKTPVKKKAAKKPAGARRKASGPPVSELITKAVAASKERSGVSLA

ALKKALAAAGYDVEKNNSRIKLGLKSLVSKGTLVQTKGTGASGSFKLNKKAATGEAKPKA

KKAGAAKPKKAAGAAKKTKKATGAATPKKTAKKTPKKAKKPAAAAVTKKVAKSPKKAKAA

KPKKAAKSAAKAVKPKAAKPKVAKPKKAAPKKK

>sp|A7MAZ5|H13_BOVIN Histone H1.3 OS=Bos taurus OX=9913 GN=H1-3 PE=1 SV=1

MSETAPVAPAAPAPAEKTPVKKKAKKSGVAAGKRKASGPPVSELITKAVAASKERSGVSL

AALKKALAAAGYDVEKNNSRIKLGLKSLVSKGTLVQTKGTGASGSFKLNKKAATGEAKPK

GKKAGAAKPKKAAGAAKKPKKSTGAATPKKAAKKTPKKVKKPAAAAGTKKVAKSPKKAKA

AKPKKPTKSPAKAKAPKPKAAKPKAAKPKATKAKKAVSKKK

>sp|A1A4R1|H2A2C_BOVIN Histone H2A type 2-C OS=Bos taurus OX=9913 GN=H2AC20 PE=2 SV=1

MSGRGKQGGKARAKAKSRSSRAGLQFPVGRVHRLLRKGNYAERVGAGAPVYMAAVLEYLT

AEILELAGNAARDNKKTRIIPRHLQLAIRNDEELNKLLGKVTIAQGGVLPNIQAVLLPKK

TESHKAKSK

>sp|Q2M2T1|H2B1K_BOVIN Histone H2B type 1-K OS=Bos taurus OX=9913 GN=H2BC12 PE=1 SV=3

MPEPAKSAPAPKKGSKKAVTKAQKIDGKKRKRSRKESYSVYVYKVLKQVHPDTGISSKAM

GIMNSFVNDIFERIAGEASRLAHYNKRSTITSREIQTAVRLLLPGELAKHAVSEGTKAVT

KYTSAK

>sp|Q32L48|H2B1N_BOVIN Histone H2B type 1-N OS=Bos taurus OX=9913 GN=H2BC15 PE=1 SV=3

MPEPSKSAPAPKKGSKKAVTKAQKKDGKKRKRSRKESYSVYVYKVLKQVHPDTGISSKAM

GNMNSFVNDIFERIAGEASRLAHYNKRSTITSREIQTAVRLLLPGELAKHAVSEGTKAVT

KYTSSK

>sp|P62808|H2B1_BOVIN Histone H2B type 1 OS=Bos taurus OX=9913 PE=1 SV=2

MPEPAKSAPAPKKGSKKAVTKAQKKDGKKRKRSRKESYSVYVYKVLKQVHPDTGISSKAM

GIMNSFVNDIFERIAGEASRLAHYNKRSTITSREIQTAVRLLLPGELAKHAVSEGTKAVT

KYTSSK

>sp|P62803|H4_BOVIN Histone H4 OS=Bos taurus OX=9913 PE=1 SV=2

MSGRGKGGKGLGKGGAKRHRKVLRDNIQGITKPAIRRLARRGGVKRISGLIYEETRGVLK

VFLENVIRDAVTYTEHAKRKTVTAMDVVYALKRQGRTLYGFGG

>sp|Q2TBU0|HPT_BOVIN Haptoglobin OS=Bos taurus OX=9913 GN=HP PE=2 SV=1

MSALQAVVTLLLCGQLLAVETGSEATADSCPKAPEIANSHVEYSVRYQCDKYYKLHAGNG

VYTFNNKQWINKDIGQQLPECEEDDSCPEPPKIENGYVEYLVRYQCKPYYTLRTCGDGVY

TFNSKKQWINKNIGQKLPECEAVCGKPKHPVDQVQRIIGGSLDAKGSFPWQAKMVSQHNL

ISGATLINERWLLTTAKNLYLGHSSDKKAKDITPTLRLYVGKNQLVEVEKVVLHPDHSKV

DIGLIKLRQKVPVNDKVMPICLPSKDYVKVGRVGYVSGWGRNENFNFTEHLKYVMLPVAD

QDKCVKHYEGVDAPKNKTAKSPVGVQPILNENTFCVGLSKYQDDTCYGDAGSAFVVHDKE

DDTWYAAGILSFDKSCAVAEYGVYVKVTSILDWVRKTIANN

>sp|P31081|CH60_BOVIN 60 kDa heat shock protein, mitochondrial OS=Bos taurus OX=9913 GN=HSPD1 PE=1 SV=2

MLRLPAVLRQMRPVSRALALHLTRAYAKDVKFGADARALMLQGVDLLADAVAVTMGPKGR

TVIIEQSWGSPRVTKDGVTVAKSIDLKDKYKNIGAKLVQDVANNTNEEAGDGTTTATVLA

RSIAKEGFEKISKGANPVEIRRGVMLAVDAVIVELKKQSKPVTTPEEIAQVATISANGDK

EIGNIISDAMKKVGRKGVITVKDGKTLNDELEIIEGMKFDRGYISPYFINTSKGQKCEFQ

DAYVLLSEKKISSVQSIVPALEIANAHRKPLVIIAEDVDGEALSTLVLNRLKVGLQVVAV

KAPGFGDNRKNQLKDMAIATGGAVFGEEGLNLNLEDVQPHDLGKVGEVIVTKDDAMLLKG

KGDKAQIEKRIQEIIEQLDITTSEYEKEKLNERLAKLSDGVAVLKVGGTSDVEVNEKKDR

VTDALNATRAAVEEGIVLGGDCALLRCIPALESITPANEDQKTGIEIIKKTLKIPAMTIA

KNAGVEGSLIVEKIMQSSSEVGYDAMLGDFVNMVEKGIIDPTKVVRTALLDAAGVASLLT

TAEVVVTEIPKEEKDPGMGGMGGMGGGMGGGMF

>sp|Q04467|IDHP_BOVIN Isocitrate dehydrogenase [NADP], mitochondrial OS=Bos taurus OX=9913 GN=IDH2 PE=1 SV=2

MAGYLRVVRSLCRASGSGSAWAPAALTAPNLQEQPRRHYADKRIKVAKPVVEMDGDEMTR

IIWQFIKEKLILPHVDVQLKYFDLGLPNRDQTNDQVTIDSALATQKYSVAVKCATITPDE

ARVEEFKLKKMWKSPNGTIRNILGGTVFREPIICKNIPRLVPGWTKPITIGRHAHGDQYK

ATDFVVDRAGTFKVVFTPKDGSGPKEWEVYNFPAGGVGMGMYNTDESISGFAHSCFQYAI

QKKWPLYMSTKNTILKAYDGRFKDIFQAIFEKHYKTEFDKHKIWYEHRLIDDMVAQVLKS

SGGFVWACKNYDGDVQSDILAQGFGSLGLMTSVLVCPDGKTIEAEAAHGTVTRHYREHQK

GRPTSTNPIASIFAWTRGLEHRGKLDGNQDLIRFAQTLEKVCVETVESGAMTKDLAGCIH

GLSNVKLNEHFLNTSDFLDTIKSNLDRALGQQ

>sp|Q2KJ63|KLKB1_BOVIN Plasma kallikrein OS=Bos taurus OX=9913 GN=KLKB1 PE=2 SV=1

MIALRQAAYFICLFATVSCGCLTQLYHNIFFRGGDVSAMYTPDAQYCQLMCTFHPRCLLF

SFLPENSTSDADKRFGCFLKDSVTGTLPRVSRTGAISGHSLKRCGHQISACHRSIYKGID

MRGVNFNASKVRSAKECQERCTNNIHCQFFTYATKTFFSAEYRNTCLLKRSPQGTPTRIK

VLSDVESGFSLKACGNSKIGCRVDIFQHSAFSDVDVAGIIAPDAFVCRTICTYHPSCLFF

TFYTNAWKTDSQRNVCFLKTSQSGSPSSPTPQENAISGYSLLTCKQTLPGTEPCHSKIYP

QVAFEGEELHVTFVKGVDGCQETCTKMIRCQFFTYSLFPEDCRGEKCKCSLRLSLDGSPT

NITYGTQASSGYSLRLCKRGDSRVCTTKRTRIVGGTNASWGEWPWQVSLQVKQRAQSHLC

GGSIIGRQWVLTAAHCFDGLLLSNIWRIYGGILNLSEITTETSFSQIKEIIVHPNYKISE

GSHDIALIKLEAPLNFTDLQKAICLPSKDDTKPVYTDCWITGWGFTEEKGKIQNTLQKAN

IPLISNEECQKSYRDYKITKQMICAGYKEGGKDACKGDSGGPLVCQHEETWHLVGITSWG

EGCARREQPGVYTKVAEYVDWILEKTQDSHGQPLRK

>sp|P01044|KNG1_BOVIN Kininogen-1 OS=Bos taurus OX=9913 GN=KNG1 PE=1 SV=1

MKLITILFLCSRLLPSLTQESSQEIDCNDQDVFKAVDAALTKYNSENKSGNQFVLYRITE

VARMDNPDTFYSLKYQIKEGDCPFQSNKTWQDCDYKDSAQAATGECTATVAKRGNMKFSV

AIQTCLITPAEGPVVTAQYECLGCVHPISTKSPDLEPVLRYAIQYFNNNTSHSHLFDLKE

VKRAQRQVVSGWNYEVNYSIAQTNCSKEEFSFLTPDCKSLSSGDTGECTDKAHVDVKLRI

SSFSQKCDLYPVKDFVQPPTRLCAGCPKPIPVDSPDLEEPLSHSIAKLNAEHDGAFYFKI

DTVKKATVQVVAGLKYSIVFIARETTCSKGSNEELTKSCEINIHGQILHCDANVYVVPWE

EKVYPTVNCQPLGQTSLMKRPPGFSPFRSVQVMKTEGSTTVSLPHSAMSPVQDEERDSGK

EQGPTHGHGWDHGKQIKLHGLGLGHKHKHDQGHGHHGSHGLGHGHQKQHGLGHGHKHGHG

HGKHKNKGKNNGKHYDWRTPYLASSYEDSTTSSAQTQEKTEETTLSSLAQPGVAITFPDF

QDSDLIATVMPNTLPPHTESDDDWIPDIQTEPNSLAFKLISDFPETTSPKCPSRPWKPVN

GVNPTVEMKESHDFDLVDALL

>sp|Q0P5J4|K1C25_BOVIN Keratin, type I cytoskeletal 25 OS=Bos taurus OX=9913 GN=KRT25 PE=2 SV=1

MSLRLPSGSRRASPRPTTGSLRLSSGGASFGAGNACSMPGIGSSFSCAFGGSSSGGNALG

GNPCAGFTVNEGGLLSGNEKVTMQNLNDRLASYLENVRALEEANADLEQKIKGWYEKFGP

GSCRGLDHDYSRYFPIIEDLKNQIIASTTSNANAVLQIDNARLTADDFRLKYENELALHQ

SVESDVNGLRRVLDEITLCRTDLEIQYETLSEELTYLKKNHKEEMQVLQCAAGGNVNVEM

NAAPGVDLTVLLNNMRAEYEALAEQNRRDAEAWFNEKSASLQQQITEDVGATTSARNELT

EMKRNLQTLEIELQSLLATKHSLECSLTETEGNYCAQLAQIQAQIGALEEQLHQVRTETE

GQKLEYEQLLDIKVHLEKEIETYCLLIGGDDGACKSGGYKSKDYGAGNVGNQMKDPVKAI

VVKKVLEEVDQRSKILTPRLHSLEEKSQSN

>sp|Q0P5J6|K1C27_BOVIN Keratin, type I cytoskeletal 27 OS=Bos taurus OX=9913 GN=KRT27 PE=2 SV=1

MSVRFSSASRRLGSCGGAGSVRLSGGGAGFGVGSTGSVPGFGSGFTCAFGGSSSAGSYSG

GLGGGSASCTAFTGNEHGLLSGNEKVTMQNLNDRLASYLDNVRALEEANADLEQKIKGWY

EKFGPGSCRGLDHDYSRYFTVIDDLRNQIISATTSNANIVLQNDNARLTADDFRLKFENE

QALHQSVDADVSSLRRVLDELTLCRTDLEIQLETLSEELAYLKKNHEEEMKALQCAAGGN

VNVEMNAAPGVDLTVLLNNMRAEYEALAEQNRRDAEAWFNEKSASLQQQISDDAGATTSA

RNELTEMKRNLQTLEIELQSLLATKHSLECSLTETEGNYCAQLAQIQAQIGALEEQLHQV

RTETEGQKLEYEQLLDIKVHLEKEIETYCRLIDGEDGSCAKSKGYGGPGHQIKDPSKATV

VKTIVEEIDPRGKVLSSRVHSVEEKSTKVNNVKSEQRVPS

>sp|Q5XQN5|K2C5_BOVIN Keratin, type II cytoskeletal 5 OS=Bos taurus OX=9913 GN=KRT5 PE=1 SV=1

MSRQSTVSFRSGGGRSFSTASAITPSVSRTSFTSVSRSGGGGGGGFGRVSLGGAYGAGGF

GSRSLYNLGGSKRISISASGGGFRNRFGAGAGGGYGFGGGAGSGFGFGGGAGGGGFGLGG

GAGFGGGFGGPGFPVCPPGGIQEVTVNQSLLTPLNLQIDPTIQRVRTEEREQIKTLNNKF

ASFIDKVRFLEQQNKVLDTKWALLQEQGTKTVRQNLEPLLEQYINNLRRQLDGIVGERGR

LDSELRNMQDLVEDFKNKYEDEINKRTTAENEFVMLKKDVDAAYMNKVELEAKVDALMDE

INFMKMFFDAELSQMQTHVSDTSVVLSMDNNRSLDLDSIIAEVKAQYEDIANRSRTEAES

WYQTKYEELQQTAGRHGDDLRNTKHEISEMNRMIQRLRSEIDNVKKQCANLQNAIADAEQ

RGELALKDARSKLAELEDALQKAKQDMARLLREYQELMNTKLALDVEIATYRKLLEGEEC

RLSGEGVGPVNISVVTNTVSSGYGGGSGFGGGLGGGLGGGLGGGLGGGLGGGLGSGLGGG

GSSSFYSSSSGGVGLGGGLSVGGSGFSASSGRSLGFGSGGGSSSSVKFVSTTSSSRKSFK

S

>sp|Q29S21|K2C7_BOVIN Keratin, type II cytoskeletal 7 OS=Bos taurus OX=9913 GN=KRT7 PE=2 SV=1

MSLHFGSQVFSSRSAAFPGRGTQVRLSSVRPGGFGSSSSLYGLGASRPRVAARSSYGAPV

GTGIRAVTINQSLLTPLQVDIDPSIQQVRQEEREQIKTLNNKFASFIDKVRFLEQQNKLL

ETKWALLQEQKSAKSNRLPGIFEAQIAGLRKQLEALQLDGGRLEVELRNMQDVVEDFKNK

YEDEINHRTAAENEFVVLKKDVDVAYMNKVELEAKVDTLNDEINFLRTLYEQELKELQSE

VSDTSVVLSMDNNRSLDLDSIIAEVKAQYEEIANRSRAEAEACYQTKFETLQAQAGKHGD

DLQNTRNEIADMNRAVQRLQAEIDSVKNQRSKLEAAIADAEQRGELAVKDARAKQEDLEA

ALQKAKQDMTRQLREYQELMNVKLALDIEIATYRKLLEGEESRLTGDGVGAVNISVVSST

GGSGSLLTFGGTMGNNALRFSSGGGPGTLKAYSMRTTSATSRSPRK

>sp|Q08D91|K2C75_BOVIN Keratin, type II cytoskeletal 75 OS=Bos taurus OX=9913 GN=KRT75 PE=2 SV=1

MSRQSTITFQTSSRRGFSTASATTPATSRSRFSSASVTHSPAGSGGLGRISGFGSRSLYN

LGGTKRVSISGCGSNFRSGFGGRASSGFGVSGGFGYGGGIGGGHGGCGFSVCPPGGIQEV

TVNQSLLTPLNLQIDPNIQRVRKEEREQIKTLNNKFASFIDKVRFLEQQNKVLETKWSLL

QEQGTRTVRQSLEPFFEAYITDLRRQLDSITTERGRLDAELRTMQDVVEDFKVRYEDEIN

KRTAAENEFVALKKDVDAAYLNKVDLEAKANSLTDEINFLQMLFEAELCQMQTRVSDTSV

VLSMDNNRSLDLDSIIAEVKAQYEEIANRSRAEAESWYQTKYEELQVTAGQHGDDLRNTK

QEISETNRMIQRLRAEIDNVKKQCASLQTAIADAEQRGELALKDARAKLVDLEEALQKSK

QDMARLLREYQELMNIKLALDVEIATYRKLLEGEECRLSGEGVSPVNISVVTSTVSSGYG

GGSSIGSGSLGLSGGSGCSFMTSGGHSLGGSSFSNSSSRGLGGSGSSFKFVSTTSSSRKS

YKH

>sp|Q148H7|K2C79_BOVIN Keratin, type II cytoskeletal 79 OS=Bos taurus OX=9913 GN=KRT79 PE=2 SV=1

MRSSVSRQTYSTKGAFSSSSASGGGGSQARTSFSSVTVSRNSGRGGGPRCGPSMGGFGSQ

SLYNLGGSKSISVSVAGGASAGRVLGGFGLGGGAYMGLGACRPMLGPVCPPGGIQQVTVN

QSLLTPLHVEIDPEIQRVRTEEREQIKTLNNKFASFIDKVRFLEQQNKVLETKWALLQEQ

GQKSGVTRNNLEPLFEHFINNLRGKLDNLQSERGRLDSELRNVQDLAEDFKTKYEDEINK

RTAAENEFVVLKKDVDAAYVGRMDLHGMVDHLMGEIDFLRHLYEEELSQVQTHVSDTSVI

LSMDNNRNLDLDSIIAEVKAQYEQIAQRSRAEAESWYQTKYEELQVTAGKHGDNLRDTKN

EIAELTRTVQRLQGEADAVKKQCQQLQTAIADAEQHGELALKDAQKKLGDLDAALNQAKE

DLARLLRDYQALMNVKLALDVEIATYRKLLESEESRMSGECPSAVSISVTGNSTTVCGGG

AAGFGGGISLGGGGGASKGRFSTNAGYSTVKGGPVSGGTSILRKTTTVKTSSRRY

>sp|P05786|K2C8_BOVIN Keratin, type II cytoskeletal 8 OS=Bos taurus OX=9913 GN=KRT8 PE=2 SV=3

MSIRVTQKSYKVSTSAPRSFSSRSYTSGPGSRISSSAFSRVGSSSSFRGGLGTGMSMAGS

YGGAPGLGGITAVTVNQSLLSPLKLEVDPNIQAVRTQEKEQIKTLNNKFASFIDKVRHLE

QQNKVLETKWNLLQQQKTARSNIDNMFESYINNLRRQLETLAQEKLKLEVELGNMQGLVE

DFKTKYEDEIQKRTDMENEFVIIKKDVDEAYMNKVELESRLEGLTDEINFYRQLYEEEIR

EMQSQISDTSVVLEMDNNRNLDLDGIIAEVKAQYEEIANRSRAEAEAMYQIKYEELQTLA

GKHGDDLRRTKTEISEMNRNINRLQAEIEGLKGQRASLEAAIADAEQRGEMAVKDAQAKL

AELEAALRNAKQDMARQLREYQELMNVKLALDVEIATYRKLLEGEESRLESGMQNMSIHT

KTTSGYAGGLTSSYGTPGFNYSLSPGSFSRTSSKPVVVKKIETRDGKLVSESSDVLSK

>sp|Q3SZH7|LKHA4_BOVIN Leukotriene A-4 hydrolase OS=Bos taurus OX=9913 GN=LTA4H PE=2 SV=3

MPEVVDTCSLASPASVCQTKHLHLRCSIDFTRRVLSGTAALTIQSQEDNLRSLILDTKDL

TIEKVVINGQEVKYTLGERQSYKGSPIEISLPIALCKNQEIVIEISFETSPKSSALQWLT

PEQTSGKEHPYLFSQCQAIHCRAILPCQDTPSVKLTYSAEVSVPKELVALMSAIRDGEAP

DPEDPNRKIYRFSQKVPIPCYLIALVVGALESRQIGPRTLVWSEKEQVEKSAYEFSETES

MLKIAEDLGGPYIWGQYDLLVLPPSFPYGGMENPCLTFVTPTLLAGDKSLSNVIAHEISH

SWTGNLVTNKTWDHFWLNEGHTVYLERHICGRLFGEKFRHFHALGGWGELQNSIKTFGET

HPFTKLVVDLTNTDPDVAYSSVPYEKGFALLFYLEQLLGGPEVFLGFLKAYVEKFSYKSI

TTDNWKDFLYSHFKDKVDILNQVDWNTWLYSPGLPPVKPNYDMTLTNACISLSQRWITAK

DDDLNSFSSADLKDFSSHQVNEFLAQMLQNAPLPLGHIKRMQEVYNFNAINNSEIRFRWL

RLCIQSKWEEAIPLALKMATEQGRMKFTRPLFKDLAAFDKSHDQAIRTYKEHKASMHPVT

AMLVGKDLKVD

>sp|P12624|MARCS_BOVIN Myristoylated alanine-rich C-kinase substrate OS=Bos taurus OX=9913 GN=MARCKS PE=1 SV=6

MGAQFSKTAAKGEATAERPGEAAVASSPSKANGQENGHVKVNGDASPAAAEPGAKEELQA

NGSAPAADKEEPAAAGSGAASPAAAEKDEPAAAAPDAGASPVEKEAPVEGEAAEPGSPTA

AEGEAASAASSTSSPKAEDGATPSPSNETPKKKKKRFSFKKSFKLSGFSFKKNKKEAGEG

GEAEGAAGASAEGGKDEASGGAAAAAGEAGAAPGEPTAAPGEEAAAGEEGAAGGDPQEAK

PEEAAVAPEKPPASEEAKAVEEPSKAEEKAEEAGVSAAGCEAPSAAGPGVPPEQEAAPAE

EAAAAPASSACAAPSQEAQPECSPEAPPAEAAE

>sp|Q3T145|MDHC_BOVIN Malate dehydrogenase, cytoplasmic OS=Bos taurus OX=9913 GN=MDH1 PE=2 SV=3

MSEPIRVLVTGAAGQIAYSLLYSIGNGSVFGKDQPIILVLLDITPMMGVLDGVLMELQDC

ALPLLKDVIATDKEEIAFKDLDVAILVGSMPRRDGMERKDLLKANVKIFKCQGAALDKYA

KKSVKVIVVGNPANTNCLTASKSAPSIPKENFSCLTRLDHNRAKAQIALKLGVTSDDVKN

VIIWGNHSSTQYPDVNHAKVKLQGKEVGVYEALKDDSWLKGEFITTVQQRGAAVIKARKL

SSAMSAAKAICDHVRDIWFGTPEGEFVSMGIISDGNSYGIPDDLLYSFPVTIKDKTWKVV

EGLPINDFSREKMDLTAKELAEEKETAFEFLASA

>sp|P07507|MGP_BOVIN Matrix Gla protein OS=Bos taurus OX=9913 GN=MGP PE=1 SV=1

MKSLLLLSILAALAVAALCYESHESLESYEINPFINRRNANSFISPQQRWRAKAQERIRE

LNKPQYELNREACDDFKLCERYAMVYGYNAAYDRYFRQRRGAK

>sp|Q2HJ49|MOES_BOVIN Moesin OS=Bos taurus OX=9913 GN=MSN PE=2 SV=3

MPKTINVRVTTMDAELEFAIQPNTTGKQLFDQVVKTIGLREIWFFGLQYQDTKGFFTWLK

LNKKVTAQDVRKESPLLFKFRAKFYPEDVSEELIQDITQRLFFLQVKEDILNDDIYCPPE

TAVLLASYAVQSKYGDFNKEVHKSGYLAGDRLLPQRVLEQHKLNKDQWEERIQVWHEEHR

GMLREDAVLEYLKIAQDLEMYGVNYFSIKNKKGSELWLGVDALGLNIYEQNDRLTPKIGF

PWSEIRNISFNDKKFVIKPIDKKAPDFVFYAPRLRINKRILALCMGNHELYMRRRKPDTI

EVQQMKAQAREEKHQKQMERALLENEKKKREMAEKEKEKIEREKEELMERLKQIEEQTKK

AQQELEEQTRRALELEQERKRAQSEAEKLAKERQEAEEAKEALLQASQDQKKTQEQLALE

MAELTARISQLEMARQKKESEAVEWQQKAQMVQEDLEKTRAELKTAMSTPHVAEPAENEQ

DEQDENGAEASAELRADAMAKDRSEEERTTEAEKNERVQKHLKALTSELANARDESKKTA

NDMIHAENMRLGRDKYKTLRQIRQGNTKQRIDEFESM

>sp|Q27991|MYH10_BOVIN Myosin-10 OS=Bos taurus OX=9913 GN=MYH10 PE=2 SV=2

MAQRTGLEDPERYLFVDRAVIYNPATQADWTAKKLVWIPSERHGFEAASIKEERGDEVLV

ELAENGKKAMVNKDDIQKMNPPKFSKVEDMAELTCLNEASVLHNLKDRYYSGLIYTYSGL

FCVVINPYKNLPIYSENIIEMYRGKKRHEMPPHIYAISESAYRCMLQDREDQSILCTGES

GAGKTENTKKVIQYLAHVASSHKGRKDHNIPGELERQLLQANPILESFGNAKTVKNDNSS

RFGKFIRINFDVTGYIVGANIETYLLEKSRAVRQAKDERTFHIFYQLLSGAGEHLKSDLL

LEGFNNYRFLSNGYIPIPGQQDKDNFQETMEAMHIMGFSHEEILSMLKVVSSVLQFGNIS

FKKERNTDQASMPENTVAQKLCHLLGMNVMEFTRAILTPRIKVGRDYVQKAQTKEQADFA

VEALAKATYERLFRWLVHRINKALDRTKRQGASFIGILDIAGFEIFELNSFEQLCINYTN

EKLQQLFNHTMFILEQEEYQREGIEWNFIDFGLDLQPCIDLIERPANPPGVLALLDEECW

FPKATDKTFVEKLVQEQGSHSKFQKPRQLKDKADFCIIHYAGKVDYKADEWLMKNMDPLN

DNVATLLHQSSDRFVAELWKDVDRIVGLDQVTGMTETAFGSAYKTKKGMFRTVGQLYKES

LTKLMATLRNTNPNFVRCIIPNHEKRAGKLDPHLVLDQLRCNGVLEGIRICRQGFPNRIV

FQEFRQRYEILTPNAIPKGFMDGKQACERMIRALELDPNLYRIGQSKIFFRAGVLAHLEE

ERDLKITDIIIFFQAVCRGYLARKAFAKKQQQLSALKVLQRNCAAYLKLRHWQWWRVFTK

VKPLLQVTRQEEELQAKDEELLKVKEKQTKVEGELEEMERKHQQLLEEKNILAEQLQAET

ELFAEAEEMRARLAAKKQELEEILHDLESRVEEEEERNQILQNEKKKMQAHIQDLEEQLD

EEEGARQKLQLEKVTAEAKIKKMEEEILLLEDQNSKFIKEKKLMEDRIAECSSQLAEEEE

KAKNLAKIRNKQEVMISDLEERLKKEEKTRQELEKAKRKLDGETTDLQDQIAELQAQIDE

LKIQVAKKEEELQGALARGDDETLHKNNALKVVRELQAQIAELQEDFESEKASRNKAEKQ

KRDLSEELEALKTELEDTLDTTAAQQELRTKREQEVAELKKALEEETKSHEAQIQDMRQR

HATALEELSEQLEQAKRFKANLEKNKQGLETDNKELACEVKVLQQVKAESEHKRKKLDAQ

VQELHAKVSEGDRLRVELAEKANKLQNELDNVSTLLEEAEKKGIKFAKDAAGLESQLQDT

QELLQEETRQKLNLSSRIRQLEEERSSLQEQQEEEEEARRSLEKQLQALQAQLTDTKKKV

DDDLGTIENLEEAKKKLLKDVEVLSQRLEEKALAYDKLEKTKTRLQQELDDLLVDLDHQR

QIVSNLEKKQKKFDQLLAEEKNISARYAEERDRAEAEAREKETKALSLARALEEALEARE

EAERQNKQLRADMEDLMSSKDDVGKNVHELEKSKRALEQQVEEMRTQLEELEDELQATED

AKLRLEVNMQAMKAQFERDLQTRDEQNEEKKRLLIKQVRELEAELEDERKQRALAVASKK

KMEIDLKDLEAQIEAANKARDEVIKQLRKLQAQMKDYQRELEEARASRDEIFAQSKESEK

KLKSLEAEILQLQEELASSERARRHAEQERDELADEIANSASGKSALLDEKRRLEARIAQ

LEEELEEEQSNMELLNDRFRKTTLQVDTLNTELAAERSAAQKSDNARQQLERQNKELKAK

LQELEGAVKSKFKATISALEAKIGQLEEQLEQEAKERAAANKLVRRTEKKLKEIFMQVED

ERRHADQYKEQMEKANARMKQLKRQLEEAEEEATRANASRRKLQRELDDATEANEGLSRE

VSTLKNRLRRGGPISFSSSRSGRRQLHIEGASLELSDDDTESKTSDINETQPPQSE

>sp|P60661|MYL6_BOVIN Myosin light polypeptide 6 OS=Bos taurus OX=9913 GN=MYL6 PE=2 SV=2

MCDFTEDQTAEFKEAFQLFDRTGDGKILYSQCGDVMRALGQNPTNAEVLKVLGNPKSDEM

NVKVLDFEHFLPMLQTVAKNKDQGTYEDYVEGLRVFDKEGNGTVMGAEIRHVLVTLGEKM

TEEEVEMLVAGHEDSNGCINYEAFVRHILSG

>sp|Q28824|MYLK_BOVIN Myosin light chain kinase, smooth muscle OS=Bos taurus OX=9913 GN=MYLK PE=1 SV=1

MDFRANLQRQVKPKTLSEEERKVHGPQQVDFRSVLAKKGTPKTPVPEKVPPPKPATPDFR

SVLGSKKKLPTENGSNNTEALNAKAAEGLKPVGNAQPSGFLKPVGNAKLADTPKPLSSTK

PAETPKPLGNVKPAETPKPLGSTKPAETPKPLGSTKPAETPKPLGNVKPAETPKPLGNIK

PTETPKPLGSTKPAETPKPLGSTKPAETPKPLGNVKPAETPKPLGNVKPAETPKPLGNVK

PAETPKPVSNAKPAETLKPVGNAKPAETPKPLSNVKPAETPKLVGNAKPAETSKPLDNAK

PAEAPKPLGNAKPAEIPKPTGKEELKKEIKNDVNCKKGHAGATDSEKRPESRGTAPTFEE

KLQDLHVAEGQKLLLQCRVSSDPPATITWTLNGKTLKTTKFIVLSQEGSLCSVSIEKALP

EDRGLYKCVAKNSAGQAESSCQVTVDVPDAPTSENAKAPEMKARRPKSSLPPVLGTESDA

TVKKKPAPKTPPKAAMPPQIIQFPEDQKVRAGESVELFGKVAGTQPITCTWMKFRKQIQD

SEHIKVENSEQGSKLTIRAARQEHCGCYTLLVENKLGSRQAQVNLTVVDKPDPPAGTPCA

SDIRSSSLTLSWYGSSYDGGSAVQSYSVEIWDSVDKTWKELATCRSTSFNVQDLLPDREY

KFRVRAINVYGTSEPSQESELTALGEKPEEEPKDEVEVSDDDEKEPEVDYRTVTVNTEQK

VSDFYDIEERLGSGKFGQVFRLVEKKTGKIWAGKFFKAYSAKEKENIRQEISIMNCLHHP

KLVQCVDAFEEKANIVMVLEIVSGGELFERIIDEDFELTERECIKYMKQISEGVEYIHKQ

GIVHLDLKPENIMCVNKTGTRIKLIDFGLARRLENAGSLKVLFGTPEFVAPEVINYEPIG

YATDMWSIGVICYILVSGLSPFMGDNDNETLANVTSATWDFDDEAFDEISDDAKDFISNL

LKKDMKNRLNCTQCLQHPWLMKDTKNMEAKKLSKDRMKKYMARRKWQKTGNAVRAIGRLS

SMAMISGLSGRKSSTGSPTSPLNAEKLESEDVSQAFLEAVAEEKPHVKPYFSKTIRDLEV

VEGSAARFDCKIEGYPDPEVVWFKDDQSIRESRHFQIDYDEDGNCSLIISDVCGDDDAKY

TCKAVNSLGEATCTAELIVETMEEGEGEGGEEEEEE

>sp|A6H767|NP1L1_BOVIN Nucleosome assembly protein 1-like 1 OS=Bos taurus OX=9913 GN=NAP1L1 PE=2 SV=1

MADIDNKEQSELDQDLDDVEEVEEEETGEETKIKARQLTVQMMQNPQILAALQERLDGLV

ETPTGYIESLPRVVKRRVNALKNLQVKCAQIEAKFYEEVHDLERKYAVLYQPLFDKRFEI

INAIYEPTEEECEWKPDEEDEISEELKEKAKVEDEKKDEEKEDPKGIPEFWLTVFKNVDL

LSDMVQEHDEPILKHLKDIKVKFSDAGQPMSFVLEFHFEPNEYFTNEVLTKTYRMRSEPD

DSDPFSFDGPEIMGCTGCQIDWKKGKNVTLKTIKKKQKHKGRGTVRTVTKTVSNDSFFNF

FAPPEVPESGDLDDDSEAILAADFEIGHFLRERIIPRSVLYFTGEAIEDDDDDYDEEGEE

ADEEGEEEGDEENDPDYDPKKDQNPAECKQQ

>sp|P81125|SNAA_BOVIN Alpha-soluble NSF attachment protein OS=Bos taurus OX=9913 GN=NAPA PE=1 SV=1

MDNSGKEAEAMALLAEAERKVKNSQSFFSGLFGGSSKIEEACEIYARAANMFKMAKNWSA

AGSAFCQAAHVHLQLQSKHDAATCFVDAGNAFKKADPQEAINCLMRAIEIYTDMGRFTIA

AKHHISIAEIYETELVDIEKAIAHYEQSADYYKGEESNSSANKCLLKVAGYAAQLEQYQK

AIDIYEQVGTNAMDSPLLKYSAKDYFFKAALCHFCIDMLNAKLAVQKYEELFPAFSDSRE

CKRIKKLLEAHEEQNVDSYTEAVKEYDSISRLDQWLTTMLLRIKKTIQGDEEDLR

>sp|A5PK51|PNCB_BOVIN Nicotinate phosphoribosyltransferase OS=Bos taurus OX=9913 GN=NAPRT PE=2 SV=2

MAAEQDPEGRAAARPLLTDLYQATMALGYWRAGRAQDQAEFELFFRQCPFGGAFALAAGL

RDCVRFLRAFRLRDADVQFLASALPPDTDPAFFEHLRALDCSGVTVRALPEGSLAFPGVP

LLQVSGPLLVVQLLETPLLCLVSYASLIATNAARLRLIAGPDKRLLEMGLRRAQGPDGGL

TASTYSYLGGFDASSNVLAGQLRGVPVAGTLAHSFVTSFSGSEVPPDPMLAPAAGQGSQV

DLAASVEMWLERVCGHLGLGVQEPHRGERAAFVAYALAFPRAFQGLLDTYSVRRSGLPNF

LAVALALQELGYQAVGVRLDSGDLLQQAREIRGVFRTAAAQFGVPWLQSVPIAVSNNIDE

EELARLAQKGSEVNVIGIGTSVVTCPRQPSLGCVYKLVSVGGQPRMKLTEDPEKQTLPGS

KAAFRLLGSDGSLLLDVLQLAEEPPPQAGQELRVWPRGARESRTVRPAHVEPLLRLWVQQ

GQLCEPLPSLAESRAFAQQSLHRLSPAHRRLEQPALYQVALSEKLQALVDRLSARGAL

>sp|Q2KJG3|SYNC_BOVIN Asparagine--tRNA ligase, cytoplasmic OS=Bos taurus OX=9913 GN=NARS PE=2 SV=3

MSLEVVRAAAGMVLAELYVSDREGNDVTGDGTKEKPFKTGLKALMTVGKEPFPTIYVDSQ

KENERWDVISKSQMKNIRKLWHREQMKSESREKKEAEDNLRREKNLEEAKKITIKNDPSL

PEPKCVKIRELKGYRGQRIKVFGWVHRLRRQGKNLMFLVLRDGTGFLQCVLSDDLCQCYN

GVVLSTESSVAVYGVLNLTPKGKQAPGGHELSCDFWELIGLAPAGGADNLINEESDVDVQ

LNNRHMMIRGENMSKILKARSVITRCFRDHFFDRGYHEITPPTLVQTQVEGGATLFKLDY

FGEEAYLTQSSQLYLETCIPALGDVFCIAQSYRAEQSRTRRHLAEYTHVEAECPFLTFEE

LLNRLEDLVCDVVDRVLKSPAGNIVRDLNPNFKPPKRPFKRMNYSDAIVWLKEHNIKKED

GTFYEFGEDIPEAPERLMTDTINEPILLCRFPVEIKSFYMQRCPEDPRLTESVDVLMPNV

GEIVGGSMRIWDNEEILAGYKREGIDPTPYYWYTDQRKYGTCPHGGYGLGLERFLTWILD

RYHIRDVCLYPRFVQRCKP

>sp|Q3ZBA8|NDRG2_BOVIN Protein NDRG2 OS=Bos taurus OX=9913 GN=NDRG2 PE=2 SV=1

MAELREVQITEEKPLLPGQTPEVAKTHSVETPYGSVTFTVYGTPKPKRPAILTYHDVGLN

YKSCFQPLFQFADMQEIIQNFVRVHVDAPGMEEGAPVFPLGYQYPSLDQLADMIPCILQY

LNFSTIIGIGVGAGAYVLSRYALTHPDTVEGLVLINIDPNAKGWMDWAAHKLTGLTSSIS

EMILGHLFSQEELSGNSELIQKYRNIIAHAPNLDNIELYWNSYNNRRDLNFVRGGDTTLK

CPVMLVVGDQAPHEDAVVECNSKLDPTQTSFLKMADSGGQPQLTQPGKLTEAFKYFLQGM

GYMASSCMTRLSRSRTASLTSAASIDGNRSRSRTLSQSSESGTLSSGPPGHTMEVSC

>sp|A4IF69|NHLC2_BOVIN NHL repeat-containing protein 2 OS=Bos taurus OX=9913 GN=NHLRC2 PE=2 SV=1

MAAPGARSCNLSGLLPAQTSLEYALLDAVTQEEKDGLVYQYLQKVDGWEQDLLVPEFPEG

LEWLNTEEPISVYKDLCGKVVILDFFTYCCINCIHLLPDLHALEHTYSDKDGLLIVGVHS

AKFPNEKVLDNIRSAVLRYNITHPVVNDADASLWQELEVSCWPTLIILGPRGNMLFSLIG

EGHKEKLFLYTSIALKYYKDRGQIRANKIGIKLYKDSLPPSPLLFPGKITVDHVSNRLVI

ADTGHHRILVVWKNGQIQYSIGGPNPGRKDGIFSESSFNSPQGVAIMNNIIYVADTENHL

IRKIDLEAEMVSTVAGIGIQGTDKEGGAKGDEQPISSPWDVVFGRSGPEVQRDNILWIAM

AGTHQIWALLLDCGRLPKKNELKKGTCLRFAGSGNEENRNNAYPHKAGFAQPSGLSLASE

GPWSCLFVADSESSTVRTVSLKDGAVKHLVGGERDPMNLFAFGDVDGVGINARLQHPLGV

TWDQKRNLLYVADSYNHKIKVVDPKTKNCTTLAGTGNASNMIGSSFTDSTFNEPGGLCIG

ENGQLLYVADTNNHQIKVLDLETKTVSVFPVFRSENAVVDGPCLAGKPKTLPKLPKSAPG

IRLAPVAASPGQTLQFKLRLDLPSGTKLTEGASSCWFLSAEGNEWLLQGQIPSGEIESIS

NQPTISLQIPGDCLSLEAILSISVFLYYCSSDSSACMMKGILFSQPLQITDTQQDCIPPV

ELKYIF

>sp|Q8MJ50|OSTF1_BOVIN Osteoclast-stimulating factor 1 OS=Bos taurus OX=9913 GN=OSTF1 PE=2 SV=1

MSKPPPKPVKPGQVKVFRALYTFEPRTPDELYFEEGDIIYITDMSDTNWWKGTCKGRTGL

IPSNYVAEQAESIDNPLHEAAKRGNLSWLRECLDNRVGVNGLDKAGSTALYWACHGGHRD

IVEMLFTQPNIELNQQNKLGDTALHAAAWKGYADIVQLLLEKGARTDLRNNEKKLALDMA

TNAACASLLKKKQGTDAVRSLSNAEDYLDDEDSD

>sp|P05307|PDIA1_BOVIN Protein disulfide-isomerase OS=Bos taurus OX=9913 GN=P4HB PE=1 SV=1

MLRRALLCLALTALFRAGAGAPDEEDHVLVLHKGNFDEALAAHKYLLVEFYAPWCGHCKA

LAPEYAKAAGKLKAEGSEIRLAKVDATEESDLAQQYGVRGYPTIKFFKNGDTASPKEYTA

GREADDIVNWLKKRTGPAASTLSDGAAAEALVESSEVAVIGFFKDMESDSAKQFFLAAEV

IDDIPFGITSNSDVFSKYQLDKDGVVLFKKFDEGRNNFEGEVTKEKLLDFIKHNQLPLVI

EFTEQTAPKIFGGEIKTHILLFLPKSVSDYEGKLSNFKKAAESFKGKILFIFIDSDHTDN

QRILEFFGLKKEECPAVRLITLEEEMTKYKPESDELTAEKITEFCHRFLEGKIKPHLMSQ

ELPDDWDKQPVKVLVGKNFEEVAFDEKKNVFVEFYAPWCGHCKQLAPIWDKLGETYKDHE

NIVIAKMDSTANEVEAVKVHSFPTLKFFPASADRTVIDYNGERTLDGFKKFLESGGQDGA

GDDDDLEDLEEAEEPDLEEDDDQKAVKDEL

>sp|P43033|LIS1_BOVIN Platelet-activating factor acetylhydrolase IB subunit beta OS=Bos taurus OX=9913 GN=PAFAH1B1 PE=1 SV=2

MVLSQRQRDELNRAIADYLRSNGYEAAYSVFKKEAELDMNEELDKKYAGLLEKKWTSVIR

LQKKVMELESKLNEAKEEFTSGGPLGQKRDPKEWIPRPPEKYALSGHRSPVTRVIFHPVF

SVMVSASEDATIKVWDYETGDFERTLKGHTDSVEDISFDHSGKLLASCSADMTIKLWDFQ

GFECIRTMHGHDHNVSSVAIMPNGDHIVSASRDKTIKMWEVQTGYCVKTFTGHREWVRMV

RPNQDGTLIASCSNDQTVRVWVVATKECKAELREHEHVVECISWAPESSYSSISEATGSE

TKKSGKPGPFLLSGSRDKTIKMWDVSTGMCLMTLVGHDNWVRGVLFHSGGKFILSCADDK

TLRVWDYKNKRCMKTLNAHEHFVTSLDFHKTAPYVVTGSVDQTVKVWECR

>sp|Q29460|PA1B3_BOVIN Platelet-activating factor acetylhydrolase IB subunit alpha1 OS=Bos taurus OX=9913 GN=PAFAH1B3 PE=1 SV=1

MSGDENPASKPTPVQDVQGDGRWMSLHHRFVADSKDKEPEVVFIGDSLVQLMHQCEIWRE

LFSPLHALNFGIGGDSTQHVLWRLENGELEHIRPKIVVVWVGTNNHGHTAEQVTGGIKAI

VQLVNERQPQARVVVLGLLPRGQHPNPLREKNRRVNELVRAALAGHPRAHFLDADPGFVH

SDGTISHHDMYDYLHLSRLGYTPVCRALHSLLLRLLTQDQGQGGAPLPEPSP

>sp|Q2HJF4|PBLD_BOVIN Phenazine biosynthesis-like domain-containing protein OS=Bos taurus OX=9913 GN=PBLD PE=2 SV=1

MKLPIFIADAFTTKAFRGNPAAVCLLENKLDEDLHQKIAKEMNLSETAFIRKLHPNDNFT

QSSCFGLRWFTPQNEVPLCGHATLASAAVLFHKIKNVHSTLTFVTMSGELKARKEEDGIV

LDLPLYPAHPQKLHEVEDLIKTAIGDTLVQDVRYSPDTKKLLVRLSDTYNRSFLESLTVN

TENLLQVETTGKVKGLILTLKGEPGGQTQAFDFYSRYFAPWYGVAEDPVTGSAHTVLSSY

WSEQLGKKDLHAFQCSNRGGELTISLRSDGRVDIKGGAALVLEGTLTA

>sp|Q5E9A3|PCBP1_BOVIN Poly(rC)-binding protein 1 OS=Bos taurus OX=9913 GN=PCBP1 PE=2 SV=1

MDAGVTESGLNVTLTIRLLMHGKEVGSIIGKKGESVKRIREESGARINISEGNCPERIIT

LTGPTNAIFKAFAMIIDKLEEDINSSMTNSTAASRPPVTLRLVVPATQCGSLIGKGGCKI

KEIRESTGAQVQVAGDMLPNSTERAITIAGVPQSVTECVKQICLVMLETLSQSPQGRVMT

IPYQPMPASSPVICAGGQDRCSDAAGYPHATHDLEGPPLDAYSIQGQHTISPLDLAKLNQ

VARQQSHFAMMHGGTGFAGIDSSSPEVKGYWASLDASTQTTHELTIPNNLIGCIIGRQGA

NINEIRQMSGAQIKIANPVEGSSGRQVTITGSAASISLAQYLINARLSSEKGMGCS

>sp|P15246|PIMT_BOVIN Protein-L-isoaspartate(D-aspartate) O-methyltransferase OS=Bos taurus OX=9913 GN=PCMT1 PE=1 SV=2

MAWKSGGASHSELIHNLRKNGIIKTDKVFEVMLATDRSHYAKCNPYMDSPQSIGFQATIS

APHMHAYALELLFDQLNEGAKALDVGSGSGILTACFARMVGPSGKVIGIDHIKELVDDSI

NNVRKDDPMLLSSGRVQLVVGDGRMGYAAEAPYDAIHVGAAAPVVPQALIDQLKPGGRLI

LPVGPAGGNQMLEQYDKLQDGSVKMKPLMGVIYVPLTDKEKQWSRWK

>sp|Q3SZ62|PGAM1_BOVIN Phosphoglycerate mutase 1 OS=Bos taurus OX=9913 GN=PGAM1 PE=2 SV=3

MAAYKLVLIRHGESTWNLENRFSGWYDADLSPAGHEEAKRGGQALRDAGYEFDICFTSVQ

KRAIRTLWTVLDAIDQMWLPVVRTWRLNERHYGGLTGLNKAETAAKHGEAQVKIWRRSYD

VPPPPMEPDHPFYSNISKDRRYADLTEDQLPSCESLKDTIARALPFWNEEIVPQIKEGKR

VLIAAHGNSLRGIVKHLEGLSEEAIMELNLPTGIPMVYELDKNLKPIKPMQFLGDEETVR

KAMEAVAAQGKAKK

>sp|Q32PA4|PHP14_BOVIN 14 kDa phosphohistidine phosphatase OS=Bos taurus OX=9913 GN=PHPT1 PE=2 SV=1

MAAAGLAQIPDVDIDSDGVFKYVLIRVYAAPPSGDPAVETKEIVRGYKWAEYHADIYDKV

SGEIQKKGYDCECLGGGRISHQSQDRKIHVYGYSMGYGRAQHSVSTEKIKAKYPDYEVTW

ADDGY

>sp|A6H742|PLSI_BOVIN Plastin-1 OS=Bos taurus OX=9913 GN=PLS1 PE=2 SV=1

MENSTTTISREELEELQEAFNKIDIDNSGYVSDYELQDLFKEASLPLPGYKVREIVEKIL

AVADNNKDSRISFEEFVSLMQELKSKDISKTFRKIINKREGITAIGGTSSISSEGTQHSY

SEEEKVAFVNWINKALENDPDCKHLIPMNPNDDSLFKSLADGILLCKMINLSEPDTIDER

AINKKKLTPFTISENLNLALNSASAIGCTVVNIGAQDLTEGKPHLVLGLLWQIIKVGLFA

DIEISRNEALIALLKEGEDLEELMRLSPEELLLQWVNYHLTNAGWPTISNFSHDIKDSRA

YFHLLNQIAPKGDRDDGPAIAIDLTGFSEKNDLKRAEFMLQEADKLGCRQFVTPADVVSG

NPKLNLAFVANLFNTYPGLHKPDNNDIDVNLLEGESKEERTFRNWMNSLGVNPYINHLYS

DLADALVIFQLYEMIRVPVDWSHVNKPPYPALGGNMKKIENCNYAVELGKNKAKFSLVGI

AGQDLNEGNSTLTLALVWQLMRRYTLNVLSDLGEGEKVNDAIIIEWVNQTLKSANKNTFI

SSFKDKSISTSLPVLDLIDAIAPNAVRQEMIKREDLSDEDKLNNAKYAISVARKIGARIY

ALPDDLVEVKPKMVMTVFACLMGKGLNKIK

>sp|Q2KIU2|PMVK_BOVIN Phosphomevalonate kinase OS=Bos taurus OX=9913 GN=PMVK PE=2 SV=3

MAPLGGVPGLVLLFSGKRKSGKDFVTEALQSRLGADVCAILRLSGPLKEQYAQEHGLDFQ

RLMDASTYKEAYRSDMIRWGEEKRQADPGFFCRKIVEGVCQPVWLVSDTRRVSDIQWFQE

AYGAVTQTVRVVATEESRQQRGWVFTPGVDDAESECGLDNFRTFDWVIENHGDEQHLEEQ

LEHLIEFIRSRL

>sp|P26882|PPID_BOVIN Peptidyl-prolyl cis-trans isomerase D OS=Bos taurus OX=9913 GN=PPID PE=1 SV=6

MSHPSPQAKPSNPSNPRVFFDVDIGGERVGRIVLELFADIVPKTAENFRALCTGEKGIGP

TTGKPLHFKGCPFHRIIKKFMIQGGDFSNQNGTGGESIYGEKFEDENFHYKHDKEGLLSM

ANAGSNTNGSQFFITTVPTPHLDGKHVVFGQVIKGMGVAKILENVEVKGEKPAKLCVIAE

CGELKEGDDWGIFPKDGSGDSHPDFPEDADVDLKDVDKILLISEDLKNIGNTFFKSQNWE

MAIKKYTKVLRYVEGSRAAAEDADGAKLQPVALSCVLNIGACKLKMSDWQGAVDSCLEAL

EIDPSNTKALYRRAQGWQGLKEYDQALADLKKAQEIAPEDKAIQAELLKVKQKIKAQKDK

EKAAYAKMFA

>sp|Q5E947|PRDX1_BOVIN Peroxiredoxin-1 OS=Bos taurus OX=9913 GN=PRDX1 PE=2 SV=1

MSSGNAKIGHRAPQFKATAVMPDGQFKDISLADYKGKYVVFFFYPLDFTFVCPTEIIAFS

DRAEEFKKLNCQVIGASVDSHFCHLAWINTPKKQGGLGPMNIPLISDPKRTIAQDYGVLK

ADEGISFRGLFIIDDKGILRQITINDLPVGRSVDETLRLVQAFQFTDKHGEVCPAGWKPG

SDTIKPDVQKSKEYFSKQK

>sp|Q9BGI2|PRDX4_BOVIN Peroxiredoxin-4 OS=Bos taurus OX=9913 GN=PRDX4 PE=2 SV=1

MEAPPPPPPLPATTLAPGRSRKLLLLPLLLFLLRAEAVRGFEAEERPRTREEECHFYAGG

QVYPGEVSRVSVAEHSLHLSKAKISKPAPYWEGTAVINGEFKELKLTDYRGKYLVFFFYP

LDFTFVCPTEIIAFGDRIDEFRSINTEVVACSVDSQFTHLAWINTPRRQGGLGSINIPLL

ADLNHQISKDYGVYLEDSGHTLRGLFIIDDKGILRQITLNDLPVGRSVDETLRLVQAFQY

TDKHGEVCPAGWKPGSETIIPDPAGKLKYFDKLN

>sp|P00517|KAPCA_BOVIN cAMP-dependent protein kinase catalytic subunit alpha OS=Bos taurus OX=9913 GN=PRKACA PE=1 SV=3

MGNAAAAKKGSEQESVKEFLAKAKEDFLKKWENPAQNTAHLDQFERIKTLGTGSFGRVML

VKHMETGNHYAMKILDKQKVVKLKQIEHTLNEKRILQAVNFPFLVKLEFSFKDNSNLYMV

MEYVPGGEMFSHLRRIGRFSEPHARFYAAQIVLTFEYLHSLDLIYRDLKPENLLIDQQGY

IQVTDFGFAKRVKGRTWTLCGTPEYLAPEIILSKGYNKAVDWWALGVLIYEMAAGYPPFF

ADQPIQIYEKIVSGKVRFPSHFSSDLKDLLRNLLQVDLTKRFGNLKNGVNDIKNHKWFAT

TDWIAIYQRKVEAPFIPKFKGPGDTSNFDDYEEEEIRVSINEKCGKEFSEF

>sp|P05131|KAPCB_BOVIN cAMP-dependent protein kinase catalytic subunit beta OS=Bos taurus OX=9913 GN=PRKACB PE=1 SV=2

MGNAATAKKGSEVESVKEFLAKAKEDFLKKWENPAPNNAGLEDFERKKTLGTGSFGRVML

VKHKATEQYYAMKILDKQKVVKLKQIEHTLNEKRILQAVNFPFLVRLEYAFKDNSNLYMV

MEYVPGGEMFSHLRRIGRFSEPHARFYAAQIVLTFEYLHSLDLIYRDLKPENLLIDHQGY

IQVTDFGFAKRVKGRTWTLCGTPEYLAPEIILSKGYNKAVDWWALGVLIYEMAAGYPPFF

ADQPIQIYEKIVSGKVRFPSHFSSDLKDLLRNLLQVDLTKRFGNLKNGVSDIKTHKWFAT

TDWIAIYQRKVEAPFIPKFRGSGDTSNFDDYEEEDIRVSITEKCGKEFCEF

>sp|P07224|PROS_BOVIN Vitamin K-dependent protein S OS=Bos taurus OX=9913 GN=PROS1 PE=1 SV=1

MRVLGGRTGTLLACLALVLPVLEANFLSRQHASQVLIRRRRANTLLEETKKGNLERECIE

ELCNKEEAREIFENNPETEYFYPKYLGCLGSFRAGLFTAARLSTNAYPDLRSCVNAISDQ

CNPLPCNEDGFMTCKDGQATFTCICKSGWQGEKCESDINECKDPVNINGGCSQICENTPG

SYHCSCKNGFVMLSNKKDCKDVDECVLKPSICGTAVCKNIPGDFECECAEGYKYNPVSKS

CDDVDECAENLCAQLCVNYPGGYSCYCDGKKGFKLAQDQKSCEAVPVCLPLDLDKNYELL

YLAEQFVGVVLYLKFRLPETTRFSAEFDFRTYDSEGVILYAESSDHSAWFLIALREGKIE

IQFKNEKTTKMTTGGKVINDGLWHMVSVEELEQSISVKIAKEAVMNINKPGSLFKPTNGF

LETKVYFAGVPRKMENALIRPINPRLDGCIRGWNLMNQGTSGVKEIIQEKQNKHCLVNVE

KGSYYPGTGVAQFSINYKNESNPEAWQINVSLNIRPSAGTGVMLALVSDNTVPFALSLVD

SATEKLQDILVSVESMVIGRIEAISLCSDQQTFLEIRVNRNNLELSTQLRKDSFHSEDFQ

RQFAILDEAMKGTVVTYLGGLPDVPFSATPVNAFYQGCMEVNINGVQVDLDEAISKHNDI

RAHSCPSVWQKTKHT

>sp|Q2KIT0|HP20_BOVIN Protein HP-20 homolog OS=Bos taurus OX=9913 PE=2 SV=1

MADLRILVSIILMTNAVLEKGGCTGPPGPPGHPGPPGIRGPPGIRGIPGLPGPPGTPGPS

VKCPCHRQSAFTVKLSGQLPSPSKPVPFTEVLYNAQRDLQEDTGVFTCRVPGNYHFLFDV

DLHHCKVTVQLMRDKSSVLEKHQVSTKEPRSLSGMLTLPLHVGEKVWLEAKVETEKPEQA

RVTIYFSGFLT

>sp|P00744|PROZ_BOVIN Vitamin K-dependent protein Z OS=Bos taurus OX=9913 GN=PROZ PE=1 SV=1

AGSYLLEELFEGHLEKECWEEICVYEEAREVFEDDETTDEFWRTYMGGSPCASQPCLNNG

SCQDSIRGYACTCAPGYEGPNCAFAESECHPLRLDGCQHFCYPGPESYTCSCARGHKLGQ

DRRSCLPHDRCACGTLGPECCQRPQGSQQNLLPFPWQVKLTNSEGKDFCGGVLIQDNFVL

TTATCSLLYANISVKTRSHFRLHVRGVHVHTRFEADTGHNDVALLDLARPVRCPDAGRPV

CTADADFADSVLLPQPGVLGGWTLRGREMVPLRLRVTHVEPAECGRALNATVTTRTSCER

GAAAGAARWVAGGAVVREHRGAWFLTGLLGAAPPEGPGPLLLIKVPRYALWLRQVTQQPS

RASPRGDRGQGRDGEPVPGDRGGRWAPTALPPGPLV

>sp|Q58CY6|PXL2B_BOVIN Prostamide/prostaglandin F synthase OS=Bos taurus OX=9913 GN=PRXL2B PE=2 SV=1

MSTVDLARVGACVLKHAVTGEAVELRNLWQEQACVVAGLRRFGCMVCRWIARDLSNLKGL

LDQHGVRLVGVGPEALGLQEFLDGGYFAGELYLDESKQFYKELGFKRYNSLSILPAALGK

PVREVAAKAKAVGIQGNLSGDLLQSGGLLVVAKGGDKVLLHFVQKSPGDYAPLESILQAL

GISAEVGPSELPQCDEEACSR

>sp|Q5E987|PSA5_BOVIN Proteasome subunit alpha type-5 OS=Bos taurus OX=9913 GN=PSMA5 PE=1 SV=1

MFLTRSEYDRGVNTFSPEGRLFQVEYAIEAIKLGSTAIGIQTSEGVCLAVEKRITSPLME

PSSIEKIVEIDAHIGCAMSGLIADAKTLIDKARVETQNHWFTYNETMTVESVTQAVSNLA

LQFGEEDADPGAMSRPFGVALLFGGVDEKGPQLFHMDPSGTFVQCDARAIGSASEGAQSS

LQEVYHKSMTLKEAIKSSLIILKQVMEEKLNATNIELATVQPGQNFHMFTKEELEEVIKD

I

>sp|Q32KL2|PSB5_BOVIN Proteasome subunit beta type-5 OS=Bos taurus OX=9913 GN=PSMB5 PE=1 SV=1

MALASVLERPLSVNRRGFFGLGGRADLLDLGPGSPSDGLSLAAPSWGVPEEPRIEILHGT

TTLAFKFRHGVIVAADSRATAGAYIASQTVKKVIEINPYLLGTMAGGAADCSFWERLLAR

QCRIYELRNKERISVAAASKLLANMVYQYKGMGLSMGTMICGWDKRGPGLYYVDSEGNRI

SGATFSVGSGSVYAYGVMDRGYSYDLEVEEAYDLARRAIYQATYRDAYSGGSVSLYHVRE

DGWIRVSSDNVADLHDKYSGSTH

>sp|Q5E9F9|PRS7_BOVIN 26S proteasome regulatory subunit 7 OS=Bos taurus OX=9913 GN=PSMC2 PE=2 SV=3

MPDYLGADQRKTKEDEKDDKPIRALDEGDIALLKTYGQSTYSRQIKQVEDDIQQLLKKIN

ELTGIKESDTGLAPPALWDLAADKQTLQSEQPLQVARCTKIINADSEDPKYIINVKQFAK

FVVDLSDQVAPTDIEEGMRVGVDRNKYQIHIPLPPKIDPTVTMMQVEEKPDVTYSDVGGC

KEQIEKLREVVETPLLHPERFVNLGIEPPKGVLLFGPPGTGKTLCARAVANRTDACFIRV

IGSELVQKYVGEGARMVRELFEMARTKKACLIFFDEIDAIGGARFDDGAGGDNEVQRTML

ELINQLDGFDPRGNIKVLMATNRPDTLDPALMRPGRLDRKIEFSLPDLEGRTHIFKIHAR

SMSVERDIRFELLARLCPNSTGAEIRSVCTEAGMFAIRARRKIATEKDFLEAVNKVIKSY

AKFSATPRYMTYN

>sp|Q2KIW6|PRS10_BOVIN 26S proteasome regulatory subunit 10B OS=Bos taurus OX=9913 GN=PSMC6 PE=2 SV=1

MADPRDKALQDYRKKLLEHKEIDGRLKELREQLKELTKQYEKSENDLKALQSVGQIVGEV

LKQLTEEKFIVKATNGPRYVVGCRRQLDKSKLKPGTRVALDMTTLTIMRYLPREVDPLVY

NMSHEDPGNVSYSEIGGLSEQIRELREVIELPLTNPELFQRVGIIPPKGCLLYGPPGTGK

TLLARAVASQLDCNFLKVVSSSIVDKYIGESARLIREMFNYARDHQPCIIFMDEIDAIGG

RRFSEGTSADREIQRTLMELLNQMDGFDTLHRVKMIMATNRPDTLDPALLRPGRLDRKIH

IDLPNEQARLDILKIHAGPITKHGEIDYEAIVKLSDGFNGADLGNVCTEAGMFAIRADHD

FVVQEDFMKAVRKVADSKKLESKLDYKPV

>sp|Q0P5A6|PSMD5_BOVIN 26S proteasome non-ATPase regulatory subunit 5 OS=Bos taurus OX=9913 GN=PSMD5 PE=2 SV=1

MAAQALALLREVSRLEAPLEELRALQSLLQSVPLSELREQAAELRLGPLFSLLNENHREQ

TTLCVSILERLLQALEPVHVARNLRVDLQRGLTHPNDSVKILTLSQVGRIVENSDAVTEI

LNNAELLKQIVYCIGGENLSVAKTAIKSLSRISLTQAGLEALFESNLLDDLKSVMKTNDI

VRYRVYELIVEISSVSPESLNCCTTSGLVTQLLRELTGEDVLVRATCIEMVTSLACTHHG

RQYLAQEGVIDQISNIIVGADADPFSSFYLPGFVKFFGNLAIMDSPQQICERYPIFMEKV

FEMTESQDPTMIGVAVDTIGILGSNVEGKQVLQKTGTRFERLLMKIGYQAKNASTELKIR

CLDALSSLFYLPPEQTDDLLRMTESWFSSLSRDPLELFRGISNQPFPELHCAALKVFTAI

ANQPWAQKLMFNSPGFVEYVMDRSVEHDKASKDAKYELVKALANSKTIAEIFGNPNYLRL

RTYLSEGPYYVKPISTTAVEGAE

>sp|Q3ZBF7|TEBP_BOVIN Prostaglandin E synthase 3 OS=Bos taurus OX=9913 GN=PTGES3 PE=1 SV=1

MQPASAKWYDRRDYVFIEFCVEDSKDVNVNFEKSKLTFSCLGGSDNFKHLNEIDLFHCID

PNDSKHKRTDRSILCCLRKGESGQSWPRLTKERAKLNWLSVDFNNWKDWEDDSDEDMSNF

DRFSEMMNNMGGDEDVDLPEVDGADDDSQDSDDEKMPDLE

>sp|Q32L99|PTGR2_BOVIN Prostaglandin reductase 2 OS=Bos taurus OX=9913 GN=PTGR2 PE=2 SV=1

MIVQRVVLNSRPGKNGHPVAENFRVEEVNLPDCVNEGQVQVRTLYLSVDPYMRCRMNEDT

GSDYITPWQLSQVVDGGGVGIIEESKHTNFMKGDFVTSFYWPWQTKVILDGNILEKVDPQ

LVDGHLSYFLGAIGMPGLTSLIGVQEKGHITAGSNQTMVVSGAAGACGSLAGQIGRLLGC

SRVVGICGTPEKCLFLTSELGFDAAINYKEGNVAEQLHKLCPAGVDVYFDNVGGDISDTV

ISQMNQNSHIILCGQISQYNKDVPYPPPLPPAIEAIQKERNITRERFLVLNYKDKFEFGI

LQLSQWFKEGKLKIKETMINGLENMGAAFQSMMTGGNIGKQIVCISGDTSL

>sp|A7MBJ4|PTPRF_BOVIN Receptor-type tyrosine-protein phosphatase F OS=Bos taurus OX=9913 GN=PTPRF PE=2 SV=1

MTPEPAPGRTMVPLVPALVMLGLVAGAHGDSKPVFVKVPEDQTGLSGGVASFVCQATGEP

KPRITWMKKGKKVSSQRFEVIEFDDGAGSVLRIQPLRVQRDEAIYECTATNSLGEINTSA

KLSVLEEEQLPLGFPSIDMGPQLKVVEKARTATMLCAAGGNPDPEISWFKDFLPVDPAAS

NGRIKQLRSGALQIESSEESDQGKYECVATNSAGTRYSAPANLYVRVRRVAPRFSIPPSS

QEVMPGGSVNLTCVAVGAPMPYVKWMMGAEELTKEDEMPVGRNVLELSNVVRSANYTCVA

ISSLGMIEATAQVTVKALPKPPIDLVVTETTATSVTLTWDSGNSEPVSYYGIQYRPAGAE

GPFQEVDGVATTRYSIGGLSPFSEYAFRVLAVNSIGRGPPSEAVRARTGEQAPSSPPRRV

QARMLSASTMLVQWEPPEEPNGLVRGYRVYYTPDSRRPLSAWHKHNTDAGLLTTVGSLLP

GITYSLRVLAFTAVGDGPPSPTIQVKTQQGVPAQPADFQAEVDSDTRIQLSWLLPPQERI

VKYELVYWAAEDEGQQHKVTFDPTSSYTVEDLKPDTLYRFQLAARSELGVGVFTPTIEAR

TAQSTPSAPPQKVTCVSVGSTTVRVSWVPPPADSRNGVITQYSVAYEAVDGEDRGRHVVD

GIGREHSSWDLVGLEKWTEYRVWVRAHTDVGPGPESSPVLVRTDEDVPSGPPRKVEVEPL

NSTAVRVSWKLPVPSKQHGQIRGYQVTYVRLENGEPRGAPIIQDVMLAEAQETTISGLTP

ETTYSITVAAYTTKGDGARSKPKIVTTTGAVPGRPTMMVSTTAMNTALLQWHPPKELPGE

LLGYRLQYRRADEARPSTIDFGKDDQHFTVTGLHKGATYIFRLTAKNRAGLGEEFEKEIT

TPEDVPSGFPQNLRVIGLTTSTTELIWDPPVLAERNGRITNYTVVYRDINSQQELQNVTA

DTHLTLSGLKPDTTYDIKVRARTSKGAGPLSPSIQSRTMPVEQVFAKNFRVEAAMKTSVL

LSWEVPDSYKSAVPFRILYNGQSVEVDGHSMRKLIADLQPNTEYSFVLMNRGSSAGGLQH

LVSIRTAPDLLPHKPLPASAYIEDGRFTLTMPRVQEPALVRWFYIMVVPIDRMGGSMLAP

QWSTPEELELDELLEAIEQGGGERLRRRRQTERLKPYVAAQVDVLPETFTLGDKKNYQGF

YNRPLSPDLSYQCFVLASLKEPVDQKRYACSPYSDEIVVQVTPAQQQEEPELLWVTGPVL

AVILIVLIVIAILLFKRKRTHSPSSKDEQSIGLKDSLLAHSSDPVEMRRLNYQTPGMRDH

PPIPITDLADNIERLKANDGLKFSQEYESIDPGQQFTWENSNLEVNKPKNRYANVIAYDH

SRVILTSIDGVPGSDYINANYIDGYRKQNAYIATQGPLPETMGDFWRMVWEQRTATVVMM

TRLEEKSRVKCDQYWPARGTETYGLIQVTLLDTVELATYTVRTFALYKSGSSEKRELRQF

QFMAWPDHGVPEYPTPILAFLRRVKACNPLDAGPMVVHCSAGVGRTGCFIVIDAMLERMK

HEKTVDIYGHVTCMRAQRNYMVQTEDQYVFIHEALLEAAMCGHTEVPARNLYAHIQKLGQ

VPPGESVTAMELEFKLLANSKAHTSRFISANLPCNKFKNRLVNIMPYELTRVCLQPIRGV

EGSDYINASFLDGYRQQKAYIATQGPLAESTEDFWRMLWEHNSTIIVMLTRLREMGREKC

HQYWPAERSARYQYFVVDPMAEYNMPQYILREFKVTDARDGQSRTIRQFQFTDWPEQGVP

KTGEGFIDFIGQVHKTKEQFGQDGPITVHCSAGVGRTGVFITLSIVLERMRYEGVVDMFQ

TVKTLRTQRPAMVQTEDQYQLCYRAALEYLGSFDHYAT

>sp|Q3MHH4|SYQ_BOVIN Glutamine--tRNA ligase OS=Bos taurus OX=9913 GN=QARS1 PE=2 SV=1

MAALDSLSLFTGLGLSEQKARETLKNTVLSAQLREAATQAQQTLGSSIDKATGTLLYGLA

SRLRDPRRLSFLVSYITSRKIHTETQLSAALEYVRSHPLDPINTEDFEQECGVGVVVTPE

QIEEAVEAAINRHRAKLLVERYHFSMGLLMGEARAALKWADGKMIKHEVDMQVLHLLGPK

TETDLEKKPKVAKARPEETDQRTAKDVVENGEVVVQTLSLMEQLRGEALKFHKPGENYKT

PGYVTTPHTMDLLKQHLDITGGQVRTRFPPEPNGILHIGHAKAINFNFGYAKANNGICFL

RFDDTNPEKEEAKFFTAIYDMVAWLGYTPYKVTYASDYFDQLYAWAVELIRRDQAYVCHQ

RGEELKGHNPLPSPWRDRPIEESLLLFEAMRKGKFAEGEATLRMKLVMEDGKMDPVAYRV

KYTPHHRTGDTWCIYPTYDYTHCLCDSIEHITHSLCTKEFQARRSSYFWLCNALDVYCPV

QWEYGRLNLHYAVVSKRKILQLVAAGAVRDWDDPRLFTLTALRRRGFPPEAINNFCARVG

VTVAQTTMEPHLLEACVRDVLNDTAPRAMAVLEPLQVVITNFPATKALDIQVPNFPADET

KGFHQVPFGSTVFIERMDFKEEPEPGYKRLAWGQPVGLRHTGYVIELQHVVKGPSGCVES

LKVTCRRADAGEKPKAFIHWVSQPLTCEIRLYERLFQHKNPEDPAEVPGGFLSDLNPASL

QVVEAALVDCSVALAKPFDKFQFERLGYFSVDPDSNQGQLVFNRTVTLKEDPGKV

>sp|Q0IIG7|RAB5A_BOVIN Ras-related protein Rab-5A OS=Bos taurus OX=9913 GN=RAB5A PE=1 SV=1

MANRGATRPNGPNTGNKICQFKLVLLGESAVGKSSLVLRFVKGQFHEFQESTIGAAFLTQ

TVCLDDTTVKFEIWDTAGQERYHSLAPMYYRGAQAAIVVYDITNEESFARAKNWVKELQR

QASPNIVIALSGNKADLANKRAVDFQEAQSYADDNSLLFMETSAKTSMNVNEIFMAIAKK

LPKNEPQNPGANSTRGRGVDLTEPTQPTRSQCCSN

>sp|Q58DS9|RAB5C_BOVIN Ras-related protein Rab-5C OS=Bos taurus OX=9913 GN=RAB5C PE=2 SV=1

MAGRGGAARPNGPAAGNKICQFKLVLLGESAVGKSSLVLRFVKGQFHEYQESTIGAAFLT

QTVCLDDTTVKFEIWDTAGQERYHSLAPMYYRGAQAAIVVYDITNTDTFARAKNWVKELQ

RQASPNIVIALAGNKADLASKRAVEFQEAQAYAEDNSLLFMETSAKTAMNVNEIFMAIAK

KLPKNEPQNAAGAPGRNRGVDLQENNPASRSQCCSN

>sp|A6QR46|RAB6B_BOVIN Ras-related protein Rab-6B OS=Bos taurus OX=9913 GN=RAB6B PE=2 SV=1

MSAGGDFGNPLRKFKLVFLGEQSVGKTSLITRFMYDSFDNTYQATIGIDFLSKTMYLEDR

TVRLQLWDTAGQERFRSLIPSYIRDSTVAVVVYDITNLNSFQQTSKWIDDVRTERGSDVI

IMLVGNKTDLADKRQITIEEGEQRAKELSVMFIETSAKTGYNVKQLFRRVASALPGMENV

QEKSKEGMIDIKLDKPQEPPASEGGCSC

>sp|Q3T0F5|RAB7A_BOVIN Ras-related protein Rab-7a OS=Bos taurus OX=9913 GN=RAB7A PE=2 SV=1

MTSRKKVLLKVIILGDSGVGKTSLMNQYVNKKFSNQYKATIGADFLTKEVMVDDRLVTMQ

IWDTAGQERFQSLGVAFYRGADCCVLVFDVTAPNTFKTLDSWRDEFLIQASPRDPENFPF

VVLGNKIDLENRQVATKRAQAWCYSKNNIPYFETSAKEAINVEQAFQTIVRNALKQETEV

ELYNEFPEPIKLDKNDRTKPSAEGCSC

>sp|P63243|RACK1_BOVIN Receptor of activated protein C kinase 1 OS=Bos taurus OX=9913 GN=RACK1 PE=2 SV=3

MTEQMTLRGTLKGHNGWVTQIATTPQFPDMILSASRDKTIIMWKLTRDETNYGIPQRALR

GHSHFVSDVVISSDGQFALSGSWDGTLRLWDLTTGTTTRRFVGHTKDVLSVAFSSDNRQI

VSGSRDKTIKLWNTLGVCKYTVQDESHSEWVSCVRFSPNSSNPIIVSCGWDKLVKVWNLA

NCKLKTNHIGHTGYLNTVTVSPDGSLCASGGKDGQAMLWDLNEGKHLYTLDGGDIINALC

FSPNRYWLCAATGPSIKIWDLEGKIIVDELKQEVISTSSKAEPPQCTSLAWSADGQTLFA

GYTDNLVRVWQVTIGTR

>sp|A7YW98|SYRC_BOVIN Arginine--tRNA ligase, cytoplasmic OS=Bos taurus OX=9913 GN=RARS1 PE=2 SV=1

MDALVAHCSARLLQQEKEIKFLTAEVDRLKNYSCSEASADLEKLREENLKLKYRLNILRK

SLQAERNKPTKTMININSCLEEVFGCAIKAAYPVLENPPLIVTPSQQPKFGDYQCNSAMG

ICQMLKTKEQKVNPREIAENIVKHLPDNEYIEKVEIAGPGFINIHLRKGFVSQQLTNLLV

NGVKIPSIGENKKVIVDFSSPNIAKEMHVGHLRSTIIGESMCRLFEFAGYNVLRLNHVGD

WGTQFGMLIAHLQDKFPDYLTVSPPIGDLQAFYKESKKRFDTEEEFKKRAYQCVVLLQSK

NPDIIKAWKLICDVSRQEFNKIYEALDISLIERGESFYQDRMHDIVKEFEDRGFVQVDDG

RKIVFVPGCSVPLTIVKSDGGYTYDTSDLAAIKQRLFEEKADMIIYVVDNGQSLHFQTVF

GAAQMIGWYDPAVTRVSHAGFGVVLGEDKKKFKTRSGETVRLIDLLEEGLKRSMDKLKEK

ERDKVLTTEELKAAQTSVAYGCIKYADLSHNRLNDYIFSFDKMLDDRGNTAAYLLYAFTR

IRSIARLANIDEEMLRKAAHETEIILDHEKEWKLGRCILRFPEVLQKILDDLLLHTLCDY

IYELATTFTEFYDSCYCVEKDRQSGEVLKVNMWRMLLCEAVAAVMAKGFDILGIKPVQRM

>sp|A6N9I4|GRP2_BOVIN RAS guanyl-releasing protein 2 OS=Bos taurus OX=9913 GN=RASGRP2 PE=2 SV=1

MAGTLDLDKGCTVEELLRGCIEAFDDSGKVRDPQLVRMFLMMHPWYIPSSQLAAKLLHIY

QQSRKDNSSSLQVKTCHLVRYWISAFPAEFDLNPELAEQIKELKALLDQEGNRRHSSLID

IENVPTYKWKRQVTQRNPVEQKKRKMSLLFDHLEPLELAAHLTYLEYRSFCKILFQDYHS

FVTHGCTVDNPVLERFISLFNSVSQWVQLMILSKPTAPQRAGVITHFVHVAEELLHLQNF

NTLMAVVGGLSHSSISRLKETHSHVSPETIKLWEGLTELVTATGNYGNYRRRLAACVGFR

FPILGVHLKDLVALQLALPDWLDPARTRLNGAKMKQLFSILEELAMVTSLRPPVQANPDL

LSLLMVSLDQYQTEDELYQLSLQREPRSKSSPTSPTTCTPPPRPPVLEEWTSAAKPKLDQ

AIMVEHIEKMVESVFRNFDVDGDGHISQEEFQIIRGNFPYLSAFGDLDQNQDGCISKEEM

VSYFLRSSSMLGGRMGFVHNFHESNSLRPVACRHCKALILGIYKQGLKCRACGVNCHKQC

KDRLSVECRRRAQSMSLEGSAPSPSPTHTHHRAFSFSLPRPGRRGSRPPEIREEEVQTVE

DGVFDIHL

>sp|Q32LP2|RADI_BOVIN Radixin OS=Bos taurus OX=9913 GN=RDX PE=2 SV=1

MPKPINVRVTTMDAELEFAIQPNTTGKQLFDQVVKTVGLREVWFFGLQYVDSKGYSTWLK

LNKKVTQQDVKKENPLQFKFRAKFFPEDVSEELIQEITQRLFFLQVKEAILNDEIYCPPE

TAVLLASYAVQAKYGDYNKEIHKPGYLANDRLLPQRVLEQHKLTKEQWEERIQNWHEEHR

GMLREDSMMEYLKIAQDLEMYGVNYFEIKNKKGTELWLGVDALGLNIYEHDDKLTPKIGF

PWSEIRNISFNDKKFVIKPIDKKAPDFVFYAPRLRINKRILALCMGNHELYMRRRKPDTI

EVQQMKAQAREEKHQKQLERAQLENEKKKREIAEKEKERIEREKEELMERLRQIEEQTMK

AQKELEEQTRKALELDQERKRAKEEAERLEKERQAAEEAKSALAKQAADQMKNQEQLAAE

LAEFTAKIALLEEAKKKKEEEATEWQHKAFAAQEDLEKTKEELKTVMSAPPPPPPPPVIP

PTENEHDEHDENNAEASAELSNDGVMNHRSEEERVTETQKNERVKKQLQALSSELAQARD

ETKKTQNDVLHAENVKAGRDKYKTLRQIRQGNTKQRIDEFEAM

>sp|Q3ZBW5|RHOB_BOVIN Rho-related GTP-binding protein RhoB OS=Bos taurus OX=9913 GN=RHOB PE=2 SV=1

MAAIRKKLVVVGDGACGKTCLLIVFSKDEFPEVYVPTVFENYVADIEVDGKQVELALWDT

AGQEDYDRLRPLSYPDTDVILMCFSVDSPDSLENIPEKWVPEVKHFCPNVPIILVANKKD

LRSDEHVRTELARMKQEPVRTDDGRAMAVRIQAYDYLECSAKTKEGVREVFETATRAALQ

KRYGSQNGCINCCKVL

>sp|P61284|RL12_BOVIN 60S ribosomal protein L12 OS=Bos taurus OX=9913 GN=RPL12 PE=2 SV=1

MPPKFDPNEIKVVYLRCTGGEVGATSALAPKIGPLGLSPKKVGDDIAKATGDWKGLRITV

KLTIQNRQAQIEVVPSASALIIKALKEPPRDRKKQKNIKHSGNITFDEIVNIARQMRHRS

LARELSGTIKEILGTAQSVGCNVDGRHPHDIIDDINSGAVECPAS

>sp|Q5E973|RL18_BOVIN 60S ribosomal protein L18 OS=Bos taurus OX=9913 GN=RPL18 PE=2 SV=3

MGVDIRHNKDRKVRRKEPKSQDIYLRLLVKLYRFLARRTNSTFNQVVLKRLFMSRTNRPP

LSLSRMIRKMKLPGREGKTAVVVGTITDDVRVQEVPKLKVCALRVSSRARSRILKAGGKI

LTFDQLALDSPKGCGTVLLSGPRKGREVYRHFGKAPGTPHSHTKPYVRSKGRKFERARGR

RASRGYKN

>sp|Q3T0R1|RS18_BOVIN 40S ribosomal protein S18 OS=Bos taurus OX=9913 GN=RPS18 PE=2 SV=3

MSLVIPEKFQHILRVLNTNIDGRRKIAFAITAIKGVGRRYAHVVLRKADIDLTKRAGELT

EDEVERVITIMQNPRQYKIPDWFLNRQKDVKDGKYSQVLANGLDNKLREDLERLKKIRAH

RGLRHFWGLRVRGQHTKTTGRRGRTVGVSKKK

>sp|P79103|RS4_BOVIN 40S ribosomal protein S4 OS=Bos taurus OX=9913 GN=RPS4 PE=2 SV=3

MARGPKKHLKRVAAPKHWMLDKLTGVFAPRPSTGPHKLRECLPLIIFLRNRLKYALTGDE

VKKICMQRFIKIDGKVRTDITYPAGFMDVISIDKTGENFRLIYDTKGRFAVHRITPEEAK

YKLCKVRKIFVGTKGIPHLVTHDARTIRYPDPLIKVNDTIQIDLETGKITDFIKFDTGNL

CMVTGGANLGRIGVITNRERHPGSFDVVHVKDANGNSFATRLSNIFVIGKGNKPWISLPR

GKGIRLTIAEERDKRLAAKQSSG

>sp|Q3T0T7|SAR1B_BOVIN GTP-binding protein SAR1b OS=Bos taurus OX=9913 GN=SAR1B PE=2 SV=1

MSFIFDWIYSGFSSVLQFLGLYKKTGKLVFLGLDNAGKTTLLHMLKDDRLGQHVPTLHPT

SEELTIAGMTFTTFDLGGHVQARRVWKNYLPAINGIVFLVDCADHERLLESKEELDSLMT

DETVANVPILILGNKIDRPEAISEERLREMFGLYGQTTGKGNVSLKELNARPLEVFMCSV

LKRQGYGEGFRWMAQYID

>sp|P49907|SEPP1_BOVIN Selenoprotein P OS=Bos taurus OX=9913 GN=SELENOP PE=2 SV=2

MWRGLGLALALCLLLTGGTESQGQSSYCKQPPPWSIKDQDPMLNSYGSVTVVALLQASUY

LCILQASRLEDLRVKLEKEGYSNISYVVVNHQGISSRLKYVHLKNKVSEHIPVYQQEENQ

PDVWTLLNGNKDDFLIYDRCGRLVYHLGLPYSFLTFTYVEDSIKTVYCEDKCGNCSLKAL

EDEDVCKNVFLATKEKTAEASQRHHHPHPHSHPHPHPHPHPHPHPHPHHGHQLHENAHLS

ESPKPDTPDTPENPPPSGLHHHHHRHKGPQRQGHSDNCDTPVGSESLQPSLPQKKLURKR

CINQLLUQFPKDSESALSSCCCHCRHLVFEKTGSAITUQCTEKLPSLCSUQGLLAEENVI

ESUQURLPPAAUQAAGQQLNPTEASTKUSUKNKAKMUKUPSN

>sp|Q2NKY7|SEPT2_BOVIN Septin-2 OS=Bos taurus OX=9913 GN=SEPTIN2 PE=2 SV=1

MSKQQQTQFINPETPGYVGFANLPNQVHRKSVKKGFEFTLMVVGESGLGKSTLINSLFLT

DLYPERVIPGAAEKIERTVQIEASTVEIEERGVKLRLTVVDTPGYGDAINCRDCFKTIIC

YIDEQFERYLHDESGLNRRHIIDNRVHCCFYFISPFGHGLKPLDVAFMKAIHNKVNIVPV

IAKADTLTLKERERLKKRILDEIEEHNIKIYHLPDAESDEDEDFKEQTRLLKASIPFSVV

GSNQLIEAKGKKVRGRLYPWGVVEVENPEHNDFLKLRTMLITHMQDLQEVTQDLHYENFR

SERLKRGGRKVENEDMNKDQILLEKEAELRRMQEMIARMQAQMQLQLQGGDGDSGVHGQH

V

>sp|Q6Q137|SEPT7_BOVIN Septin-7 OS=Bos taurus OX=9913 GN=SEPTIN7 PE=2 SV=2

MSVSARSAAAEERSVNSSTMVAQQKNLEGYVGFANLPNQVYRKSVKRGFEFTLMVVGESG

LGKSTLINSLFLTDLYSPEYPGPSHRIKKTVQVEQSKVLIKEGGVQLLLTIVDTPGFGDA

VDNSNCWQPVIDYIDSKFEDYLNAESRVNRRQMPDNRVQCCLYFIAPSGHGLKPLDIEFM

KRLHEKVNIIPLIAKADTLTPEECQQFKKQIMKEIQEHKIKIYEFPETDDEEENKLVKKI

KDRLPLAVVGSNTIIEVNGKRVRGRQYPWGVAEVENGEHCDFTILRNMLIRTHMQDLKDV

TNNVHYENYRSRKLAAVTYNGVDNNKNKGQLTKSPLAQMEEERREHVAKMKKMEMEMEQV

FEMKVKEKVQKLKDSEAELQRRHEQMKKNLEAQHKELEEKRRQFEDEKANWEAQQRILEQ

QNSSRTLEKNKKKGKIF

>sp|Q3ZEJ6|SPA33_BOVIN Serpin A3-3 OS=Bos taurus OX=9913 GN=SERPINA3-3 PE=1 SV=2

MRAERLSPLLALGLLVAGIRSVHCLPENVVVKDRHRRVDGHTLASSNTDFAFSLYKQLAL

KNPNKNVMFSPLSVSMALAFLSLGARGPTLTEILEGLKFNLTEIQETQIHQGFQHLLQAL

NRPRNQLQLSVGNAMFVQEELKLLDKFIEDARVLYSSEAFPTNFRDPEAAKSLINDYVKN

KTQGKIEELFKDLSPRTELVLVNYVYFKAQWKTRFDPKHTEQAEFHVSDNKTVEVPMMTL

DLETPYFRDEELGCTLVELTYTSNDSALFILPDKGKMQDLEAKLTPEMLTRWRNSLQPRR

IHELYLPKFSIKSNYELNDTLSQMGIKKIFTDADLSGITGTADLVVSQVVHGAALDVDEE

GTEGAAATGIGIERTFLRIIVRVNRPFLIAVVLKDTQSIIFLGKVTNPSEA

>sp|A2I7N1|SPA35_BOVIN Serpin A3-5 OS=Bos taurus OX=9913 GN=SERPINA3-5 PE=3 SV=1

MRAERTSFLLALGLLMAGIRSVHCLPENVVVKDQRRRVDSHTLASSNTDFAFSLYKQLAL

KNPNKNVMFSPLSVSMALAFLSLGARGPTLTEILEGLKFNLTEIQETQIHQGFQHLLQAL

NRPSNQLQLSVGNAMFVQEELKLLDKFIEDARVLYSSEAFPTNFRDSEAARSLINDYVKN

KTQGKIEELFKYLSPRTVLVLVNYIYFKAQWKTRFDPKHTEQAEFHVSKNKTVEVPMMTL

DLETPYFRDKELGCMLVELTYSSNDSALFILPDEGKMQDLEAKLTPETLTRWRNSLQPRR

IHELYLPKFSIKSNYELNDTLSQMGIKKIFTDADLSGITGTADLVVSQVVHGAALDVDEE

GTEGAAATGIGIERTFLRIIVRVNRPFLIAVVLKDTQSIIFLGKVTNPSEA

>sp|A6QPQ2|SPA38_BOVIN Serpin A3-8 OS=Bos taurus OX=9913 GN=SERPINA3-8 PE=2 SV=1

MRAERMSPLLALGLLVSGLCSRVHCLPENVTPEERHKGTSVDGHSLASSNTDFAFSLYKQ

LALKNPNKNVIFSPLSISIALAFLSLGARGPTVTEILEGLKFNLTETLEREIHQGFQHLL

QMLSRPSNELQLSVGNTMFVQEQLKLLDKFREDALALYTSEAFSTNFKDPETAKSLINDY

VKNKTRGKIVDLFKDLDPLTKVILVNYIYFKAQWRTPFDPKQTYKSQFHVSKNKTVEVPM

MSIGDLVTPYFRDEELDCTLVELTYTSNDSALFILPDEGKMQDLEAKLIPEMLTRWRESL

YPRGIHELNLPRFSIATDYKLKDILSQLEIKKVFTQEADLSGITDDHELEVSQVVHKAVL

DVGEEGTEGAAATGVKVGITSINNHIPLSFNRPFLIAIVLKDTQSIIFLGKVTNPSQA

>sp|Q32LM2|SGTA_BOVIN Small glutamine-rich tetratricopeptide repeat-containing protein alpha OS=Bos taurus OX=9913 GN=SGTA PE=2 SV=1

MDNKKRLAYAIIRFLHDQLRHGELSSDAQESLEVAIQCLETAFGVTVEDSDLALPQTLPE

IFEAAAAGKELPPDLRSPQETPPSEEDSAEAERLKTEGNEQMKVENFEAAVHFYGKAIEL

NPANAVYFCNRAAAYSKLGNYAGAVQDCERAICIDPSYSKAYGRMGLALSSLNKHTEAVA

YYRKALELDPDNETYKSNLKVAELRLREAPSPTGGVGSFDIAGLLNNPSFMSMASNLMNN

PQVQQLMSGMISGGHNPLGTPGTSPSQNDLASLIQAGQQFAQQMQQQNPELIEQLRSQIR

SRTPSASNDDQQE

>sp|P02722|ADT1_BOVIN ADP/ATP translocase 1 OS=Bos taurus OX=9913 GN=SLC25A4 PE=1 SV=3

MSDQALSFLKDFLAGGVAAAISKTAVAPIERVKLLLQVQHASKQISAEKQYKGIIDCVVR

IPKEQGFLSFWRGNLANVIRYFPTQALNFAFKDKYKQIFLGGVDRHKQFWRYFAGNLASG

GAAGATSLCFVYPLDFARTRLAADVGKGAAQREFTGLGNCITKIFKSDGLRGLYQGFNVS

VQGIIIYRAAYFGVYDTAKGMLPDPKNVHIIVSWMIAQTVTAVAGLVSYPFDTVRRRMMM

QSGRKGADIMYTGTVDCWRKIAKDEGPKAFFKGAWSNVLRGMGGAFVLVLYDEIKKFV

>sp|Q3SZK8|NHRF1_BOVIN Na(+)/H(+) exchange regulatory cofactor NHE-RF1 OS=Bos taurus OX=9913 GN=SLC9A3R1 PE=2 SV=1

MSADAGAGAPLPRLCCLEKGPNGYGFHLHGEKGKVGQYIRLVEPGSPAEKSGLLAGDRLV

EVNGENVEKETHQQVVNRIRAALNSVRLLVVDPETDERLQKLGVQVREEMLRAQEGPGQA

EPPAAAAEERGAGGENEPPAAAPEPREAEQSPQERRELRPRLCAMKKGPNGYGFNLHSDK

SKPGQFIRAVDPDSPAEASGLRAQDRIVEVNGVCVEGKPHGEVVSAIKAGGDEAKLLVVD

RETDEFFKKCKVIPSQEHLQGPLPEPITNGEIEKENSPEALAETASESPMPPLARTASSD

TSEELNSQDSPKKQDSTAPSSTSSSSSDPVLDFSISLAVAKERAHQKRVSKRAPQMDWSK

KNELFSNL

>sp|Q2TBW7|SNX2_BOVIN Sorting nexin-2 OS=Bos taurus OX=9913 GN=SNX2 PE=2 SV=1

MAAEREPPPLGDGKPTDFEELEDGEDLFTSTVSTLESSPSSPDPASFLAEDISTNSNGPK

PAEVALDDDREDLFAEATEEVSLDSPEREPILSSETSPAVTPVTPTTLIAPRIESKSMSA

PVIFDRSRDEIEEEANGDVFDIEIGVSDPEKVGDGMNAYMAYRVTTKTSLSMFSKSEFSV

KRRFSDFLGLHSKLASKYLHVGYIVPPAPEKSIVGMTKVKVGKEDSSSTEFVEKRRAALE

RYLQRTVKHPTLLQDPDLRQFLESSELPRAVNTQALSGAGILRMVNKAADAVNKMTIKMN

ESDAWFEEKQQQFENQDQQLRKLHASVEALVCHRKELSANTAAFAKSAAMLGNSEDHTAL

SRALSQLAEVEEKIDQLHQEQAFADFYMFSELLSDYIRLIAAVKGVFDHRVKCWQKWEDA

QITLLKKRETEAKMMVANKPDKIQQAKNEIREWEAKVQQGERDFEQISKTIRKEVGRFEK

ERVKDFKTVIIKYLESLVQTQQQLIKYWEAFLPEAKAIA

>sp|Q3ZBM5|SNX5_BOVIN Sorting nexin-5 OS=Bos taurus OX=9913 GN=SNX5 PE=2 SV=1

MAAVPEVLQQQEEDRSKLRSVSVDLNVDPSLQIDIPDALSERDKVKFTVHTKTTLPTFQS

PEFSVTRQHEDFVWLHDTLIETTDYAGLIIPPAPTKPDFDGPREKMQKLGEGEGSMTKEE

FAKMKQELEAEYLAVFKKTVSSHEVFLQRLSSHPVLSKDRNFHVFLEYDQDLSVRRKNTK

EMFGGFFKSVVKSADEVLFSGVKEVDDFFEQEKTFLINYYNRIKDSCAKADRMTRSHKNV

ADDYIHTAACLHSLALEEPTVIKKYLLKVAELFEKLRKVESRVSSDEDLKLTELLRYYML

NIEAAKDLLYRRTKALIDYENSNKALDKARLKSRDVKLAEAHQQECCQKFEQLSESAKDE

LINFKRKRVAAFRKNLIEMSELEIKHARNNVSLLQSCIDLFKNN

>sp|Q27967|SPP24_BOVIN Secreted phosphoprotein 24 OS=Bos taurus OX=9913 GN=SPP2 PE=1 SV=2

MEKMAMKMLVIFVLGMNHWTCTGFPVYDYDPASLKEALSASVAKVNSQSLSPYLFRAFRS

SVKRVNALDEDSLTMDLEFRIQETTCRRESEADPATCDFQRGYHVPVAVCRSTVRMSAEQ

VQNVWVRCHWSSSSGSSSSEEMFFGDILGSSTSRNSYLLGLTPDRSRGEPLYEPSREMRR

NFPLGNRRYSNPWPRARVNPGFE

>sp|Q2TBL6|TALDO_BOVIN Transaldolase OS=Bos taurus OX=9913 GN=TALDO1 PE=2 SV=1

MSGSPVKRQRMENALDQLKQFTTVVADTGDFHAIDEYKPQDATTNPSLILAAAQMPTYQE

LVEEAIAYGRKLGGSQEEQITNAIDKLFVLFGAEILKKIPGRVSTEVDARLSFDKDAMVA

RARRLIELYKEAGISKERILIKLSSTWEGIQAGKELEEHHGIRCNMTLLFSFAQAVACAE

AGVTLISPFVGRILDWHVANTDKKSYETQEDPGVKSVTKIYNYYKKFGYKTIVMGASFRN

TGEIKALAGCDFLTISPQLLGELLKDHSKLTPVLSAKAAQASDLEKIQLDEKAFRWLHNE

DRMAVEKLSDGIRRFAADAVKLERMLRERMFSAENGK

>sp|Q3ZBV8|SYTC_BOVIN Threonine--tRNA ligase 1, cytoplasmic OS=Bos taurus OX=9913 GN=TARS1 PE=2 SV=1

MSEEQASSPSAKMGDEEKPVGAGEEKQKEGSKKKNKEGSGDGGRAELNPWPEYINTRLEM

YNKLKAEHDSILAEKAEKDSKPIKVTLPDGKQVDAESWKTTPYQIACGISQGLADNTVIA

KVNKAVWDLDRPLEEDCTLELLKFEDEEAQAVYWHSSAHIMGEAMERVYGGCLCYGPPIE

NGFYYDMYLEEGGVSSNDFSSLETLCKKIIKEKQAFERLEVKKETLLEMFKYNKFKCRIL

NEKVNTPTTTVYRCGPLIDLCRGPHVRHTGKIKTLKIHKNSSTYWEGKSDMETLQRIYGI

SFPDPKMLKEWEKFQEEAKNRDHRKIGRDQELYFFHELSPGSCFFLPKGAYIYNTLIEFI

RSEYRKRGFQEVVTPNIYNSRLWVTSGHWEHYSENMFSFEVEKELFALKPMNCPGHCLMF

DHRPRSWRELPLRVADFGVLHRNELSGALTGLTRVRRFQQDDAHIFCAMEQIEDEIKGCL

DFLRTVYSIFGFSFKLNLSTRPEKFLGDIEVWNQAEKQLENSLNDFGEKWELNPGDGAFY

GPKIDIQIKDAIGRYHQCATIQLDFQLPIRFNLTFVSHDGDDKKKPVIIHRAILGSVERM

IAILTENYGGKWPFWLSPRQVMVVPVGPTCDEYAQKVRQQFHNAKFMVDIDLDPGCTLNK

KIRNAQLAQYNFILVVGEKEKTSGTVNIRTRDNKVHGERTISETIERLQQLKHSRSKQAE

EEF

>sp|Q32L40|TCPA_BOVIN T-complex protein 1 subunit alpha OS=Bos taurus OX=9913 GN=TCP1 PE=1 SV=1

MEGPLSVFGDRSTGEAIRSQNVMAAASIANIVKSSLGPVGLDKMLVDDIGDVTITNDGAT

ILKLLEVEHPAAKVLCELADLQDKEVGDGTTSVVIIAAELLKNADELVKQKIHPTSVISG

YRLACKEAVRYISENLIINTDELGRDCLINAAKTSMSSKVIGINGDFFANLVVDAVLAIK

YTDIRGQPRYPVNSINVLKAHGRSQMESMLINGYALNCVVGSQGMPKRIVNAKIACLDFS

LQKTKMKLGVQVVITDPEKLDQIRQRESDITKERIQKILATGANVILTTGGIDDMCLKYF

VEAGAMAVRRVLKRDLKRIAKASGATVLSTLANLEGEETFEASMLGQAEEVVQERICDDE

LILIKNTKARTSASVILRGANDFMCDEMERSLHDALCVVKRVLESKSVVPGGGAVEAALS

IYLENYATSMGSREQLAIAEFARSLPVIPNTLAVNAAQDSTDLVAKLRAFHNEAQVNPER

KNLKWIGLDLVNGKPRDNKQAGVFEPTIVKVKSLKFATEAAITILRIDDLIKLHPESKDD

KHGGYEDAVHSGALDA

>sp|Q0V8B6|TPP1_BOVIN Tripeptidyl-peptidase 1 OS=Bos taurus OX=9913 GN=TPP1 PE=2 SV=1

MGPRSGLLGLFALFVAGKCSYSPEPDQQRRLPPGWVSLGRADPEEELSLTFALRQQNVKR

LSELVQAVSDPGSPRYGKYLTLEDVAELVRPSPLTLHTVQKWLLAAGARNCHSVTTQDFL

TCWLSVRQAELLLSGAEFHHYVGGPAETHAVRSLHPYRLPKALAPHVDFVGGLHRFPPTS

TLRQHPEPQVPGTVGLHLGVTPSVIRKRYNLTAQDVGSGTTNNSQACAQFLEQYFHDSDL

AEFMRLFGGDFAHQASVARVVGQQGRGRAGIEASLDVEYLMSAGANISTWVYSSPGRHES

QEPFLQWLLLLSNESALPYVHTVSYGDDEDSLSSTYIQRVNTELMKAAARGLTLLFASGD

SGAGCWSVSGRHQFRPSFPASSPYVTTVGGTSFQNPFRVTDEVVDYISGGGFSNVFPRPS

YQEEAVTRYLSSSPHLPPSSYFNASGRAYPDVAALSDGYWVVSNHVPIPWVSGTSASTPV

FGGLLSLINEHRILRGLPPLGFLNPRLYQKHGAGLFDVTRGCHESCLNEEVEGQGFCSGP

GWDPVTGWGTPNFPALLKTLMNP

>sp|Q2HJB8|TBA8_BOVIN Tubulin alpha-8 chain OS=Bos taurus OX=9913 GN=TUBA8 PE=2 SV=1

MRECISVHVGQAGVQIGNACWELFCLEHGIQADGTFGAQASKIHDDDSFTTFFSETGNGK

HVPRAVMVDLEPTVVDEVRAGTYRHLFHPEQLITGKEDAANNYARGHYTVGKESIDLVLD

RIRKLTDACSGLQGFLIFHSFGGGTGSGFTSLLMERLSLDYGKKSKLEFAIYPAPQVSTA

VVEPYNSILTTHTTLEHSDCAFMVDNEAIYDICRRNLDIERPTYTNLNRLISQIVSSITA

SLRFDGALNVDLTEFQTNLVPYPRIHFPLVTYAPIISAEKAYHEQLSVAEITSSCFEPNS

QMVKCDPRHGKYMACCMLYRGDVVPKDVNVAIAAIKTKRTIQFVDWCPTGFKVGINYQPP

TVVPGGDLAKVQRAVCMLSNTTAIAEAWARLDHKFDLMYAKRAFVHWYVGEGMEEGEFSE

AREDLAALEKDYEEVGTDSFEEENEGEEF

>sp|Q6B856|TBB2B_BOVIN Tubulin beta-2B chain OS=Bos taurus OX=9913 GN=TUBB2B PE=1 SV=2

MREIVHIQAGQCGNQIGAKFWEVISDEHGIDPTGSYHGDSDLQLERINVYYNEATGNKYV

PRAILVDLEPGTMDSVRSGPFGQIFRPDNFVFGQSGAGNNWAKGHYTEGAELVDSVLDVV

RKESESCDCLQGFQLTHSLGGGTGSGMGTLLISKIREEYPDRIMNTFSVMPSPKVSDTVV

EPYNATLSVHQLVENTDETYCIDNEALYDICFRTLKLTTPTYGDLNHLVSATMSGVTTCL

RFPGQLNADLRKLAVNMVPFPRLHFFMPGFAPLTSRGSQQYRALTVPELTQQMFDSKNMM

AACDPRHGRYLTVAAIFRGRMSMKEVDEQMLNVQNKNSSYFVEWIPNNVKTAVCDIPPRG

LKMSATFIGNSTAIQELFKRISEQFTAMFRRKAFLHWYTGEGMDEMEFTEAESNMNDLVS

EYQQYQDATADEQGEFEEEEGEDEA

>sp|Q0IIF7|UBP14_BOVIN Ubiquitin carboxyl-terminal hydrolase 14 OS=Bos taurus OX=9913 GN=USP14 PE=2 SV=3

MPLYSVTVKWGKEKFEGVELNTDEPPMVFKAQLFALTGVQPARQKVMVKGGTLKDDDWGN

IKMKNGMTVLMMGSADALPEEPSAKTVFVEDMTEEQLASAMELPCGLTNLGNTCYMNATV

QCIRSVPELKDALKRYAGALRASGEMASAQYITAALRDLFDSMDKTSSSIPPIILLQFLH

MAFPQFAEKGEQGQYLQQDANECWVQMMRVLQQKLEAIEDDTVKETDSSSASAVTPSKKK

SLIDQFFGVEFETTMKCTESEEEEVTKGKESQLQLSCFINQEVKYLFTGLKLRLQEEITK

QSPTLQRNALYIKSSKISRLPAYLTIQMVRFFYKEKESVNAKVLKDVKFPLMLDVYELCT

PELQEKMVSFRSKFKDLEDKKVNQQPKTGDKDSSPQKEVKYEPFSFADDIGSNNCGYYDL

QAVLTHQGRSSSSGHYVSWVKRKQDEWIKFDDDKVSIVTPEDILRLSGGGDWHIAYVLLY

GPRRVEIMEEESEQ

>sp|Q3ZBT1|TERA_BOVIN Transitional endoplasmic reticulum ATPase OS=Bos taurus OX=9913 GN=VCP PE=2 SV=1

MASGADSKGDDLSTAILKQKNRPNRLIVDEAINEDNSVVSLSQPKMDELQLFRGDTVLLK

GKKRREAVCIVLSDDTCSDEKIRMNRVVRNNLRVHLGDVISIQPCPDVKYGKRIHVLPID

DTVEGITGNLFEVYLKPYFLEAYRPIRKGDIFLVRGGMRAVEFKVVETDPSPYCIVAPDT

VIHCEGEPIKREDEEESLNEVGYDDIGGCRKQLAQIKEMVELPLRHPALFKAIGVKPPRG

ILLYGPPGTGKTLIARAVANETGAFFFLINGPEIMSKLAGESESNLRKAFEEAEKNAPAI

IFIDELDAIAPKREKTHGEVERRIVSQLLTLMDGLKQRAHVIVMAATNRPNSIDPALRRF

GRFDREVDIGIPDATGRLEILQIHTKNMKLADDVDLEQVANETHGHVGADLAALCSEAAL

QAIRKKMDLIDLEDETIDAEVMNSLAVTMDDFRWALSQSNPSALRETVVEVPQVTWEDIG

GLEDVKRELQELVQYPVEHPDKFLKFGMTPSKGVLFYGPPGCGKTLLAKAIANECQANFI

SIKGPELLTMWFGESEANVREIFDKARQAAPCVLFFDELDSIAKARGGNIGDGGGAADRV

INQILTEMDGMSTKKNVFIIGATNRPDIIDPAILRPGRLDQLIYIPLPDEKSRVAILKAN

LRKSPVAKDVDLEFLAKMTNGFSGADLTEICQRACKLAIRESIESEIRRERERQTNPSAM

EVEEDDPVPEIRRDHFEEAMRFARRSVSDNDIRKYEMFAQTLQQSRGFGSFRFPSGNQGG

AGPSQGSGGGTGGNVYTEDNDDDLYG

>sp|Q2HJG5|VPS35_BOVIN Vacuolar protein sorting-associated protein 35 OS=Bos taurus OX=9913 GN=VPS35 PE=2 SV=1

MPTTQQSPQDEQEKLLDEAIQAVKVQSFQMKRCLDKNKLMDALKHASNMLGELRTSMLSP

KSYYELYMAISDELHYLEVYLTDEFAKGRKVADLYELVQYAGNIIPRLYLLITVGVVYVK

SFPQSRKDILKDLVEMCRGVQHPLRGLFLRNYLLQCTRNILPDEGEPTDEETTGDISDSM

DFVLLNFAEMNKLWVRMQHQGHSRDREKRERERQELRILVGTNLVRLSQLEGVNVERYKQ

IVLTGILEQVVNCRDALAQEYLMECIIQVFPDEFHLQTLNPFLRACAELHQNVNVKNIII

ALIDRLALFAHREDGPGIPTDIKLFDIFSQQVATVIQSRQDMPSEDVVSLQVSLINLAMK

CYPDRVDYVDKVLETTVEIFNKLNLEHIATSSAVSKELTRLLKIPVDTYNNILTVLKLKH

FHPLFEYFDYESRKSMSCYVLSNVLDYNTEIVSQDQVDSIMNLVSTLIQDQPDQPVEEPD

PEDFADEQSLVGRFIHLLRSEDPDQQYLILNTARKHFGAGGNQRIRFTLPPLVFAAYQLA

FRYKENSKVDDKWEKKCQKIFSFAHQTISALIKAELAELPLRLFLQGALAAGEIGFENHE

TVAYEFMSQAFSLYEDEISDSKAQLAAITLIIGTFERMKCFSEENHEPLRTQCALAASKL

LKKPDQGRAVSTCAHLFWSGRNTDKNGEELHGGKRVMECLKKALKIANQCMDPSLQVQLF

IEILNRYIYFYEKENDAVTIQVLNQLIQKIREDLPNLESSEETEQINKHFHNTLEHLRLR

RESPESEGPIYEGLIL

>sp|P80012|VWF_BOVIN von Willebrand factor (Fragment) OS=Bos taurus OX=9913 GN=VWF PE=1 SV=2

MFPTRLARLLLAVALTLPGALCGEGALGKSSMARCSLFGADFINTFDESMYSFSGDCSYL

LAGDCKTHSFSIVGDFQGGRRMGLSVYLGEFFDIHVFVNGTVLQGGQHVSMPYATRGLYL

ETEVGHHKLSSESYGFVARIDGSGNFQILLSDRHFNKTCGLCGDFNIFAEDDFRTQEGTL

TSDPYDFANSWALSSEEQRCPRVSPPSSSCNVSSELQKGLWEKCQLLKTASVFARCHALV

DPEPFVALCERMLCACAQGLRCPCPVLLEYARACAKQGMLLYGWADHSSCRPDCPTGMEY

KECVSPCHRTCRSLSITEVCREQCVDGCSCPEGQLLDEGRCVESTECPCVHAGKPYPPGA

SLSRDCNTCICRNSQWVCSNEDCPGECLITGQSHFKSFDDRHFTFSGVCQYLLAQDCQDH

SFSVVIETVQCADDPDAVCTRSVTVRLPSPHHGLLKLKHGGGVALDGQDVQIPLLQGDLR

IQHTVTASLQLNFGEDLQIDWDGRGRLLLKLSPVYAGRTCGLCGNYNGNQRDDFLTPAGL

VEPLVEHFGNSWKLRADCEDLQEQPSDPCSLNPRLTKFADQACAILTSPKFEACHSAVSP

LPYLRNCRYDVCACSDGRDCLCDAVANYAAACARRGVHVGWREPSFCALSCPHGQVYQQC

GTPCNLTCRSLSHPDEECTEVCLEGCFCPPGLFLDETGSCVPKAQCPCYYDGEIFQPEDI

FSDHHTMCYCEDGFMHSATSGAPGSLLPEAVLSSPLSHRSKRSLSCRPPMVKVVCPADNP

RAEGLECTKTCQNYDLECMSTGCVSGCLPAPGMVRHENRCVALERCPCFHQGREYAPGDR

VKVDCNSCVCQDRKWNCTDHVCDASCSALGLAHYFTFDGLKYLFPGECQYVLVQDHCGSN

PGTFRVLVGNEGCSVPSLKCRKRITILVEGGEIELFD

>sp|P17248|SYWC_BOVIN Tryptophan--tRNA ligase, cytoplasmic OS=Bos taurus OX=9913 GN=WARS1 PE=1 SV=3

MADMSNGEQGCGSPLELFHSIAAQGELVRDLKARNAAKDEIDSAVKMLLSLKTSYKAATG

EDYKVDCPPGDPAPESGEGLDATEADEDFVDPWTVQTSSAKGIDYDKLIVRFGSSKIDKE

LVNRIERATGQRPHRFLRRGIFFSHRDMHQILDAYENKKPFYLYTGRGPSSEAMHVGHLI

PFIFTKWLQDVFNVPLVIQMTDDEKYLWKDLTLDQAYGYAVENAKDIIACGFDINKTFIF

SDLDYMGMSPGFYKNVVKIQKHVTFNQVKGIFGFTDSDCIGKISFPAIQAAPSFSNSFPQ

IFRDRTDVQCLIPCAIDQDPYFRMTRDVAPRIGYPKPALLHSTFFPALQGAQTKMSASDP

NSSIFLTDTAKQIKTKVNKHAFSGGRDTVEEHRQFGGNCDVDVSFMYLTFFLEDDDKLEQ

IRRDYTSGAMLTGELKKELIEVLQPLIAEHQARRKEVTDEIVKEFMTPRKLSYDFQ

>sp|P68250|1433B_BOVIN 14-3-3 protein beta/alpha OS=Bos taurus OX=9913 GN=YWHAB PE=1 SV=2

MTMDKSELVQKAKLAEQAERYDDMAAAMKAVTEQGHELSNEERNLLSVAYKNVVGARRSS

WRVISSIEQKTERNEKKQQMGKEYREKIEAELQDICNDVLQLLDKYLIPNATQPESKVFY

LKMKGDYFRYLSEVASGDNKQTTVSNSQQAYQEAFEISKKEMQPTHPIRLGLALNFSVFY

YEILNSPEKACSLAKTAFDEAIAELDTLNEESYKDSTLIMQLLRDNLTLWTSENQGDEGD

AGEGEN

>sp|P68509|1433F_BOVIN 14-3-3 protein eta OS=Bos taurus OX=9913 GN=YWHAH PE=1 SV=2

MGDREQLLQRARLAEQAERYDDMASAMKAVTELNEPLSNEDRNLLSVAYKNVVGARRSSW

RVISSIEQKTMADGNEKKLEKVKAYREKIEKELETVCNDVLALLDKFLIKNCNDFQYESK

VFYLKMKGDYYRYLAEVASGEKKNSVVEASEAAYKEAFEISKEHMQPTHPIRLGLALNFS

VFYYEIQNAPEQACLLAKQAFDDAIAELDTLNEDSYKDSTLIMQLLRDNLTLWTSDQQDE

EAGEGN

>sp|Q7SIH1|A2MG_BOVIN Alpha-2-macroglobulin OS=Bos taurus OX=9913 GN=A2M PE=1 SV=2

MGKNKLLYPSLTLLLLLLLPTDASVSGKPQYMVLVPSLLHTETPEKGCLLLSHLNETVTV

SASLESVRENRSLFTDVVAEKDLFHCVSFTLPRSPTSQEVMFLTIQVKGPTQEFKKRTTV

LVKNEESLVFVQTDKPIYKPEQTVKFRIVLLDESFHPLNELVPLVYVEDPKGNRIAQWQN

LEVENGLQQLTFPLSSEPFQGSYKVVVQKGSGGTAEHPFTVEEFVLPKFEVQVRMPKIIT

ILEEEVQVSVCGLYTYGKPVPGRVTMNMCRKYRNPSNCYGEESNAVCEKFSGELNNEGCF

SQQVNTKIFQLKRQEFEMKIEVEAKIQEEGTEVELTGKGATEITTTITKLSFVTVDSNLR

RGIPFTGKVLLVDGKGVPMPNKVIFITANEANHNSNTTTDEHGLAQFSITTTKIKGTSLS

IRVKYKDHSPCYGYQWLSEEHQDAYHSANLVFSRSNSFVYLEPLPRELPCGKTQTVQAHY

VLKGQVLKDLKELVFYYLIMAKGGIVRSGTHTLPVEQGDMQGHFSMSVPVESDIAPVARL

LIYAILPDGEVVGDSARYEIEHCLANKVGLNFSPGQSFPASQAHLRVTASPQSLCALRAV

DQSVLLMRPEAELSAATVYNLLPVKDLSSFPSSVNQQEEDNEDCISHDNVYINGIMYFPV

SNTNEKDMYSFLQDMGLKAFTNSKIHKPKICPQPEEHRIQHHTLLASPVRAEMGRNRDFV

HFDDTSEPPTETVRKYFPETWIWDLVVVSSSGVHEVEVTVPDTITEWKAGALCLSRDTGL

GLSPTASLRVFQPFFVELTMPYSVIRGEAFTLKATVLNYLPKCIRVSVQLEASPAFLAVP

EKEQETYCICGNGRQTVSWAVTPKSLGNVNFTVSAEAVESQELCGSEVPVVPEHGRKDTI

IKPLLVEPEGLEKEVIFNSLLCPSVDFVFLGAEDGGQVLRHFPPAAATDTAADAHDPARP

GAKVSESLSLKLPPNVVEESARASFSVLGDILGSAMRNTQNLLQMPYGCGEQNMARFAPN

IYVLDYLNETQQLTAELKSKAILYLNTGYQRQLLYKHFDGSYSTFGEHRGNSEGNTWLTA

FVLKSFAQARGYIFIDEAHITEALTWLAQKQKSNGCFRSTGTLLNNAIKGGVDDEVTLSA

YITIALLEMPLPVTHPVVRNALFCLDSAWKSAKEGSQGSHVYTKALLAYAFALAGNQERR

TEVLTSLYEEAVKEDNTIHWTRPQKPRLLTEDIYQPRAPSAEVEMTAYVILAHVTAQPAP

NPEDLKRATSIVKWISKQQNCQGGFSSTQDTVVALHALSRYGAATFTSARKAAQVTIQSS

GTFSTKFQVENSNRLLLQQVSLPEVPGEYSMSVTGEGCVYLQTSLKYNILPKKDEFPFAL

EVQTLPQTCDGPKAHTSFQISLSVSYIGSRPASNMAIVDVKMVSGFIPLKPTVKMLERSN

VSRTEVSNNHVLIYLDKVTNETLTLTFTVLQDIPVRDLKPAIVKVYDYYETDEFAVAEYS

APCSKDIGNA

>sp|Q2UVX4|CO3_BOVIN Complement C3 OS=Bos taurus OX=9913 GN=C3 PE=1 SV=2

MKPTSGPSLLLLLLASLPMALGNPMYSMITPNILRLESEETVVLEAHGGQGTIQVSVTVH

DFPAKKQVLSNENTQLNSNNGYLSTVTIKIPASKELKSDKGHKFVTVVATFGNVQVEKVV

LISLQSGYLFIQTDKTIYTPGSTVLYRVFTVDHKLLPVGQTVFITIETPDGIPVKRDSKS

SQNQFGILTLSWNIPELVNMGVWKIKAYYEDSPQQVFSAEFEVKEYVLPSFEVQLEPEEK

FYYIDDPDGLKVNIIARFLYGEQVDGTAFVIFGVQDGDRRISLTHSLTRVPINDGNGEAI

LKRQVLLNGVQPSRADALVGKSIYVSATVILQSGSDMVEAERTGIPIVTSPYQIHFTKTP

KFFKPAMPFDLMVYVTNPDGSPARHIPVVTQGSNVQSLTQDDGVAKLSINTQNKRDPLTI

TVRTKKDNIPEGRQATRTMQALPYNTQGNSNNYLHLSVPRVELKPGETLNVNFHLRTDPG

EQAKIRYYTYMIMNKGKLLKVGRQYREPGQDLVVLPLTITSDFIPSFRLVAYYTLINAKG

QREVVADSVWVDVKDSCMGTLVVKNGGKEEKHHRPGQQITLKIEADQGARVGLVAVDKGV

FVLNKKNKLTQRKIWDVVEKADIGCTPGSGRNYAGVFTDAGLTLKTSQGLETQQRADPQC

PQPATRRRRSVQLMEKRMDKAGQYSSDLRKCCEDGMRDNPMKFPCQRRAQFILQGDACVK

AFLDCCEYITQLRQQHSRDGALELARSDLDDDIIPEEDIISRSQFPESWLWTVIEDLKQA

DKNGISTKLMNVFLKDSITTWEILAVSLSDKKGICVADPYEVTVMQDFFIDLRLPYSVVR

NEQVEIRAILYNYREAENLKVRVELLYNPAFCSLATAKKRHQQTITIPARSSVAVPYVIV

PLKIGLHEVEVKAAVYNHFISDGVKKTLKVVPEGVRVNKTVAVRTLNPEHLGQGGVQREE

VPAADLSDQVPDTESETKILLQGTPVAQMTEDAIDGERLKHLIQTPSGCGEQNMIGMTPT

VIAVHYLDSTDQWEKFGLEKRQESLELIRKGYTQQLAFRQKSSAYAAFQYRPPSTWLTAY

VVKVFALAANLIAIDSKDLCETVKWLILEKQKPDGIFQEDGPVIHQEMIGGFRDTREKDV

SLTAFVLIALHEAKDICEAQVNSLGRSIAKAGDFLENHYRELRRPYTVAIAAYALALLGK

LEGDRLTKFLNTAKEKNRWEEPNQKLYNVEATSYALLALLARKDYDTTPPVVRWLNEQRY

YGGGYGSTQATFMVFQALAQYQKDVPDHKELNLDVSIQLPSRNSAVRHRILWESASLLRS

EETKENERFTVKAEGKGQGTLSVVTVYHAKLKGKVSCKKFDLRVSIRPAPETVKKPQDAK

GSMILDICTKYLGDQDATMSILDISMMTGFSPDVEDLKTLSTGVDRYISKYEMNRDSNKN

TLIIYLDKVSHTVEDCLSFKVHQYFNVGLIQPGAVKVYSYYNLDETCIRFYHPDKEDGML

SKLCHKDTCRCAEENCFMHHTEKEVTLEDRLDKACEPGVDYVYKTRLIQKKLEDDFDEYI

MVIENIIKSGSDEVQVKQERKFISHIKCREALKLKEGAHYLVWGVSSDLWGEKPKISYII

GKDTWVELWPEAEECQDEENQKQCEDLANFTENMVVFGCPN

>sp|P02769|ALBU_BOVIN Albumin OS=Bos taurus OX=9913 GN=ALB PE=1 SV=4

MKWVTFISLLLLFSSAYSRGVFRRDTHKSEIAHRFKDLGEEHFKGLVLIAFSQYLQQCPF

DEHVKLVNELTEFAKTCVADESHAGCEKSLHTLFGDELCKVASLRETYGDMADCCEKQEP

ERNECFLSHKDDSPDLPKLKPDPNTLCDEFKADEKKFWGKYLYEIARRHPYFYAPELLYY

ANKYNGVFQECCQAEDKGACLLPKIETMREKVLASSARQRLRCASIQKFGERALKAWSVA

RLSQKFPKAEFVEVTKLVTDLTKVHKECCHGDLLECADDRADLAKYICDNQDTISSKLKE

CCDKPLLEKSHCIAEVEKDAIPENLPPLTADFAEDKDVCKNYQEAKDAFLGSFLYEYSRR

HPEYAVSVLLRLAKEYEATLEECCAKDDPHACYSTVFDKLKHLVDEPQNLIKQNCDQFEK

LGEYGFQNALIVRYTRKVPQVSTPTLVEVSRSLGKVGTRCCTKPESERMPCTEDYLSLIL

NRLCVLHEKTPVSEKVTKCCTESLVNRRPCFSALTPDETYVPKAFDEKLFTFHADICTLP

DTEKQIKKQTALVELLKHKPKATEEQLKTVMENFVAFVDKCCAADDKEACFAVEGPKLVV

STQTALA

>sp|P00735|THRB_BOVIN Prothrombin OS=Bos taurus OX=9913 GN=F2 PE=1 SV=2

MARVRGPRLPGCLALAALFSLVHSQHVFLAHQQASSLLQRARRANKGFLEEVRKGNLERE

CLEEPCSREEAFEALESLSATDAFWAKYTACESARNPREKLNECLEGNCAEGVGMNYRGN

VSVTRSGIECQLWRSRYPHKPEINSTTHPGADLRENFCRNPDGSITGPWCYTTSPTLRRE

ECSVPVCGQDRVTVEVIPRSGGSTTSQSPLLETCVPDRGREYRGRLAVTTSGSRCLAWSS

EQAKALSKDQDFNPAVPLAENFCRNPDGDEEGAWCYVADQPGDFEYCDLNYCEEPVDGDL

GDRLGEDPDPDAAIEGRTSEDHFQPFFNEKTFGAGEADCGLRPLFEKKQVQDQTEKELFE

SYIEGRIVEGQDAEVGLSPWQVMLFRKSPQELLCGASLISDRWVLTAAHCLLYPPWDKNF

TVDDLLVRIGKHSRTRYERKVEKISMLDKIYIHPRYNWKENLDRDIALLKLKRPIELSDY

IHPVCLPDKQTAAKLLHAGFKGRVTGWGNRRETWTTSVAEVQPSVLQVVNLPLVERPVCK

ASTRIRITDNMFCAGYKPGEGKRGDACEGDSGGPFVMKSPYNNRWYQMGIVSWGEGCDRD

GKYGFYTHVFRLKKWIQKVIDRLGS

>sp|P01267|THYG_BOVIN Thyroglobulin OS=Bos taurus OX=9913 GN=TG PE=1 SV=1

MALALWVFGLLDLICLASANIFEYQVDAQPLRPCELQRERAFLKREDYVPQCAEDGSFQT

VQCGKDGASCWCVDADGREVPGSRQPGRPAACLSFCQLQKQQILLSSYINSTATSYLPQC

QDSGDYSPVQCDLRRRQCWCVDAEGMEVYGTRQQGRPARCPRSCEIRNRRLLHGVGDRSP

PQCSPDGAFRPVQCKLVNTTDMMIFDLVHSYSRFPDAFVTFSSFRSRFPEVSGYCYCADS

QGRELAETGLELLLDEIYDTIFAGLDLASTFAETTLYRILQRRFLAVQLVISGRFRCPTK

CEVERFAATSFRHPYVPSCHPDGEYQAAQCQQGGPCWCVDSRGQEIPGTRQRGEPPSCAE

DQSCPSERRRAFSRLRFGPSGYFSRRSLLLAPEEGPVSQRFARFTASCPPSIKELFLDSG

IFQPMLQGRDTRFVAPESLKEAIRGLFPSRELARLALQFTTNAKRLQQNLFGGRFLVKVG

QFNLSGALGTRGTFNFSHFFQQLGLPGFQDGRALADLAKPLSVGLNSNPASEAPKASKID

VALRKPVVGSFGFEVNLQENQNALQFLSSFLELPEFLLFLQHAISVPEDIARDLGDVMEM

VFSSQGCGQAPGSLFVPACTAEGSYEEVQCFAGDCWCVDAQGRELAGSRVRGGRPRCPTE

CEKQRARMQSLLGSQPAGSSLFVPACTSKGNFLPVQCFNSECYCVDTEGQPIPGTRSALG

EPKKCPSPCQLQAERAFLGTVRTLVSNPSTLPALSSIYIPQCSASGQWSPVQCDGPPEQA

FEWYERWEAQNSAGQALTPAELLMKIMSYREAASRNFRLFIQNLYEAGQQGIFPGLARYS

SFQDVPVSVLEGNQTQPGGNVFLEPYLFWQILNGQLDRYPGPYSDFSAPLAHFDLRSCWC

VDEAGQKLEGTRNEPNKVPACPGSCEEVKLRVLQFIREAEEIVTYSNSSRFPLGESFLAA

KGIRLTDEELAFPPLSPSRETFLEKFLSGSDYAIRLAAQSTFDFYQRRLVTLAESPRAPS

PVWSSAYLPQCDAFGGWEPVQCHAATGHCWCVDGKGEYVPTSLTARSRQIPQCPTSCERL

RASGLLSSWKQAGVQAEPSPKDLFIPTCLETGEFARLQASEAGTWCVDPASGEGVPPGTN

SSAQCPSLCEVLQSGVPSRRTSPGYSPACRAEDGGFSPVQCDPAQGSCWCVLGSGEEVPG

TRVAGSQPACESPQCPLPFSVADVAGGAILCERASGLGAAAGQRCQLRCSQGYRSAFPPE

PLLCSVQRRRWESRPPQPRACQRPQFWQTLQTQAQFQLLLPLGKVCSADYSGLLLAFQVF

LLDELTARGFCQIQVKTAGTPVSIPVCDDSSVKVECLSRERLGVNITWKLQLVDAPPASL

PDLQDVEEALAGKYLAGRFADLIQSGTFQLHLDSKTFSADTSIRFLQGDRFGTSPRTQFG

CLEGFGRVVAASDASQDALGCVKCPEGSYFQDEQCIPCPAGFYQEQAGSLACVPCPEGRT

TVYAGAFSQTHCVTDCQKNEVGLQCDQDSQYRASQRDRTSGKAFCVDGEGRRLPWTEAEA

PLVDAQCLVMRKFEKLPESKVIFSADVAVMVRSEVPGSESSLMQCLADCALDEACGFLTV

STAGSEVSCDFYAWASDSIACTTSGRSEDALGTSQATSFGSLQCQVKVRSREGDPLAVYL

KKGQEFTITGQKRFEQTGFQSALSGMYSPVTFSASGASLAEVHLFCLLACDHDSCCDGFI

LVQVQGGPLLCGLLSSPDVLLCHVRDWRDPAEAQANASCPGVTYDQDSRQVTLRLGGQEI

RGLTPLEGTQDTLTSFQQVYLWKDSDMGSRSESMGCRRDTEPRPASPSETDLTTGLFSPV

DLIQVIVDGNVSLPSQQHWLFKHLFSLQQANLWCLSRCAGEPSFCQLAEVTDSEPLYFTC

TLYPEAQVCDDILESSPKGCRLILPRRPSALYRKKVVLQDRVKNFYNRLPFQKLTGISIR

NKVPMSDKSISSGFFECERLCDMDPCCTGFGFLNVSQLKGGEVTCLTLNSLGLQTCSEEY

GGVWRILDCGSPDTEVRTYPFGWYQKPVSPSDAPSFCPSVALPALTENVALDSWQSLALS

SVIVDPSIRNFDVAHISTAAVGNFSAARDRCLWECSRHQDCLVTTLQTQPGAVRCMFYAD

TQSCTHSLQAQNCRLLLHEEATYIYRKPNIPLPGFGTSSPSVPIATHGQLLGRSQAIQVG

TSWKPVDQFLGVPYAAPPLGEKRFRAPEHLNWTGSWEATKPRARCWQPGIRTPTPPGVSE

DCLYLNVFVPQNMAPNASVLVFFHNAAEGKGSGDRPAVDGSFLAAVGNLIVVTASYRTGI

FGFLSSGSSELSGNWGLLDQVVALTWVQTHIQAFGGDPRRVTLAADRGGADIASIHLVTT

RAANSRLFRRAVLMGGSALSPAAVIRPERARQQAAALAKEVGCPSSSVQEMVSCLRQEPA

RILNDAQTKLLAVSGPFHYWGPVVDGQYLRETPARVLQRAPRVKVDLLIGSSQDDGLINR

AKAVKQFEESQGRTSSKTAFYQALQNSLGGEAADAGVQAAATWYYSLEHDSDDYASFSRA

LEQATRDYFIICPVIDMASHWARTVRGNVFMYHAPESYSHSSLELLTDVLYAFGLPFYPA

YEGQFTLEEKSLSLKIMQYFSNFIRSGNPNYPHEFSRRAPEFAAPWPDFVPRDGAESYKE

LSVLLPNRQGLKKADCSFWSKYIQSLKASADETKDGPSADSEEEDQPAGSGLTEDLLGLP

ELASKTYSK

>sp|P12763|FETUA_BOVIN Alpha-2-HS-glycoprotein OS=Bos taurus OX=9913 GN=AHSG PE=1 SV=2

MKSFVLLFCLAQLWGCHSIPLDPVAGYKEPACDDPDTEQAALAAVDYINKHLPRGYKHTL

NQIDSVKVWPRRPTGEVYDIEIDTLETTCHVLDPTPLANCSVRQQTQHAVEGDCDIHVLK

QDGQFSVLFTKCDSSPDSAEDVRKLCPDCPLLAPLNDSRVVHAVEVALATFNAESNGSYL

QLVEISRAQFVPLPVSVSVEFAVAATDCIAKEVVDPTKCNLLAEKQYGFCKGSVIQKALG

GEDVRVTCTLFQTQPVIPQPQPDGAEAEAPSAVPDAAGPTPSAAGPPVASVVVGPSVVAV

PLPLHRAHYDLRHTFSGVASVESSSGEAFHVGKTPIVGQPSIPGGPVRLCPGRIRYFKI

>sp|Q28107|FA5_BOVIN Coagulation factor V OS=Bos taurus OX=9913 GN=F5 PE=1 SV=1

MFLACPGFWVLVVLGSSWAGWGNLGAEAAKLRQFYVAAQSIRWNYRPESTHLSSKPFETS

FKKIVYREYEAYFQKEKPQSRTSGLLGPTLYAEVGDIMKVHFKNKAHKPLSIHAQGIKYS

KFSEGASYSDHTLPMEKMDDAVAPGQEYTYEWIISEHSGPTHDDPPCLTHIYYSYVNLVE

DFNSGLIGPLLICKKGTLTEDGTQKMFEKQHVLMFAVFDESKSWNQTSSLMYTVNGYVNG

TMPDITVCAHDHISWHLIGMSSGPELFSIHFNGQVLEQNHHKISAITLVSATSTTANMTV

SPEGRWTIASLIPRHFQAGMQAYIDIKNCAKKTRNPKKLTRDQRRHIKRWEYFIAAEEVI

WDYAPIIPANMDKKYRSLHLDNFSNRIGKHYKKVVYKQYQDDSFTKRLEDPSSEGDGILG

PIIRAQVRDTLKIVFKNMASRSYSIYPHGVTFSPYDNEVNSSSTSGSNTMIRAVRPGETY

TYKWNILESDEPTENDAQCLTRPYYSNVDITRDLASGLIGLLLICKSRSLDRRGIQRAAD

IEQQAVFAVFDENKSWYIEDNIYKFCENPEKVKRDDPKFYESNIMSNFTLPAINGYVPES

IPILGFCFDDTVQWHFCSVGTQNDILTIHFTGHSFIYGKRHEDTLTLFPMQGESVTVTMD

NVGTWMLTTMNSNPRSKKLRLRFRDAKCIRNDDDDSYEIIYEPSGSTAMTTKKIHDSSEI

EDENDADSDYQDELALILGLRSFRNSSLNQEKDELNLTALALEKDSEFIPPSANRSLDSN

SSSRSHVSRLIAKNFAESLKTLLHLEAPAAGSPLEHAGLDKNSALNPPMAEHSSPYSEDP

REDHPLSDVTGVSLLPFGTGFKNRKPAKHQRFQVGRGQAAKHKFSQTRFPAHKTRTRLSQ

DNSSSSRMGPWEDIPSDLLLLQQKDPYKILNGEWHLVSEKGSYEIIQDANENKTVNKLPN

SPQNDSRTWGENIPFKNSHGKQSGHPTFLVTRRKPLQDRQDRRNSRLKEGLPLIRTRRKK

KEEKPAYHVPLSPRSFHPLRGEVNASFSDRRHNHSLLLHASNETSLSIDLNQTFPSMNLS

LAASLPDHDQTSPNDTTSQTSSPPDLYPTVSPEEHYQIFPIQDSDPTHSTTAPSNRSPDP

THSTTAPSNRSPPTQPSQIPNYDLRNRAIPTDVSQIFPSLELEVWQTATSLDLSQPSISP

DLGQMALSPDPGQESLSPDLGQTSLSPDLSQESLSPDLGQTALSPDPSQESLSPDLGQTA

LSPDPSQESLSPDLGQTALSPDPGQESLSPDLGQTSLSPDLSQESLSPDLGQTALSPDPS

QESLSPDLGQTALSPDPSQESLSPDLGQTSLSPDLGQESLSPDLGQTALSPDPSQESLSP

DLGQTSLSPDLGQESLSPDLGQTALSPDLSQESLSPDLGQTPLSPDLSLESLSPDLSQLD

LKQTSPPLDLNQTSHTSESSQSLPLPEFGQTFPNADIGQMPSPPPDSTLNNTFIPEEFNP

LVVVGLSRDDGDYIEIIPRQKEESSEEDYGEFEFVAYNDPYQTDLRTDINSSRNPDNIAA

WYLRSNTGNRKYYYIAAEEISWDYSKFVQSDDVDYVPEDTVYKKVVFRKYLDSTFTKLDP

QGEYEEHLGILGPVIRAEVDDVIQVRFKNLASRPYSLHAHGLSYEKSSEGKTYEDDSPEW

FKEDNAIQPNKTYTYVWHATTRSGPENPGSACRAWAYYSAVNPEKDIHSGLIGPLLICRK

GTLDKETNMPVDMREFVLLFMVFDEKKSWYYDKKPTRSWRRASSEVKNSHEFHAINGMIY

NLPGLRMYEQEWVRLHLLNLGGSRDIHVVHFHGQTLLENGTQQHQLGVWPLLPGSFKTLE

MKASKPGWWLLDTEVGEIQRAGMQTPFLIVDRECKMPMGLSTGLIADSQIQASEFWGYWE

PKLARLNNGGSYNAWIAEKLSTEFNPEPWIQVDMQKEVLLTGIQTQGAKHYLKPYYTTEF

CVAYSLDRKNWRIFKGNSTRNVMYFGGNSDASTIKENQIDPPVVARYIRISPTGSYNKPA

LRLELQGCEVNGCSTPLGMESGKIENKQITASSFKKSWWGNYWEPFLARLNAQGRVNAWQ

AKANNNNQWLQIDLLKIKKITAIVTQGCKSLSSEMYVKSYTIHYSDQGTDWKPYREKSSM

VDKIFEGNNNVRGHVKNFFNPPIISRFIRIIPKTWNQSIALRLELFGCDMY

>sp|Q29443|TRFE_BOVIN Serotransferrin OS=Bos taurus OX=9913 GN=TF PE=2 SV=1

MRPAVRALLACAVLGLCLADPERTVRWCTISTHEANKCASFRENVLRILESGPFVSCVKK

TSHMDCIKAISNNEADAVTLDGGLVYEAGLKPNNLKPVVAEFHGTKDNPQTHYYAVAVVK

KDTDFKLNELRGKKSCHTGLGRSAGWNIPMAKLYKELPDPQESIQRAAANFFSASCVPCA

DQSSFPKLCQLCAGKGTDKCACSNHEPYFGYSGAFKCLMEGAGDVAFVKHSTVFDNLPNP

EDRKNYELLCGDNTRKSVDDYQECYLAMVPSHAVVARTVGGKEDVIWELLNHAQEHFGKD

KPDNFQLFQSPHGKDLLFKDSADGFLKIPSKMDFELYLGYEYVTALQNLRESKPPDSSKD

ECMVKWCAIGHQERTKCDRWSGFSGGAIECETAENTEECIAKIMKGEADAMSLDGGYLYI

AGKCGLVPVLAENYKTEGESCKNTPEKGYLAVAVVKTSDANINWNNLKDKKSCHTAVDRT

AGWNIPMGLLYSKINNCKFDEFFSAGCAPGSPRNSSLCALCIGSEKGTGKECVPNSNERY

YGYTGAFRCLVEKGDVAFVKDQTVIQNTDGNNNEAWAKNLKKENFEVLCKDGTRKPVTDA

ENCHLARGPNHAVVSRKDKATCVEKILNKQQDDFGKSVTDCTSNFCLFQSNSKDLLFRDD

TKCLASIAKKTYDSYLGDDYVRAMTNLRQCSTSKLLEACTFHKP

>sp|P02081|HBBF_BOVIN Hemoglobin fetal subunit beta OS=Bos taurus OX=9913 PE=1 SV=1

MLSAEEKAAVTSLFAKVKVDEVGGEALGRLLVVYPWTQRFFESFGDLSSADAILGNPKVK

AHGKKVLDSFCEGLKQLDDLKGAFASLSELHCDKLHVDPENFRLLGNVLVVVLARRFGSE

FSPELQASFQKVVTGVANALAHRYH

>sp|P01017|ANGT_BOVIN Angiotensinogen OS=Bos taurus OX=9913 GN=AGT PE=1 SV=2

MAPAGLSLGAAILCLLAWAGLAAGDRVYVHPFHLLVYSKSNCDQLEKPSVETPPDPTFTP

VPIQTKSSAVDEEALWEQLVRATEKLEAEDRLRASEVGLLLNFMGFHMYKTLSETWSVAS

GAVFSPVALFSTLTSFYVGALDPTASRLQAFLGVPGEGQGCTSRLDGHKVLSSLQTIQGL

LVAQGGASSQARLLLSTVVGLFTAPGLHLKQPFVQSLSSFAPITLPRSLDLSTDPNLAAE

KINRFMQSVTGWNMGRALTAVSPDSTLLFNAYVHFQGKMKGFSLLPGLKEFWVDNTTSVS

VPMLSGTGIFHFWSDSQNNLSVTRVPLSANTYLLLIQPHHTPDLRKVEALTFQHNFLTRM

KNLSPRAIHLTMPQLTLKASYDLQDLLAQAKLPTLLGAEANLSKISDANLRVGKVLNSVL

FELKADGEQAPESVPQPAGPEALEVTLNSPFLLAVLERSSGALHFLGRVSRPLSAE

>sp|Q3T052|ITIH4_BOVIN Inter-alpha-trypsin inhibitor heavy chain H4 OS=Bos taurus OX=9913 GN=ITIH4 PE=1 SV=1

MKTPAPGRIHSIVLVLLSLAVLQTSKAQKVQNDIDIYSLTVDSKVSSRFAHTVITSRVVN

KADAVREATFQMELPKKAFITNFSMVIDGVTYPGNIKEKAAAQEQYSAAVARGESAGLVR

ATGRKTEQFQVSVSVAPAAKVTFELVYEELLARHLGAYELLLKVRPQQLVKHLQMDIHIF

EPQGISFLETESTFMTNKLAEALTTSQNKTKAHVRFKPTLSQQQKYPEKQDTVIDGSFIV

RYDVDRPLSGGSIQIENGYFVHYFAPDSLSTIPKNVIFVIDKSGSMMGRKIKQTREALIK

ILDDLSPHDQFDLISFSSEATTWKPLLVPASTENVNEAKSYATGIQAQGGTNINDAMLMA

VQLLEKANQEELLPEGSITLIILLTDGDPTVGETNPLNIQKNVRKAINGQHSLFCLGFGF

DVSYAFLEKMALENGGLARRIYEDSDSALQLQDFYQEVANPLMTSVAFEYPSNAVESVTQ

DTFRVFFKGSELVVAGKLREQSPDVLLAQIRGQLHRENITYMMMSHVAEQEEMFRSPKYI

FHSFIERLWAYLTIQQLLEQMVSALDAEKQALEARALSLSLSYSFVTPLTSMVITKPEGQ

EQSQVAEKPVEDESRGSRVYLGPMRFGHSVGDRTSRKPGGGLKLLNGTPLFGPPGPPAAA

SPFHRMTSRLVLPELMSPLAPASAPSPTSGPGGASHDTDFRIKGTTPTALPFAPVQAPSV

ILPLPGQSVDRLCVDLRRPQELVNLLSDPDQGVEVTGHFETAKARFSWIEVTFENPQVQI

HASPEHVVMTRNRRNSAYKWKETLYSVMPGLKVTMDKEGLLLLSRPDRVTIGLLFWDGPG

KGLRLLLQNTDRFSSHVSGTLGQFYQDVLWGPLDTADDSKRTLKVQGRDYSATRELKLDY

QESPPGKEISCWSVEL

>sp|P07589|FINC_BOVIN Fibronectin OS=Bos taurus OX=9913 GN=FN1 PE=1 SV=4

MLGGPGPGLLLLLAVLSLGTAVPSAGASKSRRQAQQIVQPQSPLTVSQSKPGCYDNGKHY

QINQQWERTYLGSALVCTCYGGSRGFNCESKPEPEETCFDKYTGNTYRVGDTYERPKDSM

IWDCTCIGAGRGRISCTIANRCHEGGQSYKIGDTWRRPHETGGYMLECVCLGNGKGEWTC

KPIAEKCFDQAAGTSYVVGETWEKPYQGWMMVDCTCLGEGSGRITCTSRNRCNDQDTRTS

YRIGDTWSKKDNRGNLLQCICTGNGRGEWKCERHTSLQTTSAGSGSFTDVRTAIYQPQPH

PQPPPYGHCVTDSGVVYSVGMQWLKTQGNKQMLCTCLGNGVSCQETAVTQTYGGNSNGEP

CVLPFTYNGKTFYSCTTEGRQDGHLWCSTTSNYEQDQKYSFCTDHTVLVQTRGGNSNGAL

CHFPFLYNNHNYTDCTSEGRRDNMKWCGTTQNYDADQKFGFCPMAAHEEICTTNEGVMYR

IGDQWDKQHDMGHMMRCTCVGNGRGEWTCVAYSQLRDQCIVDGITYNVNDTFHKRHEEGH

MLNCTCFGQGRGRWKCDPVDQCQDSETRTFYQIGDSWEKYLQGVRYQCYCYGRGIGEWAC

QPLQTYPDTSGPVQVIITETPSQPNSHPIQWSAPESSHISKYILRWKPKNSPDRWKEATI

PGHLNSYTIKGLRPGVVYEGQLISVQHYGQREVTRFDFTTTSTSPAVTSNTVTGETTPLS

PVVATSESVTEITASSFVVSWVSASDTVSGFRVEYELSEEGDEPQYLDLPSTATSVNIPD

LLPGRKYTVNVYEISEEGEQNLILSTSQTTAPDAPPDPTVDQVDDTSIVVRWSRPRAPIT

GYRIVYSPSVEGSSTELNLPETANSVTLSDLQPGVQYNITIYAVEENQESTPVFIQQETT

GVPRSDKVPPPRDLQFVEVTDVKITIMWTPPESPVTGYRVDVIPVNLPGEHGQRLPVSRN

TFAEVTGLSPGVTYHFKVFAVNQGRESKPLTAQQATKLDAPTNLQFINETDTTVIVTWTP

PRARIVGYRLTVGLTRGGQPKQYNVGPAASQYPLRNLQPGSEYAVSLVAVKGNQQSPRVT

GVFTTLQPLGSIPHYNTEVTETTIVITWTPAPRIGFKLGVRPSQGGEAPREVTSESGSIV

VSGLTPGVEYVYTISVLRDGQERDAPIVKKVVTPLSPPTNLHLEANPDTGVLTVSWERST

TPDITGYRITTTPTNGQQGYSLEEVVHADQSSCTFENLSPGLEYNVSVYTVKDDKESVPI

SDTIIPEVPQLTDLSFVDITDSSIGLRWTPLNSSTIIGYRITVVAAGEGIPIFEDFVDSS

VGYYTVTGLEPGIDYDISVITLINGGESAPTTLTQQTAVPPPTDLRFTNVGPDTMRVTWA

PPSSIELTNLLVRYSPVKNEEDVAELSISPSDNAVVLTNLLPGTEYLVSVSSVYEQHESI

PLRGRQKTALDSPSGIDFSDITANSFTVHWIAPRATITGYRIRHHPENMGGRPREDRVPP

SRNSITLTNLNPGTEYVVSIVALNSKEESLPLVGQQSTVSDVPRDLEVIAATPTSLLISW

DAPAVTVRYYRITYGETGGSSPVQEFTVPGSKSTATISGLKPGVDYTITVYAVTGRGDSP

ASSKPVSINYRTEIDKPSQMQVTDVQDNSISVRWLPSSSPVTGYRVTTAPKNGPGPSKTK

TVGPDQTEMTIEGLQPTVEYVVSVYAQNQNGESQPLVQTAVTNIDRPKGLAFTDVDVDSI

KIAWESPQGQVSRYRVTYSSPEDGIHELFPAPDGEEETAELQGLRPGSEYTVSVVALHDD

MESQPLIGTQSTTIPAPTNLKFTQVTPTSLTAQWTAPNVQLTGYRVRVTPKEKTGPMKEI

NLAPDSSSVVVSGLMVATKYEVSVYALKDTLTSRPAQGVVTTLENVSPPRRARVTDATET

TITISWRTKTETITGFQVDAIPANGQTPIQRTIRPDVRSYTITGLQPGTDYKIHLYTLND

NARSSPVVIDASTAIDAPSNLRFLATTPNSLLVSWQPPRARITGYIIKYEKPGSPPREVV

PRPRPGVTEATITGLEPGTEYTIQVIALKNNQKSEPLIGRKKTDELPQLVTLPHPNLHGP

EILDVPSTVQKTPFITNPGYDTGNGIQLPGTSGQQPSLGQQMIFEEHGFRRTTPPTTATP

VRHRPRPYPPNVNEEIQIGHVPRGDVDHHLYPHVVGLNPNASTGQEALSQTTISWTPFQE

SSEYIISCHPVGIDEEPLQFRVPGTSASATLTGLTRGATYNIIVEAVKDQQRQKVREEVV

TVGNSVDQGLSQPTDDSCFDPYTVSHYAIGEEWERLSDSGFKLSCQCLGFGSGHFRCDSS

KWCHDNGVNYKIGEKWDRQGENGQMMSCTCLGNGKGEFKCDPHEATCYDDGKTYHVGEQW

QKEYLGAICSCTCFGGQRGWRCDNCRRPGAEPGNEGSTAHSYNQYSQRYHQRTNTNVNCP

IECFMPLDVQADREDSRE

>sp|P06868|PLMN_BOVIN Plasminogen OS=Bos taurus OX=9913 GN=PLG PE=1 SV=2

MLPASPKMEHKAVVFLLLLFLKSGLGDLLDDYVNTQGASLLSLSRKNLAGRSVEDCAAKC

EEETDFVCRAFQYHSKEQQCVVMAENSKNTPVFRMRDVILYEKRIYLLECKTGNGQTYRG

TTAETKSGVTCQKWSATSPHVPKFSPEKFPLAGLEENYCRNPDNDENGPWCYTTDPDKRY

DYCDIPECEDKCMHCSGENYEGKIAKTMSGRDCQAWDSQSPHAHGYIPSKFPNKNLKMNY

CRNPDGEPRPWCFTTDPQKRWEFCDIPRCTTPPPSSGPKYQCLKGTGKNYGGTVAVTESG

HTCQRWSEQTPHKHNRTPENFPCKNLEENYCRNPNGEKAPWCYTTNSEVRWEYCTIPSCE

SSPLSTERMDVPVPPEQTPVPQDCYHGNGQSYRGTSSTTITGRKCQSWSSMTPHRHLKTP

ENYPNAGLTMNYCRNPDADKSPWCYTTDPRVRWEFCNLKKCSETPEQVPAAPQAPGVENP

PEADCMIGTGKSYRGKKATTVAGVPCQEWAAQEPHQHSIFTPETNPQSGLERNYCRNPDG

DVNGPWCYTMNPRKPFDYCDVPQCESSFDCGKPKVEPKKCSGRIVGGCVSKPHSWPWQVS

LRRSSRHFCGGTLISPKWVLTAAHCLDNILALSFYKVILGAHNEKVREQSVQEIPVSRLF

REPSQADIALLKLSRPAIITKEVIPACLPPPNYMVAARTECYITGWGETQGTFGEGLLKE

AHLPVIENKVCNRNEYLDGRVKPTELCAGHLIGGTDSCQGDSGGPLVCFEKDKYILQGVT

SWGLGCARPNKPGVYVRVSPYVPWIEETMRRN

>sp|P41361|ANT3_BOVIN Antithrombin-III OS=Bos taurus OX=9913 GN=SERPINC1 PE=1 SV=2

MISNGIGTVTAGKRSICLLPLLLIGLWGCVTCHRSPVEDVCTAKPRDIPVNPMCIYRSSE

KKATEGQGSEQKIPGATNRRVWELSKANSHFATAFYQHLADSKNNNDNIFLSPLSISTAF

AMTKLGACNNTLKQLMEVFKFDTISEKTSDQIHFFFAKLNCRLYRKANKSSELVSANRLF

GGKSITFNETYQDISEVVYGAKLQPLDFKGNAEQSRLTINQWISNKTEGRITDVIPPQAI

NEFTVLVLVNTIYFKGLWKSKFSPENTRKELFYKADGESCSVLMMYQESKFRYRRVAEST

QVLELPFKGDDITMVLILPKLEKTLAKVEQELTPDMLQEWLDELTETLLVVHMPRFRIED

SFSVKEQLQDMGLEDLFSPEKSRLPGIVAEGRSDLYVSDAFHKAFLEVNEEGSEAAASTV

ISIAGRSLNSDRVTFKANRPILVLIREVALNTIIFMGRVANPCVD

>sp|Q0VCU1|ACOC_BOVIN Cytoplasmic aconitate hydratase OS=Bos taurus OX=9913 GN=ACO1 PE=2 SV=1

MSNPFAHLVEPLDPAQPGKKFFNLNKLEDSRYGSLPFSIRVLLEAAIRNCDQFLVKKNDV

ENILNWKVMQHKNIEVPFKPARVILQDFTGVPAVVDFAAMRDAVKKLGGNPEKINPICPA

DLVIDHSIQVDFNRRADSLKKNQDLEFERNKERFEFLKWGSQAFHNMRIIPPGSGIIHQV

NLEYLARVVFDQDGYYYPDSLVGTDSHTTMIDGLGVLGWGVGGIEAEAVMLGQPISMVLP

QVIGYRLVGNPHPLVTSTDIVLTITKHLRQVGVVGKFVEFFGPGVAQLSIADRATIANMC

PEYGATAAFFPVDEVSIKYLVQTGRDKEKVKHIKQYLQAVGMFRDFSDSSQDPDFAQVVE

LDLKTVVPCCSGPKRPQDKVAVSDMKKDFESCLGAKQGFKGFQVAPDHHNDHKTFIYNNS

KFTLAHGSVVIAAITSCTNTSNPSVMLGAGLLAKKAVDAGLSVKPYIKTSLSPGSGVVTY

YLRESGVMPYLSQLGFDVVGYGCMTCIGNSGPLPEAVVEAIVQGDLVAVGVLSGNRNFEG

RVHPNTRANYLASPPLVIAYAIAGTIRIDFEKEPLGVNAKGQQVFLKDIWPTRDEIQAVE

RQYVIPGMFKEVYQKIETVNESWNALAAPSDKLYCWNPKSTYIKSPPFFEDLTLDLQPPK

SIVDAYVLLNLGDSVTTDHISPAGNIARNSPAARYLTNRGLTPREFNSYGSRRGNDAIMA

RGTFANIRLLNKFLNKQAPQTIHLPSGEILDVFDAAERYQQAGLPLIVLAGKEYGSGSSR

DWAAKGPFLLGIRAVLAESYERIHRSNLVGMGVIPLEYLPGENADTLGLTGRERYTISIP

ETLKPRMKVQIKLDTGKTFQAVMRFDTDVELTYFHNGGILNYMIRKMTK

>sp|P56652|ITIH3_BOVIN Inter-alpha-trypsin inhibitor heavy chain H3 OS=Bos taurus OX=9913 GN=ITIH3 PE=1 SV=2

MALAQWPYLILALLSGLAVSGFPRNPSLLLGKRSLPGRAVDGIEVYSTKVNCKVTSRFAH

NVVTTRAVNHANTAKEVSFDVELPKTAFITNFTLTIDGVTYPGKVKEKEVAKKQYEKAVS

QGKTAGLVKASGRKLEKFTVSVNVAAGSKVTFELTYEELLKRHKGKYEMYLKVQPKQLVK

HFEITVDIFEPQGISTLDAEASFITNDLLGSALTKSFSGKKGHVSFKPSLDQQRSCPTCT

DSLLKGDFIITYDVNRESPANVQIVNGYFVHFFAPQGLPVVPKSVVFVIDVSGSMHGRKM

EQTKDALLKILEDVKQDDYLNFILFSGDVTTWKDSLVPATPENIQEASKFVMDIQDRGMT

NINDALLRGISMLNKAREEHTVPERSTSIIIMLTDGDANVGESRPEKIQENVRNAIGGKF

PLYNLGFGNNLNYNFLENMALENHGLARRIYEDSDANLQLQGFYEEVANPLLTGVEVEYP

QNAILDLTQNSYQHFYDGSEIVVAGRLADEDMNSFKAAVKGHGAINDLTFTEEVDMKEME

KALQERDYIFGDYIERLWAYLTIEQLLDKRKNAQGEEKEILTAQALELSLKYHFVTPLTS

MVVTKPEDNENQTAIANKPGEGPLDAEEVPSMAYLTSYQAPQTPYYYVDGDPHFIIQIPE

KDDAICFNIDEDPGTVLRLIQDPVTGLTVNGQIIGEKTGRSDSQTRRTYFGKLGIASAQM

DFRIEVTRENITLWNGDSLSTFSWLDTVMVTQDGLSVMINRKKNMVVSFGDGVTFVVVLH

QVWKKEPAHHDFLGFYVVNSRGMSAQTHGLLGQFFHPFDFQVSDVHPGSDPTKPDATMVV

KNHQLTVTRGSQKDYRKDISVGRNVACWFVHNNGQGLIDGIHRDYIVPNLF

>sp|P34955|A1AT_BOVIN Alpha-1-antiproteinase OS=Bos taurus OX=9913 GN=SERPINA1 PE=1 SV=1

MALSITRGLLLLAALCCLAPISLAGVLQGHAVQETDDTSHQEAACHKIAPNLANFAFSIY

HHLAHQSNTSNIFFSPVSIASAFAMLSLGAKGNTHTEILKGLGFNLTELAEAEIHKGFQH

LLHTLNQPNHQLQLTTGNGLFINESAKLVDTFLEDVKNLYHSEAFSINFRDAEEAKKKIN

DYVEKGSHGKIVELVKVLDPNTVFALVNYISFKGKWEKPFEMKHTTERDFHVDEQTTVKV

PMMNRLGMFDLHYCDKLASWVLLLDYVGNVTACFILPDLGKLQQLEDKLNNELLAKFLEK

KYASSANLHLPKLSISETYDLKSVLGDVGITEVFSDRADLSGITKEQPLKVSKALHKAAL

TIDEKGTEAVGSTFLEAIPMSLPPDVEFNRPFLCILYDRNTKSPLFVGKVVNPTQA

>sp|P81187|CFAB_BOVIN Complement factor B OS=Bos taurus OX=9913 GN=CFB PE=1 SV=2

MGIGHNPRLCLVPLILGLLCGGVGMTPLPEAGPQSPCSLEGVEIKGGSFRLLKAGQVLEY

LCPSGFYPYPTQIRTCRSTGSWSTLQTQDRKIVKRAECKAIRCPRPQDFENGEYWPRAAY

YNLSDEISFRCYDGYTLRGSANRTCQGNGRWDGETAICDDGATYCPNPGIPLGTRKVGSQ

YRLEDRVTYYCNRGLTLRGSEQRTCLEGGSWSGTEPSCQDSFMYDTPAEVAEAFLSSLTE

TIEGVDAEDGHSPGEQQKRKIVLDPSGSMNIYLVLDGSDSVGAHNFTGAKNCLRDFIEKV

ASYGVKPKYGLVTYATEPKVLIRVSDPKSSEADWVTDQLNQINYADHKLKAGTNTKRALL

EVYNMMSREVNQFKETWNRTRHVIIIMTDGLHNMGGDPVTVIHDIRYLLDIGRNRKNPRE

DYLDIYVFGVGPLVNQENINALASKKDKEKHVFKLQGMENLEDVFVQMLDESRTLGLCGM

VWEHKDGTAYHKQPWQAKISVTRPSKGHESCMGAIVSEYFVLTAAHCFTVDDEKHSIKVS

LGGQRKEWEVKEILFHPKYDLNAKKAKGIPEFYDYDVALVRLKEKLKYETTIRPICLPCT

EGSIQALRLPRSTTCQQQMQELLPAKDIEALFVSESKKTLTRKAVYIKNGDKKASCERDA

LRAPGYEKVKDVSEVVTPRFLCTGGVDPYADPNTCKGDSGGPLIIHKRSRFIQVGVISWG

VVDVCKRPQQVPGYARDFHINLYQVLPWLKEKLQNEDLGFL

>sp|P28800|A2AP_BOVIN Alpha-2-antiplasmin OS=Bos taurus OX=9913 GN=SERPINF2 PE=1 SV=2

MALLWGLLALILSCLSSLCSAQFSPVSTMEPLDLQLMDGQAQQKLPPLSLLKLDNQEPGG

QIAPKKAPEDCKLSPTPEQTRRLARAMMTFTTDLFSLVAQSSTRPNLILSPLSVALALSH

LALGAQNQTLQRLKEVLHADSGPCLPHLLSRLCQDLGPGAFRLAARMYLQKGFPIKEDFL

EQSEQLFGAKPMSLTGMKGEDLANINRWVKEATEGKIEDFLSDLPDDTVLLLLNAIHFQG

FWRSKFDPNLTQRGAFHLDEQFTVPVDMMQALTYPLHWFLLEQPEIQVAHFPFKNNMSFV

VLMPTRFEWNASQVLANLTWDILHQPSLSERPTKVQLPKLHLKYQLDLVATLSQLGLQEL

FQAPDLRGISDERLVVSSVQHQSALELSEAGVQAAAATSTAMSRMSLSSFIVNRPFLFFI

LEDSTSLPLFVGSVRNPNPGAQPERKEQQDSPDGKDSFQDHKGLPRGDKPFDPDLKLGPP

SEEDYAQPSSPK

>sp|Q3SZ57|FETA_BOVIN Alpha-fetoprotein OS=Bos taurus OX=9913 GN=AFP PE=2 SV=1

MKWVVSFFLLFLLNFSDSRTMHKNAYGIDSILDSSPCSSGTNLVGLATIFFAQSVQGATY

EEVSQMVKDVLTIIEKPTGSKQPAGCLENQVSAFLEEICREKEIPEKYGLSDCCSRTGEE

RHDCFLAHKKAAPASIPPFPVLEPVTSCKSYKENRELFINRYIYEIARRHPVLYAPTILS

VANQYNKIIPHCCKAENATECFETKVTSITKELRESSLLNQHICAVMGKFGPRTFRAITV

TKVSQKFPKANFTEIQKLVMDVAHIHEECCKGNVLECLQDGERVMSYICSQQDILSRQIA

ECCKLPTTLELGHCIIHAENDDKPEGLSPNVNRFLGDRDFNQLSSRDKDLSMARFTYEYS

RRHTKLAVPIILRVAKGYQELLEKCSQSENPSECQDKGEEELEKYIQESQALAKRSCGLF

QKLGEYYLQNAFLVAYTKKAPQLTSPELMALTRKMANAGAICCHLSEDKQLACGEGVADL

IIGHLCIRHEENPINPGVDQCCTSSYSNRRPCFSSLVVDETYVPPPFSDDKFIFHKDLCQ

VQGVPLQTMKQQFLINLVKQKPQITEEQLETVVADFSGLLEKCCQSQEQEVCFTEEGPAL

ISKTRAALGV

>sp|P63258|ACTG_BOVIN Actin, cytoplasmic 2 OS=Bos taurus OX=9913 GN=ACTG1 PE=1 SV=1

MEEEIAALVIDNGSGMCKAGFAGDDAPRAVFPSIVGRPRHQGVMVGMGQKDSYVGDEAQS

KRGILTLKYPIEHGIVTNWDDMEKIWHHTFYNELRVAPEEHPVLLTEAPLNPKANREKMT

QIMFETFNTPAMYVAIQAVLSLYASGRTTGIVMDSGDGVTHTVPIYEGYALPHAILRLDL

AGRDLTDYLMKILTERGYSFTTTAEREIVRDIKEKLCYVALDFEQEMATAASSSSLEKSY

ELPDGQVITIGNERFRCPEALFQPSFLGMESCGIHETTFNSIMKCDVDIRKDLYANTVLS

GGTTMYPGIADRMQKEITALAPSTMKIKIIAPPERKYSVWIGGSILASLSTFQQMWISKQ

EYDESGPSIVHRKCF

>sp|Q3MHL4|SAHH_BOVIN Adenosylhomocysteinase OS=Bos taurus OX=9913 GN=AHCY PE=2 SV=3

MSDKLPYKVADISLAAWGRKALDLAENEMPGLMHMREMYSASKPLKGARIAGCLHMTVET

AVLIETLVALGAEVRWSSCNIFSTQDHAAAAIAKAGIPVYAWKGETDEEYLWCIEQTLYF

KDGPLNMILDDGGDLTNLIHTKYPQLLSGIRGISEETTTGVHNLYKMMAKGILKVPAINV

NDSVTKSKFDNLYGCRESLIDGIKRATDVMIAGKVAVVAGYGDVGKGCAQALRGFGARVI

ITEIDPINALQAAMEGYEVTTMDEACQEGNIFVTTTGCTDIILGQHFEQMKDDAIVCNIG

HFDVEIDVKWLNENAVEKVNIKPQVDRYLLKNGRRIILLAEGRLVNLGCAMGHPSFVMSN

SFTNQVLAQIELWTHPDKYPVGVHFLPKKLDEAVAEAHLGKLNVKLTKLTEKQAQYLGVS

REGPFKPDHYRY

>sp|Q95121|PEDF_BOVIN Pigment epithelium-derived factor OS=Bos taurus OX=9913 GN=SERPINF1 PE=1 SV=1

MQALVLLLWTGALLGFGRCQNAGQEAGSLTPESTGAPVEEEDPFFKVPVNKLAAAVSNFG

YDLYRVRSGESPTANVLLSPLSVATALSALSLGAEQRTESNIHRALYYDLISNPDIHGTY

KDLLASVTAPQKNLKSASRIIFERKLRIKASFIPPLEKSYGTRPRILTGNSRVDLQEINN

WVQAQMKGKVARSTREMPSEISIFLLGVAYFKGQWVTKFDSRKTSLEDFYLDEERTVKVP

MMSDPQAVLRYGLDSDLNCKIAQLPLTGSTSIIFFLPQKVTQNLTLIEESLTSEFIHDID

RELKTVQAVLTIPKLKLSYEGELTKSVQELKLQSLFDAPDFSKITGKPIKLTQVEHRVGF

EWNEDGAGTNSSPGVQPARLTFPLDYHLNQPFIFVLRDTDTGALLFIGKILDPRGT

>sp|Q32LP0|URP2_BOVIN Fermitin family homolog 3 OS=Bos taurus OX=9913 GN=FERMT3 PE=2 SV=1

MAGMKTATGDYIDSSWELRVFIGEEDPEAESLTLRVTGESHIGGVLLKIVEEIKRKQDWS

DHAIWWEQKRQWLLQTHWTLDKYGILADARLFFGPQHRPVILRLPNRRALRLRASFSQPL

FQAMVAICRLLSIRHPEEMSLLRAPEKEKKKKKEKEPEEEVYDLTKVVLVGGVAPASFRG

MPAHFSDSAQTEACYHMLSRPQPPPDPLLLQRLPRPSSLLDKTQLHSRWLDSSRCLMQQG

IKAGDTLWLRFKYYSFFDLDPKTDPVRLTQLYEQARWDLLLEEIDCTEEEMMVFAALQYH

INKLSQSGEVDEPAGTDSGLDDLDLALSNLEVKLEGSAPTDMLDSLTTIPELKDHLRIFR

PRKLTLKGYRQHWVVFKETTLSYYKSQDEAPGEPIQQLNLKGCEVVPDVNVSGQKFCIKL

LVPSPEGMSEIYLRCQDEQQYARWMAGCRLASKGRTMADSSYSSEVQAILAFLSLQRTGG

GGGGSGNHPQGPDASAEGLNPYGLVAPRFQRKFKAKQLTPRILEAHQNVAQLSLSEAQLR

FIQAWQSLPDFGISYVVVRFKGSRKDEILGIANNRLIRIDLSVGDVVKTWRFSNMRQWNV

NWDIRQVAIEFDEHINVAFSCVSASCRIVHEYIGGYIFLSTRERARGEELDEDLFLQLTG

GHEAF

>sp|Q27975|HS71A_BOVIN Heat shock 70 kDa protein 1A OS=Bos taurus OX=9913 GN=HSPA1A PE=1 SV=2

MAKNMAIGIDLGTTYSCVGVFQHGKVEIIANDQGNRTTPSYVAFTDTERLIGDAAKNQVA

LNPQNTVFDAKRLIGRKFGDPVVQSDMKHWPFRVINDGDKPKVQVSYKGETKAFYPEEIS

SMVLTKMKEIAEAYLGHPVTNAVITVPAYFNDSQRQATKDAGVIAGLNVLRIINEPTAAA

IAYGLDRTGKGERNVLIFDLGGGTFDVSILTIDDGIFEVKATAGDTHLGGEDFDNRLVNH

FVEEFKRKHKKDISQNKRAVRRLRTACERAKRTLSSSTQASLEIDSLFEGIDFYTSITRA

RFEELCSDLFRSTLEPVEKALRDAKLDKAQIHDLVLVGGSTRIPKVQKLLQDFFNGRDLN

KSINPDEAVAYGAAVQAAILMGDKSENVQDLLLLDVAPLSLGLETAGGVMTALIKRNSTI

PTKQTQIFTTYSDNQPGVLIQVYEGERAMTRDNNLLGRFELSGIPPAPRGVPQIEVTFDI

DANGILNVTATDKSTGKANKITITNDKGRLSKEEIERMVQEAEKYKAEDEVQRERVSAKN

ALESYAFNMKSAVEDEGLKGKISEADKKKVLDKCQEVISWLDANTLAEKDEFEHKRKELE

QVCNPIISRLYQGAGGPGAGGFGAQGPKGGSGSGPTIEEVD

>sp|P17697|CLUS_BOVIN Clusterin OS=Bos taurus OX=9913 GN=CLU PE=1 SV=1

MKTLLLLMGLLLSWESGWAISDKELQEMSTEGSKYVNKEIKNALKEVKQIKTQIEQTNEE

RKLLLSSLEEAKKKKEDALNDTRDSENKLKASQGVCNETMTALWEECKPCLKQTCMKFYA

RVCRSGSGLVGHQLEEFLNQSSPFYFWINGDRIDSLMENDREQSHVMDVMEDSFTRASSI

MDELFQDRFFLRRPQDTQYYSPFSSFPRGSLFFNPKSRFARNVMPFPLLEPFNFHDVFQP

FYDMIHQAQQAMDAHLQRTPYHFPTMEFTENNDRTVCKEIRHNSTGCLRMKDQCEKCQEI

LEVDCSASNPTQTLLRQQLNASLQLAEKFSRLYDQLLQSYQQKMLNTSALLKQLNEQFTW

VSQLANLTQSDDQHYLQVFTVNSHNSDPSIPSGLTKVIVKLFNSFPITVTVPQEVSSPNF

MENVAEKALQQYRRKSQEE

>sp|Q3SX14|GELS_BOVIN Gelsolin OS=Bos taurus OX=9913 GN=GSN PE=2 SV=1

MVVEHPEFLKAGKEPGLQIWRVEKFDLVPVPPNLYGDFFTGDAYVILKTVQLRNGNLQYD

LHYWLGNECSQDESGAAAIFTVQLDDYLNGRAVQHREVQGFESATFLGYFKSGLKYKKGG

VASGFKHVVPNEVVVQRLFQVKGRRVVRATEVPVSWESFNNGDCFILDLGNDIYQWCGSS

SNRFERLKATQVSKGIRDNERSGRARVHVSEEGAEPEAMLEVLGPKPALPAGTEDTAKED

AANRKLAKLYKVSNGAGTMSVSLVADENPFAQGALRSEDCFILDHGKDGKIFVWKGRQAN

TEERKAALKTASDFISKMDYPRQTQVSVLPEGGETPLFKQFFKNWRDPDQTDGPGLSYLS

SHIANVERVPFDAATLHTSTAMAAQHGMDDDGRGQKQIWRIEGSDKVPVDPATYGQFYGG

DSYIILYNYRHGGRQGQIIYNWQGAQSTQDEVAASAILTAQLDEELGGTPVRSRVVQGKE

PAHLMSLFGGKPMIIYRGGTSREGGQTAPASTRLFQVRASSSGATRAVEVMPKAGALNSN

DAFVLKTPSAAYLWVGAGASEAEKTGALELLRVLRAQPVQVAEGSEPDSFWEALGGKAAY

RTSPRLKDKKMDAHPPRLFACSNKIGRFVIEEVPGELMQEDLATDDVMLLDTWDQVFVWV

GKDSQEEEKTEALTSAKRYIETDPANRDRRTPITVVKQGFEPPSFVGWFLGWDDNYWSVD

PLDRALAELAA

>sp|Q27965|HS71B_BOVIN Heat shock 70 kDa protein 1B OS=Bos taurus OX=9913 GN=HSPA1B PE=2 SV=1

MAKNTAIGIDLGTTYSCVGVFQHGKVEIIANDQGNRTTPSYVAFTDTERLIGDAAKNQVA

LNPQNTVFDAKRLIGRKFGDPVVQSDMKHWPFRVINDGDKPKVQVSYKGETKAFYPEEIS

SMVLTKMKEIAEAYLGHPVTNAVITVPAYFNDSQRQATKDAGVIAGLNVLRIINEPTAAA

IAYGLDRTGKGERNVLIFDLGGGTFDVSILTIDDGIFEVKATAGDTHLGGEDFDNRLVNH

FVEEFKRKHKKDISQNKRAVRRLRTACERAKRTLSSSTQASLEIDSLFEGIDFYTSITRA

RFEELCSDLFRSTLEPVEKALRDAKLDKAQIHDLVLVGGSTRIPKVQKLLQDFFNGRDLN

KSINPDEAVAYGAAVQAAILMGDKSENVQDLLLLDVAPLSLGLETAGGVMTALIKRNSTI

PTKQTQIFTTYSDNQPGVLIQVYEGERAMTRDNNLLGRFELSGIPPAPRGVPQIEVTFDI

DANGILNVTATDKSTGKANKITITNDKGRLSKEEIERMVQEAEKYKAEDEVQRERVSAKN

ALESYAFNMKSAVEDEGLKGKISEADKKKVLDKCQEVISWLDANTLAEKDEFEHKRKELE

QVCNPIISRLYQGAGGPGAGGFGAQGPKGGSGSGPTIEEVD

>sp|Q3MHM5|TBB4B_BOVIN Tubulin beta-4B chain OS=Bos taurus OX=9913 GN=TUBB4B PE=2 SV=1

MREIVHLQAGQCGNQIGAKFWEVISDEHGIDPTGTYHGDSDLQLERINVYYNEATGGKYV

PRAVLVDLEPGTMDSVRSGPFGQIFRPDNFVFGQSGAGNNWAKGHYTEGAELVDSVLDVV

RKEAESCDCLQGFQLTHSLGGGTGSGMGTLLISKIREEYPDRIMNTFSVVPSPKVSDTVV

EPYNATLSVHQLVENTDETYCIDNEALYDICFRTLKLTTPTYGDLNHLVSATMSGVTTCL

RFPGQLNADLRKLAVNMVPFPRLHFFMPGFAPLTSRGSQQYRALTVPELTQQMFDAKNMM

AACDPRHGRYLTVAAVFRGRMSMKEVDEQMLNVQNKNSSYFVEWIPNNVKTAVCDIPPRG

LKMSATFIGNSTAIQELFKRISEQFTAMFRRKAFLHWYTGEGMDEMEFTEAESNMNDLVS

EYQQYQDATAEEEGEFEEEAEEEVA

>sp|A3KMV5|UBA1_BOVIN Ubiquitin-like modifier-activating enzyme 1 OS=Bos taurus OX=9913 GN=UBA1 PE=2 SV=1

MSSSPLSKKRRVSGPDPKPGSNCSPAHSVLSEVPSVPANGMAKNVSDADIDEGLYSRQLY

VLGHEAMKRLQTSSVLVSGLRGLGVEIAKNIILGGVKAVTLHDQGTAQWADLSSQFYLRE

EDIGKNRAEVSQPRLAELNSYVPVSAYTGPLVEDFLSDFQVVVLTNSPLEDQLRVGEFCH

SHGIKLVVADTRGLFGQLFCDFGEEMILTDSNGEQPLSAMVSMVTKDNPGVVTCLDEARH

GFESGDFVSFSEVQGMIELNGSQPMEIKVLGPYTFSICDTSNFSDYIRGGIVSQVKVPKK

ISFKSLPASLAEPDFVMTDFAKYSRPAQLHIGFQALHHFCAQHGRSPRPHNEEDAAELVT

IAQAVNARSLPAVQQGSLDEDLIRKLAYVAAGDLAPINAFIGGLAAQEVMKACSGKFMPI

MQWLYFDALECLPEDKEALTEDKCLPRQNRYDGQVAVFGSDLQERLGKQKYFLVGAGAIG

CELLKNFAMIGLGCAEDGEIVVTDMDTIEKSNLNRQFLFRPWDVTKLKSDTAAAAVRQMN

PHIRVTSHQNRVGPDTERIYDDDFFQNLDGVTNALDNVDARMYMDRRCVYYRKPLLESGT

LGTKGNVQVVIPFLTESYSSSQDPPEKSIPICTLKNFPNAIEHTLQWARDEFEGLFKQPA

ENVNQYLTDPKFVERTLRLAGTQPLEVLEAVQRSLVLQRPQTWADCVTWACHHWHTQYSN

NIRQLLHNFPPDQLTSSGAPFWSGPKRCPHPLTFDVSNPLHLDYVIAAANLFAQTYGLTG

SQDRAAVATLLQSVQVPEFTPKSGVKIHVSDQELQSANASVDDSRLEELKATLPSPEKLP

GFKMYPIDFEKDDDTNFHMDFIVAASNLRAENYDIPPADRHKSKLIAGKIIPAIATTTAA

VVGLVCLELYKVVQGHRQLNSYKNGFLNLALPFFGFSEPLAAPRHQYYNQEWTLWDRFEV

QGLQPNGEEMTLKQFLDYFKTEHKLEITMLSQGVSMLYSFFMPAAKLKERLDQPMTEIVS

RVSKRKLGRHVRALVLELCCNDESGEDVEVPYVRYTIR

>sp|Q0VCK0|PUR9_BOVIN Bifunctional purine biosynthesis protein ATIC OS=Bos taurus OX=9913 GN=ATIC PE=2 SV=1

MAPGQLALFSVSDKNGLVEFARNLASVGLNLIASGGTAKALRDAGLAVRDVSELTGFPEM

LGGRVKTLHPAVHAGILARDIPEDSADMAKLDFNLIRVVVCNLYPFGKTVASPGVTVEEA

VEHIDIGGVTLLRAAAKNHARVTVVCEPEDYAAVASEMQDSDSKDTSLETRRQLALKAFT

HTAQYDEAISDYFRKEYSKGVSQMPLRYGMNPHQTPAQLYTLKPKLPITVLNGAPGFINL

CDALNAWQLVKELKEALGLPAAASFKHVSPAGAAVGIPLSEDEANVCMVYDLYKTLTPVA

TAYARARGADRMSSFGDFVALSDVCDVPTAKIISREVSDGIIAPGYENEALKILSKKKNG

NYCVLQMDQSYIPDENEVRTLFGLRLSQKRNNSVVNRSLFSNIVTKNKDLPESALRDLIV

ATIAVKYTQSNSVCYAKNGQVIGIGAGQQSRIHCTRLAGDKANCWWLRHHPQVLSMKFKT

GVKRAEISNAIDQYVTGTIGEGEDLIKWKALFEEVPELLTETEKKEWIDKLNEVSISSDA

FFPFRDNVDRAKRSGVAYIAAPSGSAADKVVIEACDELGIILAHTNLRLFHH

>sp|Q92176|COR1A_BOVIN Coronin-1A OS=Bos taurus OX=9913 GN=CORO1A PE=1 SV=3

MSRQVVRSSKFRHVFGQPAKADQCYEDVRVSQNTWDSGFCAVNPKFVALICEASGGGAFL

VLPLGKTGRVDKNVPMVCGHTAPVLDIAWCPHNDNVIASGSEDCSVMVWEIPDGGLTLPL

REPVVTLEGHTKRVGIVAWHPTAQNVLLSAGCDNVILVWDVGTGVAVLTLGSDVHPDTIY

SVDWSRDGALICTSCRDKRVRIIEPRKGTIVAEKDRPHEGTRPVRAVFVSDGKILTTGFS

RMSERQVALWDTKHLEEPLSLQELDTSSGVLLPFFDPDTNIVYLCGKGDSSIRYFEITSE

APFLHYLSMFSSKESQRGMGYMPKRGLEVNKCEIARFYKLHERKCEPIAMTVPRKSDLFQ

EDLYPPTAGPDAALTAEEWLGGRDAGPLLISLKDGYVPPKSRELRVNRGLDTGRKRTTPE

ASGAPSSDAISRLEEEMRKLQATVQELQKRLDRLEETVQAK

>sp|Q76LV1|HS90B_BOVIN Heat shock protein HSP 90-beta OS=Bos taurus OX=9913 GN=HSP90AB1 PE=2 SV=3

MPEEVHHGEEEVETFAFQAEIAQLMSLIINTFYSNKEIFLRELISNASDALDKIRYESLT

DPSKLDSGKELKIDIIPNPQERTLTLVDTGIGMTKADLVNNLGTIAKSGTKAFMEALQAG

ADISMIGQFGVGFYSAYLVAEKVVVITKHNDDEQYAWESSAGGSFTVRADHGEPIGRGTK

VILHLKEDQTEYLEERRVKEVVKKHSQFIGYPITLYLEKEREKEISDDEAEEEKGEKEEE

DKDDEEKPKIEDVGSDEEDDSGKDKKKKTKKIKEKYIDQEELNKTKPIWTRNPDDITQEE

YGEFYKSLTNDWEDHLAVKHFSVEGQLEFRALLFIPRRAPFDLFENKKKKNNIKLYVRRV

FIMDSCDELIPEYLNFIRGVVDSEDLPLNISREMLQQSKILKVIRKNIVKKCLELFSELA

EDKENYKKFYEAFSKNLKLGIHEDSTNRRRLSELLRYHTSQSGDEMTSLSEYVSRMKETQ

KSIYYITGESKEQVANSAFVERVRKRGFEVVYMTEPIDEYCVQQLKEFDGKSLVSVTKEG

LELPEDEEEKKKMEESKAKFENLCKLMKEILDKKVEKVTISNRLVSSPCCIVTSTYGWTA

NMERIMKAQALRDNSTMGYMMAKKHLEINPDHPIVETLRQKAEADKNDKAVKDLVVLLFE

TALLSSGFSLEDPQTHSNRIYRMIKLGLGIDEDEVTAEEPSAAVPDEIPPLEGDEDASRM

EEVD

>sp|P08169|MPRI_BOVIN Cation-independent mannose-6-phosphate receptor OS=Bos taurus OX=9913 GN=IGF2R PE=1 SV=2

MEAAAGRSSHLGPAPAGRPPRCPLLLQLQLLLLLLLLPPGWVPGAAGTQGAEFPELCSYT

WEAVDTKNNMLYKINICGNMGVAQCGPSSAVCMHDLKTDSFHSVGDSLLKTASRSLLEFN

TTVNCKQQNHKIQSSITFLCGKTLGTPEFVTATDCVHYFEWRTTAACKKNIFKANKEVPC

YAFDRELKKHDLNPLIKTSGAYLVDDSDPDTSLFINVCRDIEVLRASSPQVRVCPTGAAA

CLVRGDRAFDVGRPQEGLKLVSNDRLVLSYVKEGAGQPDFCDGHSPAVTITFVCPSERRE

GTIPKLTAKSNCRFEIEWVTEYACHRDYLESRSCSLSSAQHDVAVDLQPLSRVEASDSLF

YTSEADEYTYYLSICGGSQAPICNKKDAAVCQVKKADSTQVKVAGRPQNLTLRYSDGDLT

LIYFGGEECSSGFQRMSVINFECNQTAGNNGRGAPVFTGEVDCTYFFTWDTKYACVHEKE

ALLCGVSDGKQRFDLSALARHSELEQNWEAVDGSQREAEKKHFFINICHRVLQTGQARGC

PEDAAVCAVDKNGSKNLGRFISSPTREKGNIQLSYSDGDECGGGQKIITNITLMCKPGDL

ESAPVLTTSRADGCFYEFEWRTAAACVLSRTEGDNCTVFDSQAGFSFDLTPLTKKDAYKV

ETDKYEFHINVCGPVSVGACPPDSGACQVSRSDRKSWNLGRSNAKLSYYDGMIQLTYRDG

TPYNNEKRTPRATLITFLCDRDAGVGFPEYQEEDNSTYNFRWYTSYACPEEPLECIVTDP

VTLDQYDLSRLAKSEGGPGGNWYSLDNGGARSTWRKYYINVCRPLNPVPGCDRYASACQM

KYQGEQGSYSETVSISNLGVAKTGPMVEDSGSLLLEYVNGSACTTSDQRRTTYTTRIHLV

CSTGSLYTHPIFSLNWECVVSFLWNTAAACPIRITTDIDQVCSIKDPNSGYVFDLNPLNN

SRGYVVLGIGKTFLFNVCGDMPACGTLDGKPASGCEAEVQMDDMKTLKPGRLVGLEKSLQ

LSTEGFITLNYTGLPSHPNGRADAFIIRFVCNDDVYPGTPKFLHQDIDSSLGIRDTFFEF

ETALACVPSPVDCQVTDPAGNEYDLSGLSKARKPWTAVDTFDEGKKRTFYLSVCTPLPYI

PGCHGTAVGCCLVTEDSKLNLGVVQISPQVGANGSLSLVYVNGDKCKNQRFSTRINLECA

HTTGSPTFQLQNDCEYVFLWRTVEACPVVRAEGDYCEVRDPRHGNLYNLIPLGLNDTVVR

AGEYTYYFRVCGELTSGVCPTSDKSKVISSCQEKRGPQGFQKVAGLFNQKLTYENGVLKM

NYTGGDTCHKVYQRSTTIFFYCDRSTQAPVFLQETSDCSYLFEWRTQYACPPYDLTECSF

KNEAGETYDLSSLSRYSDNWEAVTGTGSTEHYLINVCKSLSPQAGSDPCPPEAAVCLLGG

PKPVNLGRVRDSPQWSQGLTLLKYVDGDLCPDQIRKKSTTIRFTCSESHVNSRPMFISAV

EDCEYTFSWPTAAACAVKSNVHDDCQVTNPATGHLFDLSSLSGRAGFTAAYSEKGLVYLS

VCGDNENCANGVGACFGQTRISVGKASKRLTYVDQVLQLVYEGGSPCPSKTGLSYKSVIS

FVCRPEVGPTNRPMLISLDKRTCTLFFSWHTPLACEQTTECSVRNGSSLIDLSPLIHRTG

GYEAYDESEDDGSDTSPDFYINICQPLNPMHGLACPAGTAVCKVPVDGPPIDIGRVAGPP

ILNPIANEVYLNFESSTPCLADRHFNYTSLITFHCKRGVSMGTPKLLRTSVCDFVFEWET

PLVCPDEVKTDGCSLTDEQLYYSFNLSSLSKSTFKVTRGPHTYSVGVCTAAAGLDEGGCK

DGAVCLLSGSKGASFGRLASMKLDYRHQDEAVILSYANGDTCPPETEDGEPCVFPFVFNG

KSYEECVVESRARLWCATTANYDRDHEWGFCKHSTSHRTSVIIFKCDEDADVGRPQVFSE

VRGCEVTFEWKTKVVCPPKKMECKFVQKHRTYDLRLLSSLTGSWSFVHNGASYYINLCQK

IYKGPQDCSERASVCKKSTSGEVQVLGLVHTQKLDVVDDRVIVTYSKGHYCGDNKTASAV

IELTCAKTVGRPSFTRFDVDSCTYHFSWDSRAACAVKPQEVQMVNGTITNPANGRSFSLG

DIYFKRFSASGDVRTNGDRYIYEIQLSSITGSSSPACSGASICQRKANDQHFSRKVGTSN

QTRYYVQDGDLDVVFTSSSKCGKDKTKSVSSTIFFHCDPLVKDGIPEFSHETADCQYLFS

WHTSAVCPLGAGFDEEIAGDDAQEHKGLSERSQAVGAVLSLLLVALTACLLTLLLYKKER

REMVMSRLTNCCRRSANVSYKYSKVNKEEEADENETEWLMEEIQPPAPRPGKEGQENGHV

AAKSVRAADTLSALHGDEQDSEDEVLTLPEVKVRPPGRAPGAEGGPPLRPLPRKAPPPLR

ADDRVGLVRGEPARRGRPRAAATPISTFHDDSDEDLLHV

>sp|P60712|ACTB_BOVIN Actin, cytoplasmic 1 OS=Bos taurus OX=9913 GN=ACTB PE=1 SV=1

MDDDIAALVVDNGSGMCKAGFAGDDAPRAVFPSIVGRPRHQGVMVGMGQKDSYVGDEAQS

KRGILTLKYPIEHGIVTNWDDMEKIWHHTFYNELRVAPEEHPVLLTEAPLNPKANREKMT

QIMFETFNTPAMYVAIQAVLSLYASGRTTGIVMDSGDGVTHTVPIYEGYALPHAILRLDL

AGRDLTDYLMKILTERGYSFTTTAEREIVRDIKEKLCYVALDFEQEMATAASSSSLEKSY

ELPDGQVITIGNERFRCPEALFQPSFLGMESCGIHETTFNSIMKCDVDIRKDLYANTVLS

GGTTMYPGIADRMQKEITALAPSTMKIKIIAPPERKYSVWIGGSILASLSTFQQMWISKQ

EYDESGPSIVHRKCF

>sp|Q3T0P6|PGK1_BOVIN Phosphoglycerate kinase 1 OS=Bos taurus OX=9913 GN=PGK1 PE=2 SV=3

MSLSNKLTLDKLDVKGKRVVMRVDFNVPMKNNQITNNQRIKAAVPSIKYCLDSGAKSVVL

MSHLGRPDGVPMPDKYSLQPVAVELKSLLGKDVLFLKDCVGPEVEKACADPAAGSVILLE

NLRFHVEEEGKGKDASGNKVKAEPTKIEAFRASLSKLGDVYVNDAFGTAHRAHSSMVGVN

LPKKAGGFLMKKELNYFAKALESPERPFLAILGGAKVADKIQLISNMLDKVNEMIIGGGM

AFTFLKVLNNMEIGTSLFDEEGSKIVKDLMSKADKNGVKITLPVDFVTADKFDENAKTGQ

ATVASGIPAGWMGLDCGPESSKKYAEAVARAKQIVWNGPVGVFEWEAFARGTKALMDEVV

KATSRGCITIIGGGDTATCCAKWNTEDKVSHVSTGGGASLELLEGKVLPGVDALSSV

>sp|Q2KJD0|TBB5_BOVIN Tubulin beta-5 chain OS=Bos taurus OX=9913 GN=TUBB5 PE=2 SV=1

MREIVHIQAGQCGNQIGAKFWEVISDEHGIDPTGTYHGDSDLQLDRISVYYNEATGGKYV

PRAILVDLEPGTMDSVRSGPFGQIFRPDNFVFGQSGAGNNWAKGHYTEGAELVDSVLDVV

RKEAESCDCLQGFQLTHSLGGGTGSGMGTLLISKIREEYPDRIMNTFSVVPSPKVSDTVV

EPYNATLSVHQLVENTDETYCIDNEALYDICFRTLKLTTPTYGDLNHLVSATMSGVTTCL

RFPGQLNADLRKLAVNMVPFPRLHFFMPGFAPLTSRGSQQYRALTVPELTQQVFDAKNMM

AACDPRHGRYLTVAAVFRGRMSMKEVDEQMLNVQNKNSSYFVEWIPNNVKTAVCDIPPRG

LKMAVTFIGNSTAIQELFKRISEQFTAMFRRKAFLHWYTGEGMDEMEFTEAESNMNDLVS

EYQQYQDATAEEEEDFGEEAEEEA

>sp|Q2KJH4|WDR1_BOVIN WD repeat-containing protein 1 OS=Bos taurus OX=9913 GN=WDR1 PE=2 SV=3

MPYEIKKVFASLPQVERGVSKIVGGDPKGNSFLYTNGKCVILRNIDNPAIADIYTEHAHQ

VVVAKYAPSGFYIASGDVSGKLRIWDTTQKEHLLKYEYQPFAGKIKDIAWTEDSKRIAVV

GEGREKFGAVFLWDSGSSVGEITGHNKVINSVDIKQSRPYRLVTGSDDNCAAFFEGPPFK

FKFTISDHGRFVNCVRFSPDGNRFATASADGQIFIYDGKTGEKVCALGGSKAHDGGIYAI

SWSPDSTHLLSASGDKTSKIWDVNVNSVVNTFTMGSNVLDQQLGCLWQKDHLLSISLSGY

INYLDKNNPSKPLRVIKGHSKSIQCLTVHKNGGKSYIYSGSHDGHINYWDSETGENDSFA

GKGHTNQVSRMTVDEHGQLVSCSMDDTVRYTNLTLRDYSGQGVVKLDVQPKCLAVGPGGY

TVVVCIGQIVLLKDQRKCFSIDNPGYEPEVVAVHPGGETVAVGGADGNVRLYSILGTTLK

DEGKLLEAKGPVTDLAFSHDGAFLAVCDASKVVTVFSVADGYSENNVFYGHHAKIVCLAW

SPDNEHFASGGMDMMVYVWTLSDPETRVKIQDAHRLHHVSSLAWLDEHTLVTTSHDASVK

EWTIAY

>sp|Q3ZCJ2|AK1A1_BOVIN Aldo-keto reductase family 1 member A1 OS=Bos taurus OX=9913 GN=AKR1A1 PE=2 SV=1

MAASCILLHTGQKMPLIGLGTWKSDPGQVKAAIKYALSVGYRHIDCAAIYGNETEIGEAL

KENVGPGKLVPREELFVTSKLWNTKHHPEDVEPALRKTLADLQLEYLDLYLMHWPYAFER

GDSPFPKNADGTIRYDSTHYKETWRALEALVAKGLVRALGLSNFNSRQIDDVLSVASVRP

AVLQVECHPYLAQNELIAHCQARNLEVTAYSPLGSSDRAWRDPEEPVLLKEPVVLALAEK

HGRSPAQILLRWQVQRKVSCIPKSVTPSRILENIQVFDFTFSPEEMKQLDALNKNLRFIV

PMLTVDGKRVPRDAGHPLYPFNDPY

>sp|Q29RQ1|CO7_BOVIN Complement component C7 OS=Bos taurus OX=9913 GN=C7 PE=2 SV=1

MKAITLLFLVGFIGEFQVFSSASSPINCQWGSYAPWSECNGCTKTQTRRRSIAVYGQYGG

HSCVGSAFETQPCQPTRGCPTEDGCGERFRCFSGQCISKSLVCNGDSDCEEDGADEDRCE

DAESRPACDKDKPPPNIELTGRGYNALTGQFRNQVLNTKSFGGQCRKVYSGDGRDFYRLS

GNILSYTFQVKINNDFNNEFYNSTWAYVKETSTEHSSSSKGRFLFFSSSSSSHGYSSNTN

ILTKKKSYQLLVLQNTVEVAQFINNNPEFLQLAESFWKELSYLPSLYDYSAYRRLIDQYG

THYLQSGSLGGEYKVIFHMDSEKVKKFDFHSEDKRKCASSHFQFLFTSSKQKCTTMEEVL

KSVSENEGNLLRGVPFVRGGHSGFLAGLSYLDLNNPAGNKRRYSQWAGSVPDLPEVIKQK

LTPLYELVKEVPCASVKKLYLKRAIEEYLDEFDPCHCRPCHNGGMATVQGSQCQCYCKPK

TSGVACEQGVLLGDQAGGVDGGWNCWSSWGPCVQGKKTRSRQCNNPSPSAGGKSCIGETS

ETRQCEDEELEHLRLLEPHCFPLSLVPTKFCSSPPALKDGFVQDEGATFPVGKNIMYTCK

EGYSLVGDPVARCGEDLQWLVGNMHCQKIACVLPALMDGIQSHPHKPFYTIGEKVTISCS

GGRSLEGPSTFLCSSSLKWSPEVKDVQCVQREAPLTPKVPKCQPWEKLQNSRCVCKMPYE

CGSSLGVCARDERSKRILPLTVCKMHVLQCQGRNYTVAGRESCTLPASAEKACGVCPLWE

KCDAEGSECVCRAASECEEAGFSVCVEVNGREQTMTECEAGVLRCRGLSISVTGIRPCVA

EAA

>sp|Q2KJ83|CBPN_BOVIN Carboxypeptidase N catalytic chain OS=Bos taurus OX=9913 GN=CPN1 PE=2 SV=1

MSDLVSIFLHLLLFKLVAPVTFRHHRYDDLVRMLYKVHNECPHITRVYSIGRSVKGRHLY

VLEFSDYPGIHEPLEPEVKYVGNMHGNEVLGRELLLQLSEFLCEEFRNRNQRIVRLVEDT

RIHIMPSMNPDGYEVAAAAQERDISGYLVGRNNANGVDLNRNFPDLNTYIYYNEKNGGPN

HHFPLPDNWKSQVEPETQAVIQWIRSFNFVLSANLHGGAVVANYPYDKSLGHRVRGFRRT

ANTPTPDDKLFQKLAKIYSYAHGWMHQGWNCGDYFPDGITNGASWYSLSKGMQDFNYLHT

NCFEITLELSCDKFPLQGELQREWLGNREALIQFLEQVHQGIKGMVRDENYNNLADAVIS

VGGINHDVTSGAHGDYFRLLLPGTYTVTATAPGFDPETVSVTVGPAEPKLVNFQLKRSTP

QAAPKRRIPNSGHRGRVLPKKVQPRAARKKETMMKQPQRGPA

>sp|Q3SYU2|EF2_BOVIN Elongation factor 2 OS=Bos taurus OX=9913 GN=EEF2 PE=2 SV=3

MVNFTVDQIRAIMDKKANIRNMSVIAHVDHGKSTLTDSLVCKAGIIASARAGETRFTDTR

KDEQERCITIKSTAISLFYELSENDLNFIKQSKDGSGFLINLIDSPGHVDFSSEVTAALR

VTDGALVVVDCVSGVCVQTETVLRQAIAERIKPVLMMNKMDRALLELQLEPEELYQTFQR

IVENVNVIISTYGEGESGPMGNIMIDPVLGTVGFGSGLHGWAFTLKQFAEMYVAKFAAKG

EGQLGPAERAKKVEDMMKKLWGDRYFDPATGKFSKSANSPDGKKLPRTFCQLILDPIFKV

FDAIMNFKKEETAKLIEKLDIKLDSEDKDKEGKPLLKAVMRRWLPAGDALLQMITIHLPS

PVTAQKYRCELLYEGPPDDEAAMGIKSCDPKGPLMMYISKMVPTSDKGRFYAFGRVFSGL

VSTGLKVRIMGPNYTPGKKEDLYLKPIQRTILMMGRYVEPIEDVPCGNIVGLVGVDQFLV

KTGTITTFEHAHNMRVMKFSVSPVVRVAVEAKNPADLPKLVEGLKRLAKSDPMVQCIIEE

SGEHIIAGAGELHLEICLKDLEEDHACIPIKKSDPVVSYRETVSEESNVLCLSKSPNKHN

RLYMKARPFPDGLAEDIDKGEVSARQELKQRARYLAEKYEWDVAEARKIWCFGPDGTGPN

ILTDITKGVQYLNEIKDSVVAGFQWATKEGALCEENMRGVRFDVHDVTLHADAIHRGGGQ

IIPTARRCLYASVLTAQPRLMEPIYLVEIQCPEQVVGGIYGVLNRKRGHVFEETQVAGTP

MFVVKAYLPVNESFGFTADLRSNTGGQAFPQCVFDHWQILPGDPFDNTSRPSQVVAETRK

RKGLKEGIPALDNFLDKL

>sp|Q9XSJ4|ENOA_BOVIN Alpha-enolase OS=Bos taurus OX=9913 GN=ENO1 PE=1 SV=4

MSILKVHAREIFDSRGNPTVEVDLFTAKGLFRAAVPSGASTGIYEALELRDNDKTRYMGK

GVSKAVEHINKTIAPALVSKKLNVVEQEKIDKLMIEMDGTENKSKFGANAILGVSLAVCK

AGAVEKGVPLYRHIADLAGNAEVILPVPAFNVINGGSHAGNKLAMQEFMILPVGAENFRE

AMRIGAEVYHNLKNVIKEKYGKDATNVGDEGGFAPNILENKEALELLKNAIGKAGYSDKV

VIGMDVAASEFYRSGKYDLDFKSPDDPSRYITPDELANLYKSFIRDYPVVSIEDPFDQDD

WEAWQKFTASAGIQVVGDDLTVTNPKRIAKAVSEKSCNCLLLKVNQIGSVTESLQACKLA

QSNGWGVMVSHRSGETEDTFIADLVVGLCTGQIKTVAPCRSERLAKYNQILRIEEELGSK

AKFAGRSFRNPLAK

>sp|P10096|G3P_BOVIN Glyceraldehyde-3-phosphate dehydrogenase OS=Bos taurus OX=9913 GN=GAPDH PE=1 SV=4

MVKVGVNGFGRIGRLVTRAAFNSGKVDIVAINDPFIDLHYMVYMFQYDSTHGKFNGTVKA

ENGKLVINGKAITIFQERDPANIKWGDAGAEYVVESTGVFTTMEKAGAHLKGGAKRVIIS

APSADAPMFVMGVNHEKYNNTLKIVSNASCTTNCLAPLAKVIHDHFGIVEGLMTTVHAIT

ATQKTVDGPSGKLWRDGRGAAQNIIPASTGAAKAVGKVIPELNGKLTGMAFRVPTPNVSV

VDLTCRLEKPAKYDEIKKVVKQASEGPLKGILGYTEDQVVSCDFNSDTHSSTFDAGAGIA

LNDHFVKLISWYDNEFGYSNRVVDLMVHMASKE

>sp|Q3MHN5|VTDB_BOVIN Vitamin D-binding protein OS=Bos taurus OX=9913 GN=GC PE=2 SV=1

MKRILVFLLAVAFVHALERGRDYEKDKVCKDLASLGREDFTSLSMVLYSRKFPSGTFEQI

SHLVNEVVSLTVTCCAEGADPDCYDNRTSALSDKSCESNSPFPVHPGTPECCTHEGLEKK

LCMAALKHQPQEFPTYVEPTNDEICEAFRKDPKDFADRFMYEYSINYGQAPLTLLVGYTK

SYLSMVGSCCTSPNPTVCFLKERLQLKHFSLLTIMTNRICSQYAAYGKEKSRLSHLIKFA

QKVPTAHLEDVLPLAEDITTILSKCCDSVSEDCIKELPEYAVKLCDNLSTKNSKFKDCCQ

EKTPMEIFVCAYFMPASPNPELPDVKLPMNKDVCDEGNTKVLDQYIFELSRKTQIPEVFL

TKILESTLKSLDECCHSESSTACLNAKGPQLTRELSSFIQKGQELCADYSENTFTEYKKK

LAERLRGKFPDATETDLQELVAKRSDFASKCCSVNSPPLYCNSEIDAEINTLQS

>sp|Q9N2I2|IPSP_BOVIN Plasma serine protease inhibitor OS=Bos taurus OX=9913 GN=SERPINA5 PE=1 SV=1

MRLCLFLCLVLLGPRMATLRRSQKKKIQEVPPAVTTAPPGSRDFVFDLYRALAAAAPAQN

IFFSPLSITVSLAMLSLGAQSNTKAQILEGLGIGPGEGSEEELHSASQRLLRELQQPQDS

LQLSLGNALFTKPRLPIQEAFLGAMRTLYLADTFPTNFEDPEGAKKKINDYVAKQTKGKI

VDLIKSLDGTQVMVMVNYIFFKAKWETSFNLKSTHEQDFYVTPETVVRVPMMKQQDQFYY

LLDRNLSCKVVGVPYQGNATAFFILPREGEMEQVENGLKEKTLKKWLRMPMKRRLELYLP

KFSIEGSYQLEEVLPKLGIRDIFTSDADLTGISNHSSIRVSEMVHKAVVEVDESGTQAAA

ATGMVITFKSARLGSQRIVFNRPFLVLIVKNSKHILFLGKVTRP

>sp|P01030|CO4_BOVIN Complement C4 (Fragments) OS=Bos taurus OX=9913 GN=C4 PE=1 SV=2

NVNFQKAIHEKLGQYTSPVAKRCCQDGLTRLPMARTCEQRAARVQQPACREPFLSCCQFA

ESLRKKARTRGQVGLARVGFSVVPIAAAAVSLKVVARGSFDFPVGDAISKILQVEREGAL

HREEMVYELNPLDPLGRTLEIPGNSDPNIIPEGDFKSFVRVTASDPLEALGSEGALSPGG

LASLLRLPQGCAEQTMTLLAPTLAASRYLDKTEQWSMLPPETKDRAVDLIQKGYTRIQEF

RKRDGSYGAWLHRDSSTWLTAFVLKILSLAQDQVXGSAEKLQETATWLLSQQRDDGPFHD

PCPVIHREMQGGLVGSDETVALTAFVVIALHHGLAVLPDKNSRVENSISRANTFLGAKAT

SGLLGSHASAITAYALSLTEAPEDLRRVAHNNLMAMAKDIGDKLYWGSVTTSPSNVLSPT

PAPRSPADPIPQAPAMSIETTAYGLLHLLLWEGKAELADQAASWLTRQGSFQGGFRSTQD

TVVALDALSAYWIASYTAEEKGLNVTLSSLGRSGLKSHVLQLTNHQVHRLEEELQFSLGS

KINVEVRGNSKGTLKVLRSYNVMDMTNTTCQDLQIEVTVMGHVEYTMEAEEDYEDYEYED

LLAGDDPEAHSRXVTPLQLFDGRRNRRRREAPKAAEERESRVQYTVCIWRTGKVGLSGMA

IADITLLSGFHALRADLEKLTSLSDRYVSHFETEGPHVLLYFDSVPTSRECVGFGAVQEV

PVGLVQPASAILYDYYNPEHKCSVFYGAPRKSKLLSTLCSADVCQCAEGKCPRQRRALER

GQQDLEGYRMKFACYSPRVDYGFQVKVLREDSRAAFRLFETRITQVLHFTKDAGATADQT

RNFLVRASCRLQLEPGKEYLIMGLDGATYDLKGDPQYLLDSNSWIEEMPSERMCQSTRHR

TPCAQLNSFLQEYGTQXCQV

>sp|Q28106|CNTN1_BOVIN Contactin-1 OS=Bos taurus OX=9913 GN=CNTN1 PE=2 SV=1

MKMWLLFSLLVIISFKTCLSEFTWHRRYGHGVSEEDKGFGPIFEEQPINTIYPEESPEGK

VSLNCRARASPFPVYKWRMNNGDIDLTSDRYSMVGGNLVINNPDKQKDAGIYYCLASNNY

GMVRSTEATLSFGYLDPFPPEERPEVRVKEGKGMVLLCDPPYHFPDDLSYRWLLNEFPVF

ITMDKRRFVSQTNGNLYIANVEASDKGNYSCFVSSPSITKSVFSKFIPLIPLPERTTKPY

PADIVVQFKDVYALMGQNVTLECFALGNPVPDIRWRKVLEPMPSTAEISTSGAVLKIFNI

QLEDEGIYECEAENNRGKDKHQARIYVQAFPEWVEHINDTEVDIGSDLYWPCVATGKPIP

TIRWLKNGYSYHRGELRLYDVTFENAGMYQCIAENTHGAIYANAELKILALAPTFEMNPM

KKKILAAKGGRVIIECKPKAAPKPTFLWSKGTERLVNSSRILIWEDGSLEINNITRSDGG

VYTCFVENNKGKANSTGTLVITDPTRIILAPINADITVGENATMQCAASFDPALDLTFVW

SFNGYVIDFNKENIHYQRNFMLDSNGELLIRNAQLKHAGRYTCTAQTIVDNSSASADLVV

RGPPGPPGGLRIEDIRATSVALTWSRGSDNHSPISKYTIQTKTILSDDWKDAKTDPPIIE

GNMEAARAVDLIPWMEYEFRVVATNTLGIGEPSIPSNKIKTDGAAPNVAPSDVGGGGGSN

RELTITWAPLSREYHYFNNFGYIVAFKPFDGEEWKKVTVTNPDTGRYVHKDETMRPSTAF

QVKVKAFNNKGDGPYSLTAVIHSAQDAPSEAPTAVGVKVLSSSEISVHWEHVVEKIVESY

QIRYWASHDKEAAAHRVQVASQEYSARLENLLPDTQYFVEVRACNSAGCGPPSDMTETFT

KKAPPSQPPRIISSVRSGSRYIITWDHVVALSNESTVTGYKVLYRPDGQHDGKLYSTHKH

SIEVPIPRDGEYVVEVRAHSDGGDGVVSQVKISGASILSPCLLGFLLPALGILVYLEF

>sp|A6H768|GALK1_BOVIN Galactokinase OS=Bos taurus OX=9913 GN=GALK1 PE=2 SV=2

MAASEQPQAGELLAKARRAFLEEFGAEPELAVSAPGRVNLIGEHTDYNRGLVLPMALELV

TVLVGSPRVDGLVSLLTTSEDADEPRRLQFPLPTSQRPLEPGTPHWANYVKGVIQHYPAA

PLPGFSAVVVSSVPLGGGLSSSASLEVATYTFLQQLCPDSGTIAARAQVCQRAEHSFAGV

PCGIMDQLIALLGQRGHALLIDCRSLETSLVPLSDPKLAVLITNSNVRHSLGSSEYPLRR

RQCEEVARALGKESLREVQLEELEAGRDLMSTEAFRRARHVVGEIQRTAQAAAALRRGDY

RAFGRLMVESHHSLRDDYEVSCPELDQLVEAALSAPGVYGSRMTGGGFGGCTVTLLEASA

APRVMQHIQEQYHGTATFYLSQAADGAKVLHF

>sp|Q9TTE1|SPA31_BOVIN Serpin A3-1 OS=Bos taurus OX=9913 GN=SERPINA3-1 PE=1 SV=3

MRAERTSFLLALGLLVAGIRSVHCLPENVVVKDQHRRVDGHTLASSNTDFAFSLYKQLAL

KNPNKNVILSPLSVSIALAFLSLGARGSTLTEILEGLKFNLTEIQEKEIHHSFQHLLQAL

NQPSNQLQLSVGNAMFVQEELKLLDKFIEDAQVLYSSEAFPTNFRDSEAARSLINDYVKN

KTQGKIEELFKYLSPRTELVLVNYIYFKAQWKTPFDPKHTEQAEFHVSDNKTVEVPMMTL

DLETPYFRDEELGCTLVELTYTSNDSALFILPDEGKMRDLEAKLTPETLTRWRNSLQPRR

IHELYLPKFSIKSNYELNDILSQLGIRKIFANADLSGITGTADLVVSQVVHGAALDVDEE

GTEGAAATGISMERTILRIIVRVNRPFLIAIVLKDTQSIIFLGKVTNPSEA

>sp|Q2KJH9|AL9A1_BOVIN 4-trimethylaminobutyraldehyde dehydrogenase OS=Bos taurus OX=9913 GN=ALDH9A1 PE=2 SV=1

MSTGTFVVSQPLNYRGGARVEPVDASGTEKAFEPASGRVIATFTCSGEKEVNLAVQDAKA

AFKIWSQKSGMERCRILLEAARIIRERRDEIATMETINNGKSIFEARWDIDTSWQCLEYY

AGLAGSMAGEHIQLPGGSFGYTRREPLGVCVGIGAWNYPFQIACWKSAPALACGNAMVFK

PSPFTPVSVLLLAEIYTEAGVPPGLFNVVQGGAATGQFLCQHRDVAKVSFTGSVPTGSKI

MEMSAKGIKPVTLELGGKSPLIIFSDCDMKNAVKGALMANFLTQGEVCCNGTRVFVQKEI

LDQFTEEVVKQTQRIKIGDPLLEDTRMGPLINRPHLERVLGFVKVAKEQGAKVLCGGDVF

VPEDPKLKDGYYMRPCVLTNCRDDMTCVKEEIFGPVMSILSFDTEAEVLERANDTTFGLA

AGVFTRDIQRAHRVVAELQAGMCFINNYNVSPVELPFGGYKKSGFGRENGRVTIEYYSQL

KTVCVEMGDVESAF

>sp|P15497|APOA1_BOVIN Apolipoprotein A-I OS=Bos taurus OX=9913 GN=APOA1 PE=1 SV=3

MKAVVLTLAVLFLTGSQARHFWQQDDPQSSWDRVKDFATVYVEAIKDSGRDYVAQFEASA

LGKQLNLKLLDNWDTLASTLSKVREQLGPVTQEFWDNLEKETASLRQEMHKDLEEVKQKV

QPYLDEFQKKWHEEVEIYRQKVAPLGEEFREGARQKVQELQDKLSPLAQELRDRARAHVE

TLRQQLAPYSDDLRQRLTARLEALKEGGGSLAEYHAKASEQLKALGEKAKPVLEDLRQGL

LPVLESLKVSILAAIDEASKKLNAQ

>sp|Q3SYV4|CAP1_BOVIN Adenylyl cyclase-associated protein 1 OS=Bos taurus OX=9913 GN=CAP1 PE=2 SV=3

MADMRNLVERLERVVGRLEAVSHASDTHCGYGDSAAKAGTTPYVQAFDSLLAGPVAEYLK

ISKEIGGDVQKHAEMVHTGLKLERALLVTASQCQQPAGNKLSDLLAPISEQIQEVVTFRE

KNRGSKLFNHLSAVSESIQALGWVAMAPKPGPYVKEMNDAAMFYTNRVLKEYKDVDKKHV

DWVKAYLSIWTELQAYIKEFHTTGLAWSRTGPVAKELSGLPSGPSAGSGPPPPPPGPPPP

PVPTSSGSDDSASRSALFAQINQGESITHALKHVSDDMKTHKNPALKAQSGLIRSGPKPF

SASKPDPPKPVAKKEPALLELEGKKWRVENQENVSNLMIEDTELKQVAYIFKCVNSTLQI

KGKINSITVDNCKKLGLVFDDVVGIVEIINSKDVKVQVMGKVPTISINKTDGCHVYLSKN

SLDCEIVSAKSSEMNVLIPTEGGDFNEFPVPEQFKTLWNGQKLVTTVTEIAG

>sp|Q76LV2|HS90A_BOVIN Heat shock protein HSP 90-alpha OS=Bos taurus OX=9913 GN=HSP90AA1 PE=1 SV=3

MPEETQAQDQPMEEEEVETFAFQAEIAQLMSLIINTFYSNKEIFLRELISNSSDALDKIR

YESLTDPSKLDSGKELHINLIPNKQDRTLTIVDTGIGMTKADLINNLGTIAKSGTKAFME

ALQAGADISMIGQFGVGFYSAYLVAEKVTVITKHNDDEQYAWESSAGGSFTVRTDTGEPM

GRGTKVILHLKEDQTEYLEERRIKEIVKKHSQFIGYPITLFVEKERDKEVSDDEAEEKED

KEEEKEKEEKESDDKPEIEDVGSDEEEEEKKDGDKKKKKKIKEKYIDQEELNKTKPIWTR

NPDDITNEEYGEFYKSLTNDWEDHLAVKHFSVEGQLEFRALLFVPRRAPFDLFENRKKKN

NIKLYVRRVFIMDNCEELIPEYLNFIRGVVDSEDLPLNISREMLQQSKILKVIRKNLVKK

CLELFTELAEDKENYKKFYEQFSKNIKLGIHEDSQNRKKLSELLRYYTSASGDEMVSLKD

YCTRMKENQKHIYYITGETKDQVANSAFVERLRKHGLEVIYMIEPIDEYCVQQLKEFEGK

TLVSVTKEGLELPEDEEEKKKQEEKKTKFENLCKIMKDILEKKVEKVVVSNRLVTSPCCI

VTSTYGWTANMERIMKAQALRDNSTMGYMAAKKHLEINPDHSIIETLRQKAEADKNDKSV

KDLVILLYETALLSSGFSLEDPQTHANRIYRMIKLGLGIDEDDPTADDSSAAVTEEMPPL

EGDDDTSRMEEVD

>sp|P19120|HSP7C_BOVIN Heat shock cognate 71 kDa protein OS=Bos taurus OX=9913 GN=HSPA8 PE=1 SV=2

MSKGPAVGIDLGTTYSCVGVFQHGKVEIIANDQGNRTTPSYVAFTDTERLIGDAAKNQVA

MNPTNTVFDAKRLIGRRFDDAVVQSDMKHWPFMVVNDAGRPKVQVEYKGETKSFYPEEVS

SMVLTKMKEIAEAYLGKTVTNAVVTVPAYFNDSQRQATKDAGTIAGLNVLRIINEPTAAA

IAYGLDKKVGAERNVLIFDLGGGTFDVSILTIEDGIFEVKSTAGDTHLGGEDFDNRMVNH

FIAEFKRKHKKDISENKRAVRRLRTACERAKRTLSSSTQASIEIDSLYEGIDFYTSITRA

RFEELNADLFRGTLDPVEKALRDAKLDKSQIHDIVLVGGSTRIPKIQKLLQDFFNGKELN

KSINPDEAVAYGAAVQAAILSGDKSENVQDLLLLDVTPLSLGIETAGGVMTVLIKRNTTI

PTKQTQTFTTYSDNQPGVLIQVYEGERAMTKDNNLLGKFELTGIPPAPRGVPQIEVTFDI

DANGILNVSAVDKSTGKENKITITNDKGRLSKEDIERMVQEAEKYKAEDEKQRDKVSSKN

SLESYAFNMKATVEDEKLQGKINDEDKQKILDKCNEIINWLDKNQTAEKEEFEHQQKELE

KVCNPIITKLYQSAGGMPGGMPGGMPGGFPGGGAPPSGGASSGPTIEEVD

>sp|Q05443|LUM_BOVIN Lumican OS=Bos taurus OX=9913 GN=LUM PE=1 SV=1

MNLGVFPLLLALIGGASSTYPDYYEYYDFPQALYGRSSPNCAPECNCPESYPSAMYCDEL

KLKSVPMVPPGIKYLYLRNNQIDHIDDKAFENVTDLQWLILDHNLLENSKIKGKVFSKLK

QLKKLHINYNNLTESVGPLPKSLVDLQLTNNKISKLGSFDGLVNLTFIHLQHNQLKEDAV

SAALKGLKSLEYLDLSFNQMTKLPSGLPVSLLTLYLDNNKISNIPDEYFKRFSALQYLRL

SHNELADSGVPGNSFNVSSLLELDLSYNKLKSIPTVNENLENYYLEVNELEKFDVKSFCK

ILGPLSYSKIKHLRLDGNHITQTSLPPDMYECLRVANEITVN

>sp|Q2KJF1|A1BG_BOVIN Alpha-1B-glycoprotein OS=Bos taurus OX=9913 GN=A1BG PE=1 SV=1

MSAWAALLLLWGLSLSPVTEQATFFDPRPSLWAEAGSPLAPWADVTLTCQSPLPTQEFQL

LKDGVGQEPVHLESPAHEHRFPLGPVTSTTRGLYRCSYKGNNDWISPSNLVEVTGAEPLP

APSISTSPVSWITPGLNTTLLCLSGLRGVTFLLRLEGEDQFLEVAEAPEATQATFPVHRA

GNYSCSYRTHAAGTPSEPSATVTIEELDPPPAPTLTVDRESAKVLRPGSSASLTCVAPLS

GVDFQLRRGAEEQLVPRASTSPDRVFFRLSALAAGDGSGYTCRYRLRSELAAWSRDSAPA

ELVLSDGTLPAPELSAEPAILSPTPGALVQLRCRAPRAGVRFALVRKDAGGRQVQRVLSP

AGPEAQFELRGVSAVDSGNYSCVYVDTSPPFAGSKPSATLELRVDGPLPRPQLRALWTGA

LTPGRDAVLRCEAEVPDVSFLLLRAGEEEPLAVAWSTHGPADLVLTSVGPQHAGTYSCRY

RTGGPRSLLSELSDPVELRVAGS

>sp|Q5E9B5|ACTH_BOVIN Actin, gamma-enteric smooth muscle OS=Bos taurus OX=9913 GN=ACTG2 PE=2 SV=1

MCEEETTALVCDNGSGLCKAGFAGDDAPRAVFPSIVGRPRHQGVMVGMGQKDSYVGDEAQ

SKRGILTLKYPIEHGIITNWDDMEKIWHHSFYNELRVAPEEHPTLLTEAPLNPKANREKM

TQIMFETFNVPAMYVAIQAVLSLYASGRTTGIVLDSGDGVTHNVPIYEGYALPHAIMRLD

LAGRDLTDYLMKILTERGYSFVTTAEREIVRDIKEKLCYVALDFENEMATAASSSSLEKS

YELPDGQVITIGNERFRCPETLFQPSFIGMESAGIHETTYNSIMKCDIDIRKDLYANNVL

SGGTTMYPGIADRMQKEITALAPSTMKIKIIAPPERKYSVWIGGSILASLSTFQQMWISK

PEYDEAGPSIVHRKCF

>sp|P14568|ASSY_BOVIN Argininosuccinate synthase OS=Bos taurus OX=9913 GN=ASS1 PE=2 SV=1

MSGKGSVVLAYSGGLDTSCILVWLKEQGYDVIAYLANIGQKEDFEEARKKALKLGAKKVF

IEDISKEFVEEFIWPAIQSSALYEDRYLLGTSLARPCIARKQVEIAQREGAKYVSHGATG

KGNDQIRFELTCYSLAPQIKVIAPWRMPEFYNRFQGRNDLMEYAKQHGIPVPVTPKNPWS

MDENLMHISYEAGILENPKNQAPPGLYTKTQDPAKAPNSPDMLEIEFKKGVPVKVTNVGD

GTTHSTALELFLYLNEVAGKHGVGRIDIVENRFIGMKSRGIYETPAGTILYHAHLDIEAF

TMDREVRKIKQGLGLKFAELVYTGFWHSPECEFVRHCIAKSQERVEGKVQVSVFKGQVYI

LGRESPLSLYNEELVSMNVQGDYEPVDATGFININSLRLKEYHRLQNKVTAK

>sp|Q58D62|FETUB_BOVIN Fetuin-B OS=Bos taurus OX=9913 GN=FETUB PE=1 SV=1

MNVLLLLVLCTLAMGCGATSPPQPAARPSSLLSLDCNSSYVLDIANDILQDINRDRKDGY

VLSLNRVSDAREHRQEAGLGSLFYFTLDVLETGCHVLSRTSWKNCEVRIFHESVYGQCKA

IFYINKEKRIFYLPAYNCTLRPVSQSAIIMTCPDCPSTSPYDLSNPRFMETATESLAKYN

SESPSKQYSLVKITKTSSQWVFGPAYFVEYLIKESPCVKSEGSSCALESPGSVPVGICHG

SLGEPQGNQGKIISVTCSFFNSQAPTPRGENATVNQRPANPSKTEELQQQNTAPTNSPTK

AVPKGSVQYLPDWDKKREGSQEKDPVETFPVQLDLTTNPQGESLDVSFLFQEPMEEKVVV

LPFPSKEQRSAECPGPAQKGYPFILPS

>sp|Q28035|GSTA1_BOVIN Glutathione S-transferase A1 OS=Bos taurus OX=9913 GN=GSTA1 PE=2 SV=3

MAGKPTLHYFNGRGRMECIRWLLAAAGVEFEEKFIEKPEDLDKLKNDGSLMFQQVPMVEI

DGMKLVQTRAILNYIATKYNLYGKDMKERALIDMYSEGVADLGEMIMHFPLCPPAEKDAK

LTLIREKTTNRYLPAFENVLKSHGQDYLVGNKLSRADIHLVELLYYVEELDPSLLANFPL

LKALKARVSNIPAVKKFLQPGSQRKPPTDEKKIEEARKVFKF

>sp|P38657|PDIA3_BOVIN Protein disulfide-isomerase A3 OS=Bos taurus OX=9913 GN=PDIA3 PE=2 SV=1

MRLRRLALFPGLALLLAAARLAAASDVLELTDDNFESRITDTGSSGLMLVEFFAPWCGHC

KKLAPEYEAAATRLKGIVPLAKVDCTANTNTCNKYGVSGYPTLKIFRDGEESGAYDGPRT

ADGIVSHLKKQAGPASVPLKSEEEFEKFISDKDASVVGFFKDLFSEAHSEFLKAASNLRD

NYRFAHTNVESLVNKYDDDGEGITLFRPSHLTNKFEDKTVAYTEQKMTSGKIKRFIQENI

FGICPHMTEDNKDLLQGKDLLIAYYDVDYEKNAKGSNYWRNRVMMVAKKFLDAGQKLHFA

VASRKTFSHELSDFGLESTTGEIPVVAVRTAKGEKFVMQEEFSRDGKALERFLEDYFDGN

LKRYLKSEPIPESNDGPVKVVVAENFDEIVNNENKDVLIEFYAPWCGHCKNLEPKYKELG

EKLRKDPNIVIAKMDATANDVPSPYEVRGFPTIYFSPANKKQNPKKYEGGRELSDFISYL

KREATNPPVIQEEKPKKKKKAQEDL

>sp|Q08DP0|PGM1_BOVIN Phosphoglucomutase-1 OS=Bos taurus OX=9913 GN=PGM1 PE=2 SV=1

MVKIVTVKTKAYQDQKPGTSGLRKRVKVFQSSSNYAENFIQSIISTVEPAQRQEATLVVG

GDGRFYMKEAIQLIVRIAAANGIGRLVIGQNGILSTPAVSCIIRKIKAIGGIILTASHNP

GGPNGDFGIKFNISNGGPAPEAITDKIFQISKTIEEYAICPDLHVDLGVLGKQQFDLENK

FKPFTVEIVDSVEAYATMLRNIFDFNALKELLSGPNRLKIRIDAMHGVVGPYVKKILCEE

LGAPANSAVNCVPLEDFGGHHPDPNLTYAADLVETMKTGEHDFGAAFDGDGDRNMILGKH

GFFVNPSDSVAVIAANIFSIPYFQQTGVRGFARSMPTSGALDRVANATKIALYETPTGWK

FFGNLMDASKLSLCGEESFGTGSDHIREKDGLWAVLAWLSILATRKQSVEDILKDHWQKY

GRNFFTRYDYEEVEAEGANKMMKELEALISDRSFVGKQFPVGDKVYTVEKIDNFEYSDPV

DGSISRNQGLRLLFADGSRIIFRLSGTGSAGATIRLYIDSYEKDLAKIYQDPQVMLAPLI

SIALKVSQLQEKTGRTAPTVIT

>sp|A2I7M9|SPA32_BOVIN Serpin A3-2 OS=Bos taurus OX=9913 GN=SERPINA3-2 PE=3 SV=1

MRAERTSFLLALGLLVAGIRSVHCLPENVVVKDQHRRVDGHTLASSNTDFAFSLYKQLAL

KNPNKNVILSPLSVSIALAFLSLGARGSTLTEILEGLKFNLTEIQEKEIHHSFQHLLQAL

NQPSNQLQLSVGNAMFVQEELKLLDKFIEDAQVLYSSEAFPTNFRDSEAARSLINDYVKN

KTQGKIEELFKYLSPRTELVLVNYIYFKAQWKTPFDPKHTEQAEFHVSDNKTVEVPMMTL

DLETPYFRDEELGCTLVELTYTSNDSALFILPDEGKMRDLEAKLTPETLTRWRNSLQPRR

IHELYLPKFSIKSNYELNDILSQLGIRKIFANADLSGITGTADLVVSQVVHGAALDVDEE

GTEGVAATGIGIERTFLRIIVRVNRPFLIAVVLKDTQSIIFLGKVTNPSEA

>sp|P81948|TBA4A_BOVIN Tubulin alpha-4A chain OS=Bos taurus OX=9913 GN=TUBA4A PE=1 SV=2

MRECISVHVGQAGVQMGNACWELYCLEHGIQPDGQMPSDKTIGGGDDSFTTFFCETGAGK

HVPRAVFVDLEPTVIDEIRNGPYRQLFHPEQLITGKEDAANNYARGHYTIGKEIIDPVLD

RIRKLSDQCTGLQGFLVFHSFGGGTGSGFTSLLMERLSVDYGKKSKLEFSIYPAPQVSTA

VVEPYNSILTTHTTLEHSDCAFMVDNEAIYDICRRNLDIERPTYTNLNRLISQIVSSITA

SLRFDGALNVDLTEFQTNLVPYPRIHFPLATYAPVISAEKAYHEQLSVAEITNACFEPAN

QMVKCDPRHGKYMACCLLYRGDVVPKDVNAAIAAIKTKRSIQFVDWCPTGFKVGINYQPP

TVVPGGDLAKVQRAVCMLSNTTAIAEAWARLDHKFDLMYAKRAFVHWYVGEGMEEGEFSE

AREDMAALEKDYEEVGIDSYEDEDEGEE

>sp|P81947|TBA1B_BOVIN Tubulin alpha-1B chain OS=Bos taurus OX=9913 PE=1 SV=2

MRECISIHVGQAGVQIGNACWELYCLEHGIQPDGQMPSDKTIGGGDDSFNTFFSETGAGK

HVPRAVFVDLEPTVIDEVRTGTYRQLFHPEQLITGKEDAANNYARGHYTIGKEIIDLVLD

RIRKLADQCTGLQGFLVFHSFGGGTGSGFTSLLMERLSVDYGKKSKLEFSIYPAPQVSTA

VVEPYNSILTTHTTLEHSDCAFMVDNEAIYDICRRNLDIERPTYTNLNRLISQIVSSITA

SLRFDGALNVDLTEFQTNLVPYPRIHFPLATYAPVISAEKAYHEQLSVAEITNACFEPAN

QMVKCDPRHGKYMACCLLYRGDVVPKDVNAAIATIKTKRSIQFVDWCPTGFKVGINYQPP

TVVPGGDLAKVQRAVCMLSNTTAIAEAWARLDHKFDLMYAKRAFVHWYVGEGMEEGEFSE

AREDMAALEKDYEEVGVDSVEGEGEEEGEEY

>sp|P68138|ACTS_BOVIN Actin, alpha skeletal muscle OS=Bos taurus OX=9913 GN=ACTA1 PE=1 SV=1

MCDEDETTALVCDNGSGLVKAGFAGDDAPRAVFPSIVGRPRHQGVMVGMGQKDSYVGDEA

QSKRGILTLKYPIEHGIITNWDDMEKIWHHTFYNELRVAPEEHPTLLTEAPLNPKANREK

MTQIMFETFNVPAMYVAIQAVLSLYASGRTTGIVLDSGDGVTHNVPIYEGYALPHAIMRL

DLAGRDLTDYLMKILTERGYSFVTTAEREIVRDIKEKLCYVALDFENEMATAASSSSLEK

SYELPDGQVITIGNERFRCPETLFQPSFIGMESAGIHETTYNSIMKCDIDIRKDLYANNV

MSGGTTMYPGIADRMQKEITALAPSTMKIKIIAPPERKYSVWIGGSILASLSTFQQMWIT

KQEYDEAGPSIVHRKCF

>sp|Q3ZC07|ACTC_BOVIN Actin, alpha cardiac muscle 1 OS=Bos taurus OX=9913 GN=ACTC1 PE=2 SV=1

MCDDEETTALVCDNGSGLVKAGFAGDDAPRAVFPSIVGRPRHQGVMVGMGQKDSYVGDEA

QSKRGILTLKYPIEHGIITNWDDMEKIWHHTFYNELRVAPEEHPTLLTEAPLNPKANREK

MTQIMFETFNVPAMYVAIQAVLSLYASGRTTGIVLDSGDGVTHNVPIYEGYALPHAIMRL

DLAGRDLTDYLMKILTERGYSFVTTAEREIVRDIKEKLCYVALDFENEMATAASSSSLEK

SYELPDGQVITIGNERFRCPETLFQPSFIGMESAGIHETTYNSIMKCDIDIRKDLYANNV

LSGGTTMYPGIADRMQKEITALAPSTMKIKIIAPPERKYSVWIGGSILASLSTFQQMWIS

KQEYDEAGPSIVHRKCF

>sp|P81644|APOA2_BOVIN Apolipoprotein A-II OS=Bos taurus OX=9913 GN=APOA2 PE=1 SV=2

MKLLALTVLLLTICGLEGALVRRQAEESNLQSLVSQYFQTVADYGKDLVEKAKGSELQTQ

AKAYFEKTQEELTPFFKKAGTDLLNFLSSFIDPKKQPATR

>sp|Q03247|APOE_BOVIN Apolipoprotein E OS=Bos taurus OX=9913 GN=APOE PE=1 SV=1

MKVLWVAVVVALLAGCQADMEGELGPEEPLTTQQPRGKDSQPWEQALGRFWDYLRWVQTL

SDQVQEELLNTQVIQELTALMEETMKEVKAYKEELEGQLGPMAQETQARVSKELQAAQAR

LGSDMEDLRNRLAQYRSEVQAMLGQSTEELRARMASHLRKLPKRLLRDADDLKKRLAVYQ

AGASEGAERSLSAIRERFGPLVEQGQSRAATLSTLAGQPLLERAEAWRQKLHGRLEEVGV

RAQDRLDKIRQQLEEVHAKVEEQGNQMRLQAEAFQARLRSWFEPLVEDMQRQWAGLVEKV

QLALRPSPTSPPSENH

>sp|P52898|DDBX_BOVIN Dihydrodiol dehydrogenase 3 OS=Bos taurus OX=9913 PE=2 SV=1

MDPKGQRVKLNDGHFIPVLGFGTFAPREVPKSEALEVTKFAIEAGFRHIDSAHLYQNEEQ

VGQAIRSKIADGTVKREDIFYTSKLWSTSLRPELVRPALEKSLNNLQLDYVDLYIIHFPV

ALKPGETLFPTDENGKPIFDSVDLCRTWEALEKCKDAGLTKSIGVSNFNHKQLEKILNKP

GLKYKPVCNQVECHPYFNQSKLLDFCKSHDIVLVAYGALGSQRLKEWVNPNLPFLLEDPV

LSAIAKKHRQTPALVALRYQIQRGVVVLAKSYNKKRIKENIQVFDFELTPEDMKAIDGLN

SNMRYNELLLGVGHPEYPFVEEY

>sp|P01966|HBA_BOVIN Hemoglobin subunit alpha OS=Bos taurus OX=9913 GN=HBA PE=1 SV=2

MVLSAADKGNVKAAWGKVGGHAAEYGAEALERMFLSFPTTKTYFPHFDLSHGSAQVKGHG

AKVAAALTKAVEHLDDLPGALSELSDLHAHKLRVDPVNFKLLSHSLLVTLASHLPSDFTP

AVHASLDKFLANVSTVLTSKYR

>sp|P06394|K1C10_BOVIN Keratin, type I cytoskeletal 10 OS=Bos taurus OX=9913 GN=KRT10 PE=3 SV=1

MSVRYSSSKQYSSSRSGGGGGGGSSLRISSSKGSLGGGYSSGGFSGGSFSRGSSAGGCFG

GSSSIYGGGLGSGFGGGYGSSFGGSYGGSFGGGYGGGGFGGGSFGGGSFGGGLGGGFGDG

GLISGNQKITMQNLNDRLASYLDKVRALEESNYELEVKIKEWYEKYGNSRQREPRDYSKY

YQTIDDLKNQIFNLTTDNANILIQVDNARLAADDFRLKYENEVTLRQSVEADINGLRRVL

DELTLTKTDLEMQIESLTEELAYLKKNHEEEMRDLQNVSTGDVNVEMNAAPGVDLTELLN

NMRSQYEQLAEKNRRDAEAWFNEKSKELTTEINSNLEQVSSHKSEITELRRTIQGLEIEL

QSQLALKQSLEASLAETEGRYCVQLSQIQSQISSLEEQLQQIRAETECQNAEYQQLLDIK

IRLENEIQTYRSLLEGEGSSGGGSYGGGRGYGGSSGGGGGGYGGGSSSGGYGGGSSSGGG

HGGSSGGSYGGGSSSGGGHGGGSSSGGHKSTTTGSVGESSSKGPRY

>sp|A1A4J1|PFKAL_BOVIN ATP-dependent 6-phosphofructokinase, liver type OS=Bos taurus OX=9913 GN=PFKL PE=2 SV=1

MASVDLEKLRTTGAGKAIGVLTSGGDAQGMNAAVRAVTRMGIYVGAKVFLIYEGYEGLVE

GGENIKQANWLSVSNIIQLGGTVIGSARCKAFTTREGRRAAAYNLVQRGITNLCVIGGDG

SLTGANIFRSEWGSLLEELVSEGKISEGTAQTYSHLNIAGLVGSIDNDFCGTDMTIGTDS

ALHRIMEVIDAITTTAQSHQRTFVLEVMGRHCGYLALVSALASGADWLFIPEAPPEDGWE

NFMCERLGETRSRGSRLNIIIIAEGAIDRNGKPISSRYVKDLVVQRLGFDTRVTVLGHVQ

RGGTPSAFDRILSSKMGMEAVMALLEATPDTPACVVSLSGNQSVRLPLMECVQMTKEVQK

AMDEKRFDEAIQLRGGSFENNWNIYKLLSHQKISKEKTNFSLAILNVGAPAAGMNAAVRS

AVRSGISQGHTVYVVHDGFEGLAKNQVQEVSWHDVAGWLGRGGSMLGTKRTLPKGFMEKI

VENIRLHNIHALLVIGGFEAYEGVLQLVEARGRYEELCIVMCVIPATISNNVPGTDFSLG

SDTAVNAAMESCDRIKQSASGTKRRVFIVETMGGYCGYLATVTGIAVGADAAYVFEDPFN

IQDLKANVEHMTEKMKTEIQRGLVLRNEKCHEHYTTEFLYNLYSSEGKGVFDCRTNVLGH

LQQGGAPTPFDRNYGTKLGVKAIIWMSEKLRAVYRNGRVFANASDSACVIGLQKKVVAFS

PVTELKKDTDFEHRMPREQWWLNLRLMLKMLAHYRISMADYVSGELEHVTRRTLSIETGF

>sp|Q2HJ86|TBA1D_BOVIN Tubulin alpha-1D chain OS=Bos taurus OX=9913 GN=TUBA1D PE=1 SV=1

MRECISVHVGQAGVQIGNACWELYCLEHGIQPDGQMPSDKTIGGGDDSFNTFFSETGAGK

HVPRAVFVDLEPTVIDEVRTGTYRQLFHPEQLITGKEDAANNYARGHYTIGKELIDLVLD

RIRKLADQCTGLQGFLIFHSFGGGTGSGFTSLLMERLSVDYGKKSKLEFSIYPAPQVSTA

VVEPYNSILTTHTTLEHSDCAFMVDNEAIYDICRRNLDIERPTYTNLNRLIGQIVSSITA

SLRFDGALNVDLTEFQTNLVPYPRIHFPLATYAPVISAEKAYHEQLSVAEITNACFEPAN

QMVKCDPRHGKYMACCLLYRGDVVPKDVNAAIATIKTKRTIQFVDWCPTGFKVGINYQPP

TVVPGGDLAKVQRAVCMLSNTTAIAEAWARLDHKFDLMYAKRAFVHWYVGEGMEEGEFSE

AREDMAALEKDYEEVGMDSVEGEGEEEEGDEY

>sp|Q1JPJ2|XPP1_BOVIN Xaa-Pro aminopeptidase 1 OS=Bos taurus OX=9913 GN=XPNPEP1 PE=2 SV=1

MAPKITSELLRQLRQAMRNLEYVTEPIQAYIIPSGDAHQSEYIAPCDCRRAFVSGFDGSA

GTAIVTEEHAAMWTDGRYFLQAAKQMDSNWTLMKMGLKDTPTQEDWLVSVLPEGSRVGVD

PLIIPTDYWKKMAKVLRSAGHHLIPVKDNLVDKIWTDRPERPCKPLITLGLDYTGISWKD

KVADLRLKMAERNVVWFVVTALDEIAWLFNLRGSDVEHNPVFFSYAILGLETIMLFIDGD

RIDAPIVKEHLLLDLGLEAEYRIQVLPYKSILSELKILCASLSPREKVWVSDKASYAVSE

AIPKDHRCCMPYTPICIAKAVKNSAESEGMRRAHIKDAVALCELFNWLEKEVPKGGVTEI

SAANKAEEFRRQQADFVDLSFPTISSTGPNGAIIHYAPVPETNRTLSLDEVYLIDSGAQY

KDGTTDVTRTMHFGTPTAYEKECFTYVLKGHIAVSAAVFPTGTKGHLLDSFARSALWDSG

LDYLHGTGHGVGSFLNVHEGPCGISYKTFSDEPLEAGMIVTDEPGYYEDGAFGIRIENVV

LVVPVKTKYNFNNRGSLTFEPLTLVPIQTKMIDVDSLTDKECDWLNSYHLTCRDVIGKEL

QKQGRQEALEWLIRETQPISKQP

>sp|P00978|AMBP_BOVIN Protein AMBP OS=Bos taurus OX=9913 GN=AMBP PE=1 SV=2

MRSLSGLLLLLTACLAVNASSVPTLPDDIQVQENFDLSRIYGKWFNVAVGSTCPWLKRFK

EKMTMSTVVLIAGPTSKEISVTNTHRRKGVCESISGTYEKTSADGKFLYHKAKWNITMES

YVVHTNYDEYAIFLTKKLSRRHGPTITVKLYGREPQLRESLLEEFREVALGVGIPEDAIF

TMPDRGECVPGEQDPVPTPLSRARRAVLTQEEEGSGAGQPVTNFSKKADSCQLDYSQGPC

LGLFKRYFYNGTSMACETFLYGGCMGNGNNFLSEKECLQTCRTVEACNLPIVQGPCRSYI

QLWAFDAVKGKCVRFSYGGCKGNGNKFYSEKECKEYCGIPGEADEELLRFSN

>sp|P52556|BLVRB_BOVIN Flavin reductase (NADPH) OS=Bos taurus OX=9913 GN=BLVRB PE=1 SV=2

MVVKKIALFGATGNTGLTTLAQAVQAGYEVTVLVRDPSRLPSEGPQPAHVVVGDVRQPAD

VDKTVAGQDAVIVLLGTRNDLSPTTVMSEGAQNIVAAMKAHGVDKVVACTSAFLLWDPSK

VPPRLQDVTDDHIRMHKVLQQSGLKYVAVMPPHIGDHPLTGAYTVTLDGRGPSRVISKHD

LGHFMLHCLTTDKYDGHTTYPSHVYE

>sp|P68103|EF1A1_BOVIN Elongation factor 1-alpha 1 OS=Bos taurus OX=9913 GN=EEF1A1 PE=1 SV=1

MGKEKTHINIVVIGHVDSGKSTTTGHLIYKCGGIDKRTIEKFEKEAAEMGKGSFKYAWVL

DKLKAERERGITIDISLWKFETSKYYVTIIDAPGHRDFIKNMITGTSQADCAVLIVAAGV

GEFEAGISKNGQTREHALLAYTLGVKQLIVGVNKMDSTEPPYSQKRYEEIVKEVSTYIKK

IGYNPDTVAFVPISGWNGDNMLEPSANMPWFKGWKVTRKDGNASGTTLLEALDCILPPTR

PTDKPLRLPLQDVYKIGGIGTVPVGRVETGVLKPGMVVTFAPVNVTTEVKSVEMHHEALS

EALPGDNVGFNVKNVSVKDVRRGNVAGDSKNDPPMEAAGFTAQVIILNHPGQISAGYAPV

LDCHTAHIACKFAELKEKIDRRSGKKLEDGPKFLKSGDAAIVDMVPGKPMCVESFSDYPP

LGRFAVRDMRQTVAVGVIKAVDKKAAGAGKVTKSAQKAQKAK

>sp|P02672|FIBA_BOVIN Fibrinogen alpha chain OS=Bos taurus OX=9913 GN=FGA PE=1 SV=5

MFSVRDLCLVLSLVGAIKTEDGSDPPSGDFLTEGGGVRGPRLVERQQSACKETGWPFCSD

EDWNTKCPSGCRMKGLIDEVDQDFTSRINKLRDSLFNYQKNSKDSNTLTKNIVELMRGDF

AKANNNDNTFKQISEDLRSRIEILRRKVIEQVQRIKVLQKNVRDQLVDMKRLEVDIDIKI

RSCKGSCSRALEHKVDLEDYKNQQKQLEQVIAINLLPSRDIQYLPLIKMSTITGPVPREF

KSQLQEAPLEWKALLEMQQTKMVLETFGGDGHARGDSVSQGTGLAPGSPRKPGTSSIGNV

NPGSYGPGSSGTWNPGRPEPGSAGTWNPGRPEPGSAGTWNPGRPEPGSAGTWNPGRPEPG

SAGTWNPGRPEPGSAGTWNTGSSGSSSFRPDSSGHGNIRPSSPDWGTFREEGSVSSGTKQ

EFHTGKLVTTKGDKELLIDNEKVTSGHTTTTRRSCSKVITKTVTNADGRTETTKEVVKSE

DGSDCGDADFDWHHTFPSRGNLDDFFHRDKDDFFTRSSHEFDGRTGLAPEFAALGESGSS

SSKTSTHSKQFVSSSTTVNRGGSAIESKHFKMEDEAESLEDLGFKGAHGTQKGHTKARPA

RGIHTSPLGEPSLTP

>sp|P50397|GDIB_BOVIN Rab GDP dissociation inhibitor beta OS=Bos taurus OX=9913 GN=GDI2 PE=2 SV=3

MNEEYDVIVLGTGLTECILSGIMSVNGKKVLHMDRNPYYGGESASITPLEDLYKRFKIPG

APPASMGRGRDWNVDLIPKFLMANGQLVKMLLFTEVTRYLDFKVTEGSFVYKGGKIYKVP

STEAEALASSLMGLFEKRRFRKFLVYVANFDENDPRTFEGIDPKKTSMREVYKKFDLGQD

VIDFTGHALALYRTDDYLDQPCCETINRIKLYSESLARYGKSPYLYPLYGLGELPQGFAR

LSAIYGGTYMLNKPIEEIIMQNGKVIGVKSEGEIARCKQLICDPSYVKDRVEKVGQVIRV

ICILSHPIKNTNDANSCQIIIPQNQVNRKSDIYVCMISSAHNVAAQGKYIAIASTTVETK

EPEKEIRPALELLEPIEQKFVSISDLLVPKDLGTESQIFISRTYDATTHFETTCDDIKDI

YKRMMGSEFDFEEMKRKKNDIYGEE

>sp|P28801|GSTP1_BOVIN Glutathione S-transferase P OS=Bos taurus OX=9913 GN=GSTP1 PE=1 SV=2

MPPYTIVYFPVQGRCEAMRMLLADQGQSWKEEVVAMQSWLQGPLKASCLYGQLPKFQDGD

LTLYQSNAILRHLGRTLGLYGKDQQEAALVDMVNDGVEDLRCKYVSLIYTNYEAGKEDYV

KALPQHLKPFETLLSQNKGGQAFIVGDQISFADYNLLDLLRIHQVLAPSCLDSFPLLSAY

VARLNSRPKLKAFLASPEHMNRPINGNGKQ

>sp|P01045|KNG2_BOVIN Kininogen-2 OS=Bos taurus OX=9913 GN=KNG2 PE=1 SV=1

MKLITILFLCSRLLPSLTQESSQEIDCNDQDVFKAVDAALTKYNSENKSGNQFVLYRITE

VARMDNPDTFYSLKYQIKEGDCPFQSNKTWQDCDYKDSAQAATGQCTATVAKRGNMKFSV

AIQTCLITPAEGPVVTAQYECLGCVHPISTKSPDLEPVLRYAIQYFNNNTSHSHLFDLKE

VKRAQKQVVSGWNYEVNYSIAQTNCSKEEFSFLTPDCKSLSSGDTGECTDKAHVDVKLRI

SSFSQKCDLYPGEDFLPPMVCVGCPKPIPVDSPDLEEALNHSIAKLNAEHDGTFYFKIDT

VKKATVQVVGGLKYSIVFIARETTCSKGSNEELTKSCEINIHGQILHCDANVYVVPWEEK

VYPTVNCQPLGQTSLMKRPPGFSPFRSVQVMKTEGSTTVSLPHSAMSPVQDEERDSGKEQ

GPTHGHGWDHGKQIKLHGLGLGHKHKHDQGHGHHRSHGLGHGHQKQHGLGHGHKHGHGHG

KHKNKGKNNGKHYDWRTPYLASSYEDSTTSSAQTQEKTEETTLSSLAQPGVAITFPDFQD

SDLIATVMPNTLPPHTESDDDWIPDIQTEPNSLAFKLISDFPETTSPKCPSRPWKPVNGV

NPTVEMKESHDFDLVDALL

>sp|A7E3W2|LG3BP_BOVIN Galectin-3-binding protein OS=Bos taurus OX=9913 GN=LGALS3BP PE=1 SV=1

MAPLRLFWIWLLVVGTRGVKDGDMRLADGGSANQGRVEIYYNGQWGTVCENMWDLTDASV

VCRALGFQNATEALGGAAFGPGYGPIMLDEVRCTGTEPSLANCSSLGWMRSNCRHDKDAS

VICTNETRGVYTLDLSGELPAALEQIFESQKGCDLFITVKVREEDEIAMCAHKLILSTNP

EAHGLWKEPGSRVTMEVDAECVPVVKDFIRYLYSRRIDVSLSSVKCLHKLASAYQAKQLQ

SYCGHLFAILIPQDPSFWTPLELYAYALATRDPVLEEICVQFLAWNFGALTQAEAWPSVP

PALLQGLLSRTELVVPSELVLLLAVDKWSQERRTSHKEVEALVGQVRFPMMPPQDLFSLQ

FNLSLYWSHEALFQKKILQALEFHTVPFELLAQYWGLNLTEGTYQPRLYTSPTWSQSVMS

SSYNPSRSFQTPQHPSFLFHDSSVSWSFVYLPTLQSCWNYGFSCSSDDPPLLALSKSSYS

KSNPTIGYENRALLHCEGSFVVDVIDFKGWKALVPSALATNSSRSTSLFPCPSGVFSRFQ

VVIRPFYLTNSTDMD

>sp|Q3B7M9|PYGB_BOVIN Glycogen phosphorylase, brain form OS=Bos taurus OX=9913 GN=PYGB PE=2 SV=3

MAKPLTDGERRKQISVRGLAGLGDVAEVRKSFNRHLHFTLVKDRNVATRRDYYLALAHTV

RDHLVGRWIRTQQRYYERDPKRIYYLSLEFYMGRTLQNTMVNLGLQNACDEAIYQLGLDL

EELEEIEEDAGLGNGGLGRLAACFLDSMATLGLAAYGYGIRYEFGIFNQKIVNGWQVEEA

DDWLRYGNPWEKARPEYMLPVHFYGRVEHSPEGVRWLDTQVVLAMPYDTPVPGYKNDTVN

TMRLWSAKAPNDFKLHDFNVGGYIEAVLDRNLAENISRVLYPNDNFFEGKELRLKQEYFV

VAATLQDIIRRFKSSKFGCRDPVRTSFETFPDKVAIQLNDTHPALAIPELMRILVDVEKV

DWDKAWEITKKTCAYTNHTVLPEALERWPVSMFEKLLPRHLDIIYAINQRHLDHVAALFP

GDVDRLRRMSVIEEGDCKRINMAHLCVIGSHAVNGVARIHSEIVRQSVFKDFYELEPEKF

QNKTNGITPRRWLLLCNPGLAETIVERIGEGFLTDLSQLKKLLPLVGDEALIRDVAQVKQ

ENKVKFSAFLEKQYGVKVNPSSMFDVHVKRIHEYKRQLLNCLHVVTLYNRIKKDPTQAFV

PRTVMIGGKAAPGYHMAKKIIKLVTSIGNIVNHDPIVGDRLKVIFLENYRVSLAEKVIPA

ADLSQQISTAGTEASGTGNMKFMLNGALTIGTMDGANVEMAEEAGAENLFIFGLRVEDVE

ALDRKGYNAHEYYDRLPELRQAVDQINGGFFSPREPDCFKDVVNMLLNHDRFKVFADYEA

YVACQARVDQLYRNPKEWTKKVIRNIACSGKFSSDRTITEYAHDIWGAEPPALQTPPPSL

PRD

>sp|O46375|TTHY_BOVIN Transthyretin OS=Bos taurus OX=9913 GN=TTR PE=1 SV=1

MASFRLFLLCLAGLVFVSEAGSVGAGEPKCPLMVKVLDAVRGSPAANVGVKVFKKAADET

WEPFASGKTSESGELHGLTTEDKFVEGLYKVELDTKSYWKSLGISPFHEFAEVVFTANDS

GPRHYTIAALLSPYSYSTTALVSSPKA

>sp|Q3ZBU7|TBB4A_BOVIN Tubulin beta-4A chain OS=Bos taurus OX=9913 GN=TUBB4A PE=2 SV=1

MREIVHLQAGQCGNQIGAKFWEVISDEHGIDPTGTYHGDSDLQLERINVYYNEATGGNYV

PRAVLVDLEPGTMDSVRSGPFGQIFRPDNFVFGQSGAGNNWAKGHYTEGAELVDAVLDVV

RKEAESCDCLQGFQLTHSLGGGTGSGMGTLLISKIREEFPDRIMNTFSVVPSPKVSDTVV

EPYNATLSVHQLVENTDETYCIDNEALYDICFRTLKLTTPTYGDLNHLVSATMSGVTTCL

RFPGQLNADLRKLAVNMVPFPRLHFFMPGFAPLTSRGSQQYRALTVPELTQQMFDAKNMM

AACDPRHGRYLTVAAVFRGRMSMKEVDEQMLSVQSKNSSYFVEWIPNNVKTAVCDIPPRG

LKMAATFIGNSTAIQELFKRISEQFTAMFRRKAFLHWYTGEGMDEMEFTEAESNMNDLVS

EYQQYQDATAEEGEFEEEAEEEVA

>sp|P12378|UGDH_BOVIN UDP-glucose 6-dehydrogenase OS=Bos taurus OX=9913 GN=UGDH PE=1 SV=2

MFEIKKICCIGAGYVGGPTCSVIAHMCPEIRVTVVDINESRINAWNSPTLPIYEPGLKEV

VESCRGKNLFFSTNIDDAIKEADLVFISVNTPTKTYGMGKGRAADLKYIEACARRIVQNS

HGYKIVTEKSTVPVRAAESIRRIFDANTKPNLNLQVLSNPEFLAEGTAIKDLKNPDRVLI

GGDETPEGQRAVQALCAVYEHWVPREKILTTNTWSSELSKLTANAFLAQRISSINSISAL

CEATGADVEEVATAIGMDQRIGNKFLKASVGFGGSCFQKDVLNLVYLCEALNLPEVARYW

QQVIDMNDYQRRRFASRIIDSLFNTVTDKKIAILGFAFKKDTGDTRESSSIYISKYLMDE

GAHLHIYDPKVPREQIVVDLSHPGVSKDDQVARLVTISKDPYEACDGAHAVVICTEWDMF

KELDYERIHKKMLKPAFIFDGRRVLDGLHNELQTIGFQIETIGKKVSSKRIPYAPSGEIP

KFSLQDMPNKKPRV

>sp|P62739|ACTA_BOVIN Actin, aortic smooth muscle OS=Bos taurus OX=9913 GN=ACTA2 PE=1 SV=1

MCEEEDSTALVCDNGSGLCKAGFAGDDAPRAVFPSIVGRPRHQGVMVGMGQKDSYVGDEA

QSKRGILTLKYPIEHGIITNWDDMEKIWHHSFYNELRVAPEEHPTLLTEAPLNPKANREK

MTQIMFETFNVPAMYVAIQAVLSLYASGRTTGIVLDSGDGVTHNVPIYEGYALPHAIMRL

DLAGRDLTDYLMKILTERGYSFVTTAEREIVRDIKEKLCYVALDFENEMATAASSSSLEK

SYELPDGQVITIGNERFRCPETLFQPSFIGMESAGIHETTYNSIMKCDIDIRKDLYANNV

LSGGTTMYPGIADRMQKEITALAPSTMKIKIIAPPERKYSVWIGGSILASLSTFQQMWIS

KQEYDEAGPSIVHRKCF

>sp|Q3SZD7|CBR1_BOVIN Carbonyl reductase [NADPH] 1 OS=Bos taurus OX=9913 GN=CBR1 PE=2 SV=1

MSSSNCVALVTGANKGIGFVIVRDLCRRFSGDVVLTARDEARGRAAVQQLQAEGLSPLFH

QLDIDDRQSIRALRDFLRKEYGGLDVLVNNAGIAFKTADTTPFHIQAEVTMKTNFFGTRD

VCTELLPLIKPQGRVVNVSSFVSVNSLKKCSRELQQKFRSETITEEELVGLMNKFVEDTK

NGVHRKEGWPDTAYGVTKIGVTVLSRIHARKLSEQRGGDKILLNACCPGWVRTDMGGPKA

SKSPEEGAETPVYLALLPSDAEGPHGEFISEKRVVQW

>sp|P35445|COMP_BOVIN Cartilage oligomeric matrix protein OS=Bos taurus OX=9913 GN=COMP PE=1 SV=2

MVLAAARVLLLTLAALGASGQGQMPLGGDLGPQMLRELQETNAALQDVRDLLRQQVKEIT

FLKNTVMECDACGMQPARTPKLTVRPLSQCSPGFCFPGVACTETANGARCGPCPEGFTGN

GSHCADVNECTAHPCFPRVRCINTSPGFRCEACPPGFSGPTHEGVGLAFAKANKQVCTDI

NECETGQHNCVPNSVCVNTVGSFQCGPCQPGFVGDQASGCRRRPQRFCPDGTPSPCHEKA

DCVLERDGSRSCVCAVGWAGNGLICGRDTDLDGFPDEKLRCSERQCRKDNCVTVPNSGQE

DVDQDGIGDACDPDADGDGVLNEKDNCPLVRNPDQRNTDGDKWGDACDNCRSQKNDDQKD

TDKDGRGDACDDDIDGDRIRNPVDNCPKVPNSDQKDTDGDGVGDACDNCPQKSNADQRDV

DHDFVGDACDSDQDQDGDGHQDSKDNCPTVPNSAQQDSDHDGQGDACDDDDDNDGVPDSR

DNCRLVPNPGQEDMDRDGVGDACQGDFDADKVVDKIDVCPENAEVTLTDFRAFQTVVLDP

EGDAQIDPNWVVLNQGMEIVQTMNSDPGLAVGYTAFNGVDFEGTFHVNTATDDDYAGFIF

GYQDSSSFYVVMWKQMEQTYWQANPFRAVAEPGIQLKAVKSSTGPGEQLRNALWHTGDTA

SQVRLLWKDPRNVGWKDKTSYRWFLQHRPQVGYIRVRFYEGPELVADSNVILDTTMRGGR

LGVFCFSQENIIWANLRYRCNDTIPEDYEAQRLLQA

>sp|A7MBI7|COMT_BOVIN Catechol O-methyltransferase OS=Bos taurus OX=9913 GN=COMT PE=2 SV=1

MLEAPPLLLVAGGVGLALLALRWLATTDLQFFGRAFIVWNEFIMKPIRNLLMGSSKEQRI

LQHVLQHAVAGDPQSVVAAIDSYSLEKEWAMHVGEKKGQIVDRVLREQQPSVLLELGAYC

GYSAVRMARLLLPGARLLTIEFNPDYAAITQRMVEFAGLQDKVTVVLGASQDIIPQLKKK

YDVDTLDMVFLDHWKDRYLPDMLLLEECGLLREGTVLLADNVIYPGAPDFLEYVRGNSRF

ECSHFSSYLEYSKVVDGLEKVVYKGLSGPARP

>sp|O46415|FRIL_BOVIN Ferritin light chain OS=Bos taurus OX=9913 GN=FTL PE=2 SV=3

MSSQIRQNYSTEVEAAVNRLVNMQLRASYTYLSLGFYFDRDDVALEGVGHFFRELAKEKR

EGAERLLKLQNQRGGRALFLDVQKPSQDEWGKTQDAMEAALLVEKNLNQALLDLHGLASA

RGDPHICDFLENHFLDEEVKLIKKMGDHLTNLRRLAGPQAGLGEYLFERLTLKHD

>sp|Q3ZBD7|G6PI_BOVIN Glucose-6-phosphate isomerase OS=Bos taurus OX=9913 GN=GPI PE=2 SV=4

MAALTQNPQFKKLKTWYEQHGSDLNLRRLFEGDRDRFNRFSLNLNTNHGHILVDYSKNLV

TETVMQMLVDVAKSRGVEAARERMFTGEKINFTEDRAVLHVALRNRSNAPILVDGKDVMP

EVNRVLEKMKSFCQRVRSGEWKGYSGKAITDVINIGIGGSDLGPLMVTEALKPYSSEGPR

VWFVSNIDGTHIAKTLATLNPESSLFIIASKTFTTQETITNAETAKEWFLLSAKDPSAVA

KHFVALSTNTAKVKEFGIDPQNMFEFWDWVGGRYSLWSAIGLSIALHVGFDNFEQLLSGA

HWMDQHFRTTPLEKNAPVLLALLGIWYINCFGCETHAMLPYDQYLHRFAAYFQQGDMESN

GKYITKSGTRVNYQTGPIVWGEPGTNGQHAFYQLIHQGTKMIPCDFLIPVQSQHPIRNGL

HHKILLANFLAQTEALMRGKSTEEARKELQAAGRSPEDFEKLLPHKVFEGNRPTNSIVFT

KLTPFILGALIAMYEHKIFVQGIIWDINSFDQWGVELGKQLAKKIEPELDGSSPVTSHDS

STNGLINFIKQEREARS

>sp|O18879|GSTA2_BOVIN Glutathione S-transferase A2 OS=Bos taurus OX=9913 GN=GSTA2 PE=2 SV=4

MAGKPKLHYFNGRGRMECIRWLLAAAGVEFEEKFIEQPEDLDKLRNDGSLMFQQVPMVEI

DGMKLVQTRAILNYIATKYNLYGKDMKERALIDMYSEGVEDLGEMIMHLPLCPPDQKDAK

IAQIKERTTNRYFPAFEKVLKNHGQDYLVGNKLSKADIHLVELLYYVEELDPSLLANFPL

LKGLKARVSSLPAVKKFLQPGSQRKPPMDEKNLEEAKRIFRIK

>sp|P02070|HBB_BOVIN Hemoglobin subunit beta OS=Bos taurus OX=9913 GN=HBB PE=1 SV=1

MLTAEEKAAVTAFWGKVKVDEVGGEALGRLLVVYPWTQRFFESFGDLSTADAVMNNPKVK

AHGKKVLDSFSNGMKHLDDLKGTFAALSELHCDKLHVDPENFKLLGNVLVVVLARNFGKE

FTPVLQADFQKVVAGVANALAHRYH

>sp|Q3SZV7|HEMO_BOVIN Hemopexin OS=Bos taurus OX=9913 GN=HPX PE=2 SV=1

MARALRVPVALWLLGLCWSLAKAHPLARAPELGHGVEGGNVAKPDPEVTERCSDGWGFDA

TTLDEHGNMLFLKGEFVWKGHAWARQLISERWKDAPSPVDAAFRYDRNSVLLIKGDKFWV

YPPEKGEEYPKLLQEKFPGIPFPLDAAVECHRGECSHEGVFFFQGNHTWFWDFSTKTIKK

RSWPAVGNCSSAIRWLNRYYCFRGNKFLRFDPVTGEVNSTYPRDVRDYFMSCPNRGHAHR

NATQHMDKRCSPHLVLSALLSDNHSATYAFSENHYWRLDSSRDGWHSWRIEHLWPQGPST

VDAAFLWDKKLYLIQGTQVYIFLTRAGYTLVKDYPKQLEKEFGSPDGVCLHSVDAAFTCP

GSSQLYIMAGQKLWRLDLNLGAQATWTELPWLHTKVDGALCTEKSLGPHSCSANGLGLYL

VQGPNLYCYKDVEELSKTKDLPQAQRMNSLLGCAPHQHS

>sp|Q3SZR3|A1AG_BOVIN Alpha-1-acid glycoprotein OS=Bos taurus OX=9913 GN=ORM1 PE=2 SV=1

MALLWALAVLSHLPLLDAQSPECANLMTVAPITNATMDLLSGKWFYIGSAFRNPEYNKSA

RAIQAAFFYLEPRHAEDKLITREYQTIEDKCVYNCSFIKIYRQNGTLSKVESDREHFVDL

LLSKHFRTFMLAASWNGTKNVGVSFYADKPEVTQEQKKEFLDVIKCIGIQESEIIYTDEK

KDACGPLEKQHEEERKKETEAS

>sp|P62935|PPIA_BOVIN Peptidyl-prolyl cis-trans isomerase A OS=Bos taurus OX=9913 GN=PPIA PE=1 SV=2

MVNPTVFFDIAVDGEPLGRVSFELFADKVPKTAENFRALSTGEKGFGYKGSCFHRIIPGF

MCQGGDFTRHNGTGGKSIYGEKFDDENFILKHTGPGILSMANAGPNTNGSQFFICTAKTE

WLDGKHVVFGKVKEGMNIVEAMERFGSRNGKTSKKITIADCGQI

>sp|Q32KN8|TBA3_BOVIN Tubulin alpha-3 chain OS=Bos taurus OX=9913 GN=TUBA3 PE=2 SV=1

MRECISIHVGQAGVQIGNACWELYCLEHGIQPDGQMPSDKTIGGGDDSFNTFFSETGAGK

HVPRAVFVDLEPTVVDEVRTGTYRQLFHPEQLITGKEDAANNYARGHYTIGKEIVDLVLD

RIRKLADLCTGLQGFLIFHSFGGGTGSGFASLLMERLSVDYGKKSKLEFAIYPAPQVSTA

VVEPYNSILTTHTTLEHSDCAFMVDNEAIYDICRRNLDIERPTYTNLNRLIGQIVSSITA

SLRFDGALNVDLTEFQTNLVPYPRIHFPLATYAPVISAEKAYHEQLSVAEITNACFEPAN

QMVKCDPRHGKYMACCMLYRGDVVPKDVNAAIATIKTKRTIQFVDWCPTGFKVGINYQPP

TVVPGGDLAKVQRAVCMLSNTTAIAEAWARLDHKLDLMYAKRAFVHWYVGEGMEEGEFSE

AREDLAALEKDYEEVGVDSVEAEAEEGEEY

>sp|Q3Y5Z3|ADIPO_BOVIN Adiponectin OS=Bos taurus OX=9913 GN=ADIPOQ PE=1 SV=1

MLLQGALLLLLALPSHGEDNMEDPPLPKGACAGWMAGIPGHPGHNGTPGRDGRDGTPGEK

GEKGDPGLVGPKGDTGETGITGIEGPRGFPGTPGRKGEPGESAYVYRSAFSVGLERQVTV

PNVPIRFTKIFYNQQNHYDGTTGKFLCNIPGLYYFSYHITVYLKDVKVSLYKNDKALLFT

HDQFQDKNVDQASGSVLLYLEKGDQVWLQVYEGENHNGVYADNVNDSTFTGFLLYHNIVE

>sp|A6QR56|A16A1_BOVIN Aldehyde dehydrogenase family 16 member A1 OS=Bos taurus OX=9913 GN=ALDH16A1 PE=2 SV=1

MAATRTASRACEIFTTLEYGPAPESHACALAWLDTQDRHLGHYVNGQWLKPEHRSSVPCQ

DPITGENLASCLQAQSEDVAAAVEAARASLENWSTQPGAIRAQHLTRLAKVIQKHQRLLW

TLESLVTGRAVREVRDRDVPLAQQLLQYHAVQAHTQEEALAGWEPMGVIGLILSPTFPFL

DMMWRICPALAVGCTVVVLVPPASPTPLLLAQLAGELGPFPGILNVISGPASLGPVLAAQ

PGVQKVAFCGAIEEGRALRRTLAGWVPELGLALGAESLLLLTEVADVDSAVEGIVDAAWS

DRSPGGLRLLIQEAVWDETMRRLQERMGRLRCGHGLDGAVDMGARGAAARDLAQRYVSEA

QSQGAQVFQAGSEPSDSPFFPPTLVSDLPPASPCTQAEVPWPLVVASPFRTAKEALAVAN

GTPRGGSASVWSERLGQALELAYGLQVGTVWINAHGLRDPAVPTGGCKESGSSWHGGQDG

LYEYLRPSGTPAWIPYLSKTLNYDAFGLALPSTLPAGPETGPAPPYGLFVGGRFQAPGAR

SSRPIQDSQGSLQGYVAEGGAKDIRGAVEAAHQAAPGWMSQSPAARAALLWALAAALQRR

EPNLVSRLERHGVELKVAKAEVELSVKRLRAWGARVQAQGCALQVAELRGPVLRLREPLG

VLAIVCPDEWPLLAFVSLLAPALAHGNTVVLVPSGACPIPALEVCQEMATLLPAGLVNVV

TGDRDHLTRCLALHQDIQALWYFGSAQGSQFVEWASAGNLKPVWVNRGCPRAWDQEAEGA

GPELGRRAARTKALWLPMGD

>sp|P84080|ARF1_BOVIN ADP-ribosylation factor 1 OS=Bos taurus OX=9913 GN=ARF1 PE=1 SV=2

MGNIFANLFKGLFGKKEMRILMVGLDAAGKTTILYKLKLGEIVTTIPTIGFNVETVEYKN

ISFTVWDVGGQDKIRPLWRHYFQNTQGLIFVVDSNDRERVNEAREELMRMLAEDELRDAV

LLVFANKQDLPNAMNAAEITDKLGLHSLRHRNWYIQATCATSGDGLYEGLDWLSNQLRNQ

K

>sp|Q5EA01|B4GA1_BOVIN Beta-1,4-glucuronyltransferase 1 OS=Bos taurus OX=9913 GN=B4GAT1 PE=2 SV=2

MQMSYAIRCAFYQLLLAALMLVAMLQLLYLSLLSGLHGQEEQDQYFEFFPPSPRSVDQVK

AQLRTALASGGVLDASGDYRVYRGLLKTTMDPNDVILATHASVDNLLHLSGLLERWEGPL

SVSVFAATKEEAQLATVLTYALSSHCPDMRARVAMHLVCPSRYEAAVPDPREPGEFALLR

SCQEVFDKLARVAQPGVNYALGTNVSYPNNLLRNLAREGANYALVIDVDMVPSEGLWRSL

REMLDQSKQWAGTALVVPAFEIRRARRMPMNKNELLQLYQVGEVRPFYYGLCTPCQAPTN

YSRWVNLPEETLLRPAYVVPWQDPWEPFYVAGGKVPTFDERFRQYGFNRISQACELHVAG

FDFEVLNEGFLVHKGFKEVLKFHPQKEAENQHNKILYRQFKQELKAKYPDSPRHC

>sp|Q3MHN2|CO9_BOVIN Complement component C9 OS=Bos taurus OX=9913 GN=C9 PE=2 SV=1

MSAGQRFAFAICILEISLLRAGPTPSYDPAERQGTPLPIDCRMSSWSEWSKCDPCLKQMF

RSRSIEIFGQFNGRKCVDAVGDRQQCVPTEACEDPEEGCGNDFQCGTGRCIKNRLLCNED

NDCGDYSDEDNCEQDPRPPCRNRVVEESELARTAGFGINILGMDPLSTPFDNQYYNGLCD

RVWDGNTLTYYRRPWNVASLTYDTKADKNFRTENHEESIQILRTIIEEKKLNFNAGLSVK

YTPVEAIEKNKCVDLEHSDKGSTSSPSKLAAEAKFRFTYSKDDIYRLLSSYSAKQEKMFL

HVKGKVHLGRFVMRSRDVMLQTTFLDSINTLPTTYEKGEYFAFLETYGTHYSSSGSLGGL

YELIYVLDKKSMEQKDIELRDVQRCLGFDLDLSLKVGVEVTGNFDSKLCSKKGMGQTETN

PEADLFDDVITFIRGGTRKYATELKEKLLRGARMINVTDFVNWAASLNHAPVLISQKLVP

IYDLIPVKMKDAHLKKQNLERAIEDYINEFSVRKCQPCQNGGTVVLLDGECVCSCPKEFK

GVACEIKK

>sp|Q5E9F7|COF1_BOVIN Cofilin-1 OS=Bos taurus OX=9913 GN=CFL1 PE=2 SV=3

MASGVAVSDGVIKVFNDMKVRKSSTPEEVKKRKKAVLFCLSEDKKNIILEEGKEILVGDV

GQTVDDPYATFVKMLPDKDCRYALYDATYETKESKKEDLVFIFWAPECAPLKSKMIYASS

KDAIKKKLTGIKHELQANCYEEVKDRCTLAEKLGGSAVISLEGKPL

>sp|Q9XSC6|KCRM_BOVIN Creatine kinase M-type OS=Bos taurus OX=9913 GN=CKM PE=1 SV=2

MPFGNTHNKHKLNFKAEEEYPDLSKHNNHMAKALTLEIYKKLRDKETPSGFTLDDVIQTG

VDNPGHPFIMTVGCVAGDEESYTVFKDLFDPIIQDRHGGFKPTDKHKTDLNHENLKGGDD

LDPNYVLSSRVRTGRSIKGYALPPHCSRGERRAVEKLSVEALNSLTGEFKGKYYPLKSMT

EQEQQQLIDDHFLFDKPVSPLLLASGMARDWPDARGIWHNDNKSFLVWVNEEDHLRVISM

EKGGNMKEVFRRFCVGLQKIEEIFKKAGHPFMWNEHLGYVLTCPSNLGTGLRGGVHVKLA

HLSKHPKFEEILTRLRLQKRGTGGVDTAAVGSVFDVSNADRLGSSEVEQVQLVVDGVKLM

VEMEKKLEKGQSIDDMIPAQK

>sp|Q5E9B7|CLIC1_BOVIN Chloride intracellular channel protein 1 OS=Bos taurus OX=9913 GN=CLIC1 PE=2 SV=3

MAEEQPQVELFVKAGSDGAKIGNCPFSQRLFMVLWLKGVTFNVTTVDTKRRTETVQKLCP

GGQLPFLLYGTEVHTDTNKIEEFLEAVLCPPRYPKLAALNPESNTAGLDIFAKFSAYIKN

SNPALNDNLEKGLLKALKVLDNYLTSPLPDEVDETSAEDEGISQRKFLDGNELTLADCNL

LPKLHIVQVVCKKYRGFSIPDVFRGVHRYLRNAYAREEFASTCPDDEEIELAYEQVAKAL

K

>sp|Q2KIG3|CBPB2_BOVIN Carboxypeptidase B2 OS=Bos taurus OX=9913 GN=CPB2 PE=1 SV=1

MKLYSLGVLVATVLFCGEHAFAFQRGQVLSALPRTSRQVQILQNVTTTYKIVLWQPVAAE

YIVKGYEVHFFVNASDVSNVKAHLNASRIPFRVLVENVEDLIRQQTSNDTISPRASSSYY

EQYHSLNEIYSWIEVMTERYPDMVEKIHIGSSYEKYPLYVLKVSKKEQRAKNAMWIDCGI

HAREWISPAFCLWFVGSVTYYYGKEKMHTNLLKHMDFYIMPVVNVDGYDYTWKKDRMWRK

NRSLHEKNACVGTDLNRNFASKHWCGEGASSSSCSEIYCGTYPESEPEVKAVADFLRRNI

KHIKAYISMHSYSQKIVFPYSYSRSRSKDHEELSLVAREAVFAMENIHRNIRYTHGSGSE

SLYLAPGGSDDWIYDLGIKYSFTFELRDKGKYGFLLPESYIRPTCSEALVAVAKIASHVV

KNV

>sp|Q2KIA5|DNM1L_BOVIN Dynamin-1-like protein OS=Bos taurus OX=9913 GN=DNM1L PE=2 SV=1

MEALIPVINKLQDVFNTVGADIIQLPQIVVVGTQSSGKSSVLESLVGRDLLPRGTGIVTR

RPLILQLVHVAPEDKRKTTGEENDPATWKNSRHLSKGVEAEEWGKFLHTKNKLYTDFDEI

RQEIENETERISGNNKGVSPEPIHLKIFSPNVVNLTLVDLPGMTKVPVGDQPKDIELQIR

ELILRFISNPNSIILAVTAANTDMATSEALKISREVDPDGRRTLAVITKLDLMDAGTDAM

DVLMGRVIPVKLGIIGVVNRSQLDINNKKSVTDSIRDEYAFLQKKYPSLANRNGTKYLAR

TLNRLLMHHIRDCLPELKTRINVLAAQYQSLLNSYGEPVDDKSATLLQLITKFATEYCNT

IEGTAKYIETSELCGGARICYIFHETFGRTLESVDPLGGLNTIDILTAIRNATGPRPALF

VPEVSFELLVKRQIKRLEEPSLRCVELVHEEMQRIIQHCSNYSTQELLRFPKLHDAIVEV

VTCLLRKRLPVTNEMVHNLVAIELAYINTKHPDFADACGLMNNNIEEQRRNRLARELPSA

VSRDKSSKVPSALAPASQEPSPAASAEADGKLIQESRRETKNAASGGGGVGDAVQEPTTG

NWRGMLKTSKAEELLAEEKSKPIPIMPASPQKGHAVNLLDVPVPVARKLSAREQRDCEVI

ERLIKSYFLIVRKNIQDSVPKAVMHFLVNHVKDTLQSELVGQLYKSSLLDDLLTESEDMA

QRRKEAADMLKALQGASQIIAEIRETHLW

>sp|O77783|EXT2_BOVIN Exostosin-2 OS=Bos taurus OX=9913 GN=EXT2 PE=1 SV=1

MCASVKYNIRGPALIPRMKTKHRIYYITLFSIVLLGLIATGMFQFWPHSIESSGDWSVEK

RTGRDVPLVRLPADSPVPERGDLSCRMHTCFDVYRCGFNPKNKIKVYIYPLKKYVGEAGV

PVSSTISREYNELLTAISDSDYYTDDVTRACLFVPSIDLLNQNSLRVKETAQALAQLSRW

DRGTNHLLFNMLPGGPPDYNTALDVPRDRALLAGGGFSTWTYRQGYDVSIPVYSPLSAEV

DLPEKGPGPRRYFLLSSQVALHPEYREDLAALQARHGEAVLVLDKCSNLSEGVPAARRRC

HQQQAFDYPQVLQEATFCMVLRGARLGQAVLSDVLRAGCVPVIIADSYVLPFSEVLDWKR

ASVVVPEEKMSDVYSILQSIPRRQIEEMQRQARWFWEAYFQSIKAIALATLQIINDRIYP

YAAISYEDWNDPPAVKWGSVSNPLFLPLIPPQSQGFTAIVLTYDRVESLFRVITEVSKVP

SLSKLLVVWNNQNKNPPEDSLWPKIRVPLKVVRTAENKLSNRFFPYDEIETEAVLAIDDD

IIMLTSDELQFGYEVWREFPDRLVGYPGRLHLWDHEMNKWKYESEWTNEVSMVLTGAAFY

HKYFNYLYTYKMPGDIKNWVDAHMNCEDIAMNFLVANVTGKAVIKVTPRKKFKCPECTAI

DGLSLDQTHMVERSECINKFASVFGTMPLKVVEHRADPVLYKDDFPEKLKSFPNIGSL

>sp|Q5EA79|GALM_BOVIN Galactose mutarotase OS=Bos taurus OX=9913 GN=GALM PE=2 SV=1

MVSVTRAVFGDLPLGAGTVEKFQLQSDQLRVDIISWGCTITALEVKDRQGRASDVVLGFD

ELEGYLQKQPYFGAVVGRVANRIAKGTFTLDGKEYKLAINNGPNSLHGGVKGFDKVLWTP

RVLSNGVEFSRVSPDGEEGYPGELKVWVMYTLDGGELVVNYRAQASQTTPVNLTNHSYFN

LAGQGSPNIYDHEVTIEADAFLPVDEVLIPTGEIASVQGTAFDLRKPVELGKHLQEFHVN

GFDHNFCLKGSKEKRFCARVHHAGSGRVLEVYTTQPGVQFYTGNFLDGTLKGKSGAGYPK

HSGFCLETQSWPDAVNQPHFPPVLLKPGEEYDHTTWFKFSVA

>sp|Q0VCX2|BIP_BOVIN Endoplasmic reticulum chaperone BiP OS=Bos taurus OX=9913 GN=HSPA5 PE=2 SV=1

MKLSLVAAVLLLLLGTARAEEEDKKEDVGTVVGIDLGTTYSCVGVFKNGRVEIIANDQGN

RITPSYVAFTPEGERLIGDAAKNQLTSNPENTVFDAKRLIGRTWNDPSVQQDIKFLPFKV

VEKKTKPYIQVDVGGGQTKTFAPEEISAMVLTKMKETAEAYLGKKVTHAVVTVPAYFNDA

QRQATKDAGTIAGLNVMRIINEPTAAAIAYGLDKREGEKNILVFDLGGGTFDVSLLTIDN

GVFEVVATNGDTHLGGEDFDQRVMEHFIKLYKKKTGKDVRKDNRAVQKLRREVEKAKRAL

SSQHQARIEIESFYEGEDFSETLTRAKFEELNMDLFRSTMKPVQKVLEDSDLKKSDIDEI

VLVGGSTRIPKIQQLVKEFFNGKEPSRGINPDEAVAYGAAVQAGVLSGDQDTGDLVLLDV

CPLTLGIETVGGVMTKLIPRNTVVPTKKSQIFSTASDNQPTVTIKVYEGERPLTKDNHLL

GTFDLTGIPPAPRGVPQIEVTFEIDVNGILRVTAEDKGTGNKNKITITNDQNRLTPEEIE

RMVNDAEKFAEEDKKLKERIDTRNELESYAYSLKNQIGDKEKLGGKLSSEDKETMEKAVE

EKIEWLESHQDADIEDFKAKKKELEEIVQPIISKLYGSAGPPPTSEEEAADKDEL

>sp|Q0VCM5|ITIH1_BOVIN Inter-alpha-trypsin inhibitor heavy chain H1 OS=Bos taurus OX=9913 GN=ITIH1 PE=1 SV=1

MGLRGLLCVCLVSLLALQAVAAQGSPTRNPKGGKKRMAVDAAVDGVVIRSLKVNCKVTSR

FAHYIITSQVVNSADTAKEVSFNVEIPKTAFISDFAITADENAFTGDIKDKVTAWKQYRK

AAISGENAGLVRASGRTMEQFSIHIIVGPRSKATFRLTYEEVLRRKLMQYDIVIKVKPQQ

LVQHFEIDVDIFEPQGIRKLDVEASFLPKELAAQLIKKSFSGKKGHVLFRPTVSQQQTCP

TCSTTLLNGDFKVTYDVNRDDACDLLVANNYFAHFFAPQNLKKLNKNVVFVIDISSSMEG

QKLKQTKEALHKILGDMRPGDYFDLVLFGSAVQSWKGSLVQASPANLEAARNFVQQFSLA

GATNLNGGLLRGIEILNKAQQSLPELSNHASILIMLTDGEPTEGVMDRTQILKNVRDGIK

GRFPLYNLGFGHDVDLNFLEVMSLENNGRVQRIYEDHDATQQLQGFYEQVANPLLRDVEL

LYPREAVSDLTQHRHKQYYEGSEIMVAGRIADHKLSSFKADVRAHGEGQEFMTTCLVDKE

EMKKLLRERGHMLENHVERLWAYLTIQELLAKRMKLEGQEKANVSAKALQMSLAYQFVTP

LTSMTVRGMTDQDGLEPIIDKPLDDYLPLEMVGPRKTFMLQASQPAPTHSSLDIKKLPDQ

VTGVDTDPHFLIHVPQKEDTLCFNINEEPGVVLSLVQDPDTGFSVNGQLIGNEAGSPGKH

EGTYFGRLGIANPATDFQLEVTPQNITLNPGSGGPVFSWRDQAFLRQNEVLVTINRKRNL

VVSVEDGGTFEVVLHRVWRGSAVRQDFLGFYVLDSHRMSARTHGLLGQFFHPFDYKVSNL

HPGSDPTKTDATMVVKNRRLTVTRGLQKDYRKDPRHGAEVTCWFIHNNGDGLIDGIHTDY

IVPDIF

>sp|A1L595|K1C17_BOVIN Keratin, type I cytoskeletal 17 OS=Bos taurus OX=9913 GN=KRT17 PE=2 SV=1

MTTTIRHFSSGSIKGSSGLAGGSSRSCRVSGSLGGGSCRLGSAGGLGSGLGGSSYSSCYS

FGSGGGYGSGGYVSGGYGGGFGGVDGLLVGGEKATMQNLNDRLASYLDKVRALEEANTEL

ELKIRDWYQKQAPGPAPDYSSYFKTIEDLRNKIHTATVDNANLLLQIDNARLAADDFRTK

FETEQALRVSVEADINGLRRVLDELTLARADLEMQIENLKEELAYLRKNHEEEMKALRGQ

VGGEINVEMDAAPGVDLSRILNEMRDQYEKMAEKNRKDAEDWFFSKTEELNREVATNSEL

VQSGKSEISELRRTLQALEIELQSQLSMKASLEGSLAETENRYCMQLSQIQGLIGSVEEQ

LAQLRCEMEQQNQEYKILLDVKTRLEQEIATYRRLLEGEDAHLTQYKTKEPVTTRQVRTI

VEEVQDGRVISSREQVHQTSH

>sp|P19858|LDHA_BOVIN L-lactate dehydrogenase A chain OS=Bos taurus OX=9913 GN=LDHA PE=2 SV=2

MATLKDQLIQNLLKEEHVPQNKITIVGVGAVGMACAISILMKDLADEVALVDVMEDKLKG

EMMDLQHGSLFLRTPKIVSGKDYNVTANSRLVIITAGARQQEGESRLNLVQRNVNIFKFI

IPNIVKYSPNCKLLVVSNPVDILTYVAWKISGFPKNRVIGSGCNLDSARFRYLMGERLGV

HPLSCHGWILGEHGDSSVPVWSGVNVAGVSLKNLHPELGTDADKEQWKAVHKQVVDSAYE

VIKLKGYTSWAIGLSVADLAESIMKNLRRVHPISTMIKGLYGIKEDVFLSVPCILGQNGI

SDVVKVTLTHEEEACLKKSADTLWGIQKELQF

>sp|Q0VCM4|PYGL_BOVIN Glycogen phosphorylase, liver form OS=Bos taurus OX=9913 GN=PYGL PE=2 SV=1

MAKPLTDQEKRRQISIRGIVGVENVAELKKGFNRHLHFTLVKDRNVATPRDYFFALAHTV

RDHLVGRWIRTQQYYYEKCPKRVYYLSLEFYMGRTLQNTMINLGLQNACDEAIYQLGLDM

EELEEIEEDAGLGNGGLGRLAACFLDSMATLGLAAYGYGIRYEYGIFNQKIRDGWQIEEA

DDWLRHGNPWEKARPEFMLPVHFYGRVEHTEAGTKWTDTQVVLALPYDTPVPGYLNNTVN

TMRLWSARAPNDFNLRDFNVGDYIQAVLDRNLAENISRVLYPNDNFFEGKELRLKQEYFV

VAATLQDVIRRFKASKFDSSNSTKTAFDAFPDQVAIQLNDTHPSLAIPELMRIFVDIEKL

PWSKAWEITQKTFAYTNHTVLPEALERWPVELVEKLLPRHLQIIYEINQKHLDKIAALFP

KDVDRLRRMSLIEEEGGKRINMAHLCIVGSHAVNGVAKIHSDIVKTQVFKDFSELEPDKF

QNKTNGITPRRWLLLCNPGLAELIAEKIGEDYVKDLSQLTKLNSFLGDDIFLREISNVKQ

ENKLKFSQFLEKEYKVKINPSSMFDVQVKRIHEYKRQLLNCLHVVTMYNRIKKDPKKLFV

PRTVIIGGKAAPGYYMAKLIIKLITSVAEVVNNDPVVGSKLKLIFLENYRVSLAEKVIPA

TDLSEQISTAGTEASGTGNMKFMLNGALTIGTMDGANVEMAEEAGEENLFIFGMRIEDVA

ALDKKGYEAKEYYEALPELKLAIDQIDKGFFSPKQPDLFKDLVNMLFYHDRFKVFADYEA

YVKCQEKVSQLYMNPKAWNIMVLKNIAASGKFSSDRTIKEYARDIWNMEPSDIKISLSSD

PSGGANKANGK

>sp|Q3T054|RAN_BOVIN GTP-binding nuclear protein Ran OS=Bos taurus OX=9913 GN=RAN PE=2 SV=3

MAAQGEPQVQFKLVLVGDGGTGKTTFVKRHLTGEFEKKYVATLGVEVHPLVFHTNRGPIK

FNVWDTAGQEKFGGLRDGYYIQAQCAIIMFDVTSRVTYKNVPNWHRDLVRVCENIPIVLC

GNKVDIKDRKVKAKSIVFHRKKNLQYYDISAKSNYNFEKPFLWLARKLIGDPNLEFVAMP

ALAPPEVVMDPALAAQYEHDLEVAQTTALPDEDDDL

>sp|Q9TTJ5|RGN_BOVIN Regucalcin OS=Bos taurus OX=9913 GN=RGN PE=2 SV=1

MSSIKIECVLRENCHCGESPVWEEASNSLLFVDIPAKKVCRWDSLSKQVQRVTVDAPVSS

VALRQSGGYVATVGTKFCALNWEDQSAVVLATVDKEKKNNRFNDGKVDPAGRYFAGTMAE

ETAPAVLERRQGSLYSLFPDHHVEKYFDQVDISNGLDWSMDHKIFYYIDSLSYSVDAFDY

DLQTGKISNRRSVYKLEKEEQIPDGMCIDVEGKLWVACYNGGRVIRLDPETGKRLQTVKL

PVDKTTSCCFGGKDYSEMYVTCARDGLDSKGLLQQPEAGGIFKITGLGVKGIPPYPYTG

>sp|E1BF81|CBG_BOVIN Corticosteroid-binding globulin OS=Bos taurus OX=9913 GN=SERPINA6 PE=3 SV=1

MLPTLYTCLLWLSTSGLWTVQAKGSDTDMSTRNPHRDLAPNNVDFAFTLYKHLVASAPGK

NVFISPVSISTALAMLSLGARGYTREQLLQGLGFNLTEMSEGEIHRAFRHLHHLLRESNT

TLDMTMGNALFLDHSLELLESFSADTKHYYELEALTTDFQDWAGASRQINEYIKNKTQGN

IVDLFSESDSSATLILVNYIFFKGMWAHSFDLESTREENFHVNEATTVQVPMMFQSNTIK

YLNDSVLPCQLVQLDYTGNETVFFVLPVKGKMDSVITALSRDTIQRWSKSLTMSQVDLYI

PKISISGAYDLGGIMGDMGIADLLSNRTHFSGITQEALPKVSKVVHKAALQVDEKGLEAD

APTRVSLSAAPGPLTLRFNRPFIIMIFDDFTWSSLFLGKVVNPT

>sp|Q95JC7|AAAT_BOVIN Neutral amino acid transporter B(0) OS=Bos taurus OX=9913 GN=SLC1A5 PE=2 SV=1

MVADPPKGDPKGYAAAEPTANGVSMLVPIEDVGSLKGGRCGSGDQVRRCLRANLLVLLTV

VAVVAGVALGLGVSGAGGAFALGPARLEAFSFPGELLLRLLKMIILPLVVCSLIGGAASL

DPSALGRLGAWALLFFLVTTLLASALGVGLALALQPGAAFAAINTSVGAPVEEAPSKEVL

DSFLDLVRNIFPSNLVSAAFRSYTTSYKERLFNGTLVKVPTGGEVEGMNILGLVVFAIIF

GVALRKLGPEGELLIRFFNSFNDATMVLVSWIMWYAPVGILFLVAGKIVEMENVGLLFAS

LGKYILCCLLGHAIHGLLTLPLIYFLFARKNPYRFLWGIMTPLATAFGTSSSSATLPLMM

KCVEEKNGVARHISRFILPIGATVNMDGAALFQCVAAVFIAQLNHRSLDFVKIITILVTA

TASSVGAAGIPSGGVLTLAIILEAVNLPVHDISLILAVDWLVDRSCTVLNVEGDAFGAGL

LQSYLDRTENCNSVPELIQVKSEMPLAALPVPGEEGNPLLKGCPGPAGDADTCEKESVM

>sp|Q3ZCJ7|TBA1C_BOVIN Tubulin alpha-1C chain OS=Bos taurus OX=9913 GN=TUBA1C PE=1 SV=1

MRECISIHVGQAGVQIGNACWELYCLEHGIQPDGQMPSDKTIGGGDDSFNTFFSETGAGK

HVPRAVFVDLEPTVIDEVRTGTYRQLFHPEQLISGKEDAANNYARGHYTIGKEIIDLVLD

RVRKLADQCTGLQGFLVFHSFGGGTGSGFTSLLMERLSVDYGKKSKLEFSIYPAPQVSTA

VVEPYNSILTTHTTLEHSDCAFMVDNEAIYDICRRNLDIERPTYTNLNRLMSQIVSSITA

SLRFDGALNVDLTEFQTNLVPYPRIHFPLATYAPVISAEKAYHEQLSVAEITNACFEPAN

QMVKCDPRHGKYMACCLLYRGDVVPKDVNAAIATIKTKRTIQFVDWCPTGFKVGINYQPP

TVVPGGDLAKVQRAVCMLSNTTAVAEAWARLDHKFDLMYAKRAFVHWYVGEGMEEGEFSE

AREDMAALEKDYEEVGADSAEGDDEGDEY

>sp|P16116|ALDR_BOVIN Aldo-keto reductase family 1 member B1 OS=Bos taurus OX=9913 GN=AKR1B1 PE=1 SV=2

AHNIVLYTGAKMPILGLGTWKSPPGKVTEAVKVAIDLGYRHIDCAHVYQNENEVGLALQA

KLQEKVVKREDLFIVSKLWCTYHDKDLVKGACQKTLSDLKLDYLDLYLIHWPTGFKPGKD

FFPLDEDGNVIPSEKDFVDTWTAMEELVDEGLVKAIGVSNFNHLQVEKILNKPGLKYKPA

VNQIECHPYLTQEKLIQYCNSKGIVVTAYSPLGSPDRPWAKPEDPSILEDPRIKAIADKY

NKTTAQVLIRFPIQRNLIVIPKSVTPERIAENFQVFDFELDKEDMNTLLSYNRDWRACAL

VSCASHRDYPFHEEF

>sp|P17690|APOH_BOVIN Beta-2-glycoprotein 1 OS=Bos taurus OX=9913 GN=APOH PE=1 SV=4

MPPPALVLLLGFLCHVAIAGRTCPKPDELPFSTVVPLKRTYEPGEQIVFSCQPGYVSRGG

IRRFTCPLTGLWPINTLKCMPRVCPFAGILENGTVRYTTFEYPNTISFSCHTGFYLKGAS

SAKCTEEGKWSPDLPVCAPITCPPPPIPKFASLSVYKPLAGNNSFYGSKAVFKCLPHHAM

FGNDTVTCTEHGNWTQLPECREVRCPFPSRPDNGFVNHPANPVLYYKDTATFGCHETYSL

DGPEEVECSKFGNWSAQPSCKASCKLSIKRATVIYEGERVAIQNKFKNGMLHGQKVSFFC

KHKEKKCSYTEDAQCIDGTIEIPKCFKEHSSLAFWKTDASDVKPC

>sp|Q5E9I6|ARF3_BOVIN ADP-ribosylation factor 3 OS=Bos taurus OX=9913 GN=ARF3 PE=2 SV=3

MGNIFGNLLKSLIGKKEMRILMVGLDAAGKTTILYKLKLGEIVTTIPTIGFNVETVEYKN

ISFTVWDVGGQDKIRPLWRHYFQNTQGLIFVVDSNDRERVNEAREELMRMLAEDELRDAV

LLVFANKQDLPNAMNAAEITDKLGLHSLRHRNWYIQATCATSGDGLYEGLDWLANQLKNK

K

>sp|Q29RU4|CO6_BOVIN Complement component C6 OS=Bos taurus OX=9913 GN=C6 PE=2 SV=1

MARHSVMYFILLSALIDKSQACFCDHYPWSQWSSCSKTCNSGTQTRQRRIVTDKYYFENF

CGQLCTKQESRECNWQTCPINCRLGDYGPWSDCDPCVQKRFKVRSILRPSQFGGQPCTEP

LMTFQPCIPSKLCKIEEIDCKNKFRCDSGRCIASKLECNGENDCGDNSDERNCGRKKTVC

SRSHNPIPGVQLMGMGFHFLAGEPRGEVLDNSFTGGVCRTVKSSRASNPYRVPANLENVN

FEVQTKEDDLEADFYDDLIPLEDNKDQEALGSGLATSSFRVPIFYSSKRSQSSSHSSAFK

QAIQASQKKASSFIRIHKVIKVLNFTMKTKDLQLSDVFLKALNHLPLEYNSALYSRIFDD

FGTHYFTSGSLGGVYDLLYQFSKEELKNSGLTQEEAKNCIRIETKKRFLFVKKTKVEHRC

TTNKLSEKYEGSFMQGSEKSISLVQGGRSAYAAALAWEKGSPVPEERVFSDWLESVKENP

SVIDFALAPITDLVRNIPCAVTRRNNLRRAFREYAAKFDPCQCARCPNSGRPVLSGTECL

CVCQSGTYGENCERRSPDYKSNAVDGNWGCWSSWSSCDATYRRSRTRECNNPAPQQGGKR

CEGERRQEEHCTFSIMQNDGQPCISDDEDMKETDLPELESDSGCPQPVPPENGFIRNEKK

QYSVGEEVEILCFTGFKAVGYQYFRCLPDRSWRQGDVECQRTECLKPIVPEGLTLSPFQT

LYKIGDSIELTCPRGLVVNGPSRYTCSGDSWTPPISDSLSCEKDVLTGLRGHCQPGQKQL

GSECVCMSPEEDCGHYSEEICVLDTTSSDYFTSSACKLLAEKCLNNQQLHFVHIGSCEEG

PQLKWGLERIKLSSSSTKNESCGYDTCYNWEKCSATTSKCVCLLPSQCTKGGDQLFCVQI

GSSANGKTMNICEVGAVKCAKREMEILHSGRC

>sp|Q95M17|CHIA_BOVIN Acidic mammalian chitinase OS=Bos taurus OX=9913 GN=CHIA PE=1 SV=1

MAKLIFLTGLAFLLNAQLGSAYQLVCYFSNWAQYRPGLGSFKPDNIDPCLCTHLIYAFAG

MSNSEITTIEWNDVALYSSFNDLKKKNSQLKILLAIGGWNFGTAPFTAMVATPENRKTFI

SSVIKFLHQYGFDGLDFDWEYPGFRGSPSQDKHLFTVLVQETREAFEQEAKQTNKPRLLV

TAAVAAGISNIQAGYEIPQLSQYLDFIHVMTYDFHGSWEGYTGENSPLYKYPTDTGSNTY

LNVEYAMNYWKKNGAPAEKLIIGFPAYGHNFILRDASNNGIGAPTSGAGPAGPYTREAGF

WAYYEICAFLKDGATEAWDDSQNVPYAYKGTEWVGYDNVNSFRIKAQWLKENNFGGAMVW

AIDLDDFTGTFCNQGKFPLINTLKDALGLKSATCNASTQSSEPNSSPGNESGSGNKSSSS

EGRGYCAGKADGLYPVADNRNAFWNCVNGITYKQNCLTGLVFDTSCHCCNWA

>sp|P00741|FA9_BOVIN Coagulation factor IX OS=Bos taurus OX=9913 GN=F9 PE=1 SV=2

MWCLNMIMAESPGLVTICLLGYLLSAECTVFLDRENATKILHRPKRYNSGKLEEFVRGNL

ERECKEEKCSFEEAREVFENTEKTTEFWKQYVDGDQCESNPCLNGGMCKDDINSYECWCQ

AGFEGTNCELDATCSIKNGRCKQFCKRDTDNKVVCSCTDGYRLAEDQKSCEPAVPFPCGR

VSVSHISKKLTRAETIFSNTNYENSSEAEIIWDNVTQSNQSFDEFSRVVGGEDAERGQFP

WQVLLHGEIAAFCGGSIVNEKWVVTAAHCIKPGVKITVVAGEHNTEKPEPTEQKRNVIRA

IPYHSYNASINKYSHDIALLELDEPLELNSYVTPICIADRDYTNIFLKFGYGYVSGWGKV

FNRGRSASILQYLKVPLVDRATCLRSTKFSIYSHMFCAGYHEGGKDSCQGDSGGPHVTEV

EGTSFLTGIISWGEECAMKGKYGIYTKVSRYVNWIKEKTKLT

>sp|P50448|F12AI_BOVIN Factor XIIa inhibitor OS=Bos taurus OX=9913 PE=1 SV=1

MASRLTPLTLLLLLLLAGDRVTSDMIVGPGNLQEGESEGDSQKGGILDGESIQGNEDSPT

LPITNLTVVPATVTKPFSQPATEPVQSTIQPTAEPFCLAPVTSCSDSEIRSAEAVLGEAL

TDFSLRLYQDFSVLKKRETNFIFSPFSIASLLTQILLGAGGETRVSLEHLLSYPQNFSCV

HHALRAFMSEGFTSFSQIFHSSDLTIKDTFAEASQRLYGSSPRPLGNDSTASLELINDWV

AKKTNLRIRRLLDSLPEDTRLILLNAVALSAKWKIAFDKGRTSTKPFHLKSSAIKVPMMN

SKKYPVASFTDRTLNRPGGRLQLSHNLSFVILVPQTVKHHLQDLEQALSTAVFKAVIKKL

EMTKFHPTHLTMPRIKVQSSQDMLDYFDFIYDVNLCGLTEDPDVQVSGIRHQATLELTES

GVDATAASVVSVARNLLLFEVQQPFLFLLWDQQHKFPVFMGRVYDPKG

>sp|P02676|FIBB_BOVIN Fibrinogen beta chain OS=Bos taurus OX=9913 GN=FGB PE=1 SV=2

QFPTDYDEGQDDRPKVGLGARGHRPYDKKKEEAPSLRPVPPPISGGGYRARPATATVGQK

KVERKPPDADGCLHADPDLGVLCPTGCKLQDTLVRQERPIRKSIEDLRNTVDSVSRTSSS

TFQYITLLKNMWKGRQNQVQDNENVVNEYSSHLEKHQLYIDETVKNNIPTKLRVLRSILE

NLRSKIQKLESDVSTQMEYCRTPCTVTCNIPVVSGKECEKIIRNEGETSEMYLIQPEDSS

KPYRVYCDMKTEKGGWTVIQNRQDGSVDFGRKWDPYKQGFGNIATNAEGKKYCGVPGEYW

LGNDRISQLTNMGPTKLLIEMEDWKGDKVTALYEGFTVQNEANKYQLSVSKYKGTAGNAL

IEGASQLVGENRTMTIHNSMFFSTYDRDNDGWKTTDPRKQCSKEDGGGWWYNRCHAANPN

GRYYWGGAYTWDMAKHGTDDGVVWMNWQGSWYSMKKMSMKIRPYFPEQ

>sp|P21856|GDIA_BOVIN Rab GDP dissociation inhibitor alpha OS=Bos taurus OX=9913 GN=GDI1 PE=1 SV=1

MDEEYDVIVLGTGLTECILSGIMSVNGKKVLHMDRNPYYGGESSSITPLEELYKRFQLLE

GPPETMGRGRDWNVDLIPKFLMANGQLVKMLLYTEVTRYLDFKVVEGSFVYKGGKIYKVP

STETEALASNLMGMFEKRRFRKFLVFVANFDENDPKTFEGVDPQNTSMRDVYRKFDLGQD

VIDFTGHALALYRTDDYLDQPCLETINRIKLYSESLARYGKSPYLYPLYGLGELPQGFAR

LSAIYGGTYMLNKPVDDIIMENGKVVGVKSEGEVARCKQLICDPSYVPDRVRKAGQVIRI

ICILSHPIKNTNDANSCQIIIPQNQVNRKSDIYVCMISYAHNVAAQGKYIAIASTTVETT

DPEKEVEPALELLEPIDQKFVAISDLYEPIDDGSESQVFCSCSYDATTHFETTCNDIKDI

YKRMAGSAFDFENMKRKQNDVFGEADQ

>sp|P37141|GPX3_BOVIN Glutathione peroxidase 3 OS=Bos taurus OX=9913 GN=GPX3 PE=2 SV=2

MARLFRASCLLSLLLAGFIPPSQGQEKSKTDCHAGVGGTIYEYGALTIDGEEYIPFKQYA

GKYILFVNVASYUGLTGQYVELNALQEELEPFGLVILGFPCNQFGKQEPGENSEILATLK

YVRPGGGFTPNFQLFEKGDVNGEKEQKFYTFLKNSCPPTSELLGSPDRLFWEPMKVHDIR

WNFEKFLVGPDGIPIMRWYHRTTVNSVKMDILTYMRRRAVWEAKGK

>sp|P19879|MIME_BOVIN Mimecan OS=Bos taurus OX=9913 GN=OGN PE=1 SV=2

MKTLQSTLLLFLFVPLIKPAPPSQQDSRIIYDYGTDNLEETFFSQDYEDKYLDGKSTKEK

ETMIIVPDEKSFQLQKDENITPLPPKKENDEMPTCLLCVCLSGSVYCEEVDIDAVPPLPK

ESAYLYARFNKIKKLTAKDFADIPNLRRLDFTGNLIEDIEDGTFSKLSLLEELTLAENQL

LKLPVLPPKLTLFNAKYNKIKSRGIKANTFKKLHNLSFLYLDHNALESVPLNLPESLRVI

HLQFNNITSITDDTFCKANDTSYIRDRIEEIRLEGNPVILGKHPNSFICLKRLPIGSYI

>sp|P02584|PROF1_BOVIN Profilin-1 OS=Bos taurus OX=9913 GN=PFN1 PE=1 SV=2

MAGWNAYIDNLMADGTCQDAAIVGYKDSPSVWAAVPGKTFVNITPAEVGILVGKDRSSFF

VNGLTLGGQKCSVIRDSLLQDGEFTMDLRTKSTGGAPTFNITVTMTAKTLVLLMGKEGVH

GGMINKKCYEMASHLRRSQY

>sp|P61223|RAP1B_BOVIN Ras-related protein Rap-1b OS=Bos taurus OX=9913 GN=RAP1B PE=2 SV=1

MREYKLVVLGSGGVGKSALTVQFVQGIFVEKYDPTIEDSYRKQVEVDAQQCMLEILDTAG

TEQFTAMRDLYMKNGQGFALVYSITAQSTFNDLQDLREQILRVKDTDDVPMILVGNKCDL

EDERVVGKEQGQNLARQWNNCAFLESSAKSKINVNEIFYDLVRQINRKTPVPGKARKKSS

CQLL

>sp|A2I7N3|SPA37_BOVIN Serpin A3-7 OS=Bos taurus OX=9913 GN=SERPINA3-7 PE=3 SV=1

MRTERTSFLLALGLLVSGFCSRVHCLPENVTPEEQHKGTSVDGHSLASSNTDFALSLYKQ

LALKDPNKNVIFSPLSISIALAFLSLGAHDHTVTEILEGLKFNLTETPETEIHQGFQHLL

QTFNQPSNQLQLSVGNAMFASEELKLLDKFRKDAEAFYASEVLSTNFKDSEAAVKLINEY

VKNKTHGKIEKLFNDLDVLTNLILLNYIFFKAQWKTPFNPNHTYESEFHVSQNERVIVPM

MTLYLETPYFRDEELGCTLVELTYTSNDSALFILPDEGKMQDLEAKLTPETLTRWRSSLQ

PRLIHRLRLPRFSISSHYQLKDILSQLGIKKIFTSDAGFSGITDDHKLAVSHVIHKAVLD

VGEEGTEGAAVTAVVMATSSLLHTLTVSFNRPFLLSIFCKETQSIIFWGKVTNPKEA

>sp|Q5E9P9|GLYC_BOVIN Serine hydroxymethyltransferase, cytosolic OS=Bos taurus OX=9913 GN=SHMT1 PE=2 SV=3

MAAPVNKAPRDANLWSLHEKMLAQPLKDNDVEVYNIIKKESNRQRVGLELIASENFASRA

VLEALGSCLNNKYSEGYPGQRYYGGTEFIDELEVLCQKRALQVYGLDSQCWGVNVQPYSG

SPANFAVYTALVEPHGRIMGLDLPDGGHLTHGFMTDKKKISATSIFFESMPYKVNPDTGY

INYDQLEENARLFHPRLIIAGTSCYSRNLDYARLRKIADDNGAYLMADMAHVSGLVAAGV

VPSPFEHCHVVSTTTHKTLRGCRAGMIFYRKGVRSVDPKTGRETRYNLESLINSAVFPGL

QGGPHNHAIAGVAVALKQAMTPEFRAYQRQVVANCRALAEALMGLGYRVVTGGSDNHLIL

VDLRSKGTDGGRAEKVLEACSIACNKNTCPGDKSALRPSGLRLGTPALTSRGLLEEDFQK

VAHFIHRGIELTLQIQDAVGVKATLKEFMEKLAGAEEHHRAVAALRAEVESFATLFPLPG

LPGF

>sp|P19217|ST1E1_BOVIN Sulfotransferase 1E1 OS=Bos taurus OX=9913 GN=SULT1E1 PE=1 SV=1

MSSSKPSFSDYFGKLGGIPMYKKFIEQFHNVEEFEARPDDLVIVTYPKSGTTWLSEIICM

IYNNGDVEKCKEDVIFNRVPYLECSTEHVMKGVKQLNEMASPRIVKSHLPVKLLPVSFWE

KNCKIIYLSRNAKDVVVSYYFLILMVTAIPDPDSFQDFVEKFMDGEVPYGSWFEHTKSWW

EKSKNPQVLFLFYEDMKENIRKEVMKLLEFLGRKASDELVDKIIKHTSFQEMKNNPSTNY

TTLPDEVMNQKVSPFMRKGDVGDWKNHFTVALNEKFDMHYEQQMKGSTLKFRTKI

>sp|Q2TA49|VASP_BOVIN Vasodilator-stimulated phosphoprotein OS=Bos taurus OX=9913 GN=VASP PE=2 SV=3

MSETVVCTSRATVMLYDDSNKRWLPAGTGPQAFSRVQIYHNPTANSFRVVGRKMQPDQQV

VINCAIVRGVKYNQATPNFHQWRDARQVWGLNFGSKEDATQFANGMASALEALEGGGPLP

PPPPTAPPTWSAQNGPSPEEMEQQKRQQQSELMERERRASNAGGPPAASAGAPPPPPGPP

PPPGPPPPPGLSSSGVSAATQGAGGGPPPAPPLPTAQGPSGGGTGAPSLASAIAGAKLRK

VSKQEEASAGPVAPKAESSRSTGGGLMEEMNAMLARRRKATQVGEKPAKDESANQEESDA

RVPAHSESVRRPWEKNSTTLPRMKSSSSVTTSEAHPATPSSSDESDLERVKQELLEEVRK

ELQKVKEEIIEAFVQELRKRGAP

>sp|Q32PF2|ACLY_BOVIN ATP-citrate synthase OS=Bos taurus OX=9913 GN=ACLY PE=2 SV=1

MSAKAISEQTGKELLYKYICTTSAIQNRFKYARVTPDTDWARLLQDHPWLLSQSLVVKPD

QLIKRRGKLGLIGVNLTLDGVKSWLKPRLGQEATVGKATGFLKNFLIEPFVPHTQEEEFY

VCIYATREGDYVLFHHEGGVDVGDVDAKAQKLLVGVDEKLNPEDIKKHLLVHAPEDKKEI

LASFISGLFNFYEDLYFTYLEINPLVVTKDGVYVLDLAAKVDATADYICKVKWGDIEFPP

PFGREAYPEEAYIADLDAKSGASLKLTLLNPKGRIWTMVAGGGASVVYSDTICDLGGVNE

LANYGEYSGAPSEQQTYDYAKTILSLMTREKHPDGKILIIGGSIANFTNVAATFKGIVRA

IRDYQGPLKEHEVTIFVRRGGPNYQEGLRVMGEVGKTTGIPIHVFGTETHMTAIVGMALG

HRPIPNQPPTAAHTANFLLNASGSTSTPAPSRTASFSESRTDEVAPAKKAKPAMLQGKSA

TLFSRHTKAIVWGMQTRAVQGMLDFDYVCSRDEPSVAAMVYPFTGDHKQKFYWGHKEILI

PVFKNMADAMKKHPEVDVLINFASLRSAYDSTMETMNYAQIRTIAIIAEGIPEALTRKLI

KKADQKGVTIIGPATVGGIKPGCFKIGNTGGMLDNILASKLYRPGSVAYVSRSGGMSNEL

NNIISRTTDGVYEGVAIGGDRYPGSTFMDHVLRYQDTAGVKMIVVLGEIGGTEEYKICRG

VTEGRITKPVVCWCIGTCAAMFSSEVQFGHAGACANQASETAVAKNQALKEAGVFVPRSF

DELGEIIQSVYEDLVARGVIVPAQEVPPPTVPMDYSWARELGLIRKPASFMTSICDERGQ

ELIYAGMPITEVFKEEMGIGGVLGLLWFQKRLPKYSCQFIEMCLMVTADHGPAVSGAHNT

IICARAGKDLVSSLTSGLLTIGDRFGGALDAAAKMFSKAFDSGIIPMEFVNKMKKEGKLI

MGIGHRVKSINNPDMRVQILKDYVRQHFPATPLLDYALEVEKITTSKKPNLILNVDGLIG

VAFVDMLRHCGSFTREEADEYIDIGALNGIFVLGRSMGFIGHYLDQKRLKQGLYRHPWDD

ISYVLPEHMSM

>sp|P61157|ARP3_BOVIN Actin-related protein 3 OS=Bos taurus OX=9913 GN=ACTR3 PE=1 SV=3

MAGRLPACVVDCGTGYTKLGYAGNTEPQFIIPSCIAIKESAKVGDQAQRRVMKGVDDLDF

FIGDEAIEKPTYATKWPIRHGIVEDWDLMERFMEQVIFKYLRAEPEDHYFLLTEPPLNTP

ENREYTAEIMFESFNVPGLYIAVQAVLALAASWTSRQVGERTLTGTVIDSGDGVTHVIPV

AEGYVIGSCIKHIPIAGRDITYFIQQLLRDREVGIPPEQSLETAKAVKERYSYVCPDLVK

EFNKYDTDGSKWIKQYTGINAISKKEFSIDVGYERFLGPEIFFHPEFANPDFTQPISEVV

DEVIQNCPIDVRRPLYKNIVLSGGSTMFRDFGRRLQRDLKRTVDARLKLSEELSGGRLKP

KPIDVQVITHHMQRYAVWFGGSMLASTPEFYQVCHTKKDYEEIGPSICRHNPVFGVMS

>sp|Q3ZC42|ADHX_BOVIN Alcohol dehydrogenase class-3 OS=Bos taurus OX=9913 GN=ADH5 PE=2 SV=1

MANQVIKCKAAVAWEAGKPLSIEEVEVAPPKAHEVRIKIIATAVCHTDAYTLSGADPEGN

YPVILGHEGAGIVESVGEGVTKLKAGDTVIPLYIPQCGECKFCLNPKTNLCQKIRVTQGK

GLMPDGTSRFTCKGKTILHYMGTSTFSEYTVVADISVAKIDPLAPLDKVCLLGCGISTGY

GAALNAAKVEPGSTCAVFGLGGVGLAVIMGCKMAGAARIIGVDINKDKFARAKEFGASEC

INPQDFSKPIQEVLIEMTDGGVDYSFECIGNVKVMRAALEACHKGWGISVVVGVAASGEE

IATRPFQLVTGRTWKGTAFGGWKSVESVPKLVSEYMSKKIKVDEFVTHSLPFDQINEAFD

LMHAGKSIRTVVKL

>sp|A3KN12|PUR8_BOVIN Adenylosuccinate lyase OS=Bos taurus OX=9913 GN=ADSL PE=2 SV=1

MAAAGDRGGREAACGHDSYRSPLASRYASPEMCFLFSDKYKFRTWRQLWLWLAEAEQTLG

LPITDEQIQEMKSNLDNIDFRMAAEEEKQLRHDVMAHVHTFAHCCPKAASIIHLGATSCY

VGDNTDLIILRNAFDLLLPKLARVISRLADFAKEQADLPTLGFTHFQPAQLTTVGKRCCL

WIQDLCMDLQNLKRVRDELRFRGVKGTTGTQASFLQLFEGDDQKVEQLDKMVTEKAGFKR

AFIITGQTYTRKVDIEVLSVLASLGASVHKICTDIRLLANLKEMEEPFEKQQIGSSAMPY

KRNPMRSERCCSLARHLMALVMDPLQTASVQWFERTLDDSANRRICLAEAFLTADTVLNT

LQNISEGLVVYPKVIERRVQQELPFMATENIIMAMVKAGGNRQDCREKIRVLSQQAAAVV

KQEGGDNDLIERIQADAYFSPIHSQLDHLLDPSSFTGRASQQVQRFLEEEVCPLLKPYES

VMKVKAELRL

>sp|Q0P5F9|AL8A1_BOVIN 2-aminomuconic semialdehyde dehydrogenase OS=Bos taurus OX=9913 GN=ALDH8A1 PE=2 SV=1

MAGRGGLLMLENFIGGKFLPCSSYLDSYDPSTGEVYCHVPNSGKEEIEAAVEAARAAFPG

WSSRSPQERSQVLQRLADLLEQSLEELAQAESKDQGKTITLARTMDIPRAVHNFRFFASS

ILHHTSECTQMDHLGCLHYTVRAPVGIAALISPWNLPLYLLTWKIAPAIAAGNTVIAKPS

ELTSVTAWMMCRLLEKAGVPPGVVNIVFGTGPRVGEALVSHPEVPLISFTGSQPTAERIM

QLSAPHCKKLSLELGGKNPAVIFEDANLAECIPTTVRSSFANQGEICLCTSRIFVQRSIY

SEFLKRFVEAARMWKVGIPSDPSADMGALISKAHLEKVRSYIKKARMEGAQILCGEGVDK

LNLPPRNQAGYFMLPTVITDVKDESCCMKEEIFGPVTCVVPFDSEEEVIQRANNVKYGLA

ATVWSGNVGRVHRVAKKLQSGLVWTNCWLIRELNLPFGGMKSSGVGREGAKDSYEFFTEV

KTITVKH

>sp|Q56JW4|APT_BOVIN Adenine phosphoribosyltransferase OS=Bos taurus OX=9913 GN=APRT PE=2 SV=1

MADPELQLVARRIRSFPNFPIPGVLFRDISPVLKDPTSFRASINLLANHLKKAHGGRIDY

IAGLDSRGFLFGPSLAQELGLGCILIRKRGKLPGPTVCASYALEYGKGELEIQRDALEPG

QKVVVVDDLLATGGTMCAACELLGQLRAEVLECVSLVELTSLKGREKLGAVPFFSLLQYE

>sp|P52193|CALR_BOVIN Calreticulin OS=Bos taurus OX=9913 GN=CALR PE=1 SV=2

MLLPVPLLLGLLGLAAADPTVYFKEQFLDGDGWTERWIESKHKPDFGKFVLSSGKFYGDQ

EKDKGLQTSQDARFYALSARFEPFSNKGQTLVVQFTVKHEQNIDCGGGYVKLFPAGLDQT

DMHGDSEYNIMFGPDICGPGTKKVHVIFNYKGKNVLINKDIRCKDDEFTHLYTLIVRPNN

TYEVKIDNSQVESGSLEDDWDFLPPKKIKDPDAAKPEDWDDRAKIDDPTDSKPEDWDKPE

HIPDPDAKKPEDWDEEMDGEWEPPVIQNPEYKGEWKPRQIDNPEYKGIWIHPEIDNPEYS

PDSNIYAYENFAVLGLDLWQVKSGTIFDNFLITNDEAYAEEFGNETWGVTKAAEKQMKDK

QDEEQRLHEEEEEKKGKEEEEADKDDDEDKDEDEEDEDEKEEEEEEDAAAGQAKDEL

>sp|Q32PH8|EF1A2_BOVIN Elongation factor 1-alpha 2 OS=Bos taurus OX=9913 GN=EEF1A2 PE=2 SV=1

MGKEKTHINIVVIGHVDSGKSTTTGHLIYKCGGIDKRTIEKFEKEAAEMGKGSFKYAWVL

DKLKAERERGITIDISLWKFETTKYYITIIDAPGHRDFIKNMITGTSQADCAVLIVAAGV

GEFEAGISKNGQTREHALLAYTLGVKQLIVGVNKMDSTEPAYSEKRYDEIVKEVSAYIKK

IGYNPATVPFVPISGWHGDNMLEPSPNMPWFKGWKVERKEGNASGVSLLEALDTILPPTR

PTDKPLRLPLQDVYKIGGIGTVPVGRVETGILRPGMVVTFAPVNITTEVKSVEMHHEALS

EALPGDNVGFNVKNVSVKDIRRGNVCGDSKSDPPQEAAQFTSQVIILNHPGQISAGYSPV

IDCHTAHIACKFAELKEKIDRRSGKKLEDNPKSLKSGDAAIVEMVPGKPMCVESFSQYPP

LGRFAVRDMRQTVAVGVIKNVEKKSGGAGKVTKSAQKAQKAGK

>sp|A5D7I4|EXT1_BOVIN Exostosin-1 OS=Bos taurus OX=9913 GN=EXT1 PE=2 SV=1

MQAKKRYFILLSAGSCLALLFYFGGLQFRASRSHSRREEHSGRNGLHHPSPDHFWPRFPD

ALRPFVPWDQLENEDSSVHVSPRQKREANSSIYKGKKCRMESCFDFTLCKKNGFKVYVYP

QQKGEKIAESYQNILAAIEGSRFYTSDPSQACLFVLSLDTLDRDQLSPQYVHNLRSKVQS

LHLWNNGRNHLIFNLYSGTWPDYTEDVGFDIGQAMLAKASISTENFRPNFDVSIPLFSKD

HPRTGGERGFLKFNTIPPLRKYMLVFKGKRYLTGIGSDTRNALYHVHNGEDVVLLTTCKH

GKDWQKHKDSRCDRDNTEYEKYDYREMLHNATFCLVPRGRRLGSFRFLEALQAACVPVML

SNGWELPFSEVINWNQAAVIGDERLLLQIPSTIRSIHQDKILALRQQTQFLWEAYFSSVE

KIVLTTLEIIQDRIFKHISRNSLIWNKHPGGLFVLPQYSSYLGDFPYYYANLGLKPPSKF

TAVIHAVTPLVSQSQPVLKLLVAAAKSQYCAQIIVLWNCDKPLPAKHRWPATSVPVIVIE

GESKVMSSRFLPYDNIITDAVLSLDEDTVLSTTEVDFAFTVWQSFPERIVGYPARSHFWD

NSKERWGYTSKWTNDYSMVLTGAAIYHKYYHYLYTHYLPASLKNMVDQLANCEDILMNFL

VSAVTKLPPIKVTQKKQYKETMMGQTSRASRWADPDHFAQRQSCMNTFASWFGYMPLIHS

QMRLDPVLFKDQVSILRKKYRDIERL

>sp|P34933|HSP72_BOVIN Heat shock-related 70 kDa protein 2 OS=Bos taurus OX=9913 GN=HSPA2 PE=2 SV=2

MSARGPAIGIDLGTTYSCVGVFQHGKVEIIANDQGNRTTPSYVAFTDTERLIGDAAKNQV

AMNPTNTIFDAKRLIGRKFEDATVQSDMKHWPFRVVSEGGKPKVQVEYKGEIKTFFPEEI

SSMVLTKMKEIAEAYLGGKVQSAVITVPAYFNDSQRQATKDAGTITGLNVLRIINEPTAA

AIAYGLDKKGCAGGEKNVLIFDLGGGTFDVSILTIEDGIFEVKSTAGDTHLGGEDFDNSM

VSHLAEEFKRKHKKDIAPNKRAVRRLRTACERAKRTLSSSTQASIEIDSLYEGVDFYTSI

TRARFEELNADLFRGTLEPVEKALRDAKLDKGQIQEIVLVGGSTRIPKIQKLLQDFFNGK

ELNKSINPDEAVAYGAAVQAAILIGDKSENVQDLLLLDVTPLSLGIETAGGVMTPLIKRN

TTIPTKQTQTFTTYSDNQSSVLVQVYEGERAMTKDNNLLGKFDLTGIPPAPRGVPQIEVT

FDIDANGILNVTAADKSTGKENKITITNDKGRLSKDDIDRMVQEAERYKSEDEANRDRVA

AKNAVESYTYNIKQTVEDEKLRGKISDQDKNKILDKCQEVINWLDRNQMAEKDEYEHKQK

ELERVCNPIISKLYQGGPGGGGGSGASGGPTIEEVD

>sp|Q5E9B1|LDHB_BOVIN L-lactate dehydrogenase B chain OS=Bos taurus OX=9913 GN=LDHB PE=2 SV=4

MATLKEKLIAPVAEEETRIPNNKITVVGVGQVGMACAISILGKSLTDELALVDVLEDKLK

GEMMDLQHGSLFLQTPKIVADKDYSVTANSKIVVVTAGVRQQEGESRLNLVQRNVNVFKF

IIPQIVKYSPDCIIIVVSNPVDILTYVTWKLSGLPKHRVIGSGCNLDSARFRYLMAEKLG

IHPSSCHGWILGEHGDSSVAVWSGVNVAGVSLQELNPEMGTDNDSENWKEVHKMVVESAY

EVIKLKGYTNWAIGLSVADLIESMLKNLSRIHPVSTMVKGMYGIENEVFLSLPCILNARG

LTSVINQKLKDEEVAQLKKSADTLWGIQKDLKDL

>sp|Q5EAD2|SERA_BOVIN D-3-phosphoglycerate dehydrogenase OS=Bos taurus OX=9913 GN=PHGDH PE=2 SV=3

MAFANLRKVLISDSLDPCCRKILQDGGLQVVEKQNLSKEELIAELQDCEGLIVRSATKVT

SDIINAAEKLQVVGRAGTGVDNVDLEAATRKGILVMNTPNGNSLSAAELTCGMIMCLARQ

IPQAAASMKDGKWERKKFMGTELNGKVLGILGLGRIGREVATRMQSFGMKTIGYDPIISP

EVSASFGVQQLPLEQIWPLCDFITVHTPLLPSTTGLLNDSTFAQCKKGVCVVNCARGGIV

DEGALLRALQSGQCAGAALDVFTEEPPRDRALVNHENVISCPHLGASTKEAQSRCGEEIA

LQFVDMVKGKALAGVVNAQALASIFCPHTKPWVSLAKALGALMQAWAGSPKGAIQVVTQG

SSLKNSGSCLAPAVIIGLLKDASQQSNVNLVNAMLLVKEAGLDVTTSHNPATPREQDFGE

CLLTVALAGAPYQAVGLVQGTKPVLQALNGAVFRPEVPLHPGQPLLMFRAQASNPAMLPT

MIGLLAEAGVQLLSYQSSVVSDGETWHVMSISSLLPSLAPWKPHVTEAFQFCF

>sp|P00745|PROC_BOVIN Vitamin K-dependent protein C (Fragment) OS=Bos taurus OX=9913 GN=PROC PE=1 SV=1

XTSLLLFVTIWGISSTPAPPDSVFSSSQRAHQVLRIRKRANSFLEELRPGNVERECSEEV

CEFEEAREIFQNTEDTMAFWSFYSDGDQCEDRPSGSPCDLPCCGRGKCIDGLGGFRCDCA

EGWEGRFCLHEVRFSNCSAENGGCAHYCMEEEGRRHCSCAPGYRLEDDHQLCVSKVTFPC

GRLGKRMEKKRKTLKRDTNQVDQKDQLDPRIVDGQEAGWGESPWQAVLLDSKKKLVCGAV

LIHVSWVLTVAHCLDSRKKLIVRLGEYDMRRWESWEVDLDIKEVIIHPNYTKSTSDNDIA

LLRLAKPATLSQTIVPICLPDSGLSERKLTQVGQETVVTGWGYRDETKRNRTFVLSFIKV

PVVPYNACVHAMENKISENMLCAGILGDPRDACEGDSGGPMVTFFRGTWFLVGLVSWGEG

CGRLYNYGVYTKVSRYLDWIYGHIKAQEAPLESQVP

>sp|P62833|RAP1A_BOVIN Ras-related protein Rap-1A OS=Bos taurus OX=9913 GN=RAP1A PE=1 SV=1

MREYKLVVLGSGGVGKSALTVQFVQGIFVEKYDPTIEDSYRKQVEVDCQQCMLEILDTAG

TEQFTAMRDLYMKNGQGFALVYSITAQSTFNDLQDLREQILRVKDTEDVPMILVGNKCDL

EDERVVGKEQGQNLARQWCNCAFLESSAKSKINVNEIFYDLVRQINRKTPVEKKKPKKKS

CLLL

>sp|A2I7N2|SPA36_BOVIN Serpin A3-6 OS=Bos taurus OX=9913 GN=SERPINA3-6 PE=3 SV=1

MRTERVSPLLALGILVAGLCSRVHCLPENVTPEEQHKVTSVDGHSLASSNTDFAFSLYKQ

LALKDPNKNVIFSPLSVSIALAFLSLGAHGPTVTEILEGLKFNLTETPETEIHQGFQHLL

QTFNQPSNQLQLSVGNAIFVQEELKLLDKFIEDARVLYSSEAFPTNFRDPEAAKSLINDY

VKNKTQGKIEELFKDLSPRTELVLVNYVYFKAQWKTRFDPKHTEKTEFHVSDNKTVEVPM

MTLDLETPYFRDEELGCTLVELTYTSNDSALFILPDKGKMQDLEAKLTPEMLTRWRNSLQ

PRRIHELYLPKFSIKSNYELNDTLSQMGIKKIFTDADLSGITGTADLVVSQVVHGAALDV

DEEGTEGAAATGIGIERTFLRIIVRVNRPFLIAVVLKDTQSIIFLGKVTNPSEA

>sp|Q3SWW8|TSP4_BOVIN Thrombospondin-4 OS=Bos taurus OX=9913 GN=THBS4 PE=2 SV=1

MLAPRGATFLLLHLALQPWLGAGAQATPQVFDLLPSASQRLNPSVLQPILTDPTLNELYV

ISTFKLQSKSSATIFGLYSSADHSKYFEFTVMGRLNKAILRYLKNDGRIHLVVFNNLQLA

DGRRHRLLLRLTNLHRGAGSVELFLDCTRVDSIHNLPRAFSGLAQSPEAVELRTFQRKAH

DSLEELKLVVRGSLIQVASLQDCFLQQSEPLATTNTGDFNRQFLGQMSQLNQLLGEVKDL

LRQQVKETSFLRNTIAECQACGPLSFQSPTPNTLMPVVPAASPTPPVRRCDSNPCFRGVR

CTDTRDGFQCGPCPEGYTGNGIVCSDVDECRYHPCYPGVRCVNLAPGFRCDACPVGFTGP

MMQGVGISFAKTNKQVCTDIDECRNGACVLNSICINTLGSYRCGPCKPGYIGDQMRGCKM

ERNCRDPELNPCSVNAQCIEERQGDVTCVCGVGWAGDGYICGKDVDIDSYPDEELPCSAR

NCKKDNCKYVPNSGQEDADRDGIGDACDDDADGDGILNEQDNCVLTHNVDQRNSDKDIFG

DACDNCRNVLNNDQKDTDGDGKGDACDDDMDGDGIKNILDNCQKVPNSDQEDRDGDGVGD

ACDSCPEVSNPNQSDVDNDLVGDSCDTNQDSDGDGHQDSTDNCPTVINSAQLDTDKDGIG

DECDDDDDNDGIPDLVPPGPDNCRLVPNPAQEDSNSDGVGDICEADFDQDQVIDRIDVCP

ENAEVTLTDFRAYQTVVLDPEGDAQIDPNWVVLNQGMEIVQTMNSDPGLAVGYTAFNGVD

FEGTFHVNTQTDDDYAGFIFGYQDSSSFYVVMWKQTEQTYWQATPFRAVAEPGIQLKAVK

SKTGPGEHLRNSLWHTGDTSDQVRLLWKDSRNVGWKDKVSYRWFLQHRPQVGYIRVRFYE

GSELVADSGVTIDTTMRGGRLGVFCFSQENIIWSNLKYRCNDTIPEDFQEFQTQNFDRLD

K

>sp|Q58DK4|TKFC_BOVIN Triokinase/FMN cyclase OS=Bos taurus OX=9913 GN=TKFC PE=2 SV=1

MTSKKLVNSVAGCADDALAGLVACNPSLQLLQGHRVALRSDLDSLKGRVALLSGGGSGHE

PAHAGFIGKGMLTGVIAGAVFTSPAVGSILAAIRAVAQAGTVGTLLIVKNYTGDRLNFGL

AREQARAEGIPVEMVVVGDDSAFTVLKKAGRRGLCGTVLIHKVAGALAEAGVGLEEITDR

VSVVAKAMGTLGVSLSSCSVPGSKPTFELSADEVELGLGIHGEAGVRRIKMATANEIVAL

MLDHMTSSSNASHVPVPPGSSVVLMVNNLGGLSFLELGIIADAAVCSLEGHGVKIARALV

GTFMSALEMPGVSLTLLLVDEPLLKLIDAETTASAWPNVAKVWVTGRKRSRAAPTEPLAA

PDSTTAAGEASKQMVLVLEWVCTTLLGLEEHLNALDRAAGDGDCGTTHSRAARAIXGWLK

EGPPPASPAQLLSKLSFLLLEKMGGSSGALYGLFLTAAAQPLKAKTDLPAWSAAMDAGLE

AMQKYGKAAPGDRTMLDSLWAAGQELQAWKSPGANMLQILTKAVKSAEAAAEATKNMEAG

AGRASYISSARLDQPDPGAVAAAAILRAILEVLQSQGA

>sp|Q5E956|TPIS_BOVIN Triosephosphate isomerase OS=Bos taurus OX=9913 GN=TPI1 PE=2 SV=3

MAPSRKFFVGGNWKMNGRKNNLGELINTLNAAKVPADTEVVCAPPTAYIDFARQKLDPKI

AVAAQNCYKVANGAFTGEISPGMIKDLGATWVVLGHSERRHVFGESDELIGQKVAHALAE

GLGVIACIGEKLDEREAGITEKVVFEQTKVIADNVKDWSKVVLAYEPVWAIGTGKTATPQ

QAQEVHEKLRGWLKSNVSDAVAQSARIIYGGSVTGATCKELASQPDVDGFLVGGASLKPE

FVDIINAKQ

>sp|P41541|USO1_BOVIN General vesicular transport factor p115 OS=Bos taurus OX=9913 GN=USO1 PE=1 SV=1

MNFLRGVMGGQSAGPQHTEAETIQKLCDRVASSTLLDDRRNAVRALKSLSKKYRLEVGIQ

AMEHLIHVLQTDRSDSEIIGYALDTLYNIISNDEEEEVEENSTRQSEDLGSQFTEIFIKQ

QENVTLLLSLLEEFDFHVRWPGVKLLTSLLKQLGPQVQQIILVSPMGVSRLMDLLADSRE

VIRNDGVLLLQALTRSNGAIQKIVAFENAFERLLDIITEEGNSDGGIVVEDCLILLQNLL

KNNNSNQNFFKEGSYIQRMKPWFEVGDENSGWSAQKVTNLHLMLQLVRVLVSPNNPPGAT

SSCQKAMFQCGLLQQLCTILMATGVPADILTETINTVSEVIRGCQVNQDYFASVNAPSNP

PRPAIVVLLMSMVNERQPFVLRCAVLYCFQCFLYKNQKGQGEIVSTLLPSTIDATGNTVS

AGQLLCGGLFSTDSLSNWCAAVALAHALQENATQKEQLLRVQLATSIGNPPVSLLQQCTN

ILSQGSKIQTRVGLLMLLCTWLSNCPIAVTHFLHNSANVPFLTGQIAENLGEEEQLVQGL

CALLLGISIYFNDNSLETYMKEKLKQLIEKRIGKENFIEKLGFISKHELYSRASQKPQPN

FPSPEYMIFDHEFTKLVKELEGVITKAIYKSSEEDKKEEEVKKTLEQHDSIVTHYKNMIR

EQDLQLEELKQQISTLKCQNEQLQTAVTQQVSQIQQHKDQYNLLKVQLGKDSQHQGPYTD

GAQMNGVQPEEISRLREEIEELKSNRELLQSQLAEKDSLIENLKSSQLSPGTNEQSSATA

GDSEQIAELKQELATLKSQLNSQSVEITKLQTEKQELLQKTEAFAKSAPVPGESETVIAT

KTTDVEGRLSALLQETKELKNEIKALSEERTAIKEQLDSSNSTIAILQNEKNKLEVDITD

SKKEQDDLLVLLADQDQKIFSLKNKLKELGHPVEEEDELESGDQDDEDDEDEDDGKEQGH

I

>sp|G3MYZ3|AFAM_BOVIN Afamin OS=Bos taurus OX=9913 GN=AFM PE=1 SV=1

MKQLKLTGFVIFFFFLTESLTLPTQPQDVDDVRITQKFIDDNIGYITIIAFAQYIQEASF

EEVEMLVKAMTEYRDKCLADRTLPECSKLANEVLLENICAMEGLPQKYNFSHCCHKVDFE

RRLCFFHNKKADIGLLPPLPTLDPEEKCQTYKNNRESFLNNYVYEVSRRNPFVFAPTLLT

VAARFEEMTKTCCEEQEKANCFQTKAEPFIYYLKALSSYQKNACRALMKFGRQILQSINI

AILSQKFPKIGFKQLTSLLEDVSSKYDGCCEGDVVQCIRGRSKVMSHICSKQDSISSKIK

DCCEKKIPERGECIIYSNKDDRPNDLSLREAKFIESDNVCEKRDADQANFMAEFLYEYSR

RHPELSTPELLRIAKVYKDLLKECCNMENPPECYRHAENRFNETTEKSLKIVQRECEHFQ

NLGKDDLKYHYLINLTKLAPQLSTEELTFLGKEMVMALTTCCTLSEEFACVDNLVDLVLG

ELCGINENRNINPAVDHCCKTNFAFRRSCFESLEADKTYVPPSTSQGLFTFHADLCQAHN

EELQRKKDRFLVNLVKLKPELAGEELWSLLADFTNVVEKCCKAQEPEACFKEESPKLAAK

SQAA

>sp|P84081|ARF2_BOVIN ADP-ribosylation factor 2 OS=Bos taurus OX=9913 GN=ARF2 PE=2 SV=1

MGNVFEKLFKSLFGKKEMRILMVGLDAAGKTTILYKLKLGEIVTTIPTIGFNVETVEYKN

ISFTVWDVGGQDKIRPLWRHYFQNTQGLIFVVDSNDRERVNEAREELTRMLAEDELRDAV

LLVFVNKQDLPNAMNAAEITDKLGLHSLRQRNWYIQATCATSGDGLYEGLDWLSNQLKNQ

K

>sp|Q0VCX1|C1S_BOVIN Complement C1s subcomponent OS=Bos taurus OX=9913 GN=C1S PE=2 SV=2

MWCIVLFSLVAWVYAEPTMYGEILSPNYPQVYPNEVEKSWDIEVPAGYGIHLYFTHLDIE

LSENCSYDSVQIMSGGHEEGKLCGRRTNKNSNSPVVKEFHIPYSKLQVIFRSDFSNEERF

TGFAAYYVAEDIDECTAFADAPCSHFCNNFLGGYFCSCPPEYFLHEDKKNCGVNCSGNVF

TTMTGEVESPNYPSPYPESSRCDYQIQLEEGFRVVVTMRREDFDVEPADSEGHCPDSLLF

VAGDQHFGPYCGNGFPGPLTIETQSSALNIIFQTDGSEQRKGWKFRYHGDPIPCPKEVTA

NSFWEPERAKYVFRDVVKITCVDGFEVVQGSVGSPSFYSTCQSNGKWSNSKLRCQPVDCG

APEPIQHGRVEDPESTLFGSITRYSCEMPYYSMECEGSEVYHCSGNGSWVNKVLGIEPPK

CIAVCGTPSEPFRSTQRIFGGSIAKIENFPWQVFFSNPWAGGALIDEYWVLTAAHVVEGN

DIPVMYVGSSSVVTSQLSNAQMLTAERVFIHPGWEVLDPSITRKNFDNDIALVRLRDPVK

MGPKVAPICLPGTSSEYDPPENVLGLISGWGRTNVKSHVIKLRGAKLPVAPLSKCREMKG

VNPGIDISSFVFTENMICAGNDKGVDSCDGDSGGAFAVQDPKENKPKFYVAGLVSWGPQC

GTYGIYTRVKNYVDWIRKTMQEYSAPSVD

>sp|P22226|CTHL1_BOVIN Cathelicidin-1 OS=Bos taurus OX=9913 GN=CATHL1 PE=1 SV=2

METPRASLSLGRWSLWLLLLGLALPSASAQALSYREAVLRAVDQLNEQSSEPNIYRLLEL

DQPPQDDEDPDSPKRVSFRVKETVCSRTTQQPPEQCDFKENGLLKRCEGTVTLDQVRGNF

DITCNNHQSIRITKQPWAPPQAARLCRIVVIRVCR

>sp|Q3SZ54|IF4A1_BOVIN Eukaryotic initiation factor 4A-I OS=Bos taurus OX=9913 GN=EIF4A1 PE=2 SV=1

MSASQDSRSRDNGPDGMEPEGVIESNWNEIVDSFDDMNLSESLLRGIYAYGFEKPSAIQQ

RAILPCIKGYDVIAQAQSGTGKTATFAISILQQIELDLKATQALVLAPTRELAQQIQKVV

MALGDYMGASCHACIGGTNVRAEVQKLQMEAPHIIVGTPGRVFDMLNRRYLSPKYIKMFV

LDEADEMLSRGFKDQIYDIFQKLNSNTQVVLLSATMPSDVLEVTKKFMRDPIRILVKKEE

LTLEGIRQFYINVEREEWKLDTLCDLYETLTITQAVIFINTRRKVDWLTEKMHARDFTVS

AMHGDMDQKERDVIMREFRSGSSRVLITTDLLARGIDVQQVSLVINYDLPTNRENYIHRI

GRGGRFGRKGVAINMVTEEDKRTLRDIETFYNTSIEEMPLNVADLI

>sp|Q3ZC09|ENOB_BOVIN Beta-enolase OS=Bos taurus OX=9913 GN=ENO3 PE=2 SV=1

MAMQKIFAREILDSRGNPTVEVDLHTAKGRFRAAVPSGASTGIYEALELRDGDKSRYLGK

GVLKAVEHINKTLGPALLEKKLSVVDQEKVDKFMIELDGTENKSKFGANAILGVSLAVCK

AGAAEKGVPLYRHIADLAGNPELILPVPAFNVINGGSHAGNKLAMQEFMILPVGASSFRE

AMRIGAEVYHHLKGVIKAKYGKDATNVGDEGGFAPNILENNEALELLKTAIQAAGYPDKV

VIGMDVAASEFYRNGKYDLDFKSPDDPARHISGEKLGELYKNFIKNYPVVSIEDPFDQDD

WATWTSFLSGVNIQIVGDDLTVTNPKRIAQAVEKKACNCLLLKVNQIGSVTESIQACKLA

QSNGWGVMVSHRSGETEDTFIADLVVGLCTGQIKTGAPCRSERLAKYNQLMRIEEALGDK

AVFAGRKFRNPKAK

>sp|P00743|FA10_BOVIN Coagulation factor X OS=Bos taurus OX=9913 GN=F10 PE=1 SV=1

MAGLLHLVLLSTALGGLLRPAGSVFLPRDQAHRVLQRARRANSFLEEVKQGNLERECLEE

ACSLEEAREVFEDAEQTDEFWSKYKDGDQCEGHPCLNQGHCKDGIGDYTCTCAEGFEGKN

CEFSTREICSLDNGGCDQFCREERSEVRCSCAHGYVLGDDSKSCVSTERFPCGKFTQGRS

RRWAIHTSEDALDASELEHYDPADLSPTESSLDLLGLNRTEPSAGEDGSQVVRIVGGRDC

AEGECPWQALLVNEENEGFCGGTILNEFYVLTAAHCLHQAKRFTVRVGDRNTEQEEGNEM

AHEVEMTVKHSRFVKETYDFDIAVLRLKTPIRFRRNVAPACLPEKDWAEATLMTQKTGIV

SGFGRTHEKGRLSSTLKMLEVPYVDRSTCKLSSSFTITPNMFCAGYDTQPEDACQGDSGG

PHVTRFKDTYFVTGIVSWGEGCARKGKFGVYTKVSNFLKWIDKIMKARAGAAGSRGHSEA

PATWTVPPPLPL

>sp|Q3SZB7|F16P1_BOVIN Fructose-1,6-bisphosphatase 1 OS=Bos taurus OX=9913 GN=FBP1 PE=2 SV=3

MTDQAAFDTNIVTVTRFVMEEGRKARGTGEMTQLLNSLCTAVKAISTAVRKAGIAHLYGI

AGTTNVTGDQVKKLDVLSNDLVVNVLKSSFATCVLVSEEDEHAIIVEPEKRGKYVVCFDP

LDGSSNIDCLVSIGTIFGIYKKISKDDPSEKDALQPGRNLVAAGYALYGSATMLVLAMAN

GVNCFMLDPAIGEFILVDRDVKIKKKGSIYSLNEGYAKDFDPALTEYVQRKKFPPDNSAP

YGARYVGSMVADVHRTLVYGGIFMYPANKKSPSGKLRLLYECNPMAYVIEKAGGMATTGK

ETVLDIVPTDIHQKSPIILGSPEDVTEFLEIYKKHAAK

>sp|Q59A32|PUR2_BOVIN Trifunctional purine biosynthetic protein adenosine-3 OS=Bos taurus OX=9913 GN=GART PE=2 SV=1

MAARVLVIGNGGREHTLAWKLAQSTHVKQVLVTPGNAGTACSEKISNTDISISDHTALAQ

FCKDEKIEFVVVGPEAPLAAGIVGNLNSVGVRCFGPTAQAAQLESSKRFAKEFMDRHGIS

TARWRAFTKPKEACDFIMSADFPALVVKASGLAAGKGVIVAKSKEEACEAVREIMQGKAF

GEAGETVVIEELLEGEEVSCLCFTDGRTVAPMPPAQDHKRLLEGDEGPNTGGMGAYCPAP

QVSKDLLLKIKNNILQRTVDGMQEEGMPYTGVLYAGIMLTKNGPKVLEFNCRFGDPECQV

ILPLLKSDLYEVIQSILDGLLCTSLPVWLDNCAAVTVVMASKGYPGDYTKGVEITGFPEA

QALGLEVFQAGTALKDGKVVTNGGRVLTVTAIRENLISALEEARKGLAAIKFEGAVYRKD

IGFRAIAFLQQPRGLTYKESGVDIAAGNMLVQKIKPLAKATSRPGCDVDLGGFAGLFDLK

AAGFTDPLLACGTDGVGTKLKIAQQCSKHDTIGQDLVAMCVNDILAQGAEPLFFLDYFSC

GKLDLRTTEAVITGIAKACKKAGCALLGGETAEMPDMYPPGEYDLAGFAVGAMERDQKLP

QLERITEGDAVIGIASSGLHSNGFSLVRKIVAKSSLEYSSPAPGGCGDQTLGDLLLTPTK

IYSRSLLPVLRSGRVKAVAHITGGGLLENIPRVLPQKLGVNLDAQTWRVPRIFSWLQQEG

HLSEEEMARTFNCGIGAALVVSEDLVKQTLQDIEQHQEEACVIGRVVACPKGSPRVKVEH

LIETMQINGSVLENGTLRNHFSVQPKKARVAVLISGTGSNLQALIDSTREPSSLAHIVIV

ISNKAAVAGLDKAEKAGIPTRVINHKLYKNRAAFDTAIDEVLEEFSTDIVCLAGFMRILS

GPFVRKWNGKMLNIHPSLLPSFKGSNAHEQVLDAGVTVTGCTVHFVAEDVDAGQIILQEA

VPVKRGDTVETLSERVKLAEHKIFPSALQLVASGAVRLGENGRICWVTED

>sp|Q9N0V4|GSTM1_BOVIN Glutathione S-transferase Mu 1 OS=Bos taurus OX=9913 GN=GSTM1 PE=1 SV=3

MPMILGYWDIRGLAHAIRLLLEYTDTNYEERQYSVGDAPDYDRSQWLNEKFKLGLDFPNL

PYLIDGTHKLTQSNAILRYIARKHNLCGETEEEMIRVDILENQVMDVRLAMARICYSPDF

EKLKPGFLKEIPEKIKLFSEFLGKRPWFAGDKLTYVDFLVYDVLDMHRIFEPKCLDAFPN

LKDFISRFEGLKKISAYMKSSRFLPGPLFMKLAVWGNK

>sp|Q3T149|HSPB1_BOVIN Heat shock protein beta-1 OS=Bos taurus OX=9913 GN=HSPB1 PE=2 SV=1

MAERRVPFSLLRGPSWDPFRDWYPAHSRLFDQAFGLPRLPEEWSQWLSHSGWPGYVRALP

AAAIEGPAYNRALSRQLSSGVSEIQQTADRWRVSLDVNHFAPEELTVKTKDGVVEITGKH

EERQDEHGYISRCFTRKYTLPPGVDPTLVSSSLSPEGTLTVEAPLPKSATQSAEITIPVT

FQARAQLGGPEAGKSEQPENK

>sp|Q2KJC6|METK1_BOVIN S-adenosylmethionine synthase isoform type-1 OS=Bos taurus OX=9913 GN=MAT1A PE=2 SV=1

MNGPVDGLCDHSLSEEGAFMFTSESVGEGHPDKICDQISDAVLDAHLKQDPNAKVACETV

CKTGMVLLCGEITSMAMVDYQRVVRETIQHIGYDDSAKGFDFKTCNVLVALEQQSPDIAQ

CVHLDRNEEDVGAGDQGLMFGYATDETEECMPLTIMLAHRLNARMAELRRSGQLPWLQPD

SKTQVTVQYTQDNGAVIPMRVHTVVISVQHNEDITLEDMRRALKEQVIRAVVPARYLDED

TIYHLQPSGRFVIGGPQGDAGVTGRKIIVDTYGGWGAHGGGAFSGKDYTKVDRSAAYAAR

WVAKSLVKAGLCRRVLVQVSYAIGVAEPLSISIFTYGTSQKTERELLDVVNKNFDLRPGV

IVRDLDLKKPIYQKTACYGHFGRSEFPWEVPKKLVF

>sp|O02659|MBL2_BOVIN Mannose-binding protein C OS=Bos taurus OX=9913 GN=MBL PE=2 SV=1

MSLFTSLPFLLLTAVTASCADTETENCENIRKTCPVIACGPPGINGIPGKDGRDGAKGEK

GEPGQGLRGSQGPPGKMGPQGTPGIPGIPGPIGQKGDPGENMGDYIRLATSERATLQSEL

NQIKNWLIFSLGKRVGKKAFFTNGKKMPFNEVKTLCAQFQGRVATPMNAEENRALKDLVT

EEAFLGITDQETEGKFVDLTGKGVTYQNWNDGEPNNASPGEHCVTLLSDGTWNDIACSAS

FLTVCEFSL

>sp|Q1RMU3|P4HA1_BOVIN Prolyl 4-hydroxylase subunit alpha-1 OS=Bos taurus OX=9913 GN=P4HA1 PE=1 SV=1

MIWYILVVGILLPQSLAHPGFFTSIGQMTDLIHTEKDLVTSLKDYIKAEEDKLEQIKKWA

EKLDRLTSTATKDPEGFVGHPVNAFKLMKRLNTEWSELENLVLKDMSDGFISNLTIQRQY

FPNDEDQVGAAKALLRLQDTYNLDTDTISKGDLPGVKHKSFLTVEDCFELGKVAYTEADY

YHTELWMEQALRQLDEGEVSTVDKVSVLDYLSYAVYQQGDLDKALLLTKKLLELDPEHQR

ANGNLKYFEYIMAKEKDANKSSSDDQSDQKTTLKKKGAAVDYLPERQKYEMLCRGEGIKM

TPRRQKKLFCRYHDGNRNPKFILAPAKQEDEWDKPRIIRFHDIISDAEIEVVKDLAKPRL

RRATISNPITGDLETVHYRISKSAWLSGYENPVVSRINMRIQDLTGLDVSTAEELQVANY

GVGGQYEPHFDFARKDEPDAFKELGTGNRIATWLFYMSDVLAGGATVFPEVGASVWPKKG

TAVFWYNLFASGEGDYSTRHAACPVLVGNKWVSNKWLHERGQEFRRPCTLSELE

>sp|O77834|PRDX6_BOVIN Peroxiredoxin-6 OS=Bos taurus OX=9913 GN=PRDX6 PE=1 SV=3

MPGGLLLGDEAPNFEANTTIGRIRFHDYLGDSWGILFSHPRDFTPVCTTELGRAAKLAPE

FAKRNVKMIALSIDSVEDHLAWSKDINAYNGEEPTEKLPFPIIDDKNRDLAIQLGMLDPA

EKDEKGMPVTARVVFIFGPDKKLKLSILYPATTGRNFDEILRVIISLQLTAEKRVATPVD

WKNGDSVMVLPTIPEEEAKKLFPKGVFTKELPSGKKYLRYTPQP

>sp|P00515|KAP2_BOVIN cAMP-dependent protein kinase type II-alpha regulatory subunit OS=Bos taurus OX=9913 GN=PRKAR2A PE=1 SV=2

MSHIQIPPGLTELLQGYTVEVLRQRPPDLVDFAVDYFTRLREARSRASTPPAAPPSGSQD

FDPGAGLVADAVADSESEDEEDLDVPIPGRFDRRVSVCAETYNPDEEEEDTDPRVIHPKT

DQQRCRLQEACKDILLFKNLDPEQLSQVLDAMFERTVKVDEHVIDQGDDGDNFYVIERGT

YDILVTKDNQTRSVGQYDNHGSFGELALMYNTPRAATIVATSEGSLWGLDRVTFRRIIVK

NNAKKRKMFESFIESVPLLKSLEVSERMKIVDVIGEKVYKDGERIITQGEKADSFYIIES

GEVSILIKSKTKVNKDGENQEVEIARCHKGQYFGELALVTNKPRAASAYAVGDVKCLVMD

VQAFERLLGPCMDIMKRNISHYEEQLVKMFGSSMDLIDPGQ

>sp|P62998|RAC1_BOVIN Ras-related C3 botulinum toxin substrate 1 OS=Bos taurus OX=9913 GN=RAC1 PE=1 SV=1

MQAIKCVVVGDGAVGKTCLLISYTTNAFPGEYIPTVFDNYSANVMVDGKPVNLGLWDTAG

QEDYDRLRPLSYPQTDVFLICFSLVSPASFENVRAKWYPEVRHHCPNTPIILVGTKLDLR

DDKDTIEKLKEKKLTPITYPQGLAMAKEIGAVKYLECSALTQRGLKTVFDEAIRAVLCPP

PVKKRKRKCLLL

>sp|Q9GMB8|SYSC_BOVIN Serine--tRNA ligase, cytoplasmic OS=Bos taurus OX=9913 GN=SARS1 PE=2 SV=3

MVLDLDLFRVDKGGDPALIRESQEKRFKDPGLVDQLVKADSEWRRCRFRADNLNKLKNLC

SKTIGEKMKKKEPVGNDESIPEDVLNLDDLTADTLTNLKVSQIKKVRLLVDEAILKCDAE

RIKLEAERFESLREIGNLLHPSVPISDDEDADNKVERIWGDCTVRKKYSHVDLVVMVDGF

EGEKGAVVAGSRGYFLKGVLVFLEQALIQFALRTLASRGYTPIYTPFFMRKEVMQEVAQL

SQFDEELYKVIGKGSEKSDDNSYEEKYLIATSEQPIAALHRDEWLRPEDLPIKYAGLSTC

FRQEVGSHGRDTRGIFRVHQFEKIEQFVYSSPHDNKSWEMFEEMIATAEEFYQSLGIPYH

IVNIVSGSLNHAASKKLDLEAWFPGSGAFRELVSCSNCTDYQARRLRIRYGQTKKMMDKV

EFVHMLNATMCATTRTICAILENYQTEKGILVPEKLKEFMPPGLQELIPFVKAAPIDQEP

SKKQKKQHEGSKKKGAARDVALESQLQNMEVTDA

>sp|Q9TT36|THBG_BOVIN Thyroxine-binding globulin OS=Bos taurus OX=9913 GN=SERPINA7 PE=2 SV=1

MPLFSLVLLILGLHCAPPNSCEGKITSCLSPQQNATLYKMSSINADFAFNLYRRFTVEIP

DQNIFFSPVSIPAGLAMLSLGACSSTQTQILEGLGFNLTDTPVAEIQQGFQHLICSLNFP

KKELELQMGNALFIGKQLKPLEKFLDDVKNLYETEVFSTDFSNVSAAQQEINSHVEKQTK

GKIVGLIQDLKPNTITVLVNYLCFKAQWANPFDPSKTEEGSSFLVDKTTTVQVPMMHQME

QYYHLVDTELNCTVLQMDYSKNALALFVLPKEGQMEWVEGAMSSKTLKKWNRLLRKGWVD

LFVPKFSISATYDLGDILLKMGIQDAFADNADFSGLTKDNGLKVSNVAHKAMFYIGEKGT

EAVPEVRFLNQPETTLLHPIIQFDRSFLLLILEKNTRSILFLGKVVDPTEA

>sp|Q2KJH6|SERPH_BOVIN Serpin H1 OS=Bos taurus OX=9913 GN=SERPINH1 PE=2 SV=1

MRALLLISTICLLARALAAEVKKPAAAAAPGTAEKLSPKAATLAERSAGLAFSLYQAMAK

DQAVENILLSPVVVASSLGLVSLGGKAATASQAKAVLSAEQLRDDEVHAGLGELLRSLSN

STARNVTWKLGSRLYGPSSVSFAEDFVRSSKQHYNCEHSKINFRDKRSALQSINEWAAQT

TDGKLPEVTKDVERTDGALLVNAMFFKPHWDERFHHKMVDNRGFMVTRSYTVGVTMMHRT

GLYNYYDDEKEKLQMVEMPLAHKLSSLIIIMPHHVEPLERLEKLLTKEQLKVWMGKMQKK

AVAISLPKGVVEVTHDLQKHLAGLGLTEAIDKNKADLSRMSGKKDLYLASVFHATAFEWD

TDGNPFDQDIYGREELRSPKLFYADHPFIFLVRDTQSGSLLFIGRLVRPKGDKMRDEL

>sp|P50227|ST1A1_BOVIN Sulfotransferase 1A1 OS=Bos taurus OX=9913 GN=SULT1A1 PE=1 SV=2

MELIQDTSRPPAKYVKGIPLIKYFAEALGPLESFEAWPDDLLISTYPKSGTTWVSEILDL

IYQEGDLEKCQRAPVFLRVPFLEFSAPGVPTGVELLKDTPAPRLLKTHLPLALLPKTLLD

QKVKVIYIARNAKDVAVSYYHFYRMAKVHPDPGTWDSFLEKFMAGEVCYGSWYQHVQEWW

ELSHTHPVLYLFYEDIKEDPKREIQKILEFIGRSLPEETVDHIVQRTSFKEMKKNPMTNY

STIPTAVMDHSISAFMRKGITGDWKSTFTVAQNELFEAHYAKKMAGCKLRFRWEL

>sp|Q5E9F5|TAGL2_BOVIN Transgelin-2 OS=Bos taurus OX=9913 GN=TAGLN2 PE=2 SV=3

MANRGPAYGLSREVQQKIEKQYDADLEQILIQWITTQCRKDVGRPQPGRENFQNWLKDGT

VLCELINGLYPEGQAPVKKIQASTMAFKQMEQISQFLQAAERYGINTTDIFQTVDLWEGK

NMACVQRTLMNLGGLAVARDDGLFSGDPNWFPKKSKENPRYFSDNQLQEGKNVIGLQMGT

NRGASQAGMTGYGMPRQIL

>sp|P63103|1433Z_BOVIN 14-3-3 protein zeta/delta OS=Bos taurus OX=9913 GN=YWHAZ PE=1 SV=1

MDKNELVQKAKLAEQAERYDDMAACMKSVTEQGAELSNEERNLLSVAYKNVVGARRSSWR

VVSSIEQKTEGAEKKQQMAREYREKIETELRDICNDVLSLLEKFLIPNASQAESKVFYLK

MKGDYYRYLAEVAAGDDKKGIVDQSQQAYQEAFEISKKEMQPTHPIRLGLALNFSVFYYE

ILNSPEKACSLAKTAFDEAIAELDTLSEESYKDSTLIMQLLRDNLTLWTSDTQGDEAEAG

EGGEN

>sp|Q32KY0|APOD_BOVIN Apolipoprotein D OS=Bos taurus OX=9913 GN=APOD PE=2 SV=1

MVPVLLLLPALAGLFGAAEGQAFHLGKCPHPPVQENFDVNKYLGKWYEIEKIPVSFEKGS

CIQANYSLKENGNVEVINKELRADGTVNQIEGEATPENITEPAKLAVKFFWFMPSAPYWV

LATDYENYALVYSCTTIIWLFHMDHVWILGRNPYLPPETVTYLKDILTSNNIEVEKMTIT

DQVNCPESM

>sp|Q17QH6|COL11_BOVIN Collectin-11 OS=Bos taurus OX=9913 GN=COLEC11 PE=2 SV=1

MKRALALMGLAFLCVLRAGAAQQTVDDACSVQILVPGLKGDAGEKGDKGAPGRPGRVGPT

GEKGDVGDKGQKGGVGRHGKIGPIGSKGEKGDSGDIGPPGPNGEPGIPCECSQLRKAIGE

MDNQVTQLTAELKFIKNAVAGVRETEQKMYLLVKEEKRYLDAQLACQGRGGTLSMPKDEA

ANALLAAYITQAGLARVFIGINDLEREGAFVYADRSPMQTFSKWRSGEPNNAYDEEDCVE

LVASGGWNDVACHLTMHFLCEFDKEHV

>sp|P25975|CATL1_BOVIN Procathepsin L OS=Bos taurus OX=9913 GN=CTSL PE=1 SV=3

MNPSFFLTVLCLGVASAAPKLDPNLDAHWHQWKATHRRLYGMNEEEWRRAVWEKNKKIID

LHNQEYSEGKHGFRMAMNAFGDMTNEEFRQVMNGFQNQKHKKGKLFHEPLLVDVPKSVDW

TKKGYVTPVKNQGQCGSCWAFSATGALEGQMFRKTGKLVSLSEQNLVDCSRAQGNQGCNG

GLMDNAFQYIKDNGGLDSEESYPYLATDTNSCNYKPECSAANDTGFVDIPQREKALMKAV

ATVGPISVAIDAGHTSFQFYKSGIYYDPDCSSKDLDHGVLVVGYGFEGTDSNNNKFWIVK

NSWGPEWGWNGYVKMAKDQNNHCGIATAASYPTV

>sp|Q1JP75|DCXR_BOVIN L-xylulose reductase OS=Bos taurus OX=9913 GN=DCXR PE=2 SV=1

MDLRLAGRRALVTGAGKGIGRSIVKALHAAGARVVAVSRTQADLDSLVRECPGVETVCVD

LADWEATEQALGGVGPVDLLVNNAAVAFLQPFLEVTKEAYDMSFSVNLRAVIQVSQIVAR

GLIARGAPGVIVNVSSQASQRGLTNHSVYCSTKGALDTLTKVMAVELGPHKIRVNAVNPT

VVMTPMGQAAWSDPQKAKAMLDRIPLGRFAEVENVVDTILFLLSDRSSMTTGSTVPVDGG

FLAT

>sp|Q3SZV3|EF1G_BOVIN Elongation factor 1-gamma OS=Bos taurus OX=9913 GN=EEF1G PE=2 SV=1

MAAGTLYTYPENWRAFKALIAAQYSGAQVRVLSAPPHFHFGQTNRTPEFLRKFPAGKVPA

FEGDDGFCVFESNAIAYYVSNEELRGSTPEAAAQVVQWVSFADSDIVPPASTWVFPTLGI

MHHNKQATENAKEEVRRILGLLDAHLKTRTFLVGERVTLADITVVCTLLWLYKQVLEPSF

RQAFPNTNRWFLTCINQPQFRAVLGEVKLCEKMAQFDAKKFAESQPKKDTPRKEKGSREE

KLKPQAERKEGKEEKKAAAPAPEEELDECEQALAAEPKAKDPFAHLPKSTFVLDEFKRKY

SNEDTLSVALPYFWDHFDKDGWSLWYSEYRFPEELTQTFMSCNLITGMFQRLDKLRKNAF

ASVILFGTNNSSSISGVWDFRGQELAFPLSPDWQVDYESYTWRKLDPGSEETQTLVREYF

CWEGAFQHVGKAFNQGKIFK

>sp|Q08E20|ESTD_BOVIN S-formylglutathione hydrolase OS=Bos taurus OX=9913 GN=ESD PE=2 SV=1

MALKQVSSSKCFGGLQKVFEHDSVELKCKMKFAVYLPPKAETGKCPVLYWLSGLTCTEQN

FISKSGYHQAASEHGLVVIAPDTSPRGCNIKGEEDSWDFGTGAGFYVDATEDLWKTNYRM

YSYVTKELPQLVNDNFPVDPQRMSVFGHSMGGHGALICALKNPGKYKSVSAFAPLCNPVL

CRWGKKAFTGYLGTDQSKWEAYDATYLVKSYPGPQLDILIDQGKEDEFLSDGQLLPDNFI

AACTEKKIPVVFRLQEGYDHSYYFIATFIADHIRHHAKYLNA

>sp|Q5NTB3|FA11_BOVIN Coagulation factor XI OS=Bos taurus OX=9913 GN=F11 PE=2 SV=1

MTLLYQMVHFALFASVAGECVTTLFQDACFKGGDITVAFAPNAKHCQIICTHHPRCLLFT

FMTESSSEDPTKWYTCILKDSVTETLPMVNMTGAISGYSSKQCLHHISACSKDMYVDLNM

KGMNYNSSLAQSARECQQRCTDDTHCHFFTFATRHFPSIKDRNTCLLKNTQTGTPTSITK

LHEVVSGFSLKSCGLSNLACIRDIFPRTAFVDITIDTVMAPDPFVCRSICTHHPSCLFFT

FLSEEWPTASERNLCLLKTSSSGLPSARFRKNRAFSGFSLQHCQHSVPVFCHSSFYRNTD

FLGEELDIVDADSHEACQKTCTNSIRCQFFTYSPSQESCNGGKGKCYLKLSANGSPTKIL

HGTGSISGYTLRLCKMDNVCTTKIKTRIVGGTQSVHGEWPWQITLHVTSPTQRHLCGGAI

IGNQWILTAAHCFNEVKSPNVLRVYSGILNQSEIKEDTSFFGVQEIIIHDQYEKAESGYD

IALLKLETAMNYTDSQWPICLPSKGDRNVMYTECWVTGWGYRKLRDKIQNTLQKAKVPLM

TNEECQAGYREHRITSKMVCAGYREGGKDACKGDSGGPLSCKHNEVWHLVGITSWGEGCG

QRERPGVYSNVVEYVDWILEKTQGP

>sp|P13605|FMOD_BOVIN Fibromodulin OS=Bos taurus OX=9913 GN=FMOD PE=1 SV=2

MQWASILLLAGLCSLSWAQYEEDSHWWFQFLRNQQSTYDDPYDPYPYEPYEPYPYGGEEG

PAYAYGSPPQPEPRDCPQECDCPPNFPTAMYCDNRNLKYLPFVPSRMKYVYFQNNQISSI

QEGVFDNATGLLWIALHGNQITSDKVGKKVFSKLRHLERLYLDHNNLTRIPSPLPRSLRE

LHLDHNQISRVPNNALEGLENLTALYLHHNEIQEVGSSMKGLRSLILLDLSYNHLRKVPD

GLPSALEQLYLEHNNVFSVPDSYFRGSPKLLYVRLSHNSLTNNGLASNTFNSSSLLELDL

SYNQLQKIPPVSTNLENLYLQGNRINEFSISSFCTVVDVMNFSKLQVLRLDGNEIKRSAM

PADAPLCLRLASLIEI

>sp|Q2KJE5|G3PT_BOVIN Glyceraldehyde-3-phosphate dehydrogenase, testis-specific OS=Bos taurus OX=9913 GN=GAPDHS PE=2 SV=1

MSKRDIVLTNVTVVQLLRQPCPEPRVEAEPEPPAQPQPQPEPIKEEVPPPPPPPPAPKKV

RELIVGINGFGRIGRLVLRACMEKGVKVVAVNDPFIDLEYMVYMFKYDSTHGRYKGNVEH

KKGQLVVDNNEISVFQCKQPKEIPWKSVGSPFVVEATGVYLSLEETKAHIEAGAQRVVIC

APSPDAPMFVMGVNEKEYNPSSMKIVSNASCTTNCLAPLAKVIHERFGILEGLMTTVHSY

TATQKTVDGPSKKAWRDGRGAHQNIIPASTGAAKAVGKVIPDLKGKLTGMAFRVPTPDVS

VVDLTCRLAQPTPYSAIKDAIKAAAKGPMAGILAYTEDEVVSTDFLSDTHSSIFDAKAGI

ALNDNFVKLISWYDNEYGYSNRVVDLVRYMFSRDK

>sp|P80109|PHLD_BOVIN Phosphatidylinositol-glycan-specific phospholipase D OS=Bos taurus OX=9913 GN=GPLD1 PE=1 SV=1

MSAFRFWSGLLMLLGFLCPRSSPCGISTHIEIGHRALEFLHLQDGSINYKELLLRHQDAY

QAGSVFPDSFYPSICERGQFHDVSESTHWTPFLNASVHYIRKNYPLPWDEDTEKLVAFLF

GITSHMVADVNWHSLGIEQGFLRTMAAIDFHNSYPEAHPAGDFGGDVLSQFEFKFNYLSR

HWYVPAEDLLGIYRELYGRIVITKKAIVDCSYLQFLEMYAEMLAISKLYPTYSVKSPFLV

EQFQEYFLGGLEDMAFWSTNIYHLTSYMLKNGTSNCNLPENPLFITCGGQQNNTHGSKVQ

KNGFHKNVTAALTKNIGKHINYTKRGVFFSVDSWTMDSLSFMYKSLERSIREMFIGSSQP

LTHVSSPAASYYLSFPYTRLGWAMTSADLNQDGYGDLVVGAPGYSHPGRIHVGRVYLIYG

NDLGLPRIDLDLDKEAHGILEGFQPSGRFGSAVAVLDFNVDGVPDLAVGAPSVGSEKLTY

TGAVYVYFGSKQGQLSSSPNVTISCQDTYCNLGWTLLAADVNGDSEPDLVIGSPFAPGGG

KQKGIVAAFYSGSSYSSREKLNVEAANWMVKGEEDFAWLGYSLHGVNVNNRTLLLAGSPT

WKDTSSQGHLFRTRDEKQSPGRVYGYFPPICQSWFTISGDKAMGKLGTSLSSGHVMVNGT

RTQVLLVGAPTQDVVSKVSFLTMTLHQGGSTRMYELTPDSQPSLLSTFSGNRRFSRFGGV

LHLSDLDNDGLDEIIVAAPLRITDATAGLMGEEDGRVYVFNGKQITVGDVTGKCKSWVTP

CPEEKAQYVLISPEAGSRFGSSVITVRSKEKNQVIIAAGRSSLGARLSGVLHIYRLGQD

>sp|P00435|GPX1_BOVIN Glutathione peroxidase 1 OS=Bos taurus OX=9913 GN=GPX1 PE=1 SV=3

MCAAQRSAAALAAAAPRTVYAFSARPLAGGEPFNLSSLRGKVLLIENVASLUGTTVRDYT

QMNDLQRRLGPRGLVVLGFPCNQFGHQENAKNEEILNCLKYVRPGGGFEPNFMLFEKCEV

NGEKAHPLFAFLREVLPTPSDDATALMTDPKFITWSPVCRNDVSWNFEKFLVGPDGVPVR

RYSRRFLTIDIEPDIETLLSQGASA

>sp|Q148C9|HEBP1_BOVIN Heme-binding protein 1 OS=Bos taurus OX=9913 GN=HEBP1 PE=2 SV=1

MLGMIKNSLFGSVETWPWQVLSKGSKGDVFYEERACEGGKFATVEVTDKPVDEALREAMP

KVMKYVGGSNDKGLGMGMTVPISFAVFPSDDGDLQKKLKVWFRIPNKFQSDPPAPSDDSI

KIEDREGITVYSTQFGGYAKAADYAAQAAQLRSALESTAKYQTDFYFCTGYDPPMKPYGR

RNEVWLVKSSE

>sp|Q95M18|ENPL_BOVIN Endoplasmin OS=Bos taurus OX=9913 GN=HSP90B1 PE=2 SV=1

MRALWVLGLCCVLLTFGSVRADDEVDVDGTVEEDLGKSREGSRTDDEVVQREEEAIQLDG

LNASQIRELREKSEKFAFQAEVNRMMKLIINSLYKNKEIFLRELISNASDALDKIRLISL

TDENALAGNEELTVKIKCDKEKNLLHVTDTGVGMTREELVKNLGTIAKSGTSEFLNKMTE

AQEDGQSTSELIGQFGVGFYSAFLVADKVIVTSKHNNDTQHIWESDSNEFSVIADPRGNT

LGRGTTITLVLKEEASDYLELDTIKNLVKKYSQFINFPIYVWSSKTETVEEPAEEEEAAK

EDKEESDDEAAVEEEEDEKKPKTKKVEKTVWDWELMNDIKPIWQRPSKEVEEDEYKAFYK

SFSKESDDPMAYIHFTAEGEVTFKSILFVPTSAPRGLFDEYGSKKSDYIKLYVRRVFITD

DFHDMMPKYLNFVKGVVDSDDLPLNVSRETLQQHKLLKVIRKKLVRKTLDMIKKIADEKY

NDTFWKEFGTNIKLGVIEDHSNRTRLAKLLRFQSSHHPSDMTSLDQYVERMKEKQDKIYF

MAGASRKEAESSPFVERLLKKGYEVIYLTEPVDEYCIQALPEFDGKRFQNVAKEGVKFDE

SEKSKESREAVEKEFEPLLNWMKDKALKDKIEKAVVSQRLTESPCALVASQYGWSGNMER

IMKAQAYQTGKDISTNYYASQKKTFEINPRHPLIRDMLRRVKEDEDDKTVSDLAVVLFET

ATLRSGYLLPDTKAYGDRIERMLRLSLNIDPDAKVEEEPEEEPEETTEDTAEDTEQDEEE

EMDAGTDEEEQETAEKSTAEKDEL

>sp|Q2TBX4|HSP13_BOVIN Heat shock 70 kDa protein 13 OS=Bos taurus OX=9913 GN=HSPA13 PE=2 SV=1

MAGEMTILGSAVLTLLLAGYLAQQYLPLPTPKVIGIDLGTTYCSVGVFFPGTGKVKVIPD

ENGHISIPSMVSFTDDDVYVGYESLELADSNPQNTIYDAKRFIGKVFTPEELEAEIGRYP

FKVLNKNGMVEFSVTSNETITVSPEYVGSRLLLKLKEMAEEYLGMPVANAVISVPAEFDL

KQRNSTIQAANLAGLKILRVINEPTAAAMAYGLHKAEVFHVLVIDLGGGTLDVSLLNKQG

GMFLTRAMSGNNKLGGQDFNQRLLQYLYKQIYQTYGFLPSRKEEIHRLRQAVEMVKLNLT

LHETAQMSVLLTVEENDRKGPPTSDSELPKDKFSQANDPHVDSMFGANLSEKKNGEGQVL

FETEISRKLFDTLNEDLFQKILVPIQQVLKEGHLEKTEIDEVVLVGGSTRIPRIRQVIQE

FFGKDPNTSVDPDLAVVTGVAIQAGIDGGSWPLQVSALEIPNKHLQKTNFN

>sp|Q3SWY2|ILK_BOVIN Integrin-linked protein kinase OS=Bos taurus OX=9913 GN=ILK PE=2 SV=1

MDDIFTQCREGNAVAVRLWLDNTENDLNQGDDHGFSPLHWACREGRSAVVEMLIMRGARI

NVMNRGDDTPLHLAASHGHRDIVQKLLQYKADINAVNEHGNVPLHYACFWGQDQVAEDLV

ANGALVSICNKYGEMPVDKAKPPLRELLRERAEKMGQNLNRIPYKDTFWKGTTRTRPRNG

TLNKHSGIDFKQLNFLAKLNENHSGELWKGRWQGNDIVVKMLKVRDWSTRKSRDFNEECP

RLRIFSHPNVLPVLGACQSPPAPHPTLITHWMPYGSLYNVLHEGTNFVVDQSQAVKFALD

MARGMAFLHTLEPLIPRHALNSRSVMIDEDMTARISMADVKFSFQCPGRMYAPAWVAPEA

LQKKPEDTNRRSADMWSFAVLLWELVTREVPFADLSNMEIGMKVALEGLRPTIPPGISPH

VCKLMKICMNEDPAKRPKFDMIVPILEKMQDK

>sp|Q8SPJ1|PLAK_BOVIN Junction plakoglobin OS=Bos taurus OX=9913 GN=JUP PE=2 SV=1

MEVMNLIEQPIKVTEWQQTYTYDSGIHSGANTCVPSLSSKGLIEEDEACGRQYTLKKTTT

YTQSVPPGQGDLEYQMSTTARAKRVREAMCPGVTGEDSSLLLTTQVEGQTTNLQRLAEPS

QLLKSAIVHLINYQDDAELATRALPELTKLLNDEDPVVVTKAAMIVNQLSKKEASRRALM

GSPQLVAAVVRTMQNTSDLDTARCTTSILHNLSHHREGLLAIFKSGGIPALVRMLSSPVE

SVLFYAITTLHNLLLYQEGAKMAVRLADGLQKMVPLLNKNNPKFLAITTDCLQLLAYGNQ

ESKLIILANGGPQALVQIMRNYSYEKLLWTTSRVLKVLSVCPSNKPAIVEAGGMQALGKH

LTSNSPRLVQNCLWTLRNLSDVATKQEGLESVLKILVNQLSVDDVNVLTCATGTLSNLTC

NNSKNKTLVTQNSGVEALIHAILRAGDKDDITEPAVCALRHLTSRHPEAEMAQNSVRLNY

GIPAIVKLLNQPNQWPLVKATIGLIRNLALCPANHAPLQEAAVIPRLVQLLVKAHQDAQR

HVAAGTQQPYTDGVRMEEIVEGCTGALHILARDPMNRMEIFRLNTIPLFVQLLYSSVENI

QRVAAGVLCELAQDKEAADAIDAEGASAPLMELLHSRNEGTATYAAAVLFRISEDKNPDY

RKRVSVELTNSLFKHDPAAWEAAQSMIPMNEPYADDMDATYRPMYSSDVPMDPLEMHMDM

DGDYPIDTYSDGLRPPYATADHMLA

>sp|P05785|K1C14_BOVIN Keratin, type I cytoskeletal 14 (Fragment) OS=Bos taurus OX=9913 GN=KRT14 PE=2 SV=1

SVEEQLAQLRCEMEQQNQEYKILLDVKTRLEQEIATYRRLLEGEDAHLSSSQFSSGSQSS

RDVSSSRQVRTKVVDVHDGKVVFTHEQIVRTKN

>sp|Q2TBI0|LBP_BOVIN Lipopolysaccharide-binding protein OS=Bos taurus OX=9913 GN=LBP PE=2 SV=1

MVTSTGTLPSLLLGTLLTFTSGALGANPGLVVRITDQGLEYVAQEELLALQSKLHKVTLP

DFNGDVRIKHFGSVDYRFHSLNIQSCKLLGSALKLLPNQGLHFSISDSFIQVTGDWKVRK

RILRLDGSFDVKVKGITISVNLLLDSEPSGRPKVAVSSCSSHIRDVEVHISGDLGWLLNL

FHNQIESRFRRVLESKICEIIEDSVTSELQPYLQTLPVTTEIDHLAGLDYSLMGAPQATA

QMLDVMFKGEIFSRDDRFPVAFLAPVMNLPEEHSRMVYFAISDYAFNTASLVYHKAGFLN

FTITDDVIPPDSSIRQNTKSFRAFVPRIARLYPNTNLELQGAVISAPCLNFSPGNLSTAA

QMEIEAFVLLPNSVKEPVFRLSVATNVSAMLTFNTSKITGFLEPGKIQVELKESKVGRFN

VELLEALLNYYLLNNFYPKVNDKLAEGFPLPLLRKIQLYDPILQIHKDFLFLGTNVRYLR

V

>sp|P11151|LIPL_BOVIN Lipoprotein lipase OS=Bos taurus OX=9913 GN=LPL PE=1 SV=2

MESKALLLLALSVCLQSLTVSRGGLVAADRITGGKDFRDIESKFALRTPEDTAEDTCHLI

PGVTESVANCHFNHSSKTFVVIHGWTVTGMYESWVPKLVAALYKREPDSNVIVVDWLSRA

QQHYPVSAGYTKLVGQDVAKFMNWMADEFNYPLGNVHLLGYSLGAHAAGIAGSLTNKKVN

RITGLDPAGPNFEYAEAPSRLSPDDADFVDVLHTFTRGSPGRSIGIQKPVGHVDIYPNGG

TFQPGCNIGEALRVIAERGLGDVDQLVKCSHERSVHLFIDSLLNEENPSKAYRCNSKEAF

EKGLCLSCRKNRCNNMGYEINKVRAKRSSKMYLKTRSQMPYKVFHYQVKIHFSGTESNTY

TNQAFEISLYGTVAESENIPFTLPEVSTNKTYSFLLYTEVDIGELLMLKLKWISDSYFSW

SNWWSSPGFDIGKIRVKAGETQKKVIFCSREKMSYLQKGKSPVIFVKCHDKSLNRKSG

>sp|Q2TBQ8|6PGL_BOVIN 6-phosphogluconolactonase OS=Bos taurus OX=9913 GN=PGLS PE=2 SV=1

MAAPAPRLISVFSSPQELGASLAQLVVQQAACCLADAGARFTLGLSGGSLVSMLARELPA

AAAPAGPASLARWTLGFCDERLVPFEHAESTYGLYRTHLLSKLPIFDSQVITINPALPVE

EAAEDYAKKLRQAFQGDSIPVFDLLILGVGPDGHTCSLFPDHPLLQEREKIVAPISDSPK

PPPQRVTLTLPVLNAARTVIYVATGEGKAAILKRILEDKEENPLPAALVQPSAGKLCWFL

DEAAARLLTVPFEKHSTL

>sp|Q32PI5|2AAA_BOVIN Serine/threonine-protein phosphatase 2A 65 kDa regulatory subunit A alpha isoform OS=Bos taurus OX=9913 GN=PPP2R1A PE=1 SV=1

MAAADGDDSLYPIAVLIDELRNEDVQLRLNSIKKLSTIALALGVERTRSELLPFLTDTIY

DEDEVLLALAEQLGTFTTLVGGPEYVHCLLPPLESLATVEETVVRDKAVESLRAISHEHS

PSDLEAHFVPLVKRLAGGDWFTSRTSACGLFSVCYPRVSSAVKAELRQYFRNLCSDDTPM

VRRAAASKLGEFAKVLELDNVKSEIIPMFSNLASDEQDSVRLLAVEACVNIAQLLPQEDL

EALVMPTLRQAAEDKSWRVRYMVADKFTELHKAVGPEITKTDLVPAFQNLMKDCEAEVRA

AASHKVKEFCENLSADCRENVIMTQILPCIKELVSDANQHVKSALASVIMGLSPILGKDS

TIEHLLPLFLAQLKDECPEVRLNIISNLDCVNEVIGIRQLSQSLLPAIVELAEDAKWRVR

LAIIEYMPLLAGQLGVEFFDEKLNSLCMAWLVDHVYAIREAATSNLKKLVEKFGKEWAHA

TIIPKVLAMSGDPNYLHRMTTLFCINVLSEVCGQDITTKHMLPTVLRMAGDPVANVRFNV

AKSLQKIGPILDNSTLQSEVKPVLEKLTQDQDVDVKYFAQEALTVLSLA

>sp|P79334|PYGM_BOVIN Glycogen phosphorylase, muscle form OS=Bos taurus OX=9913 GN=PYGM PE=2 SV=3

MSRPLTDQEKRKQISVRGLAGVENVTELKKNFNRHLHFTLVKDRNVATPRDYYFALAYTV

RDHLVGRWIRTQQHYYEKDPKRIYYLSLEFYIGRTLQNTMVNLALENACDEATYQLGLDM

EELEEIEEDAGLGNGGLGRLAACFLDSMATLGLAAYGYGIRYEFGIFNQKISGGWQMEEA

DDWLRYGNPWEKARPEFTLPVHFYGRVEHTSQGAKWVDTQVVLAMPYDTPVPGYRNNVVN

TMRLWSAKAPNDFNLKDFNVGGYIQAVLDRNLAENISRVLYPNDNFFEGKELRLKQEYFV

VAATLQDIIRRFKSSKFGCLDPVRTNFDAFPDKVAIQLNDTHPSLAIPELMRILVDQERL

EWEKAWEVTVKTCAYTNHTVLPEALERWPVHLIETLLPRHLQIIYEINQRFLNRVAAAFP

GDVDRLRRMSLVEEGAVKRINMAHLCIAGSHAVNGVARIHSEILKKTIFKDFYELEPHKF

QNKTNGITPRRWLVMCNPGLAEIIAERIGEEYIADLDQLRKLLSYVDDESFIRDVAKVKQ

ENKLKFSAYLEKEYKVHINPNSLFDIQVKRIHEYKRQLLNCLHVITLYNRIKKEPNKFFV

PRTVMIGGKAAPGYHMAKMIIKLITAIGDVVNHDPVVGDRLRVIFLENYRVSLAEKVIPA

ADLSEQISTAGTEASGTGNMKFMLNGALTIGTMDGANVEMAEEAGEENFFIFGMRVEDVE

RLDQKGYNAQEYYDRIPELRHVIDQLSSGFFSPKQPDLFKDIVNMLMHHDRFKVFADYEE

YIKCQERVSALYKNPREWTRMVIRNIATSGKFSSDRTIAQYAREIWGVEPTRQRMPAPDE

KI

>sp|Q3T0Z7|DHPR_BOVIN Dihydropteridine reductase OS=Bos taurus OX=9913 GN=QDPR PE=2 SV=1

MAAAAGEARRVLVYGGRGALGSRCVQAFRARNWWVASIDVQENEEASANVVVKMTDSFTE

QADQVTAEVGKLLGTEKVDAILCVAGGWAGGNAKSKSLFKNCDLMWKQSVWTSTISSHLA

TKHLKEGGLLTLAGARAALDGTPGMIGYGMAKAAVHQLCQSLAGKSSGLPPGAAAVALLP

VTLDTPVNRKSMPEADFSSWTPLEFLVETFHDWITEKNRPSSGSLIQVVTTEGKTELTAA

SP

>sp|Q3MHP2|RB11B_BOVIN Ras-related protein Rab-11B OS=Bos taurus OX=9913 GN=RAB11B PE=2 SV=3

MGTRDDEYDYLFKVVLIGDSGVGKSNLLSRFTRNEFNLESKSTIGVEFATRSIQVDGKTI

KAQIWDTAGQERYRAITSAYYRGAVGALLVYDIAKHLTYENVERWLKELRDHADSNIVIM

LVGNKSDLRHLRAVPTDEARAFAEKNNLSFIETSALDSTNVEEAFKNILTEIYRIVSQKQ

IADRAAHDESPGNNVVDISVPPTTDGQKPNKLQCCQNL

>sp|Q2HJH2|RAB1B_BOVIN Ras-related protein Rab-1B OS=Bos taurus OX=9913 GN=RAB1B PE=2 SV=1

MNPEYDYLFKLLLIGDSGVGKSCLLLRFADDTYTESYISTIGVDFKIRTIELDGKTIKLQ

IWDTAGQERFRTITSSYYRGAHGIIVVYDVTDQESYANVKQWLQEIDRYASENVNKLLVG

NKSDLTTKKVVDNTTAKEFADSLGIPFLETSAKNATNVEQAFMTMAAEIKKRMGPGAASG

GERPNLKIDSTPVKQAGGGCC

>sp|P62992|RS27A_BOVIN Ubiquitin-40S ribosomal protein S27a OS=Bos taurus OX=9913 GN=RPS27A PE=1 SV=2

MQIFVKTLTGKTITLEVEPSDTIENVKAKIQDKEGIPPDQQRLIFAGKQLEDGRTLSDYN

IQKESTLHLVLRLRGGAKKRKKKSYTTPKKNKHKRKKVKLAVLKYYKVDENGKISRLRRE

CPSDECGAGVFMASHFDRHYCGKCCLTYCFNKPEDK

>sp|Q58D31|DHSO_BOVIN Sorbitol dehydrogenase OS=Bos taurus OX=9913 GN=SORD PE=1 SV=3

MAAAKPENLSLVVHGPGDLRLENYPIPEPGPNEVLLKMHSVGICGSDVHYWQHGRIGDFV

VKKPMVLGHEASGTVVKVGSLVRHLQPGDRVAIEPGAPRETDEFCKIGRYNLSPTIFFCA

TPPDDGNLCRFYKHNANFCYKLPDNVTFEEGALIEPLSVGIHACRRAGVTLGNKVLVCGA

GPIGLVSLLAAKAMGAAQVVVTDLSASRLSKAKEVGADFILQISNESPQEIAKKVEGLLG

SKPEVTIECTGVETSIQAGIYATHSGGTLVLVGLGSEMTSVPLVHAATREVDIKGVFRYC

NTWPMAISMLASKSVNVKPLVTHRFPLEKALEAFETSKKGLGLKVMIKCDPNDQNP

>sp|Q28178|TSP1_BOVIN Thrombospondin-1 OS=Bos taurus OX=9913 GN=THBS1 PE=2 SV=2

MGLAWGLGVLLLLHACGSNRIPESGGDNSVFDIFELTGAARKRSGRRLVKGPDPSSPAFR

IEDANLIPPVPDKKFQDLVDAVRAEKGFLLLASLRQMKKTRGTLLAVERKDHSGQVFSVI

SNGKAGTLDLSLTVQGKQHVVSVEEALLATGQWKSITLFVQEDRAQLYIDCEKMENAELD

VPIQSIFTRDLASIARLRIAKGGVNDNFQGVLQNVRFVFGTTPEDILRNKGCSSSTSVFV

TLDNNVVNGSSPAIRTDYIGHKTKDLQAICGISCDELSSMVLELRGLRTIVTTLQDSIRK

VTEENKELANELRRPPLCYHNGVQYRTGDEWTVDSCTECRCQNSVTICKKVSCPIMPCSN

ATVPDGECCPRCWPSDSADDGWSPWSEWTSCSVTCGNGIQQRGRSCDSLNNRCEGSSVQT

RTCHIQECDKRFKQDGGWSHWSPWSSCSVTCGDGVITRIRLCNSPSPQMNGKPCEGKARE

TKACQKDSCPINGGWGPWSPWDICSVTCGGGVQKRSRLCNNPKPQFGGKDCVGDVTENQI

CNKQDCPIDGCLSNPCFAGVQCTSYPDGSWKCGACPPGYSGDGVECKDVDECKEVPDACF

NHNGEHRCENTDPGYNCLPCPPRFTGSQPFGRGVEHATANKQVCKPRNPCTDGTHDCNKN

AKCNYLGHYSDPMYRCECKPGYAGNGIICGEDTDLDGWPNEDLLCVANATYHCRKDNCPN

LPNSGQEDYDKDGIGDACDDDDDNDKIPDDRDNCPFHYNPAQYDYDRDDVGDRCDNCPYN

HNPDQADTDNNGEGDACAADIDGDSILNERDNCQYVYNVDQKDTDMDGVGDQCDNCPLEH

NPDQLDSDSDRIGDTCDNNQDIDEDGHQNNLDNCPYVPNANQADHDKDGKGDACDHDDDN

DGIPDDRDNCRLVPNPDQKDSDGDGRGDACKDDFDQDKVPDIDDICPENVDISETDFRRF

QMIPLDPKGTSQNDPNWVVRHQGKELVQTVNCDPGLAVGYDEFNAVDFSGTFFINTERDD

DYAGFVFGYQSSSRFYVVMWKQVTQSYWDTNPTRAQGYSGLSVKVVNSTTGPGEHLRNAL

WHTGNTSGQVRTLWHDPRHIGWKDFTAYRWHLSHRPKTGFIRVVMYEGKKIMADSGPIYD

KTYAGGRLGLFVFSQEMVFFSDLKYECRDS

>sp|P63048|RL40_BOVIN Ubiquitin-60S ribosomal protein L40 OS=Bos taurus OX=9913 GN=UBA52 PE=1 SV=2

MQIFVKTLTGKTITLEVEPSDTIENVKAKIQDKEGIPPDQQRLIFAGKQLEDGRTLSDYN

IQKESTLHLVLRLRGGIIEPSLRQLAQKYNCDKMICRKCYARLHPRAVNCRKKKCGHTNN

LRPKKKVK

>sp|P0CG53|UBB_BOVIN Polyubiquitin-B OS=Bos taurus OX=9913 GN=UBB PE=1 SV=1

MQIFVKTLTGKTITLEVEPSDTIENVKAKIQDKEGIPPDQQRLIFAGKQLEDGRTLSDYN

IQKESTLHLVLRLRGGMQIFVKTLTGKTITLEVEPSDTIENVKAKIQDKEGIPPDQQRLI

FAGKQLEDGRTLSDYNIQKESTLHLVLRLRGGMQIFVKTLTGKTITLEVEPSDTIENVKA

KIQDKEGIPPDQQRLIFAGKQLEDGRTLSDYNIQKESTLHLVLRLRGGMQIFVKTLTGKT

ITLEVEPSDTIENVKAKIQDKEGIPPDQQRLIFAGKQLEDGRTLSDYNIQKESTLHLVLR

LRGGC

>sp|P0CH28|UBC_BOVIN Polyubiquitin-C OS=Bos taurus OX=9913 GN=UBC PE=1 SV=1

MQIFVKTLTGKTITLEVEPSDTIENVKGKIQEKEGIPPDQQRLIFAGKQLEDGRTLSDYN

IQKESTLHLVLRLRGGMQIFVKTLTGKTITLEVEPSDTIENVKAKIQDKEGIPPDQQRLI

FAGKQLEDGRTLSDYNIQKESTLHLVLRLRGGMQIFVKTLTGKTITLEVEPSDTIENVKA

KIQDKEGIPPDQQRLIFAGKQLEDGRTLSDYNIQKESTLHLVLRLRGGMQIFVKTLTGKT

ITLEVEPSDTIENVKAKIQDKEGIPPDQQRLIFAGKQLEDGRTLSDYNIQKESTLHLVLR

LRGGMQIFVKTLTGKTITLEVEPSDTIENVKAKIQDKEGIPPDQQRLIFAGKQLEDGRTL

SDYNIQKESTLHLVLRLRGGMQIFVKTLTGKTITLEVEPSDTIENVKAKIQDKEGIPPDQ

QRLIFAGKQLEDGRTLSDYNIQKESTLHLVLRLRGGMQIFVKTLTGKTITLEVEPSDTIE

NVKAKIQDKEGIPPDQQRLIFAGKQLEDGRTLSDYNIQKESTLHLVLRLRGGMQIFVKTL

TGKTITLEVEPSDTIENVKAKIQDKEGIPPDQQRLIFAGKQLEDGRTLSDYNIQKESTLH

LVLRLRGGMQIFVKTLTGKTITLEVEPSDTIENVKAKIQDKEGIPPDQQRLIFAGKQLED

GRTLSDYNIQKESTLHLVLRLRGGVLSSPF

>sp|Q58CQ9|VNN1_BOVIN Pantetheinase OS=Bos taurus OX=9913 GN=VNN1 PE=1 SV=1

MIMSQLLNYVAVLFFCVSRASSLDTFIAAVYEHAVILPNATLVPVSPEEALAVMNRNLDL

LEGAVTSASKQGAHIIVTPEDGIYGFNFTRESIYPYLEDIPDPQVNWIPCNNPDRFGHTP

VQQRLSCLAKDNSIYIVANIGDKKSCNASDPQCPPDGRYQYNTDVVFDSKGKLVARYHKQ

NLFLNEDQFNAPKEPEVVTFNTTFGKFGIFTCFDILFHDPAVTLVRDSHVDTILFPTAWM

NVLPHLSAIEFHSAWAMGMRVNFLASNLHYPLKKMTGSGIYAPDSPRAFHYDMKTEEGKL

LLAQLDSHPHPTPVVNWTSYASGVEAHSVGNQEFTGIIFFDEFTFLELKEIGGNYTVCQR

DLCCHLSYKMSEKRSDEVYALGAFDGLHTVEGSYYLQICTLLKCKTTDLHTCGDSVETAS

TRFEMFSLSGTFGTQYVFPEVLLSEIQLAPGEFQVSNDGRLFSLKPTSGPVLTVTLFGRL

YEKDSAPNTLSDLTTQALRLNPKTDAWKSK

>sp|P62261|1433E_BOVIN 14-3-3 protein epsilon OS=Bos taurus OX=9913 GN=YWHAE PE=2 SV=1

MDDREDLVYQAKLAEQAERYDEMVESMKKVAGMDVELTVEERNLLSVAYKNVIGARRASW

RIISSIEQKEENKGGEDKLKMIREYRQMVETELKLICCDILDVLDKHLIPAANTGESKVF

YYKMKGDYHRYLAEFATGNDRKEAAENSLVAYKAASDIAMTELPPTHPIRLGLALNFSVF

YYEILNSPDRACRLAKAAFDDAIAELDTLSEESYKDSTLIMQLLRDNLTLWTSDMQGDGE

EQNKEALQDVEDENQ

>sp|Q3SZI4|1433T_BOVIN 14-3-3 protein theta OS=Bos taurus OX=9913 GN=YWHAQ PE=1 SV=1

MEKTELIQKAKLAEQAERYDDMATCMKAVTEQGAELSNEERNLLSVAYKNVVGGRRSAWR

VISSIEQKTDTSDKKLQLIKDYREKVESELRSICTTVLELLDKYLIANATNPESKVFYLK

MKGDYFRYLAEVACGDDRKQTIDNSQGAYQEAFDISKKEMQPTHPIRLGLALNFSVFYYE

ILNNPELACTLAKTAFDEAIAELDTLNEDSYKDSTLIMQLLRDNLTLWTSDSAGEECDAA

EGAEN

>sp|A6QLP2|SAHH3_BOVIN Adenosylhomocysteinase 3 OS=Bos taurus OX=9913 GN=AHCYL2 PE=1 SV=1

MSVQVVSAAAAAKVPEVELKDLSPSEAEPQLGLSTAAVSAMAPPAGGGDPEAPAPAAERP

PAPGPGSGPAAALSPAAGKVPQASAMKRSDPHHQHQRHRDGGEALVSPDGTVTEAPRTVK

KQIQFADQKQEFNKRPTKIGRRSLSRSISQSSTDSYSSAASYTDSSDDETSPRDKQQKNS

KGNSDFCVKNIKQAEFGRREIEIAEQEMPALMALRKRAQGEKPLAGAKIVGCTHITAQTA

VLMETLGALGAQCRWAACNIYSTLNEVAAALAESGFPVFAWKGESEDDFWWCIDRCVNVE

GWQPNMILDDGGDLTHWIYKKYPNMFKKIKGIVEESVTGVHRLYQLSKAGKLCVPAMNVN

DSVTKQKFDNLYCCRESILDGLKRTTDMMFGGKQVVVCGYGEVGKGCCAALKAMGSIVYV

TEIDPICALQACMDGFRLVKLNEVIRQVDIVITCTGNKNVVTREHLDRMKNSCIVCNMGH

SNTEIDVASLRTPELTWERVRSQVDHVIWPDGKRIILLAEGRLLNLSCSTVPTFVLSITA

TTQALALIELYNAPEGRYKQDVYLLPKKMDEYVASLHLPTFDAHLTELTDEQAKYLGLNK

NGPFKPNYYRY

>sp|P48644|AL1A1_BOVIN Retinal dehydrogenase 1 OS=Bos taurus OX=9913 GN=ALDH1A1 PE=1 SV=3

MSSSAMPDVPAPLTNLQFKYTKIFINNEWHSSVSGKKFPVFNPATEEKLCEVEEGDKEDV

DKAVKAARQAFQIGSPWRTMDASERGRLLNKLADLIERDHLLLATMEAMNGGKLFSNAYL

MDLGGCIKTLRYCAGWADKIQGRTIPMDGNFFTYTRSEPVGVCGQIIPWNFPLLMFLWKI

GPALSCGNTVVVKPAEQTPLTALHMGSLIKEAGFPPGVVNIVPGYGPTAGAAISSHMDVD

KVAFTGSTEVGKLIKEAAGKSNLKRVSLELGGKSPCIVFADADLDNAVEFAHQGVFYHQG

QCCIAASRLFVEESIYDEFVRRSVERAKKYVLGNPLTPGVSQGPQIDKEQYEKILDLIES

GKKEGAKLECGGGPWGNKGYFIQPTVFSDVTDDMRIAKEEIFGPVQQIMKFKSLDDVIKR

ANNTFYGLSAGIFTNDIDKAITVSSALQSGTVWVNCYSVVSAQCPFGGFKMSGNGRELGE

YGFHEYTEVKTVTIKISQKNS

>sp|P27479|LOX15_BOVIN Polyunsaturated fatty acid lipoxygenase ALOX15 OS=Bos taurus OX=9913 GN=ALOX15 PE=1 SV=3

MGLYRVRVSTGSSFCAGSNNQVHLWLVGEHGEAALGWRLRPARGKEVEFQVDVSEYLGRL

LFVKLRKRHLLSDDAWFCNWISVQGPGASGNEFRFPCYRWVEGDGILSLPEGTGRTVVDD

PQGLFKKHREEELAERRKLYRWGNWKDGLILNIAGATINDLPVDERFLEDKRIDFEASLT

KGLADLAIKDSLNILTCWKSLDDFNRIFWCGQSKLAERVRDSWKEDALFGYQFLNGTNPM

LLRRSVRLPARLEFPPGMGELQAELEKELQQGTLFEADFSLLDGIKANVILCTQQYVAAP

LVMLKLQPDGKLLPMAIQLQLPHKGSPPPPLFLPTDPPMTWLLAKCWVRSSDFQLHELHS

HLLRGHLVAEVIAVATMRCLPSIHPMFKLLIPHLRYTMEINIRARTGLVSDSGVFDQVVS

TGGGGHVELLQRAGAFLTYSSFCPPDDLADRGLLGVKSSFYAQDALRLWEILSRYVEGIV

SLHYKTDESVRDDIELQAWCRDITEIGLLGAQDRGFPVTLQSKDQLCHFVTMCIFTCTGQ

HSSTHLGQLDWYSWVPNAPCTMRLPPPTTKDVTLEKVMATLPNFHQASLQMSITWQLGRR

QPIMVALGQHEEEYFSGPEPKAVLKKFREELAALEKDIEIRNAQLDWPYEYLRPSLVENS

VAI

>sp|P48034|AOXA_BOVIN Aldehyde oxidase 1 OS=Bos taurus OX=9913 GN=AOX1 PE=1 SV=2

MEGGSELLFYVNGRKVTEKNVDPETMLLPYLRKKLRLTGTKYGCGGGGCGACTVMISRYN

PITKKIRHYPANACLTPICSLYGAAVTTVEGIGSTKTRIHPVQERIAKCHGTQCGFCTPG

MVMSLYTLLRNHPEPTLTQLNDALGGNLCRCTGYRPIINACKTFCKTSGCCQSKENGVCC

LDQGMNGLPEFEEGNETSLKLFSEEEFLPLDPTQELIFPPELMTMAEKKTQKTRIFGSDR

MTWISPVTLKELLEAKVKYPQAPVVMGNTSVGPDMKFKGIFHPVIISPDRIEELSVVNYT

DNGLTLGAAVSLAEVKDILANVTRKLPEEKTQMYHALLKHLETLAGPQIRNMASLGGHIV

SRHPDSDLNPLLAVGNCTLNLLSKEGRRQIPLNEQFLRKCPSADLKPEEILISVNIPYSR

KWEFVSAFRQAQRQQNALAIVNSGMRVCFGKGDGIIRELSIAYGGVGPTTILANNSCQKL

IGRPWNEEMLDAACRLILDEVSLPGSAPGGRVEFKRTLIVSFLFKFYLEVSQILKGMDLV

HYPSLASKYESALEDLHSRHYWSTLKYQNADLKQLSQDPIGHPIMHLSGIKHATGEAIYC

DDMPVVDRELFLTFVTSSRAHAKIVSIDVSAALSLPGVVDILTGEHLPGINTTFGFLTDA

DQLLSTDEVSCVGQLVCAVIADSEVQARRAAQQVKIVYQDLEPVILTIEEAIQNKSFFEP

ERKLEYGNVDEAFKMVDQILEGEIHMGGQEHFYMETQSMLVVPKGEDREIDVYVSAQFPK

YIQDITASVLKVSANKVMCHVKRVGGAFGGKVTKTGVLAAITAFAANKHGRPVRCILERG

EDILITGGRHPYLGKYKAGFMNDGRILALDMEHYNNAGAFLDESLFVIEMGLLKLENAYK

FPNLRCRGWACRTNLPSNTALRGFGFPQAGLITEACITEVAAKCGLPPEKVRMINMYKEI

DQTPYKQEINTKNLTQCWKECMATSSYTLRKAAVEKFNSENYWKKKGLAMVPLKYPIGLG

SVAAGQAAALVHIYLDGSVLVTHGGIEMGQGVHTKMIQVVSRELRMPLSSIHLRGTSTET

IPNTNPSGGSVVADLNGLAVKDACQTLLKRLKPIISKNPKGTWKDWAQAAFNESISLSAT

GYFRGYESNINWETGEGHPFEYFVYGAACSEVEIDCLTGAHKNIRTDIVMDVGYSINPAL

DVGQIEGAFIQGMGLYTIEELNYSPQGVLYTRGPNQYKIPAICDIPMELHISFLPPSENS

NTLYSSKGLGESGIFLGCSVFFAIHDAIRAARQERGLPGPLRLNSPLTPEKIRMACEDKF

TKMIPRDEPGSYVPWSVPI

>sp|Q3T004|SAMP_BOVIN Serum amyloid P-component OS=Bos taurus OX=9913 GN=APCS PE=2 SV=1

MNKLMSWVSVLIILPEAFAQTDLRGKVFVFPRESSTDHVTLITKLEKPLKNLTLCLRAYS

DLSRGYSLFSYNIHSKDNELLVFKNGIGEYSLYIGKTKVTVRATEKFPSPVHICTSWESS

TGIAEFWINGKPLVKRGLKQGYAVGAHPKIVLGQEQDSYGGGFDKNQSFMGEIGDLYMWD

SVLSPEEILLVYQGSSSISPTILDWQALKYEIKGYVIVKPMVWG

>sp|Q32PJ2|APOA4_BOVIN Apolipoprotein A-IV OS=Bos taurus OX=9913 GN=APOA4 PE=2 SV=1

MFLKAVVLSLALVAVTGAEAEVNADQVATVIWDYFSQLGNNAKKAVEHIQKSELTQQLNT

LFQDKLGEVSTYTDDLQKKLVPFATELHERLTKDSEKLKEEIRKELEDLRARLLPHATEV

SQKIGDNVRELQQRLGPYAEELRTQVDTQAQQLRRQLTPYVERMEKVMRQNLDQLQASLA

PYAEELQATVNQRVEELKGRLTPYADQLQTKIEENVEELRRSLAPYAQDVQGKLNHQLEG

LAFQMKKHAEELKAKISAKAEELRQGLVPLVNSVHGSQLGNAEDLQKSLAELSSRLDQQV

EDFRRTVGPYGETFNKAMVQQLDTLRQKLGPLAGDVEDHLSFLEKDLRDKVSSFFNTLKE

KESQAPALPAQEEMPVPLGG

>sp|P47865|AQP1_BOVIN Aquaporin-1 OS=Bos taurus OX=9913 GN=AQP1 PE=1 SV=3

MASEFKKKLFWRAVVAEFLAMILFIFISIGSALGFHYPIKSNQTTGAVQDNVKVSLAFGL

SIATLAQSVGHISGAHLNPAVTLGLLLSCQISVLRAIMYIIAQCVGAIVATAILSGITSS

LPDNSLGLNALAPGVNSGQGLGIEIIGTLQLVLCVLATTDRRRRDLGGSGPLAIGFSVAL

GHLLAIDYTGCGINPARSFGSSVITHNFQDHWIFWVGPFIGAALAVLIYDFILAPRSSDL

TDRVKVWTSGQVEEYDLDADDINSRVEMKPK

>sp|Q3SZF2|ARF4_BOVIN ADP-ribosylation factor 4 OS=Bos taurus OX=9913 GN=ARF4 PE=2 SV=3

MGLTISSLFSRLFGKKQMRILMVGLDAAGKTTILYKLKLGEIVTTIPTIGFNVETVEYKN

ICFTVWDVGGQDKIRPLWRHYFQNTQGLIFVVDSNDRERIQEGAEELQKMLQEDELRDAV

LLLFANKQDLPNAMAISEMTDKLGLQSLRNRTWYVQATCATQGTGLYEGLDWLSNELSKR

>sp|Q1JP79|ARC1A_BOVIN Actin-related protein 2/3 complex subunit 1A OS=Bos taurus OX=9913 GN=ARPC1A PE=2 SV=1

MSLHQFLLEPITCHAWNRDRTQIALSPNNHEVHIYKKNGGQWVKAHELKEHNGHITGIDW

APKSDRIVTCGADRNAYVWSQKDGVWKPTLVILRINRAATFVKWSPLENKFAVGSGARLI

SVCYFESENDWWVSKHIKKPIRSTVLSLDWHPNNVLLAAGSCDFKCRVFSAYIKEVDEKP

ASTPWGSKMPFGQLMSEFGGSGTGGWVHGVSFSASGSRLAWVSHDSTVSVADASKSVQVS

TLKTEFLPLLSVSFVSENSVVAAGHDCCPMLFNYDDRGCLTFVSKLDIPKQSIQRNMSAM

ERFRNMDKRATTEDRNTALETLHQNSITQVSIYEVDKQDCRKFCTTGIDGAMTIWDFKTL

ESSIQGLRIM

>sp|Q148J6|ARPC4_BOVIN Actin-related protein 2/3 complex subunit 4 OS=Bos taurus OX=9913 GN=ARPC4 PE=1 SV=3

MTATLRPYLSAVRATLQAALCLENFSSQVVERHNKPEVEVRSSKELLLQPVTISRNEKEK

VLIEGSINSVRVSIAVKQADEIEKILCHKFMRFMMMRAENFFILRRKPVEGYDISFLITN

FHTEQMYKHKLVDFVIHFMEEIDKEISEMKLSVNARARIVAEEFLKNF

>sp|Q3ZBN5|ASPN_BOVIN Asporin OS=Bos taurus OX=9913 GN=ASPN PE=2 SV=1

MKVYVLLVFLTLCSAKPLFHPSYLTLKNLMLKDMEDEGDSDADNSLFPTREPINPFFPFD

LFSTCPFGCQCYSRVVHCSDLGLSSVPSNIPFDTRMVDLQNNKIKEIKENDFKGLTSLYA

LILNNNKLTKIHPKAFLTTKKLRRLYLSHNQLSEIPLNLPKSLAELRIHDNKVKKIQKAT

FKGMNALHVLEMSANPLDNNGIEPGAFEGVTVFHIRIAEAKLTSIPKELPSTLLELHLDY

NKISVVELEDFKRYKDLQRLGLGNNRITDIENGSLANIPRVREIHLENNKLKKVPSGLQE

LKYLQIIFLHSNSITKVGVNDFCPTVPKMKKSLYSAISLSNNPVKYWEVQPATFRCVLSR

MSVQLGNFRK

>sp|Q08DA1|AT1A1_BOVIN Sodium/potassium-transporting ATPase subunit alpha-1 OS=Bos taurus OX=9913 GN=ATP1A1 PE=1 SV=1

MGKGVGRDKYEPAAVSEHGDKKKAKKERDMDELKKEVSMDDHKLSLDELHRKYGTDLSRG

LTTARAAEILARDGPNALTPPPTTPEWVKFCRQLFGGFSMLLWIGAVLCFLAYGIQAATE

EEPQNDNLYLGVVLSAVVIITGCFSYYQEAKSSKIMESFKNMVPQQALVIRNGEKMSINA

EEVVVGDLVEVKGGDRIPADLRIISANGCKVDNSSLTGESEPQTRSPDFTNENPLETRNI

AFFSTNCVEGTARGIVVYTGDRTVMGRIATLASGLEGGQTPIAAEIEHFIHIITGVAVFL

GVSFFILSLILEYTWLEAVIFLIGIIVANVPEGLLATVTVCLTLTAKRMARKNCLVKNLE

AVETLGSTSTICSDKTGTLTQNRMTVAHMWFDNQIHEADTTENQSGVSFDKTSATWLALS

RIAGLCNRAVFQANQDNLPILKRAVAGDASESALLKCIEVCCGSVKEMRERYTKIVEIPF

NSTNKYQLSIHKNANAGEPRHLLVMKGAPERILDRCSSILIHGKEQPLDEELKDAFQNAY

LELGGLGERVLGFCHLLLPDEQFPEGFQFDTDDVNFPVDNLCFVGLISMIDPPRAAVPDA

VGKCRSAGIKVIMVTGDHPITAKAIAKGVGIISEGNETVEDIAARLNIPVSQVNPRDARA

CVVHGSDLKDMTPEQLDDILKYHTEIVFARTSPQQKLIIVEGCQRQGAIVAVTGDGVNDS

PALKKADIGVAMGIAGSDVSKQAADMILLDDNFASIVTGVEEGRLIFDNLKKSIAYTLTS

NIPEITPFLIFIIANIPLPLGTVTILCIDLGTDMVPAISLAYEQAESDIMKRQPRNPQTD

KLVNERLISMAYGQIGMIQALGGFFTYFVIMAENGFLPNHLLGIRVTWDDRWINDVEDSY

GQQWTYEQRKIVEFTCHTAFFVSIVVVQWADLVICKTRRNSVFQQGMKNKILIFGLFEET

ALAAFLSYCPGMGVALRMYPLKPTWWFCAFPYSLLIFVYDEVRKLIIRRRPGGWVEKETY

Y

>sp|A2VDL6|AT1A2_BOVIN Sodium/potassium-transporting ATPase subunit alpha-2 OS=Bos taurus OX=9913 GN=ATP1A2 PE=1 SV=1

MGRGAGREYSPAATTAENGGGKKKQKEKELDELKKEVAMDDHKLSLDELGRKYQVDLSKG

LTNQRAQDILARDGPNALTPPPTTPEWVKFCRQLFGGFSILLWIGAILCFLAFGIQAAME

DEPSNDNLYLGVVLAAVVIVTGCFSYYQEAKSSKIMDSFKNMVPQQALVVREGEKMQINA

EEVVVGDLVEVKGGDRVPADLRIISSHGCKVDNSSLTGESEPQTRSPEFTHENPLETRNI

CFFSTNCVEGTARGIVIATGDRTVMGRIATLASGLEVGRTPIAMEIEHFIQLITGVAVFL

GVSFFVLSLILGYSWLEAVIFLIGIIVANVPEGLLATVTVCLTLTAKRMARKNCLVKNLE

AVETLGSTSTICSDKTGTLTQNRMTVAHMWFDNQIHEADTTEDQSGATFDKRSPTWTALS

RIAGLCNRAVFKAGQENISVSKRDTAGDASESALLKCIELSCGSVRKMRDRNPKVAEIPF

NSTNKYQLSIHEREDSPQSHVLVMKGAPERILDRCSSILVQGKEIPLDKEMQDAFQNAYL

ELGGLGERVLGFCQLNLPSAKFPRGFKFDTDELNFPTEKLCFVGLMSMIDPPRAAVPDAV

GKCRSAGIKVIMVTGDHPITAKAIAKGVGIISEGNETVEDIAARLNIPVSQVNPREAKAC

VVHGSDLKDMTSEQLDEILKNHTEIVFARTSPQQKLIIVEGCQRQGAIVAVTGDGVNDSP

ALKKADIGIAMGIAGSDVSKQAADMILLDDNFASIVTGVEEGRLIFDNLKKSIAYTLTSN

IPEITPFLLFIIANIPLPLGTVTILCIDLGTDMVPAISLAYEAAESDIMKRQPRNPQTDK

LVNERLISMAYGQIGMIQALGGFFTYFVILAENGFLPSRLLGIRLDWDDRSMNDLEDSYG

QEWTYEQRKVVEFTCHTAFFASIVVVQWADLIICKTRRNSVFQQGMKNKILIFGLLEETA

LAAFLSYCPGMGVALRMYPLKVTWWFCAFPYSLLIFIYDEVRKLILRRYPGGWVEKETYY

>sp|D3K0R6|AT2B4_BOVIN Plasma membrane calcium-transporting ATPase 4 OS=Bos taurus OX=9913 GN=ATP2B4 PE=1 SV=2

MTNPTEHTLPSNSILESREGEFGCTVMDLRKLMELRSSDAIDQINVHYGGVMNLCSRLKT

NPVEGLSGNPADLEKRKQVFGQNLIPPKKPKTFLELVWEALQDVTLIILEIAAIISLVLS

FYRPPGGENEQCGLAVTSPEDEGEAEAGWIEGAAILFSVIIVVLVTAFNDWSKEKQFRGL

QNRIEKEQKFSVIRNGHIIQLPVAEIVVGDIAQIKYGDLLPADGILIQGNDLKIDESSLT

GESDHVKKSLERDPMLLSGTHVMEGSGRMVVTAVGINSQTGIIFTLLGASEGEEEEKKKK

GKKQGVPENRNKAKTQDGVALEIQPLNSQEGIDSEEKEKKAAKLPKKEKSVLQGKLTRLA

VQIGKAGLIMSAITVLILILYFVIDNFVIQRRPWLAECTPIYVQYFVKFFIIGVTVLVVA

VPEGLPLAVTISLAYSVKKMMKDNNLVRHLDACETMGNATAICSDKTGTLTMNRMSVVQA

YIGDTRYHQIPSPDDLVPKVLDLIVNGISINSAYTSKILPPEKEGGLPRQVGNKTECALL

GFVSDLKQDYHAVRSEVPEEKLYKVYTFNSVRKSMSTVIEKPGGGYRMYSKGASEIILRK

CNRILDKKGEAVPFKNKDRDEMVRTVIEPMACEGLRTLCIAYRDFNDGEPPWDNESEILT

ELTCIAVVGIEDPVRPEVPEAIAKCKRAGITVRMVTGDNINTARAIATKCGIVTPGDDFL

CLEGKEFNRLIRNEKGEVEQEKLDKIWPKLRVLARSSPTDKHTLVKGIIDSTVGDQRQVV

AVTGDGTNDGPALKKADVGFAMGIAGTDVAKEASDIILTDDNFTSIVKAVMWGRNVYDSI

SKFLQFQLTVNVVAVIVAFTGACITQDSPLKAVQMLWVNLIMDTFASLALATEPPTDSLL

KRRPYGRNKPLISRTMMKNILGHAVYQLTVIFFLVFAGEKFFDIDSGRRAPLHSPPSQHY

TIIFNTFVLMQLFNEINSRKIHGERNVFSGIFRNLIFCSVVLGTFISQIIIVEFGGKPFS

CTKLTLSQWFWCLFIGIGELLWGQVISTIPTQSLKFLKEAGHGTTKEEITKDAEGLDEID

HAEMELRRGQILWFRGLNRIQTQIKVVKAFHSSLHESIQKPKNQNSIHNFMTHPEFTIDE

EGPRTPLLDEQEEEIFEKVSKPGTKTSSLDGEVTPQTNKNNNTVDCCQVQIVASHSDSPL

HSLETSV

>sp|Q3T014|PMGE_BOVIN Bisphosphoglycerate mutase OS=Bos taurus OX=9913 GN=BPGM PE=2 SV=3

MSKYKLIMLRHGEGAWNKENRFCSWVDQKLNSDGLQEARNCGKQLKALNFEFDLVFTSIL

NRSIHTAWLILEELGQEWVPVESSWRLNERHYGALISLNREQMALNHGEEQVRLWRRSYN

VTPPPIEESHPYYHEIYNDRKYKVCDVPLDQLPRSESLKDVLERLLPYWNERIAPEVLRG

KTVLISAQGNSCRALLKYLEGISDEEIINITLPTGVPILLELDENLRTVGPHQFLGDQEA

IQAAIKKVDDQGKVKRADK

>sp|Q28065|C4BPA_BOVIN C4b-binding protein alpha chain OS=Bos taurus OX=9913 GN=C4BPA PE=2 SV=1

MKHQRVPVMILHSKGTMASWPFSRLWSISDPILFQVTLVATLLATVLGSCGIPPYLDFAF

PINELNETRFETGTTLRYTCRPGYRISSRKNFLICDGTDNWKYKEFCVKKRCENPGELLN

GQVIVKTDYSFGSEIEFSCSEGYVLIGSANSYCQLQDKGVVWSDPLPQCIIAKCEPPPTI

SNGRHNGGDEDFYTYGSSVTYSCDRDFSMLGKASISCRVENKTIGVWSPSPPSCKKVICV

QPVVKDGKITSGFGPIYTYQQSIVYACNKGFRLEGDSLIHCEADNSWNPPPPTCELNGCL

GLPHIPHALWERYDHQTQTEQQVYDIGFVLSYKCHFGYKPETDGPTTVTCQSNLEWSPYI

ECKEVCCPEPNLNNYGSITLHRRPSTSTHCTYISGDKISYECHSKYMFDALCTKHGTWSP

RTPECRPDCKSPPVIAHGQHKVVSKFFTFDHQAVYECDKGYILVGAKELSCTSSGWSPAV

PQCKALCLKPEIEYGRLSVEKVRYVEPEIITIQCESGYSVVGSENITCSEDRTWYPEVPK

CEWEYPEGCEQVVTGRKLLQCLSRPEEVKLALEVYKLSLEIEILQTNKLKKEAFLLRERE

KNVTCDFNPE

>sp|A7MBJ5|CAND1_BOVIN Cullin-associated NEDD8-dissociated protein 1 OS=Bos taurus OX=9913 GN=CAND1 PE=2 SV=1

MASASYHISNLLEKMTSSDKDFRFMATNDLMTELQKDSIKLDDDSERKVVKMILKLLEDK

NGEVQNLAVKCLGPLVSKVKEYQVETIVDTLCTNMLSDKEQLRDISSIGLKTVIGELPPA

SSGSALAANVCKKITGRLTSAIAKQEDVSVQLEALDIMADMLSRQGGLLVNFHPSILTCL

LPQLTSPRLAVRKRTIIALGHLVMSCGNIVFVDLIEHLLSELSKNDSMSTTRTYIQCIAA

ISRQAGHRIGEYLEKIIPLVVKFCNVDDDELREYCIQAFESFVRRCPKEVYPHVSTIINI

CLKYLTYDPNYNYDDEDEDENAMDADGGDDDDQGSDDEYSDDDDMSWKVRRAAAKCLDAV

VSTRHEMLPEFYKTVSPALISRFKEREENVKADVFHAYLSLLKQTRPVQSWLCDPDAMEQ

GETPLTMLQSQVPNIVKALHKQMKEKSVKTRQCCFNMLTELVNVLPGALTQHIPVLVPGI

IFSLNDKSSSSNLKIDALSCLYVILCNHSPQVFHPHVQALVPPVVACVGDPFYKITSEAL

LVTQQLVKVIRPLDQPSSFDATPYIKDLFTCTIKRLKAADIDQEVKERAISCMGQIICNL

GDNLGSDLPNTLQIFLERLKNEITRLTTVKALTLIAGSPLKIDLRPVLGEGVPILASFLR

KNQRALKLGTLSALDILIKNYSDSLTAAMIDAVLDELPPLISESDMHVSQMAISFLTTLA

KVYPSSLSKISGSILNELIGLVRSPLLQGGALSAMLDFFQALVVTGTNNLGYMDLLRMLT

GPVYSQSTALTHKQSYYSIAKCVAALTRACPKEGPAVVGQFIQDVKNSRSTDSIRLLALL

SLGEVGHHIDLSGQLELKSVILEAFSSPSEEVKSAASYALGSISVGNLPEYLPFVLQEIT

SQPKRQYLLLHSLKEIISSASVVGLKPYVENIWALLLKHCECAEEGTRNVVAECLGKLTL

IDPETLLPRLKGYLISGSSYARSSVVTAVKFTISDHPQPIDPLLKNCIGDFLKTLEDPDL

NVRRVALVTFNSAAHNKPSLIRDLLDTVLPHLYNETKVRKELIREVEMGPFKHTVDDGLD

IRKAAFECMYTLLDSCLDRLDIFEFLNHVEDGLKDHYDIKMLTFLMLVRLSTLCPSAVLQ

RLDRLVEPLRATCTTKVKANSVKQEFEKQDELKRSAMRAVAALLTIPEAEKSPLMSEFQS

QISSNPELAAIFESIQKDSSSTNLESMDTS

>sp|P00432|CATA_BOVIN Catalase OS=Bos taurus OX=9913 GN=CAT PE=1 SV=3

MADNRDPASDQMKHWKEQRAAQKPDVLTTGGGNPVGDKLNSLTVGPRGPLLVQDVVFTDE

MAHFDRERIPERVVHAKGAGAFGYFEVTHDITRYSKAKVFEHIGKRTPIAVRFSTVAGES

GSADTVRDPRGFAVKFYTEDGNWDLVGNNTPIFFIRDALLFPSFIHSQKRNPQTHLKDPD

MVWDFWSLRPESLHQVSFLFSDRGIPDGHRHMNGYGSHTFKLVNANGEAVYCKFHYKTDQ

GIKNLSVEDAARLAHEDPDYGLRDLFNAIATGNYPSWTLYIQVMTFSEAEIFPFNPFDLT

KVWPHGDYPLIPVGKLVLNRNPVNYFAEVEQLAFDPSNMPPGIEPSPDKMLQGRLFAYPD

THRHRLGPNYLQIPVNCPYRARVANYQRDGPMCMMDNQGGAPNYYPNSFSAPEHQPSALE

HRTHFSGDVQRFNSANDDNVTQVRTFYLKVLNEEQRKRLCENIAGHLKDAQLFIQKKAVK

NFSDVHPEYGSRIQALLDKYNEEKPKNAVHTYVQHGSHLSAREKANL

>sp|Q3ZBH0|TCPB_BOVIN T-complex protein 1 subunit beta OS=Bos taurus OX=9913 GN=CCT2 PE=1 SV=3

MASLSLAPVNIFKAGADEERAETARLSSFIGAIAIGDLVKSTLGPKGMDKILLSSGRDAS

LMVTNDGATILKNIGVDNPAAKVLVDMSRVQDDEVGDGTTSVTVLAAELLREAESLIAKK

IHPQTIIAGWREATKAARQALLNSAVDHGSDEVKFRQDLMNIAGTTLSSKLLTHHKDHFT

KLAVEAVLRLKGSGNLEAIHVIKKLGGSLADSYLDEGFLLDKKIGVNQPKRIENAKILIA

NTGMDTDKIKIFGSRVRVDSTAKVAEIEHAEKEKMKEKVERILKHGINCFINRQLIYNYP

EQLFGAAGVMAIEHADFVGVERLALVTGGEIASTFDHPELVKLGSCKLIEEVMIGEDKLI

HFSGVALGEACTIVLRGATQQILDEAERSLHDALCVLAQTVKDSRTVYGGGCSEMLMAHA

VTQLASRTPGKEAVAMESYAKALRMLPTIIADNAGYDSADLVAQLRAAHSEGKTTAGLDM

KEGTIGDMSVLGITESFQVKRQVLLSAAEAAEVILRVDNIIKAAPRKRVPDHHPC

>sp|Q95122|CD14_BOVIN Monocyte differentiation antigen CD14 OS=Bos taurus OX=9913 GN=CD14 PE=2 SV=2

MVCVPYLLLLLLPSLLRVSADTTEPCELDDDDFRCVCNFTDPKPDWSSAVQCMVAVEVEI

SAGGRSLEQFLKGADTNPKQYADTIKALRVRRLKLGAAQVPAQLLVAVLRALGYSRLKEL

TLEDLEVTGPTPPTPLEAAGPALTTLSLRNVSWTTGGAWLGELQQWLKPGLRVLNIAQAH

SLAFPCAGLSTFEALTTLDLSDNPSLGDSGLMAALCPNKFPALQYLALRNAGMETPSGVC

AALAAARVQPQSLDLSHNSLRVTAPGATRCVWPSALRSLNLSFAGLEQVPKGLPPKLSVL

DLSCNKLSREPRRDELPEVNDLTLDGNPFLDPGALQHQNDPMISGVVPACARSALTMGVS

GALALLQGARGFA

>sp|P30932|CD9_BOVIN CD9 antigen OS=Bos taurus OX=9913 GN=CD9 PE=2 SV=2

MPVKGGTKCIKYLLFGFNFIFWLAGIAVLSVGLWLRFDSQTKSIFEQENNDSSFYTGVYI

LIGAGALMMLVGFLGCCGAVQESQCMLGLFFSFLLVIFAIEVAAAIWGYSHKEEVIKEVQ

KFYEDTYNKLKNKDEPQRETLKAIHIALDCCGLTGVPEQFLTDTCPPKNLIDSLKTRPCP

EAIDEIFRSKFHIIGAVGIGIAVVMIFGMVFSMILCCAIRRNRDMV

>sp|Q2KJ93|CDC42_BOVIN Cell division control protein 42 homolog OS=Bos taurus OX=9913 GN=CDC42 PE=1 SV=1

MQTIKCVVVGDGAVGKTCLLISYTTNKFPSEYVPTVFDNYAVTVMIGGEPYTLGLFDTAG

QEDYDRLRPLSYPQTDVFLVCFSVVSPSSFENVKEKWVPEITHHCPKTPFLLVGTQIDLR

DDPSTIEKLAKNKQKPITPETAEKLARDLKAVKYVECSALTQKGLKNVFDEAILAALEPP

EPKKSRRCVLL

>sp|Q28085|CFAH_BOVIN Complement factor H OS=Bos taurus OX=9913 GN=CFH PE=1 SV=3

MRFPAKIVWLVLWTVCVAEDCKEPPPRKETEILSGSWTEQTYQEGTQATYKCRPGYRTLG

SIVMMCRGGKWVSLHPSRICRKKPCAHPGDTPFGSFHLAEGTQFEYGAKVVYTCDEGYQM

VGEMNFRECDTNGWTNDIPICEVVKCLPVTEPENGKIFSDALEPDQEYTYGQVVQFECNS

GYMLDGPKQIHCSAGGVWSAETPKCVEIFCKTPVILNGQAVLPKATYKANERVQYRCAAG

FEYGQRGDTICTKSGWTPAPTCIEITCDPPRIPNGVYRPELSKYRGQDKITYECKKGFFP

EIRGTDATCTRDGWVPVPRCAWKPCSYPVIKHGRLYYSYRGYFPARVNQQFVYSCDHHFV

PPSQRSWDHLTCTAEGWSPEEPCLRQCIFNYLENGHTPYREEKYLQGETVRVRCYEGYSL

QNDQNTMTCTESGWSPPPRCIRVKTCSKSNIRIENGFLSESTFTYPLNKQTEYKCKPGYV

TADGKTSGLITCLKNGWSAQPVCIKSCDRPVFEKARVKSDGTWFRLNDRLDYECVDGYEN

RDGRTTGSIVCGQDGWSDKAACYERECSIPEMDPYLNAYPRKETYKVGDVLKFSCSQGRI

MVGADSVQCYHFGWSPKLPTCKVKKVKSCALPPELPNGKRKEIHKEEYAHNEVVEYACNP

RFLMKGSHKIQCVDGEWTALPVCIEEERTCGNIPDLDHGDVKPSVPPYHHGDSVEFSCRE

AFTMIGPRFITCISGEWTQPPQCIATDELRKCKGSTLFPPEGRQAHKIEYDHNTNKSYQC

RGKSEHKHSICINGEWDPKVDCNEEAKIQLCPPPPQVPNACDMTTTVNYQDGEKISILCK

ENYLIQDAEEIVCKDGRWQSIPRCIEKIGCSQPPQIDHGTISSSSSAEERREIHEQRLYA

HGTKLSYTCEEGFEISENNVIICHMGKWSSPPQCVGLPCGLPPYVQNGVVSHKKDRYQYG

EEVTYDCDEGFGTDGPASIRCLGGEWSRPQDCISTNCVNLPTFEDAVLTDREKDFYRSGE

QVAFKCLSYYQLDGSNTIQCIKSKWIGRPACRDVSCGNPPQVENAIIHNQKSKYQSEERA

RYECIGNYDLFGEMEVVCLNGTWTEPPQCKDSQGKCGPPPPIDNGDITSLLQSVYPPGMI

VEYRCQAYYELRGNKNVVCRNGEWSQLPKCLEACVISEETMRKHHIQLRWKHDKKIYSKT

EDTIEFMCQHGYRQLTPKHTFRATCREGKVVYPRCG

>sp|Q5EA61|KCRB_BOVIN Creatine kinase B-type OS=Bos taurus OX=9913 GN=CKB PE=1 SV=1

MPFSNSHNTLKLRFPAEDEFPDLSGHNNHMAKVLTPELYAELRAKSTPSGFTVDDVIQTG

VDNPGHPYIMTVGCVAGDEESYDVFKELFDPIIEDRHGGYKPTDEHKTDLNPDNLQGGDD

LDPNYVLSSRVRTGRSIRGFCLPPHCSRGERRAIEKLAVEALSSLDGDLAGRYYALKSMT

EAEQQQLIDDHFLFDKPVSPLLLASGMARDWPDARGIWHNDNKTFLVWINEEDHLRVISM

QKGGNMKEVFTRFCNGLTQIETLFKSKNYEFMWNPHLGYILTCPSNLGTGLRAGVHIKLP

HLGKHEKFPEVLKRLRLQKRGTGGVDTAAVGGVFDVSNADRLGFSEVELVQMVVDGVKLL

IEMEQRLEQGQAIDDLMPAQK

>sp|P42916|CL43_BOVIN Collectin-43 OS=Bos taurus OX=9913 GN=CL43 PE=1 SV=2

MLPLPLSILLLLTQSQSFLGEEMDVYSEKTLTDPCTLVVCAPPADSLRGHDGRDGKEGPQ

GEKGDPGPPGMPGPAGREGPSGRQGSMGPPGTPGPKGEPGPEGGVGAPGMPGSPGPAGLK

GERGTPGPGGAIGPQGPSGAMGPPGLKGDRGDPGEKGARGETSVLEVDTLRQRMRNLEGE

VQRLQNIVTQYRKAVLFPDGQAVGEKIFKTAGAVKSYSDAEQLCREAKGQLASPRSSAEN

EAVTQLVRAKNKHAYLSMNDISKEGKFTYPTGGSLDYSNWAPGEPNNRAKDEGPENCLEI

YSDGNWNDIECREERLVICEF

>sp|Q2KIS7|TETN_BOVIN Tetranectin OS=Bos taurus OX=9913 GN=CLEC3B PE=2 SV=1

MELWGPCVLLCLFSLLTQVTAETPTPKAKKAANAKKDAVSPKMLEELKTQLDSLAQEVAL

LKEQQALQTVCLKGTKVHMKCFLAFVQAKTFHEASEDCISRGGTLGTPQTGSENDALYEY

LRQSVGSEAEVWLGFNDMASEGSWVDMTGGHIAYKNWETEITAQPDGGKVENCATLSGAA

NGKWFDKRCRDKLPYVCQFAIV

>sp|Q9XSA7|CLIC4_BOVIN Chloride intracellular channel protein 4 OS=Bos taurus OX=9913 GN=CLIC4 PE=2 SV=3

MALSMPLNGLKEEDKEPIIELFVKAGSDGESIGNCPFSQRLFMILWLKGVVFSVTTVDLK

RKPADLQNLAPGTHPPFITFNNEVKTDVNKIEEFLEEVLCPPKYLKLSPKHPESNTAGMD

IFAKFSAYIKNSRPEANEALERGLLKTLQKLDEYLNSPLPDEIDENSMEDIKFSTRKFLD

GNEMTLADCNLLPKLHIVKVVAKKYRNFDIPKGMTGIWRYLTNAYSRDEFTNTCPSDKEV

EIAYSDVAKRLTK

>sp|Q3ZC84|CNDP2_BOVIN Cytosolic non-specific dipeptidase OS=Bos taurus OX=9913 GN=CNDP2 PE=2 SV=1

MSALTTLFKYVDENQDRYVKKLAEWVAIQSVSAWPEKRGEIRRMMEVAAADIKQLGGSVQ

LVDIGTQKLPDGSEIPLPPILLGKLGSDPQKKTVCIYGHLDVQPAALEDGWDSEPFTLVE

RDGKLFGRGATDDKGPVAGWINALEAFQKTKQEVPVNVRFCLEGMEESGSEGLDALIFAQ

KDAFFKDVDYVCISDNYWLGKNKPCITYGLRGICYFFIEVECSDKDLHSGVYGGSVHEAM

TDLIMLMGCLMDKKGKILIPGISEAVAPVTEEELELYDKIDFDLEEYARDVGAGTLLHGC

KKDILMHRWRYPSLSLHGIEGAFSGSGAKTVIPRKVVGKFSIRLVPNMTPEVVSEQVTSY

LTKKFAELHSPNKFKVYMGHGGKPWVSDFNHPHYLAGRRALKTVFGVEPDLTREGGSIPV

TLTFQEATGKNVMLLPVGSADDGAHSQNEKLNRRNYIEGTKMLAAYLYEVSQLKD

>sp|P02453|CO1A1_BOVIN Collagen alpha-1(I) chain OS=Bos taurus OX=9913 GN=COL1A1 PE=1 SV=3

MFSFVDLRLLLLLAATALLTHGQEEGQEEGQEEDIPPVTCVQNGLRYHDRDVWKPVPCQI

CVCDNGNVLCDDVICDELKDCPNAKVPTDECCPVCPEGQESPTDQETTGVEGPKGDTGPR

GPRGPAGPPGRDGIPGQPGLPGPPGPPGPPGPPGLGGNFAPQLSYGYDEKSTGISVPGPM

GPSGPRGLPGPPGAPGPQGFQGPPGEPGEPGASGPMGPRGPPGPPGKNGDDGEAGKPGRP

GERGPPGPQGARGLPGTAGLPGMKGHRGFSGLDGAKGDAGPAGPKGEPGSPGENGAPGQM

GPRGLPGERGRPGAPGPAGARGNDGATGAAGPPGPTGPAGPPGFPGAVGAKGEGGPQGPR

GSEGPQGVRGEPGPPGPAGAAGPAGNPGADGQPGAKGANGAPGIAGAPGFPGARGPSGPQ

GPSGPPGPKGNSGEPGAPGSKGDTGAKGEPGPTGIQGPPGPAGEEGKRGARGEPGPAGLP

GPPGERGGPGSRGFPGADGVAGPKGPAGERGAPGPAGPKGSPGEAGRPGEAGLPGAKGLT

GSPGSPGPDGKTGPPGPAGQDGRPGPPGPPGARGQAGVMGFPGPKGAAGEPGKAGERGVP

GPPGAVGPAGKDGEAGAQGPPGPAGPAGERGEQGPAGSPGFQGLPGPAGPPGEAGKPGEQ

GVPGDLGAPGPSGARGERGFPGERGVQGPPGPAGPRGANGAPGNDGAKGDAGAPGAPGSQ

GAPGLQGMPGERGAAGLPGPKGDRGDAGPKGADGAPGKDGVRGLTGPIGPPGPAGAPGDK

GEAGPSGPAGPTGARGAPGDRGEPGPPGPAGFAGPPGADGQPGAKGEPGDAGAKGDAGPP

GPAGPAGPPGPIGNVGAPGPKGARGSAGPPGATGFPGAAGRVGPPGPSGNAGPPGPPGPA

GKEGSKGPRGETGPAGRPGEVGPPGPPGPAGEKGAPGADGPAGAPGTPGPQGIAGQRGVV

GLPGQRGERGFPGLPGPSGEPGKQGPSGASGERGPPGPMGPPGLAGPPGESGREGAPGAE

GSPGRDGSPGAKGDRGETGPAGPPGAPGAPGAPGPVGPAGKSGDRGETGPAGPAGPIGPV

GARGPAGPQGPRGDKGETGEQGDRGIKGHRGFSGLQGPPGPPGSPGEQGPSGASGPAGPR

GPPGSAGSPGKDGLNGLPGPIGPPGPRGRTGDAGPAGPPGPPGPPGPPGPPSGGYDLSFL

PQPPQEKAHDGGRYYRADDANVVRDRDLEVDTTLKSLSQQIENIRSPEGSRKNPARTCRD

LKMCHSDWKSGEYWIDPNQGCNLDAIKVFCNMETGETCVYPTQPSVAQKNWYISKNPKEK

RHVWYGESMTGGFQFEYGGQGSDPADVAIQLTFLRLMSTEASQNITYHCKNSVAYMDQQT

GNLKKALLLQGSNEIEIRAEGNSRFTYSVTYDGCTSHTGAWGKTVIEYKTTKTSRLPIID

VAPLDVGAPDQEFGFDVGPACFL

>sp|P02465|CO1A2_BOVIN Collagen alpha-2(I) chain OS=Bos taurus OX=9913 GN=COL1A2 PE=1 SV=2

MLSFVDTRTLLLLAVTSCLATCQSLQEATARKGPSGDRGPRGERGPPGPPGRDGDDGIPG

PPGPPGPPGPPGLGGNFAAQFDAKGGGPGPMGLMGPRGPPGASGAPGPQGFQGPPGEPGE

PGQTGPAGARGPPGPPGKAGEDGHPGKPGRPGERGVVGPQGARGFPGTPGLPGFKGIRGH

NGLDGLKGQPGAPGVKGEPGAPGENGTPGQTGARGLPGERGRVGAPGPAGARGSDGSVGP

VGPAGPIGSAGPPGFPGAPGPKGELGPVGNPGPAGPAGPRGEVGLPGLSGPVGPPGNPGA

NGLPGAKGAAGLPGVAGAPGLPGPRGIPGPVGAAGATGARGLVGEPGPAGSKGESGNKGE

PGAVGQPGPPGPSGEEGKRGSTGEIGPAGPPGPPGLRGNPGSRGLPGADGRAGVMGPAGS

RGATGPAGVRGPNGDSGRPGEPGLMGPRGFPGSPGNIGPAGKEGPVGLPGIDGRPGPIGP

AGARGEPGNIGFPGPKGPSGDPGKAGEKGHAGLAGARGAPGPDGNNGAQGPPGLQGVQGG

KGEQGPAGPPGFQGLPGPAGTAGEAGKPGERGIPGEFGLPGPAGARGERGPPGESGAAGP

TGPIGSRGPSGPPGPDGNKGEPGVVGAPGTAGPSGPSGLPGERGAAGIPGGKGEKGETGL

RGDIGSPGRDGARGAPGAIGAPGPAGANGDRGEAGPAGPAGPAGPRGSPGERGEVGPAGP

NGFAGPAGAAGQPGAKGERGTKGPKGENGPVGPTGPVGAAGPSGPNGPPGPAGSRGDGGP

PGATGFPGAAGRTGPPGPSGISGPPGPPGPAGKEGLRGPRGDQGPVGRSGETGASGPPGF

VGEKGPSGEPGTAGPPGTPGPQGLLGAPGFLGLPGSRGERGLPGVAGSVGEPGPLGIAGP

PGARGPPGNVGNPGVNGAPGEAGRDGNPGNDGPPGRDGQPGHKGERGYPGNAGPVGAAGA

PGPQGPVGPVGKHGNRGEPGPAGAVGPAGAVGPRGPSGPQGIRGDKGEPGDKGPRGLPGL

KGHNGLQGLPGLAGHHGDQGAPGAVGPAGPRGPAGPSGPAGKDGRIGQPGAVGPAGIRGS

QGSQGPAGPPGPPGPPGPPGPSGGGYEFGFDGDFYRADQPRSPTSLRPKDYEVDATLKSL

NNQIETLLTPEGSRKNPARTCRDLRLSHPEWSSGYYWIDPNQGCTMDAIKVYCDFSTGET

CIRAQPEDIPVKNWYRNSKAKKHVWVGETINGGTQFEYNVEGVTTKEMATQLAFMRLLAN

HASQNITYHCKNSIAYMDEETGNLKKAVILQGSNDVELVAEGNSRFTYTVLVDGCSKKTN

EWQKTIIEYKTNKPSRLPILDIAPLDIGGADQEIRLNIGPVCFK

>sp|Q0V8F1|CORO7_BOVIN Coronin-7 OS=Bos taurus OX=9913 GN=CORO7 PE=2 SV=1

MNRFKVSKFRHTEARQPRREAWIGDIRAGTAPSCGNHIKASCSLIAFNSDHPGVLGIVPL

ESQGEDKRQVTHLGCHSDLVTDLDFSPFDDFLLATASADRTVKLWRLPLSGQALPSGPGL

LLGPEDAQVEVLQFHPTADGVLLSAAGRAVKVWDATKQQPLTELATHGDLVQGAAWSRDG

ALLGTTCKDKQLRIFDPRAKPEAAQSTPAHENSRDGRLVWTGTQEYLVSTGFNQMREREV

KLWDTRLFSAALTSLTLDTSPRSLVPLLDPDSGLLVLAGKGENQLYCYEAAPQQPALSPV

TQCLLESVLRGAALVPRRALAVMGCEVLRVLQLSDTAIVPISYHVPRKTVEFHEDLFPDT

AGCVPASDPHAWWAGSDQQVQRVSLHPARRAHPSFTSCLAPPAELTPATAQPAGTPEGFS

STPSSLTSPSTPSSLGPSLTSTSGIGTSPSQRSLQSLLGPSSKFRHAQGSVLHRDSHITN

LKGLNLTTPGESDGFCANQLRVAVPLLSSGGQVAVLELRKPGRLPDTALPTLQNGVAVTD

LAWDPFDPHRLAVAGEDARIRLWRVPPDGLQEVLTMPEAVLTGHTEKIYSLRFHPLAADV

LASSSYDLTVRIWDLKVGAEQLRLQGHRDQIFGLAWSPDGQQLATVCKDGRLRIYEPRGS

PEPLQEGPGPEGARGARVVWVCDGHYLLVSGFDSRSERQLLLYSAKALAGGPSAVLGLDV

APSTLLPSYDPDTGLVLLTGKGDTRVFLYELLPGAPFFLECNSFTSPDPHKGFILLPKTE

CDVREVEFARCLRLRQTSLEPVAFRLPRVRKEFFQDDVFPDTTVSWEPALSAEAWLGGAN

GTPRLLSLQPPGMTPVSQAPREAPARRAPSSVYLEEKSDQQKKEELLSAMVAKLGNREDP

LPQDSFEGVDEDEWD

>sp|A5D785|XPO2_BOVIN Exportin-2 OS=Bos taurus OX=9913 GN=CSE1L PE=2 SV=1

MELSDANLQTLTEYLKKTLDPDPAIRRPAEKFLESVEGNQNYPLLLLTLLEKSQDNVIKV

CASVTFKNYIKRNWRIVEDEPNKICEADRVAIKANIVHLMLSSPEQIQKQLSDAISIIGR

EDFPQKWPDLLTEMVNRFQSGDFHVINGVLRTAHSLFKRYRHEFKSNELWTEIKLVLDAF

ALPLTNLFKATIELCSTHANDASALRILFSSLILISKLFYSLNFQDLPEFFEDNMETWMN

NFHTLLTLDNKLLQTDDEEEAGLLELLKSQICDNAALYAQKYDEEFQRYLPRFVTAIWNL

LVTTGQEVKYDLLVSNAIQFLASVCERPHYKNLFEDQNTLTSICEKVIVPNMEFRAADEE

AFEDNSEEYIRRDLEGSDIDTRRRAACDLVRGLCKFFEGPVTGIFSGYVNSMLQEYAKNP

SVNWKHKDAAIYLVTSLASKAQTQKHGITQANELVNLTEFFVNHILPDLKSANVNEFPVL

KADGIKYITIFRNQVPKEHLLVSIPLLINHLQAESIVVHTYAAHALERLFTMRGPNSATL

FTAAEIAPFVEILLTNLFKALTLPGSSENEYIMKAIMRSFSLLQEAIIPYIPTLITQLTQ

KLLAVSKNPSKPHFNHYMFEAICLSIRITCKSNPAAVVNFEEALFLVFTEILQNDVQEFI

PYVFQVMSLLLETHKNDIPSSYMALFPHLLQPVLWERTGNIPALVRLLQAFLERGSNTIA

SAAADKIPGLLGVFQKLIASKANDHQGFYLLNSIIEHMPPESVDQYRKQIFILLFQRLQN

SKTTKFIKSFLVFINLYCIKYGALALQEIFDGIQPKMFGMVLEKIIIPEIQKVSGNVEKK

ICAVGITKLLTECPPMMDTEYTKLWTPLLQSLIGLFELPEDDTIPDEEHFIDIEDTPGYQ

TAFSQLAFAGKKEHDPVGQMVNNPKIHLAQSLHKLSTACPGRVPSMVSTSLNAEALQYLQ

GYLQAASVTLL

>sp|Q5E998|CATL2_BOVIN Cathepsin L2 OS=Bos taurus OX=9913 GN=CTSV PE=2 SV=1

MNPSFFLTVLCLGVASAAPKLDPNLDAHWHQWKATHRRLYGMNEEEWRRAVWEKNKKIID

LHNQEYSEGKHGFRMAMNAFGDMTNEEFRQVMNGFQNQKHKKGKLFHEPLLVDVPKSVDW

TKKGYVTPVKNQGQCGSCWAFSATGALEGQMFRKTGKLVSLSEQNLVDCSRAQGNQGCNG

GLMDNAFQYIKDNGCLDSEESYPYLATDTNSCNYKPECSAANDTGFVDIPQREKALMKAV

ATVGPISVAIDAGHTSFQFYKSGIYYDPDCSSKDLDHGVLVVGYGFEGTDSNNNKFWIVK

NSWGPEWGWNGYVKMAKDQNNHCGIATAASYPTV

>sp|Q28007|DPYD_BOVIN Dihydropyrimidine dehydrogenase [NADP(+)] OS=Bos taurus OX=9913 GN=DPYD PE=1 SV=1

MAPVLSKDVADIESILALNPRTQSRATLRSTLAKKLDKKHWKRNPDKNCFNCEKLENNFD

DIKHTTLGERGALREAMRCLKCADAPCQKSCPTNLDIKSFITSISNKNYYGAAKMIFSDN

PLGLTCGMVCPTSDLCVGGCNLYATEEGPINIGGLQQYATEVFKAMNIPQIRNPSLPPPE

KMPEAYSAKIALLGAGPASISCASFLARLGYNDITIFEKQEYVGGISTSEIPQFRLPYDV

VNFEIELMKDLGVKIICGKSLSVNDITLSTLKEEGYKAAFIGIGLPEPKKDHIFQGLTQD

QGFYTSKDFLPLVAKSSKAGMCACHSPLLSIRGTVIVLGAGDTAFDCATSALRCGARRVF

IVFRKGFVNIRAVPEEVELAREEKCEFLPFLSPRKVIVKGGRIVAMQFVRTEQDETGKWN

EDGDQIACLKADVVISAFGSVLSDPKVKEALSPIKFNRWDLPEVDPETMQTSEPWVFAGG

DVVGIANTTVEAVNDGKQASWYIHRYIQSQYGASVSAKPELPLFYTPIDLVDISVEMAAL

KFTNPFGLASATPTTSSSMIRRAFEAGWAFALTKTFSLDKDIVTNVSPRIIRGTTSGPMY

GPGQSSFLNIELISEKTAAYWCQSVTELKADFPDNIVIASIMCSYNRNDWMELSRKAEAS

GADALELNLSCPHGMGERGMGLACGQDPELVRNICRWVRQAVRIPFFAKLTPNVTDIVSI

ARAAKEGGANGVTATNTVSGLMGLKADGTPWPAVGREKRTTYGGVSGTAIRPIALRAVTT

IARALPEFPILATGGIDSAESGLQFLHGGASVLQVCSAIQNQDFTIIQDYCTGLKALLYL

KSIEELQDWDGQSPATKSHQKGKPVPCIAELVGKKLPSFGPYLEKCKKIIAEEKLRLKKE

NVTVLPLERNHFIPKKPIPSVKDVIGKALQYLGTYGELNNTEQVVAVIDEEMCINCGKCY

MTCNDSGYQAIQFDPETHLPTVTDTCTGCTLCLSVCPIIDCIKMVSRTTPYEPKRGLPLA

VNPVS

>sp|Q3SZ65|IF4A2_BOVIN Eukaryotic initiation factor 4A-II OS=Bos taurus OX=9913 GN=EIF4A2 PE=2 SV=1

MSGGSADYNREHGGPEGMDPDGVIESNWNEIVDNFDDMNLKESLLRGIYAYGFEKPSAIQ

QRAIIPCIKGYDVIAQAQSGTGKTATFAISILQQLEIEFKETQALVLAPTRELAQQIQKV

ILALGDYMGATCHACIGGTNVRNEMQKLQAEAPHIVVGTPGRVFDMLNRRYLSPKWIKMF

VLDEADEMLSRGFKDQIYEIFQKLNTSIQVVLLSATMPTDVLEVTKKFMRDPIRILVKKE

ELTLEGIKQFYINVEREEWKLDTLCDLYETLTITQAVIFLNTRRKVDWLTEKMHARDFTV

SALHGDMDQKERDVIMREFRSGSSRVLITTDLLARGIDVQQVSLVINYDLPTNRENYIHR

IGRGGRFGRKGVAINFVTEEDKRILRDIETFYNTTVEEMPMNVADLI

>sp|P31976|EZRI_BOVIN Ezrin OS=Bos taurus OX=9913 GN=EZR PE=1 SV=2

MPKPINVRVTTMDAELEFAIQPNTTGKQLFDQVVKTIGLREVWYFGLQYVDNKGFPTWLK

LDKKVSAQEVRKESPLQFKFRAKFYPEDVAEELIQDITQKLFFLQVKEGILSDEIYCPPE

TAVLLGSYAVQAKFGDYNKELHKAGYLGSERLIPQRVMDQHKLTRDQWEDRIQVWHAEHR

GMLKDSAMLEYLKIAQDLEMYGINYFEIKNKKGTDLWLGVDALGLNIYEKDDKLTPKIGF

PWSEIRNISFNDKKFVIKPIDKKAPDFVFYAPRLRINKRILQLCMGNHELYMRRRKPDTI

EVQQMKAQAREEKHQKQLERQQLETEKKRRETVEREKEQMMREKEELMLRLQDYEEKTRK

AEKELSDQIQRALKLEEERKRAQEEAGRLEADRLAALRAKEELERQAADQIKSQEQLATE

LAEYTAKIALLEEARRRKENEVEEWQLRAKEAQDDLVKTREELHLVMTAPPPPPVYEPVN

YHVHEGPQEEGTELSAELSSEGILDDRNEEKRITEAEKNERVQRQLMTLTSELSQARDEN

KRTHNDIIHNENMRQGRDKYKTLRQIRQGNTKQRIDEFEAM

>sp|P12260|F13A_BOVIN Coagulation factor XIII A chain (Fragment) OS=Bos taurus OX=9913 GN=F13A1 PE=1 SV=2

MSESSGTAFGGRRAIPPNTSNAAENDPPTVELQGLVPRGFNPQDYLNVTNVHLFKERWDS

NKVDHHTDKYSNDKLIVRRGQSFYIQIDFNRPYDPTRDLFRVEYVIGLYPQENKGTYIPV

PLVSELQSGKWGAKVVMREDRSVRLSVQSSADCIVGKFRMYVAVWTPYGVIRTSRNPETD

TYILFNPWCEEDAVYLEN

>sp|Q71SP7|FAS_BOVIN Fatty acid synthase OS=Bos taurus OX=9913 GN=FASN PE=1 SV=1

MEEVVITGMSGKLPESENLEEFWANLIGGVDMVTDDDRRWKAGLYGLPRRSGKLKDLSRF

DASFFGVHPKQAHNMDPQLRLLLEVTYEAIVDAGINPASIRGTNTGVWVGVSGSEASEAL

SRDPETLVGYSMVGCQRAMLANRLSFFFDFKGPSITLDTACSSSLLALQRAYQAIQRGEC

AMAIVGGVNIRLKPNTSVQFMKLGMLSPEGTCKFFDASGNGYCRAKAVMAILLTKKSLAR

RVYATILNAGTNTDGCKEKGVTFPSGEAQEQLISSLYKPAGLDPETLEYVEAHGTGTKVG

DPQELNGIVQALCGTRQSPLRIGSTKSNMGHPEPASGLAALAKVLLSLEHGLWAPNLHFH

NPNPKIPALQDGRLQVVDRPLPVLGGNVGINSFGFGGSNVHVILQPNSQPLPPPAPHAAL

PRLLRASGRTLEGVQGLLELGLQHSQNLAFVSMLNDIATPSPAAMPFRGYAVLGSQGGSQ

KVQQVLAGKRPLWFICSGMGTQWRGMGLSLMRLSRFRDSILRSDEAVKPLGLQVSQLLLS

TDEAIFDDMVISFVSLTAIQIALIDLLTSMGLQPDGIIGHSLGEVACGYADGCISQEEAI

LSAYWRGQCIKEANIPPGAMAAVGLTWEECKQRCPPGIVPACHNCIDTVTISGPQASMLE

FVQQLKQEGVFAKEVRTGGMAFHSYFMDAIAPMLLQQLKKVIREPQPRSPRWLSTSIPET

QWQESLARTFSAEYNVNNLVSPVLFQEALWRVPEDAVVLEIAPHALLQAVLKRGLKSSCT

IIPLMKKDHRDNLEFFLSNVGQLYLTGIDVNPNGLFPPVEFPAPRGTPLISPHIKWDHSQ

TWDVPTAEDFPSGSSSSSATIYKIDINPESPDHYLVDHCIDGRIIFPGTGYLCLVWKTLA

RALDQNMEHTPVVFEDVTLHQAVILPKTGIVLLKVRLLEASCTFEVSENGNLIASGKVYQ

WEDPNPKLFDNRYGPDPATPVDPTTAIHLSRGDVYKELQLQGFNYGPYFQGILEASSEGN

TGQLLWKDNWVTFMDTMLQMSILAPSKRSLRLPTRITAIYIHPATHQQKLYTLQDKTQVA

DVVINRCLDTTVAGGIYISRIHTSVAPRHQQEQLVPILEKFCFTPHVETGCLAGNLALQE

ELQLCVGLAQALQTRVAQQGIKMVVPGLDGAQAPQEAPQQGLPRLLATACQLQLNGNLQM

EMGQILAQERALLCDDPLLSGLLNSPALKACVTLALENMTSLKMKVVLAGDGQLYSRIPT

LLNTQPLLELDYTATDRHPQALEAAQAKLQQLDITQGQWDPSDPAPSNLGGANLVVCNYA

LASLGDPATAVGNMVAALKEGGFLLLHTLLRGHPLGETVTFLTCPEPQQGQRHLLSQDEW

ERLFAGASLHLVALKKSFYGSVLFLCRRLAPLDSPIFLPVEDTSFQWVDSLKNILADSSS

RAVWLMAVGCTTSGVVGLVNCLRKEPDGHRIRCVLVSNLNSTSPIPETDPKSLELQKVLQ

SDLVMNVYRDGAWGAFRHFPLEQDKPEEQTEHAFINVLTRGDLSSIRWVCSPLRHSQPTA

PGFQLCTIYYASLNFKRNHAGHGQAVPRRHPRNWASRNCLLGMEFSGRDASGKRVMGLVP

AEGLATSTLVPQSFLWDVPSNWTLEEAASVPVVYSTAYYALMVRGRMQPGETVLIHSGSG

GVGQAAIAIALSLGCRVFPLVGSAEKRAYLQSRFPQLNETSFANSRDTSFEQHVLWHTAG

KGADLVLNSLAEEKLQASVRCLAQHGRFLEIGKFDLSKNHPLGMAIFLKNVTFHGILLDS

LFEENNTMWQEVSTLLKAGIRKGVVQPLKRTVFPRTQAEDAFRYMAQGKHIGKVVIQVRE

EEQEAVLHGTKPTQMVALCKTFCPAHKSYIITGGLGGFGLELAHWLVERGAQKLVLTSRS

GIRTGYQARQVHEWRRQGVQVLVSTSDVSTLDGTRSLITEAAQLGPVGGIFNLAVVLRDA

MLDNQTPEFFQDVNKPKYNGTLNLDRVTREACPELDYFEVFSSVSCGRGNAGQTNYGFAN

STMERICEKRRHDGLPGLAVQWGAIADVGLLMELKGTKDKAIGGTLPQRITSCMEVLDLF

LNQPHPVLSSFVLAEKATSRGPSGSHQDLVKAVTHILGIRDLATVNLDSSLSDLGLDSLM

GVEVRQMLEREHNLLLSMREIRQLTIHKLQEISAQAGTADELTDSTPKFGSPAQSHTQLN

LSTLLVNPEGPTLTRLNSVQSSERPLFLVHPIEGSTTVFHSLATKLSIPTYGLQCTGAAP

LDSIQSLATYYIECIRQVQPEGNYRIAGYSYGACVAFEMCSQLQAQQNAGPTNNSLFLFD

GSHTFVMAYTQSYRAKLNPGCEAEAEAEAMCFFMQQFTEAEHSRVLEALLPLGDLEARVA

ATVELIVQSHAGLDRHALSFAARSFYHKLRAAEEYTPRATYHGNVTLLRAKMGSAYQEGL

GADYNLSQVCDGKVSVHIIEGDHRTLLEGSGLESILSIIHSSLAEPRVSVREG

>sp|P12799|FIBG_BOVIN Fibrinogen gamma-B chain OS=Bos taurus OX=9913 GN=FGG PE=1 SV=1

MSWSSHPPSVIFYILSLLSSACLAYVATRDNCCILDERFGSYCPTTCGIADFLNNYQTSV

DKDLRTLEGILYQVENKTSEARELVKAIQISYNPDQPSKPNNIESATKNSKSMMEEIMKY

ETLISTHESTIRFLQEVYNSNSQKIVNLRDKVVQLEANCQEPCQDTVKIHDVTGRDCQDV

ANKGAKESGLYFIRPLKAKQFLVYCEIDGSGNGWTVFQKRLDGSLDFKKNWIQYKEGFGH

LSPTGTGNTEFWLGNEKIHLISTQSSIPYVLRIQLEDWNGRTSTADYASFKVTGENDKYR

LTYAYFIGGDAGDAFDGYDFGDDSSDKFFTSHNGMQFSTWDSDNDKYDGNCAEQVGIGWW

MNKCHAGHLNGVYYQGGTYSKTSTPNGYDNGIIWATWKSRWYSMKKTTMKIIPLNRLAIG

EGQQHQLGGAKQVGVEHHVEIEYD

>sp|O46414|FRIH_BOVIN Ferritin heavy chain OS=Bos taurus OX=9913 GN=FTH1 PE=2 SV=3

MTTASPSQVRQNYHQDSEAAINRQINLELYASYVYLSMSYYFDRDDVALKNFAKYFLHQS

HEEREHAERLMKLQNQRGGRIFLQDIKKPDRDDWENGLTAMECALCLERSVNQSLLELHK

LATEKNDPHLCDFIETHYLNEQVEAIKELGDHITNLRKMGAPGSGMAEYLFDKHTLGHSE

S

>sp|Q2T9Y6|GSH0_BOVIN Glutamate--cysteine ligase regulatory subunit OS=Bos taurus OX=9913 GN=GCLM PE=2 SV=1

MGTDSRAAGALLARASTLHLQTGNLLNWGRLRKKCPSTHSEELRDCIQKTLNEWSSQISP

DLIREFPDVLECTVSHAVEKINPDEREEMKVSAKLFIVGSNSSSSTRNAVDMACSVLGVA

QLDSVIIASPPVEDGVNLSLEHLQPYWEELQNLVQSKKIVAIGTSDLDKTQLEQLYQWAQ

VKPNSNQVNLASCCVMPPDLTAFAKQFDIQLLTHNDPKELLSEASFQEALQESIPDIRAH

EWVPLWLLRYSVIVKSRGIIKSKGYILQAKRKGS

>sp|Q2YDG0|GPC5C_BOVIN G-protein coupled receptor family C group 5 member C OS=Bos taurus OX=9913 GN=GPRC5C PE=2 SV=2

MAIHRTVLMCLGLPLFLLPGARAQEQAPPGCSPDLNPLYYNLCDRSEAWGIILEAVAGAG

VVTTFVLTIILVASLPFVQDTKKRSLLGTQVFFLLGTLGLFCLVFACVVKPSFSTCASRR

FLFGVLFAICFSCLVAHVLALHFLVRKNHGPRGWVIFLVALLLSLVEVIINTEWLIITLV

RGAGTEGDALGNGSAGWVAVSPCAIANADFVMALIYVMLLLLCAFSGAWSALCGRFKRWR

KHGVFILLTTTASIAVWVVWIVMYTYGNRQHNSPTWDDPTLAIALATNAWAFVLFYVIPE

VSQVTRSSPEQSYQGDLYPTRGVGYETILKEQKGQSMFVENKAFSMDEPASAKRPVSPYS

GYNGQLLTSMYQPTEMTLMHKAPSDGAYDVILPRATANSQVTGSANSTLRAEDIYAAQGR

QEATLPKEGKNSQVFRNPYVWD

>sp|Q0VCA8|3HAO_BOVIN 3-hydroxyanthranilate 3,4-dioxygenase OS=Bos taurus OX=9913 GN=HAAO PE=1 SV=1

MERPVRVKAWVEENRGSFLPPVCNKLLHQKQLKIMFVGGPNTRKDYHIEEGEEVFYQLEG

DMLLRVLERGKHRDVVIRQGEIFLLPAGVPHSPQRFANTVGLVIERRRLKTELDGLRYYV

GDTTDVLFEKWFYCEDLGTQLAPIIQEFFSSEQYRTGKPNPDQLLKEPPFPLSTRSVMEP

MCLEAWLDGHRKELQAGTPLSLFGDTYESQVMVHGQGSSEGLRRDVDVWLWQLEGSSVVT

MEGQRLSLTLDDSLLVPAGTLYGWERGQGSVALSVTQDPACKKSLG

>sp|Q5E9Z2|HABP2_BOVIN Hyaluronan-binding protein 2 OS=Bos taurus OX=9913 GN=HABP2 PE=2 SV=1

MFARMSDLHVLLLMVLAGKTAFGLSLLSFLTEPDPDWTPDQYEYSQEYNNQEENASSTTA

YSDNPDWYYEEDDPCLSNPCTHGGDCLVSGATFTCRCPDPFSGNRCQNVQNKCKNNPCGR

GDCLITQSPPYHRCACKHPYRGSDCSRVVPVCRPNPCQNGGTCSRQRRRSKFTCACPDQF

KGKLCEIGPDDCYVDDGYSYRGRVSKTIHQHTCLYWNSHLLLQEKYNMFMEDAEAHGIGE

HNFCRNPDGDKKPWCFIKVNNVKVKWEYCDVPACSALDVANPEGRPTEPLTKFPEFGSCG

RTEIAEKKVKRIFGGFKSTAGKHPWQASLQTSLRLTVSTPQGHYCGGALIHPCWVLTAAH

CTEIKTKYLKVVLGDQDLTKTEFHEQSFGVQKIFKYSHYIEIDDIPYNDIALLKLKPVDG

HCALESKYVKTVCLPDGPFLSGTECYISGWGVTETGEGSRHLLDAKVKLISNTICNSRQL

YDHSIDDNMICAGNLQKPGQDSCQGDSGGPLTCEKDGTSYIYGIVSWGLECGKRPGVYTQ

VTKFLTWIKATMEKEASF

>sp|Q3T0D0|HNRPK_BOVIN Heterogeneous nuclear ribonucleoprotein K OS=Bos taurus OX=9913 GN=HNRNPK PE=2 SV=1

METEQPEETFPNTETNGEFGKRPAEDMEEEQAFKRSRNTDEMVELRILLQSKNAGAVIGK

GGKNIKALRTDYNASVSVPDSSGPERILSISADIETIGEILKKIIPTLEEGLQLPSPTAT

SQLPLESDAVECLNYQHYKGSDFDCELRLLIHQSLAGGIIGVKGAKIKELRENTQTTIKL

FQECCPQSTDRVVLIGGKPDRVVECIKIILDLISESPIKGRAQPYDPNFYDETYDYGGFT

MMFDDRRGRPVGFPMRGRGGFDRMPPGRGGRPMPPSRRDYDDMSPRRGPPPPPPGRGGRG

GSRARNLPLPPPPPPRGGDLMAYDRRGRPGDRYDGMVGFSADETWDSAIDTWSPSEWQMA

YEPQGGSGYDYSYAGGRGSYGDLGGPIITTQVTIPKDLAGSIIGKGGQRIKQIRHESGAS

IKIDEPLEGSEDRIITITGTQDQIQNAQYLLQNSVKQYADVEGF

>sp|P33433|HRG_BOVIN Histidine-rich glycoprotein (Fragments) OS=Bos taurus OX=9913 GN=HRG PE=1 SV=1

AVNPTGCDAVEPVAVRALDLINKGRDGYLFQLLRVADAHLDKVESIAVYYLVESDCPVLS

RKHWDDCELNVTVIGQCKLAGPEDLSVNDFNCTTSSVSSALTNMRARGGEGTSYFLDFSV

RNCSSHHFPRHHIFGFCRADLFYDVEASDLETPKDIVTNCEVFHRRFSAVQHHLGRPFHS

GEHEHSPAGRPPFKPSGSKDHGHPHESYNFRCPPPLEHKNHSDSPPFQARAPLPFPPPGL

RCPHPPFGTKGNHRPPHDHSSDEHHPHGHHPHGHHPHGHHPHGHHPPDNDFYDHGPCDPP

PHRPPPRHSKERGPGKGHFRFHWRPTGYIHRLPSLKKGEVLPLPEANFPSFSLPNHNNPL

QPEIQAFPQSASESCPGTFNIKFLHISKFFAYTLPK

>sp|F1N152|HTRA1_BOVIN Serine protease HTRA1 OS=Bos taurus OX=9913 GN=HTRA1 PE=2 SV=1

MQPPRAPFLPPPTPPLLLLLLLLAAPASAQPARAGRSAPGAAGCPERCDPARCAPPPGSC

EGGRVRDACGCCEVCGAPEGAECGLQEGPCGEGLQCVVPFGVPASATVRRRAQSGLCVCA

SNEPVCGSDAKTYTNLCQLRAASRRSERLHQPPVIVLQRGACGQGQEDPNSLRHKYNFIA

DVVEKIAPAVVHIELFRKLPFSKREVPVASGSGFIVSEDGLIVTNAHVVTNKHRVKVELK

NGATYEAKIKDVDEKADIALIKIDHQGKLPVLLLGRSSELRPGEFVVAIGSPFSLQNTVT

TGIVSTTQRGGKELGLRNSDMDYIQTDAIINYGNSGGPLVNLDGEVIGINTLKVTAGISF

AIPSDKIKKFLTESHDRQAKGKAITKKKYIGIRMMSLTPSKAKELKDRHRDFPDVLSGAY

IIEVIPDTPAEAGGLKENDVIISINGQSVVSANDVSDVIKKESTLNMVVRRGNEDIMITV

IPEEIDP

>sp|P13384|IBP2_BOVIN Insulin-like growth factor-binding protein 2 OS=Bos taurus OX=9913 GN=IGFBP2 PE=1 SV=2

MQPRLGGPALLLLPPLLLLLLLGAGGGDCGARAEVLFRCPPCTPESLAACKPPPGAAAGP

AGDARVPCELVREPGCGCCSVCARLEGERCGVYTPRCGQGLRCYPNPGSELPLRALVHGE

GTCEKHGDAEYSASPEQVADNGEEHSEGGLVENHVDGNVNLMGGGGGAGRKPLKSGMKEL

AVFREKVTEQHRQMGKGGKHHLGLEEPKKLRPPPARTPCQQELDQVLERISTMRLPDERG

PLEHLYSLHIPNCDKHGLYNLKQCKMSLNGQRGECWCVNPNTGKLIQGAPTIRGDPECHL

FYNEQQGARGVHTQRMQ

>sp|Q05718|IBP6_BOVIN Insulin-like growth factor-binding protein 6 OS=Bos taurus OX=9913 GN=IGFBP6 PE=2 SV=2

MTPHRLLPPLLLTLLLAARPGGALARCPGCGQGVSAGCPGGCAEEEDGGPAAEGCAEAGG

CLRREGQQCGVYTPNCAPGLQCQPPEKEDLPLRALLQGRGRCGRARTPSGENPKESKPQA

GTARSQDVNRRDQQRNSGTSTTPSRSNSGGVQDTEMGPCRKHLDSVLQQLQTEVFRGAHT

LYVPNCDHRGFYRKRQCRSSQGQRRGPCWCVERMGQPLPGSSEGGDGSSLCPTGSSG

>sp|P53712|ITB1_BOVIN Integrin beta-1 OS=Bos taurus OX=9913 GN=ITGB1 PE=1 SV=3

MNLQLIFWIGLISSVCCVFGQADENRCLKANAKSCGECIQAGPNCGWCTNSTFLQEGMPT

SARCDDLEALKKKGCHPNDIENPRGSKDIKKNKNVTNRSKGTAEKLQPEDITQIQPQQLV

LQLRSGEPQTFTLKFKRAEDYPIDLYYLMDLSYSMKDDLENVKSLGTDLMNEMRRITSDF

RIGFGSFVEKTVMPYISTTPAKLRNPCTNEQNCTSPFSYKNVLSLTDKGEVFNELVGKQR

ISGNLDSPEGGFDAIMQVAVCGSLIGWRNVTRLLVFSTDAGFHFAGDGKLGGIVLPNDGQ

CHLENDVYTMSHYYDYPSIAHLVQKLSENNIQTIFAVTEEFQPVYKELKNLIPKSAVGTL

SANSSNVIQLIIDAYNSLSSEVILENSKLPEGVTINYKSYCKNGVNGTGENGRKCSNISI

GDEVQFEISITANKCPNKNSETIKIKPLGFTEEVEIILQFICECECQGEGIPGSPKCHDG

NGTFECGACRCNEGRVGRHCECSTDEVNSEDMDAYCRKENSSEICSNNGECVCGQCVCRK

RDNTNEIYSGKFCECDNFNCDRSNGLICGGNGVCKCRVCECNPNYTGSACDCSLDTTSCM

AVNGQICNGRGVCECGACKCTDPKFQGPTCEMCQTCLGVCAEHKECVQCRAFNKGEKKDT

CAQECSHFNITKVENRDKLPQPGQVDPLSHCKEKDVDDCWFYFTYSVNGNNEATVHVVET

PECPTGPDIIPIVAGVVAGIVLIGLALLLIWKLLMIIHDRREFAKFEKEKMNAKWDTGEN

PIYKSAVTTVVNPKYEGK

>sp|Q148H6|K1C28_BOVIN Keratin, type I cytoskeletal 28 OS=Bos taurus OX=9913 GN=KRT28 PE=2 SV=1

MSLRFSSGSRHICLRSGTESVRPSSGGTGFAGSNVYGNSGAGCGFSYALGGGLGSLPGGD

HAGGIPGSGTCVGFAGSEGGLFSGNEKVTMQNLNDRLASYLDNVRALEEANAELERKIKS

WYEKHGPGSCHGLDHDYSRYHLTIEDLKNKIISSTTANANVILQIDNARLAADDFRLKYE

NELALHQNTEADINGLRRVLDELTLCRTDQELQYESLSEEMTYLKKNHEEEVKALQCVAG

GNVNVEMNAAPGVDLTLLLNNMRAEYEDLAEQNRRDAEAWFNEKSASLQQQISDDAGAAS

SARGELTEMKRTVQTLDIELQSLLATKHSLECSLMETEGNYCAQLAQIQAQIGALEEQLH

QVRTETEGQKLEYEQLLDIKVHLEKEIETYCRLIDGDRNSCSKSKGFGSGSPGNSSKDLS

RTTLVKTVVEEIDQRGKVLSSRVQSIEEKTSKMTNGKTKQRVPF

>sp|Q148H5|K2C71_BOVIN Keratin, type II cytoskeletal 71 OS=Bos taurus OX=9913 GN=KRT71 PE=2 SV=1

MSRQFTCKSGAAAKGGFSGCSAVLSGGSTSSYRAGGKGLSGGFGSRSLYNLGGVRSISFN

VASGSGKSGGYGFGRGRASGFAGSMFGSVALGPMCPTVCPPGGIHQVTVNESLLAPLNVE

LDPEIQKVRAQEREQIKALNNKFASFIDKVRFLEQQNQVLETKWELLQQLDLNNCKNNLE

PILEGYISNLRKQLETLSGDRVRLDSELRSVRDVVEDYKKRYEEEINRRTAAENEFVLLK

KDVDAAYANKVELQAKVDSMDQEIKFFKCLYEAEIAQIQSHISDMSVILSMDNNRDLNLD

SIIDEVRAQYEDIALKSKAEAEALYQTKFQELQLAAGRHGDDLKNTKNEISELTRLIQRI

RSEIENVKKQASNLETAIADAEQRGDNALKDARAKLDELEAALHQSKEELARMMREYQEL

MSLKLALDMEIATYRKLLESEECRMSGEFPSPVSISIISSTSGSGGYGFRPSSVSGGYVA

NSGSCISGVCSVRGGESRSRSSTTDYKDALGKGSSLSAPSKKASR

>sp|Q3T056|LDH6B_BOVIN L-lactate dehydrogenase A-like 6B OS=Bos taurus OX=9913 GN=LDHAL6B PE=2 SV=1

MSWTVGILRASQRAGTVRANFLCLGMAPSPCPPAATPLGGAWAVAPASKMATVKCELMKN

FTSEEPVRNSKISIVGTGSVGMACAVSILLKGLSDELALVDVDEGRLKGETMDLQHGSLF

VKMPNIVSSRDYVVTANSNLVIITAGARQEKGETRLNLVQRNVAIFKLMMSSIVQYSPRC

KLIVVSNPVDILTYVAWKLSAFPQNRVIGSGCNLDTARFRFLIGQRLSIHSESCHGWILG

EHGDSSVPVWSGVNIAGVPLKELNLDIGTDKDPEQWKNVHKDVVASAYEIIKMKGYTYWA

IGLSVADLTESILKNLRRVHPVSTRIKGLYGINEEVFLSVPCILGESGITDLIKVKLAPE

EEARLQKSAKTLWDIQKELKF

>sp|Q2KJ33|LIMS2_BOVIN LIM and senescent cell antigen-like-containing domain protein 2 OS=Bos taurus OX=9913 GN=LIMS2 PE=2 SV=2

MTGSNMSNALANAVCQRCQARFAPAERIVNSNGELYHEHCFVCAQCFRPFPEGLFYEFEG

RKYCEHDFQMLFAPCCGSCGEFIIGRVIKAMNNNWHPGCFRCELCDVELADLGFVKNAGR

HLCRPCHNREKAKGLGKYICQRCHLVIDEQPLMFKNDAYHPDHFSCTHCGKELTAEAREL

KGELYCLPCHDKMGVPICGACRRPIEGRVVNALGKQWHVEHFVCAKCEKPFLGHRHYEKK

GLAYCETHYNQLFGDVCYTCSHVIEGDVVSALNKAWCVHCFSCSTCNSRLTLKNKFVEFD

MKPVCKRCYEKFPLELKKRLKKLSELAARRAQPKSSGLHPA

>sp|P24627|TRFL_BOVIN Lactotransferrin OS=Bos taurus OX=9913 GN=LTF PE=1 SV=2

MKLFVPALLSLGALGLCLAAPRKNVRWCTISQPEWFKCRRWQWRMKKLGAPSITCVRRAF

ALECIRAIAEKKADAVTLDGGMVFEAGRDPYKLRPVAAEIYGTKESPQTHYYAVAVVKKG

SNFQLDQLQGRKSCHTGLGRSAGWIIPMGILRPYLSWTESLEPLQGAVAKFFSASCVPCI

DRQAYPNLCQLCKGEGENQCACSSREPYFGYSGAFKCLQDGAGDVAFVKETTVFENLPEK

ADRDQYELLCLNNSRAPVDAFKECHLAQVPSHAVVARSVDGKEDLIWKLLSKAQEKFGKN

KSRSFQLFGSPPGQRDLLFKDSALGFLRIPSKVDSALYLGSRYLTTLKNLRETAEEVKAR

YTRVVWCAVGPEEQKKCQQWSQQSGQNVTCATASTTDDCIVLVLKGEADALNLDGGYIYT

AGKCGLVPVLAENRKSSKHSSLDCVLRPTEGYLAVAVVKKANEGLTWNSLKDKKSCHTAV

DRTAGWNIPMGLIVNQTGSCAFDEFFSQSCAPGADPKSRLCALCAGDDQGLDKCVPNSKE

KYYGYTGAFRCLAEDVGDVAFVKNDTVWENTNGESTADWAKNLNREDFRLLCLDGTRKPV

TEAQSCHLAVAPNHAVVSRSDRAAHVKQVLLHQQALFGKNGKNCPDKFCLFKSETKNLLF

NDNTECLAKLGGRPTYEEYLGTEYVTAIANLKKCSTSPLLEACAFLTR

>sp|Q3MHR0|LYPA1_BOVIN Acyl-protein thioesterase 1 OS=Bos taurus OX=9913 GN=LYPLA1 PE=2 SV=1

MCGNNMSAPLPAIVPAARKATAAVIFLHGLGDTGHGWAEAFAGIRSAHIKYICPHAPVMP

VTLNMNMAMPSWFDIIGLSPDSLEDETGIKQAAENVKALIDQEVKNGIPSNRIILGGFSQ

GGALSLYTALTTQQKLAGVTALSCWLPLRASFPQGPIGGVNRDISILQCHGDLDPLVPLM

FGSLTAEKLKTLVNPANVTFRTYAGMMHSSCQQEMMDIKQFIDKLLPPVD

>sp|Q3SZP2|MARE2_BOVIN Microtubule-associated protein RP/EB family member 2 OS=Bos taurus OX=9913 GN=MAPRE2 PE=2 SV=1

MPGPTQTLSPNGENNNDIIQDNGTIIPFRKHTVRGERSYSWGMAVNVYSTSITQETMSRH

DIIAWVNDIVSLNYTKVEQLCSGAAYCQFMDMLFPGCISLKKVKFQAKLEHEYIHNFKLL

QASFKRMNVDKVIPVEKLVKGRFQDNLDFIQWFKKFYDANYDGKEYDPVEARQGQDAIPP

PDPGEQIFNLPKKSHHANSPTAGAAKSSPASKPGSTPSRPSSAKRASSSGSASRSDKDLE

TQVIQLNEQVHSLKLALEGVEKERDFYFGKLREIELLCQEHGQENDDLVQRLMDVLYASD

EHEGHPEEPEAEEQVHEQQPQQQEEY

>sp|Q2NL31|MTNA_BOVIN Methylthioribose-1-phosphate isomerase OS=Bos taurus OX=9913 GN=MRI1 PE=2 SV=1

MTLEAIRYSRGSLQILDQLLLPQQSRYEAVGSVRQAWEAIRAMKVRGAPAIALVGCLSLA

VELQAGAGGPGLAALVAFVQDALSFLVTARPTAVNMARAARDLADLAAQEAEREGATEEA

VRERVICWAEDMLDKDLRDNRSIGDLGAHHLLKRAAPQGGKVTVLTHCNTGALATAGYGT

ALGVIRSLHNLGRLEHAFCTETRPYNQGARLTAFELVYEQIPATLIADSMAAAAMAHQGV

SAVVVGADRVVANGDTANKVGTYQLAIAAKHHGIPFYVAAPSSSCDLRLETGREIVIEER

PDQELTDVNGVRIAAPGIGVWNPAFDVTPHDLITGGIITELGVFAPEELQAALSATIS

>sp|P54149|MSRA_BOVIN Mitochondrial peptide methionine sulfoxide reductase OS=Bos taurus OX=9913 GN=MSRA PE=1 SV=2

MLSATRRALQLFHSLFPIPRMGDSAAKIVSPQEALPGRKEPLVVAAKHHVNGNRTVEPFP

EGTQMAVFGMGCFWGAERKFWTLKGVYSTQVGFAGGYTPNPTYKEVCSGKTGHAEVVRVV

FQPEHISFEELLKVFWENHDPTQGMRQGNDHGSQYRSAIYPTSAEHVGAALKSKEDYQKV

LSEHGFGLITTDIREGQTFYYAEDYHQQYLSKDPDGYCGLGGTGVSCPLGIKK

>sp|Q24K22|HGFL_BOVIN Hepatocyte growth factor-like protein OS=Bos taurus OX=9913 GN=MST1 PE=2 SV=1

MGWLPLLLLLIWFSGAPGQRSPLNDFQVLRGTELQHLLHSVGPGPWQEDVANAEECAGLC

GPLLDCRAFHYNLSSHGCQLLPWTQHSPHTRLQRSGRCDLFQKKNYVRTCIVDNGVEYRG

TVAITVGGLPCQRWSHRFPNDHKFTPTLRNGLEENFCRNPDRDPGGPWCYTTDPAVRFQS

CGIKSCREATCLWCNGEDYRGSVDSTESGRECQRWDLQHPHPHPFEPGFWTKIWTTTIAG

IRTARSGPGAIPPTRRWRESSATYPAAVQRSEAQPSQEATTLNCFRGKGEGYRGTVNTTA

AGVPCQRWDAQLPHQHRFAPEKYACKDLRENFCRNPDGSEAPWCFTSRPGMRMAFCYQIR

RCTDDVRPEDCYHGAGELYRGSVSKTRKGIRCQNWSAETPHKPQFKHTSAPHTPLEENFC

RNPDGDSHGPWCYTTDPGTPFDYCALRRCDDDQQPSILETAHQVLFDKCGKRVTRVDPLH

SKLRVVGGQPGNSPWTVSLRNRQGQHFCGGSLVKEQWVLTARQCFSSCHMSLVGYEVWLG

TLFQDPQPGEPDLQHIPMAKMVCGPSGSQLVLLKLERPVILNQRVALICLPPERYVVPPG

TRCEIAGWGETKGTGDDTVLNIALLSVISNQECNVKHRGRVRESEMCTAGLLAPVGACEG

DYGGPLACFTHDCWVLQGIIIPNRVCARPRWPAVFMRVSVFVDWIHKVMRLG

>sp|Q2HJ33|OLA1_BOVIN Obg-like ATPase 1 OS=Bos taurus OX=9913 GN=OLA1 PE=2 SV=1

MPPKKGGDGIKPPPIIGRFGTSLKIGIVGLPNVGKSTFFNVLTNSQASAENFPFCTIDPN

ESRVPVPDERFDFLCQYHKPASKIPAFLNVVDIAGLVKGAHNGQGLGNAFLSHISACDGI

FHLTRAFEDDDITHVEGSVDPVRDIEIIHEELQLKDEEMVGPIIDKLEKVAVRGGDKKLK

PEYDIMCKVKSWVIDQKKPVRFYHDWNDKEIEVLNKHLFLTSKPMVYLVNLSEKDYIRKK

NKWLIKIKEWVDKYDPGALVIPFSGALELRLQELSAEERQKYLEANMTQSALPKIIKAGF

AALQLEYFFTAGPDEVRAWTIRKGTKAPQAAGKIHTDFEKGFIMAEVMKYEDFKEEGSEN

AVKAAGKYRQQGRNYIVEDGDIIFFKFNTPQQPKKK

>sp|P61286|PABP1_BOVIN Polyadenylate-binding protein 1 OS=Bos taurus OX=9913 GN=PABPC1 PE=2 SV=1

MNPSAPSYPMASLYVGDLHPDVTEAMLYEKFSPAGPILSIRVCRDMITRRSLGYAYVNFQ

QPADAERALDTMNFDVIKGKPVRIMWSQRDPSLRKSGVGNIFIKNLDKSIDNKALYDTFS

AFGNILSCKVVCDENGSKGYGFVHFETQEAAERAIEKMNGMLLNDRKVFVGRFKSRKERE

AELGARAKEFTNVYIKNFGEDMDDERLKDLFGKFGPALSVKVMTDESGKSKGFGFVSFER

HEDAQKAVDEMNGKELNGKQIYVGRAQKKVERQTELKRKFEQMKQDRITRYQGVNLYVKN

LDDGIDDERLRKEFSPFGTITSAKVMMEGGRSKGFGFVCFSSPEEATKAVTEMNGRIVAT

KPLYVALAQRKEERQAHLTNQYMQRMASVRAVPNPVINPYQPAPPSGYFMAAIPQTQNRA

AYYPPSQIAQLRPSPRWTAQGARPHPFQNMPGAIRPAAPRPPFSTMRPASSQVPRVMSTQ

RVANTSTQTMGPRPAAAAAAATPAVRTVPQYKYAAGVRNPQQHLNAQPQVTMQQPAVHVQ

GQEPLTASMLASAPPQEQKQMLGERLFPLIQAMHPTLAGKITGMLLEIDNSELLHMLESP

ESLRSKVDEAVAVLQAHQAKEAAQKAVNSATGVPTV

>sp|Q28156|PDE5A_BOVIN cGMP-specific 3',5'-cyclic phosphodiesterase OS=Bos taurus OX=9913 GN=PDE5A PE=1 SV=1

MERAGPGSARPQQQWDQDSVEAWLDDHWDFTFSYFVRKGTREMVNAWFAERVHTIPVCKE

GIKGHTESCSCPLQPSPRAESSVPGTPTRKISASEFDRPLRPIVIKDSEGTVSFLSDSDK

KEQMPLTSPRFDNDEGDQCSRLLELVKDISSHLDVTALCHKIFLHIHGLISADRYSLFLV

CEDSSNDKFLISRLFDVAEGSTLEEASNNCIRLEWNKGIVGHVAAFGEPLNIKDAYEDPR

FNAEVDQITGYKTQSILCMPIKNHREEVVGVAQAINKKSGNGGTFTEKDEKDFAAYLAFC

GIVLHNAQLYETSLLENKRNQVLLDLASLIFEEQQSLEVILKKIAATIISFMQVQKCTIF

IVDEDCSDSFSSVFHMECEELEKSSDTLTRERDANRINYMYAQYVKNTMEPLNIPDVSKD

KRFPWTNENMGNINQQCIRSLLCTPIKNGKKNKVIGVCQLVNKMEETTGKVKAFNRNDEQ

FLEAFVIFCGLGIQNTQMYEAVERAMAKQMVTLEVLSYHASAAEEETRELQSLAAAVVPS

AQTLKITDFSFSDFELSDLETALCTIRMFTDLNLVQNFQMKHEVLCKWILSVKKNYRKNV

AYHNWRHAFNTAQCMFAALKAGKIQKRLTDLEILALLIAALSHDLDHRGVNNSYIQRSEH

PLAQLYCHSIMEHHHFDQCLMILNSPGNQILSGLSIEEYKTTLKIIKQAILATDLALYIK

RRGEFFELIMKNQFNLEDPHQKELFLAMLMTACDLSAITKPWPIQQRIAELVATEFFDQG

DRERKELNIEPADLMNREKKNKIPSMQVGFIDAICLQLYEALTHVSEDCFPLLDGCRKNR

QKWQALAEQQEKTLINGESSQTKRN

>sp|Q5E9E1|PDLI1_BOVIN PDZ and LIM domain protein 1 OS=Bos taurus OX=9913 GN=PDLIM1 PE=2 SV=3

MTTLQIVLQGPGPWGFRLVGGKDFEQPLAISRVTPGSKAAIGNLCVGDVITAIDGENTSN

MTHLEAQNKIKGCTDNMTLTVARSEQKIWSPLVTEEGKRHPYKMNLASEPQEALHIGSAH

NRSAVPFTASPAFSSAPRVITNQYNNPAGLYSSENISSFNNALESKTAASGQENGRALDH

SQLPSGLVIDKESEVYKMLQEKQELNEPPKQSTSFLVLQEILESEEKGDPNKPSGFRSVK

APVTKVAASIGNAQKLPMCDKCGTGIVGVFVKLRERHRHPECYVCTDCGTNLKQKGHFFV

EDQIYCEKHARERVTPPEGYDVITVFPK

>sp|P13696|PEBP1_BOVIN Phosphatidylethanolamine-binding protein 1 OS=Bos taurus OX=9913 GN=PEBP1 PE=1 SV=2

MPVDLSKWSGPLSLQEVDERPQHPLQVKYGGAEVDELGKVLTPTQVKNRPTSITWDGLDP

GKLYTLVLTDPDAPSRKDPKYREWHHFLVVNMKGNNISSGTVLSDYVGSGPPKGTGLHRY

VWLVYEQEGPLKCDEPILSNRSGDHRGKFKVASFRKKYELGAPVAGTCYQAEWDDYVPKL

YEQLSGK

>sp|Q0IIG5|PFKAM_BOVIN ATP-dependent 6-phosphofructokinase, muscle type OS=Bos taurus OX=9913 GN=PFKM PE=2 SV=1

MTHEHHAAKTLGIGKAIAVLTSGGDAQGMNAAVRAVVRVGIYTGARVFFVHEGYQGLVDG

GDNIREATWESVSMMLQLGGTVIGSARCKDFREREGRLRAAHNLVKRGITNLCVIGGDGS

LTGADTFRSEWSDLLSDLQKSGKITAEEATKSSYLNIVGLVGSIDNDFCGTDMTIGTDSA

LHRIIEIVDAITTTAQSHQRTFVLEVMGRHCGYLALVTSLSCGADWVFIPECPPDDDWEE

HLCRRLSETRNRGSRLNIIIVAEGAIDKNGKPITSEDIKNLVVKRLGYDTRVTVLGHVQR

GGTPSAFDRILGSRMGVEAVMALLEGTPDTPACVVSLSGNQAVRLPLMECVQVTKDVTRA

MDERRFDEALKLRGRSFMNNWEVYKLLAHVRPPKSKSGSHTVAVMNVGAPAAGMNAAVRS

TVRIGLIQGNRMLVVHDGFEGLAKGQIEEAGWSYVGGWTGQGGSKLGTKRTLPKKSFEQI

SANITKFNIQGLVIIGGFEAYTGGLELMEGRKQYDELCIPFVVIPATVSNNVPGSDFSVG

ADTALNTICMTCDRIKQSAAGTKRRVFIIETMGGYCGYLATMAGLAAGADAAYIFEDPFT

IRDLQVNVEHLVQKMKTTVKRGLVLRNEKCNENYTTDFIFNLYSEEGKGIFDSRKNVLGH

MQQGGSPTPFDRNFATKMGAKAMNWMSGKIKESYRNGRIFANSPDSGCVLGMRKRALVFQ

PVTELKEQTDFEHRIPKEQWWLKLRPILKILAKYEIDLDTSEHAHLEHISRKRSGEANV

>sp|Q3SZJ9|PMM2_BOVIN Phosphomannomutase 2 OS=Bos taurus OX=9913 GN=PMM2 PE=2 SV=1

MAAPGPALCLFDVDGTLTAPRQKITKDMDCFLQKLRQKIKIGVVGGSDFEKVQEQLGDDV

IKKYDYVFPENGLVAYRDGKLLCKQNIQGHLGEALIQDLINYCLSYIAKIKLPKKRGTFI

EFRNGMLNVSPIGRSCSQEERIEFYELDQKENIRQKFVEDLRKEFAGKGLTFSIGGQISF

DVFPDGWDKRYCLGHVEKDGYKTIYFFGDKTMPGGNDHEIFTDPRTVGYTVAAPEDTRRI

CEELFC

>sp|Q08E11|PPIC_BOVIN Peptidyl-prolyl cis-trans isomerase C OS=Bos taurus OX=9913 GN=PPIC PE=2 SV=1

MGPGLRPLLPLVLCVGLSALVPSAGASGFRKRGPSVTAKVFFDVRIGDKDVGRIVIGLFG

KVVPKTVENFVALATGEKGYGYKGSKFHRVIKDFMIQGGDFTRGDGTGGISIYGETFPDE

NFKLKHYGIGWVSMANAGPDTNGSQFFITLTKPTWLDGKHVVFGKVLDGMTVVHSIELQA

TDGHDRPFTDCSIVNSGKIDVKTPFVVEVSDW

>sp|P67774|PP2AA_BOVIN Serine/threonine-protein phosphatase 2A catalytic subunit alpha isoform OS=Bos taurus OX=9913 GN=PPP2CA PE=1 SV=1

MDEKVFTKELDQWIEQLNECKQLSESQVKSLCEKAKEILTKESNVQEVRCPVTVCGDVHG

QFHDLMELFRIGGKSPDTNYLFMGDYVDRGYYSVETVTLLVALKVRYRERITILRGNHES

RQITQVYGFYDECLRKYGNANVWKYFTDLFDYLPLTALVDGQIFCLHGGLSPSIDTLDHI

RALDRLQEVPHEGPMCDLLWSDPDDRGGWGISPRGAGYTFGQDISETFNHANGLTLVSRA

HQLVMEGYNWCHDRNVVTIFSAPNYCYRCGNQAAIMELDDTLKYSFLQFDPAPRRGEPHV

TRRTPDYFL

>sp|Q0P594|PP2AB_BOVIN Serine/threonine-protein phosphatase 2A catalytic subunit beta isoform OS=Bos taurus OX=9913 GN=PPP2CB PE=1 SV=1

MDDKAFTKDLDQWVEQLNECKQLNENQVRTLCEKAKEILTKESNVQEVRCPVTVCGDVHG

QFHDLMELFRIGGKSPDTNYLFMGDYVDRGYYSVETVTLLVALKVRYPERITILRGNHES

RQITQVYGFYDECLRKYGNANVWKYFTDLFDYLPLTALVDGQIFCLHGGLSPSIDTLDHI

RALDRLQEVPHEGPMCDLLWSDPDDRGGWGISPRGAGYTFGQDISETFNHANGLTLVSRA

HQLVMEGYNWCHDRNVVTIFSAPNYCYRCGNQAAIMELDDTLKYSFLQFDPAPRRGEPHV

TRRTPDYFL

>sp|Q9BGI3|PRDX2_BOVIN Peroxiredoxin-2 OS=Bos taurus OX=9913 GN=PRDX2 PE=2 SV=1

MACVCKAHVGKPAPEFQATAVVDGAFKEVKLSDYKGKYVVLFFYPLDFTFVCPTEIVAFS

DRAAEFHKLNCEVLGVSVDSQFTHLAWINTPRKEGGLGPLNIPLLADVTRKLSSDYGVLK

EDEGIAYRGLFVIDGKGVLRQVTINDLPVGRSVDEALRLVQAFQYTDEHGEVCPAGWTPG

SDTIKPNVDDSKEYFSKHN

>sp|Q9XTA2|PPCE_BOVIN Prolyl endopeptidase OS=Bos taurus OX=9913 GN=PREP PE=2 SV=1

MLSFQYPDVYRDETAVQDYHGHKICDPYAWLEDPDSEQTKAFVEAQNKITVPFLEQCPIR

GLYKERMTELYDYPKYSCNFKKGKRYFYFYNTGLQNQRVLYVQDSLEGEARVCLDPNTLS

DDGTVALRGYAFSEDGEYVAYGLSASGSDWVTIKFMKVDGAKELADVLERVKFSCMAWTH

DGKGMFYNAYPQQDGKSDGTETSTNLHQKLCYHVLGTDQSEDILCAEFPDEPKWMGGAEL

SDDGRYVLLSIREGCDPVNRLWYCDLHQEPNGITGILKWVKLIDNFEGEYDYVTNEGTVF

TFKTNRHSPNYRLINIDFTDPEESRWKVLVPEHEKDVLEWVACVRSNFLVLCYLHDVKNT

LQLHDMATGALLKTFPLEVGSVVGYSGQKKDTEIFYQFTSFLSPGIIYHCDLTKEELEPR

VFREVTVKGIDASDYQTVQIFYPSKDGTKIPMFIVHKKGIKLDGSHPAFLYGYGGFNISI

TPNYSVCRLIFVRHMGGVLAVANIRGGGEYGETWHKGGILANKQNCFDDFQCAAEYLIKE

GYTSPKRLTINGGSNGGLLVATCANQRPDLFGCVIAQVGVMDMLKFHKYTIGHAWTTDYG

CSDNKQHFEWLIKYSPLHNVKLPEADDIQYPSMLLLTADHDDRVVPLHSPKFIATLQHLV

GRSRKQNNPLLIHVDTKAGHGAGKPTAKVIEEVSDMFAFIARCLNIDWIQ

>sp|P00514|KAP0_BOVIN cAMP-dependent protein kinase type I-alpha regulatory subunit OS=Bos taurus OX=9913 GN=PRKAR1A PE=1 SV=2

MASGTTASEEERSLRECELYVQKHNIQALLKDSIVQLCTARPERPMAFLREYFEKLEKEE

AKQIQNLQKAGSRADSREDEISPPPPNPVVKGRRRRGAISAEVYTEEDAASYVRKVIPKD

YKTMAALAKAIEKNVLFSHLDDNERSDIFDAMFPVSFIAGETVIQQGDEGDNFYVIDQGE

MDVYVNNEWATSVGEGGSFGELALIYGTPRAATVKAKTNVKLWGIDRDSYRRILMGSTLR

KRKMYEEFLSKVSILESLDKWERLTVADALEPVQFEDGQKIVVQGEPGDEFFIILEGSAA

VLQRRSENEEFVEVGRLGPSDYFGEIALLMNRPRAATVVARGPLKCVKLDRPRFERVLGP

CSDILKRNIQQYNSFVSLSV

>sp|Q3T0Y5|PSA2_BOVIN Proteasome subunit alpha type-2 OS=Bos taurus OX=9913 GN=PSMA2 PE=1 SV=3

MAERGYSFSLTTFSPSGKLVQIEYALAAVAGGAPSVGIKAANGVVLATEKKQKSILYDER

SVHKVEPITKHIGLVYSGMGPDYRVLVHRARKLAQQYYLVYQEPIPTAQLVQRVASVMQE

YTQSGGVRPFGVSLLICGWNEGRPYLFQSDPSGAYFAWKATAMGKNYVNGKTFLEKRYNE

DLELEDAIHTAILTLKESFEGQMTEDNIEVGICNEAGFRRLTPTEVKDYLAAIA

>sp|Q3ZBG0|PSA7_BOVIN Proteasome subunit alpha type-7 OS=Bos taurus OX=9913 GN=PSMA7 PE=1 SV=1

MSYDRAITVFSPDGHLFQVEYAQEAVKKGSTAVGVRGKDIVVLGVEKKSVAKLQDERTVR

KICALDDNVCMAFAGLTADARIVINRARVECQSHRLTVEDPVTVEYITRYIASLKQRYTQ

SNGRRPFGISALIVGFDFDGTPRLYQTDPSGTYHAWKANAIGRGAKSVREFLEKNYTDEA

IETDDLTIKLVIKALLEVVQSGGKNIELAVMRRDQPLKILNPEEIEKYVAEIEKEKEENE

KKKQKKAS

>sp|P33672|PSB3_BOVIN Proteasome subunit beta type-3 OS=Bos taurus OX=9913 GN=PSMB3 PE=1 SV=3

MSIMSYNGGAVMAMKGKNCVAIAADRRFGIQAQMVTTDFQKIFPMGDRLYIGLAGLATDV

QTVAQRLKFRLNLYELKEGRQIKPYTLMSMVANLLYEKRFGPYYTEPVIAGLDPKTFKPF

ICSLDLIGCPMVTDDFVVSGTCTEQMYGMCESLWEPNMDPEHLFETISQAMLNAVDRDAV

SGMGVIVHIIEKDKITTRTLKARMD

>sp|Q2KI42|PSD11_BOVIN 26S proteasome non-ATPase regulatory subunit 11 OS=Bos taurus OX=9913 GN=PSMD11 PE=2 SV=3

MAAAAVVEFQRAQSLLSTDREASIDILHSIVKRDIQENDEEAVQVKEQSILELGSLLAKT

GQAAELGGLLKYVRPFLNSISKAKAARLVRSLLDLFLDMEAATGQEVELCLECIEWAKSE

KRTFLRQALEARLVSLYFDTKRYQEALHLGSQLLRELKKMDDKALLVEVQLLESKTYHAL

SNLPKARAALTSARTTANAIYCPPKLQATLDMQSGIIHAAEEKDWKTAYSYFYEAFEGYD

SIDSPKAITSLKYMLLCKIMLNTPEDVQALVSGKLALRYAGRQTEALKCVAQASKNRSLA

DFEKALTDYRAELRDDPIISTHLAKLYDNLLEQNLIRVIEPFSRVQIEHISSLIKLSKAD

VERKLSQMILDKKFHGILDQGEGVLIIFDEPPVDKTYEAALETIQNMSKVVDSLYNKAKK

LT

>sp|Q3SZJ4|PTGR1_BOVIN Prostaglandin reductase 1 OS=Bos taurus OX=9913 GN=PTGR1 PE=2 SV=1

MVHAKSWTLKKHFVGYPTNSDFELKTVELPPLKDGEVLLEALYLTVDPYMRIMAKSLKEG

DMMMGEQVARVVESKNSAFPTGTIVLAPSGWTTHSISNGEKLEKVLAEWPDTLPLSLALG

TVGMPGLTAYFGLLDICGVKGGETVLVSAAAGAVGSIVGQIAKLKGCKVVGTAGSDEKVA

WLKKHGFDVALNYKTVKSLEEALKEAAPEGYDCYFDNVGGEFSNVAITQMKKFGRIAICG

AISVYNRTSPLSPGPSPEIIIFKELHLQGFVVYRWQGEVRQKALRDLLKWVSEGKIQYHE

HVTEGFENMPAAFIGLLKGENLGKAIVKA

>sp|Q2TA29|RB11A_BOVIN Ras-related protein Rab-11A OS=Bos taurus OX=9913 GN=RAB11A PE=2 SV=3

MGTRDDEYDYLFKVVLIGDSGVGKSNLLSRFTRNEFNLESKSTIGVEFATRSIQVDGKTI

KAQIWDTAGQERYRAITSAYYRGAVGALLVYDIAKHLTYENVERWLKELRDHADSNIVIM

LVGNKSDLRHLRAVPTDEARAFAEKNGLSFIETSALDYTNVEAAFQTILTEIYRIVSQKQ

MSDRRENDMSPSNNVVPIHVPPTTENKPKVQCCQNI

>sp|Q1RMR4|RAB15_BOVIN Ras-related protein Rab-15 OS=Bos taurus OX=9913 GN=RAB15 PE=2 SV=1

MAKQYDVLFRLLLIGDSGVGKTCLLCRFTDNEFHSSHISTIGVDFKMKTIEVDGIKVRIQ

IWDTAGQERYQTITKQYYRRAQGIFLVYDISSERSYQHIMKWVSDVDEYAPEGVQKILIG

NKADEEQKRQVGREQGQQLAREYGMDFYETSACTNLNIKESFTRLTELVLQAHRKELEGL

RTRANHELALAELEEDEGKPEGPANSSKTCWC

>sp|A4FV54|RAB8A_BOVIN Ras-related protein Rab-8A OS=Bos taurus OX=9913 GN=RAB8A PE=2 SV=1

MAKTYDYLFKLLLIGDSGVGKTCVLFRFSEDAFNSTFISTIGIDFKIRTIELDGKRIKLQ

IWDTAGQERFRTITTAYYRGAMGIMLVYDITNEKSFDNIRNWIRNIEEHASADVEKMILG

NKCDVNDKRQVSKERGEKLALDYGIKFMETSAKANINVENAFYTLARDIKAKMDKKLEGN

SPQGSNQGVKITPDQQKRSSFFRCVLL

>sp|Q2HJI8|RAB8B_BOVIN Ras-related protein Rab-8B OS=Bos taurus OX=9913 GN=RAB8B PE=2 SV=1

MAKTYDYLFKLLLIGDSGVGKTCLLFRFSEDAFNTTFISTIGIDFKIRTIELDGKKIKLQ

IWDTAGQERFRTITTAYYRGAMGIMLVYDITNEKSFDNIKNWIRNIEEHASSDVERMILG

NKCDMNDKRQVSKERGEKLAIDYGIKFLEASAKSSMNVEEAFFTLARDIMTKLNRKMNDS

NSSGAGGPVKITENRSKKTSFFRCLLL

>sp|Q9TU25|RAC2_BOVIN Ras-related C3 botulinum toxin substrate 2 OS=Bos taurus OX=9913 GN=RAC2 PE=2 SV=1

MQAIKCVVVGDGAVGKTCLLISYTTNAFPGEYIPTVFDNYSANVMVDSKPVNLGLWDTAG

QEDYDRLRPLSYPQTDVFLICFSLVSPASYENVRAKWFPEVRHHCPSTPIILVGTKLDLR

DDKDTIEKLKEKKLAPITYPQGLALAKEIDSVKYLECSALTQRGLKTVFDEAIRAVLCPQ

PTRPQKRPCSIL

>sp|P18902|RET4_BOVIN Retinol-binding protein 4 OS=Bos taurus OX=9913 GN=RBP4 PE=1 SV=1

ERDCRVSSFRVKENFDKARFAGTWYAMAKKDPEGLFLQDNIVAEFSVDENGHMSATAKGR

VRLLNNWDVCADMVGTFTDTEDPAKFKMKYWGVASFLQKGNDDHWIIDTDYETFAVQYSC

RLLNLDGTCADSYSFVFARDPSGFSPEVQKIVRQRQEELCLARQYRLIPHNGYCDGKSER

NIL

>sp|Q9GKN7|RHAG_BOVIN Ammonium transporter Rh type A OS=Bos taurus OX=9913 GN=RHAG PE=2 SV=1

MRFKFPLMAIGLEVVMIVLFALFVQYETSVNTSRNPNETESAAMDVEKTMESYPFFQDVH

IMVFAGFGFLMTFLWKYGFSGVGINLLIAALGLQWGTIIQGIFRSHGQKFLIEMKNMIHA

DFSTVTVLISFGAVLGKTSPVQMLIMTILEITVYAANEYLVFKILWASDTGESMTIHAFG

AYFGLAVAGILYRSGLKEKHSNEESVYHSDLFAMIGSLFLWIFWPSFNSATADEAKKQYR

AIVNTYFSLAASVVTAYACSSLLESRGKLNMVHIQNATLAGGVAVGTCADMEIPPYYAMI

IGSIAGAVSVFGFKFLTPLFTTKLRIHDTCGVHNLHGLPGVIGGLAGIITVALEESDSTK

TVSQAAALGSSIATALVGGLITGAILKIPFWAQPPDEDCYDDSVYWEVPERKEYDNHFHE

LLSTLH

>sp|P61585|RHOA_BOVIN Transforming protein RhoA OS=Bos taurus OX=9913 GN=RHOA PE=1 SV=1

MAAIRKKLVIVGDGACGKTCLLIVFSKDQFPEVYVPTVFENYVADIEVDGKQVELALWDT

AGQEDYDRLRPLSYPDTDVILMCFSIDSPDSLENIPEKWTPEVKHFCPNVPIILVGNKKD

LRNDEHTRRELAKMKQEPVKPEEGRDMANRIGAFGYMECSAKTKDGVREVFEMATRAALQ

ARRGKKKSGCLVL

>sp|Q1RMJ6|RHOC_BOVIN Rho-related GTP-binding protein RhoC OS=Bos taurus OX=9913 GN=RHOC PE=2 SV=1

MAAIRKKLVIVGDGACGKTCLLIVFSKDQFPEVYVPTVFENYIADIEVDGKQVELALWDT

AGQEDYDRLRPLSYPDTDVILMCFSIDSPDSLENIPEKWTPEVKHFCPNVPIILVGNKKD

LRQDEHTRRELAKMKQEPVRSEEGRDMANRISAFGYLECSAKTKEGVREVFEMATRAGLQ

VRKNKRRRGCPIL

>sp|P26452|RSSA_BOVIN 40S ribosomal protein SA OS=Bos taurus OX=9913 GN=RPSA PE=2 SV=4

MSGALDVLQMKEEDVLKFLAAGTHLGGTNLDFQMEQYIYKRKSDGIYIINLKRTWEKLLL

AARAIVAIENPADVSVISSRNTGQRAVLKFAAATGATPIAGRFTPGTFTNQIQAAFREPR

LLVVTDPRADHQPLTEASYVNLPTIALCNTDSPLRYVDIAIPCNNKGAHSVGLMWWMLAR

EVLRMRGTISREHPWEVMPDLYFYRDPEEIEKEEQAAAEKAVTKEEFQGEWTAPAPEFTA

AQPEVADWSEGVQVPSVPIQQFPTEDWSAQPSTEDWSAAPTAQATEWVGTTTEWS

>sp|A2VDS1|SCLY_BOVIN Selenocysteine lyase OS=Bos taurus OX=9913 GN=SCLY PE=2 SV=1

MEAAGARNRDARSRAEKSPPESRKVYMDYNATTPLEPEVIEAMTEAMREAWGNPSSSYPA

GRKAKEIINTARENLAKMIGGQPQDVIFTSGGTESNNLVIQSVVKHFHKVHAANGDTGGH

PNPVDGALPHIITCTVEHDSIRLPLEHLREERVAEVTFVPVSKVNGQVEAEDILAAVRPA

TCLVTIMLANNETGVIMPVPEISRRVRALNQQRVAGGLPGVLVHTDAAQALGKQRVDVRD

LGVDFLTIVGHKFYGPRIGALYVRGLGEHTPLYPMLFGGGQERNFRPGTENTPMIAGLGK

AAELVAENGEAYEAHMRGVRDYLEERLAAEFGERIHLNSQFPGAERLPNTCNFSIRGPQL

QGRAVLAQCRTLLASVGAACHSDLGDRPSPVLLSCGVPVDVARNAIRLSVGRSSTRAEVD

LVVQDLKQAVARLEGQA

>sp|Q2KJ32|SBP1_BOVIN Methanethiol oxidase OS=Bos taurus OX=9913 GN=SELENBP1 PE=1 SV=1

MATKCGKCGPGYPSPLEAMKGPREELVYLPCIYRNTGTEAPDYLATVDVNPKSPQYSQVI

HRLPMPNLKDELHHSGWNTCSSCFGDSTKSRTKLLLPSLISSRVYVVDVATEPRAPKLHK

VVEPEEIHAKCDLSYLHTSHCLASGEVMISALGDPRGNGKGGFVLLDGETFEVKGTWEQP

GGAAPMGYDFWYQPRHNVMISTEWAAPNVLRDGFNPADVEAGLYGQHLYVWDWQRHERVQ

TLTLQDGLIPLEIRFLHNPAADQGFVGCALGSNIQRFYKNQGGTWSVEKVIQVPPKKVKG

WILPEMPSLITDILLSLDDRFLYFSNWLHGDLRQYDISDPKRPRLVGQIFLGGSIVKGGP

VQVLEDQELKCQPEPLVVKGKRVAGGPQMIQLSLDGTRLYVTTSLYSAWDKQFYPDLIRE

GSVMLQIDVDTVRGGLKLNPNFLVDFGKEPLGPALAHELRYPGGDCSSDIWL

>sp|P27674|GTR1_BOVIN Solute carrier family 2, facilitated glucose transporter member 1 OS=Bos taurus OX=9913 GN=SLC2A1 PE=1 SV=1

MEPTSKKLTGRLMLAVGGAVLGSLQFGYNTGVINAPQKVIEEFYNQTWVQRYGEPIPPAT

LTTLWSLSVAIFSVGGMIGSFSVGLFVNRFGRRNSMLMMNLLAFVSAVLMGFSKLGKSFE

MLILGRFIIGVYCGLTTGFVPMYVGEVSPTELRGALGTLHQLGIVVGILIAQVFGLDSIM

GNQELWPLLLSVIFIPALLQCILLPFCPESPRFLLINRNEENRAKSVLKKLRGTADVTRD

LQEMKEESRQMMREKKVTILELFRSAAYRQPILIAVVLQLSQQLSGINAVFYYSTSIFEK

AGVQQPVYATIGSGIVNTAFTVVSLFVVERAGRRTLHLIGLAGMAGCAVLMTIALALLER

LPWMSYLSIVAIFGFVAFFEVGPGPIPWFIVAELFSQGPRPAAIAVAGFSNWTSNFIVGM

CFQYVEQLCGPYVFIIFTVLLVLFFIFTYFKVPETKGRTFDEIASGFRQGGASQSDKTPE

ELFHPLGADSQV

>sp|Q0VD19|ASM_BOVIN Sphingomyelin phosphodiesterase OS=Bos taurus OX=9913 GN=SMPD1 PE=2 SV=1

MPRHGVSPGQGLPRSGREQASDRSLGAPCLRLLWLGLALALALPNSPVLWSPAEARPLPT

QGHPAKFIRIAPQLQEAFGWWNLTCPTCKGLFTAIDFGLRNQASVAWVGSVAIKLCVLLK

IAPPAVCQSAVQLFEDDMVEVWTRSVLSPSEACGLLLGSSCGHWDIFSSWNISLPAVPKP

PPQPPKPPAPGSPVSRVLFLTDLHWDHDYLEGTDPNCENPLCCRRDSGPPPASQPGAGYW

GEYSKCDLPLRTLESLLSGLGPAGPFDMVYWTGDIPAHNIWQQSRQDQLRALTTITALVK

KFLGPVPVYPAVGNHESTPVNGFPPPFIKGNQSSHWLYEAMAEAWEPWLPAEALRTLRIG

GFYALSPRPGLRLISLNMNFCSRENFWLLINSTDPAGQLQWLVGELQAAEDRGDKVHIIG

HIPPGHCLKSWSWNYYRIVERYENTLAGQFFGHTHVDEFEVFYDEETLSRPLSVAFLAPS

ATTYIGLNPGYRVYQIDGNYSGSSHVVLDHETYIMNLTEANEPGATPHWYLLYRARETYG

LPNALPTAWHDLVYRMRKDTQLFQTFWFLYHKGHPPSEPCGTPCRLATLCAQLSARSDSP

ALCRHLVPDASLPDVQSLWSMPLLC

>sp|Q08DX7|SPNS1_BOVIN Protein spinster homolog 1 OS=Bos taurus OX=9913 GN=SPNS1 PE=2 SV=1

MSGSDTAPFLSQADDTDDGPAPGTPGLPGSMGNPKSEDPAVPDQEGLQRITGLSSGHSAL

IVAVLCYINLLNYMDRFTVAGVLPDIEQFFDIGDGSSGLIQTVFISSYMVLAPVFGYLGD

RYNRKYLMCGGIAFWSLVTLGSSFIPRERFWLLLLTRGLVGVGEASYSTIAPTLIADLFV

ADQRSRMLSVFYFAIPVGSGLGYIAGSKVKDVAGDWHWALRVTPGLGVLAVVLLFLVVQE

PPRGAVERHSDSPPLNPTSWWADLRALARNPSFILSSLGFTAVAFVTGSLALWAPAFLLR

SRVVLGETPPCLPGDSCSSSDSLIFGLITCLTGVLGVGLGVEISRRLRRTNPRADPLVCA

AGLLGSAPFLFLALACARGSIVATYIFIFIGETLLSMNWAIVADILLYVVIPTRRSTAEA

FQIVLSHLLGDAGSPYLIGSISDRLRRDWPPSFLSEFRALQFSLMLCAFVGALGGAAFLG

TAIFIESDRRQAQLHVQGLLPETGPTDDRIVVPQRGRSTRVPVSSVLI

>sp|P55906|BGH3_BOVIN Transforming growth factor-beta-induced protein ig-h3 OS=Bos taurus OX=9913 GN=TGFBI PE=1 SV=2

MALLGRLLPLALALALGPAATPAGPARSPYQLVLQHSRLRGRQHGPNVCAVQKLIGTNKK

YFTNCKQWYQRKICGKSTVISYECCPGYEKVPGEKGCPAALPLSNLYETLGVVGATTTQL

YTDRTEKLRPEMEGPGSFTIFAPSNEAWSSLPAEVLDSLVSNVNIELLNALRYHMVDRRV

LTDELKHGMALTSMYQNSNIQIHHYPNGIVTVNCARLLKADHHATNGVVHLIDKVISTVT

NNIQQIIEIEDTFETLRAAVAASGLNTLLEGDGQYTLLAPTNEAFEKIPAETLNRILGDP

EALRDLLNNHILKSAMCAEAIVAGLSLETLEGTTLEVGCSGDMLTINGKPIISNKDVLAT

NGVIHFIDELLIPDSAKTLFELAADSDVSTAIDLFRQAGLSSHLSGNERLTLLAPMNSVF

KDGTPPINARTKNLLLNHMIKDQLASKYLYHGQTLDTLGGRKLRVFVYRNSLCIENSCIA

AHDKRGRYGTLFTMDRMLTPPMGTVMDVLKGDNRFSMLVAAIQSAGLTETLNREGVYTVF

APTNEAFQALPRGELNKLMGNAKELANILKYHVGDEILVSGGIGALVRLKSLQGDKLEVS

SKNNVVSVNKEPVAEVDIMATNGVVHAISSVLQPPANRPQERGDELADSALEIFKQASAF

SRATQSSVKLAPVYQRLLERMKH

>sp|Q06805|TIE1_BOVIN Tyrosine-protein kinase receptor Tie-1 OS=Bos taurus OX=9913 GN=TIE1 PE=2 SV=1

MVWLEPPLLLPIFFLASHVGAAVDLTLLADLRLTEPQRFFLTCVSGEAGAGRGSDAWGPP

LLLEKDDRIVRTPRPWQPPHIARNGSSRVTVRGFSQPSDLVGVFSCVGGGGTRVLYVHNS

PGAHLLPDKVTHTVNKGDTAVLSARVRKEKQTDVIWKSNGSYFYTLDRHEAQDGQFLLQL

PNVQPSSSGIYSATYLEASPLGSAFFRLIVRGCEAGRWGQDCTKECPGCLHGGVCHDQDG

ECVCPPGFTGTRCEQACREGRFGQSCQEQCPGTSGCRGLTFCLPDPYGCSCGSGWRGSQC

QEACAPGRFGADCHLQCQCQNGGTCDRFSGCVCPSGWHGMHCEKSDRIPQILDMVSELEF

NLDTMPRINCAAAGNPFPVRGSMELRKPDGTVLLSTKAIVEPDRTTAEFEVPRLALGDSG

LWECRVSTSGGQDSRRFRINVKVPPVPLTAPRLLAKQSRQLVVSPLVSFSGDGPIASVRL

HYRPQDSTMAWSTIVVDPSENVTLMNLRPKTGYSVRVQLSRPGEGGEGAWGPPTLMTTDC

PEPLLKPWLEGWHVEGPDRLRVSWSLPPVPGPLVGDGFLLRLWDGARGQERRENVSSPQA

RTALLTGLTPGTYYQLDVRLYHCTLLGPASPAARVLLPPSGPPAPRHLHAQALSDSEIQL

MWQRPEAAAGPISKYIVEVQVAGGSGDPLWMDVDRPEETSTIVRGLNASTRYLFRVRASV

QGPGDWSNVVEQSTLGNGLQIEGPVQEIHAAEEGLDQQLVLAVVGSVSATCLTILAALLT

LACIRKSCLHRRRTFTYQSGSGEETILQFSSGTLTLTRRPKPQPEPLNYPVLEWEDITFE

DLIGEGNFGQVIRAMIKKDGLKMNAAIKMLKEYASENDHRDFAGELEVLCKLGHHPNIIN

LLGACENRGYLYIAIEYAPYGNLLDFLRKSRVLETDPAFAREHGTASTLSSRQLLRFASD

AANGMQYLSEKQFIHRDLAARNVLVGENLASKIADFGLSRGEEVYVKKTMGRLPVRWMAI

ESLNYSVYTTKSDVWSFGVLLWEIVSLGGTPYCGMTCAELYEKLPQAYRMEQPRNCDDEV

YELMRQCWRDRPYERPPFAQIALQLGRMLEARKAYVNMSLFENFTYAGIDATAEEA

>sp|P31754|UMPS_BOVIN Uridine 5'-monophosphate synthase OS=Bos taurus OX=9913 GN=UMPS PE=2 SV=1

MAAADALLGSLVTGLYDVQAFKFGNFVLKSGLSSPVYIDLRGIISRPSILNQVAEMLFQT

AENAEINFDTVCGVPYTALPLATIVCSTHEIPMLIRRKEKKDYGTKRLIEGAVNPGDTCL

IIEDVVSSGSSVWETAEVLQKEGLKVTDAVVLVDREQGGRDNLQARGIRLHSVCTLSTVL

CILEQQKKINAETVERVKRFIQENAFVAANPNDSLPSVKKEPKELSFGARAELPGTHPVA

AKLLRLMQKKETNLCLSADVSESRELLQLADALGSRICLLKIHVDILNDFTLDVMKELTT

LAKRHEFLIFEDRKFADIGNTVKKQYEGGVFKIASWADLVNAHAVPGSGVVKGLEEVGLP

LHRACLLVAEMSSAGTLATGSYTEAAVQMAEEHSEFVIGFISGSRVSMKPEFLHLTPGVQ

LEAGGDNLGQQYHSPQEVIGKRGSDIIIVGRGIIASANQLEAAKMYRKAAWEAYLSRLAV

>sp|Q3SYZ6|XYLB_BOVIN Xylulose kinase OS=Bos taurus OX=9913 GN=XYLB PE=2 SV=1

MAERAARHCCLGWDFSTQQVKVVAVDAELSVFYEDSVHFDRDLVEFGTQGGVHVHKDGLT

VTSPVLMWVQALDIILEKMKASGFDFSQVLALSGAGQQHGSVYWKTGASQVLTSLSPDLP

LREQLQACFSISNCPVWMDSSTAAQCRQLEAAVGGAQALSLLTGSRAYERFTGNQIAKIY

QQNPEAYSHTERISLVSSFAASLFLGSYSPVDYSDGSGMNLLQIQDKVWSQACLGACAPR

LEEKLGRPVPSCSIVGAISSYFVQRYGFPPECKVVAFTGDNPASLAGMRLEEGDIAVSLG

TSDTLFLWLQEPTPALEGHIFCNPVDPQHYMALLCFKNGSLMREKIRDESASGSWSKFSK

ALQSTGMGNSGNLGFYFDVMEITPEIIGRHRFTAENHEVSAFPQDVEIRALIEGQFMAKK

IHAEALGYRVMPKTKILATGGASHNRDILQVLADVFGAPVYVIDTANSACVGSAYRAFHG

PSLLCLVSIY

>sp|P68252|1433G_BOVIN 14-3-3 protein gamma OS=Bos taurus OX=9913 GN=YWHAG PE=1 SV=2

MVDREQLVQKARLAEQAERYDDMAAAMKNVTELNEPLSNEERNLLSVAYKNVVGARRSSW

RVISSIEQKTSADGNEKKIEMVRAYREKIEKELEAVCQDVLSLLDNYLIKNCSETQIESK

VFYLKMKGDYYRYLAEVATGEKRATVVESSEKAYSEAHEISKEHMQPTHPIRLGLALNYS

VFYYEIQNAPEQACHLAKTAFDDAIAELDTLNEDSYKDSTLIMQLLRDNLTLWTSDQQDD

DGGEGNN

>sp|Q8SQA4|AGRE5_BOVIN Adhesion G protein-coupled receptor E5 OS=Bos taurus OX=9913 GN=ADGRE5 PE=2 SV=1

MGGPHGGPFLLFHVLCFLLTLSEVGSQNSKACALPCPPNSSCVNGTACRCAPGFISFSGE

IFTDPLESCDDINECGPPSPVDCGSSADCQNTEGGYYCTCSPGYEPVSGAMIFRNESENT

CRDVDECSSGQHQCHNSTVCFNTVGSYTCHCREGWEPKHGLKNKQKDTICKEISFPAWTA

PPGIKSRSLSAFFERVQKMSRDFKPAMAKKSMQDLVGSVDDLLKNSGDLESLDQSSKHVT

VTHLLSGLEQILRTLAKAMPKGSFTYRSLDNTELSLVVQEQGKGNVTVGQSHARMLLDWA

VAAAAEESGPTVVGILSSQNMKKLLANASLKLDSEKLKETYKSPVRGAKVTLLSAVSSVF

LSNTNTEKLDSNVSFAFALHEQPELKPRQELICAFWKKDSNGNGSWATTGCWKMGRGNGS

ITCQCSHLSSFAILMAHYDVEDPKLALITKVGLALSLACLLLCILTFLLVRPIQGSRTTV

HLHLCICLFVGSAIFLAGIENEGGEVGTRCRLVAVLLHYCFLAAFCWMSLEGVELYFLVV

RVFQGQGMRKLWLCLIGYGVPLIIVGISAGAYSKGYGREKFCWLNFEGGFLWSFVGPVTF

IVLGNAIIFVITVWKLTQKFSEINPDIKKLKKARVLTITAIAQLFVLGCTWVFGLLLFNP

ESWVLSYIFSILNCLQGFFLFVLYCLLNKKVREEYRKWACMVAGNKYSEFATTTSGSGSS

HNQTQALRPSESGM

>sp|Q58CQ2|ARC1B_BOVIN Actin-related protein 2/3 complex subunit 1B OS=Bos taurus OX=9913 GN=ARPC1B PE=1 SV=4

MAYHSFLVEPISCHAWNKDRTQIAICPNNHEVHIYEKSGNKWVQVHELKEHNGQVTGIDW

APDSNRIVTCGTDRNAYVWTLKGRTWKPTLVILRINRAARCVRWAPNEKKFAVGSGSRVI

SICYFEQENDWWVCKHIKKPIRSTVLSLDWHPNSVLLAAGSCDFKCRIFSAYIKEVEERP

APTPWGSKMPFGELMFESSSSCGWVHGVCFSANGSRVAWVSHDSTVCLADADKKMAVATL

ASETLPLLAVTFITESSLVAAGHDCFPVLFTYDSAAGKLSFGGRLDVPKQSSQRGLTARE

RFQNLDKKASSEGSAAAGAGLDSLHKNSVSQISVLSGGKAKCSQFCTTGMDGGMSIWDVR

SLESALKDLKIV

>sp|P08037|B4GT1_BOVIN Beta-1,4-galactosyltransferase 1 OS=Bos taurus OX=9913 GN=B4GALT1 PE=1 SV=3

MKFREPLLGGSAAMPGASLQRACRLLVAVCALHLGVTLVYYLAGRDLRRLPQLVGVHPPL

QGSSHGAAAIGQPSGELRLRGVAPPPPLQNSSKPRSRAPSNLDAYSHPGPGPGPGSNLTS

APVPSTTTRSLTACPEESPLLVGPMLIEFNIPVDLKLVEQQNPKVKLGGRYTPMDCISPH

KVAIIIPFRNRQEHLKYWLYYLHPILQRQQLDYGIYVINQAGESMFNRAKLLNVGFKEAL

KDYDYNCFVFSDVDLIPMNDHNTYRCFSQPRHISVAMDKFGFSLPYVQYFGGVSALSKQQ

FLSINGFPNNYWGWGGEDDDIYNRLAFRGMSVSRPNAVIGKCRMIRHSRDKKNEPNPQRF

DRIAHTKETMLSDGLNSLTYMVLEVQRYPLYTKITVDIGTPS

>sp|P21809|PGS1_BOVIN Biglycan OS=Bos taurus OX=9913 GN=BGN PE=1 SV=3

MWPLWPLAALLALSQALPFEQKAFWDFTLDDGLPMLNDEEASGAETTSGIPDLDSLPPTY

SAMCPFGCHCHLRVVQCSDLGLKAVPKEISPDTTLLDLQNNDISELRKDDFKGLQHLYAL

VLVNNKISKIHEKAFSPLRKLQKLYISKNHLVEIPPNLPSSLVELRIHDNRIRKVPKGVF

SGLRNMNCIEMGGNPLENSGFEPGAFDGLKLNYLRISEAKLTGIPKDLPETLNELHLDHN

KIQAIELEDLLRYSKLYRLGLGHNQIRMIENGSLSFLPTLRELHLDNNKLSRVPAGLPDL

KLLQVVYLHTNNITKVGVNDFCPVGFGVKRAYYNGISLFNNPVPYWEVQPATFRCVTDRL

AIQFGNYKK

>sp|A4FUA8|CAZA1_BOVIN F-actin-capping protein subunit alpha-1 OS=Bos taurus OX=9913 GN=CAPZA1 PE=2 SV=1

MADFEDRVSDEEKVRIAAKFITHAPPGEFNEVFNDVRLLLNNDNLLREGAAHAFAQYNMD

QFTPVKIEGYEDQVLITEHGDLGNSRFLDPRNKISFKFDHLRKEASDPQPEEADGGLKSW

RESCDSALRAYVKDHYSNGFCTVYAKTIDGQQTIIACIESHQFQPKNFWNGRWRSEWKFT

ITPPTAQVVGVLKIQVHYYEDGNVQLVSHKDVQDSVTVSNEAQTAKEFIKIIEHAENEYQ

TAISENYQTMSDTTFKALRRQLPVTRTKVDWNKILSYKIGKEMQNA

>sp|P04258|CO3A1_BOVIN Collagen alpha-1(III) chain OS=Bos taurus OX=9913 GN=COL3A1 PE=1 SV=1

EYEAYDVKSGVAGGGIAGYPGPAGPPGPPGPPGTSGHPGAPGAPGYQGPPGEPGQAGPAG

PPGPPGAIGPSGKDGESGRPGRPGPRGFPGPPGMKGPAGMPGFPGMKGHRGFDGRNGEKG

EPGAPGLKGENGVPGEDGAPGPMGPRGAPGERGRPGLPGAAGARGNDGARGSDGQPGPPG

PPGTAGFPGSPGAKGEVGPAGSPGSSGAPGQRGEPGPQGHAGAPGPPGPPGSDGSPGGKG

EMGPAGIPGAPGLIGARGPPGPPGTNGVPGQRGAAGEPGKNGAKGDPGPRGERGEAGSPG

IAGPKGEDGKDGSPGEPGANGLPGAAGERGVPGFRGPAGANGLPGEKGPPGDRGGPGPAG

PRGVAGEPGRNGLPGGPGLRGIPGSPGGPGSNGKPGPPGSQGETGRPGPPGSPGPRGQPG

VMGFPGPKGNDGAPGKNGERGGPGGPGPQGPAGKNGETGPQGPPGPTGPSGDKGDTGPPG

PQGLQGLPGTSGPPGENGKPGEPGPKGEAGAPGIPGGKGDSGAPGERGPPGAGGPPGPRG

GAGPPGPEGGKGAAGPPGPPGSAGTPGLQGMPGERGGPGGPGPKGDKGEPGSSGVDGAPG

KDGPRGPTGPIGPPGPAGQPGDKGESGAPGVPGIAGPRGGPGERGEQGPPGPAGFPGAPG

QNGEPGAKGERGAPGEKGEGGPPGAAGPAGGSGPAGPPGPQGVKGERGSPGGPGAAGFPG

GRGPPGPPGSNGNPGPPGSSGAPGKDGPPGPPGSNGAPGSPGISGPKGDSGPPGERGAPG

PQGPPGAPGPLGIAGLTGARGLAGPPGMPGARGSPGPQGIKGENGKPGPSGQNGERGPPG

PQGLPGLAGTAGEPGRDGNPGSDGLPGRDGAPGAKGDRGENGSPGAPGAPGHPGPPGPVG

PAGKSGDRGETGPAGPSGAPGPAGSRGPPGPQGPRGDKGETGERGAMGIKGHRGFPGNPG

APGSPGPAGHQGAVGSPGPAGPRGPVGPSGPPGKDGASGHPGPIGPPGPRGNRGERGSEG

SPGHPGQPGPPGPPGAPGPCCGAGGVAAI

>sp|Q2TBN5|COMD9_BOVIN COMM domain-containing protein 9 OS=Bos taurus OX=9913 GN=COMMD9 PE=2 SV=1

MAALTAENFAALQSLLKASSKDVVRQLCQESFSSSALGSKNLLDVTCSSLSVTQEEAEQL

LQALHRLTRLAVFRDLSSAEAILALFPENFHQNLKNLLTKIILEHVSAWRAEAQVNQISL

PRLVDLDWRVDIKTSSDSISRMAVPTCLLQMKIQEDPSLCGDRPSVSDVTVELSKETLDT

MLDGLGRIRDQLSAVASK

>sp|P00794|CHYM_BOVIN Chymosin OS=Bos taurus OX=9913 GN=CYM PE=1 SV=3

MRCLVVLLAVFALSQGAEITRIPLYKGKSLRKALKEHGLLEDFLQKQQYGISSKYSGFGE

VASVPLTNYLDSQYFGKIYLGTPPQEFTVLFDTGSSDFWVPSIYCKSNACKNHQRFDPRK

SSTFQNLGKPLSIHYGTGSMQGILGYDTVTVSNIVDIQQTVGLSTQEPGDVFTYAEFDGI

LGMAYPSLASEYSIPVFDNMMNRHLVAQDLFSVYMDRNGQESMLTLGAIDPSYYTGSLHW

VPVTVQQYWQFTVDSVTISGVVVACEGGCQAILDTGTSKLVGPSSDILNIQQAIGATQNQ

YGEFDIDCDNLSYMPTVVFEINGKMYPLTPSAYTSQDQGFCTSGFQSENHSQKWILGDVF

IREYYSVFDRANNLVGLAKAI

>sp|P00376|DYR_BOVIN Dihydrofolate reductase OS=Bos taurus OX=9913 GN=DHFR PE=1 SV=3

MVRPLNCIVAVSQNMGIGKNGDLPWPPLRNEFQYFQRMTTVSSVEGKQNLVIMGRKTWFS

IPEKNRPLKDRINIVLSRELKEPPKGAHFLAKSLDDALELIEDPELTNKVDVVWIVGGSS

VYKEAMNKPGHVRLFVTRIMQEFESDAFFPEIDFEKYKLLPEYPGVPLDVQEEKGIKYKF

EVYEKNN

>sp|O02675|DPYL2_BOVIN Dihydropyrimidinase-related protein 2 OS=Bos taurus OX=9913 GN=DPYSL2 PE=1 SV=1

MSYQGKKNIPRITSDRLLIKGGKIVNDDQSFYADIYMEDGLIKQIGENLIVPGGVKTIEA

HSRMVIPGGIDVHTRFQMPDQGMTSADDFFQGTKAALAGGTTMIIDHVVPEPGTSLLAAF

DQWREWADSKSCCDYSLHVDITEWHKGVQEEMEALVKDHGVNSFLVYMAFKDRFQLTDSQ

IYEVLSVIRDIGAIAQVHAENGDIIAEEQQRILDLGITGPEGHVLSRPEEVEAEAVNRSI

TIANQTNCPLYITKVMSKSAAEVIAQARKKGTVVYGEPITASLGTDGSHYWSKNWAKAAA

FVTSPPLSPDPTTPDFLNSLLSCGDLQVTGSAHCTFNTAQKAVGKDNFTLIPEGTNGTEE

RMSVIWDKAVVTGKMDENQFVAVTSTNAAKVFNLYPRKGRIAVGSDADLVIWDPDSVKTI

SAKTHNSSLEYNIFEGMECRGSPLVVISQGKIVLEDGTLHVTEGSGRYIPRKPFPDFVYK

RIKARSRLAELRGVPRGLYDGPVCEVSVTPKTVTPASSAKTSPAKQQAPPVRNLHQSGFS

LSGAQIDDNIPRRTTQRIVAPPGGRANITSLG

>sp|Q2NL22|IF4A3_BOVIN Eukaryotic initiation factor 4A-III OS=Bos taurus OX=9913 GN=EIF4A3 PE=2 SV=3

MAATATMATSGSARKRLLKEEDMTKVEFETSEEVDVTPTFDTMGLREDLLRGIYAYGFEK

PSAIQQRAIKQIIKGRDVIAQSQSGTGKTATFSISVLQCLDIQVRETQALILAPTRELAV

QIQKGLLALGDYMNVQCHACIGGTNVGEDIRKLDYGQHVVAGTPGRVFDMIRRRSLRTRA

IKMLVLDEADEMLNKGFKEQIYDVYRYLPPATQVVLISATLPHEILEMTNKFMTDPIRIL

VKRDELTLEGIKQFFVAVEREEWKFDTLCDLYDTLTITQAVIFCNTKRKVDWLTEKMREA

NFTVSSMHGDMPQKERESIMKEFRSGASRVLISTDVWARGLDVPQVSLIINYDLPNNREL

YIHRIGRSGRYGRKGVAINFVKNDDIRILRDIEQYYSTQIDEMPMNVADLI

>sp|Q6EWQ7|IF5A1_BOVIN Eukaryotic translation initiation factor 5A-1 OS=Bos taurus OX=9913 GN=EIF5A PE=2 SV=3

MADDLDFETGDAGASATFPMQCSALRKNGFVVLKGRPCKIVEMSTSKTGKHGHAKVHLVG

IDIFTGKKYEDICPSTHNMDVPNIKRNDFQLIGIQDGYLSLLQDSGEVREDLRLPEGDLG

KEIEQKYDCGEEILITVLSAMTEEAAVAIKAMAK

>sp|P22457|FA7_BOVIN Coagulation factor VII OS=Bos taurus OX=9913 GN=F7 PE=1 SV=2

MLSQAWALALLCFLLSLWGSLPAVFLPQEQALSILHRPRRANGFLEELLPGSLERECREE

LCSFEEAHEIFRNEERTRQFWVSYNDGDQCASSPCQNGGSCEDQLRSYICFCPDGFEGRN

CETDKQSQLICANDNGGCEQYCGADPGAGRFCWCHEGYALQADGVSCAPTVEYPCGKIPV

LEKRNGSKPQGRIVGGHVCPKGECPWQAMLKLNGALLCGGTLVGPAWVVSAAHCFERLRS

RGNLTAVLGEHDLSRVEGPEQERRVAQIIVPKQYVPGQTDHDVALLQLAQPVALGDHVAP

LCLPDPDFADQTLAFVRFSAVSGWGQLLERGVTARKLMVVLVPRLLTQDCLQQSRQRPGG

PVVTDNMFCAGYSDGSKDACKGDSGGPHATRFRGTWFLTGVVSWGEGCAAAGHFGIYTRV

SRYTAWLRQLMGHPPSRQGFFQVPLLP

>sp|Q2TBQ3|GAMT_BOVIN Guanidinoacetate N-methyltransferase OS=Bos taurus OX=9913 GN=GAMT PE=2 SV=1

MSAPAATPIFAPGENCSPAWRAAPAAYDASDTHLQILGKPVMERWETPYMHALAAAAASR

GGRVLEVGFGMAIAATKVQEAPIEEHWIIECNEGVFQRLQDWALQQPHKVVPLKGLWEEV

APTLPDSHFDGILYDTYPLSEETWHTHQFNFIRDHAFRLLKPGGVLTYCNLTSWGELMKT

KYSDITTMFEETQVPALLEAGFRRDNIRTQVMELVPPANCRYYAFPRMITPLVTKH

>sp|P11017|GBB2_BOVIN Guanine nucleotide-binding protein G(I)/G(S)/G(T) subunit beta-2 OS=Bos taurus OX=9913 GN=GNB2 PE=2 SV=3

MSELEQLRQEAEQLRNQIRDARKACGDSTLTQITAGLDPVGRIQMRTRRTLRGHLAKIYA

MHWGTDSRLLVSASQDGKLIIWDSYTTNKVHAIPLRSSWVMTCAYAPSGNFVACGGLDNI

CSIYSLKTREGNVRVSRELPGHTGYLSCCRFLDDNQIITSSGDTTCALWDIETGQQTVGF

AGHSGDVMSLSLAPDGRTFVSGACDASIKLWDVRDSMCRQTFIGHESDINAVAFFPNGYA

FTTGSDDATCRLFDLRADQELLMYSHDNIICGITSVAFSRSGRLLLAGYDDFNCNIWDAM

KGDRAGVLAGHDNRVSCLGVTDDGMAVATGSWDSFLKIWN

>sp|A7YWP4|HUTH_BOVIN Histidine ammonia-lyase OS=Bos taurus OX=9913 GN=HAL PE=2 SV=1

MPRYTVHVRGEWLAVPCQDAQLTVGWLGREAVRRYIKNKPDNGGFASVDDARFLVRRCKG

LGLLDNEDPLDVALEDNEFVEVVIEGDAMSPDFIPSQPEGVYLYSKYREPEKYIALDGDS

LTTEDLVSLGKGHYKIKLTPTAEKRVQKSREVIDRIVEEKTVVYGITTGFGKFARTVIPV

SKLEELQFNLVRSHSSGVGKPLSPERCRMLLALRINVLAKGYSGISLGTLKQVIEVFNAS

CLPYVPEKGTVGASGDLAPLSHLALGLIGEGKMWSPKSGWADAKYVLAAHGLKPIVLKPK

EGLALINGTQMITSLGCEAVERASAIARQADIVAALTLEVLKGTTKAFDTDIHAVRPHRG

QVEVAFRFRSLLDSDHHPSEIAESHRFCDRVQDAYTLRCCPQVHGVVNDTIAFVKNIITT

EINSATDNPMVFASRGETISGGNFHGEYPAKALDYLAIGVHELASISERRIERLCNPSLS

ELPAFLVAEGGLNSGFMIAHCTAAALVSENKALCHPSSVDSLSTSAATEDHVSMGGWAAR

KALRVIEHVEQVLAIELLAACQGIEFLRPLKTTTPLEKVYDLVRSVVRPWIKDRFMAPDI

EAAHRLLVEQKVWEVAAPYIEKYRMEHIPESRPVSPTAFSLEFLHKKSTKIPESEDL

>sp|Q5EA20|HPPD_BOVIN 4-hydroxyphenylpyruvate dioxygenase OS=Bos taurus OX=9913 GN=HPD PE=2 SV=3

MTTYSDKGEKPERGRFLHFHSVTFWVGNAKQAASYYCSKLGFEPLAYKGLETGSREVVSH

VVKQGQIVFVFSSALNPWNKEMGDHLVKHGDGVKDIAFEVEDCDYIVQKARERGAKIVRE

PWVEQDKLGKVKFAVLQTYGDTTHTLVEKMNYTGRFLPGFEAPPFMDPQLSKLPSCSLEI

IDHIVGNQPDQEMVSASEWYLKNLQFHRFWSVDDTQVHTEYSSLRSVVVANYEESIKMPI

NEPAPGKKKSQIQEYVDYNGGAGVQHIALKTKDIITAIRHLRERGVEFLAVPSTYYKQLR

EKLKMAKIRVKENIDILEELKILVDYDEKGYLLQIFTKPMQDRPTLFLEVIQRHNHQGFG

AGNFNSLFKAFEEEQDLRGNLTDMEPNGVVSGM

>sp|P0CB32|HS71L_BOVIN Heat shock 70 kDa protein 1-like OS=Bos taurus OX=9913 GN=HSPA1L PE=3 SV=1

MAAAKGTAIGIDLGTTYSCVGVFQHGKVEIIANDQGNRTTPSYVAFTDTERLIGDAAKNQ

VAMNPQNTVFDAKRLIGRKFNDPVVQSDMKLWPFQVINEGGKPKVMVSYKGEKKAFYPEE

ISSMVLTKMKETAEAFLGYTVTNAVITVPAYFNDSQRQATKDAGVIAGLNVLRIINEPTA

AAIAYGLDKAGQGERHVLIFDLGGGTFDVSVLTIDDGIFEVKATAGDTHLGGEDFDNRLV

SHFVEEFKRKHKKDISQNKRAVRRLRTACERAKRTLSSSTQANLEIDSLYEGIDFYTSIT

RARFEELCADLFRGTLEPVEKALRDAKMDKAKIHDIVLVGGSTRIPKVQRLLQDYFNGRD

LNKSINPDEAVAYGAAVQAAILMGDKSEKVQDLLLLDVAPLSLGLETAGGVMTVLIKRNS

TIPTKQTQIFTTYSDNQPGVLIQVYEGERAMTRDNNLLGRFDLTGIPPAPRGVPQIEVTF

DIDANGILNVTAMDKSTGKANKITITNDKGRLSKEEIERMVLDAEKYKAEDEVQREKIAA

KNALESYAFNMKSAVSDEGLQGKISESDKKKILSKCNEVLLWLEANQLAEKDEFDHKRKE

LEQVCNPIITKLYQGGCTGPSCGTGYTPGRAATGPTIEEVD

>sp|Q9XSG3|IDHC_BOVIN Isocitrate dehydrogenase [NADP] cytoplasmic OS=Bos taurus OX=9913 GN=IDH1 PE=2 SV=1

MSQKIQGGSVVEMQGDEMTRIIWELIKEKLIFPYVELDLHSYDLGIENRDATNDQVTKDA

AEAIKKYNVGVKCATITPDEKRVEEFKLKQMWKSPNGTIRNILGGTVFREAIICKNIPRL

VSGWVKPIIIGRHAYGDQYRATDFVVPGPGKVEISYTPSDGSPKTVYLVHNFTESGGVAM

GMYNQDKSIEDFAHSSFQMALSKNWPLYLSTKNTILKKYDGRFKDIFQEIYDKQYKSEFE

AQNIWYEHRLIDDMVAQAMKSEGGFIWACKNYDGDVQSDSVAQGYGSLGMMTSVLVCPDG

KTVEAEAAHGTVTRHYRMYQKGQETLTNPIASIFAWTRGLAHRAKLDNNKELSFFAKALE

EVCIETIEAGFMTKDLAACIKGLPNVQRSDYLNTFEFMDKLGENLQLKLAQAKL

>sp|P08728|K1C19_BOVIN Keratin, type I cytoskeletal 19 OS=Bos taurus OX=9913 GN=KRT19 PE=2 SV=1

MTSYSYRQSSSTSSFGGMGGGSMRFGAGGAFRAPSIHGGSGGRGVSVSSARFVSSSSGGY

GGGYGGALATSDGLLAGNEKLTMQNLNDRLASYLEKVRALEEANGDLEVKIRDWYQKQGP

GPARDYSHYFKTIEDLRDQILGATIENSKIVLQIDNARLAADDFRTKFETEQALRMSVEA

DINGLRRVLDELTLARTDLEMQIEGLKEELAYLKKNHEEEMSVLKGQVGGQVSVEVDSAP

GIDLAKILSDMRSQYEVIAEKNRKDAEAWFISQTEELNREVAGHTEQLQISKTEVTDLRR

TLQGLEIELQSQLSMKAALEGTLAETEARFGAQLAQIQALISGIEAQLSDVRADTERQNQ

EYQHLMDIKTRLEQEIATYRNLLEGQDAYFNDLSLAKAL

>sp|Q29451|MA2B1_BOVIN Lysosomal alpha-mannosidase OS=Bos taurus OX=9913 GN=MAN2B1 PE=1 SV=4

MVGDARPSGVRAGGCRGAVGSRTSSRALRPPLPPLSSLFVLFLAAPCAWAAGYKTCPKVK

PDMLNVHLVPHTHDDVGWLKTVDQYFYGIYNNIQPAGVQYILDSVISSLLANPTRRFIYV

EIAFFSRWWRQQTNATQKIVRELVRQGRLEFANGGWVMNDEATTHYGAIIDQMTLGLRFL

EETFGSDGRPRVAWHIDPFGHSREQASLFAQMGFDGFFFGRLDYQDKKVRKKTLQMEQVW

RASTSLKPPTADLFTSVLPNMYNPPEGLCWDMLCADKPVVEDTRSPEYNAKELVRYFLKL

ATDQGKLYRTKHTVMTMGSDFQYENANTWFKNLDKLIQLVNAQQRANGIRVNVLYSTPAC

YLWELNKANLSWSVKKDDFFPYADGPYMFWTGYFSSRPALKRYERLSYNFLQVCNQLEAL

AGPAANVGPYGSGDSAPLNEAMAVLQHHDAVSGTSRQHVANDYARQLSEGWRPCEVLMSN

ALAHLSGLKEDFAFCRKLNISICPLTQTAERFQVIVYNPLGRKVDWMVRLPVSKHVYLVK

DPGGKIVPSDVVTIPSSDSQELLFSALVPAVGFSIYSVSQMPNQRPQKSWSRDLVIQNEY

LRARFDPNTGLLMELENLEQNLLLPVRQAFYWYNASTGNNLSSQASGAYIFRPNQNKPLF

VSHWAQTHLVKASLVQEVHQNFSAWCSQVVRLYPRQRHLELEWTVGPIPVGDGWGKEVIS

RFDTALATRGLFYTDSNGREILERRRNYRPTWKLNQTEPVAGNYYPVNSRIYITDGNMQL

TVLTDRSQGGSSLRDGSLELMVHRRLLKDDARGVGEPLNKEGSGLWVRGRHLVLLDKKET

AAARHRLQAEMEVLAPQVVLAQGGGARYRLEKAPRTQFSGLRRELPPSVRLLTLARWGPE

TLLLRLEHQFAVGEDSGRNLSSPVTLDLTNLFSAFTITNLRETTLAANQLLAYASRLQWT

TDTGPTPHPSPSRPVSATITLQPMEIRTFLASVQWEEDG

>sp|P46196|MK01_BOVIN Mitogen-activated protein kinase 1 OS=Bos taurus OX=9913 GN=MAPK1 PE=2 SV=3

MAAAAAAGAGPEMVRGQVFDVGPRYTNLSYIGEGAYGMVCSAYDNVNKVRVAIKKISPFE

HQTYCQRTLREIKILLRFRHENIIGINDIIRAPTIEQMKDVYIVQDLMETDLYKLLKTQH

LSNDHICYFLYQILRGLKYIHSANVLHRDLKPSNLLLNTTCDLKICDFGLARVADPDHDH

TGFLTEYVATRWYRAPEIMLNSKGYTKSIDIWSVGCILAEMLSNRPIFPGKHYLDQLNHI

LGILGSPSQEDLNCIINLKARNYLLSLPHKNKVPWNRLFPNADSKALDLLDKMLTFNPHK

RIEVEQALAHPYLEQYYDPSDEPVAEAPFKFDMELDDLPKEKLKELIFEETARFQPGYRS

>sp|Q1JPA6|ARY1_BOVIN Arylamine N-acetyltransferase 1 OS=Bos taurus OX=9913 GN=NAT1 PE=2 SV=1

MDIDAYFERIGYKNSRDKLDLETLTDILQHQIRAIPFENLNIHCGEAMELDLEVIFDQIV

RRKRGGWCLQVNHLLYWALTMIGFETTILGGYVYNTFNDKYSSAMIHLLLKVTIDGRDYI

ADAGFGRSYQMWQPLELISGKYQPQTPCIFRLTEDRGTWYLDQIRREQYIPNQDFLDSDL

LEKNEYRKIYSFTLEPRTIKDFESVNTYLQESPASVFTSKSFCSLQTPEGVHCLVGFTLT

YRRFNYKDNTDLVEFKTLNEKEIEENLKNIFNISLEKKLTPKHGDKFFTI

>sp|Q0VCN1|NMRL1_BOVIN NmrA-like family domain-containing protein 1 OS=Bos taurus OX=9913 GN=NMRAL1 PE=2 SV=1

MADKKLVVVFGATGAQGGSVARTLLEDGTFRVRVVTRDPGQRAAKQLRLQGAEVVQGDQD

DEASMELALSGAHATFIVTNYWENCSQEQEVKQGKLLADLAKRLGLRYVVYSGLENIKKL

TAGRLTVGHFDGKGEVEEYFRDIGVPMTSVRLPCYFENLLSYFLPQKAPDGRSYLLSLPM

GDVPIDGMSVADLGPVVLSLLKTPEEYVGRNIGLSTCRHTVEEYAALLTKHTGKAVRDAK

TSPEDYEKLGFPGAQDLANMFRFYALKPDRNIELTLKLNPKARRLDQWLEQHKEDFAGL

>sp|Q29RU2|OIT3_BOVIN Oncoprotein-induced transcript 3 protein OS=Bos taurus OX=9913 GN=OIT3 PE=2 SV=1

MPQLLLLACLLIIVTRVAPRALDPCSAYISLNEPWRNTEHQFDESRGSPLCDNSVDGEWY

RFTGMAGDAMPTFCIPENHCGTHAPVWLNGSHPLEGDGIVQRQACASFNGNCCLWNTTVE

VKSCPGGYYVYRLTKPSVCFHVYCGHFYDICDDDCHGSCLGTSECTCAPGTVLGPDRQTC

FDENECEQNNGGCSEICVNLKNSYRCECGIGRVLRSDGKTCEDIEGCHNNNGGCSHSCLT

SETGYQCECPRGLVLSEDNHTCQVPVFCKSNTIEVSIPRDLVGGLELFLTNTSCRGVSNG

THVNILFSLKTCGTVVDVVNDKIVASNLVTGLPKQTPGSSGDIIIRTSKLLIPVTCEFPR

LYTISEGYVPNLRNTPLEIMSRSHGIFPFTLEIFKDHEFEEPYREALPTLKLRDSLYFGI

EPLVHVNGLESLVESCFATPTSKIDEIMKYYIIQDGCVSDDSVKQYTSRDHLAKHFQVPV

FKFVGKDHKEVFLHCRVLVCGMLDERSRCAQGCHRRVRREASTEGEDASGPRSQMLTGGP

ISIDWED

>sp|Q0VCP3|OLFL3_BOVIN Olfactomedin-like protein 3 OS=Bos taurus OX=9913 GN=OLFML3 PE=2 SV=1

MGPHTQLLILLLLSWLGPLQGQQHHLVEYMERRLAALEERLAQCQDQSSRHAAELRDFKN

KMLPLLEVAEKEREALRTEADTISGRVDRLEREVDYLETQNPALPCVEVDEKVTGGPGTK

GKGRRNEKYDMITDCGYTISQVRSMKILKRFGGPAGLWTKDPLGPAEKIYVLDGTQNDTA

FVFPRLRDFTLAMAARKASRVRVPFPWVGTGQLVYGGFLYYARRPPGGPGGGGELQNTLQ

LIKFHLANRTVVDSSVFPAEGLIPPYGLTADTYIDLAADEEGLWAVYATREDDRHLCLAK

LDPQTLDTEQQWDTPCPRENAEAAFVICGTLYVVYNTRPASRARIQCSFDASGTLTPERA

ALPYFPRRYGAHASLRYNPRERQLYAWDDGYQIVYKLEMRKKEEEV

>sp|O77742|OMD_BOVIN Osteomodulin OS=Bos taurus OX=9913 GN=OMD PE=1 SV=1

MGFSSLVCVLFFFLGVKVYCQYESYQWDEDYDQEPDDVYQTEFQFQQNINYEAPFHQHTL

GCASECFCPPNFPSSMYCDNRKLKTIPNIPAHIQQVYLQFNEIEAVTADSFINATHLKEI

NLSHNKIKSQKIDHGVFATLPNLLQLHLQHNNLEDFPFPLPKSLERIFLGYNEISRLQTN

AVNGLVNLTMLDLCFNKIDDSVLQEKVLAKMEKLMQLNLCNNRLESMPPGLPSSLMYLSL

ENNSISSIPENYFNKLPKLHALRISHNKLQDIPYNIFNLSNLIELNVGHNKLKQAFYIPR

NLEHLYLENNEIENVNVTVMCPSVDPLHYHHLTHIRIDQNKLKAPISSYIFLCFPHIHTI

YYGEQQSTNGQTIQLKTQVFRRFQDDGDSEDHDDHHEGPEEEGTEENIDAHYYGSQEWQE

TI

>sp|P68401|PA1B2_BOVIN Platelet-activating factor acetylhydrolase IB subunit alpha2 OS=Bos taurus OX=9913 GN=PAFAH1B2P68402 PE=1 SV=1

MSQGDSNPAAIPHAAEDIQGDDRWMSQHNRFVLDCKDKEPDVLFVGDSMVQLMQQYEIWR

ELFSPLHALNFGIGGDTTRHVLWRLKNGELENIKPKVIVVWVGTNNHENTAEEVAGGIEA

IVQLINTRQPQAKIIVLGLLPRGEKPNPLRQKNAKVNQLLKVSLPKLANVQLLDTDGGFV

HSDGAISCHDMFDFLHLTGGGYAKICKPLHELIMQLLEETPEEKQTTIA

>sp|Q2KIH7|PH4H_BOVIN Phenylalanine-4-hydroxylase OS=Bos taurus OX=9913 GN=PAH PE=2 SV=1

MSALVLESRALGRKLSDFGQETSYIEGNSDQNAVSLIFSLKEEVGALARVLRLFEENDIN

LTHIESRPSRLRKDEYEFFTNLDQRSVPALANIIKILRHDIGATVHELSRDKKKDTVPWF

PRTIQELDNFANQVLSYGAELDADHPGFKDPVYRARRKQFADIAYNYRHGQPIPRVEYTE

EEKKTWGTVFRTLKSLYKTHACYEHNHIFPLLEKYCGFREDNIPQLEEVSQFLQSCTGFR

LRPVAGLLSSRDFLGGLAFRVFHCTQYIRHGSKPMYTPEPDICHELLGHVPLFSDRSFAQ

FSQEIGLASLGAPDEYIEKLATIYWFTVEFGLCKQGDSIKAYGAGLLSSFGELQYCLSDK

PKLLPLELEKTAVQEYTITEFQPLYYVAESFNDAKEKVRNFAATIPRPFSVHYDPYTQRI

EVLDNTQQLKILADSISSEVEILCSALQKLK

>sp|Q3T0W4|PP1R7_BOVIN Protein phosphatase 1 regulatory subunit 7 OS=Bos taurus OX=9913 GN=PPP1R7 PE=1 SV=1

MAAERGAGQQQSQEMMEVDRRVESEESGDEEGKKQNSGMVADLSAHSLKDGEERGDEDPE

EGQELPVDMETISLDRDAEDVDLNHYRIGKIEGFEVLKKVKTLCLRQNLIKCIENLEGLQ

SLRELDLYDNQIRRIENLDALTELEVLDISFNLLRNIEGIDKLTRLKKLFLVNNKINKIE

NISSLHQLQMLELGSNRIRAIENIDTLTNLESLFLGKNKITKLQNLDALTNLTVLSMQSN

RLTKIEGLQSLVNLRELYLSHNGIEVIEGLDNNNKLTMLDIASNRIKKIENVSHLTELQE

FWMNDNLLDCWSDLDELKGARSLETVYLERNPLQRDPQYRRKIMLALPSVRQIDATFVRF

>sp|P05980|PGFS1_BOVIN Prostaglandin F synthase 1 OS=Bos taurus OX=9913 PE=1 SV=3

MDPKSQRVKLNDGHFIPVLGFGTYAPEEVPKSEALEATKFAIEVGFRHVDSAHLYQNEEQ

VGQAIRSKIADGTVKREDIFYTSKLWCNSLQPELVRPALEKSLQNLQLDYVDLYIIHSPV

SLKPGNKFVPKDESGKLIFDSVDLCHTWEALEKCKDAGLTKSIGVSNFNHKQLEKILNKP

GLKYKPVCNQVECHPYLNQSKLLEFCKSHDIVLVAYAALGAQLLSEWVNSNNPVLLEDPV

LCAIAKKHKQTPALVALRYQVQRGVVVLAKSFNKKRIKENMQVFDFELTPEDMKAIDGLN

RNIRYYDFQKGIGHPEYPFSEEY

>sp|P52897|PGFS2_BOVIN Prostaglandin F synthase 2 OS=Bos taurus OX=9913 PE=2 SV=1

MDPKSQRVKFNDGHFIPVLGFGTYAPEEVPKSEALEATKFAIEVGFRHVDSAHLYQNEEQ

VGQAIRSKIADGTVKREDIFYTSKLWCNSLQPELVRPALEKSLQNLQLDYVDLYIIHSPV

SLKPGNKFVPKDESGKLIFDSVDLCHTWEALEKCKDAGLTKSIGVSNFNHKQLEKILNKP

GLKYKPVCNQVECHPYLNQSKLLEFCKSHDIVLVAYAALGAQLLSEWVNSNNPVLLEDPV

LCAIAKKHKQTPALVALRYQVQRGVVVLAKSFNKKRIKENMQVFDFELTPEDMKAIDGLN

RNTRYYDFQQGIGHPEYPFSEEY

>sp|Q3T0X5|PSA1_BOVIN Proteasome subunit alpha type-1 OS=Bos taurus OX=9913 GN=PSMA1 PE=1 SV=1

MFRNQYDNDVTVWSPQGRIHQIEYAMEAVKQGSATVGLKSKTHAVLVALKRAQSELAAHQ

KKILHVDNHIGISIAGLTADARLLCNFMRQECLDSRFVFDRPLPVSRLVSLIGSKTQIPT

QRYGRRPYGVGLLIAGYDDMGPHIFQTCPSANYFDCRAMSIGARSQSARTYLERHMSEFM

ECNLNELVKHGLRALRETLPAEQDLTTKNVSIGIVGKDLEFTIYDDDDVSPFLEGLEERP

QRKAQPTQPADEPAEKADEPMEH

>sp|Q3ZCK9|PSA4_BOVIN Proteasome subunit alpha type-4 OS=Bos taurus OX=9913 GN=PSMA4 PE=1 SV=1

MSRRYDSRTTIFSPEGRLYQVEYAMEAIGHAGTCLGILANDGVLLAAERRNIHKLLDEVF

FSEKIYKLNEDMACSVAGITSDANVLTNELRLIAQRYLLQYQEPIPCEQLVTALCDIKQA

YTQFGGKRPFGVSLLYIGWDKHYGFQLYQSDPSGNYGGWKATCIGNNSAAAVSMLKQDYK

EGEMTLKSALALAIKVLNKTMDVSKLSAEKVEIATLTRENGKTVIRVLKQKEVEQLIKKH

EEEEAKAEREKKEKEQKEKDK

>sp|Q2YDE4|PSA6_BOVIN Proteasome subunit alpha type-6 OS=Bos taurus OX=9913 GN=PSMA6 PE=1 SV=1

MSRGSSAGFDRHITIFSPEGRLYQVEYAFKAINQGGLTSVAVRGKDCAVIVTQKKVPDKL

LDSSTVTHLFKITENIGCVMTGMTADSRSQVQRARYEAANWKYKYGYEIPVDMLCKRIAD

ISQVYTQNAEMRPLGCCMILIGIDEEQGPQVYKCDPAGYYCGFKATAAGVKQTESTSFLE

KKVKKKFDWTFEQTVETAITCLSTVLSIDFKPSEIEVGVVTVENPKFRILTEAEIDAHLV

ALAERD

>sp|Q3MHN0|PSB6_BOVIN Proteasome subunit beta type-6 OS=Bos taurus OX=9913 GN=PSMB6 PE=1 SV=1

MAATLVAARGTRPAPAWGPEAIAPDWENREVSTGTTIMAVQFDGGVVLGADSRTTTGSYI

ANRVTDKLTPIHDRIFCCRSGSAADTQAVADAVTYQLGFHSIELNEPPLVHTAASLFKEM

CYRYREDLMAGIIIAGWDPQEGGQVYSVPMGGMMVRQPFAIGGSGSSYIYGYVDATYREG

MTKEECLQFTANALALAMERDGSSGGVIRLAAIAEPGVERQVLLGDQIPKFTIATLPPL

>sp|Q2KJ25|PSD12_BOVIN 26S proteasome non-ATPase regulatory subunit 12 OS=Bos taurus OX=9913 GN=PSMD12 PE=2 SV=3

MADGGSERADGRIVKMEVDYSATVDQRLPECEKLAKEGRLQEVIETLLSLEKQTRTASDM

VSTSRILVAIVKMCYEAKEWDLLNENIMLLSKRRSQLKQAVAKMVQQCCTYVEEITDLPI

KLRLIDTLRMVTEGKIYVEIERARLTKTLATIKEQNGDVKEAASILQELQVETYGSMEKK

ERVEFILEQMRLCLAVKDYIRTQIISKKINTKFFQEENTEKLKLKYYNLMIQLDQHEGSY

LSICKHYRAIYDTPCIQAESEKWQQALKSVVLYVILAPFDNEQSDLVHRISGDKKLEEIP

KYKDLLKLFTTMELMRWSTLVEDYGMELRKGSLESPATDVFGYTEEGEKRWKDLKNRVVE

HNIRIMAKYYTRITMKRMAQLLDLSVDESEAFLSNLVVNKTIFAKVDRLAGIINFQRPKD

PNNLLNDWSQKLNSLMSLVNKTTHLIAKEEMIHNLQ

>sp|P56701|PSMD2_BOVIN 26S proteasome non-ATPase regulatory subunit 2 OS=Bos taurus OX=9913 GN=PSMD2 PE=1 SV=2

MEEGGRDKAPLQPQQPPATSPGSGDEKPSGKERRDAGDKDKEQELSEEDKQLQDELEMLV

ERLGEKDTSLYRPALEELRRQIRSSTTSMTSVPKPLKFLRPHYGKLKEIYENMAPGENKR

FAADIISVLAMTMSGERECLKYRLVGSQEELASWGHEYVRHLAGEVAKEWQELDDAEKTQ

REPLLTLVKEIVPYNMAHNAEHEACDLLMEIEQVDMLEKDIDENAYAKVCLYLTSCVNYV

PEPENSALLRCALGVFRKFSRFPEALRLALMLNDMELVEDIFTSCKDVVVQKQMAFMLGR

HGVFLELSEDVEEYEDLTEIMSNVQLNSNFLALARELDIMEPKVPDDIYKTHLENNRFGG

SGSQVDSARMNLASSFVNGFVNAAFGQDKLLTDDGNKWLYKNKDHGMLSAAASLGMILLW

DVDGGLTQIDKYLYSSEDYIKSGALLACGIVNSGVRNECDPALALLSDYVLHNSNTMRLG

SIFGLGLAYAGSNREDVLTLLLPVMGDSKSSMEVAGVTALACGMIAVGSCNGDVTSTILQ

TIMEKSETELKDTYARWLPLGLGLNHLGKGEAIEAILAALEVVSEPFRSFANTLVDVCAY

AGSGNVLKVQQLLHICSEHFDSKEKEEDKDKKEKKDKDKKEAPADMGAHQGVAVLGIALI

AMGEEIGAEMALRTFGHLLRYGEPTLRRAVPLALALISVSNPRLNILDTLSKFSHDADPE

VSYNSIFAMGMVGSGTNNARLAAMLRQLAQYHAKDPNNLFMVRLAQGLTHLGKGTLTLCP

YHSDRQLMSQVAVAGLLTVLVSFLDVRNIILGKSHYVLYGLVAAMQPRMLVTFDEELRPL

PVSVRVGQAVDVVGQAGKPKTITGFQTHTTPVLLAHGERAELATEEFLPVTPILEGFVIL

RKNPNYDL

>sp|Q2KJ46|PSMD3_BOVIN 26S proteasome non-ATPase regulatory subunit 3 OS=Bos taurus OX=9913 GN=PSMD3 PE=2 SV=1

MKQEGSARRRGADKAKPPPGGGEQEPPPPPAPQDVEMKEEAAAGGGSTGETAGKTAAAAA

EHSQRELDTVTLEDIKEHVKQLEKAVSGKEPRFVLRALRMLPSTSRRLNHYVLYKAVHGF

FTSNNATRDFLLPFLEEPMDTEADLQFRPRTGKAASAPLLPEVEAYLQLLMVIFLMNSKR

YKEAQKISDDLMQKISTQNRRALDLVAAKCYYYHARVYEFLDKLDVVRSFLHARLRTATL

RHDTDGQATLLNLLLRNYLHYSLYDQAEKLVSKSVFPEQANNNEWARYLYYTGRIKAIQL

EYSEARRTMTNALRKAPQHTAVGFKQTVHKLLIVVELLLGEIPDRLQFRQPSLKRSLMPY

FLLTQAVRTGNLAKFNQVLDQFGEKFQADGTYTLIIRLRHNVIKTGVRMISLSYSRISLA

DIAQKLQLDSPEDAEFIVAKAIRDGVIEASINHEKGYVQSKEMIDIYSTREPQLAFHQRI

SFCLDIHNMSVKAMRFPPKSYNKDLESAEERREREQQDLEFAKEMAEDDDDSFP

>sp|Q0VCG9|PTX3_BOVIN Pentraxin-related protein PTX3 OS=Bos taurus OX=9913 GN=PTX3 PE=2 SV=1

MHISVILFCALWSAVSAENSDDYELMYVNLDNEIDNGLHPTEDPTPCDCSRENSEWDKLF

TMLENSQMREGMLLQATDVMLRGELQKLQAELGRLEGSLQKLCGPEAPSETRLARALDDL

LQASRDAGRRLARLEDAGALRPQEEAGRALGAVLEELRRTRADLRAVQGWAASRWLPAGC

ETAILFPMRSKKIFASVHPVTPMKLETFSACIWVKATEVLNKTVLFSYGTKRNPYEIQLY

LSYRSIMLVVGGEENRLVADAVISLGTWTHLCSTWDSKKGHMALWVNGDSVATAVDMATG

HVVPEGGILQIGQEKNGCCVGGGFDETLAFSGRLTGFNIWEGVLSNEEIREAGGAESCHI

RGNVVGWGVTEIQPHGGAQYVY

>sp|P82943|REG1_BOVIN Regakine-1 OS=Bos taurus OX=9913 PE=1 SV=2

MRVSLAALAFLLTLAVLHSEANEEPAGNMRVCCFSSVTRKIPLSLVKNYERTGDKCPQEA

VIFQTRSGRSICANPGQAWVQKYIEYLDQMSK

>sp|O02739|SPB6_BOVIN Serpin B6 OS=Bos taurus OX=9913 GN=SERPINB6 PE=2 SV=1

MDALSEANGTFALTLLKKLGEGNSKNVFISPLSISSALAMVLLGAKGNTAAQMCQTLSLN

KSSGGGEDVHQGFQNLLSEVNRRDTQYLLRTANRLFGEKTYDFLSSFKDSCHKFYQAEME

ELDFVSATEQSRKHINTWVAEKTEGKIRDLLPANSVNPMTRLVLVNAIYFKGNWDTQFNK

EHTEERPFRVSKNVEKPVQMMFKKSTCKITYIGEISTQILVLPYVGQELNMVILLPSEST

DLNTVEKALTYEKFIAWTKPDVMDEEEVEVFLPRFTLEESYDMEEFLQELGMTDAFEETR

ADFSGMSSGRGLHLSKVMHKSFVEVTEEGTEAAAATGAVVMMRCLMVVPRFNANHPFLFF

IQHSKTGAILFCGRFCSP

>sp|Q32LM0|SHLB1_BOVIN Endophilin-B1 OS=Bos taurus OX=9913 GN=SH3GLB1 PE=2 SV=1

MNIMDFNVKKLAADAGTFLSRAVQFTEEKLGQAEKTELDAHLENLLSKAECTKIWTEKIM

KQTEVLLQPNPNARIEEFVYEKLDRKAPSRINNPELLGQYMIDAGTEFGPGTAYGNALIK

CGETQKRIGTADRELIQTSALNFLTPLRNFIEGDYKTIAKERKLLQNKRLDLDAAKTRLK

KAKAAETRASSEQELRITQSEFDRQAEITRLLLEGISSTHAHHLRCLNDFVEAQMTYYAQ

CYQYMLDLQKQLGSFPSNYHSNNNQTAVAPVPSASSNVIGSSALTSTSSLVITSPSNLTD

LKECGGSRRARVLYDYDAANSTELSLLADEVITVFSVVGMDSDWLMGERGNQKGRVPITY

LELLN

>sp|Q5E9S9|S38A5_BOVIN Sodium-coupled neutral amino acid transporter 5 OS=Bos taurus OX=9913 GN=SLC38A5 PE=2 SV=1

MAISSAEGMELQDPKMNGALPGNAVEQEHEGFLPSHSPSPGRKPAQFMDFEGKTSFGMSV

FNLSNAIMGSGILGLAYAMAHTGILLFLALLLCIALLSSYSIHLLLTCAGVVGIRAYEQL

GQRALGPAGKVVVAAVICLHNVGAMSSYLFIIKSELPLVIATFLDMDPEGDWFLKGNLLI

IIVSVLIILPLALMRHLGYLGYTSGLSLTCMLFFLISVIYKKFQLGCTVGHNGTAVESKS

SPSLPIHGLNTSCEAQMFTADSQMFYTVPIMAFAFVCHPEVLPIYTELCRPSKRRMQAVA

NVSIGAMFCMYGLTATFGYLTFYSSVEAEMLHMYSQHDLLILCVRLAVLLAVTLTVPVVL

FPIRRALQQLLFPSKAFSWPRHVAIALILLVLVNVLVICVPTIRDIFGVIGSTSAPSLIF

ILPSIFYLRIVPSEVEPLYSWPKIQALCFGVLGVLFMAISLGFMFANWATGQSHVSGH

>sp|Q95115|STA5A_BOVIN Signal transducer and activator of transcription 5A OS=Bos taurus OX=9913 GN=STAT5A PE=2 SV=2

MAGWIQAQQLQGDALRQMQVLYGQHFPIEVRHYLAQWIESQPWDAIDLDNPQDRAQATQL

LEGLVQELQKKAEHQVGEDGFLLKIKLGHYATQLQNTYDRCPMELVRCIRHILYNEQRLV

REANNGSSSAGILVDAMSQKHLQINQTFEELRLVTQDTENELKKLQQTQEYFIIQYQESL

RIQAQFAQLAQLNPQERLSRETALQQKQVSLEAWLQREAQTLQQYRVELAEKHQKTLQLL

RKQQTIILDDELIQWKRRQQLAGNGGPPEGSLDVLQSWCEKLAEIIWQNRQQIRRAEHLC

QQLPIPGPVEEMLAEVNATITDIISALVTSTFIIEKQPPQVLKTQTKFAATVRLLVGGKL

NVHMNPPQVKATIISEQQAKSLLKNENTRNECSGEILNNCCVMEYHQATGTLSAHFRNMS

LKRIKRADRRGAESVTEEKFTVLFESQFSVGSNELVFQVKTLSLPVVVIVHGSQDHNATA

TVLWDNAFAEPGRVPFAVPDKVLWPQLCEALNMKFKAEVQSNRGLTKENLVFLAQKLFNS

SSSHLEDYNGMSVSWSQFNRENLPGWNYTFWQWFDGVMEVLKKHHKPHWNDGAILGFVNK

QQAHDLLINKPDGTFLLRFSDSEIGGITIAWKFDSPDRNLWNLKPFTTRDFSIRSLADRL

GDLNYLIYVFPDRPKDEVFSKYYTPVLAKAVDGYVKPQIKQVVPEFVSASADSAGSNATY

MDQAPSPAVCPQPHYNMYPQNPDPVLDQDGEFDLDETMDVARHVEELLRRPMDSLEPSLP

PPTGLFTPGRGSLS

>sp|Q9TUM3|STA5B_BOVIN Signal transducer and activator of transcription 5B OS=Bos taurus OX=9913 GN=STAT5B PE=2 SV=2

MAVWIQAQQLQGDALHQMQALYGQHFPIEVRHYLSQWIEGQAWDSIDLDNPQENIKATQL

LEGLVQELQKKAEHQVGEDGFLLKIKLGHYATQLQNTYDRCPMELVRCIRHILYNEQRLV

REANNGTSPAGSLADAMSQKHLQINQTFEELRLVTQDTENELKKLQQTQEYFIIQYQESL

RIQAQFAQLAQLNPQERLSRETALQQKQLSLEAWLQREAQTLQQYRVELAEKHQKTLQLL

RKQQTIILDDELIQWKRRQQLAGNGGPPEGSLDVLQSWCEKLAEIIWQNRQQIRRAEHFC

QQLPIPGPVEEMLAEVNATITDIISALVTSTFIIEKQPPQVLKTQTKFAATVRLLVGGKL

NVHMNPPQVKATIISEQQAKSLLKNENTRNDYSGEILNNCCVMEYHQATGTLSAHFRNMS

LKRIKRSDRRGAESVTEEKFTILFESQFSVGGNELVFQVKTLSLPVVVIVHGSQDNNATA

TVLWDNAFAEPGRVPFAVPDKVLWPQLCEALNMKFKAEVQSNRGLTKENLVFLAQKLFNS

SSSHLEDYNGMSVSWSQFNRENLPGRNYTFWQWFDGVMEVLKKHLKPHWNDGAILGFVNK

QQAHDLLINKPDGTFLLRFSDSEIGGITIAWKFDSQERMFWNLMPFTTRDFSIRSLADRL

GDLSYLIYVFPDRPKDEVYSKYYTPVPCEPATAKAVDGYVKPQIKQVVPEFVSASADSAG

GSATYMDQAPSPAVCPQPHYNMYPQNPDPVLDNDGDFDLDDTIDVARRVEELLGRPMDSQ

WIPHAQS

>sp|Q28205|TBCD_BOVIN Tubulin-specific chaperone D OS=Bos taurus OX=9913 GN=TBCD PE=1 SV=1

MALSEEPAAGAAEDPVEDPVEDAGEDAALACGAALESFGESAETRELLGHLPAVLADRSA

REGALERFRVIMDKYQEQPHLLDPHLEWMLNLLLEFVQNKTSPADLVHLAFKFLYIISKV

RGYKTFLRLFPHEVADVQPVLDMFTNQNPKDHETWETRYMLLLWLSVTCLIPFDFSRLDG

NLSQPGQERASTMDRILQVAESYLVVSDKARDAAAVLVSKFVTRPDVKQKKMASFLDWSL

CTLARSSFQTIEGVIAMDGTLQALAQIFKHGKREDCLPYAATVLQCLDSCRLPDSNQTLL

RKLGVKLVQRLGLTFLKPQVAKWRYQRGCRSLAESLQHSIQNPREPVTQAETPDSDGQDD

VPEEVESVIEQLLVGLKDKDTIVRWSAAKGIGRMAGRLPKELADDVTGSVLDCFSFQETD

SAWHGGCLALAELGRRGLLLPSRLSDVVPVILRALTYEEKRGACSVGSNVRDAACYVCWA

FARAYEPQELKPFVAAISSALVIATVFDRDVNCRRAASAAFQENVGRQGTFPHGIDILTT

ADYFAVGNRSNCFLVISMFIAGFPEYTQPMIEHLVTMKVGHWDGTIRELSAKALRNLAQR

APEHTAREVFPRLLSMTQSPDLHTRHGAVLACAEVARSLHTLATQQGRPVSDFLDEKAMH

GLKQIHQQLYDRQLYRGLGGELMRQAVCILIENVALSKMPFRGDAVIDGWQWLINDTLKN

LHLISSHSRQHIKEAAVSALAALCSEYHAQEPGEAEAAAQEELVKLYLAELQSPEEMTRC

GCALALGALPAFFLKGRLRQVLAGLRAVTHISPKDVSFAEARRDALKAISRICQTVGVRA

EGPPDEAVCRENVSQIYCTLLDCLKDYTTDSRGDVGAWVREAAMTSLMDLTLLLGRNQPE

LIEAPLCQQLMCCLAQQASEKIDRFRAHAARVFLALLHADSPAIPHVPARPELERLFPRA

AVASVNWGAPSQAFPRMARLLGLPAYRYHVLLGLAVSVGGLTESTVRYSTQGLFEYMKEI

QNDPAALEDFGGTLLQVFEDNLLNDRVSVPLLKTLDQMLANGCFDIFTAQENHPFCVKLL

ALCKEEIKKSKDVQKLRSSIAVFCGLVQFPGDVRRKVLLQLFLLLCHPFPVIRKNTASQV

YEMVLTYDVVPTAVLDEVMAVLSSTAWDAELPVVRAQRNRLCDLLGVPRPQLVPKPAVR

>sp|Q3SZP7|VILI_BOVIN Villin-1 OS=Bos taurus OX=9913 GN=VIL1 PE=2 SV=3

MTKLSAQVKGSLNITTPGVQIWRIEAMQMVPVPSNSFGSFFDGDCYVIQAIHKTGSNLSY

DIHYWIGQASSQDEQGAAAIYTTQMDDFLKGRAVQHREVQGNESDTFRGYFKKGIVIRKG

GVASGMKQVETNSYDIQRLLHVKGKRNVVAGEVEMSWKSFNRGDVFLLDLGKLIIQWNGP

ESNHMERLRGMNLAKEIRDQERGGRTYVGVVDGEDEKASPQLMEIMNHVLGQRKELKAAV

ADTVVEPALKAALKLYHVSDSEGKVVVREIATQPLTQDLLSHEDCYILDQGGLKIYVWKG

KNANAQEKKEAMNQALNFIKAKQYPPSTQVELQNDGAESAVFQQLFQKWTVPNRTTGLGK

THTVGSVAKVEQVKFDAMSMHVQPQVAAQQKMVDDGSGEVQMWRIENLELVPVNTKWLGH

FFGGDCYLLLYTYFINEKPHYLLYIWQGSQASQDEITASAYQAVILDQEYNNEPVQIRVP

MGKEPPHLMSIFKGCMVVYQGGTSRANSVEPVPSTRLFQVRGTSANNTKAFEVSPRAASL

NSNDVFILKTQSCCYLWCGKGCSGDEREMAKMVADTVSRTEKQVVVEGQEPANFWLALGG

KAPYASTKRLQEENLVITPRLFECSNQTGRFLATEIPDFNQDDLEEDDVFLLDVWDQVFF

WIGKNANEDEKKAAATTVQEYLKTHPGGRDLETPIIVVKQGHEPPTFTGWFLAWDPFKWN

NSKSYEDLKAELGNSGDWSQITAELTSSKPEAFNANSNLSSGPLPIFPLEQLVNKPTEEL

PEGVDPSRREEHLSIEDFTRALGMTPSAFWALPRWKQQNLKKEKGLF

>sp|P48616|VIME_BOVIN Vimentin OS=Bos taurus OX=9913 GN=VIM PE=1 SV=3

MSTRSVSSSSYRRMFGGPGTASRPSSTRSYVTTSTRTYSLGSALRPTTSRTLYTSSPGGV

YATRSSAVRLRSGVPGVRLLQDSVDFSLADAINTEFKNTRTNEKVELQELNDRFANYIDK

VRFLEQQNKILLAELEQLKGQGKSRLGDLYEEEMRELRRQVDQLTNDKARVEVERDNLAE

DIMRLREKLQEEMLQREEAESTLQSFRQDVDNASLARLDLERKVESLQEEIAFLKKLHDE

EIQELQAQIQEQHVQIDMDVSKPDLTAALRDVRQQYESVAAKNLQEAEEWYKSKFADLSE

AANRNNDALRQAKQESNEYRRQVQTLTCEVDALKGTNESLERQMREMEENFSVEAANYQD

TIGRLQDEIQNMKEEMARHLREYQDLLNVKMALDIEIATYRKLLEGEESRISLPLPNFSS

LNLRETNLDSLPLVDTHSKRTLLIKTVETRDGQVINETSQHHDDLE

>sp|Q8SPP7|PGRP1_BOVIN Peptidoglycan recognition protein 1 OS=Bos taurus OX=9913 GN=PGLYRP1 PE=1 SV=1

MSRRYTPLAWVLLALLGLGAAQDCGSIVSRGKWGALASKCSQRLRQPVRYVVVSHTAGSV

CNTPASCQRQAQNVQYYHVRERGWCDVGYNFLIGEDGLVYEGRGWNTLGAHSGPTWNPIA

IGISFMGNYMHRVPPASALRAAQSLLACGAARGYLTPNYEVKGHRDVQQTLSPGDELYKI

IQQWPHYRRV

>tr|A0A0A0MP92|A0A0A0MP92_BOVIN Serpin A3-7 OS=Bos taurus OX=9913 GN=SERPINA3-7 PE=1 SV=1

MRTERTSFLLALGLLVSGFCSRVHCLPENVTPEEQHKGTSVDGHSLASSNTDFAFSLYKQ

LALKDPNKNVIFSPLSISIALAFLSLGAHDHTVTEILEGLKFNLTETPETEIHQGFQHLL

QTFNQPSNQLQLSVGNAMFVSEELKLLDKFRKDAEAFYASEVLSTNFKDSEAAVKLINEY

VKNKTHGKIEKLFNDLDVLTNLILLNYIFFKAQWKTPFNPNHTYESEFHVSQNERVIVPM

MTLYLETPYFRDEELGCTLVELTYTSNDSALFILPDEGKMQDLEAKLTPETLTRWRNSLQ

PRLIHRLRLPRFSISSHYQLKDILSQLGIKKIFTSDADFSGITDDHKLAVSHVIHKAVLD

VGEEGTEGAAVTAVVMATSSLLHTLTVSFNRPFLLSIFCKETQSIIFLGKVTNPKEA

>tr|A0A0A8J2N9|A0A0A8J2N9_BOVIN Amino acid transporter OS=Bos taurus OX=9913 GN=ASCT2 PE=2 SV=1

MVADPPKGDPKGYAAAEPTANGVSMLVPIEDVGSLKGGRCGSGDQVRRCLRANLLVLLTV

VAVVAGVALGLGVSGAGGAFALGPARLEAFSFPGELLLRLLKMIILPLVVCSLIGGAASL

DPSALGRLGAWALLFFLVTTLLASALGVGLALALQPGAAFAAINTSVGAPVEEAPSKEVL

DSFLDLVRNIFPSNLVSAAFRSYTTSYKERLFNGTLVKVPTGGEVEGMNILGLVVFAIIF

GVALRKLGPEGELLIRFFNSFNDATMVLVSWIMWYAPVGILFLVAGKIVEMENVGLLFAS

LGKYILCCLLGHAIHGLLTLPLIYFLFARKNPYRFLWGIMTPLATAFGTSSSSATLPLMM

KCVEEKNGVARHISRFILPIGATVNMDGAALFQCVAAVFIAQLNHRSLDFVKIITILVTA

TASSVGAAGIPSGGVLTLAIILEAVNLPVHDISLILAVDWLVDRSCTVLNVEGDAFGAGL

LQSYLDRTENCNSVPELIQVKSEMPLAALPVPGEEGNPLLKGCPGPAGDADTCEKESVM

>tr|A0A0F6QMJ3|A0A0F6QMJ3_BOVIN Complement component 5 OS=Bos taurus OX=9913 GN=C5 PE=2 SV=1

MGLWGILCFLIFLGKSWGQEQTYVISAPKVFHVGAYENVVIQAYGYTEEFDATVSIKSFP

DKKVTYSSGHVTLSAENKFQNSATLTIQPKQLSERQSSVSHVYLEVTSKHFSKAKKMPVT

YDNGFLFIHTDKPVYTPHQSVKVRVYSLNDDLKPAKRETVLTFIDPEGSEVDMVEENDYT

GIISFPDFKIPSNPKYGVWTIRAKYKEDFSTTGTAYFEIKEYVLPHFSISIEPKSNFIGY

KDFTNFEITIRARYFYNKVVNEADVYITFGIRDNLEDNQKEMMQKAMQSTALINGMAQVT

FNSETAVKELSYESLEDLNNKYLYIGVTIIESTGGFSEEAEIPGIKYVLSPYKLNLVATP

LFVKPGIPYSVKVQVKDSFDHLVGGIPVTLSAKTLDANQEITDLESKKSVTRSSDGVASF

VVNLPSEVTVLEFNIKTEDLDLPEENQASEDYRAVAYSSLSQSYLYIDWTENYKHLLVGA

HLNIVVTPRSPYIDKITHYNFLILSKGKIVHFGTRDKLSDSAYQSINIPVTQDMVPSARL

LVYYIVTGEQTAELVSDSVWLNIEEKCGNQLEVHLSPETDAYSPGQRVSLNMETELDSWV

ALTAVDSAIYGVQRTAKRPLERVFQTLEKGDLGCGAGGGRDNAEVFYLAGLTFLTNANAD

DTRENDEPCKEILRPKRMLKKKIEEEAAKYRNAWVKKCCYDGAHRNDDETCEQRAARIQA

GPICIKAFKSCCAIASQFRADEHHKNMQLGRLHIKSLLPVTKPEIRSYFPESWLWEVHRV

PKRNQLQFVLPDSLTTWEIQGVGISNSGICVADTLKAKVFKSVFLEMNIPYTVVRGEQVQ

LSGTVYNYRTSGIQFCVKMHPVEGICSSGTPVTDSQGRRFSKCVLQKIEGSSNHLVTFTV

LPLEVGLHNLSFSLETSVGNEILVKTLRVVPEGVKRESYAGVTLDPQGVYGIISRRKEFP

YRIPLNMVPKTKVKRTVSIKGLLIGEIMSAVLSQEGIDSLTRLPKGNAEAELMSVVPVFY

VYHYLEAGHNWDIFSTNSLTQKQNLKTKLKEGMVSIMSFRNADYSYSMWKGGSASTWLTA

FALRVLGQISKYVDQNQNSICNSLLWLIEKCQLENGSFKENSDYQPVKLQGTLPVEAREK

TLYLTAFAVIGIRKAFDICPLEKISTAVSKADIFLHENALSSQSAFTLAIAAYALSLGDK

AHPQFRAIVSALKRKAFVKGNPPIYRFWKDDLQQRDSFVPNTGTARMVETTAYALLTNLN

LKDMNYVNPVIKWLSEEQRYGGGFYSTQDTINAIEGLTEYSLLVRKLRLNMDVKVSYKNK

GDLYHYKMSDKYFLGRPIEVPLNDDLVVLSTGQSSGLATVHVKTVVHKISTSEEVCSFHL

KIETREIEAFGYSSSEYKRIVACASYKLSSEESSSGSSHAVMDISLPTGVNANPEDLKAL

VEQVDQLLTDYEIKDGHVILQLNSIPSNEFLCVRFRIIELFEVGFLSPATFTVYEYHRPD

KQCTMFYSTSNTKLQKVCEGVTCKCIEADCGQMQAELDLTISAATRKETACKPEIAYAYK

VEIVAVTEENAFVKYTATLLDIYKAGEAAAERDAEITFIKKTSCSNANLEKRKQYLIMGK

EALQIKHNFRFKYVYPLDSSTWIEYWPTDSRCPSCQRFLANLDEFTEDIFLNGCENA

>tr|A0A140T843|A0A140T843_BOVIN Beta-2-glycoprotein 1 OS=Bos taurus OX=9913 GN=APOH PE=4 SV=1

MLPPALVLLLGFLCHVAIAGRTCPKPDELPFSTVVPLKRTYEPGEQIVFSCQPGYVSRGG

IRRFTCPLTGLWPINTLKCMPRVCPFAGILENGTVRYTTFEYPNTISFSCHTGFYLKGAS

SAKCTEEGKWSPDLPVCAPITCPPPPIPKFASLSVYKPLAGNNSFYGSKAVFKCLPHHAM

FGNDTVTCTEHGNWTQLPECREVRCPFPSRPDNGFVNHPANPVLYYKDTATFGCHETYSL

DGPEEVECSKFGNWSAQPSCKASCKLSIKRATVIYEGERVAIQNKFKNGMLHGQKVSFFC

KNKEKKCSYTEDAQCIDGTIEIPKCFKEHSSLAFWKTDASDVKPC

>tr|A0A140T851|A0A140T851_BOVIN Vitamin K-dependent protein C OS=Bos taurus OX=9913 GN=PROC PE=4 SV=2

MAAGGQPCSFSAAHPNSVFSSSQRAHQVLRIRKRANSFLEELRPGNVERECSEEVCEFEE

AREIFQNTEDTMAFWSKYSDGDQCEDRPSGSPCDLPCCGRGKCIDGLGGFRCDCAEGWEG

RFCLHEVRFSNCSAENGGCAHYCMEEEGRRHCSCAPGYRLEDDHQLCVSKVTFPCGRLGK

RMEKKRKTLKRDTNQVDQKDQLDPRIVDGQEAGWGESPWQAVLLDSKKKLVCGAVLIHVS

WVLTVAHCLDSRKKLIVRLGEYDMRRWESWEVDLDIKEVIIHPNYTKSTSDNDIALLRLA

KPATLSQTIVPICLPDSGLSERKLTQVGQETVVTGWGYRDETKRNRTFVLSFIKVPVVPY

NACVHAMENKISENMLCAGILGDPRDACEGDSGGPMVTFFRGTWFLVGLVSWGEGCGRLY

NYGVYTKVSRYLDWIYGHIKAQEAPLESQVP

>tr|A0A140T872|A0A140T872_BOVIN Dihydrofolate reductase OS=Bos taurus OX=9913 GN=DHFR PE=3 SV=1

MVRPLNCIVAVSQNMGIGKNGNLPWPPLRNEFQYFQRMTTVSSVEGKQNLVIMGRKTWFS

IPEKNRPLKDRINIVLSRELKEPPKGAHFLAKSLDDALELIEDPELTNKVDVVWIVGGSS

VYKEAMNKPGHVRLFVTRIMQEFESDAFFPEIDFEKYKLLPEYPGVPLDVQEEKGIKYKF

EVYEKNN

>tr|A0A140T874|A0A140T874_BOVIN 2-aminomuconic semialdehyde dehydrogenase OS=Bos taurus OX=9913 GN=ALDH8A1 PE=3 SV=1

MAGRGGLLMLENFIGGKFLPCSSYLDSYDPSTGEVYCHVPNSGKEEVEAAVEAARAAFPG

WSSRSPQERSQVLQRLADLLEQSLEELAQAESKDQGKTITLARTMDIPRAVHNFRFFASS

ILHHTSECTQMDHLGCLHYTVRAPVGIAALISPWNLPLYLLTWKIAPAIAAGNTVIAKPS

ELTSVTAWMMCRLLEKAGVPPGVVNIVFGTGPRVGEALVSHPEVPLISFTGSQPTAERIM

QLSAPHCKKLSLELGGKNPAVIFEDANLAECIPTTVRSSFANQGEICLCTSRIFVQRSIY

SEFLKRFVEAARMWKVGIPSDPSADMGALISKAHLEKVRSYIKKARMEGAQILCGEGVDK

LNLPPRNQAGYFMLPTVITDVKDESCCMKEEIFGPVTCVVPFDSEEEVIQRANNVKYGLA

ATVWSGNVGRVHRVAKKLQSGLVWTNCWLIRELNLPFGGMKSSGVGREGAKDSYEFFTEV

KTITVKH

>tr|A0A140T881|A0A140T881_BOVIN Apolipoprotein E OS=Bos taurus OX=9913 GN=APOE PE=3 SV=2

MKGSLGWPIASQKMKVLWVAVVVALLAGCQADMEGELGPEEPLTTQQPRGKDSQPWEQAL

GRFWDYLRWVQTLSDQVQEELLNTQVIQELTALMEETMKEVKAYKEELEGQLGPMAQETQ

ARVSKELQAAQARLGSDMEDLRNRLAQYRSEVQAMLGQSTEELRARMASHLRKLRKRLLR

DADDLKKRLAVYQAGASEGAERSLSAIRERFGPLVEQGQSRAATLSTLAGQPLLERAEAW

RQKLHGRLEEVGVRAQDRLDKIRQQLEEVHAKVEEQGNQMRLQAEAFQARLRSWFEPLVE

DMQRQWAGLVEKVQLALRPSPTSPPSENH

>tr|A0A140T887|A0A140T887_BOVIN Uridine 5'-monophosphate synthase OS=Bos taurus OX=9913 GN=UMPS PE=3 SV=1

MAAADALLGSLVTGLYDVQAFKFGNFVLKSGLSSPVYIDLRGIISRPSILNQVAEMLFQT

AENAEINFDTVCGVPYTALPLATIVCSTHEIPMLIRRKEKKDYGTKRLIEGAVNPGDTCL

IIEDVVSSGSSVWETAEVLQKEGLKVTDAVVLVDREQGGRDNLQARGIRLHSVCTLSTVL

RILEQQKKINAETVERVKRFIQENAFVAANPNDSLPSVKKEPKELSFGARAELPGTHPVA

AKLLRLMQKKETNLCLSADVSESRELLQLADALGSRICLLKIHVDILNDFTLDVMKELTT

LAKRHEFLIFEDRKFADIGNTVKKQYEGGVFKIASWADLVNAHAVPGSGVVKGLEEVGLP

LHRACLLVAEMSSAGTLATGSYTEAAVQMAEEHSEFVIGFISGSRVSMKPEFLHLTPGVQ

LEAGGDNLGQQYHSPQEVIGKRGSDIIIVGRGIIASANQLEAAKMYRKAAWEAYLSRLAV

>tr|A0A140T891|A0A140T891_BOVIN Pantetheinase OS=Bos taurus OX=9913 GN=VNN1 PE=3 SV=1

MIMSQLLNYVAVLFFCVSRASSLDTFIAAVYEHAVILPNATLVPVSPEEALAVMNRNLDL

LEGAVTSASKQGAHIIVTPEDGIYGFNFTRESIYPYLEDIPDPQVNWIPCNNPDRFGHTP

VQQRLSCLAKDNSIYIVANIGDKKSCNASDPQCPPDGRYQYNTDVVFDTKGKLVARYHKQ

NLFLNEDQFNAPKEPEVVTFNTTFGKFGIFTCFDILFHDPAVTLVRDSRVDTILFPTAWM

NVLPHLSAIEFHSAWAMGMRVNFLASNLHYPLKKMTGSGIYAPDSPRAFHYDMKTEEGKL

LLAQLDSHPHPTPVVNWTSYASGVEAHSVGNQEFTGIIFFDEFTFLELKEIGGNYTVCQR

DLCCHLSYKMSEKRSDEVYALGAFDGLHTVEGSYYLQICTLLKCKTTDLHTCGDSVETAS

TRFEMFSLSGTFGTQYVFPEVLLSEIQLAPGEFQVSNDGRLFSLKPTSGPVLTVTLFGRL

YEKDSAPNTLSDLTTQALRLNPKTDAWKSK

>tr|A0A140T894|A0A140T894_BOVIN 14-3-3 protein beta/alpha OS=Bos taurus OX=9913 GN=YWHAB PE=3 SV=1

MTMDKSELVQKAKLAEQAERYDDMAAAMKAVTEQGHELSNEERNLLSVAYKNVVGARRSS

WRVISSIEQKTERNEKKQQMGKEYREKIEAELQDICNDVLELLDKYLIPNATQPESKVFY

LKMKGDYFRYLSEVASGDNKQTTVSNSQQAYQEAFEISKKEMQPTHPIRLGLALNFSVFY

YEILNSPEKACSLAKTAFDEAIAELDTLNEESYKDSTLIMQLLRDNLTLWTSENQGDEGD

AGEGEN

>tr|A0A140T896|A0A140T896_BOVIN Serine hydroxymethyltransferase OS=Bos taurus OX=9913 GN=SHMT1 PE=3 SV=1

MAAPVNKAPRDANLWSLHEKMLAQPLKDNDVEVYNIIKKESNRQRVGLELIASENFASRA

VLEALGSCLNNKYSEGYPGQRYYGGTEFIDELEVLCQKRALQVYGLDSQCWGVNVQPYSG

SPANFAVYTALVEPHGRIMGLDLPDGGHLTHGFMTDKKKISATSIFFESMPYKVNPDTGY

INYDQLEENARLFHPRLIIAGTSCYSRNLDYARLRKIADDNGAYLMADMAHVSGLVAAGV

VPSPFEHCHVVSTTTHKTLRGCRAGMIFYRKGVRSVDPKTGRETRYNLESLINSAVFPGL

QGGPHNHAIAGVAVALKQAMTPEFRAYQRQVVANCRALAEALMGLGYRVVTGGSDNHLIL

VDLRSKGTDGGRAEKVLEACSIACNKNTCPGDKSALRPSGLRLGTPALTSRGLLEEDFQK

VAHFIHRGIELTLQIQDAVGVKATLKEFMEKLAGAEEHQRAVAALRAEVESFATLFPLPG

LPGF

>tr|A0A140T897|A0A140T897_BOVIN Albumin OS=Bos taurus OX=9913 GN=ALB PE=4 SV=1

MKWVTFISLLLLFSSAYSRGVFRRDTHKSEIAHRFKDLGEEHFKGLVLIAFSQYLQQCPF

DEHVKLVNELTEFAKTCVADESHAGCEKSLHTLFGDELCKVASLRETYGDMADCCEKQEP

ERNECFLSHKDDSPDLPKLKPDPNTLCDEFKADEKKFWGKYLYEIARRHPYFYAPELLYY

ANKYNGVFQECCQAEDKGACLLPKIETMREKVLTSSARQRLRCASIQKFGERALKAWSVA

RLSQKFPKAEFVEVTKLVTDLTKVHKECCHGDLLECADDRADLAKYICDNQDTISSKLKE

CCDKPLLEKSHCIAEVEKDAIPENLPPLTADFAEDKDVCKNYQEAKDAFLGSFLYEYSRR

HPEYAVSVLLRLAKEYEATLEECCAKDDPHACYSTVFDKLKHLVDEPQNLIKQNCDQFEK

LGEYGFQNALIVRYTRKVPQVSTPTLVEVSRSLGKVGTRCCTKPESERMPCTEDYLSLIL

NRLCVLHEKTPVSEKVTKCCTESLVNRRPCFSALTPDETYVPKAFDEKLFTFHADICTLP

DTEKQIKKQTALVELLKHKPKATEEQLKTVMENFVAFVDKCCAADDKEACFAVEGPKLVV

STQTALA

>tr|A0A140T8A5|A0A140T8A5_BOVIN Isocitrate dehydrogenase [NADP] OS=Bos taurus OX=9913 GN=IDH1 PE=1 SV=1

MSQKIQGGSVVEMQGDEMTRIIWELIKEKLIFPYVELDLHSYDLGIENRDATNDQVTKDA

AEAIKKYNVGVKCATITPDEKRVEEFKLKQMWKSPNGTIRNILGGTVFREAIICKNIPRL

VSGWVKPIIIGRHAYGDQYRATDFVVPGPGKVEISYTPSDGSPKTVYLVHNFTESGGVAM

GMYNQDKSIEDFAHSSFQMALSKNWPLYLSTKNTILKKYDGRFKDIFQEIYDKQYKSEFE

AQNIWYEHRLIDDMVAQAMKSEGGFIWACKNYDGDVQSDSVAQGYGSLGMMTSVLVCPDG

KTVEAEAAHGTVTRHYRMYQKGQETSTNPIASIFAWTRGLAHRAKLDNNKELSFFAKALE

EVCIETIEAGFMTKDLAACIKGLPNVQRSDYLNTFEFMDKLGENLQLKLAQAKL

>tr|A0A140T8C8|A0A140T8C8_BOVIN Kininogen-1 OS=Bos taurus OX=9913 GN=KNG1 PE=4 SV=1

MKLITILFLCSRLLPSLTQESSQEIDCNDQDVFKAVDAALTKYNSENKSGNQFVLYRITE

VARMDNPDTFYSLKYQIKEGDCPFQSNKTWQDCDYKDSAQAATGECTATVAKRGNMKFSV

AIQTCLITPAEGPVVTAQYECLGCVHPISTKSPDLEPVLRYAIQYFNNNTSHSHLFDLKE

VKRAQRQVVSGWNYEVNYSIAQTNCSKEEFSFLTPDCKSLSSGDTGECTDKAHVDVKLRI

SSFSQKCDLYPVKDFVQPPTRLCAGCPKPIPVDSPDLEEPLSHSIAKLNAEHDGAFYFKI

DTVKKATVQVVAGLKYSIVFIARETTCSKGSNEELTKSCEINIHGQILHCDANVYVVPWE

EKVYPTVNCQSLGQTSLMKRPPGFSPFRSVQVMKTEGSTTVSVPHSAMSPVQDEERDSGK

EQGPTHGHGWDHGKQIKLHGLGLGHKHKHDQGHGHHRSHGLGHGHQKQHGLGHGHKHGHG

HGKHKNKGKNNGKHYDWRTPYLASSYEDSTTSSAQTQEKTEETTLSSLAQPGVAITFPDF

QDSDLIATVMPNTLPPHTESDDDWIPDIQTEPNSLAFKLISDFPETTSPKCPSRPWKPVN

GVNPTVEMKESHDFDLVDALL

>tr|A0A1C9EIX3|A0A1C9EIX3_BOVIN Heat shock protein beta-1 OS=Bos taurus OX=9913 GN=HSPB1 PE=2 SV=1

MAERRVPFSLLRGPSWDPFRDWYPAHSRLFDQAFGLPRLPEEWSQWLSHSGWPGYVRALP

AAAIEGPAYNRALSRQLSSGVSEIQQTADRWRVSLDVNHFAPEELTVKTKDGVVEITGKH

EERQDEHGYISRCFTRKYTLPPGADPTLVSSSLSPEGTLTVEAPLPKSATQSAEITIPVT

FQARAQLGGPEAGKSEQPENK

>tr|A0A1C9EIX6|A0A1C9EIX6_BOVIN Heat shock protein beta-1 OS=Bos taurus OX=9913 GN=HSPB1 PE=2 SV=1

MAERRVPFSLLRGPSWDPFRDWYPAHSRLFDQAFGLPRLPEEWSQWLSHSGWPGYVRALP

AAAIEGPAYNRALSRQLSSGVSEIQQTADRWRVSLDVNHFAPEELTVKTKDGVVEITGKH

EERQDEHGYISRCFTRKYTLPPGVDPTLVPSSLSPEGTLTVEAPLPKSATQSAEITIPVT

FQARAQLGGPEAGKSEQPENK

>tr|A0A1K0FUD3|A0A1K0FUD3_BOVIN Globin C1 OS=Bos taurus OX=9913 GN=GLNC1 PE=3 SV=1

MVLSAADKGNVKAAWGKVGGHAAEYGAEALERMFLSFPTTKTYFPHFDLSHGSAQVKGHG

AKVAAALTKAVEHLDDLPGALSELSDLHAHKLRVDPVNFKLLSHSLLVTLASHLPSDFTP

AVHASLDKFLANVSTVLTSKYR

>tr|A0A2Q9|A0A2Q9_BOVIN Alanine--tRNA ligase (Fragment) OS=Bos taurus OX=9913 PE=3 SV=1

MDSTLTAGEIRQRFIDFFKRNEHTYVHSSATIPLDDPTLLFANAGMNQFKPIFLNTIDPS

HPMAKLSRAANTQKCIRAGGKHNDLDDVGKDVYHHTFFEMLGSWSFGDYFKELACKMALE

LLTQEFGIPVERLYVTYFGGDEAAGLEPDLECKQIWQNLGLDDSRILPGNMKDNFWEMGD

TGPCGPCSEIHYDRIGGRDAAHLVNQDDPNVLEIWNLVFIQYNRETDGILKPLPKKSIDT

GMGLERLVSVLQNKMSNYDTDLFVPYFEAIQKGTGARPYTGRVGAEDADGIDMAYRVLAD

HARTITVALADGGRPDNTGRGYVLRRILRRAVRYSHEKLNASRGFFATLVDVVVQSLGDA

FPELKKDPDMVKDIINEEEVQFLKTLSRGRRILDRKIQSLGDSKTIPGDTAWLLYDTYGF

PVDLTALIAEEKGLVVDMDGFDEERKLAQLKSQGKGAGGEDLIMLDIYAIEELREKGLEA

TDDSPKYSYHSDSSGTYVFESAVATVMALRRDRMFVEEASTGQECGVVLDKTCFYAEQGG

QTYDEGYLVKVDDSSEDKTEFTVKNTQVRGGYVLHIGTLYGSLKVGDQVRLFIDEPRRRP

VMSNHTATHILNFALRSVLGEADQRGSLVAPDRLRFDFTAKGAMSTQQIKKAEEIANEMI

EAAKPVYTEDCSLAAAKAIQGLRAVFDETYPDPVRVVSIGVPVSELLDDPSGPAGSLTSV

EFCGGTHLQNSSHAGAFVIVSEEAIAKGIRRIVAVTGAEAQKALRKAESLKKSLCVVEAK

VKAQAAPNKDVQREIADLGEALATAIIPQWQKDEFRENLKSLKKVMDDLDRASKADVQKR

VLEKTKQLIDSSPNQPLVILEVESGASAK

>tr|A0A3B0J3V0|A0A3B0J3V0_BOVIN Adiponectin D OS=Bos taurus OX=9913 GN=ADID PE=4 SV=1

MLLQGALLLLLALPSHGEDNMEDPPLPKGACAGWMAGIPGHPGHNGTPGRDGRDGTPGEK

GEKGDPGLVGPKGDTGETGITGIEGPRGFPGTPGRKGEPGESAYVYRSAFSVGLERQVTV

PNVPIRFTKIFYNQQNHYDGTTGKFLCNIPGLYYFSYHITVYLKDVKVSLYKNDKALLFT

HDQFQDKNVDQASGSVLLYLEKGDQVWLQVYEGENHNGVYADNVNDSTFTGFLLYHNIVE

>tr|A0A3Q1LFG8|A0A3Q1LFG8_BOVIN F-actin-capping protein subunit alpha OS=Bos taurus OX=9913 GN=CAPZA1 PE=1 SV=1

MPNWKVELSLLLGIHVPLCPLSVRIAAKFITHAPPGEFNEVFNDVRLLLNNDNLLREGAA

HAFAQYNMDQFTPVKIEGYEDQVLITEHGDLGNSRFLDPRNKISFKFDHLRKEASDPQPE

EADGGLKSWRESCDSALRAYVKDHYSNGFCTVYAKTIDGQQTIIACIESHQFQPKNFWNG

RWRSEWKFTITPPTAQVVGVLKIQVHYYEDGNVQLVSHKDVQDSVTVSNEAQTAKEFIKI

IEHAENEYQTAISENYQTMSDTTFKALRRQLPVTRTKVDWNKILSYKIGKEMQNA

>tr|A0A3Q1LFR2|A0A3Q1LFR2_BOVIN Glutathione transferase OS=Bos taurus OX=9913 GN=GSTM4 PE=1 SV=1

MPMILGYWDIRGLAHAIRLLLEYTDTNYEERQYSVGDAPDYDRSQWLDEKFKLGLDFPNL

PYLIDGTHKLTQSNAILRYIARKHNMCGETEEEMIRVDILENQVMDVRFAMARICYSPDF

EKLKPGFLKEIPEKIKLFSEFLGKRPWFAGDKLSYVDFLVYDVLDMHRIFEPKCLDAFPN

LKDFISRFEGLKKISAYMKSSRFLPGPLFLKLAVWGNK

>tr|A0A3Q1LFR7|A0A3Q1LFR7_BOVIN Amino acid transporter OS=Bos taurus OX=9913 GN=SLC1A5 PE=3 SV=1

MVADPPKGDPKGYAAAEPTANGVSMLVPIEDVGSLKGGRCGSGDQVRRCLRANLLVLLTV

VAVVAGVALGLGVSGAGGAFALGPARLEAFSFPGELLLRLLKMIILPLVVCSLIGGAASL

DPSALGRLGAWALLFFLVTTLLASALGVGLALALQPGAAFAAINTSVGAPVEEAPSKEVL

DSFLDLVRSEPALPGGGYTTSYKERLFNGTLVKVPTGGEVEGMNILGLVVFAIIFGVALR

KLGPEGELLIRFFNSFNDATMVLVSWIMWYAPVGILFLVAGKIVEMENVGLLFASLGKYI

LCCLLGHAIHGLLTLPLIYFLFARKNPYRFLWGIMTPLATAFGTSSSSATLPLMMKCVEE

KNGVARHISRFILPIGATVNMDGAALFQCVAAVFIAQLNHRSLDFVKIITILVTATASSV

GAAGIPSGGVLTLAIILEAVNLPVHDISLILAVDWLVDRSCTVLNVEGDAFGAGLLQSYL

DRTENCNSVPELIQVKSEMPLAALPVPGEEGNPLLKGCPGPAGDADTCEKESVM

>tr|A0A3Q1LGM4|A0A3Q1LGM4_BOVIN Complement factor I OS=Bos taurus OX=9913 GN=CFI PE=1 SV=1

MKLGHVILLLLCFYLSFCEDNFRKRGKSKAVKKSEAHHAPEASLSKETEASSEVKPTSTQ

DTSQKDFVDKKCLTEKHTHLSCNKVFCQPWQKCIDGTCLCKLPYQCPKNGTRVCSTNGKS

YSTYCQQKSFECYRPEAKFLKSGACTGGGQFSVSLSNGKQDSEGIVAVKLADLDTKMFVC

GDSWSITEANVACIDRGFQLGALDTHRRDPDPNSAECLHVRCRGLETSLAECTFTKGVHN

SEGLAGVVCYTESAAPPKKDSFQCVNGKRIPQKKACDGVNDCVDKSDELCCKDCRGEGFL

CKSGVCIPKQYKCNGELDCITGEDEVGCEETGHPEIKEAAEMLTADMDAERKFTKSFLPK

LSCGVKNNMHIRRKRVVGGKPAKMGEFPWQMAIKEGDKIHCGGIYIGGCWILTAAHCVRI

SRMHRYQIWTSFTDWLRPGFQTVVHSVNRIIIHENYNGTTYQNDIALIEMKKRPNEKECV

LSKSIPACVPWSPYLFQPNDKCIVSGWGREKGTDDGSIDACKGDSGGPLVCQDVNNVTYV

WGVVSWGENCGKSEFPGVYTKVANYFDWISQHVGRSLISQHNI

>tr|A0A3Q1LI40|A0A3Q1LI40_BOVIN Complement component C7 OS=Bos taurus OX=9913 GN=C7 PE=3 SV=1

MKAITLLFLVGFIGEFQVFSSASSPINCQWGSYAPWSECNGCTKTQTRRRSIAVYGQYGG

HSCVGSAFETQPCQPTRGCPTEDGCGERFRCFSGQCISKSLVCNGDSDCEEDGADEDRCE

DAESRPACDKDKPPPNIELTGRGYNALTGQFRNQVLNTKSFGGQCRKVYSGDGRDFYRLS

GNILSYTFQVKINNDFNNEFYNSTWAYVKETSTEHSSSSKGRFLFFSSSSSSHGYSSNTN

ILTKKKSYQLLVLQNTVEVAQFINNNPEFLQLAESFWKELSYLPSLYDYSAYRRLIDQYG

THYLQSGSLGGEYKVIFHMDSEKVKKFDFHSEDKRKCASSHFQFLFTSSKQKCTTMEEVL

KSVSENEGNLLRGVPFVRGGHSGFLAGLSYLDLNNPAGNKRRYSQWAGSVPDLPEVIKQK

LTPLYELVKEVPCASVKKLYLKRAIEEYLDEFDPCHCRPCHNGGMATVQGSQCQCYCKPK

TSGVACEQGVLLGDQAGGVDGGWNCWSSWGPCVQGKKTRSRQCNNPSPSAGGKSCIGETS

ETRQCEDGELEHLRLLEPHCFPLSLVPTKFCSSPPALKDGFVQDEGATFPVGKNIMYTCK

EGYSLVGDPVARCGEDLQWLVGNMHCQKIACVLPALMDGIQSHPHKPFYTIGEKVTISCS

GGRSLEGPSTFLCSSSLKWSPEVKDVQCVQREAPLTPKVPKCQPWEKLQNSRCVCKMPYE

CGSSLGVCARDERSKRILPLTVCKMHVLQCQGRNYTVAGRESCTLPASAEKACGVCPLWE

KCDAEGSECVCRAASECEEAGFSVCVEVNGREQTMTECEAGVLRCRGLSISVTGIRPCAA

EAA

>tr|A0A3Q1LJG6|A0A3Q1LJG6_BOVIN Aldehyde oxidase OS=Bos taurus OX=9913 GN=AOX1 PE=3 SV=1

LSGGAQDLDYTLKRDDLYITEKNVDPETMLLPYLRKKLRLTGTKYGCGGGGCGACTVMIS

RYNPITKKIRHYPANACLTPICSLYGAAVTTVEGIGSTKTRIHPVQERIAKCHGTQCGFC

TPGMVMSLYTLLRNHPEPTLTQLNDALGGNLCRCTGYRPIINACKTFCKTSGCCQSKENG

VCCLDQGINGLPEFEEGNETSLKLFSEEEFLPLDPTQELIFPPELMTMAEKKTQKTRIFG

SDRMTWISPVTLKELLEAKVKYPQAPVVMGNTSVGPDMKFKGIFHPVIISPDRIEELSVV

NYTDNGLTLGAAVSLAEVKDILANVTRKLPEEKTQMYHALLKHLETLAGPQIRNMASLGG

HIVSRHPDSDLNPLLAVGNCTLNLLSKEGRRQIPLNEQFLRKCPSADLKPEEILISVNIP

YSRKWEFVSAFRQAQRQQNALAIVNSGMRVCFGKGDGIIRELSIAYGGVGPTTILANNSC

QKLIGRPWNEEMLDAACRLILDEVSLPGSAPGGRVEFKRTLIVSFLFKFYLEVSQILKGM

DPVHYPSLASKNESALEDLHSRHYWSTLKYQNADPKQLSQDPVGHPIMHLSGIKHATGEA

VYCDDMPVVDRELFLTFVTSSRAHAKIVSIDVSAALSLPGVVDILTGEHLPSITTTFDFL

TDADQLLSTDEVSCVGQLVCAVIADSEVQARRAAQQVKIVYQDLEPVILTIEVMSSRPER

KLEYGNVDEAFKVVDQILEGEIHMGGQEHFYMETQSMLVVPKGEDREIDVYVSAQFPKYI

QDITASVLKVSANKVMCHVKRVGGAFGGKVTKTGVLAAITAFAANKHGRPVRCILERGED

ILITGGRHPYLGKYKAGFMNDGRILALDMEHYNNAGAFLDESLFVIEMGLLKLENAYKFP

NLRCRGWACRTNLPSNTALRGFGFPQAGLITEACITEVAAKCGLPPEKVRMINMYKEIDQ

TPYKQEINTKNLTQCWKECMATSSYTLRKAAVEKFNSENYWKKKGLAMVPLKYPIGLGSV

AAGQAAALVHIYLDGSVLVTHGGIEMGQGVHTKMIQVASRELRMPLSSIHLRGTSTETIP

NTNPSGGSVVADLNGLAVKDACQTLLKRLKPIISKNPKGTWKDWAQAAFNESISLSATGY

FRGYESNINWETGEGHPFEYFVYGAACSEVEIDCLTGAHKNIRTDIVMDVGYSINPALDV

GQIEGAFIQGMGLYTIEELNYSPQGVLYTRGPNQYKIPAICDIPMELHISFLPPSENSNT

LYSSKGLGESGIFLGCSVFFAIHDAIRAARQERGLPGPLRLNSPLTPEKIRMACEDKFTK

MIPRDEPGSYVPWSVPI

>tr|A0A3Q1LJU5|A0A3Q1LJU5_BOVIN EMAP like 2 OS=Bos taurus OX=9913 GN=EML2 PE=3 SV=1

MRLLWAVPDSTERDLGSTNPGPRIVLGGLAGGVPTPSPFLGILGDDPGARPGVGRLMPGG

PSRGCWNEGRCYGNGRQGLGGGDRARAGTGGAGGGCGGAMAERGPAFCGLYDTSSLLQYC

NDDNLSGTSGMEVDDRVSALEQRLQLQEDELAVLKAALADALRRLRACEEQGAALRARGT

PKGRAPPRLGTTASVCQLLKGLPTRTPLNGSGPPRRVGGYATSPSSPKKEATSGRSSARR

YLSPERLASVRREDPRSRTTSSSSNCSAKKEGKTKEVIFSMAEEGSVKMFLRGRPVPMLI

PEELVPTYSLDTRSELPSHRFKLDWVYGYRGRDCRANLYLLPTGEIVYFVASVAVLYSVE

EQRQRHYLGHNDDIKCLAVHPDMVTIATGQVAGTTKEGKPLPPHVRIWDSVSLSTLHVLG

LGVFDRAVCCVGFSKSNGGNLLCAVDESNDHVLSVWDWAKETKVVDVKCSNEAVLVATFH

PTDPTVLITCGKSHVYFWNLEGGSLSKRQGLFEKYEKPKYVLCVTFLEGGDVVTGDSGGN

LYVWGKGGNRITQAVLGAHDGGVFGLCALRDGTLVSGGGRDRRVVLWGSDYSKLQEVEVP

EDFGPVRTVAEGRGDTLYVGTTRNSILQGSVHTGFSLLVQGHMEELWGLATHPSRAQFVT

CGQDKLVHLWSVESHQPLWSRTIEDPARSAGFHPSGSVLAIGTVTGRWLLLDTETHDLVA

IHTDGNEQISVVSFSPDGAYLAVGSHDNLVYVYTVDQGGRKVSRLGKCSGHSSFITHLDW

AQDSSCFVTNSGDYEILYWDSSTCKQITTAETVRNVEWATATCVLGFGVFGIWSEGADGT

DINAVARSHDGKLLASADDFGKVHLFSYPCCQPRALSHKYGGHSSHVTNVAFLWDDSMAL

TTGGKDTSVLQWRVV

>tr|A0A3Q1LJZ4|A0A3Q1LJZ4_BOVIN FAT atypical cadherin 1 OS=Bos taurus OX=9913 GN=FAT1 PE=4 SV=1

MGRHLASLLLLLRLLQHFRDGDGSGTLEEMPLQFTHFQYNVTVHENSAAKTYVGHPVKMG

IYMTNPLWELRYKIISGDNENLFKAEEYVLGDFCFLRIRTKGGNTAILNREVKDHYTLIV

KAVEKNTNAEARTQVRVQVLDTNDLRPLFSPTSYSVSLPENTAIRTSIARVSATDADIGT

NGEFYYSFKDRTDMFAIHPTSGTIVLTGRLDYTETSVYEMEILAVDRGMKLYGSSGISSM

AKLTVHVEQANACAPVITAVTSSPSELDRDPTYAIVTVDDCDQGANGEVASLSIVAGDLL

QQFRTVRSSPGSREYKLKATGAIDWDSHPFGYNLTLQAKDKGTPPQFSSVKVIHVISPRF

KAGPVRFEKEVYRAEISEFAPPHTPVVMVKATPSYPHLKYVFKSTPGKAKFSLNHNTGLI

SILEPLKRQQASHFELEVTTSDRKASTRVLVKVLSANSHPPEFTQTAYKASFDENVPIGT

TVMSVSAVDPDEGENGYVTYSIANLNHVPFVINHFTGAVSTSENLDYELMPRVYTLRIRA

SDWGSPYRREIEILATLTLNNLNDNTPLFEKINCEGTIPRDLGVGEQITTVSAIDADELQ

LVRYQIEAGNELDLFSLNPNSGVLSLKQSLMDGLGAKVSFHSLRITATDGENFATPLYIN

MTVAALRKPVSLQCEETGVAKMLAEKLLQANKLHSQGEVEDVFFDSHSVNAHAPQFRSTL

PAGIQVKENQPVGSSLLVMNATDLDTGFNGKLVYAVSGGNEDSCFIMDMETGMLKILSPL

DRETTDRYTLNITVSDLGLPQRAAWHLLEIRVLDANDNPPEFLQESYFVEVSEDKEINSE

IIQVEATDKDLGPNGHVRYSILTDTDKFSIDSVTGVVKILNPLDREEQQVHYLKVEARDQ

AREEPQLLSTVILKVSLDDVNDNPPKFIPPNYRVKVREDLPEGTIIMWLEAHDPDLGQSS

QVRYSLLDHGEGNFDVDKLSGAVRIIQQLDFEKKQVYNLTVRAKDKGKPISLSSTCYVEV

EVIDVNENLHPPVFPSFVEKGAVKENAPLGSSVMKVSARDEDMGRDGEIHYSIRDGSGVG

VFRIDEETGVIETSDRLDRESTSHYWLTVYASDQGVVPLSSFVEVYIEVEDVNDNAPQTS

EPVYYPEIMENSPKDVSVVQIEAFDPDSSSDDKLTYKITSGNPQGFFSINSKTGLITTTA

RKLDREQQDEHILEVTVTDNGSPPKSTITRVIVKILDENDNKPQFLQKFYKIRLPEREKP

ERERNAKREPLYRVIATDKDEGPNAEISYSIEEGNEHGKFFIEPKTGVVLSKKFSGAGEY

DILSIKAVDNGRPQKSSTARLHIEWISKPKPSLEPISFEESFFTFTVMESDPVAHMIGVI

SVEPPGIPLWFDIIGDTLAREVFYIFPMTCDLNCTAEGGNYDSHFDVDRGTGTIIIAKPL

DAEQKSNYNLTVEATDGTTTILTQVFIKVIDTNDHRPQFSTSKYEVVIPEDTVPETEILQ

ISAVDKDEKNKLIYTLQSSIDPLSLKKFRLDPATGSLYTSEKLDHEAIHQHILTVMVRDQ

DVPVKRNFARIVVNVSDTNDHAPWFTSSSYEGRVYESAAVGSVVLQVTALDKDKGKNAEV

LYSIESGNIGNSFTIDPILGSIKTARELDRSNQVAYDLMVKATDRGDPPMSEITSVRVFV

TIADNASPKFTSKEYSVEISETVGIGSFVGMVTAHSQSSVVYEIKDGNVADAFDINPHSG

SIITQKALDFETLPIYTLTIQGTNMAGLSTNTTVLVHLLDENDNLPVFVQAEYTGLISES

ASINSVVLTDRNVPLVIRATDADRESNALLVYHIVEPSVHKYFTIDSSTGAIHTVLSLDY

EETSTFHFTVQVHDMGTPRLFAEYAANVTIHVIDINDCPPVFSTSLYEASLLLPTYRGVK

VVAVNATDADSSAFSQLMYSITEGNIGEKFLMDRKTGTITVQNTTQLRSRYELTVRASDG

RFASFAAIKINVKESKESQLKFTQDFYSAIVKENSTEARTLAVITALGNPINEPLFYHIL

NPDPRFRISRTSGVLSTTGIPFDREQQEAFDVVVEVTRERKPPAVAHVIVKVVIEDQNDN

APVFVNLPYYALVKVDAAVGQVIRHVTAVDRDSGRNGEVHYFLKERHEHFQIGSSGEISL

KKPFEPDTLNKEYLITVVARDGGDPAFSAEVIVPITVMNKAMPVFEKPFYSAEIPENAQL

HSPVVHVQANSPEGLKVHYSITEGDPFSQFTVNFNTGVISVVAPLDFESHPAYKLSIRAT

DSLTGAHAEVFVDIIVEDINDNPPVFAQQSYATTLSEASVIGTSVVQVRATDADSEPNRG

ISYHMVGNHSKSHDHFHVDSGTGLISLVRTLDYEQFRQHQISVRAVDGGMPPLSSDVVVT

VDVTDLNDHPPLFDQQLYEARISEHAAHGHFVTCVRAYDADSSDADKLEYSILSGNDHKN

FVIDGKTGIITLSNLRRHALKPSYSLQVSASDGVFRSSAQVHVTVIGGNLHSPLFLQNEY

EVELAENAPLHTLVTEVKASDGDSGIYGHVTYHIVNDFAKDRFYTNERGQIFTLEKLDRE

TPAEKVIPIRFMAKDAGGKVAFCTINVILTDDNDNAPQFRATKFEVNIGSSAPKGTSVIK

VLASDADEGSNADITYAIEADSESVKENLEINRASGVITTKESLIGLENEVFTFFVRAVD

NGSPQRESVVPVYVKVLPPEMRLPRFSEPFYTYTVSEDVPIGTEIDLIRAEHSGTVLYSL

VKGNTPESNRDEFFVIDRQSGRLKLEKSLDHETTKWFQFSVLARCTHGDYELVASVDVSI

QVKDTNDNSPVLESSPYEAFIVENLPAGSRVIQIRASDLDSGSNGQVMYNLDQSQSVDVI

ESFAVNMETGWITTLRELDHEKRDSYQIKVVASDHGEKVQLSSTAIVDVTVTDVNDSPPR

FTAEIYKGTVSEDDPPGGVIAIVSTTDADSEEINRQVTYYITGGDPLGQFAIENIQNEWK

VYVKKPLDREERDSYLLTITATDGTFSSKAIVEVKVLDANDNTPVCEKTLYSDTIPEDAF

PGKLIMQVSATDADIRSNAEITYTLFGPGAEKFKLNPDTGELKTSAPLDREEQAAYHLLV

KATDGGGRFCQASVVLTLEDVNDNAPEFSADPYTITVFENTEPGTLLTRVQATDADAGLN

RKISYSLLNSADGQFSINELSGIIQLEKPLDRELQAVYTLTLKAVDHGLPRRLTATGTIV

ISVLDINDNPPVFEYREYGATVSEDILIGTEVLQVYAASRDIEANAEITYSIISGNEHGK

FSIDSKTGAIFIIENLDYESSHEYYLTVEATDGGTPSLSDVATVSINVTDINDNAPVFSQ

DTYTAVVSEDAVLEQSVITVMADDADGPSNSHIHYAIIDGNQGSPFTIDPARGEVKVTKL

LDRETISGYTLTVQASDNGSPPRVTTTTVNIDVSDVNDNAPVFSRGNYSLIIQENKPVGF

SVLQLVVTDRDSSHNGPPFFFSIVSGNEEGAFEVNQQGALLTAAAINRKVKDHYLLHVKV

ADNGKPQLSSLTYIDIRVIEESVYPPAILPLEIFITAFGEEYSGGVIGKIHATDQDVYDT

LTYSLDPQMDSLFSVSSTGGKLIAHKKLDIGQYLLNVSVTDGKFTTAADITVQVRQVTQE

MLNQTVAVRFANLTPEEFVGDYWRNFQRALRNILGVRRNDIQIVSLQPAEPHPHLDVLLF

VERSGVTHVSTRQLLQKINSSVSDVEEIIGVRILEVFRKLCAGLDCPWKFCDEKVSVDES

VMSTHSTARLSFVTPRHRRMAVCLCREGKCPLVHHGCEDNPCPEGSECVTDPREETYTCV

CPGGKSGQCPGSASVTFTGNSFVKYRLMENENKLEMKLTMRLRTYSAHAVVMYARGTDYS

ILEIHNGRLQYKFDCGSGPGIVSVQSIQVNDGLWHAVSLEVNGNYARLVLDQVHTASGTA

PGTLKTLNLDSHVYFGGHVRQQGSRHGRSPQVGNGFRGCMDSIYLNGQELPLNNRPRSYA

HIEESVDVSPGCLLTATEDCSSSPCQNGGVCHPSPTGGYYCKCNTLYVGTYCEVSVNPCS

SNPCLYGGTCIVDNGDFVCQCRGLYSGQRCQLSPYCKDEPCKNGGTCFDSLDGAVCQCDS

GFRGERCQSDVDECAGNPCRNGALCENTHGSYHCNCSHEYKGKHCEDVAPNQYVSTPWNI

GLAEGIGIVVFITGIFLLVLVFVFCRKMISRKKKPQPEPEDKHLGPSAAFLQRPYFDSKL

NKNIYSDIPPQVPVRPISYTPSIPSDSRNNLDRNSFEGSAIPEHPEFSTFNPESMHGHRK

AVAVCSVAPNLPPPPPSNSPSDSDSIQKPSWDFDYDTKVVDLDPCLSKKPLEEKPSQPYS

ARESLSEVHSLSSFQSESCDDNASIVTVIHLVNAVVDMVTEEESLAAPDLSKSRGYHWDT

SDWMPSVPLPDIQEFPNYEVIDEQTPLYSADPNAIDTDYYPGGYDIESDFPPPPEDFPGA

DELPPLPPEFSDQFESIHPPRDAPAAGSPGSASRGRQRFHPNQYLPSFYAVDLSEPQKAG

AGDGSARREPYAPYPAGYPRTFEAPAVESLPLSVYASTASCSDVSACCEAESEVMMSDYE

SGDDGPFEEVTVPPLDSQQHTEV

>tr|A0A3Q1LK49|A0A3Q1LK49_BOVIN Inter-alpha-trypsin inhibitor heavy chain H2 OS=Bos taurus OX=9913 GN=ITIH2 PE=1 SV=1

MILSNFLSQRSISGESGERTEDVDQVTVYSYKVQSTITSRVANTVIQTKVVNHSPQPQNV

VFDVQIPKGAFISNFSMTVDGTTFTSSIKEKTVGRALYAQARAKGKTAGLVRSRALDMED

FKTEVSIAPGAKVQFELHYQEVKWRKLGSYEHRLHLKPGRLAKHLEVDVRIIEPQGLRSL

HVLDTFDGHFDGVPVVSKGPQKAHIAFKPTVAQQRKCLNCSETMVDGELVVMYDVNREEK

VGELEVFNGYFVHFFAPENMDPIPKNILFVIDVSGSMWGIKMKQTVEAMKTILDDLRTED

HFSVVDFNHNVRTWRNDLVSATKTQVADAKNYIEKIQPSGGTNINEALLRAIFILNEANN

LGMLDPNSVSLIILVSDGDPTVGELKLSKIQKNVKQNIRDNISLFSLGIGFDVDYDFLKR

LSNDNRGIAQRIYGNQDTSVQLKKFYNQVSTPLLRNVQFNYPQPSVTDVTQNSFHNYFGG

SEIVVAGKVDPEKLGQLQSIITATSANAELVLETLAEMDGLEDFLSKDKHADPDFTKKLW

AYLTINQLLAERSLAPTAAMKRKITKTILQMSLDHHIVTPLTAMVIENEAGDERMLADSP

PQDHSCCSGALYHGSKVSPNSVPSWVNPSPAPVLPMPAVGAQVLESTPPPHVMRGILVNG

QLVGAKKPKNKKLSTYFGKLGFYFQREDVKVEISTETISLSRGSRTSVLSWSDTAHVLNQ

RVLVSVKKEKTVTVTLDKEMFFSVLLHRVWKKHPINVDFLGIYIPPTNKFSPNVHGLIGQ

FMNEPKIHVFNERPGKDPEKPEASMEVKGQKLVVTRGLQKDYRTDRVFGTDVPCWFVHNS

GKGFIDGHYKDYFVPQLYSFLRRP

>tr|A0A3Q1LKB9|A0A3Q1LKB9_BOVIN Carboxypeptidase B2 OS=Bos taurus OX=9913 GN=CPB2 PE=3 SV=1

MKLYSLGVLVATVLFCGEHAFAFQRGQVLSALPRTSRQVQILQNVTTTYKIVLWQPVAAE

YIVKGYEVHFFVNASDVSNVKAHLNASRIPFRVLVENVEDLIRQQTSNDTISPRASSSYY

EQYHSLNEIYSWIEVMTERYPDMVEKIHIGSSYEKYPLYVLKVSKKEQRAKNAMWIDCGI

HAREWISPAFCLWFVGSDRMWRKNRSLHEKNACVGTDLNRNFASKHWCGEGASSSSCSEI

YCGTYPESEPEVKAVADFLRRNIKHIKAYISMHSYSQKIVFPYSYSRSRSKDHEELSLVA

REAVFAMENIHRNIRYTHGSGSESLYLAPGGSDDWIYDLGIKYSFTFELRDKGKYGFLLP

ESYIRPTCSEALVAVAKIASHVVKNV

>tr|A0A3Q1LKF1|A0A3Q1LKF1_BOVIN Catalase OS=Bos taurus OX=9913 GN=CAT PE=1 SV=1

MADNRDPASDQMKHWKEQRAAQKPDVLTTGGGNPVGDKLNSLTVGPRGPLLVQDVVFTDE

MAHFDRERIPERVVHAKGAGAFGYFEVTHDITRYSKAKVFEHIGKRTPIAVRFSTVAGES

GSADTVRDPRGFAVKFYTEDGNWDLVGNNTPIFFIRDALLFPSFIHSQKRNPQTHLKDPD

MVWDFWSLRPESLHQVSFLFSDRGIPDGHRHMNGYGSHTFKLVNANGEAVYCKFHYKTDQ

GIKNLSVEDAARLAHEDPDYGLRDLFNAIATGNYPSWTLYIQVMTFSEAEIFPFNPFDLT

KGRLFAYPDTHRHRLGPNYLQIPVNCPYRARVANYQRDGPMCMMDNQGGAPNYYPNSFSA

PEHQPSALEHRTHFSGDVQRFNSANDDNVTQVRTFYLKVLNEEQRKRLCENIAGHLKDAQ

LFIQKKAVKNFSDVHPEYGSRIQALLDKYNEEKPKVSPEQPSCGRGCVSWIGTVGFLFSD

SPQGQLFSFLSSCSQSQLRFKDLTWKAQPLHLSPGPYKTLGCVGVCVCVGV

>tr|A0A3Q1LKF4|A0A3Q1LKF4_BOVIN Serine hydroxymethyltransferase OS=Bos taurus OX=9913 GN=SHMT1 PE=3 SV=1

MAAPVNKAPRDANLWSLHEKMLAQPLKDNDVEVYNIIKKESNRQRVGLELIASENFASRA

VLEALGSCLNNKYSEGYPGQRYYGGTEFIDELEVLCQKRALQVYGLDSQCWGVNVQPYSG

NGQVQTSLGGPLPASLLGLPGKESISRTPCQMGEMRSPPSPSTVNPDTGYINYDQLEENA

RLFHPRLIIAGTSCYSRNLDYARLRKIADDNGAYLMADMAHVSGLVAAGVVPSPFEHCHV

VSTTTHKTLRGCRAGMIFYRKGVRSVDPKTGRETRYNLESLINSAVFPGLQGGPHNHAIA

GVAVALKQAMTPEFRAYQRQVVANCRALAEALMGLGYRVVTGGSDNHLILVDLRSKGTDG

GRAEKVLEACSIACNKNTCPGDKSALRPSGLRLGTPALTSRGLLEEDFQKVAHFIHRGIE

LTLQIQDAVGVKATLKEFMEKLAGAEEHQRAVAALRAEVESFATLFPLPGLPGF

>tr|A0A3Q1LKL7|A0A3Q1LKL7_BOVIN Adhesion G protein-coupled receptor E5 OS=Bos taurus OX=9913 GN=ADGRE5 PE=4 SV=1

MGGPHGGPFLLFHVLCFLLTLSEVGSQNSKACALPCPPNSSCVNGTACRCAPGFISFSGE

IFTDPLESCDDINECGPPSPVDCGSSADCQNTEGGYYCTCSPGYEPVSGAMIFRNESENT

CRDVDECQHRPRVCKGRSVCINTEGSYTCQCPPGLEFSPEDPRHCTDVDECSSGQHQCHN

STVCFNTVGSYTCHCREGWEPKHGLKNKQKDTICKEISFPAWTAPPGIKSRSLSAFFERV

QKMSRDFKPAMAKKSMQDLVGSVDDLLKNSGDLESLDQSSKHVTVTHLLSGLEQILRTLA

KAMPKGSFTYRSLDNTELSLVVQEQGKGNVTVGQSHARMLLDWAVAAAAEESGPTVVGIL

SSQNMKKLLANASLKLDSEKLKETYKSPVRGAKVTLLSAVSSVFLSNTNTEKLDSNVSFA

FALHEQPELKPRQELICAFWKKDSNGNGSWATTGCWKMGRGNGSITCQCSHLSSFAILMA

HYDVEDPKLALITKVGLALSLACLLLCILTFLLVRPIQGSRTTVHLHLCICLFVGSAIFL

AGIENEGGEVGTRCRLVAVLLHYCFLAAFCWMSLEGVELYFLVVRVFQGQGLRKLWLCLI

GYGVPLIIVGISAGAYSKGYGREKFCWLNFEGGFLWSFVGPVTFIVLGNAIIFVITVWKL

TQKFSEINPDIKKLKKARVLTITAIAQLFVLGCTWVFGLLLFDPESWVLSYIFSILNCLQ

GFFLFVLYCLLNKKVREEYRKWACMVAGNKYSEFATTTSGSGSSHNQTQALRPSESGM

>tr|A0A3Q1LKR8|A0A3Q1LKR8_BOVIN E1 ubiquitin-activating enzyme OS=Bos taurus OX=9913 GN=UBA1 PE=1 SV=1

MSYKRGPSYMIHEWARPAYIRVSGGWGPRFCSGLGLRGRHLVSAAAEVKEVAGTSRTTTT

REEKSAAVILGGPGGGEEEEEEKEEEGGGRACLRLLEELAAARPGEPALMSSSPLSKKRR

VSGPDPKPGSNCSPAHSVLSEVPSVPANGMAKNVSDADIDEGLYSRQLYVLGHEAMKRLQ

TSSVLVSGLRGLGVEIAKNIILGGVKAVTLHDQGTAQWADLSSQFYLREEDIGKNRAEVS

QPRLAELNSYVPVSAYTGPLVEDFLSDFQVVVLTNSPLEDQLRVGEFCHSHGIKLVVADT

RGLFGQLFCDFGEEMILTDSNGEQPLSAMVSMVTKDNPGVVTCLDEARHGFESGDFVSFS

EVQGMIELNGSQPMEIKVLGPYTFSICDTSNFSDYIRGGIVSQVKVPKKISFKSLPASLA

EPDFVMTDFAKYSRPAQLHIGFQALHHFCAQHGRSPRPHNEEDAAELVTIAQAVNARSLP

AVQQGSLDEDLIRKLAYVAAGDLAPINAFIGGLAAQEVMKACSGKFMPIMQWLYFDALEC

LPEDKEALTEDKCLPRQNRYDGQVAVFGSDLQERLGKQKYFLVGAGAIGCELLKNFAMIG

LGCAEDGEIVVTDMDTIEKSNLNRQFLFRPWDVTKLKSDTAAAAVRQMNPHIRVTSHQNR

VGPDTERIYDDDFFQNLDGVTNALDNVDARMYMDRRCVYYRKPLLESGTLGTKGNVQVVI

PFLTESYSSSQDPPEKSIPICTLKNFPNAIEHTLQWARDEFEGLFKQPAENVNQYLTDPK

FVERTLRLAGTQPLEVLEAVQRSLVLQRPQTWADCVTWACHHWHTQYSNNIRQLLHNFPP

DQLTSSGAPFWSGPKRCPHPLTFDVSNPLHLDYVIAAANLFAQTYGLTGSQDRAAVATLL

QSVQVPEFTPKSGVKIHVSDQELQSANASVDDSRLEELKATLPSPEKLPGFKMYPIDFEK

DDDTNFHMDFIVAASNLRAENYDIPPADRHKSKLIAGKIIPAIATTTAAVVGLVCLELYK

VVQGHRQLNSYKNGFLNLALPFFGFSEPLAAPRHQYYNQEWTLWDRFEVQGLQPNGEEMT

LKQFLDYFKTEHKLEITMLSQGVSMLYSFFMPAAKLKERLDQPMTEIVSRVSKRKLGRHV

RALVLELCCNDESGEDVEVPYVRYTIR

>tr|A0A3Q1LLU1|A0A3Q1LLU1_BOVIN von Willebrand factor OS=Bos taurus OX=9913 GN=VWF PE=4 SV=1

MFPTRLARLLLAVALTLPGALCGEGALGKSSMARCSLFGADFINTFDESMYSFSGDCSYL

LAGDCKTHSFSIVGDFQGGRRMGLSVYLGEFFDIHVFVNGTVLQGGQHVSMPYATRGLYL

ETEVGHHKLSSESYGFVARIDGSGNFQILLSDRHFNKTCGLCGDFNIFAEDDFRTQEGTL

TSDPYDFANSWALSSEEQRCPRVSPPSSSCNVSSELQKGLWEKCQLLKTASVFARCHALV

DPEPFVALCERMLCACAQGLRCPCPVLLEYARACAKQGMLLYGWADHSSCRPDCPAGMEY

KECVSPCHRTCRSLSITEVCREQCVDGCSCPEGQLLDEGRCVESTECPCVHAGKPYPPGA

SLSRDCNTCICRNSQWVCSNEDCPGECLITGQSHFKSFDDRHFTFSGVCQYLLAQDCQDH

SFSVVIETVQCADDPDAVCTRSVTVRLPSPHHGLLKLKHGGGVALDGQDIQIPLLQGDLR

IQHTVTASLQLNFGEDLQIDWDGRGRLLLKLSPVYAGRTCGLCGNYNGNQRDDFLTPAGL

VEPLVEHFGNSWKLRADCEDLQEQPSDPCSLNPRLTKFADQACAILTSPKFEACHSAVSP

LPYLRNCRYDVCACSDGRDCLCDAVANYAAACARRGVHVGWREPSFCALSCTHGQVYQQC

GTPCNLTCRSLSHPDEECTEVCLEGCFCPPGLFLDETGSCVPKAQCPCYYDGEIFQPEDI

FSDHHTMCYCEDGFMHCSTSGAPGSLLPEAVLSSPLSHRSKRSLSCRPPMVKVVCPADNP

RAEGLECTKTCQNYDLECMSTGCVSGCLCPPGMVRHENRCVALERCPCFHQGREYAPGDR

VKVDCNSCVCQDRKWNCTDHVCDASCSALGLAHYFTFDGLKYLFPGECQYVLVQDHCGSN

PGTFRVLVGNEGCSVPSLKCRKRITILVEGGEIELFDGEVNVKTPMKDETHFEVVESGRY

ITVLLGKALSVVWDGHLAVSVFLKRTYQERVCGLCGNFDGVQNNDLTSSSLQVEENPVDF

GNSWKVSPQCADTQKVRLDSAPAICHDNVMKQTMVDSSCRVLTSDVFRECNRLVNPEPYL

DVCIYDSCSCESIGDCSCFCDTIAAYAHECAQHGQVVTWRTATLCPQNCEERNLKESGYQ

CEWRYNSCAPACPVTCQHPEPLACPVQCVEGCHAHCPPGKILDELLQTCVNPEDCPVCQV

EGRRLASGKKVTLNPGDPEHCQICHCDGVSLTCEACKEPGGPPPTEGPVIPTTPYVEDIP

EPPLHDFFCSKLLDLVFLLDGSSKLSEADFETLKAFVVGMMERLHISQKRIRVAVVEYHD

GSHAYLALQDRKRPSELRRIAGQVKYAGSEVASTSEVLKYTLFQIFGRIDRPEASRVALL

LTASQEPPRLARNLVRYVQGLKKKKVSVVPVGIGPHASLKQIRLIEKQAPENKAFVLSGV

HELEQRMDDIVGYLCDLALEVPAPTLTQHPLAAQVTVAPQLLGPSLPGPKRSSVVLDVAF

LLEGSDEVGEANFNRSAEFVEEVIRRMDVGQDGIHVTVLQYSYVVTVEHSFREPQSKDVV

LQRLREVRYRGGNQTNTGLALQYLSEHSFSASQGDREQAPNLVYMVTGSPASDKIQRMPG

DIQLVPIGVGPRVDVQELERVSWPQTPIFIQDFERLPREAPDLVLQRCCSEDGPHLPTLA

PAPDCSQPLDVVLLLDGSSTSPASYFDEMKSFAKAFISKANLGPQLTQVSVLQYGSNTNV

DVPWDIHVDKAHLLSLVDPMHREGGPSQVGQALSFAARYITSQVHGARPSASKVVVILVT

GSSMDSVEAAAAAARSNRVAVFPIGIGDQYDAAQLRVLAGPGASSNVAELQRIEDLPSMV

ALGNSFFQRLCSGFVSVCVDEDGNERRPGDVWTLLDQCHTVTCLPDGQTLLKSHRVNCDQ

GPQPSCPDGQTPLRMEEACGCRWACPCVCTGSSTRHIVTFDGRNFKLTGNCSYVLFHNKE

QDLEVILHNGFCSAGARQACMKSVEVKQNGLSVELRSNMEVMVNGRLVSVPYLGGDMEVR

VYGTIMFEVRFMGHILSFTPQNNEFQLQLRPKIFASKTYGLCGFCDENGANDFMLRDGTV

TEDWKTLVREWTVQQPGQTCPLGPEEPCPISQGSRCQVLLSELFAECHKVLAPATFHAMC

QQDSCQQEQVCEAIASYAHLCRTKGVCVDWRTPDFCAVSCPPSLIYNHCESGCPRQCEGN

SSSCGDHPLEGCFCPPHQVMLEGSCVPEEACTQCVGDDGIRHQLLETWVPDHQPCQICTC

LSGRKVNCTTQPCPSAGVPTCGPCEVARLRQSTQQCCPEYECVCDLVSCDVPPVPNCEDG

LQPVLANPGECRPSFTCVCRKEECPKGSPPSCPPHRMPALRKTRCCDEYECACNCTNSTV

SCPLGYLASTITNDCSCTTTTCLPDKVCVHRGTIYPVGQFWEEGCDTCTCTDLEDAVMGL

RVAQCSQKPCADICRPGFTYVLREGECCGRCLPSACEVVTGSPRGDSQTQWKSVGSHWAS

PENPCLIHECVRVEEEVFVQQRNVSCPQLNVPVCPLGFQLRCQTLGCCPTCRCEPVQACV

LNGTIIGPGKTVMVDACMTCRCHVQAGVISGFKLECRKTTCQACPAGYMEEKLQGECCGR

CLPTACTIQLRGGQIMMLKRDETLQDGCDSHFCKVNKRGEFIWEKRVMSCPPFNEHKCLA

EGGEIMKIPGTCCDTCEEPECKDITARVKSIKVGDCKSEDEVDIHYCQGRCTSKALYSID

TEDVQDQCSCCSPTRTEPMPVPLRCTNGSIIHHVVLNALQCQCSSRKCSP

>tr|A0A3Q1LM32|A0A3Q1LM32_BOVIN Complement component C6 OS=Bos taurus OX=9913 GN=C6 PE=3 SV=1

MARHSVLYFILLSALIDKSQACFCDHYPWSQWSSCSKTCNSGTQTRQRRIVTDKYYFENF

CGQLCTKQESRECNWQTCPINCRLGDYGPWSDCDPCVQKRFKVRSILRPSQFGGQPCTEP

LMTFQPCIPSKLCKIEEIDCKNKFRCDSGRCIASKLECNGENDCGDNSDERNCGRKKTVC

SRSHNPIPGVQLMGMGFHFLAGEPRGEVLDNSFTGGVCRTVKSSRASNPYRVPANLENVN

FEVQTKEDDLEADFYDDLIPLEDNKDQEALGSGLATSSFRVPIFYSSKRSQSSSHSSAFK

QAIQASQKKASSFIRIHKVIKVLNFTMKTKDLQLSDVFLKALNHLPLEYNSALYSRIFDD

FGTHYFTSGSLGGVYDLLYQFSKEELKNSGLTKEEAKNCIRIETKKRFLFVKKTKVEHRC

TTNKLSEKYEGSFMQGSEKSISLVQGGRSAYAAALAWEKGSPVPEERVFSDWLESVKENP

SVIDFALAPITDLVRNIPCAVTRRNNLRRAFREYAAKFDPCQCARCPNSGRPVLSGTECL

CVCQSGTYGENCERRSPDYKSNAVDGNWGCWSSWSSCDATYRRSRTRECNNPAPQQGGKR

CEGERRQEEHCTFSIMQNDGQPCISDDEDMKETDLPELESDSGCPQPVPPENGFIRNEKK

QYSVGEEVEILCFTGFKAVGYQYFRCLPDRSWRQGDVECQRTECLKPIVPEGLTLSPFQT

LYKIGDSIELTCPRGLVVNGPSRYTCSGDSWTPPISDSLSCEKDVLTGLRGHCQPGQKQL

GSECVCMSPEEDCGHYSEEICVLDTTSSDYFTSSACKLLAEKCLNNQQLHFLHIGSCEEG

PQLKWGLERIKLSSSSTKNESCGYDTCYNWEKCSDTLVSTKRTLFGEMKPFCWSAGAQKQ

ENVPVVEYSIIFLSSSPSLEVSRALCQSSGGRTFI

>tr|A0A3Q1LMS5|A0A3Q1LMS5_BOVIN Heat shock cognate 71 kDa protein OS=Bos taurus OX=9913 GN=HSPA8 PE=1 SV=1

MSKGPAVGIDLGTTYSCVGVFQHGKVEIIANDQGNRTTPSYVAFTDTERLIGDAAKNQVA

MNPTNTVFDAKRLIGRRFDDAVVQSDMKHWPFMVVNDAGRPKVQVEYKGETKSFYPEEVS

SMVLTKMKEIAEAYLGKVSTIVCIFAYPTLPAYFNDSQRQATKDAGTIAGLNVLRIINEP

TAAAIAYGLDKKVGAERNVLIFDLGGGTFDVSILTIEDGIFEVKSTAGDTHLGGEDFDNR

MVNHFIAEFKRKHKKDISENKRAVRRLRTACERAKRTLSSSTQASIEIDSLYEGIDFYTS

ITRARFEELNADLFRGTLDPVEKALRDAKLDKSQIHDIVLVGGSTRIPKIQKLLQDFFNG

KELNKSINPDEAVAYGAAVQAAILSGDKSENVQDLLLLDVTPLSLGIETAGGVMTVLIKR

NTTIPTKQTQTFTTYSDNQPGVLIQVYEGERAMTKDNNLLGKFELTGIPPAPRGVPQIEV

TFDIDANGILNVSAVDKSTGKENKITITNDKGRLSKEDIERMVQEAEKYKAEDEKQRDKV

SSKNSLESYAFNMKATVEDEKLQGKINDEDKQKILDKCNEIINWLDKNQTAEKEEFEHQQ

KELEKVCNPIITKLYQSAGGMPGGMPGGMPGGFPGGGAPPSGGASSGPTIEEVD

>tr|A0A3Q1LMT0|A0A3Q1LMT0_BOVIN Phosphopyruvate hydratase OS=Bos taurus OX=9913 GN=ENO3 PE=1 SV=1

MAMQKIFAREILDSRGNPTVEVDLHTAKGRFRAAVPSGASTGIYEALELRDGDKSRYLGK

GEERPRNLTSALPSLNLPFQKLSVVDQEKVDKFMIELDGTENKSKFGANAILGVSLAVCK

AGAAEKGVPLYRHIADLAGNPELILPVPAFNVINGGSHAGNKLAMQEFMILPVGASSFRE

AMRIGAEVYHHLKGVIKAKYGKDATNVGDEGGFAPNILENNEALELLKTAIQAAGYPDKV

VIGMDVAASEFYRNGKYDLDFKSPDDPPHCNFLTHYPFDPFDQDDWATWTSFLSGVNIQI

VGDDLTVTNPKRIAQAVEKKACNCLLLKVNQIGSVTESIQACKLAQSNGWGVMVSHRSGE

TEDTFIADLVVGLCTGQIKTGAPCRSERLAKYNQLMRYRGHSDMLLSILFIG

>tr|A0A3Q1LMV5|A0A3Q1LMV5_BOVIN Glutathione S-transferase OS=Bos taurus OX=9913 GN=GSTP1 PE=3 SV=1

MPPYTIVYFPVQGRCEAMRMLLADQGQSWKEEVVAMQSWLQGPLKASCLYGQLPKFQDGD

LTLYQSNAILRHLGRTLGLYGKDQQEAALVDMVNDGVEDLRCKYVSLIYTNYEAGKEDYV

KALPQHLKPFETLLSQNKGGQAFIVGDQISFADYNLLDLLRIHQVLAPSCLDSFPLLSAY

VARLNSRPKLKAFLASPGAHEPAHQRQWETVRACGTLCARQGAACSSLSPGPIKLPREKQ

>tr|A0A3Q1LMZ4|A0A3Q1LMZ4_BOVIN Neogenin 1 OS=Bos taurus OX=9913 GN=NEO1 PE=3 SV=1

MAAEPGAWRLLSTSFWLCCLLLLGRRAPGVAAARSGSPPQSSGSSIRTFTPFYFLVEPVD

TLSVRGSSVTLNCSAYSEPSPKIEWKKDGTLLNLVSDDRRQLLPDGSLFISSVVHSKHNK

PDEGYYQCVATVESLGTIVSRTAKLTVAGLPRFASQPEPSSVYVGNSAVLNCEVNADLVP

FVRWEQNKQPLLLDDRVIKLPSGMLVISNATEGDGGLYRCMVESGGPPKYSEEAELKVLP

DPGATSNLVFLKQPSSLIRVIGQSAVLPCVASGLPTPSIRWVKNQETVDTESSGRLVLLA

GGSLEIDDVTEDDAGTYFCIADNGNETIEAQAELTVQAQPEFLKQPTNIYAHESMDIVFE

CEVTGKPTPTVKWVKNGDMVIPSDYFKIVKEHNLQVLGLVKSDEGFYQCIAENDVGNAQA

GAQLIILEHDVAIPTLPPTSLTSATTDHLAPATAGPLPSAPRDVVASLVSTRFIKLTWRT

PASDPHGDNLTYSVFYTKEGIARERVENTSRPGEMQVTIQNLMPATVYIFRVMAQNKHGP

GESSAPLRVETQPEVQLPGPAPNIRACATSPTSITVTWETPLSGNGEIQNYKLYYMEKGT

DKEQDVDVSSHSHTINGLKKYTEYSFRVVAYNKHGPGVSTQDVAVRTFSDVPSAAPQNLS

LEVRNSKSIVIHWQPPPPAAQNGEITGYKVRYRKASRKSDVTETLVSGTQLSQLIEGLDR

GTEYNFRVAALTVNGTGPATDWLSAETFESDLDETRVPEVPSSLHVRPLVTSIVVSWTPP

ENQNIVVRGYAIGYGIGSPHAQTIKVDYKQRYYTIENLDPSSHYVITLKAFNNVGEGIPL

YESAVTRPHTVPTSPPKDVTVVSKEGKPRTIIVNWQPPSEANGKITGYIIYYSTDVNAEI

HDWVIEPVVGNRLTHQIQELTLDTPYYFKIQARNSKGMGPMSEAVQFRTPKASGSAGKGS

RLPDLGSDYKPPMSGSNSPHGSPTSPLDSNMLLVIIVSVGVITIVVVVIIAVFCTRRTTS

HQKKKRAACKSVNGSHKYKGNSKDVKPPDLWIHHERLELKPIDKSPDPNPIMTDTPIPRN

SQDITPVDNSMDSNIHQRRNSYRGHESEDSMSTLAGRRGMRPKMMMPFDSQPPQPVISAH

PIHSLDNPHHHFHSSSLASPARSHLYHPGSPWPIGTSMSLSDRANSTESVRNTPSTDTMP

ASSSQTCCTDHQDPEGATSSSYLASSQEEDSGQSLPTAHVRPSHPLKSFAVPAIPPTGPP

TYDPALPSTPLLSQQALNHHIHAVKTASIGTLGRSRPPMPVVVPSAPEVQETTRMLEDSE

SSYEPDELTKEMAHLEGLMKDLNAITTA

>tr|A0A3Q1LP81|A0A3Q1LP81_BOVIN Dihydropyrimidine dehydrogenase [NADP(+)] OS=Bos taurus OX=9913 GN=DPYD PE=3 SV=1

MAPVLSKDVADIESILALNPRTQSRATLRSTLAKKLDKKHWKRNPDKNCFNCEKLENNFD

DIKHTTLGERGALREAMRCLKCADAPCQKSCPTNLDIKSFITSISNKNYYGAAKMIFSDN

PLGLTCGMVCPTSDLCVGGCNLYATEEGPINIGGLQQYATEVFKAMNIPQIRNPSLPPPE

KMPEAYSAKIVLLGAGPASISCASFLARLGYNDITIFEKQEYVGGLSTSEIPQFRLPHDV

VNFEIELMKDLGVKIICGKSLSVNDITLSTLKEEGYKAAFIGIGLPEPKKDHIFQGLTQD

QGFYTSKDFLPLVAKSSKAGMCACHSPLLSIRGTVIVLGAGDTAFDCATSALRCGARRVF

IVFRKGFVNIRAVPEEVELAREEKCEFLPFLSPRKVIVKGGRIVAMQFVRTEQDETGKWN

EDEDQIVCLKADVVISAFGSVLSDPKVKEALSPIKFNRWDLPEVDPETMQTSEPWVFAGG

DVVGIANTTVEAVNDGKQASWYIHRYIQSQYGASVSAKPELPLFYTPIDLVDISVEMAGL

KFTNPFGLASATPTTSSSMIRRAFEAGWAFALTKTFSLDKDIVTNVSPRIIRGTTSGPMY

GPGQSSFLNIELISEKTAAYWCQSVTELKADFPDNIVIASIMCSYNRNDWMELSRKAEAS

GADALELNLSCPHGMGERGMGLACGQDPELVRNICRWVRQAVRIPFFAKLTPNVTDIVSI

ARAAKEGGANGVTATNTVSGLMGLKADGTPWPAVGREKRTTYGGVSGTAIRPIALRAVTT

IARALPEFPILATGGIDSAESGLQFLHGGASVLQKLPSFGPYLEKRKKIIAEEKLRLKKE

NVTVLPLERNHFIPKKPIPSVKDVIGKALQYLGTYGELNNTEQVVAVIDEEMCINCGKCY

MTCNDSGYQAIQFDPETHLPTVTDTCTGCTLCLSVCPIIDCIKMVSRTTPYEPKRGLPLA

VNPVS

>tr|A0A3Q1LPF0|A0A3Q1LPF0_BOVIN Apolipoprotein E OS=Bos taurus OX=9913 GN=APOE PE=3 SV=1

MKVLWVAVVVALLAGCQADMEGELGPEEPLTTQQPRGKDSQPWEQALGRFWDYLRWVQTL

SDQVQEELLNTQVIQELTALMEETMKEVKAYKEELEGQLGPMAQETQARVSKELQAAQAR

LGSDMEDLRNRLAQYRSEVQAMLGQSTEELRARMASHLRKLRKRLLRDADDLKKRLAVYQ

AGASEGAERSLSAIRERFGPLVEQGQSRAATLSTLAGQPLLERAEAWRQKLHGRLEEVGV

RAQDRLDKIRQQLEEVHAKVEEQGNQMRLQAEAFQARLRSWFEPLVEDMQRQWAGLVEKV

QLALRPSPTSPPSENH

>tr|A0A3Q1LPT9|A0A3Q1LPT9_BOVIN STEAP3 metalloreductase OS=Bos taurus OX=9913 GN=STEAP3 PE=3 SV=1

MPSEHLPEFCVPSQSQAAYGSGGDSRKNGWPDHSGAVRGRMRQILASACVATRTSGKMDK

PLISHHLVDSDGSLAEAPSEVPKVGILGSGDFARSLATRLVGSGFSVVVGSRNPKRMAGL

FPSAAQVTLQEEAVGSPEVIFVAMFREHYSTLCGLSDQLAGKILVDVSNPTEQEHLQHHQ

SNAEYLASLFPTCAVVKAFNVISAWTLQSGPRDGNRQVPICSDQPEAKRTVLEMVRAMGF

TPVDMGSLASAREVEAMPLRLFPGWKVPALLALGLFIFFYAYNFVRDVLEPYVQEGKNKF

YKLPVFVVNTTLPCVAYVLLSLVYLPGVLAAALQLRRGTKYQRFPDWLDHWLQHRKQIGL

LSFFCAALHALYSLCLPLRRSHRYDLVNLAIKQVLTNKSHLWNEEEVWRMEIYLSLGVLA

LGMLSLLAVTSLPSIANSLNWREFSFVQSTLGFVALVLSTLHTLTYGWTRAFEESRYKFF

LPPTFTLTLLVPCVIILAKGLFLLPCISRRLTKIRRGWEKDGSVRFTLPVDHALAQKTSH

V

>tr|A0A3Q1LPX4|A0A3Q1LPX4_BOVIN Hydroxymethylglutaryl-CoA synthase OS=Bos taurus OX=9913 GN=HMGCS1 PE=1 SV=1

MPGSLPLNAEACWPKDVGIVALEIYFPSQYVDQAELEKYDGVDAGKYTIGLGQAKMGFCT

DREDINSLCMTVVQNLMERNSLSYDCIGRLEVGTETIIDKSKSVKTNLMQLFEESGNTDI

EGIDTTNACYGGTAAVFNAVNWIESSSWDGRYALVVAGDIAVYATGNARPTGGVGAVAML

IGPNAPLIFERGLRGTHMQHAYDFYKPDMLSEYPIVDGKLSIQCYLSALDRCYSVYRKKI

RARWQKEGNDRDFTLNDFGFMIFHSPYCKLVQKSLARMLLNDFLNDQNRDKNSIYSGLEA

FGDVKLEDTYFDRDVEKAFMKASSELFNQKTKASLLVSNQNGNMYTSSVYGSLASVLAQY

SPQQLAGKRIGVFSYGSGLAATLYSLKVTQDATPGSALDKIVASICDLKSRLDSRTCVAP

DIFAENMKLREDTHHLANYIPQSSIDSLFEGTWYLVRVDEKHRRTYARRPSLNDDTLNEG

VGLVHPSTTAEHIPSPAKKVPRLPATAAEPEAAVISNGEH

>tr|A0A3Q1LQ21|A0A3Q1LQ21_BOVIN Inter-alpha-trypsin inhibitor heavy chain H3 OS=Bos taurus OX=9913 GN=ITIH3 PE=3 SV=1

MALAQWPYLILALLSGLAVSGFPRNPSLLLGKRSLPGRAVDGIEVYSTKVNCKVTSRFAH

NVVTTRAVNHANTAKEVSFDVELPKTAFITNFTLTIDGVTYPGKVKEKEVAKKQYEKAVS

QGKTAGLVKASGRKLEKFTVSVNVAAGSKVTFELTYEELLKRHKGKYEMYLKVQPKQLVK

HFEITVDIFEPQGISTLDAEASFITNDLLGSALTKSFSGKKGHVSFKPSLDQQRSCPTCT

DSLLKGDFIITYDVNRESPANVQIVNGYFVHFFAPQGLPVVPKSVVFVIDVSGSMHGRKM

EQTKDALLKILEDVKQDDYLNFILFSGDVTTWKDSLVPATPENIQEASKFVMDIQDRGMT

NINDALLRGISMLNKAREEHTVPERSTSIIIMLTDGDANVGESRPEKIQENVRNAIGGKF

PLYNLGFGNNLNYNFLENMALENHGLARRIYEDSDANLQLQGFYEEVANPLLTGVEVEYP

QNAILDLTQNSYQHFYDGSEIVVAGRLADEDMNSFKAAVKGHGAINDLTFTEEVDMKEME

KALQERDYIFGDYIERLWAYLTIEQLLDKRKNAQGEEKEILTAQALELSLKYHFVTPLTS

MVVTKPEDNENQTAIANKPGEGRHRRWGATTSYQAPQTPYYYVDGDPHFIIQIPEKDDAI

CFNIDEDPGTVLRLIQDPVTGLTVNGQIIGEKTGRSDSQTRRTYFGKLGIASAQMDFRIE

VTRENITLWNGDSLSTFSWLDTVMVTQDGLSVMINRKKNMVVSFGDGVTFVVVLHQVWKK

EPAHHDFLGFYVVNSRGMSAQTHGLLGQFFHPFDFQVSDVHPGSDPTKPDATMVVKNHQL

TVTRGSQKDYRKDISVGRNVACWFVHNNGQGLIDGIHRDYIVPNLF

>tr|A0A3Q1LQI3|A0A3Q1LQI3_BOVIN Plexin B2 OS=Bos taurus OX=9913 GN=PLXNB2 PE=3 SV=1

MALQLWALALLGLAGTSASLRPRKLDSFRSETELNHLVVDEASGMVYVGAVNMLYQLSAD

LQLEQRVATGPALDNKKCTPPIEVSQCHEAVQTDNVNQLLLLDPPRSRLVECGSLFKGIC

ALRALGNISTRLFYEDGSGEKSFVASNDESVATVGLVSAADPGGERLLFVGKGNGPHDNG

VIVSTRLLDRTEGREAFEAYTDHATYKAGYMSSNTQQFVAAFEDGPYVFFVFNQQDKHPA

RNRTLLARMCKQDPFYYSYLEVDLSCLDPGDAQAPAFGTCLAASLDRPGSGGVLYAVFST

DGRGGRGPRTGLCLFPLDEVHRRMEANRDACYTGAREVARDTFYKPFHGDIQCGGHVSVR

GLSVLTGAWATCGGVCAWPCLTCVHMPPCCEGCVRLHGCVGFPRAGQRGHPHGCLFSQVL

GAPGVGAWSSEHPGALVWPGWFWGMCAAECWGPSRERGLGQALTPWSAGCQQEFPVWLGA

LALPAGQPRRPLGQSRAAPWRPEPDGRDRDHRERPHHRLSGHLGWPGPQGVLRPGWQLHG

VRRHPRGDQQENQERPGAGRRPGQPVRHDPGQGVPASRAGVCQLLQLRSLPELTGPLLRL

VCRRGPVSAQGLGPAQNLGVQPCPSPGADGHQSKTAPRPRSCTRKAECPRADESGHWLWS

RNESCVAVTGAHPQNMSRQAQGEVQLTVSPLPALRDDDTLLCLFGGSPPHPARLQEGAVV

CNSPSRSSLPRTPPGQDHVAVTIRLHFKRGNVFLTSHQYPFYDCREAMSLQENLPCISCS

SNRWTCQWALLEHACQQASPGPEDGVVGAHMEDDCPQFLNPSPLVIPVNHETDVTFQGKN

LDTVQGSPLLVGSDLLKFEALVSTQEPGTFSFRTPKLTHDANETLPLHLYVKSAGKNVDS

RLQVTLYNCSFGRSDCSLCLAADPAYRCVWCSGRSRCVYEALCSNATSECPPPVVTRIQP

ETGPLGGGIRVTILGSNLGVRADDVKRVTVAGQNCAFEPERYSVSTRIVCTIEAAEEPCT

GGVEVDINGKLGHSPPHAQFTYQQPQPHSVEPKQGPQAGGTTLTINGTHLDTGSEEDVRV

TLSDVPCNVTQFGAQLQCVTGPQAVPGELALKIYYGGSEVPNPGITFTYRENPVLRAFEP

LRSFVSGGRSINVTGQGFSLIQRFAMVVIAEPLQSWRRRRETGPLRSVTVVGTEYVFYND

SKVVFLSPAVPEEPEAYNLTALIRMDGHQALLRTEAGAFEYVADPTFENFTGGVKKQVNK

LIHARGTNLNKAMTIHEAEAFVGAERCIMKTLTETDLYCEPPEVQPPPKRRQKRDTAHNL

PEFIVKFGSREWVLGRVEYDTRASDVPLSLILPLVIGPMVAVIAVSVYCYWRKSQQAERE

YEKIKSQLEGLEESVRDRCKKEFTDLMIEMEDQTNDVHEAGIPVLDYKTYTDRVFFLPSK

DGDKDVMITGKLDIPESRRQVVEQALYQFSNLLNSKCFLINFIHTLENQREFSARAKVYF

ASLLTVALHGKLEYYTDIMRTLFLELMEQYVVAKNPKLMLRRSETVVERMLSNWMSICLY

QYLKDSAGEPLYKLFKAIKHQVEKGPVDAVQKKAKYTLNDTGLLGDDVEYTPLTVSVIVQ

DEGVDAVPVKVLNCDTISQVKEKIIDQVYRTQPCSRWPKADSVVLEWRPGSTAQILSDLD

LTSQREGRWRRVNTLMHYNVRDGATLILSKVGVSQQPEDSQQDLPGERHALLEEENRVWH

LVRPTDEVDEGKSKRGSVKEKERTKAITEIYLTRLLSVKGTLQQFVDNFFQSVLAPGNAV

PPAVKYFFDFLDEQAEKHDIKDEDTIHIWKTNSLPLRFWVNILKNPHFIFDVHVHEVVDA

SLSVIAQTFMDACTRTEHKLSRDSPSNKLLYAKEISTYKKMVEDYYKGIRQMVQVSDQDM

NTHLAEISRAHTDSLNTLVALHQLYQYTQKYYDEIINALEEDPAAQKMQLAFRLQQIAAA

LENKVTDL

>tr|A0A3Q1LQY7|A0A3Q1LQY7_BOVIN STEAP3 metalloreductase OS=Bos taurus OX=9913 GN=STEAP3 PE=3 SV=1

MDKPLISHHLVDSDGSLAEAPSEVPKVGILGSGDFARSLATRLVGSGFSVVVGSRNPKRM

AGLFPSAAQVTLQEEAVGSPEVIFVAMFREHYSTLCGLSDQLAGKILVDVSNPTEQEHLQ

HHQSNAEYLASLFPTCAVVKAFNVISAWTLQSGPRDGNRQVPICSDQPEAKRTVLEMVRA

MGFTPVDMGSLASAREVEAMPLRLFPGWKVPALLALGLFIFFYAYNFVRDVLEPYVQEGK

NKFYKLPVFVVNTTLPCVAYVLLSLVYLPGVLAAALQLRRGTKYQRFPDWLDHWLQHRKQ

IGLLSFFCAALHALYSLCLPLRRSHRYDLVNLAIKQVLTNKSHLWNEEEVWRMEIYLSLG

VLALGMLSLLAVTSLPSIANSLNWREFSFVQSTLGFVALVLSTLHTLTYGWTRAFEESRY

KFFLPPTFTLTLLVPCVIILAKGLFLLPCISRRLTKIRRGWEKDGSVRFTLPVDHALAQK

TSHV

>tr|A0A3Q1LRD1|A0A3Q1LRD1_BOVIN Phosphoglucomutase-1 OS=Bos taurus OX=9913 GN=PGM1 PE=3 SV=1

MVKIVTVKTKAYQDQKPGTSGLRKRVKVFQSSSNYAENFIQSIISTVEPAQRQEATLVVG

GDGRFYMKEAIQLIVRIAAANGIGRLVIGQNGILSTPAVSCIIRKIKAIGGIILTASHNP

GGPNGDFGIKFNISNGGPAPEAITDKIFQISKTIEEYAICPDLHVDLGVLGKQQFDLENK

FKPFTVEIVDSVEAYATMLRNIFDFNALKELLSGPNRLNIRIDAMHGVVGPYVKKILCEE

LGAPANSAVNCVPLEDFGGHHPDPNLTYAADLVETMKTGEHDFGAAFDGDGDRNMILGKH

GFFVNPSDSVAVIAANIFSIPYFQQTGVRGFARSMPTSGALDRVANATKIALYETPTGWK

FFGNLMDASKLSLCGEESFGTGSDHIREKDGLWAVLAWLSILATRKQSVEDILKDHWQKY

GRNFFTRYDYEEVEAEGANKMMKELEALISDRSFVGKQFPVGDKVYTVEKIDNFEYSDPV

DGSISRNQGLRLLFADGSRIIFRLSGTGSAGATIRLYIDSYEKDLAKIYQDPQVMLAPLI

SIALKVSQLQEKTGRTAPTVIT

>tr|A0A3Q1LRL7|A0A3Q1LRL7_BOVIN Xylulose kinase OS=Bos taurus OX=9913 GN=XYLB PE=1 SV=1

MAERAARHCCLGWDFSTQQVKVVAVDAELSVFYEDSVHFDRDLVEFGTQGGVHVHKDGLT

VTSPVLMWVQALDIILEKMKASGFDFSQVLALSGAGQQHGSVYWKTGASQVLTSLSPDLP

LREQLQACFSISNCPVWMDSSTAAQCRQLEAAVGGAQALSLLTGSRAYERFTGNQIAKIY

QQNPEAYSHTERISLVSSFAASLFLGSYSPVDYSDGSGMNLLQIQDKVWSQACLGACAPR

LEEKLGRPVPSCSIVGAISSYFVQRYGFPPECKVVAFTGDNPASLAGMRLEEGDIAVSLG

TSDTLFLWLQEPTPALEGHIFCNPVDPQHYMALLCFKNGSLMREKIRDESASGSWSKFSK

ALQSTGMGNSGNLGFYFDVMEITPEIIGRHRFTAENHEVSAFPQDVEIRALIEGQFMAKK

IHAEALGYRVMPKTKILATGGASHNRDILQVLADVFGAPVYVIDTANSACVGSAYRAFHG

LAAGTDMPFAEIVKLAPNPRLVATPTPGASQVYEALLQRYAMLEQRVLSQTRGPPE

>tr|A0A3Q1LS55|A0A3Q1LS55_BOVIN Alpha-1-acid glycoprotein OS=Bos taurus OX=9913 GN=ORM1 PE=3 SV=1

MALLWALAVLSLLPLLDAQSPECANLMTVAPITNATMDLLSGKWFYIGSAFRNPEYNKSA

RAIQAAFFYLEPRHAEDKLITREYQTIEDKCVYNCSFIKIYRQNGTLSKVESDREHFVDL

LLSKHFRTFMLAASWNGTKNVGVSFYADKPEVTQEQKKEFLDVIKCIGIQESEIIYTDEK

KVLSHLE

>tr|A0A3Q1LS65|A0A3Q1LS65_BOVIN Homogentisate 1,2-dioxygenase OS=Bos taurus OX=9913 GN=HGD PE=1 SV=1

MVAFVSCHMQYISGFGNECASEDPRCPGALPEGQNNPQVCPYNLYAEQLSGSAFTCPRST

NKRSWLYRILPSVSHKPFEFIDQGHITHNWDEVDPDPNQGPRLAAFTFPSCAGLSQGLHT

LCGAGDIRSNNGLAIHIFLCNTSMGNRCFYNSDGDLLIVPQKGKLLIYTEFGKMLVQPNE

ICVIQRGMRFSIDVFEETRGYILEVYGVHFELPDLGPIGANGLANPRDFLIPVAWYEDRQ

VPGGYTVINKYQGKLFAAKQDVSPFNVVAWHGNYTPYKYNLENFMVINSVAFDHADPSIF

TVLTAKSVRPGVAIADFVIFPPRWGVADKTFRPPYYHRNCMSEFMGLIKGHYEAKQGGFL

PGGGSLHSPMTPHGPDANCFEKASKAKLAPERIADGTMAFMFESSLNIAVTKWGLKTSNC

LDENYYKCWEPLKSRFTPNSRNPAETN

>tr|A0A3Q1LS74|A0A3Q1LS74_BOVIN Complement factor H OS=Bos taurus OX=9913 GN=CFH PE=4 SV=1

MRFPAKIVWLVLWTICVAEDCKEPPPRKETEILSGSWTEQTYQEGTQATYKCRPGYRTLG

SIVMMCRGGKWVSLHPSRICRKKPCAHPGDTPFGSFHLAEGNQFEYGAKVVYTCDEGYQM

VGEMNFRECDTNGWTNDIPICEVVKCLPVTEPENGKIFSDALEPDQEYTYGQVVQFECNS

GYMLDGPKQIHCSAGGVWSAETPKCVEIFCKTPVILNGQAVLPKATYKANERVQYRCAAG

FEYGQRGDTICTKSGWTPAPTCIEITCDPPRIPNGVYRPELSKYRGQDKITYECKKGFFP

EIRGTDATCTRDGWVPVPRCAWKPCSYPVIKHGRLYYSYRGYFPARVNQQFVYSCDHHFV

PPSQRSWDHLTCTAEGWSPEEPCLRQCIFNYLENGHTQHREEKYLQGETVRVRCYEGYSL

QNDQNTMTCTESGWSPPPRCIRVKTCSKSNIRIENGFLSESTFTYPLNKQTEYKCKPGYV

TADGKTSGLITCLKNGWSAQPVCIKSCDRPVFEKARVKSDGTWFRLNDRLDYECVDGYEN

RDGRTTGSIVCGQDGWSDKAACYERECSIPEMDPYLNAYPRKETYKVGDVLKFSCSQGRI

MVGADSVQCYHFGWSPKLPTCKVKKVKSCALPPELPNGKRKEIHKEEYAHNEVVEYACNP

RFLMKGSHKIQCVDGEWTALPVCIEEERTCGNIPDLDHGDVKPSVPPYHHGDSVEFSCRE

AFTMIGPRFITCISGEWTQPPQCIATDELRKCKGSTLFPPEGRPAHKIEYDHNTNKSYQC

RGKSEHKHSICINGEWDPKVDCNEEAKIQLCPPPPQVPNACDMTTTVNYQDGEKISILCK

ENYLIQDAEEIVCKDGRWQSIPRCIEKIGCSQPPQIDHGTINSSSSAEERREIHEQRLYA

HGTKLSYTCEEGFEISENNVIICHMGKWSSPPQCVGLPCGLPPYIQNGVISHKKDSYQYG

EEVTYDCDEGFGTDGPASIRCLGGEWSRPQDCISTNCVNLPTFEDAVLTDREKDFYRSGE

QVAFKCLSYYQLDGSNTIQCIKSKWIGRPACRDVSCGNPPQVENAIIHNQKSKYQSEERA

RYECIGNYDLFGEMEVVCLNGTWTEPPQCKDSQGKCGPPPPIDNGDITSLLQSVYPPGMI

VEYRCQAYYELRGNKNVVCRNGEWSQLPKCLEACVISEETMRKHHIQLRWKHDKKIYSKT

EDTIEFMCQHGYRPLTPKHTFRATCREGKVVYPRCG

>tr|A0A3Q1LSB6|A0A3Q1LSB6_BOVIN ATP-citrate synthase OS=Bos taurus OX=9913 GN=ACLY PE=3 SV=1

MSAKAISEQTGKELLYKYICTTSAIQNRFKYARVTPDTDWARLLQDHPWLLSQSLVVKPD

QLIKRRGKLGLIGVNLTLDGVKSWLKPRLGQEATVGKATGFLKNFLIEPFVPHTQEEEFY

VCIYATREGDYVLFHHEGGVDVGDVDAKAQKLLVGVDEKLNPEDIKKHLLVHAPEDKKEI

LASFISGLFNFYEDLYFTYLEINPLVVTKDGVYVLDLAAKVDATADYICKVKWGDIEFPP

PFGREAYPEEAYIADLDAKSGASLKLTLLNPKGRIWTMVAGGGASVVYSDTICDLGGVNE

LANYGEYSGAPSEQQTYDYAKTILSLMTREKHPDGKILIIGGSIANFTNVAATFKGIVRA

IRDYQGPLKEHEVTIFVRRGGPNYQEGLRVMGEVGKTTGIPIHVFGTETHMTAIVGMALG

HRPIPNQPPTAAHTANFLLNASGSTSTPAPSRTASFSESRTDEVAPAKKAKPAMLQDSVP

SPRPLQGKSATLFSRHTKAIVWGMQTRAVQGMLDFDYVCSRDEPSVAAMVYPFTGDHKQK

FYWGHKEILIPVFKNMADAMKKHPEVDVLINFASLRSAYDSTMETMNYAQIRTIAIIAEG

IPEALTRKLIKKADQKGVTIIGPATVGGIKPGCFKIGNTGGMLDNILASKLYRPGSVAYV

SRSGGMSNELNNIISRTTDGVYEGVAIGGDRYPGSTFMDHVLRYQDTAGVKMIVVLGEIG

GTEEYKICRGVTEGRITKPVVCWCIGTCAAMFSSEVQFGHAGACANQASETAVAKNQALK

EAGVFVPRSFDELGEIIQSVYEDLVARGVIVPAQEVPPPTVPMDYSWARELGLIRKPASF

MTSICDERGQELIYAGMPITEVFKEEMGIGGVLGLLWFQKRLPKYSCQFIEMCLMVTADH

GPAVSGAHNTIICARAGKDLVSSLTSGLLTIGDRFGGALDAAAKMFSKAFDSGIIPMEFV

NKMKKEGKLIMGIGHRVKSINNPDMRVQILKDYVRQHFPATPLLDYALEVEKITTSKKPN

LILNVDGLIGVAFVDMLRHCGSFTREEADEYIDIGALNGIFVLGRSMGFIGHYLDQKRLK

QGLYRHPWDDISYVLPEHMSM

>tr|A0A3Q1LSP4|A0A3Q1LSP4_BOVIN Complement C8 alpha chain OS=Bos taurus OX=9913 GN=C8A PE=3 SV=1

LFAGTLFLLDKMTVFRLFRRVSRAVQSFTPTAVSCQLDNWAEWTDCFPCQDKKYRYRSLL

QPNKFGGTICSGNVWDQASCHSPTACLSQAQCGQDFQCKETGRCLKRHLVCNGDKDCLDG

SDEDDCEDVRILENDCSQYDPIPGSEKAALGYNILTQEEAQHVYDARYYGGQCETVYNGE

WRELQYDPACERLYYGDDDKYFRKPYNFLKYHFEAQADTKISSEIYNDANDLLTKVKNDK

SVSSGLTIGVGIRGVPVTVTAGVSMSKDAAFLKKLSKYHEKKYSFMRIFTKVQTAHFKMR

RENIVLDEGMLQSLMELPERYHYGMYAKFINDYGTHYITSGSMGGVYEYILVLNREKMET

AGVTSAEIQKCFGVSLGIEYEYSEAIQIKGSSSLGPCKKSGDGKLTENEKAMGVEDFISR

VRGGSSGWGGSLTQDSSLVTYRSWGRSLKYNPAVIDFEMKPIHEILQHTNLGSLETKRQN

LRRALDKYLMEFNACRCGPCFNNGEPILEGTSCKCQCPVGHQGLACEQMQSEGAQADGRW

SCWSSWSACRSGTQERRRECNNPAPQNGGASCPGHRVQTQAC

>tr|A0A3Q1LT55|A0A3Q1LT55_BOVIN Coagulation factor XI OS=Bos taurus OX=9913 GN=F11 PE=4 SV=1

MTLLYQMVHFALFASVAGECVTTLFQDACFKGGDITVAFAPNAKHCQIICTHHPRCLLFT

FMTESSSEDPTKWYTCILKDSVTETLPMVNMTGAISGYSSKQCLHHISACSKDMYVDLNM

KGMNYNSSLAQSARECQQRCTDDTHCHFFTFATRHFPSIKDRNTCLLKNTQTGTPTSITK

LHEVVSGFSLKSCGLSNLACIRDIFPRTAFVDITIDTVMAPDPFVCRSICTHHPSCLFFT

FLSEEWPTASERNLCLLKTSSSGLPSARFRKNRAFSGFSLQHCQHSVPVFCHSSFYRNTD

FLGEELDIVDADSHEACQKTCTNSIRCQFFTYSPSQESCNGGKGKCYLKLSANGSPTKIL

HGTGSISGYTLRLCKMDNVTADILIFQVKKELRVCTTKIKTRIVGGTQSVHGEWPWQITL

HVTSPTQRHLCGGAIIGNQWILTAAHCFNEVKSPNVLRVYSGILNQSEIKEDTSFFGVQE

IIIHDQYEKAESGYDIALLKLETAMNYTDSQWPICLPSKGDRNVMYTECWVTGWGYRKLR

DKIQNTLQKAKVPLMTNEECQAGYREHRITSKMVCAGYREGGKDACKGDSGGPLSCKHNE

VWHLVGITSWGEGCGQRERPGVYSNVVEYLDWILEKTQGP

>tr|A0A3Q1LTB9|A0A3Q1LTB9_BOVIN Coagulation factor XIII A chain OS=Bos taurus OX=9913 GN=F13A1 PE=3 SV=1

MSESSWTAFGGRRAIPPNTSNAAENDPPTVELQGLVPRGFNPQDYLNVTNVHLFKERWDS

NKVDHHTDKYSNDKLIVRRGQSFYIQIDFNRPYDPTRDLFRVEYVIGLYPQENKGTYIPV

PLVSELQSGKWGAKVVMREDRSVRLSVQSSADCIVGKFRMYVAVWTPYGVIRTSRNPETD

TYILFNPWCEEDAVYLENEKEREECVLNDIGVIFYGDFNDIKSRSWSYGQFEDSILDACL

FVMDKANMDLSGRGNPIKVSRVGSAMINAKDDEGVIAGSWDNVYAYGVPPSAWTGSVDIL

LEYKSSQKPVRYGQCWVFAGVFNTFLRCLGIPARVVTNYFSAHDNDANLQLDIFLEEDGK

RELQTHQGFGVVNSDLVYVTAKKDGTHVVEALDTTHIGKLIVTKEIGGDGMKDITDTYKF

QEGQEEERLALETAMMYGAKKALNTEGVLKSKSDVRMNFEVENAVLGRDLKVIITFRNNG

SARYTVTAYLSGNISFYTGVSKAEFKNKTFEVTLEPLSFKREEVLIGAGEYMGQLLEQAF

LHFFVTARVNETRDVLAKQKSIALTVPKVVIKVRGAQVVGSDMVVTVEFTNPLKETLRNV

WIRLDGPGVTKPLRKMFREIRPNSTVQWEELCRPWVSGPRKLIASLTSDSLRHVYGELDL

QIQRRPSM

>tr|A0A3Q1LTF3|A0A3Q1LTF3_BOVIN Histidine ammonia-lyase OS=Bos taurus OX=9913 GN=HAL PE=3 SV=1

MPRYTVHVRGEWLAVPCQDAQLTVGWLGREAVRRYIKNKPDNGGFASVDDARFLVRRCKG

LGLLDNEDPLDVALEDNEFVEVVIEGDAMSPDFIPSQPEGVYLYSKYREPEKYIALDGDS

LTTEDLVSLGKGHYKIKLTPTAEKRVQKSREVIDRIVEEKTVVYGITTGFGKFARTVIPV

SKLEELQFNLVRSHSSGVGKPLSPERCRMLLALRINVLAKGYSGISLGTLKQVIEVFNAS

CLPYVPEKGTVGASGDLAPLSHLALGLIGEGKMWSPKSGWADAKYVLAAHGLKPIVLKPK

EGLALINGTQMITSLGCEAVERASAIARQADIVAALTLEVLKGTTKAFDTESHRFCDRVQ

DAYTLRCCPQVHGVVNDTIAFVKNIITTEINSATDNPMVFASRGETISGGNFHGEYPAKA

LDYLAIGVHELASISERRIERLCNPSLSELPAFLVAEGGLNSGFMIAHCTAAALVSENKA

LCHPSSVDSLSTSAATEDHVSMGGWAARKALRVIEHVEQVLAIELLAACQGIEFLRPLKT

TTPLEKVYDLVRSVVRPWIKDRFMAPDIEAAHRLLVEQKVWEVAAPYIEKYRMEHIPESR

PVSPTAFSLEFLHKKSTKIPESEDL

>tr|A0A3Q1LTP0|A0A3Q1LTP0_BOVIN Adenosine kinase OS=Bos taurus OX=9913 GN=ADK PE=1 SV=1

MTQIVTMVENILFGMGNPLLDISAVVDKDFLDKYSLKPNDQILAEEKHKELFDELVKKFK

VEYHAGGSTQNSIKVAQWMIQQPHKAATFFGCIGIDKFGEILKKKAAEAHVDAHYYEQNE

QPTGTCAACITGGNRSLVANLAAANCYKKEKHLDMEKNWMLVDKARVYYIAGFFLTVSPE

SVLKVAHHASENNRIFTLNLSAPFISQFYKESLMKVMPFVDILFGNEMEAAAFAREQGFE

TEDIKEIARKTQALPKVNSKRQRIVIFTQGREDTILATESEVTAFAVLDQDQTEIVDTNG

AGDAFVGGFLSQLVSDKPLTECIRAGHYAASVIIRRTGCTFPEKPDFH

>tr|A0A3Q1LU13|A0A3Q1LU13_BOVIN Dihydropteridine reductase OS=Bos taurus OX=9913 GN=QDPR PE=1 SV=1

LAAAAGKLSMALLCVRHCKKSVEVTIATAGVSLSRFTYHLCVFSGPLFQVTAEVGKLLGT

EKVDAILCVAGGWAGGNAKSKSLFKNCDLMWKQSVWTSTISSHLATKHLKEGGLLTLAGA

RAALDGTPGMIGYGMAKAAVHQLCQSLAGKSSGLPPGAAAVALLPVTLDTPVNRKSMPEA

DFSSWTPLEFLVETFHDWITEKNRPSSGSLIQVVTTEGKTELTAASP

>tr|A0A3Q1LUD3|A0A3Q1LUD3_BOVIN Dihydropyrimidine dehydrogenase [NADP(+)] OS=Bos taurus OX=9913 GN=DPYD PE=3 SV=1

MAPVLSKDVADIESILALNPRTQSRATLRSTLAKKLDKKHWKRNPDKNCFNCEKLENNFD

DIKHTTLGERGALREAMRCLKCADAPCQKSCPTNLDIKSFITSISNKNYYGAAKMIFSDN

PLGLTCGMVCPTSDLCVGGCNLYATEEGPINIGGLQQYATEVFKAMNIPQIRNPSLPPPE

KMPEAYSAKIVLLGAGPASISCASFLARLGYNDITIFEKQEYVGGLSTSEIPQFRLPHDV

VNFEIELMKDLGVKIICGKSLSVNDITLSTLKEEGYKAAFIGIGLPEPKKDHIFQGLTQD

QGFYTSKDFLPLVAKSSKAGMCACHSPLLSIRGTVIVLGAGDTAFDCATSALRCGARRVF

IVFRKGFVNIRAVPEEVELAREEKCEFLPFLSPRKVIVKGGRIVAMQFVRTEQDETGKWN

EDEDQIVCLKADVVISAFGSVLSDPKVKEALSPIKFNRWDLPEVDPETMQTSEPWVFAGG

DVVGIANTTVEAVNDGKQASWYIHRYIQSQYGASVSAKPELPLFYTPIDLVDISVEMAGL

KFTNPFGLASATPTTSSSMIRRAFEAGWAFALTKTFSLDKDIVTNVSPRIIRGTTSGPMY

GPGQSSFLNIELISEKTAAYWCQSVTELKADFPDNIVIASIMCSYNRNDWMELSRKAEDP

ELVRNICRWVRQAVRIPFFAKLTPNVTDIVSIARAAKEGGANGVTATNTVSGLMGLKADG

TPWPAVGREKRTTYGGVSGTAIRPIALRAVTTIARALPEFPILATGGIDSAESGLQFLHG

GASVLQVCSAIQNQDFTIIQDYCTGLKALLYLKSIEELQDWDGQSPATKSHQKGKPVPCI

AELVGKKLPSFGPYLEKRKKIIAEEKLRLKKENVTVLPLERNHFIPKKPIPSVKDVIGKA

LQYLGTYGELNNTEQVVAVIDEEMCINCGKCYMTCNDSGYQAIQFDPETHLPTVTDTCTG

CTLCLSVCPIIDCIKMVSRTTPYEPKRGLPLAVNPVS

>tr|A0A3Q1LUD9|A0A3Q1LUD9_BOVIN Protein diaphanous homolog 1 OS=Bos taurus OX=9913 GN=DIAPH1 PE=1 SV=1

MEPPGGGLGPGRGTRDKKKGRNPDELPSAGGDGGKSKKFLERFTSIRSKKEKEKPNSAHR

NSSAFYGDDPTAQSLQDVSDEQVLVLFEQMLLDMNLNEEKQQPLREKDIIIKREMVSQYL

HTSKAGMNQKESSRSAMMYIQELRSGLRDMSLLSCLESLRVSLNNNPVSWVQTFGAEGLA

SLLDILKRLHDEKEETAGGYDSRNKHEIIRCLKAFMNNKFGIKTMLETEEGILLLVRAMD

PAVPNMMIDAAKLLSALCILPQPEDMNERVLEAMTERAEMDEVERFQPLLDGLKSGTSIA

VKVGCLQLINALITPAEELDFRVHIRSELMRLGLHQVLQDLREIENEDMRVQLNVFDEQG

EEDSYDLKGRLDDIRMEMTDFSEVFQILLNTVKDSKAEQHFLSILQHLLLVRNDYEARPQ

YYKLIEECISQIVLHKNGADPDFKCRHLQIDIEGLIDQMIDKTKVEKSEAKATELEKKLD

SELTARHELQVEMKKMESDFEQKLQDIQGEKDALDSEKQKIATEKQDLEAEVSQLTGEVA

KLSKELEDAKKEVASLSAAVTAVAPPSSATVTPAPPLPGDSGPVIPPLSVPTPPLPEARV

SPPPPPPPPPLPGGGGIPPPPPPPLPGGVSIPPPPPPLPGGVSIPPPPPLPGGPGLPPPP

PPLPGGPGLPPPPPPLPGGPGLPPPPPPLPPPPLPGGPGLPPPPPPLPGGPGLPPPPPPL

PGGPGMLPPPPPFPGGIPLPPPFPGGPGIPPPLPFGVPAAPVLPFGLTPKKLYKPEVQLR

RPNWSKFVAEDLSQDCFWTKVKEDRFENSELFAKLTSTFSAQTKTSKVAKKDQEGGEEKK

SLQKKKVKELKVLDSKTAQNLSIFLGSFRMPYHEIKNVILEVNEAVLTESMIQNLIKQMP

EPEQLKMLSELKDEYDDLAESEQFGVVMGAVPRLRPRLNAILFKLQFGEQVENIKPEIVS

VTAACEEVRKSENFSSLLEITLLVGNYMNAGSRNAGAFGFNISFLCKLRDTKSTDQKMTL

LHFLAELCENDHPEVLKFPDELAHVEKASRVSAENLQKNLDQMKKQISDVERDIQNFPAA

TDEKDKFVEKMTIFVKDAQEQYNKLRMMHSNMEALYKELGEYFLFDPKKLSVEEFFMDLH

NFKNMFVQAVKENQKRRETEEKMRRAKLAKEKAEKERLEKQQKREQLIDMNAEGDETGVM

DSLLEALQSGAAFRRKRGPRQGVRKAVCAATSQLVSELTKEDAMTCVPAKMPKSEEVPTI

LEETTELLGRAS

>tr|A0A3Q1LUE9|A0A3Q1LUE9_BOVIN Ig-like domain-containing protein OS=Bos taurus OX=9913 PE=1 SV=1

MSTMAWSPLLLTLVALCTGSWAQAVLTQPSSVSGSLGQRVSITCSGSSSNVGRGNYVNWF

QQIPGSAPRTLIYGATSRASGVPDRFSGSRSGNTATLTISSLQAEDEADYFCAAYDSSSN

NGTVLQARGEVRQKPALPQVMGLPGLKHLLPEQPLLLFA

>tr|A0A3Q1LUJ8|A0A3Q1LUJ8_BOVIN Sodium/potassium-transporting ATPase subunit alpha OS=Bos taurus OX=9913 GN=ATP1A1 PE=1 SV=1

MGGMPHSAARPGPSGGFLGSSPHSGPRSLREAKAEVGRDKYEPAAVSEHGDKKKAKKERD

MDELKKEVSMDDHKLSLDELHRKYGTDLSRGLTTARAAEILARDGPNALTPPPTTPEWVK

FCRQLFGGFSMLLWIGAVLCFLAYGIQAATEEEPQNDNLYLGVVLSAVVIITGCFSYYQE

AKSSKIMESFKNMVPQQALVIRNGEKMSINAEEVVVGDLVEVKGGDRIPADLRIISANGC

KVDNSSLTGESEPQTRSPDFTNENPLETRNIAFFSTNCVEGTARGIVVYTGDRTVMGRIA

TLASGLEGGQTPIAAEIEHFIHIITGVAVFLGVSFFILSLILEYTWLEAVIFLIGIIVAN

VPEGLLATVTVCLTLTAKRMARKNCLVKNLEAVETLGSTSTICSDKTGTLTQNRMTVAHM

WFDNQIHEADTTENQSGVSFDKTSATWLALSRIAGLCNRAVFQANQDNLPILKRAVAGDA

SESALLKCIEVCCGSVKEMRERYTKIVEIPFNSTNKYQLSIHKNANAGEPRHLLVMKGAP

ERILDRCSSILIHGKEQPLDEELKDAFQNAYLELGGLGERVLGFCHLLLPDEQFPEGFQF

DTDDVNFPVDNLCFVGLISMIDPPRAAVPDAVGKCRSAGIKVIMVTGDHPITAKAIAKGV

GIISEGNETVEDIAARLNIPVSQVNPRDARACVVHGSDLKDMTPEQLDDILKYHTEIVFA

RTSPQQKLIIVEGCQRQGAIVAVTGDGVNDSPALKKADIGVAMGIAGSDVSKQAADMILL

DDNFASIVTGVEEGRLIFDNLKKSIAYTLTSNIPEITPFLIFIIANIPLPLGTVTILCID

LGTDMVPAISLAYEQAESDIMKRQPRNPQTDKLVNERLISMAYGQIGMIQALGGFFTYFV

IMAENGFLPNHLLGIRVTWDDRWINDVEDSYGQQWTYEQRKIVEFTCHTAFFVSIVVVQW

ADLVICKTRRNSVFQQGMKNKILIFGLFEETALAAFLSYCPGMGVALRMYPLKPTWWFCA

FPYSLLIFVYDEVRKLIIRRRPGGWVEKETYY

>tr|A0A3Q1LUY6|A0A3Q1LUY6_BOVIN GDP-mannose pyrophosphorylase A OS=Bos taurus OX=9913 GN=GMPPA PE=1 SV=1

MLKAVILIGGPQKGTRFRPLSFEVPKPLFPVAGVPMIQHHIEACAQVPGMQEILLIGFYQ

PDEPLTRFLEAAQQEFNLPIRYLQEFAPLGTGGGLYHFRDQILAGSPEAFFVLNADVCSD

FPLSAMLDVHRHQPHPFLLLGTTANRTQSLNYGCIVENPQTHEVLHYVEKPSTFVSDIIN

CGIYLFSPEALKPLRDVFQRNQQDGQLEDSSGLWPGAGTIRLEQDVFSALAGQGQIYVHL

TDGIWSQIKSAGSALYASRLYLSQYQLTHPERLAKHTPGGPRIRGDWIWGPPRPMVVLSP

QLGPNVSIGEGVTIGEGVRLRESIVLHGATLQEHTCVLHSIVGWGSTVGRWARVEGTPND

PNPNDPRAHMDSESLFKDGKLLPAITILGCRVRIPAEVLILNSIVLPHKELSRSFTNQII

L

>tr|A0A3Q1LVC7|A0A3Q1LVC7_BOVIN Ezrin OS=Bos taurus OX=9913 GN=EZR PE=1 SV=1

MFLNHQLYGSFDDIFPSLPVLVMLPCFSCLHNYPFGFLHSTLSSWACLQPFARLPSLLRS

LKLLYIAFSPITCRLGVGSPGAAPRPWGFTDLWINVRVTTMDAELEFAIQPNTTGKQLFD

QVVKTIGLREVWYFGLQYVDNKGFPTWLKLDKKVSAQEVRKESPLQFKFRAKFYPEDVAE

ELIQDITQKLFFLQVKEGILSDEIYCPPETAVLLGSYAVQAKFGDYNKELHKAGYLGSER

LIPQRVMDQHKLTRDQWEDRIQVWHAEHRGMLKDSAMLEYLKIAQDLEMYGINYFEIKNK

KGTDLWLGVDALGLNIYEKDDKLTPKIGFPWSEIRNISFNDKKFVIKPIDKKAPDFVFYA

PRLRINKRILQLCMGNHELYMRRRKPDTIEVQQMKAQAREEKHQKQLERQQLETEKKRRE

TVEREKEQMMREKEELMLRLQDYEEKTRKAEKELSDQIQRALKLEEERKRAQEEAGRLEA

DRLAALRAKEELERQAADQIKSQEQLATELAEYTAKIALLEEARRRKENEVEEWQLRAKE

AQDDLVKTREELHLVMTAPPPPPVYEPVNYHVHEGPQEEGTELSAELSSEGILDDRNEEK

RITEAEKNERVQRQLMTLTSELSQARDENKRTHNDIIHNENMRQGRDKYKTLRQIRQGNT

KQRIDEFEAM

>tr|A0A3Q1LVC8|A0A3Q1LVC8_BOVIN Xaa-Pro aminopeptidase 1 OS=Bos taurus OX=9913 GN=XPNPEP1 PE=3 SV=1

MAASRKAPRVRANYQDFQLKNLRIIEPNELTYSGDPGVETDSRMAPKITSELLRQLRQAM

RNLEYVTEPIQAYIIPSGDAHQSEYIAPCDCRRAFVSGFDGSAGTAIVTEEHAAMWTDGR

YFLQAAKQMDSNWTLMKMGLKDTPTQEDWLVSVLPEGSRVGVDPLIIPTDYWKKMAKVLR

SAGHHLIPVKDNLVDKIWTDRPERPCKPLITLGLDYTGLFNLRGSDVEHNPVFFSYAILG

LETIMLFIDGDRIDAPIVKEHLLLDLGLEAEYRIQVLPYKSILSELKILCASLSPREKVW

VSDKASYAVSEAIPKDHRCCMPYTPICIAKAVKNSAESEGMRRAHIKDAVALCELFNWLE

KEVPKGGVTEISAANKAEEFRRQQADFVDLSFPTISSTGPNGAIIHYAPVPETNRTLSLD

EVYLIDSGAQYKDGTTDVTRTMHFGTPTAYEKECFTYVLKGHIAVSAAVFPTGTKGHLLD

SFARSALWDSGLDYLHGTGHGVGSFLNVHEGPCGISYKTFSDEPLEAGMIVTDEPGYYED

GAFGIRIENVVLVVPVKTKYNFNNRGSLTFEPLTLVPIQTKMIDVDSLTDKECDWLNSYH

LTCRDVIGKELQKQGRQEALEWLIRETQPISKQP

>tr|A0A3Q1LVM5|A0A3Q1LVM5_BOVIN Inter-alpha-trypsin inhibitor heavy chain H1 OS=Bos taurus OX=9913 GN=ITIH1 PE=1 SV=1

MGLRGLLCVCLVSLLALQAVAAQGSPTRNPKGGKAVDGVVIRSLKVNCKVTSRFAHYIIT

SQVVNSADTAKEVSFNVEIPKTAFISDFAITADENAFTGDIKDKVTAWKQYRKAAISGEN

AGLVRASGRTMEQFSIHIIVGPRSKATFRLTYEEVLRRKLMQYDIVIKVKPQQLVQHFEI

DVDIFEPQGIRKLDVEASFLPKELAAQLIKKSFSGKKGHVLFRPTVSQQQTCPTCSTTLL

NGDFKVTYDVNRDDACDLLVANNYFAHFFAPQNLKKLNKNVVFVIDISSSMEGQKLKQTK

EALHKILGDMRPGDYFDLVLFGSAVQSWKGSLVQASPANLEAARNFVQQFSLAGATNLNG

GLLRGIEILNKAQQSLPELSNHASILIMLTDGEPTEGVMDRTQILKNVRDGIKGRFPLYN

LGFGHDVDLNFLEVMSLENNGRVQRIYEDHDATQQLQGFYEQVANPLLRDVELLYPREAV

SDLTQHRHKQYYEGSEIMVAGRIADHKLSSFKADVRAHGEGQEFMTTCLVDKEEMKKLLR

ERGHMLENHVERLWAYLTIQELLAKRMKLEGQEKANVSAKALQMSLAYQFVTPLTSMTVR

GMTDQDGLEPIIDKPLDDYLPLEMVGPRKTFMLQASQPAPTHSSLDIKKLPDQVTGVDTD

PHFLIHVPQKEDTLCFNINEEPGVVLSLVQDPDTGFSVNGQLIGNEAGSPGKHEGTYFGR

LGIANPATDFQLEVTPQNITLNPGSGGPVFSWRDQAFLRQNEVLVTINRKRNLVVSVEDG

GTFEVVLHRVWRGSAVRQDFLGFYVLDSHRMSARTHGLLGQFFHPFDYKVSNLHPGSDPT

KTDATMVVKNRRLTVTRGLQKDYRKDPRHGAEVTCWFIHNNGDGLIDGIHTDYIVPDIF

>tr|A0A3Q1LVU1|A0A3Q1LVU1_BOVIN Ferritin OS=Bos taurus OX=9913 GN=FTH1 PE=3 SV=1

MTTASPSQVRQNYHQDSEAAINRQINLELYASYVYLSMSYYFDRDDVALKNFAKYFLHQS

HEEREHAERLMKLQNQRGGRIFLQDIKKPDRDDWENGLTAMECALCLERSVNQSLLELHK

LATEKNDPHVSTLTPGSKWRRQLPEGLGRLTNNFLARSLFQLCDFIETHYLNEQVEAIKE

LGDHITNLRKMGAPGSGMAEYLFDKHTLGHSES

>tr|A0A3Q1LVV7|A0A3Q1LVV7_BOVIN Fibrinogen alpha chain OS=Bos taurus OX=9913 GN=FGA PE=4 SV=1

MGTGRGGSLPFFQLGAPLESALSTAEMFSVRDLCLVLSLVGAIKNTKCPSGCRMKGLIDE

VDQDFTSRINKLRDSLFNYQKNSKDSNTLTKNIVELMRGDFAKANNNDNTFKQISEDLRS

RIEILRRKVIEQVQRINVLQKNVRDQLVDMKRLEVDIDIKIRSCKGSCSRALEHKVDLED

YKNQQKQLEQVIAINLLPSRDIQYLPLIKMSTITGPVPREFKSQLQEAPLEWKALLEMQQ

TKMVLETFGGDGHARGDSVSQGTGLAPGSPRKPGTSSIGNVNPGSYGPGSSGTWNPGRPE

PGSAGTWNPGRPEPGSAGTWNPGRPEPGSAGTWNPGRPEPGSAGTWNPGRPEPGSAGTWN

TGSSGSSSFRPDSSGHGNIRPSSPDWGTFREEGSVSSGTKQEFHTGKLVTTKGDKELLID

NEKVTSGHTTTTRRSCSKVITKTVTNADGRTETTKEVVKSEDGSDCGDADFDWHHTFPSR

GNLDDFFHRDKDDFFTRSSHEFDGRTGLAPEFAALGESGSSSSKTSTHSKQFVSSSTTVN

RGGSAIESKHFKMEDEAESLEDLGFKGAHGTQKGHTKARPARDCYDVLQTRPSGAQSGIF

SIKLPASSKIFSVYCDQETGLGGWLLIQQRMDGSLNFNRTWQDYKRGFGSLNDKGEGEFW

LGNEYLHLLTLRGSILRVELEDWAGKGAYAEYHLRVGSEAEDYALEVSSYKGTAGDALIE

GSVEEGTEYTSHTGMRFSTFDRDADKWEDNCAEVYGGGWWYNNCQAANLNGIYYPGGSYD

PRDNSPYEIENGVVWVPFRGADYSLRAVRMKIRPLVTQ

>tr|A0A3Q1LW12|A0A3Q1LW12_BOVIN Phosphatidylinositol-glycan-specific phospholipase D OS=Bos taurus OX=9913 GN=GPLD1 PE=3 SV=1

MQEKSTLVRFWSGLLMLLGFLCPRSSPCGISTHIEIGHRALEFLHLQDGSINYKELLLRH

QDAYQAGSVFPDSFYPSICERGQFHDVSESTHWTPFLNASVHYIRKNYPLPWDEDTEKLV

AFLFGITSHMVADVNWHSLGIEQGFLRTMAAIDFHNSYPEAHPAGDFGGDVLSQFEFKFN

YLSRHWYVPAEDLLGIYKELYGRVVITKKAIVDCSYLQFLEMYAEMLAISKLYPTYSVKS

PFLVEQFQEYFLGGLEDMAFWSTNIYHLTSYMLKNGTSNCNLPENPLFITCGGQQNNTHG

SKVQKNGFHKNVTAALTKNIGKHINYTKRGVFFSVDSWTMDSLSFMYKSLERSIREMFIG

SSQPLTHVSSPSASYYLSFPYTRLGWAMTSADLNQDGYGDLVVGAPGYSHPGRIHVGRVY

LIYGNDLGLPPVDLDLDKEAHEILEGFQPSGRFGSAVAVLDFNVDGVPDLAVGAPSVGSE

KLTYTGAVYVYFGSKQGQLSSSPNVTISCQDTYCNLGWTLLAADVNGDSEPDLVIGSPFA

PGGGKQKGIVAAFYSGSSYSSQEKLNVEAANWMVKGEEDFAWLGYSLHGVNVNNRTLLLA

GSPTWKDTSSQGHLFRTRDEKQSPGRVYGYFPPICQSWFTISGDKAMGKLGTSLSSGHVM

VNGTRTQVLLVGAPTQDVVSKVSFLTMTLHQGGSTRMYELTPDSQPSLLSTFSGNRRFSR

FGGVLHLSDLDNDGSDEIIVAAPLRITDATAGLMGEEDGRVYVFNGKQITVGDVTGKCKS

WVTPCPEEKAQYVLISPEAGSRFGSSVITVRSKEKNQVIIAAGRSSLGARLSGVLHIYRL

GQD

>tr|A0A3Q1LW84|A0A3Q1LW84_BOVIN Serine/threonine-protein phosphatase 2A 65 kDa regulatory subunit A alpha isoform OS=Bos taurus OX=9913 GN=PPP2R1A PE=4 SV=1

MAAADGDDSLYPIAVLIDELRNEDVQLRLNSIKKLSTIALALGVERTRSELLPFLTDTIY

DEDEVLLALAEQLGTFTTLVGGPEYVHCLLPPLESLATVEETVVRDKAVESLRAISHEHS

PSDLEAHFVPLVKRLAGGDWFTSRTSACGLFSVCYPRVSSAVKAELRQYFRNLCSDDTPM

VRRAAASKLGEFAKVLELDNVKSEIIPMFSNLASDEQDSVRLLAVEACVNIAQLLPQEDL

EALVMPTLRQAAEDKSWRVRYMVADKFTELQKAVGPEITKTDLVPAFQNLMKDCEAEVRA

AASHKVKEFCENLSADCRENVIMTQILPCIKELVSDANQHVKSALASVIMGLSPILGKDS

TIEHLLPLFLAQLKDECPEVRLNIISNLDCVNEVIGIRQLSQSLLPAIVELAEDAKWRVR

LAIIEYMPLLAGQLGVEFFDEKLNSLCMAWLVDHVYAIREAATSNLKKLVEKFGKEWAHA

TIIPKVLAMSGDPNYLHRMTTLFCINVLSEVCGQDITTKHMLPTVLRMAGDPVANVRFNV

AKSLQKIGPILDNSTLQSEVKPVLEKLTQDQDVDVKYFAQEALTG

>tr|A0A3Q1LW96|A0A3Q1LW96_BOVIN Inter-alpha-trypsin inhibitor heavy chain H1 OS=Bos taurus OX=9913 GN=ITIH1 PE=1 SV=1

MGLRGLLCVCLVSLLALQAVAAQGSPTRNPKGGKKRMAVDAAVDGVVIRSLKVNCKVTSR

FAHYIITSQVVNSADTAKEVSFNVEIPKTAFISDFAITADENAFTGDIKDKVTAWKQYRK

AAISGENAGLVRASGRTMEQFSIHIIVGPRSKATFRLTYEEVLRRKLMQYDIVIKVKPQQ

LVQHFEIDVDIFEPQGIRKLDVEASFLPKELAAQLIKKSFSGKKGHVLFRPTVSQQQTCP

TCSTTLLNGDFKVTYDVNRDDACDLLVANNYFAHFFAPQNLKKLNKNVVFVIDISSSMEG

QKLKQTKEALHKILGDMRPGDYFDLVLFGSAVQSWKGSLVQASPANLEAARNFVQQFSLA

GATNLNGGLLRGIEILNKAQQSLPELSNHASILIMLTDGEPTEGVMDRTQILKNVRDGIK

GRFPLYNLGFGHDVDLNFLEVMSLENNGRVQRIYEDHDATQQLQGFYEQVANPLLRDVEL

LYPREAVSDLTQHRHKQYYEGSEIMVAGRIADHKLSSFKADVRAHGEGQEFMTTCLVDKE

EMKKLLRERGHMLENHVERLWAYLTIQELLAKRMKLEGQEKANVSAKALQMSLAYQFVTP

LTSMTVRGMTDQDGLEPIIDKPLDGMEMVGPRKTFMLQASQPAPTHSSLDIKKLPDQVTG

VDTDPHFLIHVPQKEDTLCFNINEEPGVVLSLVQDPDTGFSVNGQLIGNEAGSPGKHEGT

YFGRLGIANPATDFQLEVTPQNITLNPGSGGPVFSWRDQAFLRQNEVLVTINRKRNLVVS

VEDGGTFEVVLHRVWRGSAVRQDFLGFYVLDSHRMSARTHGLLGQFFHPFDYKVSNLHPG

SDPTKTDATMVVKNRRLTVTRGLQKDYRKDPRHGAEVTCWFIHNNGDGLIDGIHTDYIVP

DIF

>tr|A0A3Q1LWV8|A0A3Q1LWV8_BOVIN Ig-like domain-containing protein OS=Bos taurus OX=9913 PE=1 SV=1

MAWSPLLLTLVALFTGSWAQAVLTQPSSVSGSLGQRVSITCSGSSSNVGRGNYVNWFQQI

PGSAPRTLIYGATSRASGVPDRFSGSRSGNTATLTISSLQAEDEADYFCAAYDSSSNNGT

VLQARGEVRQKPALPQVMGLPGLKHLLPEQPLLLFA

>tr|A0A3Q1LX69|A0A3Q1LX69_BOVIN L-xylulose reductase OS=Bos taurus OX=9913 GN=DCXR PE=4 SV=1

MDLRLAGRRALVTGAGKGIGRSIVKALHAAGARVVAVSRTQADLDSLVRECPGVETVCVD

LADWEATEQALGGVGPVDLLVNNAAVAFLQPFLEVTKEAYDMSFNVNLRAVIQVSQIVAR

GLIARGAPGVIVNVSSQASQRGLTNHSVYSHRVDPLPVPVQIRVNAVNPTVVMTPMGQAA

WSDPQKAKAMLDRIPLGRFAEVENVVDTILFLLSDRSSMTTGSTVPVDGGFLAT

>tr|A0A3Q1LXH8|A0A3Q1LXH8_BOVIN Urocanate hydratase OS=Bos taurus OX=9913 GN=UROC1 PE=1 SV=1

MCSLQELCSGLPLRPLPENRGRRAGVPHAPVRTPGLSPAEEQLALKNALRYFPPDVQEVL

APEFAQELRLYGHIYMYRFCPDLEMRAYPVERYPCRTRAAAAIMLMIMNNLDPAVAQFPQ

ELVTYGGNGQVFSNWAQFWLTMSYLAQMTEEQTLVMYSGHPLGLFPSSPEAPRLVITNGM

VIPNYSSRTEYEKLFAMGVTMYGQMTAGSYCYIGPQGIVHGTVLTVLNAGRRYLGLQDLA

GKVFVTSGLGGMSGAQAKAAVIVGCIGVIAEVDGAALMKRHRQGWLMEVTDSLDRCIERL

REARKRKEVLSLGYHGNVVDLWERLVHELDTTGELLVDLGSDQTSCHNPFNGGYYPVQLG

FAEAQSLMASDPTAFKSLVQESLRRHVSAINRLAREHFFFWDYGNAFLLEAQRAGADVGK

PGANRTEFRYPSYVQHIMGDIFSQGFGPFRWVCTSGDPQDLAVTDRLATSALEEIVAAGV

TPAVKLQYVDNIRWIQEAAKHQLVVGSQARILYSDQKGRVAIAVAFNQAIARGELKAPVV

LSRDHHDVSGTDSPFRETSNIYDGSAFCADMAVQNFVGDAFRGATWIALHNGGGVGWGEV

INGGFGLVLDGTEDAEQKARRVLSWDVANGVSDRQHPPRLSPVQTADPPPSGKVCSPPAA

AVSPPATARVALHGRQPALHPGCVLSSLCPWGLGSVPFPTLRLCASESTAAHARVCPDAV

CGAGPHPRGTTYQGPKGEA

>tr|A0A3Q1LXM2|A0A3Q1LXM2_BOVIN Collagen type XV alpha 1 chain OS=Bos taurus OX=9913 GN=COL15A1 PE=4 SV=1

MAPRYDSLPRAPAPAPYRPPHLSPSTTSCRRDAQCWRLLLLLSTSALLPGVTRTRSATES

ASRGPLDLTELVGVPLPSSVSFVAGYGGFPAYSFGPGANVGRPARTLIPSTFFRDFAISV

RVKPSSPQGGVLFAITDAFQKVIYLGLRLSGVEDGRQRVILYYTEPSSQVSREAAAFLVP

VMTHKWNYFAVVVQGEEVTLLVDCEEHGRVPFPRSSQALTFEPSAGIFVGNAGATGLERF

IGSIQQLIIHPDPRTPEEMCEAEDSSASGETSGLQETDGVAEILEAVTYTQAPSKEAEVE

PIHTPPTPSPASEDAELSGEPVPEGTKETSNLSADPHSSPEQGSGEILNDTLEAVQTVDG

SPTADTGSGDGVFLHVTEESPYTEDLAATAAAGEAKVPISTAWEAEASSVPTGGLTLSMP

IEDPGEGVTLGAENEEGSAATAAGEAEEPVSIAREAEASSVPTGGLTLPMPTQDPGEMVT

LSPISEEGSTTAAAATDVPLGTFEEEEASGVPTNDLAFITPTVASEQGVTSGPGDEDLAA

ATTEEPLTAAGADTLGSTPPEGPPLPLPSVAPESGAPPGEAEEGFPGPPGPAVPTEPTVE

VEAEGSGLGWGLDVSSGSGDLVHSEELLRGPPGPPGPPGLPGIPGKPGTDVFMGPPGSPG

EDGPTGEPGPPGPEGKPGLDGASGLPGIKGEKGARGPNGSVGEKGDPGNRGLPGPPGKTG

QVGAPGVMGPPGPPGPPGPPGPGCTTGLGFEDTEGSGSIRHLHEPISGPTASSGPKGEKG

DRGPKGDRGMDGASIVGPPGPRGPPGRIEVLSSSLTNITQGFMNFSDIPELVGPPGPRGP

KGDTGVPGFPGLKGEQGEKGEPGAILTGDIPLERLRGQKGEPGEHGAPGPMGPKGPPGHK

GEFGLPGRPGRPGLNGLKGAKGDRGVMIPGPPGLPGPPGPPGPPGAVINIKGAVFPVPVR

PHCKTPVGTTYSGNSELITFHGVKGEKGSWGLPGSKGEKGDQGPQGPPGPPVDPAYLRHF

LNSLKGENGDRGIKGEKGDSNSGFSGFSVSGPPGLPGSPGLVGQKGEAVVGPQGPPGAPG

LPGPPGFGRPGSPGPPGPPGPPGPPAILGAAVALPGPPGPPGQPGLPGSRNLVTTFSNMD

DMLQKAHLVIEGTFIYLKDSTEFFIRVRDGWKKLQLGELIPIPDDSPPPPALSSNPHQPQ

LSLTSISNVNYGRPALHLVALNTPFSGDIRADFQCFQQARAAGLLSTYRAFLSSHLQDLS

TVVRKAERYSLPIVNLKGQVLFNNWDSIFSGHGGQFNTHIPIYSFDGRDVMTDPSWPQKV

IWHGSSTHGVRLVDQYCEAWRTADMAVMGLASPLNTGKILDQKAYSCANRLIVLCIENSF

MTDARK

>tr|A0A3Q1LXP4|A0A3Q1LXP4_BOVIN Beta-2-glycoprotein 1 OS=Bos taurus OX=9913 GN=APOH PE=4 SV=1

MLPPALVLLLGFLCHVAIAGRTCPKPDELPFSTVVPLKRTYEPGEQIVFSCQPGYVSRGG

IRRFTCPLTGLWPINTLKCMPRVCPFAGILENGTVRYTTFEYPNTISFSCHTGFYLKGAS

SAKCTEEGKWSPDLPVCAPITCPPPPIPKFASLSVYKPLAGNNSFYGSKAVFKCLPHHAM

FGNDTVTCTEHGNWTQLPECREVRCPFPSRPDNGFVNHPANPVLYYKDTATFGCHETYSL

DGPEEVECSKFGNWSAQPSCKASCKLSIKRATVIYEGERVAIQNKFKNGMLHGQKVSFFC

KNKEKKCSYTEDAQCIDGTIEIPKCFKVCSLVTVPQIVG

>tr|A0A3Q1LXR2|A0A3Q1LXR2_BOVIN Ras-related C3 botulinum toxin substrate 1 OS=Bos taurus OX=9913 GN=RAC1 PE=4 SV=1

MQAIKCVVVGDGAVGKTCLLISYTTNAFPGEYIPTVFDNYSANVMVDGKPVNLGLWDTAG

QEDYDRLRPLSYPQTVGETYGKEIPSRGKEKPIADVFLICFSLVSPASFENVRAKWYPEV

RHHCPNTPIILVGTKLDLRDDKDTIEKLKEKKLTPITYPQGLAMAKEIGAVKYLECSALT

QRGLKTVFDEAIRAVLCPPPVKKRKRKCLLL

>tr|A0A3Q1LY19|A0A3Q1LY19_BOVIN L-lactate dehydrogenase OS=Bos taurus OX=9913 PE=3 SV=1

MATLKDQLIQNLLKEEHVPQNKITIVGVGAVGMACAISILMKDLADEVALVDVMEDILKG

EMMDLQHGSLFLRTPKIVSGKDYNVTANSRLVIITAGARQQEGESRLNLVQRNVNIFKFI

IPNIVKYSPNCKLLVVSNPVDILTYVAWKISGFPKNRVIGSGCNLDSARFRYLMGERLGV

HPLSCHGWILGEHGDSSVPVWSGVNVAGVSLKNLHPELGTDADKEQWKAVHKQVVDSAYE

VIKLKGYTSWAIGLSVADLAESIMKNLRRVHPISTMIKGLYGIKEDVFLSVPCILGQNGI

SDVVKVTLTHEEEACLKKSADTLWGIQKELQF

>tr|A0A3Q1LYE7|A0A3Q1LYE7_BOVIN RAB1A, member RAS onco family OS=Bos taurus OX=9913 GN=RAB1A PE=1 SV=1

IDYLFKLLLIGDSGVGKSCLLLRFADDTYTESYISTIGVDFKIRTIELDGKTIKLQIWDT

AGQERFRTITSSYYRGAHGIIVVYDVTDQESFNNVKQWLQEIDRYASENVNKLLVGNKCD

LTTKKVVDYTTAKEFADSLGIPFLETSAKNATNVEQSFMTMAAEIKKRMGPGATAGGAEK

SNVKIQSTPVKQSGGGCC

>tr|A0A3Q1LYV8|A0A3Q1LYV8_BOVIN LIM and senescent cell antigen-like-containing domain protein OS=Bos taurus OX=9913 GN=LIMS1 PE=4 SV=1

MLGVASGMTNSNMANALANASCERCRGGFAPAEKIVNSNGELYHEQCFVCAQCFQQFPEG

LFYEFEGRKYCEHDFQMLFAPCCHQCGEFIIGRVIKAMNNSWHPECFRCDLCQEVLADIG

FVKNAGRHLCRPCHNREKARGLGKYICQKCHAIIDEQPLIFKNDPYHPDHFNCANCGKEL

TADARELKGELYCLPCHDKMGVPICGACRRPIEGRVVNAMGKQWHVEHFVCAKCEKPFLG

HRHYERKGLAYCETHYNQLFGDVCFHCNRVIEGDVVSALNKAWCVHCFACSTCSAKLTLK

DKFVEIDLKPVCKHCYEKMPEEFKRRLAKREREAKDKDKQKKKKPVCL

>tr|A0A3Q1LZS5|A0A3Q1LZS5_BOVIN Tetranectin OS=Bos taurus OX=9913 GN=CLEC3B PE=4 SV=1

MLARTSEGGPPAEGKSRAVTWMTTGRNRERQSLPGHQDAVSPKMLEELKTQLDSLAQEVA

LLKEQQALQTVCLKGTKVHMKCFLAFVQAKTFHEASEDCISRGGTLGTPQTGSENDALYE

YLRQSVGSEAEVWLGFNDMASEGSWVDMTGGHIAYKNWETEITAQPDGGKVENCATLSGA

ANGKWFDKRCRDKLPYVCQFAIV

>tr|A0A3Q1LZU8|A0A3Q1LZU8_BOVIN Glucosidase II alpha subunit OS=Bos taurus OX=9913 GN=GANAB PE=1 SV=1

MAAIAAVEARRRRSWTALVLACLGVCLGITFAVDRSNFKTCEESSFCKRQRSIRPGHSPY

RALLDSLQLGPDALTVHLINEVTKVVLVLELQGLQKNMTRIRIDELEPRRPRYRVPDVLV

ADPPTAGLSVSGRDDNSVELTVAEGPYKIILTARPFRLDLLEDRSLLLSVNARGLLNLEH

QRTPRVSQGSKEPAEGDGAQPEETPGDDNKPDETQGKPEHDEPGAWEETFKTHSDSKPYG

PTSVSLDFSLPGMEHVYGIPEHADNLRLKVTEGGEPYRLYNLDVFQYELYNRMALYGSVP

VLLAHSPLRDLGIFWLNAAETWVDISSNTAGKTLFGKMLDYLQGSGETPQTDVRWMSESG

IIDVFLMLGPSVFDVFRQYASLTGTQALPPLFSLGYHQSRWNYRDEADVLEVDQGFDDHN

LPCDVIWLDIEHADGKRYFTWDPSRFPQPRNMLEHLASKRRKLVAIVDPHIKVDSGYRVH

EELQNLGLYVKTRDGSDYEGWCWPGAAGYPDFTNPKMRAWWANMFHFDNYEGSAPNLYVW

NDMNEPSVFNGPEVTMLKDAQHYGGWEHRDVHNIYGLYVHMATADGLVLRSGGIERPFVL

SRAFFAGSQRFGAVWTGDNAAEWDHMKISIPMCLSLGLVGLSFCGADVGGFFKNPEPELL

VRWYQMGAYQPFFRAHAHLDTGRREPWLLPSQYHEIIRDALGQRYSLLPFWYTLFYQSHR

EGIPVMRPLWVHYPKDVTTFSIDDQFLLGDALLVHPVSDSEARGVQVYLPGQGEVWYDVQ

SYQKYHGPQTLYLPVTLSSIPVFQRGGTIVPRWMRVRRSSDCMKDDPITLFVALSLQGTA

QGELFLDDGHTFNYQTRHEFLLRRFSFSGNTLVSSSADPKGHFETPIWIERVVIIGAGKP

ATVVLQTKGSPESRLSFQHDPETSVLILRKPGVNVASDWTIHLR

>tr|A0A3Q1M0L3|A0A3Q1M0L3_BOVIN RNA helicase OS=Bos taurus OX=9913 GN=EIF4A2 PE=1 SV=1

MWNLELWKGGLVHPIYSFIFFFFNLQSNWNEIVDNFDDMNLKESLLRGIYAYGFEKPSAI

QQRAIIPCIKGYDVIAQAQSGTGKTATFAISILQQLEIEFKETQALVLAPTRELAQQIQK

VILALGDYMGATCHACIGGTNVRNEMQKLQAEAPHIVVGTPGRVFDMLNRRYLSPKWIKM

FVLDEADEMLSRGFKDQIYEIFQKLNTSIQVVLLSATMPTDVLEVTKKFMRDPIRILVKK

EELTLEGIKQFYINVEREEWKLDTLCDLYETLTITQAVIFLNTRRKVDWLTEKMHARDFT

VSALHGDMDQKERDVIMREFRSGSSRVLITTDLLARGIDVQQVSLVINYDLPTNRENYIH

RSR

>tr|A0A3Q1M0L5|A0A3Q1M0L5_BOVIN AbfB domain-containing protein OS=Bos taurus OX=9913 PE=4 SV=1

MRGTARPDCAVAIGRPLGEVVTLRFLEGSLNCSAGEMLLLWGRLVWRKMCGKPAGMVFSS

QANTLLVRQRLVRPGGGVLLRYSSQPALGAFHRGCDMQLFGPRGEISSPSMSPDGRNVGG

CRIFIDVAPWARIAIHALTVDSGTRAEGTDASYILIRDIHSLRTTAFRGQKTLYWESEGS

QAEMEFSQGFLEAHASLRGQYWTLHTRAG

>tr|A0A3Q1M0N0|A0A3Q1M0N0_BOVIN Collagen type XV alpha 1 chain OS=Bos taurus OX=9913 GN=COL15A1 PE=4 SV=1

MAPRYDSLPRAPAPAPYRPPHLSPSTTSCRRDAQCWRLLLLLSTSALLPGVTRTRSATES

ASRGPLDLTELVGVPLPSSVSFVAGYGGFPAYSFGPGANVGRPARTLIPSTFFRDFAISV

RVKPSSPQGGVLFAITDAFQKVIYLGLRLSGVEDGRQRVILYYTEPSSQVSREAAAFLVP

VMTHKWNYFAVVVQGEEVTLLVDCEEHGRVPFPRSSQALTFEPSAGIFVGNAGATGLERF

IGSIQQLIIHPDPRTPEEMCEAEDSSASGETSGLQETDGVAEILEAVTYTQAPSKEAEVE

PIHTPPTPSPASEDAELSGEPVPEGTKETSNLSADPHSSPEQGSGEILNDTLEAVQTVDG

SPTADTGSGDGVFLHVTEESPYTEDLAATAAAGEAKVPISTAWEAEASSVPTGGLTLSMP

IEDPGEGVTLGAENEEGSAATAAGEAEEPVSIAREAEASSVPTGGLTLPMPTQDPGEMVT

LSPISEEGSTTAAAATDVPLGTFEEEEASGVPTNDLAFITPTVASEQGVTSGPGDEDLAA

ATTEEPLTAAGADTLGSTPPEGPPLPLPSVAPESGAPPGEAEEGFPGPPGPAVPTEPTVE

VEAEGSGLGWGLDVSSGSGDLVHSEELLRGPPGPPGPPGLPGIPGKPGTDVFMGPPGSPG

EDGPTGEPGPPGPEGKPGLDGASGLPGIKGEKGARGPNGSVGEKGDPGNRGLPGPPGKTG

QVGAPGVMGPPGPPGPPGPPGPGCTTGLGFEDTEGSGSIRHLHEPISGPTASSGPKGEKG

DRGPKGDRGMDGASIVGPPGPRGPPGRIEVLSSSLTNITQGFMNFSDIPELVGPPGPEGM

PGLPGFPGPRGPKGDTGVPGFPGLKGEQGEKGEPGAILTGDIPLERLRGQKGEPGEHGAP

GPMGPKGPPGHKGEFGLPGRPGRPGLNGLKGAKGDRGVMIPGPPGLPGPPGPPGPPGAVI

NIKGAVFPVPVRPHCKTPVGTTYSGNSELITFHGVKGEKGSWGLPGSKGEKGDQGPQGPP

GPPVDPAYLRHFLNSLKGENGDRGIKGEKGDSNSGFSGFSVSGPPGLPGSPGLVGQKGEA

VVGPQGPPGAPGLPGPPGFGRPGSPGPPGPPGPPGPPAILGAAVALPGPPGPPGQPGLPG

SRNLVTTFSNMDDMLQKAHLVIEGTFIYLKDSTEFFIRVRDGWKKLQLGELIPIPDDSPP

PPALSSNPHQPQLSLTSISNVNYGRPALHLVALNTPFSGDIRADFQCFQQARAAGLLSTY

RAFLSSHLQDLSTVVRKAERYSLPIVNLKGQVLFNNWDSIFSGHGGQFNTHIPIYSFDGR

DVMTDPSWPQKVIWHGSSTHGVRLVDQYCEAWRTADMAVMGLASPLNTGKILDQKAYSCA

NRLIVLCIENSFMTDARK

>tr|A0A3Q1M0R0|A0A3Q1M0R0_BOVIN Adhesion G protein-coupled receptor E5 OS=Bos taurus OX=9913 GN=ADGRE5 PE=4 SV=1

MPRTHFLLEQQTETQSPSLGGAESPLFLVCVTLGTASALPLLCFLLTLSEVGSQNSKACA

LPCPPNSSCVNGTACRCAPGFISFSGEIFTDPLESCDDINECGPPSPVDCGSSADCQNTE

GGYYCTCSPGYEPVSGAMIFRNESENTCRDVDECQHRPRVCKGRSVCINTEGSYTCQCPP

GLEFSPEDPRHCTDVNECTSGKKPCHSSTHCLNSVGSYECRCRPGWKPIAGSPNGPNNTV

CEDVDECSSGQHQCHNSTVCFNTVGSYTCHCREGWEPKHGLKNKQKDTICKEISFPAWTA

PPGIKSRSLSAFFERVQKMSRDFKPAMAKKSMQDLVGSVDDLLKNSGDLESLDQSSKHVT

VTHLLSGLEQILRTLAKAMPKGSFTYRSLDNTELSLVVQEQGKGNVTVGQSHARMLLDWA

VAAAAEESGPTVVGILSSQNMKKLLANASLKLDSEKLKETYKSPVRGAKVTLLSAVSSVF

LSNTNTEKLDSNVSFAFALHEQPELKPRQELICAFWKKDSNGNGSWATTGCWKMGRGNGS

ITCQCSHLSSFAILMAHYDVEDPKLALITKVGLALSLACLLLCILTFLLVRPIQGSRTTV

HLHLCICLFVGSAIFLAGIENEGGEVGTRCRLVAVLLHYCFLAAFCWMSLEGVELYFLVV

RVFQGQGLRKLWLCLIGYGVPLIIVGISAGAYSKGYGREKFCWLNFEGGFLWSFVGPVTF

IVLGNAIIFVITVWKLTQKFSEINPDIKKLKKARVLTITAIAQLFVLGCTWVFGLLLFDP

ESWVLSYIFSILNCLQGFFLFVLYCLLNKKVGCRPGCGRSWAPPPVLGPVLP

>tr|A0A3Q1M0U5|A0A3Q1M0U5_BOVIN Receptor protein-tyrosine kinase OS=Bos taurus OX=9913 GN=TIE1 PE=3 SV=1

MVWLEPPLLLPIFFLASHVGAAVDLTLLADLRLTEPQRFFLTCVSGEAGAGRGSDAWGPP

LLLEKDDRIVRTPRPWQPPHIARNGSSRVTVRGFSQPSDLVGVFSCVGGAGTRRTRVLYV

HNSPGAHLLPDKVTHTVNKGDTAVLSARVRKEKQTDVIWKSNGSYFYTLDRHEAQDGQFL

LQLPNVQPSSSGIYSATYLEASPLGSAFFRLIVRGCEAGRWGQDCTKECPGCLHGGVCHD

QDGECVCPPGFTGTRCEQACREGRFGQSCQEQCPGTSGCRGLTFCLPDPYGCSCGSGWRG

SQCQEACAPGRFGADCHLQCQCQNGGTCDRFSGCVCPSGWHGMHCEKSDRIPQILDMVSE

LEFNLDTMPRINCAAAGNPFPVRGSMELRKPDGTVLLSTKAIVEPDRTTAEFEVPRLALG

DSGLWECRVSTSGGQDSRRFRINVKVPPVPLTAPRLLAKQSRQLVVSPLVSFSGDGPIAS

VRLHYRPQDSTMAWSTIVVDPSENVTLMNLRPKTGYSVRVQLSRPGEGGEGAWGPPTLMT

TDCPEPLLKPWLEGWHVEGPDRLRVSWSLPPVPGPLVGDGFLLRLWDGARGQERRENVSS

PQARTALLTGLTPGTYYQLDVRLYHCTLLGPASPAARVLLPPSGPPAPRHLHAQALSDSE

IQLMWQRPEAAAGPISKYIVEVQVAGGSGDPLWMDVDRPEETSTIVRGLNASTRYLFRVR

ASVQGPGDWSNVVEQSTLGNGLQIEGPVQEIHAAEEGLDQQLVLAVVGSVSATCLTILAA

LLTLACIRKSCLHRRRTFTYQSGSGEETILQFSSGTLTLTRRPKPQPEPLNYPVLEWEDI

TFEDLIGEGNFGQVIRAMIKKDGLKMNAAIKMLKEYASENDHRDFAGELEVLCKLGHHPN

IINLLGACENRGYLYIAIEYAPYGNLLDFLRKSRVLETDPAFAREHGTASTLSSRQLLRF

ASDAANGMQYLSEKQFIHRDLAARNVLVGENLASKIADFGLSRGEEVYVKKTMGRLPVRW

MAIESLNYSVYTTKSDVWSFGVLLWEIVSLGGTPYCGMTCAELYEKLPQGYRMEQPRNCD

DEVYELMRQCWRDRPYERPPFAQIALQLGRMLEARKAYVNMSLFENFTYAGIDATAEEA

>tr|A0A3Q1M0V5|A0A3Q1M0V5_BOVIN Phosphopyruvate hydratase OS=Bos taurus OX=9913 GN=ENO3 PE=1 SV=1

MEGPAHVQPPFAPLPGQGPGHGLSPPSADRARPQPSPSLRVQAGPVPRTVQASQYRDVDE

GLAGDGGPDGGRVLIAHDGCVLTAMAMQKIFAREILDSRGNPTVEVDLHTAKGRFRAAVP

SGASTGIYEALELRDGDKSRYLGKGVLKAVEHINKTLGPALLEKKLSVVDQEKVDKFMIE

LDGTENKSKFGANAILGVSLAVCKAGAAEKGVPLYRHIADLAGNPELILPVPAFNVINGG

SHAGNKLAMQEFMILPVGASSFREAMRIGAEVYHHLKGVIKAKYGKDATNVGDEGGFAPN

ILENNEALELLKTAIQAAGYPDKVVIGMDVAASEFYRNGKYDLDFKSPDDPARHISGEKL

GELYKNFIKNYPVVSIEDPFDQDDWATWTSFLSGVNIQIVGDDLTVTNPKRIAQAVEKKA

CNCLLLKVNQIGSVTESIQACKLAQSNGWGVMVSHRSGETEDTFIADLVVGLCTGQIKTG

APCRSERLAKYNQLMRYRGHSEPGHQELRAFGLEYHSPDHVCLPGLRRLLGTRLSLLDAS

SVTRRPSERLEAPGVHRTDLGLQALPPEIKHWCQPS

>tr|A0A3Q1M0W4|A0A3Q1M0W4_BOVIN Transforming growth factor beta receptor 3 OS=Bos taurus OX=9913 GN=TGFBR3 PE=4 SV=1

MGHTFGEIKMTSHCVIVMFALMSSCLAAAGPEPRAQCELSPVNASHPVQALMESFTALSG

CASRGTVGLPQEVHVLNLRAADQGPGQPQREVTLHLNPISSVHIHHKPVVFLLNSPQPLV

WHLKTERLAAGVSRLFLVSEGSLVHFSSRNFSLSAETEERSFPHGNEHLLNWARKEYGAV

TSFTELKIARNIYIKVGEDQVFPPTCNIGKNFLSLNYLAEYLQPKAAEGCVMSSQPQDKE

VHIIELITPNSNPYSAFQVDIIIDIRPSRKDPEVVKNLILILKCKKSVNWVIKSFDIKGN

LKAIAPNSIGFGKESERSMIMTKSKRDDIPSTQESLVKWALDNGYSPVTSYTVAPMANRF

HLRLESNEEMRDEEVHTVPPELRILLGPGTLPALDNPPIRGGGSRNGGFPFPFPDISRRG

RKEGGEDGILRPKDPVIPGILFPDPREPEEVQGSTDVALSVKCDNEKMTVAVDKDSFQAS

GYSGMELTLLDPTCKAKTNDTHFILESPLNGCGTRLRRAAPDGVVYYNSIVIQIPPSGDS

SGWPDGYEDLESGDNGFPGDMDEGDTSFFSRLEIVVFNCSLRQVGNPRTFQDEPNRNVTF

NMELYNTDLFLVPSQGVFSVAENGHVYVEVSVTKADQELGFAIQTCFISPYSNPDRMSDY

TIIENICPKDESVKFYNPKRVHFPIPQAEIDKKRFSFVFKPVFNTSLLFLQCELTLCTKK

EKDPQKLPKCVLPDEACTSLDASMIWAMMQNKKTFTRPLAVIHHEVPFKEIFHGLDTLTV

MGIAFAAFVIGALLTGALWYIYSHTGETAGRQQVPTSPPASENSSAAHSIGSTQSTPCSS

SSTA

>tr|A0A3Q1M119|A0A3Q1M119_BOVIN 14-3-3 protein epsilon OS=Bos taurus OX=9913 GN=YWHAE PE=1 SV=1

MDDREDLVYQAKLAEQAERYDEMVESMKKVAGMDVELTVEERNLLSVAYKNVIGARRASW

RIISSIEQKEENKGGEDKLKMIREYRQMVETELKLICCDILDVLDKHLIPAANTGESKVF

YYKMKGDYHRYLAEFATGNDRKEAAENSLVAYKAASDIAMTELPPTHPIRLGLALNFSVF

YYEILNSPDRACRLAKAAFDDAIAELDTLSEESYKDSTLIMQLLRDNLTLWTSDMQGDDS

>tr|A0A3Q1M168|A0A3Q1M168_BOVIN Alpha-1,4 glucan phosphorylase OS=Bos taurus OX=9913 GN=PYGL PE=1 SV=1

MAKPLTDQEKRRQISIRGIVGVENVAELKKGFNRHLHFTLVKDRNVATPRDYFFALAHTV

RDHLVGRWIRTQQYYYEKCPKRVYYLSLEFYMGRTLQNTMINLGLQNACDEAIYQLGLDM

EELEEIEEDAGLGNGGLGRLAACFLDSMATLGLAAYGYGIRYEYGIFNQKIRDGWQIEEA

DDWLRHGNPWEKARPEFMLPVHFYGRVEHTEAGTKWTDTQVVLALPYDTPVPGYLNNTVN

TMRLWSARAPNDFNLRDFNVGDYIQAVLDRNLAENISRVLYPNDNFFEGKELRLKQEYFV

VAATLQDVIRRFKASKFDSSNSTKTAFDAFPDQVAIQLNDTHPSLAIPELMRIFVDIEKL

PWSKAWEITQKTFAYTNHTVLPEALERWPVELVEKLLPRHLQIIYEINQKHLDKIAALFP

KDVDRLRRMSLIEEEGGKRINMAHLCIVGSHAVNGVAKIHSDIVKTQVFKDFSELEPDKF

QNKTNGITPRRWLLLCNPGLAELIAEKIGEDYVKDLSQLTKLNSFLGDDIFLREISNVKQ

ENKLKFSQFLEKEYKVKINPSSMFDVQVKRIHEYKRQLLNCLHVVTMYNRIKKDPKKLFV

PRTVIIGGKAAPGYYMAKLIIKLITSVAEVVNNDPVVGSKLKLIFLENYRVSLAEKVIPA

TDLSEQISTAGTEASGTGNMKFMLNGALTIGTMDGANVEMAEEAGEENLFIFGMRIEDVA

ALDKKGYEAKEYYEALPELKLAIDQIDKGFFSPKQPDLFKDLVNMLFYHDRFKVFADYEA

YVKCQEKVSQLYMNPKAWNIMVLKNIAASGKFSSDRTIKEYARDIWNMEPSDIKISLSSD

PSGGANKANGKASGNGASR

>tr|A0A3Q1M1U2|A0A3Q1M1U2_BOVIN Serine/threonine-protein phosphatase 2A 65 kDa regulatory subunit A alpha isoform OS=Bos taurus OX=9913 GN=PPP2R1A PE=4 SV=1

MAAADGDDSLYPIAVLIDELRNEDVQLRLNSIKKLSTIALALGVERTRSELLPFLTDTIY

DEDEVLLALAEQLGTFTTLVGGPEYVHCLLPPLESLATVEETVVRDKAVESLRAISHEHS

PSDLEAHFVPLVKRLAGGDWFTSRTSACGLFSVCYPRVSSAVKAELRQYFRNLCSDDTPM

VRRAAASKLGEFAKVLELDNVKSEIIPMFSNLASDEQDSVRLLAVEACVNIAQLLPQEDL

EALVMPTLRQAAEDKSWRVRYMVADKFTELQKAVGPEITKTDLVPAFQNLMKDCEAEVRA

AASHKVKEFCENLSADCRENVIMTQILPCIKELVSDANQHVKSALASVIMGLSPILGKDS

TIEHLLPLFLAQLKDECPEVRLNIISNLDCVNEVIGIRQLSQSLLPAIVELAEDAKWRVR

LAIIEYMPLLAGQLVRALPLPSPPPTVYAIREAATSNLKKLVEKFGKEWAHATIIPKVLA

MSGDPNYLHRMTTLFCINVLSEVCGQDITTKHMLPTVLRMAGDPVANVRFNVAKSLQKIG

PILDNSTLQSEVKPVLEKLTQDQDVDVKYFAQEALTVLSLA

>tr|A0A3Q1M1Z2|A0A3Q1M1Z2_BOVIN Tubulin alpha chain OS=Bos taurus OX=9913 GN=TUBA4A PE=3 SV=1

MRECISVHVGQAGVQMGNACWELYCLEHGIQPDGQMPSDKTIGGGDDSFTTFFCETGAGK

HVPRAVFVDLEPTVIDEIRNGPYRQLFHPEQLITGKEDAANNYARGHYTIGKEIIDPVLD

RIRKLSDQCTGLQGFLVFHSFGGGTGSGFTSLLMERLSVDYGKKSKLEFSIYPAPQVSTA

VVEPYNSILTTHTTLEHSDCAFMVDNEAIYDICRRNLDIERPTYTNLNRLISQIVSSITA

SLRFDGALNVDLTEFQTNLVPYPRIHFPLATYAPVISAEKAYHEQLSVAEITNACFEPAN

QMVKCDPRHGKYMACCLLYRGDVVPKDVNAAIAAIKTKRSIQFVDWCPTGFKVGINYQPP

TVVPGGDLAKVQRAVCMLSNTTAIAEAWARLDHKFDLMYAKRAFVHCKCMEPPTDLWA

>tr|A0A3Q1M2A8|A0A3Q1M2A8_BOVIN Complement factor I OS=Bos taurus OX=9913 GN=CFI PE=1 SV=1

MKLGHVILLLLCFYLSFCEDNFRKRGKSKAVKKSEAHHAPEASLSKETEASSEVKPTSTQ

DTSQKDFVDKKCLTEKHTHLSCNKVFCQPWQKCIDGTCLCKLPYQCPKNGTRVCSTNGKS

YSTYCQQKSFECYRPEAKFLKSGACTGGGQFSVSLSNGKQDSEGIVAVKLADLDTKMFVC

GDSWSITEANVACIDRGFQLGALDTHRRDPDPNSAECLHVRCRGLETSLAECTFTKGVHN

SEGLAGVVCYTESAAPPKKDSFQCVNGKRIPQKKACDGVNDCVDKSDELCCKETGHPEIK

EAAEMLTADMDAERKFTKSFLPKLSCGVKNNMHIRRKRVVGGKPAKMGEFPWQMAIKEGD

KIHCGGIYIGGCWILTAAHCVRISRMHRYQIWTSFTDWLRPGFQTVVHSVNRIIIHENYN

GTTYQNDIALIEMKKRPNEKECVLSKSIPACVPWSPYLFQPNDKCIVSGWGREKDNQKVY

SLRWGEVHLINNCSEFYPGRYFEKEMQCAGTDDGSIDACKGDSGGPLVCQDVNNVTYVWG

VVSWGENCGKSEFPGVYTKVANYFDWISQHVGRSLISQHNI

>tr|A0A3Q1M2H4|A0A3Q1M2H4_BOVIN Aldehyde dehydrogenase family 16 member A1 OS=Bos taurus OX=9913 GN=ALDH16A1 PE=3 SV=1

MAATRTASRACEIFTTLEYGPAPESHACALAWLDTQDRHLGHYVNGQWLKPEHRSSVPCQ

DPITGENLASCLQAQSEDVAAAVEAARASLENWSTQPGAIRAQHLTRLAKVIQKHQRLLW

TLESLVTGRAVREVRDRDVPLAQQLLQYHAVQAHTQEEALAGWEPMGVGSLDSSVLNYLL

PPGCTVVVLVPPASPTPLLLAQLAGELGPFPGILNVISGPASLGPVLAAQPGVQKVAFCG

AIEEGRALRRTLAGWVPELGLALGAESLLLLTEVADVDSAVEGIVDAAWSDRSPGGLRLL

IQEAVWDETMRRLQERMGRLRCGHGLDGAVDMGARGAAARDLAQRYVSEAQSQGAQVFQA

GSEPSDSPFFPPTLVSDLPPASPCTQAEVPWPLVVASPFRTAKEALAVANGTPRGGSASV

WSERLGQALELAYGLQVGTVWINAHGLRDPAVPMGGCKESGSSWHGGQDVSNHLPAWGEG

GFWAGAALTHPHPSSSCSPAPPYGLFVGGRFQAPGARSSRPIRDSQGSLQGYVAEGGAKD

IRGAVEAAHQAAPGWMSQSPAARAALLWALAAALQRREPNLVSRLERHGVELKVAKAEVE

LSVKRLRAWGARVQAQGCALQVAELRGPVLRLREPLGVLAIVCPDEWPLLAFVSLLAPAL

AHGNTVVLVPSGACPIPALEVCQPVDMLTMPLCLSPHQGSQFVEWASAGNLKPVWVNRGC

PRAWDQEAEGAGPELGRRAARTKALWLPMGD

>tr|A0A3Q1M3A4|A0A3Q1M3A4_BOVIN Complement C3 OS=Bos taurus OX=9913 GN=LOC528040 PE=4 SV=1

MDVPWGLGLLLLLLGIPIAQAEPLYILVTPRVLRIGSPETIHVEAHSDSSEPLSHPLEVN

LSVWDFPMKNTRVARRELVLSKENHFMDQASVTIPEDLVYPPKPGMQYVIIEANWAPTSV

SSSMNKLVLVAPHAGYIFIQMDKTIYTPEQSVQYRVYTVNHRMDPVSRTFTLDIKNPEGI

AVISKDLLPDNGVYIDSFTLPERISIGTWTIEASYQTAPKQKFKTGFEVKEYVLPSFEVQ

LTPNKTFFYLRDEVLGVNIQARYIFNKPVDGHALAIFGVKQDSRRIPIQSSLQRVEISQG

HGHISLQKDTLMAAFQGSEEDFIGASIFVNVTVFSSGGEMVQTEISGVKIVRSPYNIKFI

KTPQYFKPGMPFSFRVFVSNPDGSPASKVLVSCTNVKVHTTPRGEATLVINTEANLKELT

IQVKTEAPIQPEEQASASMTARPYSTQDESGNFLHIDVKALSTEVGSNLQLNLNTNRDSS

VSNKITRFTILVLSKGQIVHAKELKNHGSVFASTIIDVTSKMLPSFRILAFYLLPKGTGQ

DPELVADSILIDVNDKCQEKLKIGLQNEAYVQPIQPSSLVALKVTGDAEATVGLVAVDKA

VHILNSKHKFTQKKIWDTVEEHDIGCTAGSGKDRLAVFKDAGLDMKMSTGMDTLASTDWH

CPPSPSPSHRRRRSLKRLETKRKAVNKFKTELEQKCCEAGLRENPVGLSCKERIQHVRHG

PVCITAFLSCCQLSETLTREAREEQLLLGTTDEDDDLDDFFLEDEPVRSVFPESWFWKTI

TLPKTTQGISHYTTHVTMPDSITTWQFVAVSIKTGQGLCVSDPFELTVMKPFFVDLKLPF

SVIRNEQVQIQAVLYNFLQQSVKVRVEFPHKESLCSAAKPDAPSRRIVAVPPFSSKVVPF

VLLPLEIGKVDVEVKARGSVVQDHVRKTLLVQAGGQIEQISQSFLLNPQGQTKTQLVPKQ

EFLNKIPNTEADVFVSVQGDILGETILGTLTPSETWRLLRIPSGCPEQTLSSLTPVVILT

RYLDSTGQWNKVGVELREQVMKNLARGYSRMLTHRSEDGSYHTSKGNPGSTWLTSYVFRV

YALAYPTMTISALSLDSVCNIANWIITHRQRTHGNFVEESPVVMWSMQGGYHGSEADISL

TALVLIALNEGKELCSQKIPSLADSMKRAGDFLEKKLPHIRTTFAMAITSYALALIRSPR

ANDYLDSFASKNKTHWPVGLDDLDLDDSLYTIEATAYALMQKLELGRRNETHAIANWLLK

KRQLGGGFQSTQTTVVAIEALTRFREAVPFEGVQDLHIQIKSSKKALHVEWVIDEKNAYQ

LRSAKFSAEEELQIKASGTGRGTISILTVYHRSPEFLENTCKQYHLNVTLNENQKENKKG

EATFQLRMETRFQGHRDATMTIMEISLLTGFYPNQDDLKQLTSEVERYAFQYETKMNSSD

STVVLYLEKLSHKEDTVLGFRVHRMLKAEFLQAAQVTVYDYYEPSRRCTSFYNLPTEHAS

LRKICDKDVCRCAEEQCPSPKKDSNHLSQEELQTAACEAGVDFVYKASLESVETSDSNPY

IYYNMKLQAIIKSGMSKVAEEMVWEPTPGVPKWGQARPMQVNTHWLSSPSGTDSAKPLAV

KKFVTHTTCQDSLGLQEHETYLIMGQISDLWRVKSECVAGGLLLPWGLRPGPLSLPQASP

WCQNFYCTRQQESWVSGLP

>tr|A0A3Q1M3K7|A0A3Q1M3K7_BOVIN Ras-related protein Rab-7a OS=Bos taurus OX=9913 GN=RAB7A PE=1 SV=1

MTSRKKVLLKVIILGDSGVGKTSLMNQYVNKKFSNQYKATIGADFLTKEVMVDDRLVTMQ

IWDTAGQERFQSLGVAFYRGADCCVLVFDVTAPNTFKTLDSWRDEFLIQASPRDPENFPF

VVLGNKIDLENRQVATKRAQAWCYSKNNIPYFETSAKEAINVEQAFQTIARNALKQVGVC

SIGASGFGPSACTSPRGLSVQPSAPPGPEPRPGRLGLHLSPQALCAARGGGGPLRAALSR

RP

>tr|A0A3Q1M3N0|A0A3Q1M3N0_BOVIN Apolipoprotein A-IV OS=Bos taurus OX=9913 GN=APOA4 PE=3 SV=1

MYHCAHFTGRIWCSKRLSQPSEEPRMFLKAVVLSLALVAVTGAEAEVNADQVATVIWDYF

SQLGNNAKKAVEHIQKSELTQQLNTLFQDKLGEVSTYTDDLQKKLVPFATELHERLTKDS

EKLKEEIRKELEDLRARLLPHATEVSQKIGDNVRELQQRLGPYAEELRTQVDTQAQQLRR

QLTPYAERMEKVMRQNLDQLQASLAPYAEELQATVNQRVEELKGRLTPYADQLQTKIEEN

VEELRRSLAPYAQDVQGKLNHQLEGLAFQMKKHAEELKAKISAKAEELRQGLVPLVNSVH

GSQLGNAEDLQKSLAELSSRLDQQVEDFRRTVGPYGETFNKAMVQQLDTLRQKLGPLAGD

VEDHLSFLEKDLRDKNGV

>tr|A0A3Q1M478|A0A3Q1M478_BOVIN C-1-tetrahydrofolate synthase, cytoplasmic OS=Bos taurus OX=9913 GN=MTHFD1 PE=1 SV=1

FVPCMILNAVNLQIKERLKNQVIQMKEEVPGFIPGLAILQVGNRDDSNLYINMKLKAAEE

IGIKATHIKLPRTATESEVLKCITSLNEDLTVHGFIVQLPLDSENPINTETLVNAIAPEK

DVDGLNSISAGKLARGDLSNCFIPCTPKGCLELIKETGVQIAGRHAVVVGRSKIVGAPMH

DLLLWNHATVTTCHSKTANLKEEISKGDILVVAAGQPEMVKGEWIKPGAIVIDCGINYVT

DDTKPNGKKIVGDVAYTEAKERASFITPVPGGVGPMTVAMLMQSTVESAKRFLENFKPGK

WIIQYNKLNLKTPVPSDIDISRSYKPKPIGNLAREVGLRTEEVELYGETKAKVLLSALER

LKHQPDGKYVVVTGITPTPLGEGKSTTTIGLVQALGTHLHQNVFACVRQPSQGPTFGIKG

GAAGGGYSQVIPMEEFNLHLTGDIHAITAANNLVAAAIDARMFHEETQTDKALFNRLVPS

VNGVRKFSDIQIRRLRRLGIEKTDPATLTDEEINRFARLDIDPETITWQRVLDTNDRFLR

KITIGQAPTEKGHSRTAQFDISVASEIMAVLALTSSLEDMRERLGKMVVASSKKGEPIST

EDLGVSGALTVLMKDAIKPNLMQTLEGTPVFVHAGPFANIAHGNSSIIADRIALKLVGPE

GFVVTEAGFGADIGMEKFFNIKCRYSGLRPHVVVLVATVRALKMHGGGPTVRIRDWKDLP

LLFLQDLELVGKGFSNLKKQIENARMFGVPVVVAVNAFKTDTEAELDLVSRLAKEHGAFD

AVKCTHWAEGGKGALALAEAVQRAAAAPSSFQLLYDLKLPVEDKIRIIAQKIYGADNIEL

LPEAQHKAEVYTKQGFGNLPICMAKTHLSLSHNPELKGVPTGFVLPIRDIRASVGAGFLY

PLVGTMSTMPGLPTRPCFYDIDLDPETEQVNGLF

>tr|A0A3Q1M4K3|A0A3Q1M4K3_BOVIN Ubiquitin-like domain-containing protein OS=Bos taurus OX=9913 GN=LOC101902760 PE=4 SV=1

MQIFVKTLTGKTITLEVEPSDTIENVKAKIQDKEGIPPDQQRLILAGKQLEDGRTLSDYN

IQKESTLHLVLRLRGGC

>tr|A0A3Q1M4L0|A0A3Q1M4L0_BOVIN HipN domain-containing protein OS=Bos taurus OX=9913 PE=3 SV=1

MDPRKVSELRAFVKMCKQDLSVVHTEEMRFLGEWVESMGGKLPPAAHETKLEENTKEENT

DSKKAEENIKTDEPSSEERKKKKEMGDENVEITEEMMDQANDKKVAAIDALNDGELQKAI

DLFTDAIKLNPRLAILYAKRASVFIKLQKPNAAIRDCDRAIEINPDSAQPYKWRGKAHRL

LGHWEEAARDLALSWTRLSN

>tr|A0A3Q1M4P8|A0A3Q1M4P8_BOVIN Collagen type XV alpha 1 chain OS=Bos taurus OX=9913 GN=COL15A1 PE=4 SV=1

SCRRDAQCWRLLLLLSTSALLPGVTRTRSATESASRGPLDLTELVGVPLPSSVSFVAGYG

GFPAYSFGPGANVGRPARTLIPSTFFRDFAISVRVKPSSPQGGVLFAITDAFQKVIYLGL

RLSGVEDGRQRVILYYTEPSSQVSREAAAFLVPVMTHKWNYFAVVVQGEEVTLLVDCEEH

GRVPFPRSSQALTFEPSAGIFVGNAGATGLERFIGSIQQLIIHPDPRTPEEMCEAEDSSA

SGETSGLQETDGVAEILEAVTYTQAPSKEAEVEPIHTPPTPSPASEDAELSGEPVPEGTK

ETSNLSADPHSSPEQGSGEILNDTLEAVQTVDGSPTADTGSGDGVFLHVTEEVNLAATAA

AGEAKVPISTAWEAEASSVPTGGLTLSMPIEDPGEGVTLGAENEEGSAATAAGEAEEPVS

IAREAEASSVPTGGLTLPMPTQDPGEMVTLSPISEEGSTTAAAATDVPLGTFEEEEASGV

PTNDLAFITPTVASEQGVTSGPGDEDLAAATTEEPLTAAGADTLGSTPPEGPPLPLPSVA

PESGAPPVSEDFTSHRGQSHGKGEKGEAEEGFPGPPGPAVPTEPTVEVEAEGSGLGWGLD

VSSGSGDLVHSEELLRGPPGPPGPPGLPGIPGKPGTDVFMGPPGSPGEDGPTGEPGPPGP

EGKPGLDGASGLPGIKGEKGARGPNGSVGEKGDPGNRGLPGPPGKTGQVGAPGVMGPPGP

PGPPGPPGPGCTTGLGFEDTEGSGSIRHLHEPISGPTASSGPKGEKGDRGPKGDRGMDGA

SIVGPPGPRGPPGRIEVLSSSLTNITQGFMNFSDIPELVGPPGPEGMPGLPGFPGPRGPK

GDTGVPGFPGLKGEQGEKGEPGAILTGDIPLERLRGQKGEPGEHGAPGPMGPKGPPGHKG

EFGLPGRPGRPGLNGLKGAKGDRGVMIPGPPGLPGPPGPPGPPGAVINIKGAVFPVPVRP

HCKTPVGTTYSGNSELITFHGVKGEKGSWGLPGSKGEKGDQGPQGPPGPPVDPAYLRHFL

NSLKGENGDRGIKGEKGDSNSGFSGFSVSGPPGLPGSPGLVGQKGEAVVGPQGPPGAPGL

PGPPGFGRPGSPGPPGPPGPPGPPAILGAAVALPGPPGPPGQPGLPGSRNLVTTFSNMDD

MLQKAHLVIEGTFIYLKDSTEFFIRVRDGWKKLQLGELIPIPDDSPPPPALSSNPHQPQL

SLTSISNVNYGRPALHLVALNTPFSGDIRADFQCFQQARAAGLLSTYRAFLSSHLQDLST

VVRKAERYSLPIVNLKGQVLFNNWDSIFSGHGGQFNTHIPIYSFDGRDVMTDPSWPQKVI

WHGSSTHGVRLVDQYCEAWRTADMAVMGLASPLNTGKILDQKAYSCANRLIVLCIENSFM

TDARK

>tr|A0A3Q1M558|A0A3Q1M558_BOVIN Actin, alpha cardiac muscle 1 OS=Bos taurus OX=9913 GN=ACTC1 PE=3 SV=1

MCDDEETTALVCDNGSGLVKAGFAGDDAPRAVFPSIVGRPRHQGVMVGMGQKDSYVGDEA

QSKRGILTLKYPIEHGIITNWDDMEKIWHHTFYNELRVAPEEHPTLLTEAPLNPKANREK

MTQIMFETFNVPAMYVAIQAVLSLYASGRTTGIVLDSGDGVTHNVPIYEGYALPHAIMRL

DLAGRDLTDYLMKILTERGYSFVTTAEREIVRDIKEKLCYVALDFENEMATAASSSSLEK
[truncated: 499,850 more chars]
